# What do users and their aiding professionals want from future devices in upper limb prosthetics? A focus group study

**DOI:** 10.1371/journal.pone.0295516

**Published:** 2023-12-29

**Authors:** Ann-Kathrin Einfeldt, Franziska Rebmann, Dawei Yao, Christina Stukenborg-Colsmann, Christof Hurschler, Henning Windhagen, Eike Jakubowitz

**Affiliations:** 1 Laboratory for Biomechanics and Biomaterials, Department of Orthopedic Surgery, Hannover Medical School, Hannover, Germany; 2 Department of Orthopedic Surgery, Hannover Medical School, Hannover, Germany; Air University, PAKISTAN

## Abstract

**Background:**

High rejection rates of upper limb prosthetics indicate that current prosthetic devices only partially meet user demands. This study therefore investigated the benefits and challenges with current prostheses, associated services and potential areas for improvement from the perspective of upper limb prosthesis users and various professionals working in the field of upper limb and hand prosthetics.

**Methods and findings:**

Seven different focus group discussions were conducted with 32 participants. Participants were grouped by prosthesis type, if they were prosthesis users, or professionals. All focus group discussions were transcribed verbatim, and a summarizing content analysis was performed. Three main topic areas to be addressed emerged from the interviews: 1. a properly functioning prosthesis, 2. the infrastructure, and 3. users’ psychological and physical prerequisites. The interaction between a well-functioning prosthesis and a well-developed infrastructure was shown to be important for successful use.

**Conclusions:**

Our study raises many of the same issues that have been reported in previous qualitative studies, dating back over several decades. This study underlines the need to include users and professionals in the future development of prosthetic devices.

## Introduction

According to the German Federal Statistical Office, 225 amputations and exarticulations of the upper extremity were performed in Germany in 2019 [1]. Cases of amputations of the hand were significantly higher with 5650 patients affected [1]. In 2014, these numbers were 152 and 4047, respectively, which means there is an increasing trend. Even though various treatment options are available in upper limb prosthetics, high rejection rates indicate that current prosthetic devices meet the needs of users only partially or not at all [2, 3]. Therefore, users and professionals should be included in the research and development process for new prostheses. Many quantitative studies examining the opinions of prosthesis users (PUs) towards their prosthesis and their use of it have been performed [4, 5]. Qualitative studies have the potential to highlight patients’ subjective needs and perspectives [6]. Detailed research and evaluations in qualitative studies show that these studies investigate users’ and experts’ opinions with limitations regarding specific requirements for prosthesis use or care [2, 7–10]. Therefore, this study aimed to investigate, in an open-ended manner, which characteristics and properties of myoelectric prostheses could be advantageous from the perspective of upper limb prosthesis users and professionals working in upper limb and hand prosthetics. The results will be used to facilitate future developments in this field to support the design of appropriate products and better meet user needs.

The study is part of the *SoftPro* project *(Synergy-based Opensource Foundations and Technologies for Prosthetics and Rehabilitation)*. Its purpose is to integrate patient needs and expert opinions in prosthetic care into the implementation process of transforming the PISA/IIT SoftHand 2 [11], initially developed as an end effector in robotics, into a version for use in upper limb prosthetics (SoftHand Pro).

Consequently, this study addresses the following research questions about upper limb prostheses:

1. What are the most important requirements for users and professionals?
2. Have technical innovations in recent years changed the requirements of users and professionals?
3. Which unfulfilled requirements of past prosthetic devices can be addressed with future prosthetic devices?

## Methods

Seven focus group (FG) discussions were conducted between 26.10.2017 and 05.12.2017 with 32 participants from four German states (Baden-Württemberg, Berlin, Lower Saxony, and North Rhine-Westphalia). All methods were reviewed and approved by the local ethics committee of Hannover Medical School (No: 3531).

### Participants

**Fig 1:**
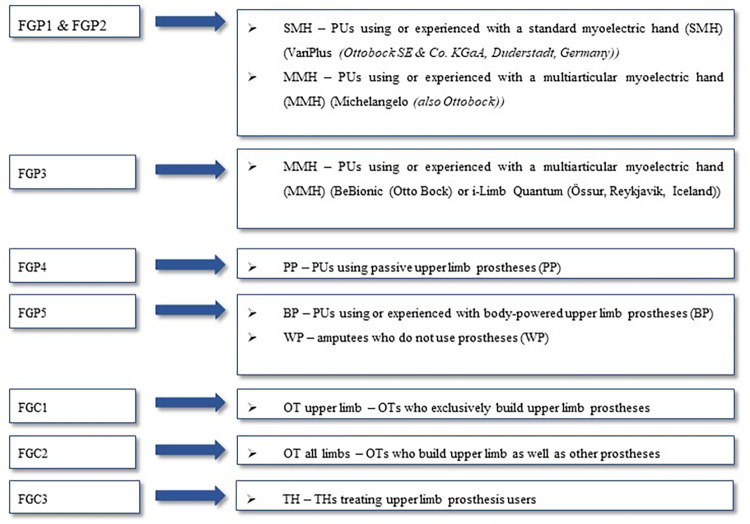
Division of the participants into the seven different focus groups (FGP = Focus Group Patient, FGC = Focus Group Caretaker) and characterization of participants in each group.

Participants included PUs, orthopedic technicians (OTs), and therapists (THs), including physiotherapists (PHTs) as well as occupational therapists (OCTs) recruited using predefined inclusion and exclusion criteria (Table 1). PUs were recruited through different medical care providers in Lower Saxony. OTs were recruited by contacting orthotics and prosthetics providers in Baden-Württemberg and Hannover. PHTs and OCTs were recruited via a German directory, including specialized therapists for upper limb amputees and their prosthetic care. According to Pollock and Böhm [12], each focus group should consist of at least three to a maximum of six participants. Participants were not mixed but instead assigned according to specific target groups (PUs and different professionals) to enable a holistic processing of the research questions and achieve FGs that were as consistent as possible. A more differentiated focus on the prosthetic hand was realized by further consolidating the PU group into subgroups based on the control aspects and kinematics of their prosthetic end effectors (Fig 1).

**Table 1:** In- and exclusion criteria.

PUs	OTs, PHTs, and OCTs
Inclusion criteria • one- or two-sided major upper limb amputation (including dysmelia) • completed rehabilitation • age ≥ 16 years • language: German • written informed consent	Inclusion criteria • ≥ 5 years of experience in upper limb prosthetic care • age ≥ 18 years • language: German • written informed consent
Exclusion criteria • cognitive deficits, limited communication skills (i.e., significant hearing or visual impairments)	

### Data collection and analysis

First, a guideline for conducting interviews based on the recommendations by Helfferich [13] was created, and seven to nine key questions were drafted for each FG. This approach resulted in three group-specific guidelines (PUs, OTs, and THs; Tables 2 & 3). These were not intended to shape the interview as a strict list of questions, but rather to help guide the conversation along topics of interest. They were discussed amongst various research groups with clinical, technical, and bioethical expertise and piloted by two amputation surgery physicians, but no revisions were raised.

**Table 2:** Summarized interview guide for the PU focus groups (FGP1 –FGP5).

**Introductory question (‘icebreaker’) (5 min):** External perception of upper limb prostheses
**Main questions (70 min):** decision for or against a prosthesis, positive and negative prosthetic properties, use of the prosthesis, request for modifications
**Closing questions (15 min):** opinion regarding innovative technologies, additions, emphases, final statements

**Table 3:** Summarized interview guide for the OT focus groups and the TH focus group (FGC1 –FGC3).

**Introductory question (‘icebreaker’) (10 min):**	focus on upper limb prostheses	focus on upper limb prostheses
**Main questions (60 min):**	requirements for adequate care, frequent adjustment issues, use of prostheses, positive and negative feedback regarding the use, requests for modifications	positive and negative feedback regarding use, frequently occurring problems during rehabilitation, important prosthetic properties
**Closing questions (20 min):**	unnecessary features, additions, emphases, final statements	request for improvements, additions, emphases, final statements

Coherency was ensured by a single moderator (MD candidate, female), who performed all FGs and was responsible for examining the collected data. A passive assistant operated the audio recordings using two microphones and documented participants’ non-verbal communication (body language and mimic expressions). The moderator ensured open-ended questions and the spontaneous addition of new topics as they arose. All FGs were held in German. After each FG, PUs were asked to fill out a questionnaire concerning their sociodemographic and prosthetic data. The questionnaire also collected data regarding prosthesis using times and satisfaction with the prosthesis on a ten point rating scale. Based on the transcription rules according to Bohnsack [14], all FGs were transcribed verbatim directly afterwards using a guide and the software f5transkript (Vers. 7, Dr. Dresing und Pehl GmbH, Marburg, Germany). Following Mayring and Fenzl [15], the transcripts were evaluated using summarizing content analysis, a method to summarize content through a process of systematic reductions. Individual sections of the transcription were first paraphrased and then generalized, whereas text components without relevant content were deleted and content-bearing components were brought into a uniform grammatical form. This allowed different categories to be defined and the frequency of their occurrence determined their relevance. The categories where not defined beforehand, which means an inductive category system was used. The category system was worked out using the software MAXQDA Analytics Pro (Vers. 2018, Verbi GmbH, Berlin, Germany).

## Results

Nineteen PUs (Table 4), nine OTs, and four THs (Table 5) were enrolled in this study. Of the PUs, 83% were male and 61% working. The ratio of acquired to congenital amputations is 11:8. The mean age of the PUs was 51.6 years (range: 16–81 years). User 19 complained of feeling unwell halfway through the focus group, so FGP5 was completed with only two participants.

**Table 4:** Distribution of subjects in the FGP (m = male, f = female, FGP = focus group of patients, SMH = standard myoelectric hand, MMH = multiarticular myoelectric hand, PP = passive prosthesis, BP = body-powered prosthesis, WP = without prosthesis.

FGP1	FGP2	FGP3	FGP4	FGP5
SMH & MMH	SMH & MMH	MMH	PP	BP & WP
User 1 (m), myoelectric prosthesis and Michelangelo Hand	User 4 (m), SensorHand Speed, Hybridarm	User 10 (m), i-Limb Quantum	User 14 (m), passive prosthesis	User 17 (m), without prosthetic fitting
User 2 (f), Dynamic Arm, myoelectric hand with wrist joint control	User 5 (m), myoelectric prosthesis, SpeedHand	User 11 (m), myoelectric prosthesis, experience with BeBionic Hand	User 15 (f), passive prosthesis	User 18 (m), body-powered prosthesis
User 3 (m), myoelectric hand	User 6 (m), myoelectric prosthesis	User 12 (f), i-Limb Quantum	User 16 (m), passive prosthesis	User 19 (f) without prosthetic fitting
	User 7 (m), myoelectric DMC Hand, VariusPlus Hand	User 13 (m), BeBionic Hand		
	User 8 (m), Michelangelo Hand			
	User 9 (m), Michelangelo Hand			

**Table 5:** Distribution of members of the focus groups of the caregivers (m = male, f = female).

FGC1	FGC2	FGC3
OTs upper limb	OTs all limbs	THs
OT 1 (m)	OT 4 (m)	TH 1 (f), PHT
OT 2 (m)	OT 5 (m)	TH 2 (m), PHT
OT 3 (m)	OT 6 (m)	TH 3 (f), OCT
	OT 7 (m)	TH 4 (m), PHT
	OT 8 (m)	
	OT 9 (m)	

Of the working PUs, 71% stated they also use the prosthesis in their leisure time, whereas 29% do not. 83% reported using their prosthesis during work. On a scale from 1 to 10 (10 = maximum satisfaction) PUs reported a mean satisfaction of 5.7 (±2.4) for the weight, 5.0 for the noise (±2.4), and 5.0 for the appearance (±2.7) of their prostheses. This data was not collected for the purpose of generalization but to provide an overview of participants’ attitudes towards their prostheses.

After reducing the material and working out its content, three topics were identified that reflect the foci of the participants:

1. Requirements for the prosthesis
2. Requirements on external conditions
3. Individual factors

These topics are presented with their subsumed main categories. These main categories were identified from the dataset based on how frequently they were mentioned. In the results, the categories are underlined and accompanied by a citation from just one subject as an example.

### Prosthesis requirements

The main categories of this topic are prosthesis design, prosthesis construction, prosthesis appearance, a feedback system, and prosthesis functionality.

### Prosthesis design

In all FGs except FGC3, prosthesis weight is considered too high and a lighter weight is preferred.

*„And also a question of the socket. If the prosthesis socket is worked accordingly, the weight is not felt that much (OT 1).”*

Some subjects (FGP1, FGP2, FGP3, FGC1, FGC2) consider battery life insufficient; others disagree and consider it sufficient. They explain that this apparent contradiction was because battery capacity relates to the power consumption of the prosthesis, prosthesis type and frequency of use, and that these requirements can be very different. Some participants (FGP1, FGP3, FGC1) want to be able to replace the battery themselves.

*„The battery capacity, at least in this hand, uh, I think it has a way bigger power consumption than an i-limb hand and therefore is stronger. That’s an advantage and also a disadvantage as the power consumption is way bigger and after half a day the battery is flat (User 13).”*

Another important aspect of prosthesis design is the prosthesis socket, especially the socket fitting. The socket should not cause any pressure points and fit tightly despite moisture (FGP2, FGP5, FGC3). It should also be stable (FGP4, FGP2) and easy to independently put on and remove (FGP1, FGP4, FGP5, FGC3).

*„The socket is the most important thing, yes (OT 3).”*

Prosthesis robustness is discussed in all eight groups. FGC3 and FGP2 emphasize that robustness is more important than a large range of functions.

*„Uh the most important thing is a reliable and robust prosthesis (User 4).”*

In four groups (FGP1, FGP3, FGC1, FGC2) the wish for a waterproof prosthesis is expressed. Also, the electrodes of electrically controlled prostheses are discussed. FGP1 and FGP2 participants report malfunctions depending on the temperature and humidity of the upper limb stump.

*„When I’m working hard and start to sweat, the electrodes start to get wet and I get erratic actions or I have to turn them off (User 1).”*

The last prosthesis design aspect relates to the material of the prosthesis glove. Six focus groups (FGP1, FGP2, FGP4, FGP5, FGC1, FGC3) mention that resistance to dirt and contamination of the glove is relevant. Difficulties in cleaning the gloves, the associated additional costs of cleaning and hygiene, and the resulting need for frequent replacements are.

*„Actually, I can only add. On the one hand, there is this dirt, which is very unpleasant, that the glove gets dirty, you then have to replace the whole thing, which is very expensive and uh the same applies to hygiene (User1).”*

### Prosthesis construction

An important aspect here is the distribution of the prosthesis weight. The desire is for the weight of the prosthesis to be shifted proximally, i.e. close to the stump. The therapists remark that muscle-strengthening muscle is important when such a shift is made.

*„Yes, I always ask myself why not build a hand and put the motor closer to the stump via a shaft. Yes, where one simply says: reduction of distal weight (OT 1).”*

### Prosthesis appearance

The relevance of prosthesis appearance is mentioned in seven of the eight focus groups (FGP1, FGP2, FGP3, FGP4, FGP5, FGC1, FGC2). Almost all participants desire a prosthesis with a physiological appearance that goes unnoticed, also under clothing. However, a few PUs prefer a conspicuous prosthesis that is unique and can still look technical.

*„What I think is important: It may look technical, but it should have a reasonably chic design (User 13).”*

### Feedback system

Various aspects of a possible feedback system are discussed. One aspect is the relevance of such a system. Five groups (FGP1, FGP4, FGP5, FGC1, FGC2) state that sensory feedback would be useful. However, some participants also think that a feedback system is unnecessary since optical and auditory control could compensate for much sensory loss.

*„Well, apart from hot and cold, I always get feedback somewhere. Because I hear my engine. And I grab (Interviewer: Yes.) And how, I also hear how tightly I grip. You’ll hear that at some point. You have that in your ears (User 7).”*

Another aspect is the impact of a feedback system on the personal environment. For example, it is noted that such a system reduces the risk of injury to other people as the grip strength when handling small children can be better assessed.

*„Yes, it has already happened. That’s why. So, I grabbed him (the son) by the arm. And I didn’t want to hold it so tight. [omitted]. if they are still a small child, then better without a prosthesis (User 5).”*

Implementing such a system is the last aspect. The possibility of an acoustic signal as feedback on the grip strength (FGP1, FGP2, FGP3, FGC1, FGC2) or temperature (FGP2, FGP5, FGC2, FGC3) is suggested. Furthermore, FGP3 repeatedly state that feedback on proprioception could be useful. A feedback signal in the form of vibrations is also discussed (FGP3, FGC1).

### Functionality of the prosthesis

Grip strength is a highly discussed aspect of functionality. In four groups (FGP3, FGP4, FGP5, FGC1) greater grip strength is mentioned as important. FGP3 and FGC3 state that grip strength is insufficient. Grip velocity is brought up here as some users (FGP2, FGP3) consider it too slow. Even velocity regulation through Dynamic Mode Control (DMC) is insufficient. The wish to be able to quickly switch between different grips is also mentioned (FGP5, FGC2, FGC3).

*„Too little power, it’s just-. Well, a Touch Bionic hand just doesn’t have enough strength to hold something, no matter what, in such a way that they … are especially when it’s fine grips, the strength uh the fine grips that require strength. A Touch Bionics hand can’t do that because the thumb turns to the side, so it was like that with mine (User 13).”*

In all groups except FGC1, participants state the relevance of a large range of functions. Desired functions are distinguished into gripping, holding, and fine-motor functions, which are all considered important. Gross motor function is mentioned less often. In the FGP2 group, the wish for simultaneously performed grips is expressed. Also, users of passive prostheses desire different grip possibilities. Requests for automatized grip patterns (FGP2), the possibility of touchpad handling (FGP1, FGP5) as well as the controlling of different grip patterns via app (FGP5, FGC3) come up. Apart from the grip capabilities, participants mention malfunctions of the prosthesis and activities that cannot be performed with the prosthesis. Examples stated here are electromagnetic interference (FGP1, FGP2) or uncontrolled movements of the prosthesis (FGP1, FGP2, FGP3, FGC1). According to the technicians, a poorly adjusted socket can be the cause of such incorrect controls.

*„And, as the gentlemen here have already said, the prosthesis does what it’s not supposed to. (Everyone laughs) It’s like sometimes a finger goes up and down, even though he really can’t do anything about it. Sounds weird, but still, yes, I think they have a life of their own (User 10).”*

Various application areas are identified as desirable for the prostheses. Operating vehicles (FGP1, FGP2, FGP3, FGP4, FGP5, FGC1, FGC2) and housekeeping (FGP1, FGP3, FGC1, FGC2) are frequently mentioned, and the use of cutlery or tools is also viewed as desirable.

Another aspect of functionality are the technical requirements for the prosthesis. In FGP1, FGP3, and FGC1, reliability of the prosthesis is considered more important than a modern appearance or large range of function. Further aspects are the wishes for intuitive movements (FGP1, FGP4, FGP5, FGC3), mechanical stability (FGP3), and the demand for a simple control of the prosthesis (FGP2, FGP5, FGC3). Automatic regripping of the prosthesis at physical contact is viewed negatively (FGP2, FGC3). Some participants also articulate the desire to individually adapt the prosthesis’ range of functions. In four groups (FGP1, FGP3, FGP4, FGP5), the participants desire more flexible fingers, whereas in another four groups (FGP1, FGP4, FGP5, FGC2) pro- and supination capabilities were requested.

### Requirements on external conditions

The main categories here include prosthesis fitting and service.

### Prosthesis fitting

In almost all groups, costs associated with owning a prosthesis are criticized.

*„And what I would wish for is that the uh (short pause), how should I put it, that cheaper solutions would be found (User 13).”*

Another fitting aspect is related to therapists, technicians, and physicians. While users underline the importance of communication with care staff (FGP3), technicians and therapists underline the relevance of interdisciplinary collaboration between professional groups. Technicians also highlight the importance of therapeutic care of the patients but are also critical about the lack of experienced therapists active in prosthetics (FGC2).

*„But here we have the actually largest deficit in this collaboration. (Interviewer: Where?) So good prosthetists that can deal with the technology there are enough of. Physiotherapists that can simply and comprehensively get to work without one of us supervising, uh yeah… I would say there is hardly anyone (OT 1).”*

A last aspect here is the equipment of the persons concerned. Five groups (FGP3, FGP4, FGC1, FGC2, FGC3) frequently mention that the type of prosthesis needs to be individually customized to users. Furthermore, users request substitutional supply (FGP3, FGC1, FGC3), especially when the prosthesis is defect. It is suggested that prostheses no longer used should be returned to care providers so that these could be offered as a replacement or testing supply for other users.

### Service

Excessive waiting times during prosthesis repair are mentioned.

*„And the service times should be well under six weeks (User 7).”*

The lack of available information about technical innovations is criticized. There is also criticism of the unrealistic advertising available on the internet, which arises false expectations among users. On top of that, trial prosthesis supply should be available for more than two weeks to ensure that a suitable prosthesis is identified and to maximize prosthesis use. Furthermore, local service centers should be able to perform standard maintenance (FGP1, FGP3, FGC2, FGC3).

### Individual factors

The main individual factors categories are psychosocial factors, somatic factors, and the prosthesis use.

### Psychosocial factors

In this category, a frequent subject of discussion is the external effect of the prosthesis. Impressions differ between greater acceptance for the arm stump or for the prosthesis. Opinions vary particularly in the case of children. Regarding sports, it is also stated that peers are afraid of being injured by the prosthesis. Five focus groups (FGP1, FGP2, FGP3, FGP5, FGC3) state that the prosthesis completes the body image. Participants also state that they seem disabled without the prosthesis (FGP3, FGP4, FGP2).

*„The positive thing is that others don’t immediately notice that you are disabled (User 14).”*

In addition, participants report that everyday life is not possible without the prosthesis as it enables independence and self-determination.

*„Because without a prosthesis (short pause) our quality of life is zero (User 11).”*

### Somatic factors

In this category, the focus is on the influence of the stump. Five groups (FGP1, FGP2, FGP3, FGC1, FGC3) mention, that amputation level influences prosthesis functionality. The disadvantages associated with a short stump are emphasized, i.e. a short stump aggravates distal gripping (FGP4, FGC2, FGC3). Participants of FGP1 also state that electrode contact depends on stump shape.

### Use of prostheses

Prosthesis use is another frequently addressed issue. Participants in the PP group repeatedly criticize the prosthesis as uncomfortable, superfluous, and disruptive and that it must offer benefits in order to be used. Within this group, it is repeatedly mentioned that there is no interest in technical innovations on the prosthesis market.

*„It’s just plain and simply superfluous. As long as you have one hand that you can really do something with, you don’t do anything with it (note: with the prosthesis). Then it’s just plain and simply a burden, the thing just gets in the way (User 14).”*

Four groups (FGP4, FGP5, FGC2, FGC3) state that dysmelia patients are used to coping without a prosthesis and that they see it more as a tool than as a replacement hand. Many participants underline that effort and benefit must be balanced when using the prosthesis (FGP4, FGP5, FGC2) and that awareness of the advantages of the prosthesis influences the acceptance (FGC1, FGC3).

*„Yes, and that (note: the myoelectric prosthesis) totally fascinated me, which is why I had it demonstrated to me because I was totally fascinated. But, as I said, even then the point hadn’t been reached, uh that uh well, from my perspective, you really have added value. The disadvantages: costs, weight, lack of robustness, sensitivity, etc. were just higher than the benefit (User 16).”*

Some participants express the importance of being independent from the prosthesis (FGP1, FGP4, FGP5). For scope of use, it is often stated that the prosthesis is only removed while in the water or sleeping (FGP1, FGP3, FGP5, FGP2) and that use of prostheses in leisure time is also highly relevant (FGP4, FGC2). However, other participants state (FGP4, FGP5) that they only use their prosthesis at work.

## Discussion

The sociodemographic data of prosthesis users is in accordance with the overall data of upper extremity amputees, as most of them are male and trauma is the most common reason for amputation [5, 16]. The results are at first discussed in relation to the research questions followed by an overview of mentioned aspects that have been addressed in the SoftPro project.

### 1. What are the most important requirements for users and professionals?

The main requirements mentioned are prosthesis weight, battery capacity, fitting and manufacture of the socket, reliability, robustness, glove’s susceptibility to dirt, prosthesis appearance, grip strength and velocity, and possibilities for fine and gross motor functions as well as physiological movements. These aspects were frequently mentioned, indicating their high relevance for the participants. Some external factors were also frequently mentioned, such as demands for an affordable prosthesis, interdisciplinary care, the possibility of trial fittings, and a fast service close to home.

### 2. Have technical innovations in recent years changed the requirements of users and professionals?

Many points of criticism still coincide with criticisms identified in comparable studies in the past. Notable criticisms are prosthesis weight, susceptibility of the prosthesis glove to dirt, fitting of the socket, and prosthesis robustness and appearance [5, 17–25]. Also, criticism of insufficient grip strength and grip speed persist [5, 8, 9, 19, 22, 26]. That most problems mentioned by patients with prosthetic hands and arms appear unchanged after two decades demonstrate the prosthetic industry’s inability to adequately address these issues. Therefore, manufacturers need to incorporate development and innovation processes that take users’ needs and professionals’ recommendations into account. As sometimes happens in other technical fields, new developments in hand prosthetics appear to have a partially detrimental effect on initially advantageous developments. One example is the improvement of battery capacity, which is canceled by the increased power consumption of multi-articulating prostheses to meet the requirement for movable fingers [4, 17].

### 3. Which unfulfilled requirements of past prosthetic devices can be addressed with future prosthetic devices?

The participants formulated specific requirements for prostheses that can be directly addressed through new technical developments. These include requirements for prosthesis design (weight, battery, socket, electrodes, robustness, prosthetic glove), structure, and appearance, a possible feedback system, and prosthesis functionality (grip strength, grip speed, grip options, intuitive grip patterns). Although these requirements can be addressed directly with new developments in prosthetics, an improved prosthesis does not guarantee successful use. For example, some users of passive prostheses find the prosthesis annoying and superfluous and are therefore not interested in new types of prostheses. In such cases, education should be carried out first, since—as participants in FGC1 and FGC3 mentioned—awareness of the advantages of the prosthesis influences acceptance.

The emphasis on relevance of the interaction between a well-functioning prosthesis and a well-developed infrastructure for successful prosthesis use has only been mentioned occasionally in other studies. This could be because previous research either focused on the prosthesis [7, 8] or on the psychosocial aspects [2] but not on the infrastructure, which also plays an important role for users.

In general, additional solutions for care and service should be developed, which besides education, include financing, alternating and test supplies, and reduced maintenance times. In addition to prosthesis and infrastructure requirements, this study shows how strong the influence of users’ individual psychological and physical prerequisites is on prosthesis use, which is why new developments should consider these. Further research is also necessary and should address whether an improved production of currently available prostheses (e.g., better adaptation of the socket, reorganization of individual components, etc.) would be enough to improve their functionality. A quantitative investigation based on this study to assess the statistical significance of the identified requirements would also be useful. Finally, it should be investigated whether close interdisciplinary support could improve the influence of psychosocial factors on prosthesis use.

In the aforementioned SoftPro project, an industrial design method with multiple strands was employed as the foundation for transferring a robotic end effector gripper into a prosthetic hand, based on the structure and kinematics of the human hand. This design method was based on a user centred approach and for this reason encompassed various aspects, including the consideration of practical issues and the desires of amputees, users, and experts in the field of upper limb prosthetics from the present focus group study.

The authors were somewhat taken aback by the long-standing inadequacies, which they encountered during the development process and were already known for years. Therefore, the development of the prosthetic hand focused on the aspects most frequently mentioned here. As a result, the primary focus during the development of the SoftHand Pro (Version 1) was on achieving a combination of fundamental and advanced grasping functionality, precise and reliable EMG control, adaptable grasping capabilities (ranging from firm to gentle and also speed-adjusted grasping), the ability to manipulate objects of varying sizes (without requiring switching processes), complete mobility of fingers and thumb (eliminating rigid finger segments through the use of soft robotics), weight reduction of the prosthetic hand (m = 520g through lightweight construction) and the prosthetic arm (optimized battery positioning), and compatibility with other myoelectric systems. Additionally, considerable attention was given to ensuring comparatively low manufacturing costs.

As part of the further development within the Technology Readiness Level framework, the SoftHand Pro (Version 3) was designed. This version aimed to improve upon the second hand version, focusing on enhancing the cosmetic appearance through anatomically optimized hand and finger proportions, improving the aesthetic appeal through an optimized cosmetic glove that facilitates easier and thorough cleaning of the hand and enables touch screen operability, improving the grasping forces, introducing passive wrist mobility, employing more robust and less error-prone mechanical and electrical components, enabling the ability to eat with cutlery, further reducing the overall weight (m = 290g), and achieving nearly complete water resistance.

Ultimately, a fundamental aspect was also ensuring a lifespan of system usage compatible with the patients’ needs. In this regard, the internal implementation scheme and the design of the finger’s elastic components were completely revolutionized. This has extended the system’s functional life from a few thousand cycles to hundreds of thousands of opening and closing cycles.

## Limitations

Although the number of participants in this study is comparable to other qualitative studies in this area, the results cannot be generalized. Rather this study should be viewed as an extensive collection of relevant aspects for developing prostheses. The group sizes varied between three and six participants. Although FGP5 started with three participants, only two were left at the end. In the focus groups of the technicians and therapists, some of participants already knew each other beforehand, which might have influenced their statements since a certain distribution of roles already existed. The moderator therefore ensured that all participants had the opportunity to speak. Finally, the number of group participants per focus group varied, which might have influenced the frequency of mentions.

## Conclusion

In this study, focus groups on the improvement of prosthetic design and care were conducted with Pua, Ots, PTHs and OTHs and qualitatively evaluated using summarizing content analysis. Focusing on the development of future prostheses, three main topics relevant for improving function and reducing rejection were identified: a properly functioning prosthesis, the infrastructure and users’ psychological and physical prerequisites. The most commonly mentioned technical deficiencies with regard to currently available hand prostheses from the users’ perspective were the robustness of the prostheses, lacking possibilities for fine and gross motor functions as well as physiological movements, and the glove’s susceptibility to dirt. This information is already used in the development of the SoftHand Pro and could play a key role for manufacturing stakeholders in significantly improving the quality level for amputees through rapid and effective technical enhancements. A successful technical addressing of these deficiencies should be in the best interest of such companies, as it presents the opportunity to generate long-term customer loyalty.

The interaction between a well-functioning prosthesis and a well-developed infrastructure was identified as an important influence on successful use. Additional solutions for supply and service are therefore needed. Most problems identified in the literature in recent decades are still not addressed today. Therefore, the prosthetic industry should try to pay more attention to users’ needs and professionals’ recommendations. Moreover, individual psychological and physical prerequisites for prosthesis use should be considered in new developments.

## Supporting information

S1 Appendix Anonymized transcripts in German language. (ZIP)
