# Supplementary material for: What do users and their aiding professionals want from future devices in upper limb prosthetics? A focus group study
Source: PLoS One. 2023 Dec 29;18(12):e0295516. doi: 10.1371/journal.pone.0295516 (PMC10756510; doi:10.1371/journal.pone.0295516)
Supplement: S1 Appendix — (ZIP) [file pone.0295516.s001.zip › FocusGroup_Transcripts/FGC2.pdf]

Interviewerin: Ähm ich würd‘ Sie bitten, wenn ich die erste Frage gleich einmal in die Runde gebe, dass da erstmal der Reihe nach geantwortet wird, dass jeder sich ganz kurz einmal vorstellt, dann hab ich nämlich auf der Aufnahme erstmal die Namen mit den Stimmen verknüpft und dann in den Weiteren ist es mir egal, nur in der Ersten, dass wir da das einmal der Reihe nach machen. (kurze Pause) Ähm ich würd‘ Sie bitten, zum Anfang, sich vorzustellen, dass Sie, als Fachmann jemanden treffen mit ‘ner Prothese und, dass Sie mal erzählen, worauf Sie da als erstes achten, was da als erstes eben wichtig ist, wenn jemand zu Ihnen kommt, der ‘ne Prothese trägt? #00:00:37-7#

Teilnehmer 30: Ähm also mein Name ist (Name von Teilnehmer 30 aus Datenschutzgründen ausgelassen), also ich würde mir erstmal den Schaft angucken, wahrscheinlich. Oder&also je nachdem was er für ein Problem hat, wenn er, wenn er jetzt äh wenn ich j-&wenn sich jetzt einfach, wenn ich mir einfach nur die Prothese angucken soll, mit: „Schau dir das Ding mal an!“, dann würd ich erstmal gucken: Wie sitzt der Schaft? (Interviewerin: Mhm (bejahend)) Das ist für mich so das Erste. #00:00:57-7#

Interviewerin: Ja. #00:00:58-8#

Teilnehmer 31: Mein Name ist (Name von Teilnehmer 31 aus Datenschutzgründen ausgelassen), wenn spontan jemand vor mir steht, würde ich ihn als erstes Mal fra-&befragen, warum er zu mir kommt. (Interviewerin: Mhm (bejahend)) Was sein Anliegen ist, würd‘ ihm erstmal zuhören. Und dann die Sache angehen, (Interviewerin: Mhm (bejahend)) ob überhaupt ein Problem die Prothese ist, oder ob’s ‘n ganz anderes, in dem Zusammenhang ist. #00:01:19-6#

Interviewerin: Mhm (bejahend). Und gibt’s aber irgendwas, was da als erstes auffällt? Oder gibt’s zum Beispiel irgendein Problem mit dem Menschen kommen, was man gleich am Anfang merkt? #00:01:28-4#

Teilnehmer 31: Ich bin ja noch ganz offen am Anfang. (Interviewerin: Mhm (bejahend)) Bis jetzt ist nur das Wort Prothese gefallen, (Interviewerin: Mhm (bejahend)) das heißt ich würd‘ dann erstmal als nächsten Schritt rangehen und gucken: Um welche Prothese geht es? (Interviewerin: Ja.) Trägt er ‘ne Armprothese? Ich weiß nicht, ob wir das schon so eingegrenzt haben hier in diesem Raum, (Interviewerin: Ja, also Armprothesen sind gemeint.) dass es ausschließlich um Armprothesen geht (Interviewerin: Mhm (bejahend))? Wenn das also die Voraussetzung ist, dass es um Armprothesen geht (Interviewerin: Ja, mhm (bejahend)) und es eine Problematik gibt mh würd ich sicherlich erstmal das gesamte Bild gucken: Ist es im Körper harmonisch eingegliedert, gibt es schon Auffälligkeiten, optische Auffälligkeiten, zu lang, zu kurz, was weiß ich. (Interviewerin: Mhm (bejahend)) Äh schlicht und ergreifend nochmal bisschen Vorgeschichte befragen, wie’s dazu kam, wo die Prothese gefertigt ist und dann schlicht und ergreifend in Verbindung mit dem Problem, was mir dann ans Herz getragen wird, dann eben tatsächlich das Hilfsmittel selber angucken. #00:02:23-0#

Interviewerin: Mhm (bejahend), ja ok. Danke. #00:02:25-6#

Teilnehmer 32: Mein Name ist (Namen von Teilnehmer 32 aus Datenschutzgründen ausgelassen) ähm Erstkontakt würd‘ ich auch erstmal fragen was derjenige für ein

Anliegen hat, würde auch die Prothese mitanschauen, ähm Stumpfverhältnisse und erstmal klären, was seine Wünsche sind, wie lange er die Prothese trägt, was er schon getragen hat und ähm wohin es gehen soll. (Interviewerin: Mhm (bejahend)) Und dann halt ähm auch schauen, wenn wir ‘ne Prothesenart halt dann auch geeignet haben, ähm auch ähm welche Anforderungen auch er an der Prothese hat und dann entscheiden, genau. #00:03:06-9#

Teilnehmer 33: Ja, mein Name ist (Name von Teilnehmer 33 aus Datenschutzgründen ausgelassen), ich würd mich auch erstmal mit dem Patienten beschäftigen, gucken: Wie sind die Stumpfverhältnisse, was ist es für ‘n Verhältnis vom Stumpf überhaupt und äh was die Ursache, dass er ‘ne Prothese trägt, wenn ich ihn nicht kenne, ob das Trauma-Amputation, Dysmelie (...?) oder sonst irgendwas. Ja und dann nach den Anliegen fragen, was gewünscht ist. Ist ja sicherlich ‘n Unterschied, ob’s schon ‘ne Vorversorgung gibt oder ob es ‘ne Erstversorgung gibt und dann entsprechend fortfahren. (Interviewerin: Mhm (bejahend)) #00:03:39-3#

Teilnehmer 34: Ja, mein Name ist (Name von Teilnehmer 34 aus Datenschutzgründen ausgelassen), natürlich würd‘ ich mir auch erstmal den Patienten angucken, ähm fragen, was er für Wünsche hat, was äh&ähm, wo die Amputation überhaupt äh ist oder ob es äh ‘ne angeborene Geschichte ist. Ähm ja. Und dann würd‘ ich auch nach den Wünschen fragen ähm, ob’s nur ‘n kosmetischer Einwand sein soll, ob’s ‘ne Myoelektrik sein soll, ob’s, oder ob’s alleine nur ‘n&‘n&‘n ‘ne Arbeitshand werden soll. (Interviewerin: Mhm (bejahend)) Genau, erstmal nach den Wünschen fragen, ich denk das ist das Wichtigste. Und ob er dann ‘n passenden Schaft hat oder nicht, dass äh spielt für mich erstmal keine Rolle. (Interviewerin: Mhm (bejahend)) #00:04:21-5#

Teilnehmer 35: Mein Name ist (Name von Teilnehmer 35 aus Datenschutzgründen ausgelassen), kann mich da den Vorrednern nur anschließen, erstmal eruieren was der Grund überhaupt für den Besuch ist, wenn er ‘ne Versorgung hat, wo die Defizite liegen seiner Meinung nach und dann so ’n bisschen das must-to-have und nice-to-have. Eigentlich eruieren: Was will derjenige, was muss er machen (Interviewerin: Mhm (bejahend)) und was würde er gerne machen und bei dem was er machen kann, wo liegen da die Problematiken. (Interviewerin: Mhm (bejahend)) Und dann kann man weitersehen, wo dann der Mangel eigentlich liegt, ob es wirklich der Schaft ist, oder ob es die Auswahl der Komponenten sind, ist dann bei mir erstmal zweitrangig. (Interviewerin: Ja.) #00:04:58-0#

Interviewerin: Ähm Herr (Name von Teilnehmer 31 aus Datenschutzgründen ausgelassen), Sie haben gesagt, dass Sie eben gucken auch wie passt das ins Körperbild und so ’n bisschen die Länge, aber auch in die Erscheinung (Teilnehmer 31: Ja.). Was&was fällt denn da auf? Wenn was zum Beispiel nicht richtig sitzt oder wenn’s ‘n Problem gibt, was fällt in so ’nem Erscheinungsbild auf, wenn da irgendwas nicht stimmt? #00:05:15-4#

Teilnehmer 31: Ja gut, sind schon erste Merkmale, was weiß ich, mh Armlängendifferenzen, was weiß ich, unterschiedliche Schulterhöhen oder ob irgend ‘nen schon (im Verbund?) mit ’m Körper gestanden wird. Ich meine das ist ähm deswegen hatt‘ ich ja vorher gesagt äh ich bin vollkommen unvoreingenommen und nachgefragt (Interviewerin: Mhm (bejahend), ja.) ob das schon auf Armprothetik

eingegrenzt wird. Das ist eigentlich ‘ne Vorgehensweise grundsätzlich, wenn jemand vor mir steht und ich kenn das Anliegen noch nicht und sei es ob’s um Beinprothesen geht oder eben um Armprothesen, was auch immer, um ‘ne Orthese (Interviewerin: Mhm (bejahend)) ist eigentlich immer erstmal so ’n erstes Durchchecken schon mal so ganz k-&ganz komplett. Was passiert da, wer steht da vor mir, wo könnten Probleme liegen, wo äh, oftmals erkennt man ja auch, sagen wir mal schon beim Reinkommen. Das ist so ‘ne gewissen Ahnung warum dieser Mensch jetzt auf einen zukommt. (Interviewerin: Mhm (bejahend)) Das ist so dieser Hintergrund. Das heißt also so das gesamte Körperschema, was weiß ich, wird eine Seite vernachlässigt, inwieweit ist das Hilfsmittel integriert indem ’s tatsächlich Funktionen ersetzt oder nur hinterhergezogen wird, wie auch immer. (Interviewerin: Ja.) #00:06:21-6#

Interviewerin: Hat da noch jemand irgendwas zu ergänzen? #00:06:24-1#

(kurze Pause) #00:06:25-4#

Interviewerin: Ok. Ähm dann würd‘ ich gerne als nächstes wissen, wenn jemand zu Ihnen kommt und ganz explizit wünscht eine moderne Prothese zu bekommen, was für Eigenschaften oder Voraussetzungen muss der Patient mit sich bringen, dass ihm so ‘ne Prothese angepasst werden kann? #00:06:44-3#

Teilnehmer 30: Ja also der muss irgendwie kognitiv in der Lage zu sein zu verstehen, wie so ‘ne Prothese funktioniert (Interviewerin: Mhm (bejahend)) und das dann auch äh umzusetzen und er muss Willens sein das zu lernen. (Interviewerin: Ja.) Also das. Ja. #00:07:00-6#

Teilnehmer 35: Also physiologische Voraussetzungen erstmal, sind die gegeben. (Interviewerin: Mhm (bejahend)) Wenn’s jetzt um myoelektrische Armprothesen geht. (Teilnehmer 30: Das natürlich.) Krieg ich überhaupt was greifbar zu machen, sind die Signale so, dass ich überhaupt in der&damit in der Lage ist äh dynamisch so ‘ne Hand zu steuern (Interviewerin: Mhm (bejahend)). Und äh ansonsten muss da erstmal eruiert werden: Kann man das schaffen? Nur weil jetzt keine da sind, heißt ja nicht, dass übermorgen immer noch keine da sind. Da haben wir ja wirklich schon die dollsten Dinge erlebt. Und, aber das sind eigentlich erstmal die Hauptmerkmale ‘ne? (Interviewerin: Mhm (bejahend)) Die man abfragen muss. #00:07:33-4#

Interviewerin: Mhm (bejahend). Und wann zum Beispiel ähm stimmen die physiologischen Bedingungen nicht, wann kommt das vor, dass man da die Signale nicht bekommt? #00:07:40-8#

Teilnehmer 35: Also wenn’s traumatische Verletzungen sind, äh zum Beispiel jetzt Autounfälle, Motorradunfälle, Muskelabrisse, eventuell verbrannte Haut oder zerstörte Hautoberfläche da ist, die stark vernarbt sind, (Interviewerin: Mhm (bejahend)) dann kriegt man mit den heutigen Methoden also über Abgriff äh Hautoberfläche Elektroden, die Muskelsignale eben teilweise sehr schlecht, gar nicht, oder wenn welche da sind, sind sie nicht explizit gut zu steuern in einem ganz bestimmten Bereich, (um?) ‘ne gewissen Grunddynamik und bestimmte Funktionen auch damit ausführen zu können. #00:08:10-2#

Interviewerin: Mhm (bejahend). Und Sie haben jetzt grade gesagt, dass das sie dann

nicht da sind, muss ja nicht heißen, dass es so bleibt, so Signale. Was kann man dann zum Beispiel machen, was hat man dann für Möglichkeiten? #00:08:20-8#

Teilnehmer 35: Versuchen aufzutrainieren. Also bei Dysmelien zum Beispiel, die nie ‘ne Hand gehabt haben (Interviewerin: Mhm (bejahend)), äh die können ja grundsätzlich hat das Gehirn für diese Seite noch nie abgespeichert, dass es Beuger und Strecker unterschiedlich bewegt. (Interviewerin: Mhm (bejahend)) Die machen meist ‘ne Doppelkontraktion, (Interviewerin: Mhm (bejahend)) das heißt sie haben dann immer dementsprechend chaotische Abläufe von Signalen, aber keine explizit differenziert unterschiedlichen Merkmale. Das kann man aber alles auftrainieren. Also ich hab‘ bisher noch keinen gehabt, wo das nicht ging. (Interviewerin: Mhm (bejahend)) Bei dem einen geht’s schnell, bei dem anderen dauert ’s länger, aber da muss man im Vorfeld halt die Rahmenbedingungen schaffen. #00:08:54-9#

Interviewerin: Mhm (bejahend), ja. Und was gibt’s sonst noch für Voraussetzungen, die man mitbringen muss, damit man so ’ne Prothese tragen kann? #00:09:02-5#

Teilnehmer 33: Die Stumpfverhältnisse müssen gegeben sein. (Interviewerin: Mhm (bejahend)) Ja grade bei Dysmelien hat man oft sehr kurze Stümpfe, ‘n großen Weichteilanteil in der Beugefalte, wenn das Unterarmstümpfe sind, die’s ja meist sind, dann muss man schon gucken ob die Hebeverhältnisse überhaupt so sind, grade bei Kindern, ob durch die Weichteile nicht ‘ne Rotation im Schaft dann zu doll ist. (Interviewerin: Mhm (bejahend)) Oder so, da muss man schon genau gucken. Mit welchem Ziel man arbeitet und vorher genau schauen: Ist das möglich oder nicht, ne? #00:09:31-1#

Interviewerin: Und wär das dann aber zum Beispiel ‘n Kriterium was das ausschließt, wenn die Stumpfverhältnisse nicht stimmen? Oder gibt’s da irgend ‘ne Möglichkeit mit der man das überbrücken kann? #00:09:44-6#

Teilnehmer 33: Man kann die Prothese so kurz wie möglich bauen, um die Hebelverhältnisse eben zu ändern, aber irgendwo gibt’s da dann auch ästhetische Grenzen. (Interviewerin: Mhm (bejahend)) Und wir haben immer noch das Problem, dass viele Hände eben doch eben zu schwer sind. Und, dass wir mit den Kinderhänden die da sind, eben ästhetisch doch nicht das oft abdecken können, was wir gern wollen. (Interviewerin: Mhm (bejahend)) Und wenn man dann in die größeren Größen geht, dann ist das oft ‘n Gewichtsproblem halt, ‘ne? Dass man vielleicht auch sogar die Prothese tragen kann, auch ansteuern kann, aber wenn man wirklich aktiv damit arbeiten möchte (Interviewerin: Mhm (bejahend)), dass es eben nicht möglich ist, weil’s einfach zu schwer ist. #00:10:29-6#

Teilnehmer 30: Aber nicht nur bei Kindern! (Teilnehmer 33: Nein, nein&nein.) Es ist ja bei erwachsenen Dysmelien ja auch schon so (Teilnehmer 33: Ich meine bei&bei&bei Dysmelien generell.), also dass&dass&dass der Hebel einfach zu, viel zu schwer wird für ganz kurze Dysmeliestümpfe. (Interviewerin: Mhm (bejahend)) #00:10:33-6#

Teilnehmer 32: Man kann dann halt auf den (oberen?) Arm zugreifen, ist aber dann halt in der Funktion eingeschränkt (Interviewerin: Mhm (bejahend)). Das heißt Streckung, Beugung dann (...?) halt reinzubekommen. (Interviewerin: Mhm (bejahend)) Ja. #00:10:48-3#

Interviewerin: Ok. Das heißt dann, obwohl ich's dann anpassen könnte, ist die Funktion einfach trotzdem beeinträchtigt? #00:10:52-2#

Teilnehmer 32: Richtig. #00:10:54-6#

Interviewerin: Und es nicken alle, das heißt das Problem ähm kennt jeder? #00:10:58-4#

Teilnehmer 31: Ist bekannt, ja. #00:10:58-9#

Interviewerin: Mhm (bejahend) #00:10:59-9#

Teilnehmer 35: Joah, in der einen oder andern Form ja. #00:11:01-1#

Interviewerin: Ok. Gibt's noch irgendwas, was Ihnen einfällt, wo Sie zum Beispiel sagen würden, weiß nicht, so wie Sie den Patienten erleben oder das was der mitbringt ist so, dass Sie sagen: „Dir würd' ich das nicht raten, dass du sowas nimmst."? #00:11:15-3#

Teilnehmer 31: Na gut, das geht ja schon äh, sagen wir mal, im ersten Gespräch oder in den ersten Gesprächen definitiv darum, schon bisschen so die Lebensumstände des Betroffenen kennenzulernen. (Interviewerin: Mhm (bejahend)) Ähm das heißt also, auch wenn vor mir 'n Mitte 20 -jähriger steht und sagt: „Ich will jetzt was modernes.", ich verbinde jetzt mal mit dem Begriff „modern" eben auch 'ne myoelektrische Prothese, äh er erzählt, dass er Dreher ist und was weiß ich alles mögliche tut und ich sehe, er kommt zu dem Gespräch schon, sagen wir mal, mit 'nem Lederschutz, einfach nur für seinen kurzen äh Stumpf, der acht Zentimeter lang ist und ich so nach Problemen frage, die sich vielleicht so für ihn im Berufsleben auftun und dann sagt er: „Nee, hab ich gar keine Probleme. Wieso? Mach ich doch alles damit." (Interviewerin: Mhm (bejahend)) So. Und genauso ist dann auch diese entsprechende Versorgung ausgegangen. Schlicht und ergreifend liegt 'ne teure myoelektrische Prothese auf 'm Regal und er holt sich immer wieder seinen alten Arbeitsarm raus, weil eben definitiv beim kurzen Stumpf die Hebelverhältnisse äh dann nicht gepasst haben und an der Maschine stehend ständig überlegen: „Umschalten, wo bin ich jetzt, in welchem Modus?" und wie auch immer, hat alles viel zu lange gedauert. Endet 'ne erstmal gut angedachte Versorgung in der Schublade. (Interviewerin: Mhm (bejahend)) #00:12:33-1#

Interviewerin: Ja. Gibt's noch irgendwelche Lebensumstände die so 'ne Prothese ausschließen? #00:12:40-0#

(kurze Pause) #00:12:40-9#

Teilnehmer 33: Äh die äußeren Lebensumstände. Wir sind ja immer wieder konfrontiert mit Mitbürgern oder Mitmenschen, die nicht aus unseren Regionen kommen und es sollte zumindest 'n Service möglich sein (Interviewerin: Ja.) und es sollte bei modernen Prothesen Strom da sein. Wir haben aber durchaus auch schon Patienten gehabt, wo beides nicht gegeben ist (Interviewerin: Mhm (bejahend)) und dann muss man schon genau überlegen: „Wie sind die in der Heimat versorgt, was können die mit der Prothese wirklich machen (Interviewerin: Mhm (bejahend)) und

wie kann denen noch geholfen werden, wenn so ‘ne Prothese ausfällt?“, ‘ne? (Interviewerin: Mhm (bejahend)) Also das passiert auch schon mal, das sollte man schon zumindest wissen, wenn man moderne Prothesen versorgt. #00:13:17-4#

Interviewerin: Mhm (bejahend). Also das einfach die Nachsorge und so ’n Reparaturservice und so was gegeben sein können? #00:13:23-5#

Teilnehmer 33: Ja. #00:13:24-1#

Interviewerin: Mhm (bejahend) #00:13:27-6#

Teilnehmer 35: Ja in den unterschiedlichen Ländern in den ländlichen Bereichen in bestimmten Ländern ist es dann schwer für die wenn irgendwas defekt ist, da an Ersatzteile oder Service zu kommen. (Interviewerin: Ja.) #00:13:36-5#

Interviewerin: Fällt jemandem noch was ein? #00:13:39-2#

(kurze Pause) #00:13:41-8#

Interviewerin: Ok. Dann würd‘ ich Sie bitten, dass Sie sich vorstellen, dass eben der Patient der kam und gesagt hat er möchte ‘ne moderne Prothese, bringt alle Voraussetzungen mit, bekommt die und bekommt die angepasst. Und wir gehen mal davon aus, dass der Schaft perfekt sitzt. Was für andere Probleme treten denn auf bei so ’ner Versorgung? Was gibt’s da noch, was mal Schwierigkeiten bereitet, wo man, wo vielleicht besonders viel Zeit ähm reingeht? Bei der Anpassung von so ‘ner Prothese. #00:14:13-4#

Teilnehmer 35: Wenn wir jetzt mal vom höher Amputierten in dem&im&im Bereich dann, im Bereich Schulter, äh&äh Schulterabgriff von den Elektroden, dass dann in den jeweiligen dynamischen Bewegungen halt bestimmte Ausfälle da sind, die Hand dann dementsprechend zu steuern (Interviewerin: Mhm (bejahend)) oder Signale kommen durch die dynamische Bewegung, die die Hand ansteuern, wo erst gar nicht will. (Interviewerin: Mhm (bejahend)) Das sind dann nochmal Sachen, die kriegt man erst im Laufe der Zeit, wenn man bei Erstversorgung, also jetzt nicht nur Erstversorgung jetzt von dem Patienten selber, sondern wenn er selber bei einem ist, dann erstmal im Laufe der Zeit mit, wo dann so die kleinen Häkchen sitzen, die man dann lösen muss. (Interviewerin: Mhm (bejahend)) Aber generell, wenn der Schaft sitzt, die Übertragung funktioniert und äh man hat das ja vorher im Test dann mehr oder weniger erarbeitet, dann sollte es eigentlich an dem Wege da, keine großen Probleme mehr geben. #00:15:09-7#

Interviewerin: Ok. Ist das bei den Anderen auch so? Oder treten da doch irgendwelche Schwierigkeiten-, oder worauf, wenn ich das, vielleicht&vielleicht formulier ich das um und nicht Schwierigkeiten, sondern wenn ich eben so ’ne Anpassung mach, was ist besonders zeitintensiv. Eben klar, der Schaft, die Anpassung davon, aber wo muss man besonders gut gucken, dass es passt, dass das richtig sitzt und richtig funktioniert? #00:15:31-2#

Teilnehmer 32: Das Training ist&ist sehr, sehr wichtig. #00:15:31-9#

Teilnehmer 30: Das ist dann das was Zeit kostet. Ich mein das Bauen ist nicht so

das Problem, aber der&der Patient muss dann lernen damit umzugehen. (Interviewerin: Mhm (bejahend)) Das ist ähm- #00:15:42-0#

Teilnehmer 34: Und dann eventuell vielleicht auch ‘n Ergotherapeuten dann hinzuzuziehen, der wirklich mit dem Patienten ähm wirklich übt. Weil ich glaube üben ist das, ist das Wichtigste an der myoelektrischen Prothese. Die wirklich richtig zu handeln. Weil bei den vielen Parametern, die man mittlerweile bei den verschiedenen Händen hat, ähm ist das, ist das wirklich wichtig. (Interviewerin: Mhm (bejahend)) #00:16:05-6#

Teilnehmer 35: Aber da haben wir ja eigentlich schon den größten Mangel in dieser Kette. (Interviewerin: Wo?) Also gute Techniker, die mit der Technik umgehen gibt’s genug, Physiotherapeuten die mit unserer Arbeit wirklich einfach übergreifend arbeiten und loslegen, ohne dass von uns einer dabeisteht, äh joah, ‘ne? Würd‘ ich mal sagen, Hauch. #00:16:25-6#

Teilnehmer 30: Da wird’s dann schon dünn. (Interviewerin: Mhm (bejahend)) #00:16:30-0#

Teilnehmer 35: Also ‘s gibt sie, aber ähm es&es nicht einfach so im Zugriff. Vielleicht in Ballungszentren. #00:16:35-0#

Teilnehmer 30: Das ist, also ich glaub einfach auch Motivation von den Patienten dann (Interviewerin: Mhm (bejahend)). Weil viele haben einfach die&die Erwartung: „Ah ja, ich krieg dann ‘ne Prothese und dann ist alles wieder so wie vorher.“. (Interviewerin: Mhm (bejahend)) Und dann ähm kriegen sie die Prothese und merken: „Scheiße, jetzt muss ich ja lernen damit umzugehen.“. (Interviewerin: Mhm (bejahend)) Und dann (geht?) der Fall und dann liegt das Ding irgendwo im Schrank und sagt: „Ach ich nehm‘ das andere da-„. Also das kann man vielleicht auch vorher nicht unbedingt immer bei jedem so wirklich äh vorher feststellen. (Interviewerin: Ja.) Weil die sagen: „Natürlich, klar bin ich motiviert und will das lernen.“ und ähm ja. Wenn ‘se dann merken was mit wie viel Arbeit das verbunden ist (Interviewerin: Mhm (bejahend)) glaub ich echt wirklich, dass da dann eher die Probleme liegen, in der Akzeptanz. #00:17:16-4#

Interviewerin: Aber ist das, ganz kurz, ist das dann auch manchmal ‘n Problem ähm wenn man die Prothese überhaupt anpasst oder aussucht welche Versorgung jemand bekommt, ist das auch ‘n Punkt wo man dann sagt eben, wenn die Motivation nicht stimmt, dass man dann doch was andres an&ans Herz legt? Oder einfach sagt: „Sowas ist vielleicht nicht geeignet.“? #00:17:33-7#

Teilnehmer 30: Gut, aber man (Interviewerin: Klar, man kann’s nicht immer wissen.) das&man kann’s nicht, man kann in die Leute nicht reingucken oder kann sich auch nochmal was ändern bei dem oder so, also des. Gut, klar, wenn man schon weiß: „Okay, mh wird nichts werden.“, dann äh kann man den ja schon versuchen dahingehend zu beraten, (Interviewerin: Mhm (bejahend)) aber ich mein, wenn er sagt: „Ich will das aber haben.“ und ja, dann will er das dann. #00:17:58-5#

Interviewerin: Ja. #00:17:56-7#

Teilnehmer 30: Sind wir ja soweit auch noch äh, wollen ja auch von was leben.

Wenn der kommt und sagt er will das und die Kasse genehmigt, dann bau ‘n wir‘s ihm halt und äh sieht er’s halt ob’s klappt oder nicht. (Interviewerin: Ja.) Also ich mein man, vielleicht überzeugt er einen ja doch noch vom Gegenteil, also (Interviewerin: Mhm (bejahend)) auch schon jetzt Beinprothesenversorgungen gehabt, also wo ich gesagt hab: „Pf, niemals läuft der.“ und vier Wochen später stand er da. (Interviewerin: Mhm (bejahend)) Also. ja. #00:18:29-8#

Interviewerin: Mhm (bejahend) #00:18:29-8#

Teilnehmer 35: Also Motivationskurven die brechen schon mal auch nach ‘ner Zeit, wenn man so die&die ersten Prozente seines Weges da äh hinter sich hat und äh die&die Anfangsphase ist ja meist, wenn man erstmal so den ersten Schritt, diesen Aha-Effekt gehabt hat, geht das ja. Wie immer im Leben: Die ersten 70 Prozent äh vom&vom Ziel was wir erreichen ist äh, gehen nachher wesentlich schneller als die letzten 30. (Interviewerin: Mhm (bejahend)) Weil die, die s-&die Steps werden dann kleiner und mit mehr Aufwendung das immer perfekter zu machen (Interviewerin: Mhm (bejahend)). Da bricht natürlich schon mal einer zusammen. Aber auch das, würde ich sagen, ist jetzt kein Weltuntergang ähm zumindest weiß der dann äh, dass er dafür noch nicht so bereit ist. (Interviewerin: Mhm (bejahend)) Und ähm dann muss er sich aber drei, vier Jahre später oder wenn’s dann durch irgendwelche anderen Sachen äh&äh zum Vorw-&zum&zum Vorschein tritt, dann keine Sorgen machen, dass er’s nicht probiert hat. Ähm das Schlimmste ist immer, wenn man nachher feststellt man hätte es machen sollen und hat’s nicht getan. Glaube das sind die größten Ärgernisse, die&die (kommt?) als festzustellen ich hab’s probiert und es geht nicht. Damit kann jemand äh über die Zeitdistanz besser leben als in der Nachsorge zu sagen: „Hätte ich es mal probiert.“. So. (Interviewerin: Mhm (bejahend), ja.) #00:19:44-4#

Teilnehmer 31: Was auch nicht ganz einfach ist, mit dem Bewusstsein zu leben grade für 60.000 ‘n äh kompletten Arm versemmelt zu haben, weil er nicht genutzt wird, weil der Krebspatient hinterher in ‘ne schwere Depression geht und sagt: „Ich mag das (nicht?).“. Am ersten Tag unterschrieben, noch schön, noch zwei Mal geübt, danach gesagt: „Das will ich nicht leisten. Das kann ich nicht leisten.“ Tut auch weh. #00:20:11-9#

Teilnehmer 35: Tut weh, aber ich sag jetzt mal, ist halt kein äh persönliches worst case. Muss man mit umgehen können, man hat’s ja nicht in der Hand. Aber soll ich derjenige sein, der jemandem diesen Versuch, wenn er ihn wirklich überzeugend darstellt, soll ich derjenige sein der sagt: „Nee.“? Ähm, ich sag mal, das ist ‘n (...?) Problem, also außer ich hab‘ jemanden vor mir sitzen, bei dem ich schon komplett zweifle, äh dass er überhaupt weiß äh warum er bei mir sitzt, dass er geschickt worden ist und nicht selber gekommen ist, so ungefähr. (Interviewerin: Mhm (bejahend)) Dann kann man das näher eruieren. Aber dadurch, dass in diesen Versorgungen im Vorfeld, bis es zu einer Definitivversorgung kommt, da hat man ja schon ‘n Weg hinter sich. (Interviewerin: Mhm (bejahend)) Also keine Krankenkasse jedenfalls, die die ich bisher kennengelernt habe macht da ‘n Stempel drunter und sagt das Go. Die wollen alle Testversorgungen, dann MDK-Vorstellung wenn’s noch geht und&und&und. Das heißt man hat ‘ne&ein Testszenario hinter sich, (Interviewerin: Mhm (bejahend)) wo man mehr über den Patienten weiß, mehr über die Rahmenbedingungen, eigentlich ihn auch schon näher kennengelernt hat und äh ich glaube, wenn man das erstmal so weit hinter sich hat und selber sagt: „Ok,

definitiv können wir bauen, die technischen Grundlagen sind da, ähm der will, der macht auch bestimmte Fortschritte.“. Man merkt ja auch wie sich jemand verhält. (Interviewerin: Mhm (bejahend)) Und dann irgendwann kommt der&der Einbruch danach. Gut, dann ist es so. (Interviewerin: Mhm (bejahend)) Also ich glaube äh die Welt hat schmerzvolleres zu verbeißen als das. #00:21:40-4#

Interviewerin: Mhm (bejahend). Herr (Name von Teilnehmer 33 aus Datenschutzgründen ausgelassen) Sie wollten vorher was sagen, ich hab‘ Sie unterbrochen. #00:21:44-5#

Teilnehmer 33: Kein Problem, aber ich glaube, also bei uns ist es zumindest so, bei uns ist schon vom Erstkontakt die Therapie dabei. (Interviewerin: Mhm (bejahend)) Und die Therapie begleitet auch über die Testphasen hinweg und die Therapie kann auch ganz genau sagen, in welche Richtung die Entwicklung ist und ich glaub es ist für ’n Techniker keine Schande, wenn er ‘ne Quantum-Hand oder ‘ne Michelangelo-Hand angedacht hat und irgendwann auf dem Weg sagt: „Der Patient schafft das nicht und er verkraftet das nicht.“. ‘N Schritt zurück zu machen, ich glaub das ist dem Patienten gegenüber fair, dem Kostenträger gegenüber fair und wir verlieren auch kein Gesicht mal zu sagen: „Das was angestrebt war ist so nicht zu erreichen.“. Und darum denk ich ist die Therapie, zumindest bei uns hier im Haus, ist von der ersten Stunde dabei und hat hinterher sicherlich auch bei den Feinheiten den größeren Part an Arbeit als wir Techniker (Interviewerin: Mhm (bejahend)) und ich glaub unsre Therapie ist so feinfühlig und macht die letzten 30 Prozent eigentlich ohne uns. Und wenn dann wirklich noch Nachjustierungen sind oder irgendwas, sind wir dabei aber ansonsten brauchen die uns auch nicht da. (Interviewerin: Mhm (bejahend)) #00:22:52-3#

Teilnehmer 32: Auch für die Motivation dann. #00:22:55-0#

Teilnehmer 33: Ja. #00:22:53-2#

Teilnehmer 32: Auch bei Kindern dann- #00:22:57-1#

Teilnehmer 31: Sieht auf ’m flachen Land allerdings etwas anders aus, da muss ich (Teilnehmer 35: Ich wollte grade sagen, das sind die Rahmenbedingungen, die&die hat man auf der Pläne nicht.) im&im Vorfeld der-. Also das sind optimale Bedingungen, ganz klar. (Interviewerin: Mhm (bejahend)) Äh die Realität, sagen wir mal auf der grünen Wiese, sieht halt definitiv aus, dass ich schon in der Vorbereitung der Versorgung gucke äh welche Therapeuten ich da mit rankriege. ‘Ne? Das ist also in meinem Fall eigentlich immer schon (tun?) äh auch hier, mal gesagt nicht speziell nur bei der Armprothetik äh- #00:23:26-4#

Teilnehmer 33: Ich glaub es gibt auch immer weniger Häuser auf ’m flachen Land, die so komplexe Versorgungen machen, ‘ne? #00:23:31-3#

Teilnehmer 31: Kommt mit dazu. Nichtsdestotrotz äh sagen wir mal, wenn einen diese Technik äh in seinem Berufsleben schon die ganze Zeit äh begleitet, man jedes Mal diese Versorgung durchgeführt hat, sich auf dem gleichen Stand hält, auf dem aktuellen Stand hält, denk ich ist es mh ja auch wirklich sinnvoll jemanden vor Ort auch auf der grünen Wiese zu versorgen, weil er schlicht und ergreifend natürlich auch schon irgendwie kurze Anfahrtswege und dergleichen (liebt?), auf der andern

Seite natürlich auch die Nachsorge. Einfach durch kurze Wege (...??) muss. (Teilnehmer 33: Gut, da muss-) Aber es muss eben sichergestellt werden, da bin ich vollkommen gleicher Meinung, äh dass in dem Versorgungsprozess alle Notwendigkeiten bereitgestellt werden. Das heißt also ich muss im Vorfeld sehen, hab‘ ich tatsächlich dafür dann die Ergotherapeutin in der Nähe, die das auch kann. Oder die zumindest bereit ist mal auch mit mir zu anderen Ergotherapeuten zu fahren, sich dort mal zu un-&unterweisen zu lassen, um dann den Kunden auch langfristig auch weiter zu betreuen. #00:24:34-5#

Interviewerin: Ich würd‘ hier mal unterbrechen, um bei der Frage nochmal zu bleiben. Und zwar Herr (Name von Teilnehmer 33 aus Datenschutzgründen ausgelassen) haben Sie gerade gesagt so in den letzten 30 Prozent betreuen die Therapeuten dann selbstständig und dann gibt’s manchmal Sachen die nachjustiert werden müssen. Was sind das denn vor allem für Sachen, was kommt da dann nochmal auf, wo man sagt da stimmt noch was nicht, da muss man an der Prothese nochmal was verändern? #00:24:54-9#

Teilnehmer 33: Also häufig ist es ja so, dass die Muskelgruppen eben doch durch die Trainingszeit auftrainiert werden (Interviewerin: Mhm (bejahend)), dass wir im Vorfeld Elektroden zu hoch eingestellt haben, nicht sensibel genug eingestellt haben (Interviewerin: Mhm (bejahend)), dass die Prothese dann bei grade Überkopf-äh-hantierungen überreagiert, dass man dann die Elektroden nochmal hier nachjustiert. (Interviewerin: Mhm (bejahend)) Das sind eigentlich so die groben Sachen, die im Nachlauf dann noch passieren. Also alles andere, denke ich, ist im Vorfeld abgefrühstückt, aber das&das haben wir dann schon auch immer wieder nochmal. #00:25:29-3#

Interviewerin: Dass man einfach in der Handhabung merkt, wo noch irgendwas nicht ganz so rundläuft? #00:25:33-6#

Teilnehmer 33: Genau. #00:25:31-8#

Teilnehmer 35: Oder den Veränderungen entsprechend anpassen, ‘ne? #00:25:36-2#

Interviewerin: Mhm (bejahend) #00:25:36-2#

Teilnehmer 35: Das sind halt, auch der eine Modus, also dass im Ruhemodus zum Beispiel nicht immer irgendwelche kleinen Ansätze der Hand dann immer dieses „did-did-did“ (Interviewerin: Mhm (bejahend)). Erstmal geht’s auf den Akku, wenn man dran arbeiten will, ist das Ding schon durch die tausend Mal Leerlauf da äh belastet und das sind Sachen, die verändern sich grade in diesem, in diesem Bereich über ‘ne gewisse Zeit. (Interviewerin: Mhm (bejahend), ja.) #00:25:55-6#

Teilnehmer 34: Das ist die Hauptnachsorge, ‘ne? Elektroden einstellen. #00:25:59-6#

Teilnehmer 35: Das ist. Ja. Ich meine heute sind’s ja nicht nur Elektroden, heute (sollen?) Parameter über die Software (...?). Aber letztendlich geht’s um das Gleiche. (Interviewerin: Mhm (bejahend)) #00:26:08-0#

Teilnehmer 33: Und vielleicht noch ein Problem was immer wieder mal vorkommt, ist bei Traumaamputationen. Dass die Stumpfverhältnisse sich dann doch ändern durch das Tragen der Prothese, dass der&dass die Schaftform halt einfach (Interviewerin: Mhm (bejahend)) nicht mehr gegeben ist. Das, also das kann auch passieren, das ist einfach so. #00:26:24-2#

Teilnehmer 35: In den ersten zwei, drei Versorgungen so durchgehend, hat man schon mehr mit, mit ‘ner, ja komplettem eigentlich Neuaufbauten zu tun. (Interviewerin: Mhm (bejahend)) Dass Muskelgruppen wieder da sind. Je nachdem wo’s ist auch jetzt im Schulterbereich, wenn’s also kurze Oberarmstümpfe sind, ähm da verändern sich auch Dinge dann. (Interviewerin: Ja.) Manchmal zum Positiven, manchmal auch zum Negativen. #00:26:47-7#

Interviewerin: Und jetzt, Herr (Name von Teilnehmer 31 aus Datenschutzgründen ausgelassen), haben Sie ganz am Anfang gesagt, was Ihnen als erstes auffällt ist so das Optische, passt das mit rein. Gibt’s da irgendwas, was ‘ne große Schwierigkeit darstellt, wenn man so ‘ne Prothese anpasst, um die einfach auch, weiß nicht eben optisch an ‘nen Menschen anzupassen? #00:27:04-6#

Teilnehmer 31: Na, es ist die Frage bezogen auf die Situation, dass ‘n vorhandenes Hilfsmittel da ist. Das heißt eigentlich, also ‘n ersten optischen Check zu machen (Interviewerin: Mhm (bejahend)). Ähm von daher ist ja dann die Anforderung in der Fertigung an mich gestellt. Wie äh gliedere ich dieses Hilfsmittel definitiv dann optisch oder kosmetisch an. (Interviewerin: Ja.) Und da sind wir dann bei dem Begriff Kosmetik. Da geht’s dann sicherlich um ‘n Teil mit dem wir auch kräftig zu tun haben. (Interviewerin: Mhm (bejahend)) Inwieweit lässt sich ‘n Hilfsmittel mit immer mehr Funktionen, grade Kinder waren (immer?) schon mal im Gespräch, äh aber wie lässt sich so ’n Hilfsmittel einordnen, ‘ne? (Interviewerin: Mhm (bejahend)) Ohne dass es je nach Kundenwunsch und ich sag mal, aus meinem Erfahrungsbereich ist der immer noch vorwiegend, der sagt ich möchte das Ganze doch unauffällig haben (Interviewerin: Mhm (bejahend)). Das heißt also lockeren, kurzärmligen mit voller Technik laufen so viel in meinem Erfahrungsbereich nicht rum. (Interviewerin: Mhm (bejahend)) Ähm das heißt also, das sind dann schlicht und ergreifend die Aufgabe dann im Versorgungsverlauf. #00:28:11-6#

Interviewerin: Mhm (bejahend). Und wo sind da die Schwierigkeiten? Damit das eben unauffällig mitreinpasst und so dem Wunsch des Patienten entspricht? #00:28:18-8#

Teilnehmer 31: Naja gut. Ähm konkret ist das Gewicht des Hilfsmittels äh Auslöser schlicht und ergreifend, dass urplötzlich Verzug in Schultern kommt, dass was weiß ich, die betroffene Seite, weil alles drangepackt ist, vom Schultergelenk über ’n Ellbogen und dann auch noch ‘ne super Hand dran, dann im Laufe der Zeit definitiv auf der Seite definitiv ‘n deutlichen Schulters-&äh Tiefstand zeigt. Das heißt also im Endeffekt dann auch die Wirbelsäule beachtet werden muss. (Interviewerin: Mhm (bejahend)) Wird tatsächlich dann auch äh in der Therapie oder dergleichen noch Muskeltraining gemacht, um das auszugleichen. Also es sind schon Überlegungen, was ist äh muskulär auch möglich, das Hilfsmittel tatsächlich zu halten, ohne dabei ‘n Körperschema vollkommen aufzugeben, ‘ne? (Interviewerin: Mhm (bejahend)) Was bringt es mir im Endeffekt, wenn ich hinterher ‘n-&‘ne Skoliose nachbehandeln muss, ‘ne? #00:29:12-1#

Interviewerin: Und hat man das als Techniker irgendeinen Spielraum, da irgendwas an der Prothese so zu verändern oder, also was&was hab‘ ich als Techniker für ‘ne Möglichkeit da eben irgendwas zu verändern, um sowas zu reduzieren? #00:29:26-0#

Teilnehmer 31: Wenn man die Zeit dazu hat: Passteilsuche. Gucken was passt zur Funktion und was ist grade aktuell äh vom Passteil her mit gleicher Funktion, vielleicht etwas leichter oder wie auch immer. (Interviewerin: Mhm (bejahend)) #00:29:35-2#

Teilnehmer 35: Wobei wir da ja an die Grenzen stoßen, wenn wir jetzt hier über die Myoelektronik reden. Äh, ich sag mal, ‘ne? Die Hände die da im Stand der Technik arbeiten, mh-. Reden wir über Gewichtsunterschiede, die glaub ich ‘ne Skoliose weder verhindern können noch sonst was. Wobei man auch sagen muss, äh dass äh wenn man keine, wenn man also Armamputationen nicht versorgt, die Fehlbildungen wesentlich drastischer sind (Interviewerin: Mhm (bejahend), vor allem in der Wirbelsäule. Und ähm wenn man schon über Gewicht, weil das Thema Gewicht ist ja immer so ’n ganz nettes Thema, aber ähm man sollte mal ‘ne Myoprothese auf ’n&auf die Waage legen und ’n frisch abgeschnittenen Arm, der jetzt nicht irgendwie in Teilstückchen zerrissen worden ist auf ‘ne Waage legen und dann würde man sehen: Wir sind da noch weit von weg, was da fehlt (lacht). Das ist halt nur, es ist anders angebunden und dadurch wird das Gewicht natürlich nachher anders wahrgenommen. Und in den Handversorgungen sitzt das schwerste Bauteil auch noch am ga::nz am Ende. (Interviewerin: Mhm (bejahend)) #00:30:36-0#

Teilnehmer 31: Über den (Muskelzug?) (Teilnehmer 35: Also ich würde mir persönlich zum Beispiel wünschen, dass die, dass die Motoren und diese Sachen eventuell so leicht werden, oder halt woanders sitzen.) (…????) #00:30:41-7#

Interviewerin: Aber das heißt da ist der Spielraum eingeschränkt, weil das einfach so vorgefertigt ist und man- #00:30:55-7#

Teilnehmer 35: Ganz genau. Das hol ich ja nur raus und verbinde es und versuche die Adaption so gut wie möglich zu machen (Interviewerin: Mhm (bejahend)). Dafür sind wir da. Aber die Passteile können wir halt im Moment nicht beeinflussen. (Interviewerin: Ja.) #00:31:06-4#

Interviewerin: Und Herr (Name von Teilnehmer 30 aus Datenschutzgründen ausgelassen), Sie wollten grade noch was ergänzen? #00:31:08-6#

Teilnehmer 30: Ähm achso ja, es&wegen&also jetzt, wegen Kosmetik. #00:31:15-0#

Interviewerin: Ja! #00:31:15-0#

Teilnehmer 30: Kommt ja auch immer drauf an, grade bei&auf’s Amputationsniveau. Also grade, wenn man jetzt von äh myoelektrische Hände, wenn man jetzt ‘n sehr langen Stumpf hat oder so (Interviewerin: Mhm (bejahend)) äh wird’s halt immer schwierig die Teile dazwischen zu kriegen (Interviewerin: Mhm (bejahend)). So. Das ist halt noch ‘n Ding, wo man halt unter der Auswahl der Passteile gucken muss:

(Interviewerin: Ja.) Ist das überhaupt machbar, sinnvoll. Ja. #00:31:37-2#

Interviewerin: Zu diesem kosmetischen Problem, gibt's da noch irgendwas, was jemand hinzufügen möchte, eben was da die Schwierigkeit ist das einfach auch an den Körperbau, an ‘ne Person anzupassen? #00:31:48-8#

Teilnehmer 32: Wenn die Passteile zu groß sind, ‘ne? #00:31:49-7#

Interviewerin: Mhm (bejahend) #00:31:48-4#

Teilnehmer 32: Das ist das größte Problem dann. #00:31:53-1#

Interviewerin: Mhm (bejahend) #00:31:53-1#

Teilnehmer 31: Oder die Akkus. Hab heute ‘ne Hand auf den Tisch gekriegt, sind übergroße AA (alle lachen) Akkus dabei, ‘ne? (Teilnehmer 35: Ja ok, ich wollte jetzt heute nicht über die Probleme (...?)) Also ich sag mal, aus den Zeiten sollten wir eigentlich raus sein, aber es ist ‘ne, also die war ja auch ‘ne aktuelle Hand, ‘ne? Äh aber ja, (was willste machen?). #00:32:09-1#

Teilnehmer 32: Ja sie arbeiten dran, ‘ne? (lacht) #00:32:13-4#

Teilnehmer 33: Genau, kam gestern Nacht im Fernsehen: Vinzenz-Hände haben jetzt einen knetbaren Akku, ich schätz mal vier Millimeter stark, 80 Millimeter lang mit USB Anschluss zum Laden. Hervorragend. Kann man direkt am Arm anformen, trägt kaum auf. #00:32:30-2#

Teilnehmer 31: Sind sogar nominiert für den Zukunftspreis mit, ‘ne? #00:32:35-4#

Teilnehmer 33: Wie bitte? #00:32:30-2#

Teilnehmer 31: Sind nominiert zum Zukunftspreis. #00:32:35-3#

Teilnehmer 35: Wobei ’s Akkus für diesen Bereich schon gibt, die ich also grade im Unterarm sehr hoch, weil ich die schon seitlich biegen können, die gibt's ja nun schon länger, ‘ne? Deshalb keine Neuheit. #00:32:44-1#

Teilnehmer 32: Nee, aber die sind halt sehr lang und das bei Kinderversorgung ist das problem- #00:32:46-8#

Teilnehmer 35: Es ist immer, ja ist immer das Problem wo, wo krieg ich sie hin, weil in der Richtung knicken, da (ist lang?) immer das Ende des Akkus (lacht). #00:32:55-7#

Teilnehmer 31: Das Kuriose ist ja, dass man auch gar keine Probleme hätte diesen knetbaren Akku an diese Hand dranzustecken, wenn da nicht die Garantieerklärung wäre, ‘ne? #00:33:03-9#

Teilnehmer 35: Ja::. #00:33:05-4#

Teilnehmer 31: Das ist ‘n bisschen das Problem. Was weiß ich, ich meine, das darf

man bei der ganzen Geschichte nicht vergessen. Wenn ich ‘ne Testversorgung mach und ich muss mir überlegen, ob ich den 400 Euro Eingussring verwende oder nicht, es geht definitiv schließlich (…??) auch um Kosten die dabei entstehen, ‘ne? #00:33:22-7#

Teilnehmer 30: Das ist dann auch- #00:33:24-1#

Teilnehmer 33: Sie bauen Prothesen aller Couleur in Ihrem Unternehmen selber? #00:33:29-3#

Teilnehmer 31: Bin ich grade dabei, ja. #00:33:30-4#

Teilnehmer 33: Und Sie sind Hersteller? #00:33:33-9#

Teilnehmer 31: Mhm (bejahend) #00:33:33-9#

Teilnehmer 33: Und welche Garantie erlischt, wenn Sie ‘n Vinzenzakku für ‘ne (..) (Teilnehmer 31: (Name eines Prothesenherstellers aus Datenschutzgründen ausgelassen) Hand nehme, dann brauch ich die (Name eines Prothesenherstellers aus Datenschutzgründen ausgelassen) Hand, wenn sie defekt ist nicht mehr einschicken.) Das glaub ich Ihnen nicht. #00:33:44-3#

Teilnehmer 31: Nee? #00:33:44-3#

Teilnehmer 33: Nee. #00:33:42-0#

Teilnehmer 35: Also man kriegt ’s schon durch, wir haben schon getauft und haben uns da auch erfolgreich gewehrt, aber ich sag jetzt mal, ganz so ohne Gegenwehr ist das da nicht. Da könnt ich also-. Ich inzwischen aus 35 Jahren, könnte da ‘n kleines Heftchen drüber schreiben, was ich schon alles machen musste, damit die Gegenseite dann so reagiert, wie es sich gehört. Ähm aber das sind Gesetzmäßigkeiten, da glaube ich, wenn der Druck von außen größer wird, wird das auch aufgeweicht. Wir haben das mit Füßen (Name eines Beinprothesenherstellers aus Datenschutzgründen ausgelassen) und da brauchen wir nicht drüber reden, darf nur der und der Fuß. Ich sag, ich hab ‘ne Dame, die hat ‘ne andere Anforderung. Entweder ich kann den Fuß drunter bauen oder es kommt das Kniegelenk nicht drunter, könnt ihr jetzt ja oder nein sagen, mir egal, nehm‘ ich ‘n andres Kniegelenk. Da kommt das „Ja.“. Man muss nur dastehen und muss sagen: „Also jetzt hier, ‘ne! Wie sieht’s aus? Wollen wir, oder wollen wir jetzt nicht?“. #00:34:36-9#

Teilnehmer 33: Ich hab‘ das jetzt nur für die obere Extremität (...?) (Teilnehmer 35: Ja ja ‘ne, ich mein jetzt nur allgemein. Aber&aber-)-. #00:34:44-5#

Interviewerin: Ja genau, um da&um da beim Thema zu bleiben, ich werd&würde da nochmal gerne unterbrechen. Das heißt aber, dass auch ‘n Problem ist, dass einfach die Akkus die mit dabei sind so lang sind oder so groß sind, dass das einfach grade in der Kinderanpassung ähm nicht reinpasst oder wie darf ich das verstehen? #00:34:58-8#

Teilnehmer 32: Oder halt zu wenig Leistung ist. Gibt’s wirklich ganz kleine Akkus, aber die- #00:35:01-3#

Interviewerin: Die haben dann die Leistung nicht? #00:35:02-4#

Teilnehmer 33: Genau, ja. #00:35:00-6#

Interviewerin: Mhm (bejahend). Ja, ok. Gibt’s zu diesem kosmetischen Aspekt noch irgendwas, was gerne jemand hinzufügen würde, was es da- #00:35:11-0#

Teilnehmer 30: Also da ist natürlich das Finanzielle. Also ich mein man kann wunderbare Kosmetiken machen (Interviewerin: Mhm (bejahend)) in Silikon mit Haaren und allem dran äh, aber da kann man dann halt auch mal schnell nochmal ‘n paar tausend Euro extra drauflegen (Interviewerin: Mhm (bejahend)). Und das zahlt halt zum Beispiel die Krankenkasse nicht. #00:35:28-5#

Interviewerin: Ok, ja. #00:35:28-5#

Teilnehmer 30: Das ist jetzt mal- #00:35:29-4#

Teilnehmer 35: Ich glaube aber, dass äh das kosmetische Abbild gar nicht das Problem ist. Also ich habe ganz wenig bei oberen Extremitäten, wenn wir das jetzt mal darauf rein beschränken, ähm es wird aufgeweicht, es ist nicht mehr so wie früher, dass alles hautfarben sein muss und mit so nem&so viel wie möglich überall verdeckt sein muss, also man geht da schon offensiver mit um, bei den Jüngeren sowieso, die machen den Schritt nach vorn und sagen: „Und? Dann hab‘ ich halt ‘ne Terminatorhand. Früher fanden wir’s geil, weil wir die Filme gesehen haben wie das aussieht und jetzt haben wir selber eine.“. Ähm ist zwar selten, dass es so offensiv gemacht wird, aber es weicht aus&auf. #00:36:06-5#

Interviewerin: Von Seiten der Patienten, (Teilnehmer 35: Von Seiten d-. Ganz genau.) dass die Forderung nicht mehr so groß ist? #00:36:07-3#

Teilnehmer 35: Dass die lieber sagen heute: „Nee, kein hautfarbenen Schaft. Ich will dann lieber,“ sagen wir mal „Carbon mit ‘nem Abbild,“ oder eben „macht mir ‘n neutralen, ich lass mir da ‘n Tattoo drauf-äh-lackieren.“ oder irgendwie sowas. Also die Sachen glaub ich werden in Zukunft nicht die Diskussion. (Interviewerin: Mhm (bejahend)) Im Gesamtabbild. Ähm bei den Handbreiten, Längen und der Darstellung hat man dann schon mal (Interviewerin: Mhm (bejahend) Erstkontakt, wenn man da sowas liegen hat (lacht) dann schon mal ‘n paar große Augen (Interviewerin: Mhm (bejahend)) ähm, dass das dann in bestimmten Situationen dann halt schon mal ‘n bisschen von den eigentlichen Größen wegläuft. (Interviewerin: Mhm (bejahend)) #00:36:45-2#

Interviewerin: Ja. Und wenn wir uns jetzt weiter vorstellen, dass eben jemand diese Prothese angepasst bekommen hat und eigentlich erstmal alles stimmt. Und dann hab‘ ich jetzt aber bei den Gesprächen mit vielen Prothesenträgern rausgehört, dass es oft der Fall ist, dass die dann eben doch nicht getragen wird. Wie wir’s vorher hatten, die landet dann irgendwo im Schrank und wird nicht benutzt. Aus Ihrer Sicht: Woran liegt das denn? Also was ist so die Hemmschwelle, dass viele dann so ’n eigentlich so ’n Gerät ja Zuhause haben aber das dann nicht genutzt wird? #00:37:12-4#

Teilnehmer 32: Teilweise haben sie sich mehr drunter versprochen. #00:37:14-7#

Interviewerin: Mhm (bejahend) #00:37:15-3#

Teilnehmer 32: Ja, dass die halt noch mehr können (Interviewerin: Mhm (bejahend)) und dass sie praktisch die Hand, die fehlende, ersetzen und ähm das machen wir auch so, dass wir gleich v-&eigentlich von Anfang an auch den Leuten die vorher noch keine Prothesen getragen haben schon auf den Weg geben, dass das halt ‘n Ersatz ist, ‘n Hilfsmittel, aber halt auf gar keinen Fall ‘ne vollwertige Hand (Interviewerin: Mhm (bejahend)) wieder wird, ‘ne? (Interviewerin: Ja.) Und ähm ja, bei Oberarmprothesen, kurzen Oberarmstümpfen haben wir’s auch schon oft gehabt, des Öfteren gehabt (Interviewerin: Mhm (bejahend)), dass halt das Gewicht ähm grad mit ’m DynamicArm zum Beispiel waren ‘se anfangs begeistert, auch Herr (Name des Patienten aus Datenschutzgründen ausgelassen) zum Beispiel, ‘ne? Auch ‘ne (Therapie?) alles gut, aber dann später, wenn man sich dann mal wieder getroffen hat, dann doch eher seltener benutzt wird das Ganze, ‘ne? #00:38:02-3#

Interviewerin: Weil’s dann doch zu schwer ist und einfach Probleme macht? #00:38:06-1#

Teilnehmer 32: Genau, jetzt bei den Oberarmprothesen speziell, ‘ne? #00:38:05-5#

Interviewerin: Ja, mhm (bejahend). #00:38:08-6#

Teilnehmer 30: Eben, weil der Aufwand halt im Vergleich zu dem Nutzen, den sie im Alltag drauß haben einfach äh gering&geringer ist als gedacht (Interviewerin: Mhm (bejahend)). #00:38:16-7#

Teilnehmer 35: Ich glaube, dass auch manchmal die menschliche Eigenschaft, die man „Bequemlichkeit“ nennt, äh ‘n ganz großer Faktor ist. Wir sind halt alles Menschen (Interviewerin: Mhm (bejahend)) und wir werden auch mal von Unfug geleitet, der uns in ‘ne Ecke bringt, wo wir eigentlich nicht hinwollten und äh solange jemand dabei ist (kurze Pause) der einen darauf stößt, der ermahnt vielleicht oder der das g-&der das einfach immer wieder zum Anstoß macht. „Mach das bitte so.“ oder „Wir können so mehr machen.“, aber Zuhause in den eigenen vier Wänden, da ist der Mensch eventuell mit sich ganz alleine und da sieht die Welt halt anders aus. #00:38:55-5#

Interviewerin: Mhm (bejahend). Und Bequemlichkeit aber inwiefern? Weil wenn ja ‘ne Prothese perfekt funktioniert und einfach zu handhaben ist, dann könnte, also dann könnte man ja davon ausgehen, dass sie halt ‘n Hilfsmittel ist und&und ‘ne Erleichterung bringt, dann wär‘ ja das Bequemere sie zu tragen? #00:39:11-0#

Teilnehmer 35: Das ist richtig. Jetzt kommt aber dann so der&der Gewohnheitsfaktor. Hab‘ ich jemand, der schon lange ohne versorgt war, dann kann der eigentlich tierisch viel, ‘ne? Ohne. Weil die Not macht erfinderisch. (Interviewerin: Ok, ja.) Und den muss ich erstmal davon wegholen. Hab‘ ich jemanden, der frisch amputiert ist und der verhältnismäßig schnell nach der Amputation versorgt wird und diesen Leerlauf quasi so gar nicht kennt, ähm da hab‘ ich noch nicht so oft festgestellt, dass es letztendlich gar nicht so oft benutzt wird, beziehungsweise im Schrank liegt, sondern eher, eher den&den anderen Effekt, wenn ich dann nicht

schnell reparieren konnte, wurde schon stündlich angerufen: „Wie sieht's denn jetzt aus?“. (Interviewerin: Mhm (bejahend)) Also man hat da beide Fälle. Das ist je nachdem wie der Verlauf halt auch gewesen ist (Interviewerin: Mhm (bejahend)). #00:40:00-7#

Interviewerin: Gibt's da noch? Ja- #00:39:59-7#

Teilnehmer 33: Ich glaub halt, man kann das auch nicht an Tragdauern festmachen. Wenn ich jetzt 'n Oberarmamputierten habe, der nur Samstag, Sonntag, wenn er Motorrad fahren will, 'ne Oberarmprothese trägt (Interviewerin: Mhm (bejahend)) und trägt die dann in der Woche vielleicht acht Stunden und sonst liegt die im Regal, aber er sagt: „Ich verbessere meine Lebensqualität, ich kann wieder Motorrad fahren.“ (Interviewerin: Mhm (bejahend)), dann hab' ich für den im Leben 'n ganz großen Schritt gemacht. Und dann liegt die Prothese eben vielleicht von Montag bis Freitag oder (seine Handhabung ohne macht im Schrank?) aber er kann seine Freizeitaktivität nutzen oder er braucht die Prothese für bestimmte Dinge. Eben 'ne geringe Tragedauer, wie Herr (Name vom Patienten aus Datenschutzgründen ausgelassen) auf der Arbeit, seine Stunden und dann sagt: „Dann ist mir die einfach zu schwer. In meiner Freizeit trag ich die nicht.“. Dann ist das völlig in Ordnung. (Interviewerin: Mhm (bejahend)) #00:40:48-2#

Teilnehmer 34: Ich glaub am Ende ist es immer wieder das Gewicht, was&was die Tragedauer definitiv verkürzt. #00:40:55-3#

Teilnehmer 35: Bei bestimmten Versorgungen. Aber wenn sie so benutzt wird, dann ist sie für mich in der Nutzung. (Teilnehmer 31 und andere: Mhm (bejahend)) Also die Darstellung jetzt, würde für mich nicht bedeuten: Ich muss jetzt mal überlegen den da hinzukriegen, dass er die 24 Stunden am besten trägt. Also das wär' dann auch schon wieder 'n kleines Verbrechen. #00:41:11-8#

Teilnehmer 30: Darum geht's ja auch nicht. #00:41:13-3#

Teilnehmer 35: Das ist f-&wäre für mich eine absolute Rechtfertigung alles zu tun. #00:41:15-6#

Teilnehmer 30: Klarer Gebrauchsvorteil- #00:41:19-6#

Teilnehmer 35: Ähm ich&ich muss nicht-. Ja. #00:41:21-2#

Teilnehmer 30: Warum soll er das Ding nicht kriegen? #00:41:20-9#

Teilnehmer 35: Ich muss sie nicht jede Minute am Tag tragen. (Interviewerin: Ja.) Also das ist auch gar nicht, ich glaube das ist auch gar nicht wo wir hinwollen. Ich glaube grade der Aspekt, was eben gefallen ist, halt diese Aufwertung äh der Lebensqualität auch wenn es immer nur für bestimmte Phasen sind, äh sind da 100 Prozent Rechtfertigung, dass das so gelaufen ist und was er damit macht, dass es in Ordnung ist. #00:41:39-1#

Teilnehmer 31: Also von daher müssen wir Ihren Ansatz präzisieren. Sie meinen <u>die</u> Hilfsmittel, die nun <u>definitiv</u> auf Dauer in der Schublade sind wahrscheinlich. Und da haben Sie relativ starke Rückmeldung gekriegt (Interviewerin: Ja.), dass&dass doch

in vielen Fällen so passiert. (Interviewerin: Ja.) #00:41:53-1#

Interviewerin: Wobei ich’s gar nicht so schlecht finde, wenn die Frage gar nicht so klar für alle ist, weil dadurch auch noch Punkte dazukommen, die eben äh sonst oft einfach unter ’m Tisch bleiben. (Teilnehmer 31: Mhm (bejahend)) Ähm was gibt’s denn für Situationen, jetzt zum Beispiel das Motorrad fahren, für die explizit ‘ne Prothese gefordert wird? Gibt’s sowas, dass jemand kommt und sagt: „Genau für sowas brauch ich ‘ne Prothese.“? Und was sind das für Situationen? #00:42:12-1#

Teilnehmer 35: Ja, ganz genau. Das sind nicht wenige. #00:42:14-2#

Interviewerin: Ja, was für welche denn? #00:42:17-1#

Teilnehmer 35: Na, ich mein, dass die den Anspruch haben. Also ich hab‘ jetzt schon in der Zeit, ich glaube, weiß nicht, in meiner Laufzeit glaub ich 10,12,13 Prothesen rein nur zum Motorrad fahren gemacht. #00:42:25-8#

Interviewerin: Motorrad fahren? Ok. #00:42:29-2#

Teilnehmer 35: Motorrad fahren, ja. #00:42:28-3#

Interviewerin: Mhm (bejahend). Und gibt’s noch andere so Situationen? Wo, eben wo da die Prothese fehlt, wo die dann benutzt wird, die dann gefordert sind? #00:42:38-6#

Teilnehmer 35: Im Freizeitbereich? Rein im Freizeitbereich? #00:42:38-3#

Interviewerin: Mhm (bejahend), zum Beispiel. Oder wie eben bei euch, der eine der&der die nur für den, für berufliche Zwecke. Was sind denn da so Situationen aus&aus der Erfahrung, wofür die Leute die am meisten nutzen? Wo die am meisten fehlt, die Prothese? #00:42:52-5#

Teilnehmer 34: Also ich hab‘ mal zum Beispiel ‘ne&‘ne&‘ne Unterarmprothese für ’n Gabelstaplerfahrer gebaut. Einfach mal ‘n Knauf rangeb-&rangebaut an den&an&an den Schaft äh damit er wieder mal&wieder ins äh Berufsleben zurückkommt. (Interviewerin: Mhm (bejahend)) #00:43:07-5#

Teilnehmer 30: Hängt sehr stark davon ab was die Leute so machen. #00:43:12-2#

Teilnehmer 35: Ja ich glaube da äh da gibt’s nichts, was man jetzt nicht sagen könnte. Weil für den einen ist (Teilnehmer 30: Ja.) und wenn er mir sagt: „Ich will mit dem Ding abends nur Fernbedienung für den Fernseher bedienen.“ (Interviewerin: Mhm (bejahend)). Äh gut, wenn er das so sieht, dann ‘ne? Dann ist das sein Wunsch. (Interviewerin: Ja.) #00:43:25-2#

Teilnehmer 32: Wenn’s zum Karten spielen, zum Karten halten halt auch, ‘ne? #00:43:27-6#

Teilnehmer 35: Nur ‘n Karten spielen, halten, genau deshalb (lacht), wo du das grade sagst. #00:43:33-0#

Teilnehmer 32: Ja (lacht). #00:43:31-8#

Teilnehmer 30: Einarmig Karten spielen ist bisschen doof, ‘ne? #00:43:35-4#

Teilnehmer 35: Ja, der ist der einarmige Bandit. Ähm- #00:43:37-9#

Teilnehmer 31: Ja gut aber ich sag mal Fahrzeuglenker, das heißt also Fahrrad oder Motorradlenker hört man schon relativ häufig (Teilnehmer 32: Ja.) (Teilnehmer 35: Ja.) da wo man definitiv den zweiten Arm braucht. #00:43:44-7#

Teilnehmer 33: Genau. Und das Faszinierende ist eben auch für die Freizeitaktivität, da wird die Prothese dann auch gewünscht und genutzt und wenn die kaputt ist, ist es viel schlimmer als (wenn?) meine Arbeitsprothese (alle lachen) kaputt ist. Also das sind auch, da stehen die (...?) (Teilnehmer 30: Kann man sich krankschreiben.) da nimmt man dann auch viel in Kauf. (Interviewerin: Mhm (bejahend)) Ich kann mich an einen Bogenschützen erinnern, mit ‘ner Oberarmprothese, das war schon ‘ne ganz schöne Herausforderung ähm diesen Kipppunkt und alles beim Bogen schießen zu finden (Interviewerin: Mhm (bejahend)) und da sind die Leute dann oft mehr bereit zu tun als die Prothese die so im Alltag integriert wird, wo wir sagen: „Ja du brauchst die doch bestimmt zum Essen und zum Getränk einschütten.“ und die Dysmelie klemmt sich die Brauseflasche unter ’n Arm und dreht sie auf (Interviewerin: Mhm (bejahend)) und nimmt sie dann um und schüttet ein. Das werden die nie mit der Prothese machen. Ich weiß aber nie ob das unser Anspruch ist, (Teilnehmer 35: Nee.) als Techniker zu sagen: „Dafür bauen wir Prothesen.“. (Teilnehmer 35: Nee.) Also in dem Moment wo wir ‘ne Lebensqualität verbessern, (Interviewerin: Mhm (bejahend)) ist das eigentlich völlig egal, für was die Prothese ist. Wir denken ja oft so unsere alltäglichen Dinge. Zähne putzen, waschen, essen, ich glaub das steht gar nicht unbedingt im Fokus (Interviewerin: Mhm (bejahend)) bei unsern Patienten, sondern die wollen die Dinge wo sie Interesse dran haben umsetzen und- #00:45:01-9#

Teilnehmer 32: Das andre wollen halt die Kassen gerne sehen. #00:45:03-0#

Teilnehmer 33: Genau. #00:45:03-6#

Teilnehmer 35: (…??) (Teilnehmer 34: Die überprüfbaren Kriterien.) Wäsche aufhängen, ja genau. #00:45:07-5#

Teilnehmer 33: Cola-Kiste schleppen und so. Das ist, ja. #00:45:12-1#

Teilnehmer 31: Ja gut bei Dysmelien ist sicherlich auch oftmals das Umfeld was sagt: „Wir möchten da ‘ne optische Normalität.“ Das heißt also Eltern, oder Großeltern (Teilnehmer 32: Genau.) (Teilnehmer 35: Auch.) oder so, die dann da ’n bisschen Druck aufbauen und sagen: „Ja das muss doch aber auch nach außen hin normal aussehen.“. #00:45:26-0#

Teilnehmer 33: Da kommt ja dann oft in der Pubertät der Knick, wo die die Prothese in die Ecke schmeißen und sagen: „Jetzt entscheide ich was ich will.“ oder umgekehrt die Damen sagen in der Pubertät: „Jetzt möcht (Teilnehmer 35: Jetzt will ich. Genau.) ich ‘ne Prothese tragen.“ Also das gibt’s natürlich auch. #00:45:39-1#

Teilnehmer 35: Ich hab’s aber auch schon gehabt, grade mit den Eltern der Druck die Normalität und vor allen Dingen auch: Da entscheidet das Kind ja halt nicht rational (Interviewerin: Mhm (bejahend)), das können wir nicht erwarten, das wär‘ ja abnormal, wenn ein Kind rational entscheiden würde. Also hat das Elternteil ja eigentlich nur die Möglichkeit: Ich baue jetzt diesen Druck auf (Interviewerin: Mhm (bejahend)), weil wenn ich jetzt sage: „Nö, ich lass meinem Kind freien Lauf.“ äh ich sag jetzt mal einfach stumpf, äh zwei Jahre, Ärztefehler irgendwas, so. 16, jetzt kommt der Punkt und jetzt legen wir mal los. Da äh (lacht), das geht jetzt aber nicht so einfach. (Interviewerin: Mhm (bejahend)) Ich glaube da ist es schon besser, wenn in diesen Fällen das Umfeld erstmal für die Normalität sorgt, damit die Normalität quasi auch wieder für denjenigen normal wird. Wenn er dann mit 18 sagt: „Ich ha-&das ist nicht meins.“ Wird ihm das niemals jemand übel nehmen zu sagen: „Jetzt ist Ende der Übung hier.“. Aber das muss er dann wirklich so entscheiden. Und dann entscheidet er das, weil er das&das andre auch kennt. (Interviewerin: Mhm (bejahend)) Aber das laufen zu lassen und am Ende nachher vor den Druck gestellt zu werden: Jetzt will ich aber und jetzt müssen wir erstmal ‘n riesen&‘n riesen Kasten aufmachen, damit das überhaupt erstmal möglich ist wieder den Startschuss zu geben, das ist glaub ich das Schlimmere. (Interviewerin: Mhm (bejahend)) #00:46:58-0#

Interviewerin: Und jetzt aber nochmal ‘n anderer Punkt und zwar wenn wir und jetzt mal vorstellen dieser Patient der kam und irgend ‘ne Versorgung haben wollte, die bekommen hat und diese Prothese wird regelmäßig getragen. Was kommt denn an Feedback von diesen Patienten, was an der Prothese gut ist und was an der schlecht ist? Oder was für Reparatur- oder Änderungswünsche kommen dann häufig von Patienten? Was haben Sie da für Erfahrungen gemacht? #00:47:23-7#

Teilnehmer 35: Also je besser das technisch umzusetzen ist, hat man in bestimmten Bereichen in der Damenwelt, dann kommt irgendwann die Kosmetik (lacht). Die wird so (...?) hochgetrieben, dass es ähm teilweise nicht mehr umzusetzen ist. (Interviewerin: Mhm (bejahend)) Das kann schon mal vorkommen. #00:47:38-3#

Interviewerin: Mhm (bejahend), was gibt’s da (Teilnehmer 35: Aber-) zum Beispiel für Forderungen an kosmetischen Sachen? #00:47:43-5#

Teilnehmer 35: Naja, dass die Hülle ich sag jetzt mal mehr oder weniger so, so schön wie möglich an die andere Seite angepasst wird. (Interviewerin: Mhm (bejahend)) Das muss nicht für den 24-stündigen Gebrauch sein, das ist dann meist auch wieder so für speziellere Sachen aber wie gesagt, das kommt dann schon mal. Wenn so das, die Technik komplett erschlossen ist (Interviewerin: Mhm (bejahend)) und es funktioniert alles, dann gibt’s auch nichts mehr zu optimieren, dann kommen solche Dinge schon mal in den Vordergrund. (Interviewerin: Mhm (bejahend)) #00:48:07-9#

Teilnehmer 31: Bei den Damen ist immer, doch eigentlich was ich dann höre, der Übergang Schaft zum Arm. Weil das ist so die erste Auffälligkeit, die man dann auch mal durch ‘ne dünne Bluse sieht, oder- (Teilnehmer 35: (..??)) #00:48:18-4#

Interviewerin: An auch&an kosmetischen Dingen, die verändert werden sollen? #00:48:20-0#

Teilnehmer 31: Ja ganz genau. Was kann man dran machen, damit das nicht sofort auffällt. (Interviewerin: Mhm (bejahend)) ‘Ne? Weil sonst, ich sag mal ‘n äh ‘nem Unterarm Form geben, ist nicht das Schwierigste auf der Welt. Aber eben tatsächlich diesen Absatz (Interviewerin: Mhm (bejahend)) und, dass der auch bei der Bluse, die dann locker drüber hängt, vielleicht auch bisschen transparent ist nicht zu sehen ist (Interviewerin: Mhm (bejahend)), das ist dann schon mal ‘ne Aufgabe, die wir gestellt kriegen. (Interviewerin: Mhm (bejahend)) #00:48:43-7#

Interviewerin: Und aber sonst, an zum Beispiel dann Reparaturen oder eben solche Sachen, die vielleicht am ehesten ‘n Problem machen, wo drüber gemeckert wird, dass irgendwas nicht so funktioniert wie’s soll, was wird da rückgemeldet? #00:48:57-1#

Teilnehmer 35: Ja ich sag mal, eher die Defekte bei mechanischen Überbeanspruchungen (lacht) (bejahendes Raunen). #00:49:00-5#

Teilnehmer 32: Wenn das zu oft passiert, ‘ne? #00:48:59-5#

Teilnehmer 35: Ja. #00:49:02-2#

Interviewerin: Mhm (bejahend). Und was sind das zum Beispiel für Sachen? Was sind so häufige Defekte? #00:49:06-4#

Teilnehmer 35: Bei den neueren Händen sind’s ganz klar Ausfall von den Einzelfingern, motorischen, einzelnen motorischen Stellen. #00:49:11-4#

Interviewerin: Bei den wo die einzelnen Finger beweglich sind? #00:49:12-2#

Teilnehmer 35: Ganz genau, dass da eben irgendwelche Sachen auftreten. #00:49:14-7#

Interviewerin: Mhm (bejahend) #00:49:15-6#

Teilnehmer 35: Und äh. Aber dann, wenn man das mal näher hinterleuchtet, waren das meist auch mechanische Überbelastungen. (Interviewerin: Mhm (bejahend)) Also entweder weil jemand mit ’m Fahrrad über Kopf gegangen ist, Kinder oder auch mal ‘n Erwachsender, aber ansonsten versuchen die dann später auch schon mal Dinge mit den Händen zu machen, für die sie einfach nicht ausgelegt sind. #00:49:32-2#

Interviewerin: Und für was ist so ‘ne Hand zum Beispiel nicht ausgelegt? #00:49:34-2#

Teilnehmer 35: Ich sag mal, man kann mit der Hand schon mal den Nagel halten, mit dem man in&in, ‘ne, den man in die Wand bringen will. Aber unbedingt den Motek (Teilnehmer 30: (...??) dann mitunter fünf Mal daneben zu hauen (lacht) und dann ‘ne, dann äh streikt die Technik dann schon mal. Aber dies kommt natürlich vor und dann sagt man: Also, ‘ne, jetzt brauchen wir mal ‘ne? Also jetzt machst das, später machst das dann aber andersrum (Interviewerin: Mhm (bejahend)). #00:50:01-6#

Teilnehmer 32: Das ist auch das, wenn wir, wenn wir Leute erkundigen, wenn wir die

beraten und die halt ‘ne super moderne Hand wollen (Interviewerin: Mhm (bejahend)), aber man hinterfragt was sie beruflich machen, wo ich dann auch sage: „Das funktioniert so nicht halt, ‘ne?“ #00:50:11-3#

Interviewerin: Ja, und aber bei welchen Sachen funktioniert ’s eben nicht? Also bei welchen beruflichen Tätigkeiten sagt man: „Dafür lieber was anderes.“? #00:50:19-2#

Teilnehmer 32: Ich sag mal so ‘ne I-limb-Hand zum Beispiel als Holzhacker (Interviewerin: Mhm (bejahend)) äh würd‘ ich ‘se auf gar keinen Fall empfehlen oder, ja. (Interviewerin: Ok, mhm (bejahend)) Dringend abraten. #00:50:29-0#

Teilnehmer 35: Naja gut, dafür haben wir ja die Arbeits-äh-geräte, ‘ne? Die man also da mechanisch höher belasten kann, ‘ne? #00:50:35-8#

Interviewerin: Ja genau, was würde man dann zum Beispiel für ’n Holzhacker für ‘ne Hand nehmen? #00:50:41-0#

Teilnehmer 35: Ja ich weiß nicht ob das äh ob das geht, das war vielleicht ‘n Extrembeispiel, aber ich sag mal, der Arbeitsgreifer hält mechanisch äh&äh von (Name eines Prothesenherstellers aus Datenschutzgründen ausgelassen), der hält schon was auf (bejahendes Raunen). (Interviewerin: Mhm (bejahend)) Also da war ich schon erstaunt bei bestimmten Sachen, die mir so (lacht) erzählt worden sind, dass der überhaupt noch lebend, also technisch lebend vor mir liegt (lacht) und nicht zerlegt. #00:51:00-9#

Teilnehmer 32: Mhm (bejahend) #00:51:04-4#

Teilnehmer 33: Ich glaub alles was uns wehtut und was wir mit der Hand nicht mehr greifen können, das können wir auch keiner Prothesenhand zumuten. (Interviewerin: Mhm (bejahend)) #00:51:10-1#

Teilnehmer 34: Das ist gut gesagt, ja. #00:51:11-0#

Teilnehmer 35: Ja. #00:51:11-8#

Teilnehmer 33: Und&und ähm wir&wir halten in ‘ner flachen Hand auch nicht unbedingt zwei Kisten Cola auf ‘n Mal ähm und wenn ich dann über zwei Griffe greifen will und will das mit ‘ner Prothesenhand machen, dann funktioniert das einfach nicht. (Interviewerin: Mhm (bejahend)) Und wenn wir uns ‘n Nagel nehmen und in die Wand hauen, hauen uns in den Finger, tut uns das auch weh. (Interviewerin: Mhm (bejahend)) Und das tut der Prothesenhand auch weh und ich glaub schon, dass auch nageln und diese Dinge und für ’n&für ’n Fräser oder ‘n Dreher der Arbeitshook schon in bestimmten Bereichen auch in der Arbeitswelt einzusetzen ist. (Teilnehmer 35: Genau.) (Interviewerin: Mhm (bejahend)) Und nicht ‘ne vielgliedrige, multifunktionale Hand (Teilnehmer 35: Nee.). Und nicht- #00:51:48-8#

Teilnehmer 35: Die ist halt zu filigran gebaut. Also das ist&das ist auch ‘ne Überbeanspruchung. #00:51:53-9#

Interviewerin: Aber aber ‘ne, würden Sie sagen, dass die überbeansprucht, oder dann eben fehlbeansprucht und benutzt wird oder würden Sie sagen, dass da die Technik nicht stark genug ist, um halt der Belastung der sie ausgesetzt sein soll äh zu ähm ja, zu widerstehen? #00:52:07-2#

Teilnehmer 35: Mh ja, so pauschal glaub ich kann man das- (Teilnehmer 32: Nee, kann man nicht so.) Nee, so pauschal kann man das nicht sagen. #00:52:12-9#

Teilnehmer 32: Also was ich den Leuten schon mitgebe (Interviewerin: Mhm (bejahend)) ist, dass halt diese hoch modernen Hände schon mehr Pflege oder mehr Wartung brauchen (Interviewerin: Mhm (bejahend)) als wie ‘ne altgediente- #00:52:28-0#

Teilnehmer 35: Dynamic Hand jetzt oder sowas, ‘ne? #00:52:29-9#

Teilnehmer 32: Genau. #00:52:29-6#

Teilnehmer 30: Na die Einflüsse von außen, die die&die Hände kaputt machen. #00:52:32-1#

Teilnehmer 35: Ja. #00:52:30-2#

Teilnehmer 30: Nicht, ni-&also- #00:52:32-2#

Teilnehmer 31: Sind aber auch oftmals dann Gewohnheiten, die sich einschleichen oder ist halt teil-&was ich auch beobachtet habe ist Anzieh(...?). Man erklärt stundenlang da vorne die Finger, das ist verbiegbar (Teilnehmer 35: Keine Stütze.) aber dann wird, sagen wir mal, nachdem eben insgesamt der Körper etwas größer geworden ist, ist natürlich auch etwas schwieriger in den Schaft reinzukommen. Das wird was weiß ich, (einfach?) beim Anziehen mal richtig schön (auf die Finger gestützt?) (Teilnehmer 35: (...???) nochmal hinterhergedrückt.) und mit Körpergewicht gearbeitet, was auf Dauer auch nicht gut ist. (Interviewerin: Mhm (bejahend)) Also das heißt, also es schleifen sich auch manchmal eben auch aus Unkenntnis, wir haben gar nicht oder ich hab damit gar nicht, von daher ja auch nicht erklärt, welche Gefahren da beim Anziehen allein entstehen können, (Interviewerin: Mhm (bejahend)) bis ich’s dann mal gesehen hab, wie die Hand angezogen wurde. Und mir war sofort klar, (Teilnehmer 35: Ok.) äh was da passiert war. #00:53:23-1#

Interviewerin: Mhm (bejahend). Und Herr (Name von Teilnehmer 34 aus Datenschutzgründen ausgelassen) Sie haben noch gesagt, dass es auch Umwelteinflüsse sind, die das&die die Prothese beschädigen? #00:53:34-0#

Teilnehmer 34: Na, damit mein ich halt Hammer, Fahrrad fahren, (Interviewerin: Ok, ja.) vom Fahrrad stürzen, (Teilnehmer 30: Feuchtigkeit.) ähm halt die&die Umwelteinflüsse meint ich damit. (Interviewerin: Mhm (bejahend), ja.) #00:53:40-6#

Teilnehmer 35: Aber bei Kindern zum Beispiel, die jetzt so, ‘ne, sind ja immer dabei ihre Grenzen weiter aufzuweichen (Interviewerin: Mhm (bejahend)) und wenn das Fahrradfahren auf normalem Wege halt gut klappt, dann wird schon mal links und rechts weggeschossen und in ’s Gelände und dann sind die Belastungen für so ’ne Daumen-Finger-Gruppe wenn’s auf ’m Lenker da drin rumwühlt natürlich auch was

Besonderes. Dabei hat’s auch schon so die kleineren Zerstörungen gegeben. #00:54:06-8#

Interviewerin: Mhm (bejahend). #00:54:09-4#

Teilnehmer 30: Oder einfach nur Loch im Kosmetikhandschuh und ‘n bisschen in strömenden Regen (Teilnehmer 35: Oder&oder das, wenn ja, zum Beispiel, ‘ne?) gekommen, ‘ne, auf ’m Fahrrad und schon rostet die I-limb-Hand. Ja, das ist halt auch mal schnell, also wenn da ‘n bisschen Feuchtigkeit reinkommt oder so. Mh. (Interviewerin: Ja.) #00:54:23-5#

Teilnehmer 35: Oder in ‘ne Pfütze gefallen, ‘ne? #00:54:26-9#

Teilnehmer 30: Ähm ja, oder irgendsowa-&also. #00:54:25-9#

Teilnehmer 35: Oder an der Nordsee, ‘ne? Ist gestolpert oder Schwester hat ‘n geschupst und wumm, war der drin gelegen. #00:54:31-2#

Teilnehmer 30: Dann hat der Nebenmann ihm ’s Bier drüber geschüttet (Teilnehmer 35: Hat&hat die Schwester in dem Moment nicht dran gedacht, ‘ne? In Kopf ge(...?)) oder irgendwas, also das ist ja ähm schnell passiert. #00:54:39-8#

Interviewerin: Und was gibt’s zum Beispiel an Rückmeldungen, weiß nicht, bekommt man so Rückmeldungen? Dass irgendwas seit die Prothese da ist besonders gut funktioniert? Dass es, dass die geschätzt wird, dass die irgendwas besonders erleichtert? #00:54:48-4#

Teilnehmer 35: Jo, das kriegen wir schon. #00:54:53-6#

Interviewerin: Was denn zum Beispiel? #00:54:55-3#

Teilnehmer 35: Ja das ist im, das ist glaub ich allgemeingültig, ob das jetzt, da&da gib-&da sch-&da ist glaub ich nichts irgendwas, was man in&in Platz eins, zwei, drei oder vier aufteilen kann. (Interviewerin: Mhm (bejahend). Nee aber nichtmal-) Also Alltags-, dass man sie halt immer mehr im Alltag gebraucht, dass wie gesagt, äh was Sie gesagt haben ähm im Freizeitbereich halt irgendwann so der Schritt gekommen ist, dass noch mehr möglich ist und die Lebensqualität da äh doch deutlich angehoben wird. (Interviewerin: Mhm (bejahend)) Ich glaube da ist die ganze Bandbreite da, ‘ne? #00:55:24-3#

Teilnehmer 32: Geht über ’n Haushalt, über ’s Kochen, dass man das alleine bewältigen kann halt, ‘ne? Das-. Genau. (Teilnehmer 35: Ja. Ganz&ganz genau, ‘ne? Einfach den Topf mal wieder ganz normal zum Tisch zu tragen, ohne dass man den Sohn, den Mann, die Tochter bitten muss, halt den schweren Römertopf da rüberzubringen, wie auch immer, oder Gusstopf. Ähm das sind so Kleinigkeiten, die dann aber doch Freude machen können in bestimmten Situationen. (Interviewerin: Mhm (bejahend)) #00:55:43-6#

Interviewerin: Und gibt’s außer diesen kosmetischen äh Wünschen, die Sie gesagt haben, hat da noch jemand irgendwas anderes, wo eben so Patienten kommen und sagen: „Ginge ’s nicht irgendwo, dass man hier noch irgendwas dreht oder macht,

dass man da irgendwas verändert, gibt’s da irgendwas?“. #00:55:57-6#

Teilnehmer 35: Also, wenn, dann waren’s bisher immer Funktionserweiterungen. Aber das ist dann Freizeitbereich, ‘ne? Wenn ‘se dann öfter kaputt geht äh bei bestimmten Sachen, dann wird gesagt: „Können wir’s nicht anders machen?“. Kann man da nicht irgendwie ‘n Adapter dran machen und den da so eben mal, ‘ne? #00:56:12-6#

Interviewerin: Bei welchen Freizeitgestaltungen zum Beispiel? #00:56:11-6#

Teilnehmer 35: Sagen wir mal, ja, Mountainbike, äh Downhill, Motocross. #00:56:22-4#

Interviewerin: Und was soll die Prothese dann können? Also was soll dann an ihr verändert werden? #00:56:23-6#

Teilnehmer 35: Nicht kaputt gehen (lacht). Ganz einfach. (Interviewerin: Mhm (bejahend), ok.) Der einfachste Wunsch: Die soll halten. #00:56:29-0#

Interviewerin: Mhm (bejahend). Fällt da noch jemandem was anderes ein? #00:56:33-1#

Teilnehmer 33: Ich glaub ein Aspekt ist immer wieder ’s Handgelenk. Wobei die Technik da schon auf ‘nem ganz guten Weg ist gegenüber den letzten zehn Jahren vielleicht (Interviewerin: Mhm (bejahend)) aber ich glaube schon, dass das Handgelenk, unser natürliches Handgelenk, immer das Problem war bei Prothesen, dass wir nicht flexibel genug waren und äh dass wir eben verschiedene Bewegungen halt mit der Technik nicht hinkriegen, weil wir nicht vierachsig sind, (Interviewerin: Mhm (bejahend)) oder man hat die Probleme, dass man in die Rotation nicht reinkommt, ‘ne, Pronation. Weil die Stümpfe zu lang sind, das sind so die Probleme wo wir dann an Grenzen stoßen auch von den Bau(...?) ‘ne? Aber diese Dynamik, was eben angeklungen ist, was so federnd und dynamisch ist, das fehlt uns oft. (Interviewerin: Mhm (bejahend)) Im Alltag aber. Das ist natürlich auch an die Technik ‘ne Herausforderung sowas auf ’n Weg zu bringen und so stabil auf ’n Weg zu bringen. Ich meine unser Handgelenk tut beim Downhill fahren dann irgendwann nach ’n paar Kilometern auch weh und wir sagen: „Ich glaub ’s reicht erstmal.“. ‘Ne Prothese sagt nicht: „Es reicht.“, die geht dann irgendwann kaputt. Und eigentlich ist es auch ‘n gutes Feedback, wenn unsere Kunden wiederkommen mit kaputten Prothesen, dann wissen wir wenigstens sie tragen sie und setzen sie ein. #00:57:54-2#

Teilnehmer 31: Das ist wohl wahr. #00:57:55-4#

Interviewerin: Gibt’s da noch irgendwas, was jemand hinzufügen möchte, zu so Feedback, das vom Patienten kommt? #00:58:03-7#

Teilnehmer 35: Naja, manchmal ist das natürlich auch so das Defizit dann, ‘ne? Dass man also, ich sag mal im Vordergrund die Sachen doch schon ganz gut erschlagen hat, wie’s so läuft und was so, was so gewünscht ist erstmal, in der Basis und nachher im&im wirklichen Leben im Haushalt dann doch nicht hingehauen hat, wo man dann halt gucken muss woran liegt es? Ähm muss ich einfach die Hand ‘n

bisschen von der Grundstatik, von ‘nem, von ‘nem Grundwinkel her ändern, damit bestimmte Sachen dann halt leichter fallen oder möglich sind, die vielleicht jetzt so dann doch nicht äh möglich waren. Aber das sind letztendlich, ja ich weiß nicht, das sind meist keine großen technischen Sachen wo man jetzt sagen muss: „Ist nicht möglich.“. (Interviewerin: Mhm (bejahend)) Das ist zwar jetzt nicht innerhalb von ‘ner halben Stunde zu ändern, weil man meist die Schäfte trennen muss, irgendwie, ‘ne? Den Winkel oder äh woanders hinschieben die Hand, damit man halt mit ‘nem bestimmten Ellbogenwinkel die Hand woanders halt noch äh&äh dann hat. Das ist nichts, was jetzt nicht umsetzbar ist. (Interviewerin: Mhm (bejahend)) Ist halt handwerkliche Arbeit da dran, ‘ne? Also, kein&kein Schwierigkeitspotenzial jetzt so gesehen. #00:59:06-5#

Teilnehmer 32: Ich sag mal, für mich als Techniker würd‘ ich mir wünschen, dass die Akkus generell, dass die kleiner und halt große Kapazität halt haben, dann. (Interviewerin: Mhm (bejahend)) #00:59:18-8#

Interviewerin: Kleiner, damit man’s eben besser unterbringen kann? #00:59:19-9#

Teilnehmer 32: Genau, richtig. Grade bei- #00:59:23-2#

Teilnehmer 35: Höher. Am besten in den Millimeter hier oben zwischen Schaft und der Außenhülle (Interviewerin: Mhm (bejahend)). #00:59:29-4#

Teilnehmer 31: Ein&ein Anspruch oder ähm ein Wunsch, den man natürlich immer schon mal wieder äh anheim getragen kriegt ist: „Warum geht das nicht so, wie ich’s im Internet sehe?“. (Interviewerin: Mhm (bejahend)) Also die Vergleichbarkeit äh ist denk ich, oder die Selbsteinschätzung oftmals noch ‘n Grund an dem oder ‘n Punkt an dem gearbeitet werden muss. Das heißt also es wird oft mal ‘n Wunsch einen äh vergleichbaren aber in der Forschung befindlichen oder im Grunde genommen in der Produktionsreife erst in vielleicht fünf oder zehn Jahren möglichen Versorgungen, wird dann versucht sich zu orientieren. (Interviewerin: Mhm (bejahend)) So nach dem Motto: „Wenn der Robotik locker kann und flüssige Bewegungen mit zwei Armen hinkriegt, warum kann ich das mit diesem blöden Scheißding nicht und muss da ewig umschalten?“, ‘ne? (Interviewerin: Mhm (bejahend)) Äh und ist in der Übungsphase, das holt den einen oder anderen runter, der da sagt: „Das muss doch anders, ich seh ’s doch da.“, ‘ne? „Guck dir den Film an, sowas will ich haben.“ (Interviewerin: Mhm (bejahend)) #01:00:32-1#

Teilnehmer 35: Also ich würde mir auch manchmal wünschen, dass äh Dinge die präsentiert werden, allgemein zugänglich im Internet, dass die mehr mit Informationen in den Rahmenbedingungen auch gefüttert werden, damit jemand das auch sehen kann, dass es das zwar gibt, im Vergleich mit den Informationen auf das, was er über sich selber weiß, aber ganz genau feststellt: Das wird jetzt nicht das sein, was mich übermorgen weiterbringt. (Interviewerin: Mhm (bejahend)) #01:00:58-8#

Interviewerin: Mhm (bejahend), also um einfach so ‘ne reelle Erwartungshaltung zu schaffen? #01:01:04-7#

Teilnehmer 35: Ja, nicht nur Zukunftssachen, ich sag jetzt mal ‘ne direkt nervenangebundene Sachen, warum gibt’s das jetzt noch nicht? Ja, weil’s halt

(Sachen?) hier noch nicht gibt. (Interviewerin: Mhm (bejahend)) Und aber es gibt ja auch Sachen, die gibt es schon. (Interviewerin: Mhm (bejahend)) Und es werden Sachen dargestellt und der sagt. „Wieso? Der läuft doch auch? Der läuft doch auch die 400 Meter Hürden.“, ‘ne? „Warum schaff ich das nicht?“. Ja, da wird aber schon, ich sag mal, Äpfel mit Birnen dann schon wieder verglichen, weil derjenige natürlich, wenn er das sieht, sagt: „Ok, das muss ich auch können.“. (Interviewerin: Mhm (bejahend)) Und wenn dabei immer noch so Rahmenbedingungen und grundsätzliche Informationen dazu gesagt werden würden, ähm dass das jetzt nicht das Normale, dass normal ist, was jedem zugänglich ist, weil die muskulären Voraussetzungen und&und&und&und&und. (Interviewerin: Mhm (bejahend)) Ähm das wär‘ manchmal ganz schön (Interviewerin: Ok, ja.), ‘ne? Die sehen dann den Sportler, der ‘n Autounfall hatte, der hat nach sechs Wochen nach äh, ‘ne, nach Amputation läuft der natürlich wieder (Interviewerin: Mhm (bejahend)). Jo, das was wir aber dann haben eben nicht, noch nicht so, ‘ne? (Interviewerin: Mhm (bejahend)) Und das gleiche haben wir in der Armwelt auch gehabt, ‘ne? Mit Sachen wie die Klavier spielende Hand, die die Japaner gemacht haben, ‘ne? Die ja wirklich Klavier gespielt hat, das war ja nun kein Fake und dann wird gesagt: „Ich w-&ich würd ganz gerne, also ich hab früher auch Klavier gespielt, kann ich das jetzt auch?“. Ich sag: „Nein. Jedenfalls nicht so.“. #01:02:28-6#

Interviewerin: Ok, dann würd‘ ich sagen, dass wir jetzt kurz äh ‘ne Pause einlegen, weil wir jetzt schon ganz gut in der Zeit vorangeschritten sind. #01:02:38-5#

(kurze Pause) #01:16:01-3#

Interviewerin: Ich unterbreche jetzt mal und äh fang einfach mit dem nächsten Punkt an, damit alle die pünktlich gehen müssen aufbrechen dürfen. Und zwar würde ich jetzt gerne, nachdem wir diese ganzen positiven und negativen Aspekte und so ’n bisschen Erfahrungen besprochen haben, mal auf was bisschen abstrakteres eingehen. Und zwar, wenn Sie jetzt die Möglichkeit hätten eben Dinge einfach zu verändern, weil Sie gesagt haben oft ist man dann so eingeschränkt durch die Vorgaben die da sind, durch das was an Material zur Verfügung steht, was würden Sie denn ähm an den Prothesen die grade auf ’m Markt sind gerne verbessern, verändern? #01:16:27-5#

Teilnehmer 35: Oh! (lacht) (kurze Pause) Ja, was würden wir ändern?

Teilnehmer 33: Also ein Punkt war der schon, der angesprochen ist, dass die Hersteller sich einbilden, dass auf meine Hand nur ‘ne Garantie gewährleistet wird, wenn ich auch den dementsprechenden (Teilnehmer 35: Ja.) Akku nehme, die Elektrode nehme und ich finde, damit können wir unsere Patienten nicht adäquat versorgen, ich würd dann noch gegebenenfalls nur Einzelteile zur Garantie und zu Reparaturen einschicken und würd für meinen Patienten, wenn ich das verantworten kann, in dem Bereich doch die Hand nehmen und den Akku nehmen und vielleicht die Elektrode, die ich selbe positionieren kann und äh das sind Dinge, wo ich einfach denke, da denkt sicherlich die Industrie jeder an sich, (Interviewerin: Mhm (bejahend)) ich glaub aber für unser Patientenklientel ist das nicht gut und ich würde mir wünschen, dass in dem Bereich der Markt nicht nur geöffnet wird wie zum Beispiel beim DynamicArm, wo ich sehe: Ja, den DynamicArm den baut kein anderer, aber da möcht ich schon, dass jeder irgendwie die Össur-Hand dran kriegt oder gut, Bebionic haben ‘se jetzt gekauft, das ist ‘n anderes Thema, aber da öffne

ich dann den Markt (Interviewerin: Mhm (bejahend)), weil ich sehe: Oh, mit der Komponente kann ich woanders noch Geld verdienen, aber ansonsten lege ich eigentlich dem Handel oder dem Zwischenhandel zum Patienten Steine in den Weg, wo ich sage das möcht ich nicht. Ich möcht nur meine Passteile verkaufen und wir sind grad im Kinderbereich dann wirklich oft gehandicapt, weil wir so kleine Komponenten nicht haben. Dann haben wir nur die fetten großen Akkus für die 2000er Hand oder so, wo ich dann einfach sage: „Gut, dann mach ich das einfach.". Ich meine das kann ich, ich bin sowieso Hersteller der Prothese und ich kann das verantworten (Interviewerin: Mhm (bejahend)), dann nehm‘ ich eben ‘n andren Akku, der vielleicht 1400 Milliampere hat und nicht nur 700 und kann dem Kind dafür aber garantieren, das kann den ganzen Tag spielen mit der Prothese und kann sie handhaben und da wir alle im gleichen Bereich liegen, was Spannung und Werte angeht von der Elektronik glaub ich, äh ist es nur ‘ne Hürde der Hersteller, weil jeder seine Produkte verkaufen will und das&das find ich ist ‘n riesen Handicap. (Interviewerin: Mhm (bejahend)) #01:18:43-0#

Interviewerin: Aber wenn wir da nicht so auf dieses eben Organisatorische ähm eingehen, sondern auf das Technische, dann sagen Sie zum Beispiel der Akku ist ‘ne Sache, die&die verändert werden sollte, wo man zum Beispiel lieber ‘n anderen benutzt. Was gibt’s noch für so technische Sachen, die man einfach an der Prothese verändern würde wenn’s möglich wäre? #01:19:06-1#

Teilnehmer 35: Ja die Grundhandform zum Beispiel bei den einzelmotorisch gesteuerten Fingerhänden. (Interviewerin: Mhm (bejahend)) Ähm das ist dann, ich mein die sind schon recht&recht gut in der Gestaltung, aber man kommt da schon an seine Grenzen, finde ich. #01:19:20-9#

Interviewerin: Und was dann genau zum Beispiel? #01:19:23-8#

Teilnehmer 35: Ja das sind dann, äh wir sind halt zu individuell mit Handbreiten, Fingerlängen und diese Sachen und es gibt halt, ich sag jetzt mal ganz äh stumpf, vier Größen inklusive Kinderhand. #01:19:34-3#

Interviewerin: Mhm (bejahend), also dass die Handgröße, ja- #01:19:35-7#

Teilnehmer 35: So. Und da hab‘ ich dann die Möglichkeit die ganz Große für Herren mit riesen Händen fällt dann schon weg, die Kinderhand fällt uns weg (Interviewerin: Mhm (bejahend)) und dann hab‘ ich in Wahrheit aus vier, hab ich eigentlich zwei (lacht) Größen meinetwegen, die ich dann verwenden kann, um das äh, um den Patienten das Beste zu geben. Und das sind dann zwei Größen nicht unbedingt immer so das Beste. (Interviewerin: Mhm (bejahend)) ‘Ne? Es gibt halt schon Übergangszeiten, wo man dann sagt: „Ok, da ist die Hand jetzt ‘n bisschen groß, hm aber, du wächst ja noch.". (Interviewerin: Mhm (bejahend), also da einfach-) Anders kann man das ja in dem Moment nicht regeln, ‘ne? (Interviewerin: Ja.) Wie will man’s machen, wenn man ganz gerne die schmalere Hand mit den längeren Fingern oder die breitere Hand mit den kürzeren Fingern je nachdem (Interviewerin: Mhm (bejahend)) und es gibt es nicht, dann muss ich das halt irgendwie anders regeln. #01:20:17-4#

Interviewerin: Mhm (bejahend), ok dann haben wir den Akku, eben die Handgröße angepasst an den Menschen. Gibt’s noch irgendwas? #01:20:23-1#

Teilnehmer 34: Ich weiß nicht ob’s geht, aber die Geräuschintensität der Motoren, ‘ne? Ich war gestern auf ’m Touch Bionics Lehrgang „sitt-sitt sitt-sitt“, das geht durch ’n ganzen Raum und jeder Anwender wird angeguckt. (Interviewerin: Mhm (bejahend)) Weil das Geräusch kommt aus der Ecke und da guck ich hin. (Interviewerin: Ja, mhm (bejahend)) Und manche stört das. (Interviewerin: Ja.) #01:20:43-9#

Interviewerin: Wir können gerne einfach nicht mal gucken äh funktioniert das, gibt’s dazu grade die Möglichkeit, sondern einfach wenn wir ganz&ganz abstrakt da einfach denken: Was ist was, was stört, was blöd läuft, was verändert werden muss. Mal ohne zu gucken: Wie kann ich das umsetzen? Sondern einfach, dass wir mal sammeln: Was sind Sachen, die verändert werden sollten? #01:21:04-4#

Teilnehmer 32: Optik, Funktion, Haltbarkeit. Das zusammen. (Alle: Mhm (bejahend)) #01:21:08-4#

Interviewerin: Mhm (bejahend) und in der Optik? Was in der Optik? #01:21:10-3#

Teilnehmer 32: Dass sie noch natürlicher wirkt. #01:21:11-0#

Interviewerin: Ok, mhm (bejahend). Und Funktion und Haltbarkeit? #01:21:14-0#

Teilnehmer 32: Jo, dass es einfach länger hält, ‘ne? #01:21:17-8#

Teilnehmer 30: Noch&noch mehr Funktionen und noch mehr Stabilität, (Teilnehmer 32: Genau.) also, dass es halt der Hand zwar vielleicht wehtut, aber sie nicht gleich kaputtgeht. Also&oder, dass halt das, was uns wehtut die Hand nicht gleich dazu äh (Teilnehmer 35: Zerstört.) bewegt den Dienst aufzugeben. #01:21:32-1#

Interviewerin: Also heißt das, aber heißt das, dass es eben länger aushält, einfach über ‘ne längere Zeit, oder dass es&dass es mehr Belastung erträgt? #01:21:42-0#

Teilnehmer 30: Sowohl als auch. #01:21:43-1#

Teilnehmer 35: Sowohl als auch glaub ich. (Teilnehmer 30: Und aber, also ich mein-) Mal in der Spitze mehr Last und (...?). #01:21:49-5#

Teilnehmer 33: Ich glaub- #01:21:51-3#

Teilnehmer 30: Ja, ab und zu wollen wir ja auch noch was reparieren, aber. (lacht) Ist jetzt nur Spaß. #01:21:53-6#

Teilnehmer 33: Ja aber ich glaub im Moment ist so die Mechanik und&und die Optik, also die Kosmetikhandschuhe bei den multiartikulierenden Händen halten einfach zu kurz (Teilnehmer 35: Ja.) (Interviewerin: Mhm (bejahend)) durch die extreme Mechanik verschleißen die in den Schwimmhäuten dermaßen schnell, dass man gar nicht hinterherkommt und wenn man überlegt, ‘n natürlich angepasster Handschuh liegt bei 4000 Euro (Interviewerin: Mhm (bejahend)) und ist nach 14 Tagen dann durch, ähm das kann einmal nicht sein und ähm da wünscht man sich sicherlich ‘ne Veränderung. Weil sowas fällt auch nicht mehr unter Garantie, wie man’s sonst

gewohnt war. #01:22:33-4#

Teilnehmer 34: Das muss ja nicht mal der teure Handschuh sein, es reicht ja schon der Michelangelohandschuh, der nach ich sag mal vier Monaten kaputt ist. (Teilnehmer 33: Ja.) Und der kost auch mal eben 900 Euro soweit ich weiß. Und das ist schon, dafür&für ’n Kosmetikhandschuh find ich das viel Geld. #01:22:48-9#

Teilnehmer 32: Und der hält noch am längsten von den ganzen Handschuhen. #01:22:52-3#

Teilnehmer 30: Wollt grad sagen, vier Monate ist ja echt gut (lacht). Also (Name von Patient aus Datenschutzgründen ausgelassen) arbeitet die in drei Wochen auf. #01:22:58-7#

Teilnehmer 34: Es kommt ja auch immer drauf an wie du sie nutzt, das war jetzt erstmal nur ‘ne Zahl in Raum geschmissen, aber ich find alleine für vier Monate 900 Euro, (Teilnehmer 30: Ja, auf jeden Fall.) find ich den Preis nicht gerechtfertigt (Teilnehmer 30: Ja, also das ist auf jeden Fall: Haltbarkeit von Kosmetikhandschuhen ist ‘n ganz großes Ding bei äh diesen Händen.) für so ’n bisschen Silikon oder PVC (…??). #01:23:17-8#

Teilnehmer 35: Wobei ich aber glaube, dass äh genau dieser Punkt weiter in der, in die Zukunft gedacht, wie gesagt, noch extremer aufweichen wird. Ich glaube, dass wir, wenn wir heute uns unterhalten, ob da vielleicht irgendwie die äh ich sag jetzt mal wie bei anderen kosmetischen Sachen, Silikonfuß und so, wie man denn, dass man das alles so natürlich wie möglich an den andern Fuß anpasst, ich glaube, dass wir in diesem Sektor da in, ja, da bin ich schon lang in Rente, aber ich sag jetzt mal in 15 oder 20 Jahren, äh glaub ich, werden wir über den Punkt gar nicht mehr so massiv diskutieren. #01:23:54-2#

Teilnehmer 30: Das glaub ich jetzt nicht. Und ich meint es ist ja auch unabhängig davon ob die, der Kosmetikhandschuh jetzt super realistisch ist, oder der ganz normale, stinknormale durchsichtige, äh die halten beide nicht länger. Das ist äh, einfach weil, das ist ja auch was, was die Komponenten untendrunter schützt (Interviewerin: Mhm (bejahend)), was ich vorhin gesagt hab mit äh&äh:: Kosmetikhandschuh, Loch drin gehabt, durch ’n Regen gefahren mit ’m Fahrrad, 8000 Euro Reparatur. (Interviewerin: Mhm (bejahend)) #01:24:19-8#

Teilnehmer 35: Aber da ist es dann der mechanische Schutz, aber nicht mehr die reine, ich sag mal, kosmetische Hülle als wirkliche Kosmetik, ‘ne? (Interviewerin: Mhm (bejahend)) Und äh, ich glaube da sind andere Sachen, wenn dann die Hand äh mit dem normalen Silikonüberzug halt ‘n Loch hat und der fällt ins Wasser, dass sie danach halt funktioniert ist glaub ich ‘n Mehrwert, sag ich jetzt mal, wie die Härchen, die auf die Kosmetik aufgeklebt sind. (Interviewerin: Mhm (bejahend)) ‘Ne? Ich glaub das kann man vernachlässigen. #01:24:42-5#

Interviewerin: Und an der Funktion? Was müsste an der Funktion verändert werden? Von der Prothese? #01:24:48-6#

Teilnehmer 35: Ich glaube das die Rückmeldung über die Hand, was da passiert, für den Patienten die jetzt nicht so da ist (Interviewerin: Mhm (bejahend)). Es sind zwar

Rückmeldungen da, die verhindern, dass man weiche Gegenstände nicht sonst wohin komprimiert und zerstört ähm aber die Rückmeldung für den Träger und Anwender. #01:25:08-4#

Interviewerin: Mhm (bejahend) auf welche (Teilnehmer 35: Über bestimmte Sachen.) Art und Weise? #01:25:11-5#

Teilnehmer 35: Ich sag jetzt mal ganz einfach, wenn der, ich sag mal wie dieses äh dieses 4D Kino, (Teilnehmer 30: (Vibrationsstrumpf?)) ‘ne? Wenn irgendwas ist, dass man’s riecht oder dass äh Wasser versprüht wird. Wenn, wenn’s in Filmen regnet, dass man das ähm realistisch mitkriegt. Ich glaube, dass solche Sachen irgendwo nicht ganz unwichtig sind. (Interviewerin: Mhm (bejahend)) Grade wenn die Funktion, die Technik, immer weiter geht und immer nutzbarer wird. In&auch in Bereiche rein, was denn ja auch manchmal vielleicht auch mit Gefährdung und sonst was damit zu tun haben kann, dann ist das vielleicht ganz gut, wenn ‘ne sensorische Rückmeldung, ich sag Mal Hitze, mechanische Belastung über Vibration (Interviewerin: Mhm (bejahend)) oder irgendwie was, dass er spürt, dass nicht die Hand aufhört zu drücken, sondern er, ich sag jetzt mal ganz doof, wenn da ‘n Bimetall, oder wenn da irgendwas ist, was ‘n bisschen warm wird und der merkt: „Mh!“; ‘ne? „Ist jetzt doch zu viel.“. Ähm, dass das vielleicht für bestimmte Sachen dann recht sinnvoll wäre. #01:26:03-9#

Interviewerin: Und für was müsste ’s so ‘ne Rückmeldung geben? #01:26:08-3#

Teilnehmer 35: Ich würde sagen so Kälte, Hitze und mechanische Überbelastung so als Grundkriterium (Interviewerin: Mhm (bejahend)). Mechanische Überbelastung, ich sag mal mit ‘nem Vibration hier, ‘ne? Wie&wie&wie ’s Handy (Interviewerin: Mhm (bejahend)) und das andere halt wirklich über die Hautoberfläche durch ‘ne Kontaktplatte Kälte und Wärme. (Interviewerin: Mhm (bejahend)) Ähm könnt ich mir vorstellen, ist ‘ne spannende Geschichte. #01:26:30-3#

Teilnehmer 32: Gibt es ja bei Zahnbürsten, wenn man zu stark drückt, (Interviewerin: Ja.) dann wird auch, dann vibriert das auch. (Teilnehmer 35: Ganz genau.) #01:26:40-6#

Teilnehmer 35: Und zeigt ja auch an, wenn’s zu lange war (lacht). #01:26:41-1#

Teilnehmer 32: Ja (lacht). #01:26:41-2#

Interviewerin: Ok und dann&dann haben wir die ähm- #01:26:43-2#

Teilnehmer 33: (…??) die Griffkraft ja. #01:26:42-7#

Interviewerin: Mhm (bejahend), genau nee, da würd‘ ich gern nochmal (Teilnehmer 31: (...??)) genauer drauf eingehen. Was sind da die wichtigen Punkte? Ist das einerseits eben die Rückmeldung eben die Griffkraft, oder&oder ähm weiß nicht die Stellung der Hand oder ist auch wichtig was tasten zu können, irgend ‘ne haptische Rückmeldung zu bekommen, was ist da Ihre Einschätzung oder Ihre Erfahrung vielleicht auch? Was melden da Patienten zurück? Was ist wichtiger oder ist beides wichtig? #01:27:09-3#

Teilnehmer 33: Also ich glaub es ist beides wichtig. (Interviewerin: Mhm (bejahend)) Damit, also so muss ich alles visuell ja doch hinterfragen, ob’s die Handstellung ist (Interviewerin: Ja.) oder die Griffkraft ist und ich glaub schon, dass beides wichtig ist. (Interviewerin: Mhm (bejahend)) Das fängt ja schon an äh bei Einarmern geht das immer noch, aber bei Doppelamputierten das fängt bei der Begrüßung an, bei Gestik an, das fängt an, wenn ich meinen nächsten Mitmenschen in irgend ‘ner Art und Weise berühre (Interviewerin: Mhm (bejahend)) oder ja. (Interviewerin: Mhm (bejahend)) #01:27:47-1#

Teilnehmer 35: Ich glaube, dass Rückmeldung über&über die Oberflächenbeschaffenheit (Interviewerin: Ja.) äh was ich greife natürlich auch nicht so uninteressant sind. Wenn ich was Scharfkantiges, extrem raues greife oder was ganz Glattes (Interviewerin: Mhm (bejahend)) ähm wie gesagt, was Sie schon sagten im Moment läuft das visuell, ich muss halt gucken. Wenn ich da rumgreife, bin ich weit genug oder schieb das weg, weil’s äh eben halt irgendwer bisschen Fett am&an dem Becher hatte oder weiß der Geier was (Interviewerin: Mhm (bejahend)) ähm und es dann nachher wirklich in der Umsetzung nicht so läuft. Und wenn sowas kommen würde, in welcher Form wei-&könnt ich jetzt nicht sagen, aber das wär‘ mit Sicherheit nicht das dööfste. #01:28:24-2#

Interviewerin: Mhm (bejahend). Und hat jemand anders ‘ne Idee noch wie man sowas technisch lösen könnte? Also wie sowas aussehen müsste, damit ’s am besten funktioniert? #01:28:33-3#

Teilnehmer 35: Schwierig. #01:28:38-4#

Teilnehmer 33: Also ich denke die Handschuhe werden einfach Leiterbahnen kriegen. Flexible Bahnen (Interviewerin: Mhm (bejahend)). Aber ich denke es geht auch nur über ‘ne vernünftige Basis. Ich weiß nicht ob’s nur, aber ich denke in der Technik, in der festen Hand wird’s schwierig sein (Interviewerin: Mhm (bejahend)) aber ich könnte mir schon vorstellen, dass über ’n&über ’n Handschuh bis zum Schaft hochgezogen, dass schon möglich ist sowas umzusetzen. (Interviewerin: Mhm (bejahend)) #01:29:06-2#

Interviewerin: Und gibt’s noch was anderes an Funktionen, die verbessert oder verändert werden müssten, wo noch, wo weiß nicht, wo vielleicht manchmal, vorher hatten wir glaub ich das Handgelenk oder noch irgendwelche anderen Sachen, die rückgemeldet werden, äh wo einfach ‘ne Funktion fehlt oder weiß nicht, sowas in die Richtung? #01:29:22-3#

Teilnehmer 33: Also die Dynamik zu erhöhen, zwischen den Stufen, (Interviewerin: Mhm (bejahend)) also das man kein umschalten, sondern dass man über ‘ne gewisse Mustererkennung dann halt fließende Bewegungen in die Hand kriegt. #01:29:33-2#

Teilnehmer 31: Eigentlich koordinierte Programme die ablaufen, dass (was?) quasi in bestimmten Grifffunktionen auch die Handdrehung mit drin ist, weil sie gelernt hat, dass hier jetzt grade nach ’m Glas gegriffen wird. #01:29:47-6#

Teilnehmer 33: Genau. Das ‘ne Hand eben angelernt wird, wie&wie unsere Kindern auch lernen, wenn du- #01:29:55-7#

Teilnehmer 31: Wundert mich, dass es in dem Sektor noch nicht weiter ist. In Spielzeug und allem möglichen Krempel sind wir da schon viel weiter, als ich jetzt neulich aus der Autowerkstatt kam, hat der mir auch ‘ne Rechnung dafür gestellt, dass er meine Abgasrückführung anlernen musste. Dacht ich: „Haja.“ (lacht). Äh aber ich sag mal die, äh die programmatische Steuerung unserer myoelektrischen Hände ist doch noch relativ simpel im Prinzip, sag ich mal. (...?)- #01:30:23-2#

Teilnehmer 30: (...??)-

Teilnehmer 35: Zu dem was heute technisch möglich wäre, wenn man das ausreizt, mit Sicherheit. Aber wie zum Beispiel mit dem Handgelenk, was es ja jetzt gibt, durch das Einrasten äh oder die&die Aufwärtsstellung falls ich ein&weil ich ja jetzt Tastatur, also PC-Arbeiten machen muss und dann mit den, mit den äh Fingermustern die ich anfahre, um das äh halt dann beidhändig irgendwie ‘n bisschen wieder zu machen. ‘N Handgelenk was ich dementsprechend einstellen würde bei bestimmten Sachen, was ich dann nicht mechanisch äh&äh hinstellen muss äh wär‘ mit Sicherheit- #01:30:54-6#

Interviewerin: Mhm (bejahend), also ‘ne gewisse Automatik? #01:30:52-4#

Teilnehmer 35: ‘N gewisser Automatismus, der dann auch äh über äh Stepmotoren da angefahren wird, wär‘ mit Sicherheit nicht schlecht. Wobei wir dann wieder bei dem distalen Gewicht sind, ‘ne? (lacht) Und dem Akku! #01:31:03-6#

Teilnehmer (31 oder 32): Und vor allen Dingen bei der Energieversorgung, genau. #01:31:06-6#

Teilnehmer 32: Das sollen die Entwickler dann regeln. #01:31:08-0#

Teilnehmer 35: Ja, ja. Aber wie gesagt, ich sag mal die äh ich glaube, dass die Akkutechnik durch andere Geschichten, die, wo der Druck viel höher ist äh für uns sowieso irgendwann abfallen werden. #01:31:18-3#

Interviewerin: Mhm (bejahend). Ich würd‘ gerne hier, weil Sie gerade unterbrochen wurden. Sie haben gesagt die Reaktionsgeschwindigkeit? #01:31:23-2#

Teilnehmer 30: Ja, also ähm f-&die Geschwindigkeit von den Motoren und, dass man beim&beim Umschalten zwischen den Stufen, ja das ist halt, was die Leute noch sehr ausbremst glaub ich. #01:31:33-1#

Interviewerin: Also damit man einfach schneller (Teilnehmer 30: Grade, grade wenn man jetzt komplexere Versorgungen hat, mit, wo man verschiedene Komponenten ansteuern kann, oder so.) reagieren kann? Mhm (bejahend), ja. Ja. #01:31:42-4#

Teilnehmer 35: Wobei ich finde die, die Griffgeschwindigkeit, wenn jemand ‘n bestimmte Signalbandbreite hat, die er dynamisch sehr gut einsetzen kann, das heißt also die Einstiegsschwelle schön weit unten äh ist ähm und der macht ‘n verhältnismäßig großes Signal, die Geschwindigkeit in der so ‘ne Hand auf und zu geht find ich jetzt nicht so, dass man sagen könnte, das muss aber drastisch erhöht werden, damit das ‘n wahnsinns Mehrgewinn wird. #01:32:12-0#

Teilnehmer 33: Aber ich glaub das war nicht gemeint. Ich glaub eher, dass ich, wenn ich zugreifen will, dann in die Rotation, dass ich dann über ‘ne andre Schaltstelle weg muss (Teilnehmer 30: Genau.). (Teilnehmer 35: Ach so in der Umschaltung, bis sie dann wieder bereit ist. Ja ok.) Wie auch immer, um in den (...???) ‘ne? Und wenn ich&wenn ich dann noch äh ‘n Handgelenk brauche äh. #01:32:28-3#

Teilnehmer 35: Ja ok. #01:32:28-3#

Teilnehmer 30: Genau (lacht). #01:32:28-8#

Teilnehmer 35: Ja das stimmt allerdings. (Teilnehmer 30: Also die&des&des Problem mehrere Sachen gleichzeitig anzusteuern im Prinzip, wenn ich jetzt-. Genau.) Wenn ich (...?) umschalten und- #01:32:36-6#

Teilnehmer 33: Und wenn ich dann nicht über ‘ne Co-Kontraktion gehe und sag, ich muss jetzt ‘n drei Mal öffnen-Signal geben, um in die Rotation zu kommen, (Teilnehmer 35: Ja.) ich glaub dann sind wir bei Zeiten die (Teilnehmer 35: Jaja, das ist richtig.) äh nicht mehr der dynamischen Hand entsprechen. #01:32:50-1#

Teilnehmer 35: Nee&nee&nee. Das sind dann einzelne Bewegungen, die wirklich äh- #01:32:53-8#

Teilnehmer 30: Also das Zusammenspiel der verschiedenen Bewegungen, vielleicht so. #01:32:55-5#

Teilnehmer 35: Jaja. #01:32:54-2#

Interviewerin: Mhm (bejahend), ja. Und gibt’s Dinge die explizit nicht verändert werden sollten? Weil wir hatten in anderen Gesprächen immer wieder das, dass irgendwer mal meinte naja, es wird immer alles totentwickelt und man hat eigentlich schon Sachen die ganz gut sind und dann will man’s verbessern und eigentlich werden Sachen dann dadurch schlechter. Gibt’s irgendwas, was Ihnen einfällt wo Sie sagen würden das ist gut, genau so, wie’s ist, oder vielleicht wie’s mal war und da wurde was in ‘ne Richtung verändert, die man eigentlich, wo man eigentlich lieber die Zeit zurückdrehen würde? #01:33:20-3#

Teilnehmer 35: Nee, eigentlich nicht. #01:33:22-7#

Teilnehmer (5?): Zum Sauerbruch-Arm (alle lachen). #01:33:24-9#

Teilnehmer 35: Also ich glaube, dass immerhin ‘n Status jetzt so gesehen, das ist schon recht gut. Ähm wenn jetzt die nächsten Entwicklungsschritte kommen, die werden dann ja wahrscheinlich in ganz andere Richtungen gehen müssen (Interviewerin: Mhm (bejahend)) um sowas, ich sag mal, um da das Rad etwas neu zu erfinden oder äh wirklich ‘n wirklich anders darzustellen. Ich glaube, dass dadurch alleine schon neue Aspekte sich dann ergeben werden. Und das ist so ’n&so ’n Ding, was sich selber glaub ich dann immer rundformt. #01:33:56-7#

Teilnehmer 33: Mir fällt glaub ich ‘n Aspekt ein (Interviewerin: Mhm (bejahend)) wo’s sich wirklich lohnt zurück zu gucken und das sind die Preise (alle lachen). Dass ich

einfach glaube, wir sind schon in einem Level angekommen, bei den Preisen, natürlich haben wir in der oberen Extremität extrem wenig Stückzahlen (Interviewerin: Mhm (bejahend)). Aber wenn ich mir überlege, dass ich für ’n Unterarm jenseits der 70.000 Euro mit ’m Handgelenk mit ‘ner Quantum-Hand liege, mit ‘nem Liner und allem Drum und Dran, dann glaub ich, sind es schon Dimensionen (Interviewerin: Mhm (bejahend)) die’s den Kostenträgern und damit natürlich auch uns den Patienten zu versorgen einfach zu schwer machen. (Interviewerin: Mhm (bejahend)) Natürlich sind Preise gerechtfertigt, aber wie hoch (Teilnehmer 35: Ja, wobei ich sagen muss, für den Entwicklungsaufwand der im&im&in der Vergangenheit betrieben worden ist und der über 20 Jahre ja länger, kein Geld erwirtschaftet hat, nur gekostet hat äh die Zeit meine ich jetzt, wenn man jetzt den Myo-Beginn nimmt und äh das hat wirklich Gelder aufgefressen und hat nichts in dem Sektor wirklich so abgeworfen, dass man sagen konnte die&die Entwicklungskosten waren in normalen Zeiten, die man in der Wirtschaft anlegt wieder&wieder drin ähm sind bestimmte Preise glaub ich schon gerechtfertigt. Weil Ideen reintun muss in Deutschland bezahlt werden. Wir können nicht alles zu Konsumerpreisen kriegen und äh wenn ich mir überlege, dass äh in anderen Bereichen wo Entwicklungskosten auch da sind, aber wesentlich größere Stückzahlen da sind, die Idee zu den Produkten wesentlich stärker und über einen längeren Zeitraum bezahlt werden muss ich ganz ehrlich sagen, find ich da diese Schere jetzt nicht so extrem, das ich sagen müsste mh ‘n Unterarm müsste jetzt auf jeden Fall, ist zu hoch oder darf nicht mehr als 40.000 Euro kosten, wenn er jetzt bei 70 oder 62, je nachdem welche Ausführung. Es ist, da würd‘ ich sagen, wenn ich das Umrechnen würde so, wir sind jetzt älter, wir haben noch so ‘n bisschen auch die D-Mark im Kopf und die Kaufkraft, aber da würd ich sagen das finde ich jetzt nicht so, dass man sagen muss äh das ist am Explodieren. Also da bin ich bei anderen Bereichen, da bin ich in anderen Bereichen des Gesundheitssystems wesentlich kritischer, wo ich sage, da sollte man mal genauer hingucken, ob das wirklich so laufen muss. (Interviewerin: Mhm (bejahend)) Ähm es darf sich natürlich aber nicht, wenn das der Hintergrund war, mit diesem wahnsinns äh ich sag mal jetzt weiterentwickeln, dass kleinere Entwicklungsschritte nachher dazu führen, dass ‘n Arm doppelt so teuer ist. (…??).) #01:36:38-0#

Interviewerin: Mhm (bejahend). Aber wenn wir das Finanzielle jetzt einmal ganz kurz beiseitelassen als Punkt, weil da kann man wahrscheinlich ganz lange bei bleiben, würd ich gern nochmal wissen, ob jemandem noch irgendwas einfällt, was funktionell oder technisch verbessert werden müsste. #01:36:52-5#

Teilnehmer 32: Mir fällt noch eins ein (Interviewerin: Mhm (bejahend)) was mir des Öfteren Anwender schon gesagt haben und zwar simpel aber ähm der Ein- und Ausschalter bei der Hand. (Interviewerin: Mhm (bejahend)) Dass der halt leichter zu bedienen ist, wie das sonst bei der, glaub ich, Speed-Hand wo man’s halt durch ’n guten ähm Druckpunkt erkennt auch (Interviewerin: Mhm (bejahend)) man so jetzt immer die Kipp-&die&die Kippschalter halt äh halt durch ‘n (...?) fühlen muss, was schon fummelig ist. (Interviewerin: Mhm (bejahend)) Das haben w-&hab‘ ich schon oft gehört jetzt. #01:37:15-3#

Interviewerin: Also, dass es da (Teilnehmer 32: Dass das fummelig ist.) leichter anzuwenden, leichter zu finden ist? #01:37:17-7#

Teilnehmer 32: Genau, ja. #01:37:16-3#

Interviewerin: Mhm (bejahend). #01:37:19-9#

Teilnehmer 33: Mh, da frag ich mich natürlich, warum geht so ‘n Hightechgerät wie entwickelt wird, nicht nach, weiß nicht, fünf Minuten nichts tun in einen Stand By Modus, um Batterien und sonst was zu schonen (Interviewerin: Mhm (bejahend)) und wenn ich das Gerät wieder ansteuere lös ich ‘n Stand By Modus auf und (Interviewerin: Ja.) kann irgendwie da die Funktionen ausführen. (Interviewerin: Mhm (bejahend)) #01:37:38-0#

Teilnehmer 30: Na, da geht’s ja quasi auch beim An- und Ausziehen oder so, dass dann die Hand nicht irgendwie, irgendwelche Sachen macht oder so, aber keine Ahnung bei w- #01:37:49-2#

Teilnehmer 35: Also generell ganz aus sollte man sie schon schalten können. #01:37:48-8#

Teilnehmer 30: Oder irgendwie mal, was weiß ich, äh keine Ahnung, bei wenn man irgendwas jetzt gegriffen hat oder dann ausschalten, wenn man nicht will, dass es sich versehentlich auf-&dass es versehentlich aufgeht, also das war er die- #01:37:59-7#

Teilnehmer 33: Gut, klaren An- und (Ausstellplatz?) das ist richtig. #01:38:00-7# #01:38:00-6#

Teilnehmer 35: Also klarer An- und Ausschalter ist technisch nicht schlecht, aber ähm ich sag mal, daran hab ich jetzt noch gar nicht, mit diesem kl-&mit der kleinen Wippe da manchmal in bestimmten Positionen dann zu finden, das ist gar nicht so (Teilnehmer 32: Richtig.) so einfach wenn man am Schaft zum Beispiel irgendwo an ‘ner Stelle ‘n Sensor hätte, wo man einfach richtig draufdrücken muss, mit ‘ner gewissen Kraft, die so durch ‘ne Körperberührung normal nicht stattfindet (Interviewerin: Mhm (bejahend)) und das irgendwo ‘ne gewisse Fläche hat, wo ich gar nicht so gezielt draufdrücken muss, dann wird das super sein, ‘ne? #01:38:27-3#

Teilnehmer 32: Oder direkt auch an der Hand selber. Das sagten auch, wenn man direkt an der Hand was zupackt, ganz kurz äh (Teilnehmer 35: Oder so, ja, ja, oder so.) draufbleibt und äh, dass man nicht das ganze System ausschalten muss. (Interviewerin: Mhm (bejahend)) #01:38:41-2#

Teilnehmer 35: Jo, das stimmt. #01:38:40-6#

Interviewerin: Mhm (bejahend), gibt’s noch irgendeinen anderen Aspekt, der da noch wichtig ist? #01:38:44-8#

(kurze Pause) #01:38:49-6#

Interviewerin: Gut. Ja wir sind ja zeitlich auch ‘n bisschen gehetzt. Dann wären wir auch fast schon am Ende. Ähm ich würde jetzt jeden bitten, nochmal ganz kurz zu überlegen, ob’s zum Schluss noch irgendwas auch zu den vorherigen Fragen und Punkten, ob’s noch irgendwas gibt, was vielleicht ergänzt werden sollte oder ähm auch einfach ‘ne Sache, dass Sie nochmal für sich überlegen und einmal äußern,

was besonders wichtig ist bei den Aspekten, was Ihnen da besonders am Herzen liegt eben von diesen, wir haben geguckt was verbessert werden kann oder sollte, was nicht verbessert werden sollte, was gut läuft, schlecht läuft, welche Erfahrungen die Leute da mitbringen, was Ihnen da besonders ‘n Herzensanliegen ist. Wenn das vielleicht jeder noch einmal sagen könnte zum Abschluss. #01:39:29-5#

(kurze Pause) #01:39:33-8#

Interviewerin: Freiwillige vor! #01:39:34-2#

Teilnehmer 35: Ja, also, ja also ich muss ganz ehrlich sagen von den Sachen die wir heute besprochen haben, ist eigentlich so ’n Paket geschnürt, da kann man eigentlich jetzt nicht sagen, das kann man raustrennen, das ist jetzt nicht ganz so wichtig. Das ist glaub ich ‘n Paket, was sich so als wichtig auch darstellt. #01:39:49-8#

Interviewerin: Mhm (bejahend). Oder gibt’s vielleicht auch Sachen die jetzt von mir nicht gefragt wurden, die aber, wo Sie finden, dass die noch wichtig wären anzumerken? #01:39:58-7#

(kurze Pause) #01:39:59-9#

Teilnehmer 35: Nee, jetzt so Ad hoc. (lacht) #01:40:05-8#

Teilnehmer 34: Nee, Sie haben die Gesprächsrunde gut geführt. #01:40:07-0#

Interviewerin: Vielen Dank. Nee, aber ich mein es gibt ja oft einfach was, wo dann doch der&der Blick eben nicht hingeht, wenn da jemandem noch irgendwas einfällt, dann gerne, oder sonst, genau Ergänzungen zu dem was Sie vorher gesagt haben. #01:40:19-6#

Teilnehmer 30: Glaub ich, (Teilnehmer 35: Nee, das, (..??)) also für mich ziemlichen Rundumschlag gemacht haben ‘ne? Also-5 #01:40:26-8#

Interviewerin: Ja. #01:40:24-4#

Teilnehmer 33: Also ich glaub schon für unsere Anwender wär‘ so ‘n bisschen mehr Selbstlerneffekt der&der Komponenten. (Interviewerin: Mhm (bejahend)) Das wär‘ schon gut, um die Patienten mit auf den Weg zu nehmen, mit den Prothesen besser arbeiten zu können. Das verbunden mit der Dynamik die wir eben noch angesprochen haben (Interviewerin: Mhm (bejahend)). #01:40:45-3#

Interviewerin: Und ganz kurz: Selbstlerneffekt, heißt? #01:40:49-4#

Teilnehmer 33: Dass Komponenten über Bewegungen (Interviewerin: Mhm (bejahend), ja.) angelernt werden. So wie sich uns- #01:40:54-0#

Interviewerin: Ok, also dass die Prothese so ’ne Art Eigenintelligenz hat? #01:40:57-7#

Teilnehmer 33: Genau. #01:40:53-1#

Interviewerin: Mhm (bejahend), ok, ja. #01:40:55-0#

Teilnehmer 35: Sich auf den individuellen Nutzer ‘n bisschen einstellt. #01:41:00-6#

Teilnehmer 30: Dass sie sich mehr- (alle sprechen durcheinander) #01:41:00-9#

Interviewerin: Ganz kurz, einmal das wir hier nicht- #01:41:06-4#

Teilnehmer 31: In andern Arbeitsbereichen ist ‘ne Brille auf, die ‘n Videosinn haben noch als Ergänzung mitbringt. Warum müssen wir ewig nur auf zwei Kanälen rummachen? #01:41:14-9#

Interviewerin: Mhm (bejahend) (Alle: Mhm (bejahend)) #01:41:18-8#

Teilnehmer 35: Ich mein letztendlich kann man sich fragen, warum die Hand nicht äh selber visuell unterstützt (Interviewerin: Mhm (bejahend)) äh und wird. Ich sag mal, der Roboter früher mussten die Leute bei (Name eines Autoherstellers aus Datenschutzgründen ausgelassen) das Rad oder bei den anderen Marken, um keine Werbung zu machen, äh drehen, damit die Löcher mit den Rad-äh-bolzen oder mit den&mit den Achsbolzen übereinander steht (Interviewerin: Mhm (bejahend)), heute äh guckt der Roboter drauf (Interviewerin: Mhm (bejahend)), die müssen nicht mal ausgerichtet werden, die können wahllos stehen, zack erkennt dies, dreht den, setzt das Ding auf und die Muttern sind drauf (Interviewerin: Mhm (bejahend)). Äh Spycams sind heute so klein, dass ‘se kaum gesehen werden, obwohl so vor einem liegen, wieso soll ‘ne Hand nicht auf einmal gucken können? #01:41:54-4#

Teilnehmer 31: Ist ’n ganz wesentlicher Unterschied: Der Industrieroboter kennt seinen Standort und seine Lage im Raum (Interviewerin: Mhm (bejahend)). Durch irgendwelche Einflüsse von außen, sei es durch elektromagnetische Gitter, durch Streifen, was weiß ich. #01:42:05-0#

Teilnehmer 35: Ja, na klar. #01:42:08-5#

Teilnehmer 31: Das kennt die Hand nicht. Die bewegt sich frei im (...?) (Teilnehmer 35: Ja, gut, aber. Ja, es muss auch noch ‘n bisschen, muss auch noch ‘n bisschen Anreiz hier sein für die Zukunft (lacht)). Aber wie gesagt die visuelle Ergänzung (Teilnehmer 35: ‘Ne? Ja.) über ‘n optischen Parameter. (Interviewerin: Mhm (bejahend)) Ich meine, wenn man gestern die Sendung im Fernsehen weiterverfolgt hat, hat man ja gesehen, dass man aus zweidimensional ohne weiteres auch ganz schnell dreidimensional machen kann. (Interviewerin: Mhm (bejahend)) Ganz schnell. #01:42:29-4#

Teilnehmer (5?): In einer Fernsehsendung. (alle lachen) #01:42:34-1#

Teilnehmer 30: Das ist ganz einfach, ‘ne? #01:42:34-2#

Teilnehmer 35: Sehen ‘se? Da ham‘ wir wieder das mit den Medien: „Das ging das gestern auch.“ (lacht) #01:42:43-2#

Teilnehmer 31: Dass aus einem zweidimensionalen Röntgenbild mit, das wär‘ jetzt

verkehrt zu sagen mit wenig Aufwand, denn der Aufwand ist eben schon eines Preises würdig, eben tatsächlich schafft ‘ne dreidimensionale Darstellung hinzukriegen. (Interviewerin: Mhm (bejahend)) So. Das heißt also da fehlt irgendwo ‘n Sensor. (Interviewerin: Ja.) Der benötigt wird um diese Information in dieses System reinzubringen. Das ist denk ich der Punkt. Ähm wo ‘se sicherlich, und wenn man andere Entwicklungen so verfolgt, eben diese (...?) aus dem Internet, ist es denk ich da auch tatsächlich der wesentliche Ansatz eben in der&an den Sensoren zu arbeiten. Welche Möglichkeiten ich habe, ich muss nicht ewig nur auf zwei Punkten, auf zwei Kanälen äh rumhacken, wenn ich gesehen habe (...?), der Herr mit den beiden Armen aus mh äh Amerika, der hat was weiß ich alleine auf einer Schulterkappe hat der 25 oder 26 doppelpolige Sensoren drauf, ‘ne? (Interviewerin: Mhm (bejahend)) Das heißt also da steckt ‘n, ‘ne ganz andere Kommunikation zwischen Körper und Prothese. (Interviewerin: Mhm (bejahend)) Gut, ganz unten stand dann auch irgendwo 500.000 Dollar oder sowas, aber das ist ‘ne andere Sache, ‘ne? #01:43:52-7#

Teilnehmer 33: Genau. #01:43:54-9#

Teilnehmer 31: Und das kann keine Solidargemeinschaft leisten. ‘Ne? Wenn wir alle Oberarm-äh-amputierten im Augenblick so versorgen würden, das macht jede Solidargemeinschaft fertig. #01:44:06-8#

Interviewerin: Mhm (bejahend) #01:44:07-6#

Teilnehmer 35: Aber wenn das irgendwann in dementsprechenden, wenn das für jeden zugänglich ist und es wird nur noch das angeboten, dann würde die Technik ja oftmals auch günstiger werden. Aber dadurch das wir ja immer so an allem festhalten, wir müssen ja das alte noch anbieten und dann das ganz Neue und das was dazwischen war muss auch noch alles im Regal liegen und das ist natürlich wesentlich teurer als wenn ich sage wofür-. Ich sag mal jetzt ganz blöd, was mich immer ärgert äh harte, steife Stromkabel. Äh Was müssen die überhaupt noch produziert werden? Nur noch hochflexible, weiche Kabel anbieten in der Menge wie ‘se gebraucht werden, dann sind die Dinger billig oder günstig. Fertig aus. So kann man auch äh Effizienz schaffen. Aber wir wollen ja immer-. Die ganze Ba-. #01:44:53-2#

Teilnehmer 30: Aber schieb mal ‘n hochflexibles Kabel durch ‘n Leerrohr. #01:44:54-4#

Teilnehmer 35: Ja ich kann es ziehen. Wenn ich vorher was durchschiebe, dann muss ich halt den Kopf vorher benutzen und darf das, darf den Zugdraht nicht vergessen (lacht). #01:45:03-0#

Interviewerin: Ähm wenn sonst niemand jetzt hier zu unsrer Fragerunde irgendwas ergänzen möchte, würde ich sagen, dass das Gespräch dann erstmal beendet ist, das heißt wir machen die Mikrofone aus.
