# Supplementary material for: What do users and their aiding professionals want from future devices in upper limb prosthetics? A focus group study
Source: PLoS One. 2023 Dec 29;18(12):e0295516. doi: 10.1371/journal.pone.0295516 (PMC10756510; doi:10.1371/journal.pone.0295516)
Supplement: S1 Appendix — (ZIP) [file pone.0295516.s001.zip › FocusGroup_Transcripts/FGC3.pdf]

Interviewerin: Ab jetzt wird aufgezeichnet. Ähm wenn ich die erste Frage in die Runde stell gleich, würd‘ ich bitten, dass erstmal alle der Reihe nach antworten und sich ganz kurz vorstellen und danach kann wild und frei geantwortet werden. Aber dass wir halt einmal zu jedem, zu jeder Stimme nochmal den Namen haben. Genau. Ich würd‘ zum Einstieg einmal bitten, dass Sie sich vorstellen, dass Sie als Fachmann oder Fachfrau im Umgang mit Menschen die Armprothesen tragen eben auf ‘ne Person mit ‘ner Armprothese zum ersten Mal treffen und würde Sie bitten dass Sie erzählen was Sie&worauf Sie da als erstes achten. Also wenn Sie den Menschen zum ersten Mal sehen und sehen der hat ‘ne Armprothese, was Ihnen da als erstes auffällt, worauf Sie als erstes achten. #00:00:44-6#

Teilnehmerin 36: Erstmal mich vorstellen, ‘ne? #00:00:40-7#

Interviewerin: Mhm (bejahend), genau. #00:00:46-7#

Teilnehmerin 36: Genau. Ja, (Name von Teilnehmerin 36 aus Datenschutzgründen ausgelassen), Physiotherapeutin ähm und ich bin hier in dem Sanitätshaus oder in der Orthopädiewerkstatt seit ähm sieben Jahren. Genau. Wenn ich jetzt auf den Patienten treffe als erstes schau ich erstmal, ob der die Prothese in der Tasche hat oder wirklich umgelegt hat (Interviewerin: Mhm (bejahend)). Ähm das ist der erste Eindruck. Und das ist wirklich beides offen, oft kommen die mit der Prothese in der Tasche, also ja. (Interviewerin: Mhm (bejahend)) #00:01:17-9#

Interviewerin: Und wenn der die Prothese trägt? Fällt dann direkt irgendwas auf, wie der damit umgeht, oder wie der damit aussieht? #00:01:27-1#

Teilnehmerin 36: Ä::hm, dann ist es eigentlich so dieser suchende Blick, äh ist&ist er jetzt links oder rechts betroffen (Interviewerin: Mhm (bejahend)), weil ich eigentlich im Vorfeld auch immer vergesse war das jetzt links oder rechts (Interviewerin: Mhm (bejahend)). Ähm ich guck mir immer nur den Namen an, wenn ich Patienten aus dem Wartebereich abhole (Interviewerin: Ja.). Also so welche Seite, so ’n bisschen dieses kurz-&kurze scannen des Patienten. #00:01:45-1#

Interviewerin: Mhm (bejahend) und macht das aber dann ‘n Unterschied auf welcher Seite das ist, wie man dann erstmal begegnet oder wie man dann, weiß nicht, miteinander anfängt zu arbeiten? #00:01:55-2#

Teilnehmerin 36: Ja, zum Guten Tag sagen, (Interviewerin: Mhm (bejahend), ja.) ‘ne? Dass man also ähm ich&ich, das gibt man ja klassisch immer mit der rechten Hand (Interviewerin: Ja, klar.) so und wenn dann rechts die Prothese ist, dann würd‘ ich einfach so ‘n bisschen zurückhaltender sein, um nicht den Patienten gleich damit zu konfrontieren, dass er das vielleicht nicht mag, nicht kann, oder so. #00:02:16-5#

Interviewerin: Ja. #00:02:16-5#

Teilnehmerin 36: Ja. #00:02:15-9#

Interviewerin: Ok. Herr (Namen von Teilnehmer 37 aus Datenschutzgründen ausgelassen)? #00:02:18-1#

Teilnehmer 37: Ja. (stellt etwas an seinem Handy ein) Ja, (Name von Teilnehmer 37

aus Datenschutzgründen ausgelassen), auch Physiotherapeut hier im Sanitätshaus. Ich hab‘ relativ wenig mit Armprothesen zu tun, bin eher für die untere Extremität äh mit zuständig. Aber wo achtet man drauf, wenn ‘n Patient mit Prothese, Armprothese kommt? Ja, man guckt auch rechts oder links (Interviewerin: Mhm (bejahend)), klar. Und ähm dann würd‘ ich jetzt drauf achten wie sitzt der Schaft vielleicht erstmal (Interviewerin: Mhm (bejahend)) oder was für ‘n Befestigungssystem gibt es dann da und dann machen wir vielleicht so ein paar Eingangstests, Untersuchungen, guckt wie nutzt er vielleicht seine Armprothese mittlerweile schon, ‘ne? #00:03:08-6#

Interviewerin: Mhm (bejahend). Und was für Tests macht man dann oder worauf guckt man dann da ganz am Anfang? #00:03:14-9#

Teilnehmer 37: Gut, also was&was für Tests würd‘ man machen? Also ich würd‘ einfach mal äh normale Bewegungsmuster vielleicht gucken. Wenn er was&was greifen soll oder wie verhält es sich bei ganz normalen äh Bewegungen von der Schulter her (Interviewerin: Mhm (bejahend)). Ist ja auch die Frage ist es&auf welcher Höhe die Amputation ist (Interviewerin: Mhm (bejahend)), ‘ne ob äh Unterarm oder Oberarm amputiert, da würd dann drauf gucken. #00:03:41-5#

Interviewerin: Ok, Danke schön. #00:03:42-4#

Teilnehmerin 38: Äh ja, ich bin (Name von Teilnehmerin 38 aus Datenschutzgründen ausgelassen), ich komm aus äh (Name der Orts aus Datenschutzgründen ausgelassen), von (Name des Sanitätshauses aus Datenschutzgründen ausgelassen). Ich hab‘ bevor ich die Ausbildung zur Technikerin gemacht habe die Ausbildung als Ergotherapeutin gemacht (Interviewerin: Mhm (bejahend)) und bei uns ist es so, wenn Kunden oder Patienten, wie man’s auch nennen will (Interviewerin: Mhm (bejahend)) mit ‘ner Prothese zu uns kommen, ist es auf jeden Fall erstmal dieser suchende Blick welche Seite, um halt wegen der Begrüßung zu schauen, wie man sich da jetzt verhält. Ganz klar. Und dann in einem Gespräch auch, wie die Prothese in das Körperbild integriert wird. Also ob auch gesprochen wird mit, also mit Händen artikuliert wird (Interviewerin: Mhm (bejahend)) oder ob die Prothese nur am Körper getragen wird. Ja und also das ist so der erste Eindruck den man hat. (Interviewerin: Mhm (bejahend)). #00:04:28-0#

Teilnehmer 39: Ja, mein Name ist (Name von Teilnehmer 39 aus Datenschutzgründen ausgelassen), ich bin auch Physiotherapeut, selbständig seit 2001, habe ein (Name und Ort des Betriebs aus Datenschutzgründen ausgelassen) und ja, mein erster Eindruck bei den Patienten ist halt tatsächlich auch, das seh‘ ich genauso: Nutzt der die Prothese oder ist es ‘n Beiwerk, wie kommt der auf mich zu, hat der sie überhaupt um? (Interviewerin: Mhm (bejahend)) Weil die meisten ähm die sie nicht nutzen, haben sie tatsächlich eher in der Tasche, das heißt für mich ist dann schon wichtig ähm wenn der reinkommt ähm ja nutz der&ist es nur ‘ne Prothese oder ähm hat er sich mit der Prothese im besten Falle angefreundet und nutzt sie, ähm das kann schon beim an-, ausziehen sein. (Interviewerin: Mhm (bejahend)) Was macht der damit, kann er sie, wenn’s ‘ne Myoelektrische ist, wo wir jetzt ja von ausgehen, (Interviewerin: Ja.) kann er sie überhaupt ansteuern oder ja, hängt die da rum, trägt er sie vielleicht nicht, weil sie gar nicht passt (Interviewerin: Mhm (bejahend))? Ähm solche Sachen halt, das sind dann so die ersten Sachen. Und ich sprech‘ ihn halt auch in der Regel drauf an ähm ist das ‘n Neupatient (Interviewerin: Mhm (bejahend)) oder soll’s ‘n-? Ähm dass ich dann erstmal, frage:

Wie nutzt er sie überhaupt oder hängt er sie abends in seine Ladeeinheit und bringt sie mit zur Therapie und die ganze andere Zeit in seiner&mh Alltag nutzt er sie gar nicht. (Interviewerin: Mhm (bejahend)) Weil das sind Sachen, die nachher ähm wichtig sind und ähm selbst wenn er sie nutzt: Wie nutzt er sie? #00:05:42-9#

Interviewerin: Mhm (bejahend) und ähm also was ändert das dann an der späteren Herangehensweise? Wenn ich jetzt seh‘, der nutzt die total oder der nutzt die überhaupt nicht, wie geht’s danach weiter, wenn man das festgestellt hat für sich? #00:05:56-2#

Teilnehmer 39: Dann, nehmen wir mal an es ist jemand der kommt zu uns und ähm hat die Prothese vielleicht vorher nicht viel genutzt. (Interviewerin: Mhm (bejahend)) Dann stellt sich je die Frage warum hat er sie nicht genutzt, kann er sie nicht ansteuern, ist die Passform nicht richtig (Interviewerin: Mhm (bejahend)), ähm häufig ist es tatsächlich auch so, ist ja bei vielen Patienten die auch mit solchen Prothesen versorgt werden sollen, dass da einfach ähm die Grundvoraussetzung noch nicht stimmt (Interviewerin: Mhm (bejahend)). Heißt ähm er hat vielleicht gar nicht die Muskelaktivität an den Stellen, wo abgenommen wird, dass er sie überhaupt ansteuern kann. Und da würde man dann rangehen. Woran liegt das und dann würd‘ ich halt mit demjenigen erstmal erarbeiten, dass nachher dementsprechend die Prothese auch sauber angesteuert werden kann. Im Idealfall ist es ja so, der Patient ist von uns so vorbereitet worden (Interviewerin: Mhm (bejahend)), hat die Tests ähm die dann durchgeführt werden ähm ja, so gut machen können (Interviewerin: Mhm (bejahend)), dass man im Idealfall ‘ne Prothese nachher anpasst und er sie ähm tatsächlich schon steuern kann (Interviewerin: Mhm (bejahend), ja.). Alles andere wäre dann Feinarbeit. Aber das war die-&wär‘ der Idealfall. #00:06:57-4#

Interviewerin: Ja, ok, mhm (bejahend). (Name von Teilnehmerin 36 aus Datenschutzgründen ausgelassen), ich hab‘ gesehen du hast jetzt irgendwas parallel notiert. Gibt’s da irgendwas noch zu ergänzen zu diesem- #00:07:04-7#

Teilnehmerin 36: Ich hab‘ ehrlich gesagt mir nochmal eure Namen und die Sanitätshäuser (alle lachen). Weil ich so schlecht im Erinnern bin, dass ich sowas in mein Leben integrieren muss, (Interviewerin: Alles gut!) die kleinen (Gedächtnisstützen?). #00:07:15-7#

Interviewerin: Gibt’s noch irgendwas, was jemand (lacht)-. Nee, sorry, alles gut. Ich dachte vielleicht gibt’s noch irgendeinen Punkt, der da mit eingebracht werde sollte. #00:07:19-9#

Teilnehmerin 36: Ich hab’s jetzt auch wieder drauf (lacht). #00:07:29-5#

Interviewerin: Aber gibt’s sonst irgendwas, was einem noch auffällt eben, weiß nicht, erstmal ja was, trägt der die oder hat der die in der Tasche, auf welcher Seite ist das, aber gibt’s auch irgendwas wo man gleich von Anfang an sagen kann: Sitzt die gut, sitzt die nicht gut, funktioniert die gut oder nicht? Sie haben schon gesagt, dieses ähm benutzt der die beim Sprechen, ist das da mit integriert. Gibt’s da noch irgendwas, wo das einen Aufschluss darüber geben kann eben wie die Prothese benutzt wird und ob die überhaupt da im Gebrauch ist, wenn jemand die trägt? #00:07:55-6#

Teilnehmer 39: Ich denke, was eben noch gar nicht zur Sprache gekommen ist, man guckt unweigerlich auch: Wer kommt denn da überhaupt rein? Wie alt ist derjenige (Interviewerin: Ja.) ähm was hat der vielleicht vorher für ‘ne Versorgung gehabt? Ich sag jetzt mal wir haben immer noch auch ähm Herrschaften aus Kriegszeiten (lacht) sag ich jetzt mal. (Interviewerin: Mhm (bejahend)) Die haben ganz andere Erfahrungen mit Prothesen ähm geschweige denn, wenn man da ankommt und sagt so, man möchte jetzt ‘ne myoelektrische Prothese denen versorgen, dann haben die vielleicht mit den ersten, die’s da gab Erfahrungen (Interviewerin: Mhm (bejahend)) wo dann ‘ne Hand, weiß ich, (lacht) sehr sehr lang am Modus gearbeitet hat (Interviewerin: Mhm (bejahend)) und dann die da wieder ranzuführen ist natürlich was anderes wenn ich jetzt ‘n jüngeren Menschen habe, der vielleicht ‘n Verkehrsunfall oder was auch immer gehabt hat, weswegen er seinen Arm verloren hat. Dann ist das nochmal wieder ‘ne ganz andre Geschichte, der steht auch noch ganz anders im Leben (Interviewerin: Mhm (bejahend)) und ähm der kann vielleicht auch schneller lernen oder schneller Sachen umsetzen, als dann jemand der über Jahre vielleicht mit 'ner ganz einfachen Versorgung, oder Schmuckversorgung oder was auch immer versorgt wird (Interviewerin: Mhm (bejahend)). Also solche Sachen schaut man sich dann schon auch mit an, wenn der grad das erste Mal kommt (Interviewerin: Ja.). #00:09:05-0#

Teilnehmer 37: Ja, das stimmt. #00:09:03-1#

Interviewerin: Ok. Und ähm dann würde mich als nächstes interessieren, wenn jetzt die Menschen einfach in die Praxis kommen und einfach zur Behandlung kommen, was melden die denn rück an ihrer Erfahrung mit der Prothese? Also was für positive oder negative Rückmeldungen bekommt man von den Menschen die Prothesen tragen? (kurze Pause) Vielleicht können wir erstmal die Positiven sammeln und erstmal überlegen was da- (Gelächter unter den Teilnehmern). Oder kommt da nichts Positives? (lacht) #00:09:29-6#

Teilnehmer 39: Doch, doch. Also es gibt ähm mir kamen jetzt gleich zwei, drei Sachen in&in&ins Gedächtnis (Interviewerin: Mhm (bejahend)). Gehen wir mal davon aus, derjenige hat vorher keine Prothese gehabt (Interviewerin: Ja.). Wir gehen davon aus, dass alle im Team, heißt Orthopädietechnik, Arzt, Therapeut ihr Bestes gegeben haben (Interviewerin: Mhm (bejahend)), derjenige ist jetzt versorgt. Und die erste Reaktion ist dann häufig, ist eigentlich jetzt egal auf welcher Höhe wir sind: „Ich bin wieder vollständig.“. (Interviewerin: Mhm (bejahend)) Das heißt wieder ein&eine komplette Person, die da in den Spiegel schaut. (Interviewerin: Mhm (bejahend)) Und ich hab da wieder eine Hand, die ich auch noch bewegen kann (Interviewerin: Mhm (bejahend)). Ähm ganz extrem wird das dann in dem Bereich ähm Kinder, ich erinnere mich da grade ähm an diesen 15-jährigen, den hatt‘ ich damals auch als Beispiel da bei der Firma (Name eines Prothesenherstellers aus Datenschutzgründen ausgelassen) gebracht ähm der auf einmal vor seiner Mutter wieder, in dem Fall mit zwei Beinen und einem Arm stand, das war für sie erstmal, den kannte sie gar nicht mehr so. (Interviewerin: Mhm (bejahend)) Das sind glaub ich so die ersten Momente für denjenigen selbst, oder auch für die Verwandtschaft, Familie etc. (Interviewerin: Mhm (bejahend)). Das ist glaub ich so das&das Erste. (Interviewerin: Mhm (bejahend)) #00:10:39-9#

Teilnehmerin 36: Ich glaub die Unterscheidung ist aber wichtig, ob das ein traumatisch(er?) Verlust ist, (Teilnehmer 39: Genau.) oder ob’s ähm angeboren ist

und das sind grundsätzlich (Teilnehmer 39: Unterschiede.) unterschiedliche ähm&äh Erfahrungen (Interviewerin: Ja.) oder auch Verhaltensweisen und ähm nach traumatischem Verl-&Verlust (Interviewerin: Mhm (bejahend)) äh::m würd ich jetzt aber gleich anführen wollen diesen negativen Aspekt, dass Patienten häufig enttäuscht sind (Interviewerin: Mhm (bejahend)), dass die äh Prothesen schwer sind, dass sie nicht spüren können, dass die kalt sind ähm weil man das auf den Hochglanzvideos, in Anführungsstrichen, ähm der ähm (Interviewerin: Mhm (bejahend)) Herstellerfirmen überhaupt nicht wahrnehmen kann. #00:11:24-6#

Interviewerin: Ja. Und was ist aber noch anders an den Erfahrungen eben wenn&ob, also abhängig davon, ob's 'ne traumatische Folge ist oder ob jemand einfach so zur Welt gekommen ist. Was än-&also was verändert das an der Erfahrung die die Leute sonst mit den Prothesen machen? #00:11:43-7#

Teilnehmerin 38: Also ganz extrem ist halt so der Umgang mit der Prothese (Interviewerin: Mhm (bejahend)) weil bei ähm angeborenen Fehlbildungen ist es so, dass es, es wird als Hilfsmittel akzeptiert (Interviewerin: Mhm (bejahend)), weil die ohne Prothese sehr gut klarkommen (Interviewerin: Ja.), weil sie's so gewohnt sind. Die sehen sich ja nicht als krank oder eingeschränkt. (Interviewerin: Mhm (bejahend)) Sie <u>sind</u> einfach so. (Interviewerin: Mhm (bejahend)) Und jemand der durch 'n Trauma seine Prothese verloren hat, hat halt viel Hoffnung so in die Prothese und denkt so: „Ok, damit wird alles wieder funktionieren.“. (Interviewerin: Mhm (bejahend)) Also die Herangehensweise an die Prothesenversorgung ist 'ne ganz andere (Interviewerin: Ok, ja, mhm (bejahend).). #00:12:14-7#

Interviewerin: Und gibt's sonst noch irgendwas was irgendwie so rückgemeldet wird, was mit der Prothese danach besonders gut wieder klappt? Zum Beispiel welche Funktionen besonders, 'ne besondere Erleichterung im Alltag danach darstellen, wenn 'ne Prothese dann wieder angepasst ist? #00:12:28-2#

Teilnehmerin 38: So dieses beidhändige Arbeiten ist immer für viele sehr wichtig, oder generell Gegenstände fixieren zu können, behalten zu können, um mit der, ja erhaltene Hand ist irgendwie doof gesagt, aber mit der anderen Körperseite (Interviewerin: Mhm (bejahend)) die voll funktionsfähig ist meistens, (Interviewerin: Mhm (bejahend)), da einfach wieder 'n viel größeren Freiheitsgrad zu haben (Interviewerin: Mhm (bejahend)), weil man nicht alles mit der Seite machen kann, sondern die Prothese eben als Unterstützung benutzen kann. (Interviewerin: Ja, mhm (bejahend).) Also wenn's alleine nur 'ne Tasche halten und die Türklinke betätigen ist (Interviewerin: Mhm (bejahend)) oder die Tasche halten und jemandem die Hand reichen (Interviewerin: Mhm (bejahend)) kann, das ist für viele schon- #00:13:01-9#

Interviewerin: Also einfach irgendwas wo halt, wo's überhaupt 'ne zweite Seite gibt, (Teilnehmerin 38: Ja.) die halt irgendwas übernehmen kann? Mhm (bejahend) #00:13:10-1#

Teilnehmer 39: Ist aber 'n Prozess, 'n langer Lernprozess. Also es ist ja nicht so, dass jemand 'ne Prothese hat und sagt (Interviewerin: Mhm (bejahend)): „Jetzt ist es, hallo, alles toll und ich mach jetzt alles damit.“ (Interviewerin: Mhm (bejahend)) ähm bei den meisten ist es ja so, die müssen erstmal sehen ähm was kann ich damit machen, was will ich damit machen, wo macht es Sinn, für <u>mich</u>, für viele ist es dann

tatsächlich so, ähm eben auch wie bei angeborenen Geschichten, dass sie natürlich sich auch erstmal Kompensationsmechanismen angewöhnt haben und (Interviewerin: Mhm (bejahend)) die Hand reagiert dann halt eben nicht, wie die gesunde Hand, unabhängig davon, dass ähm da geb‘ ich der (Name von Teilnehmerin 36 aus Datenschutzgründen ausgelassen) recht, dass es ähm einfach ja, eben kein Arm ist der warm ist, der nicht fühlen kann, aber derjenige, d-&das ist dann so ’n Findungsprozess bei den Patienten häufig erstmal. Ähm das ist ja nicht so, dass sie auf einmal ihre Prothese für alles Mögliche nutzen, sondern angucken erstmal, was geht und beziehungsweise wofür nutz ich sie wirklich (Interviewerin: Ja.). Wo auch eben natürlich die Gefahr ist, wenn jemand sagt: „Mensch, ich nutz die nur für die und die Sachen und ansonsten leg ich sie wieder beiseite.“ (lacht) (Interviewerin: Mhm (bejahend)), dann sind wir wieder bei dem Thema er trägt sie nicht regelmäßig, dann ähm hat natürlich wieder Folgeerscheinungen, dass sie unter Umständen dann irgendwann nicht mehr so funktioniert wie vorher (Interviewerin: Mhm (bejahend)). Da muss man halt dranbleiben, das ist aber dann irgendwie auch Aufgabe des Therapeuten. #00:14:23-2#

Interviewerin: Ja. Und Sie haben vorher noch gesagt, es ist vor allem so dieses man, man fühlt sich wieder vollständig. Woran wird das denn festgemacht? Ist das einfach nur dass ich seh‘ halt im Spiegel, dass da, dass da auf der andren Seite irgendwas ist oder ist das auch irgendwas, was eben an Funktion übernommen wird, wo man sagen kann, weil das wieder funktioniert oder weil das was bewegt wird ähm gibt es dieses Gefühl, dass man wieder vollständig ist danach? #00:14:46-2#

Teilnehmer 39: Ich denke der erste Moment ist schon eher das Optische: Ich habe wieder ähm zwei Arme, zwei Beine. #00:14:54-0#

Interviewerin: Ja. #00:14:54-0#

Teilnehmer 39: Äh::m und das andere ist sicherlich dann ‘n weiterer Schritt, wo man sagt Mensch, ich hab die und die Funktion, wie sie eben auch schon sagt, dass ich vielleicht ‘ne Handtasche halten kann (oder?)&und ‘ne Tür-. Das sind dann aber wirklich kleine Schritte, man&es ist ja nicht so, ähm man zaubert ‘n Arm hin (Interviewerin: Mhm (bejahend)) und derjenige macht wieder alles. Da bin ich auch bei&bei (Name von Teilnehmerin 36 aus Datenschutzgründen ausgelassen) was diese Hochglanzvideos, (Interviewerin: Mhm (bejahend)) ich erinnere mich dran, als von (Name eines Prothesenherstellers aus Datenschutzgründen ausgelassen) er hinten in seine Hosentasche greift, bezahlt und so. Alles schön und gut (Interviewerin: Mhm (bejahend)) äh::m es gibt Leute, haben wir auch bei uns schon gehabt im Hause, die wirklich ‘ne Prothese anziehen und weder bei uns danach noch wiedergesehen werden halbes Jahr, noch beim Orthopädietechniker (Interviewerin: Mhm (bejahend)) und wenn man dann mal anruft: „Ach ja, ich sollte mal zur Kontrolle kommen.“. Und derjenige kam tatsächlich an und hat mit der Prothese alles Mögliche gemacht. Das ist der Idealfall, das hab‘ ich bis jetzt ein Mal vielleicht gesehen (Interviewerin: Mhm (bejahend)). Hatten wir grad vor kurzem einen, wo wir gesagt haben: „Das kann nicht sein.“, aber ist eher die Seltenheit. Bei den anderen ist es dann so, dass man sich eigentlich eher freut, (Interviewerin: Mhm (bejahend)) wenn sie viel benutzt worden ist, grade bei Kindern ist das ja sch-&toll zu sehen, wenn sie sie wirklich viel benutzen, dann geht auch was kaputt. Wenn nichts kaputt geht, wird ‘ne Prothese nicht benutzt. #00:16:00-5#

Interviewerin: Mhm (bejahend), ok. #00:15:58-6#

Teilnehmer 39: Das kann man eigentlich, weiß nicht, (Teilnehmerin 36: Mhm (bejahend)) ich sag das jetzt mal so in Raum rein. #00:16:03-0#

Teilnehmerin 38: Wenn der Handschuh sauber ist, dann ist irgendwas falsch. #00:16:03-6#

Teilnehmer 39: Genau, dann ist irgendwas falsch. Hat er nicht mal Zeitung gelesen über die (Maß?). (lacht) Ja. #00:16:09-8#

Interviewerin: Mhm (bejahend). Und ähm noch so an negativen Punkten, jetzt außerdem, dass&dass eben irgend ‘ne Erwartung nicht erfüllt wird oder dass man halt so ‘n bisschen die Hoffnung hat, das ersetzt so den Arm der fehlt. Gibt’s noch irgendwas wo man konkret bei Übungen oder so, wo halt so ‘ne Grenze erreicht wird und derjenige der die Prothese trägt halt einfach sagt: „Weiß nicht, die&mit der Funktion komm ich nicht klar.“ oder „Das funktioniert nicht so wie ich möchte, da ist einfach ‘n Punkt wo die nicht das macht, was ich mir vorgestellt hätte.“. Gibt’s da irgendwas, was Ihnen einfällt? #00:16:37-4#

Teilnehmerin 38: Jetzt bezogen wirklich nur auf die Greifkomponente ist es ja abhängig davon, welche Hand man draufhat, welche Griffmöglichkeiten sie hat (Interviewerin: Mhm (bejahend)). Dann gibt’s ja die einfach Myo(Name eines Prothesenherstellers aus Datenschutzgründen ausgelassen), die nur öffnen und schließen kann (Interviewerin: Mhm (bejahend)) oder bis zu ‘ner Vincent oder Bebionic-Hand (..???) Pinzettengriff oder der Greifer der&wo du wirklich ‘n Stecknadelkopf mit aufheben kannst. Also da kommt man ganz schnell, je nach Greifkomponente an seine Grenzen, was man damit machen kann und was nicht. (Interviewerin: Mhm (bejahend)) Und ich glaube, dass man da eben ansetzen muss. Es kommt drauf an, wie da diese Erwartungshaltung und wie kann man dem Anwender dann seine Möglichkeiten mit der Hand zeigen. Weil alles in einer Hand geht nicht, das fängt schon eben an mit diesen Greiffunktionen und endet dann mit der Schließkraft oder was kann ich mit der Hand heben. (Interviewerin: Mhm (bejahend)) Da hat man ja überall seine Grenzen. Das ist halt kein Ersatz der menschlichen Hand. (Interviewerin: Mhm (bejahend)) #00:17:30-4#

Teilnehmer 39: Das ist im Übrigen was wo ich nicht denke, dass die Technik das so weit ähm ja, nicht <u>übernehmen</u> kann, weil das ist noch was, die Technik kann nicht sehen, was der Therapeut sieht. Das heißt, ich muss natürlich adäquat meine Therapie, meine Übungen die ich auch mit dem Patienten mache, mit der Prothese dann anpassen, weil es bringt überhaupt nichts, ähm sie hat eben so schön gesagt „Pinzettengriff“, ‘n Pinzettengriff versuchen anzufangen und er schon gleich nach der ersten Behandlung, weil’s eben nicht funktioniert so desillu-&desillesuliert ähm dass er sagt: „Ich hab keine Lust mehr.“. Also ich muss natürlich dann mit dem Patienten mitgehen, muss sehen welche Fähigkeiten hat der. Ähm muss natürlich aber ihn auch fordern, gar keine Frage, aber das ist dann eben die Kunst ähm in der Therapie die richtigen Maßnahmen zu ergreifen und ihn dann da weiterzuführen. #00:18:25-5#

Interviewerin: Mhm (bejahend) und&also was sieht denn der Therapeut anders als der Techniker oder der der die Prothese baut, als der sieht oder wahrnimmt? Wenn Sie sagen- #00:18:35-6#

Teilnehmer 39: Sie sehen nicht die Tagesform des Patienten. Sie wissen nicht, ob er Schmerzen hat, ob der heute irgend ‘ne auch ‘ne Druckstelle oder weiß der&weiß der Geier (Interviewerin: Mhm (bejahend)) was es da alles gibt. Da gibt’s ja X Komponenten, die der Therapeut erst sieht am Tag ähm wenn der zu einem kommt (Interviewerin: Mhm (bejahend)) und da&’s ist mannigfaltig. Weiß nicht, die (anderen haben?) vielleicht auch etliche Sachen zu sagen. Ähm- #00:18:56-2#

Teilnehmerin 36: Ich könnte mir vorstellen, was äh Technikern nicht immer so präsent ist, ist die unglaubliche Konzentrationsleistung (Interviewerin: Mhm (bejahend)), die der Patient grade zu Beginn äh bringen muss und ich glaube dass ähm muss&ist ganz wichtig, dass man dem Patienten es deutlich macht (Interviewerin: Mhm (bejahend)). Weil man ja, im Gegensatz zu ‘ner wirklich anstrengenden, körperlich&physisch anstrengenden Therapie das immer nicht sieht. (Interviewerin: Mhm (bejahend)). Und äh also Patienten da schon vermitteln, also nach zehn Minuten echt Pause, abschalten, durch den Raum gehen, Spaziergang machen, grade so am Anfang (Interviewerin: Mhm (bejahend)) das sind so Dinge, wo’s glaub ich manchmal so ‘n bisschen auseinandergeht. Weil der Techniker natürlich vor allen Dingen im Blick hat, welche Prothese grundsätzlich funktioniert, aber aber dann in ein Training reinzukommen ist&ist einfach der nächste Baustein (Interviewerin: Mhm (bejahend)), die sind ja auch ein Stück weit, ergänzen sich, aber sind ja unabhängig voneinander. (Interviewerin: Ja.) #00:19:48-7#

Teilnehmerin 38: Also wo ich die größte Differenz eher sehe ist, dass der Techniker mit seinem technischen Verständnis ‘ne ganz andere Versorgung schon sieht, was man alles machen <u>kann</u> und dann manchmal vielleicht so ’n bisschen verloren geht: Was braucht er eigentlich wirklich im Alltag? Weil der Techniker ja darauf basiert ist, die bestmögliche Versorgung zu machen, die überhaupt geht. Aber manchmal ist das Bestmögliche gar nicht das, was der Patient braucht. Das ist ja warum man oftmals Testversorgung macht, einfach um zu schauen ist das das Richtige, braucht er das. Es gibt tolle, multiaxiale Hände, die können dann neun Griffe, aber manchmal brauchen die das nicht. (Teilnehmer 39: Das sind die (...?)) Die brauchen einfach nur öffnen, schließen, Hausfrau, robuste Hand, bums, fertig. Aber sie hätte die Fähigkeiten was ganz anderes zu bekommen (Interviewerin: Mhm (bejahend)) an Prothesen(..?). Und das ist glaub ich das manchmal, wo man dann abwägen muss und dann in dieses, diesen Alltagsbezug mehr braucht. #00:20:36-3#

Interviewerin: Mhm (bejahend). Und wie sucht man das denn aus, eben auf den Alltag bezogen, was für ‘ne Prothese derjenige tragen sollte? Also wo sagt man jetzt hier sollte die gar nicht alles können, sondern am besten ist sie einfach möglichst einfach und kann nur dir Sachen, aber, weiß nicht, aber ist eben robust. Wonach würden Sie das aussuchen, wo würden Sie sagen wer das bekommen sollte? #00:20:57-5#

Teilnehmerin 38: Ja, je nachdem welche Ansprüche der Anwender erstmal an Prothese hat, also was ihm wichtig ist (Interviewerin: Mhm (bejahend)) und dann in was für ‘nem Umfeld er lebt. Also jetzt jemanden der viel im Garten arbeitet, dem würd‘ ich jetzt nicht die gleiche Hand empfehlen wie jemandem, der viel am Schreibtisch oder im Büro sitzt, (Interviewerin: Mhm (bejahend)) weil da der Anspruch (anders ist?). Also die Büro-Hand sag ich jetzt mal, die braucht nicht so robust sein wie eine, die ich jetzt auf der Baustelle oder im Garten benutze. Also

(Interviewerin: Mhm (bejahend)) es ist ‘ne schwierige Sache, weil eigentlich bräuchte man ja beides, (Interviewerin: Mhm (bejahend)) weil man irgendwo immer in den Bereich reinkommt, wo man präzise Arbeiten hat und auch mal robustere Arbeiten. Aber das ist noch so ’n Thema für sich, dass man eigentlich jedem Patienten Wechselversorgung anbieten müsste. Also dass man zum Beispiel eine robuste Hand hat und eine (Interviewerin: Mhm (bejahend)) präzisere, feinere Hand (Interviewerin: Mhm (bejahend)). Aber man schaut halt welche Hände auf dem Markt sind, versucht ihn möglichst viele ausprobieren zu lassen, damit nicht nur meine Meinung für ihn ausschlaggebend ist (Interviewerin: Ja.), sondern dass er’s halt äh selbst noch subjektiv bewerten kann was er braucht (Interviewerin: Mhm (bejahend)). #00:22:00-1#

Interviewerin: Und Herr (Name von Teilnehmer 37 aus Datenschutzgründen ausgelassen) fällt Ihnen noch irgendwas ein, wo so ’n, eben sowas was der Techniker sieht, oder was dem Techniker wichtig ist, ‘ne&also ‘ne Diskrepanz zu dem hat was man als Therapeut ähm also worauf man als Therapeut besonders wert legt? #00:22:17-3#

Teilnehmerin 38: Also z-&zur oberen Extremität kann ich da wenig zu sagen. #00:22:19-9#

Interviewerin: Ok, aber der Ansatz ist ja wahrscheinlich der gleiche? #00:22:24-5#

Teilnehmerin 38: (Darum?) auch keinen Patienten gehabt (Interviewerin: Mhm (bejahend)). Wie gesagt, ab’be Beine das ist mein Metier (alle lachen). (Teilnehmer 39: Ab’be Beine (lacht).) (kurze Pause) Ja, aber das geht in die Richtung, dass was ich auch schon gesagt hab, dass der&der Techniker oftmals weiß ok, wie das Passteil nennen wir’s mal äh funktioniert (Interviewerin: Ja.), was es leisten kann und ähm da dann so dies&dies&dies Training damit eher bisschen, ja nicht so sieht. (...?) dass&dass es wichtig ist, ja, dass der Patient auch ähm viel damit trainieren muss, damit er das können muss, was das Passteil hergibt (Interviewerin: Mhm (bejahend)). ‘Ne? Ist ja nicht so, wenn ich so ’ne äh wie heißen die da? Touchbionics-Hand nehme, kann ich auch nicht gleich hier Klavier spielen und auf ’m PC rumtippen (Interviewerin: Mhm (bejahend)), ‘ne? Wenn ich ‘n äh&äh Genium X3 habe, kann ich auch nicht gleich erwarten, dass das ja, dass der damit los joggt. (Interviewerin: Mhm (bejahend)) Das muss man alles erst üben und trainieren (Interviewerin: Ja.). Das ist&ist ‘n langer Weg. #00:23:26-4#

Interviewerin: Mhm (bejahend), also so ’n bisschen, dass um halt diese technischen Möglichkeiten die in so ’nem Ding überhaupt drinstecken, um die rauszuholen ist halt einfach Training und Therapie und so weiter notwendig, bis man überhaupt dahin kommt? #00:23:35-4#

Teilnehmer 37: (Genau, exakt?). #00:23:36-4#

Interviewerin: Mhm (bejahend), ja. #00:23:37-0#

Teilnehmer 37: Und in dem Zeitraum, ja das kennen Sie ja auch, muss man da auch wirklich äh intensiv zusammenarbeiten mit den Technikern (Interviewerin: Mhm (bejahend)) und die Techniker mit uns Therapeuten natürlich auch. (Interviewerin: Ja, ja, mhm (bejahend).) #00:23:51-7#

Interviewerin: Dann würd‘ ich gern jetzt aber mal nicht von der Patientensicht, sondern von Ihrer Sicht aus gucken. Nämlich wenn Sie sagen müssten was so das häufigste Problem oder die größte Schwierigkeit ist, wenn man jemanden rehabilitiert also, wenn jemand eben mit der Prothese kommt und man da auf diesem Weg ist das gemeinsam zu erarbeiten. Wo ist da so die größte Schwierigkeit, wo man hinmuss, wenn wir vielleicht mal nicht so auf das Ansteuern der Elektroden und so gucken, sondern eher auf Funktionen? Wenn man&also was da besonders schwierig ist ähm da reinzukommen oder wo da so die Probleme liegen. #00:24:25-1#

Teilnehmerin 36: Also ich würd‘ nicht mal auf die Funktion gehen. (Interviewerin: Mhm (bejahend)) Ich würd‘ auf die ähm psychologische Situation gehen. Die (finden?) auch wieder ganz unterschiedlich traumatisch oder Dysmelie, aber (Interviewerin: Mhm (bejahend)) ähm da fühl ich mich bei den Behandlungen extrem gefordert. Weil (Interviewerin: Mhm (bejahend)) nach Trauma, ähm wirklich so, dass die Patienten äh:m (Interviewerin: Mhm (bejahend)) große Probleme haben, psychologische und das kommt irgendwie indirekt in die Therapie rein, dass sich das zeigt, sagen die ab, sind die offen, sind die aufrichtig und das hat eigentlich wenig mit der Funktion zu tun, sondern mit dem Hintergrund den sie mitbringen (Interviewerin: Mhm (bejahend)) und bei&bei Kindern eigentlich ähnlich, dass da die Erwartung der&der Eltern ganz stark ist (Interviewerin: Mhm (bejahend)), die dann auch gehört haben das ist wichtig für Symmetrie und das muss jetzt und ähm da ist also Druck, unausgesprochene vielleicht auch Traurigkeit ähm der Eltern immer noch. Also das sind manchmal ganz komische Gemengelagen ähm und&wo ich manchmal auch merke da ähm&ähm da muss ich jetzt hingucken, da muss ich mich trauen ‘n Gespräch zu führen oder das anzusprechen (Interviewerin: Mhm (bejahend)), weil ich jetzt so rein funktionell ähm ich da gar nicht, eigentlich noch gar nicht hinkomme (Interviewerin: Mhm (bejahend), ja.). #00:25:55-6#

Interviewerin: Ok, also so dieses Psychologische als einfach erste Herausforderung, die einem da entgegensteht, um überhaupt erstmal dahin zu kommen, dass man halt gemeinsam arbeiten kann. Kann man das so festhalten? #00:26:07-8#

Teilnehmerin 36: Ja, bis dahin, dass es so Dinge die man im Laufe der Zeit erst versteht. Kann zum Beispiel auch so ’ne äh weitere Berufstätigkeit oder ein auch noch Richtung Rentner und (Interviewerin: Mhm (bejahend)) ähm weiß nicht (wie diese ganzen Fachbegriffe da?), Bezüge, also solche Sachen spielen da mit rein. (Interviewerin: Mhm (bejahend)) Dass der Patient vielleicht nicht mal, dass er das selber äh klar hat und reflektiert hat, dass er aber irgendwie auf der (Brende?) steht, weil er Angst hat, dann müsste er ja wieder sich integrieren und eventuell Kollegen damit konfrontieren. Also das sind manchmal so&so Dinge, die so ’n bisschen hochkommen wo ich denke: „Boah, das ist jetzt weit weg von eigentlich dem Handwerk was ich hier mache.“. (Interviewerin: Mhm (bejahend)) Ähm und ist aber wichtig, eigentlich äh weil&weil ich sonst ja gar nicht weiterkomme. (Interviewerin: Ja.) #00:26:55-2#

Teilnehmer 39: Ich denke auch die Lebenssituation in der er sich grade befindet und ähm was denn da überhaupt weitergeht bei dem, das ähm muss für denjenigen erstmal in irgendeiner Form auch geklärt sein und was heißt geklärt, das klärt sich ja meist nicht so schnell, überhaupt zu sehen ähm wie kommt man da weiter. Weiß ich Berufstätigkeit kann ja zum Beispiel auch sein, dass er (Interviewerin: Mhm

(bejahend)) erstmal eben nicht berufstätig ist, aber, nehmen wir weiß ich, ein Mann, Frau, Kind, hat grad ‘n Haus gekauft und ‘n traumatischer, ‘ne traumatische Sache, so und jetzt stellt er sich natürlich erstmal die Frage: Wie geht das alles weiter? Und da geht’s noch gar nicht darum, was willst du vielleicht im Job oder was willst du mit der Prothese, das ist sch-&auf ‘nem ganz andern Blatt Papier (Interviewerin: Mhm (bejahend)). Da muss man erstmal, überhaupt erstmal wieder in die Spur kriegen. Das mit diesem Absagen kenn ich genauso (Interviewerin: Mhm (bejahend)). Bis hin zu Eltern, die dann meinen, dass ihre Kinder das alles meistern müssten. Die aber ganz andere Sachen im Kopf haben, die nämlich vielleicht grade kein Arm, oder kein Bein haben und das mit Sicherheit nicht alleine können und die kommen dann garantiert nicht. Und dann wird’s versucht über andere Sachen zu of-&häufig auch noch abzuwälzen, sei es dann ‘n Taxiunternehmen, dass das gewährleisten soll oder sonstige Sachen, was total viel Spielchen gibt und da bin ich auch bei dir, das ist so, da ist viel Drumherum was man erstmal, muss man selbst ähm therapeutisch und auch von den Technikern etc. da haben wir diese Sachen schon erlebt, wo sich viele mit eingebracht haben und geguckt haben: Mensch, der muss erstmal in der Freizeit irgendwas wieder haben halt (Interviewerin: Mhm (bejahend)) und dann schafft man ihn auch über gewisse Kontakte die man hat und dann stellt man aber auf einmal fest, dass es trotzdem wieder zusammenbricht, weil ähm wenn er dann nicht abgeholt wird oder sonst was, derjenige, dann funktioniert doch wieder nicht, dann fängt man wieder von vorne an. Also das ist ’n, ist auch ‘n langer Weg. (Interviewerin: Mhm (bejahend)) #00:28:46-4#

Interviewerin: Und, wenn wir jetzt aber trotzdem, also ich weiß, dass das halt immer ‘n großer Punkt ist, aber wenn wir trotzdem mal dahin zurückgucken, dass wir jetzt sagen, jemand ist vielleicht auch schon länger bei Ihnen und wird einfach schon länger rehabilitiert und ist an die Prothese schon gewöhnt. Was gibt’s denn da so ähm, weiß nicht, an Problemen die vielleicht dann doch halt die Funktion betreffen, wo man sagt, weiß nicht, jemand kommt mit irgendwas und sagt: „Das sollte die eigentlich noch können, das würde ich gerne damit hinkriegen, aber der Weg dahin ist&ist lang.“ und wo sind da die Probleme? Wo&also wo sind da so die Schwierigkeiten zu sagen, dass man halt diese Prothese einfach zu ‘nem Alltagsgerät macht, das da funktioniert, also das was so der Patient an Erwartungen mitbringt, dass man da halt einfach hinkommt. Wo sind da so die Schwierigkeiten, wo muss man da durch, wenn man mal auf dieses Funktionelle achtet? #00:29:31-6#

Teilnehmer 39: Da muss man glaub ich erstmal gucken was ist realistisch möglich (Interviewerin: Mhm (bejahend)) und auch mit dem Patienten realistisch darüber sprechen, das und das hinzubekommen und dann eben auch ‘n Weg dahin aufzeigen, was heißt das zusätzlich was man machen muss und wenn irgendwas nicht realistisch ist, ähm dann muss man das auch offen kommunizieren denke ich, (Interviewerin: Mhm (bejahend)) das ist ganz wichtig. #00:29:52-5#

Interviewerin: Mhm (bejahend) und- #00:29:54-9#

Teilnehmer 39: Weil sonst gibt’s nachher ‘ne Enttäuschung, dass es nicht funktioniert. #00:29:57-7#

Interviewerin: Mhm (bejahend), ja. Und wo sind da aber die Grenzen? Also wo könnte man da sagen, das&das&da ist einfach die Grenze erreicht, das wird hier

unrealistisch, dass wir da hinkommen? #00:30:05-1#

Teilnehmer 39: (kurze Pause) (…??) #00:30:08-9#

Teilnehmer 37: Das ist (...?) so individuell. #00:30:13-8#

Teilnehmer 39: Ja, deswegen ja (lachend). #00:30:11-6#

Interviewerin: Ja aber wenn wir zum Beispiel mal Beispiele sammeln und einfach mal gucken, wo sowas zum Beispiel mal erreicht ist, wo man einfach&so wie wir vorher hatten eben jemand der im Garten arbeitet benutzt was anderes, als der der am Schreibtisch-. Also es ist klar, dass wir nicht jeden Aspekt hier sammeln, aber mal so Beispiele nennen wo&wo ‘ne Grenze ist? #00:30:27-7#

Teilnehmerin 38: So was die Funktion betrifft, wo wir (Interviewerin: Mhm (bejahend)) manchmal Schwierigkeiten haben, das ist halt dann im Bezug auf die Griffkraft (Interviewerin: Mhm (bejahend)). Also wenn jemand einen Sparschäler oder sowas halten will, oder das Besteck und die merken irgendwann ja, die Griffkraft ist nicht hoch genug, als dass ich wirklich das Messer in das Fleischstück reinstecken kann und dann auch noch hin-und herziehe. Also man ist dann irgendwie immer am Schauen wie kann ich das jetzt in der Hand miteinklemmen, dass sich das Messer in der Hand sperrt aber irgendwo ko-&stößt man da an seine Grenzen, weil man kann zum einen nicht immer die Finger alle verbiegen von der Hand, weil dann drücken sich die Motoren oder so kaputt (Interviewerin: Mhm (bejahend)) oder dann&manchmal ist es dann eben nicht möglich mit der Hand z-&also mit der Prothesenhand zu schneiden, (Interviewerin: Mhm (bejahend)) sondern dann muss man wieder umdenken, dann muss man’s halt andersrum machen, dann muss man mit der Prothese die Gabel halt festhalten. (Interviewerin: Mhm (bejahend)) Das ist halt, also Griffkraft ist wirklich das Einzige, würd ich jetzt mal sagen, wo man oft dran scheitert, aber es gibt immer direkt irgend ‘ne Alternative. (Interviewerin: Mhm (bejahend)) Weil man muss ja auch bedenken, dass die äh Anwender nicht sofort nach dem Unfall die Prothese kriegen. Das dauert ja immer ‘n paar Wochen, Monate, je nachdem wie schnell man das Ganze genehmigt bekommt und so. In der Zeit denken die sich schon so viel aus, wie sie’s anders hinkriegen (Interviewerin: Mhm (bejahend)) und das vermischen die dann nachher und deswegen ist das zwar ‘n Hindernis (Interviewerin: Mhm (bejahend)), aber (kurze Pause) irgendwie hat sich immer bis jetzt ‘ne Lösung gefunden bei uns. Oder Schuhe zubinden, hatten wir auch schon mal. Da hatte eine Dame ‘n&‘ne Hand mit der sie der Meinung war nicht genug Kraft aufbringen zu können, als dass sie das Band ziehen kann (Interviewerin: Mhm (bejahend)), weil wenn’s zu klein wird, (Interviewerin: Mhm (bejahend)) und zu viel Druck benötigt wird, dann geht das nicht. Weil oftmals, wenn die Finger dann aufeinander gehen, dann verrutschen sie auch leicht (Interviewerin: Mhm (bejahend)). Dann macht man nur ‘n kleines Stückchen auf, weil man nachgreifen will, aber dann haben sich die Motoren verändert, weil sie nicht wieder kalibriert wurden oder so (Interviewerin: Ja.) und dann sind sie dann verlaufen die Finger nicht mehr genau aufeinander und da hat man dann die Probleme (Interviewerin: Mhm (bejahend)) #00:32:14-6#

Interviewerin: Das heißt, wenn man die Prothese dahingehend verändern wollen würde, müsste es einerseits mehr Kraft in der Hand geben für solche Sachen wie schneiden, oder weiß nicht, vielleicht Werkzeug (Teilnehmerin 38: Mhm (bejahend))

und andererseits halt dieses, dass dieser feine Griff auch Kraft ausübt (Teilnehmerin 38: Ja.) und halt, weiß nicht da so genau aufeinander bleibt. #00:32:32-6#

Teilnehmerin 38: Genau, präzise bleibt. #00:32:32-3#

Interviewerin: Mhm (bejahend), ja. #00:32:33-8#

Teilnehmer 39: Ich denk mal die Schwierigkeit ist auch ähm, wir müssen natürlich jetzt, er sagte es eben schon, das ist sehr individuell (Interviewerin: Mhm (bejahend)). Je nachdem auf welche Höhe erstmal das Ganze ist, je höher ich mit dem ganzen (Interviewerin: Mhm (bejahend)) ähm ja, mit der ganzen Amputation dastehe, wenn der dann zu mir sagt: „Ich wünsche mir jetzt, dass ich mit meiner Prothese meinen Arm dahin heben kann." (Interviewerin: Mhm (bejahend)), das können Sie gerne ausprobieren mit so 'ner Prothese, wenn Sie da oben keinen Arm mehr haben. Wie wollen Sie das machen? Da müssen Sie 'ne Mechanik am Rücken oder was auch immer noch was entf-&erfinden und das ist ja häufig auch die Schwierigkeit. Das übt man ja auch mit denen, aber die haben häufig natürlich dann Sachen, wo sie sagen: „Mensch, ich möchte da und dabei hingreifen.", dazu müssten sie aber, weiß ich, den Arm in 'ne bestimmte Höhe kriegen. Da dann das noch wieder anzusteuern, ist natürlich deutlich schwerer als in einer entspannten Position, (Interviewerin: Mhm (bejahend)) solche Sachen und da muss man dann natürlich auch ausloten, wo ist denn die&die Grenze. Es gibt da bestimmte Sachen ähm das kann man gerne wollen, (Interviewerin: Mhm (bejahend)) aber bei bestimmten Versorgungen ähm wird der nie die Prothese dann so ansteuern können (Interviewerin: Ja, mhm (bejahend)). Es sei denn es gibt eine andere Ansteuerungsmöglichkeit. Dann&dann mag das vielleicht auch noch möglich sein. Da wäre dann allerdings wieder die Technik gefragt (Interviewerin: Mhm (bejahend)). Aber das sind, sind häufig Sachen ähm wo dann halt Grenzen der Prothese einfach da sind. Aber das hängt wie gesagt auch davon ab, was hab' ich vorliegen. #00:33:43-4#

Teilnehmerin 38: Wenn man die ganze Prothese betrachtet ist es ja manchmal wirklich, dass man einfach, man muss die Prothese ja an den Körper bekommen, also dass es hält (lacht) und manchmal schränkt man damit einfach den Beuge- oder den Streckwinkel ein, weil's nicht anders geht. Da hat man natürlich dann auch im Bewegungsausmaß Grenzen (Interviewerin: Mhm (bejahend)). Also das, aber ich&ich hab's jetzt so verstanden, als dass es mehr um die Greifkomponente jetzt erstmal geht, oder? #00:34:06-1#

Interviewerin: Genau, ja. Ich würd' gern eben so 'n bisschen halt einfach auf die Funktion-. Aber ich fand das schon mal ganz gut, was Sie gesagt haben mit diesem, dass eben dann da die Griffkraft fehlt, was man da eben verändern müsste. Aber das heißt eben auch mit so 'nem Sitz an der Prothese, das heißt, es müsste quasi anders befestigt sein und anders sitzen, damit halt hier das was ich mit&mit dem Arm bewegen möchte, nicht eingeschränkt ist? (Teilnehmerin 38: (nickt)) Mhm (bejahend). #00:34:29-9#

Teilnehmerin 36: Und die Situation der Griffkraft, die ist bei den Kinderhänden noch schlechter, (Teilnehmerin 38: Ja.) (Interviewerin: Mhm (bejahend)) die haben noch weniger Kraft. (Teilnehmerin 38: Das ist wirklich schlimm.) Und bei den Kinderhänden kommt noch hinzu, dass die auch ähm weniger ähm groß von

dem&das Bewegungsausmaß ist kleiner. Das heißt die können (Teilnehmerin 38: Die Griffweite ist das.) ‘ne Kinderhand, genau, die kann eine normale äh 0,7 Flasche nicht umfassen (Interviewerin: Mhm (bejahend)), diese (…?) und damit ist also, sind ganz viele Optionen, wo’s um halten geht (Interviewerin: Mhm (bejahend)), aber ich mein es geht jetzt wahrscheinlich weniger um Kinderhände aber das ist, äh begegnet uns regelmäßig. (Interviewerin: Mhm (bejahend)) Und da stellt sich natürlich schnell die Frage mit wofür (Interviewerin: Mhm (bejahend)) lohnt sich das noch? Also richtig festhalten geht nicht mit der Kinderhand und viele Dinge umgreifen auch nicht (Interviewerin: Mhm (bejahend)). #00:35:15-6#

Teilnehmerin 38: Da stoßen wir im Moment auch ganz oft an unsere Grenzen. Vor allem weil der Übergang schwierig ist, weil man einfach quasi nur diese kleinen Hände hat und dann, was ist das nächst größere? Es gibt, das ist für, ich sag mal für Jugendliche in ‘nem Alter von 14 Jahren, da passt die Größe irgendwann (Interviewerin: Mhm (bejahend)), aber was ist zwischen, ich sag jetzt mal sechs, sieben Jahre und 14? (Interviewerin: Mhm (bejahend)) Da ist so ungefähr nichts und das ist unheimlich schwierig, weil in dieser Zeit wird keine Prothese genutzt, weil die können damit nicht auf den Baum klettern oder in der Schule das benutzen (Interviewerin: Mhm (bejahend)), man kann noch nicht mal einen Prittstift damit aufmachen, weil einfach die Griffkraft nicht reicht und auch die Griffweite einfach viel zu gering ist. #00:35:46-2#

Interviewerin: Mit diesen Kinderhänden? #00:35:48-1#

Teilnehmerin 38: Mit den Kinderhänden, ja. #00:35:50-5#

Interviewerin: Ok, das heißt auch einfach die Schwierigkeit, dass eben wenn man wächst, dass es halt entweder das ganz Winzige oder das ganz Große gibt (Teilnehmerin 38: Ja.) und dass aber mit der ganz kleinen Hand einfach das, was man an Funktionen machen kann, stark eingeschränkt ist? (Teilnehmerin 38: Mhm (bejahend)) Mhm (bejahend). Und vorher (Name von Teilnehmerin 36 aus Datenschutzgründen ausgelassen), hattest du noch gesagt, dass das Gewicht für ganz viele auch so ähm als&als Problem angesehen wird, dass das ‘n Punkt ist. Gibt’s da irgendwas, wo man therapeutisch, weiß nicht, damit arbeiten kann, dass das weniger störend wahrgenommen wird, oder dass man das irgendwie umgeht? Weiß nicht, wenn&wenn das so als großes Problem von den Patienten wahrgenommen wird, gibt’s da irgendwas, was man da, wo man da ansetzen kann? #00:36:28-7#

Teilnehmer 37: Also ich denke auch, dass dann über die Zeit mitunter Gewöhnung (Teilnehmerin 36: Mhm (bejahend)) dann langgeht. (Interviewerin: Mhm (bejahend)) Genauso je mehr man äh mit&mit arbeitet, trainiert, übt, (Interviewerin: Ja.) kräftigt sich ja auch alles uUmliegendeund dann wird das Passteil irgendwann nicht mehr so schwer empfunden als das am Anfang dann (die Meinung?) war (Interviewerin: Mhm (bejahend)). ‘Ne? (Interviewerin: Ok, ja.) #00:36:52-9#

Teilnehmerin 36: Also das, aus meiner Sicht so, betrifft das auch mehr die ähm Schulter-Ex Patienten (Interviewerin: Mhm (bejahend)) ähm und ähm das was du sagst, dass man dann aber auch den Patienten sagt es geht gar nicht ums Ansteuern, (Interviewerin: Mhm (bejahend)) sondern nur ums Tragen. Also dass die nicht das Gefühl haben, wann immer sie die Prothese tragen, muss die auch in

Aktion sein (Interviewerin: Mhm (bejahend)). Sondern die können die einfach nur anlegen, die müssen die gar nicht anmachen (Interviewerin: Mhm (bejahend)), so. #00:37:19-5#

Interviewerin: Ja. Das heißt, dass&dass die Funktionen vielleicht gar nicht immer so wichtig sind, sondern auch was wir ganz am Anfang gesagt haben, halt auch dieses, diese Vollständigkeit, davon dass halt wieder was da ist? #00:37:29-9#

Teilnehmerin 36: Ä::hm ich meinte das jetzt eher (Interviewerin: Mhm (bejahend)) im Hinblick auf sich an das Gewicht (Interviewerin: Ok, ja, mhm (bejahend).) gewöhnen. Also unabhängig (Interviewerin: Ja, ok.) davon wie der Patient das wahrnimmt, ob er sich vollständig oder nicht, aber- #00:37:42-2#

Interviewerin: Ok, dass man die einfach anlegt, ohne dass die irgendwie funktioniert, (Teilnehmerin 36: Genau.) dass man sich einfach dran gewöhnt, dass&dass die da ist und dass die so viel wiegt? #00:37:48-5#

Teilnehmerin 36: Mhm (bejahend) #00:37:48-5#

Teilnehmerin 38: Im Grunde genommen stößt man ja&stößt man manchmal an seine Grenzen, wo weder der Therapeut, noch der Techniker viel dran ändern können. (Interviewerin: Mhm (bejahend)) Wenn man jetzt zum Beispiel ‘n ultra kurzen Unterarmstumpf hat, dann ist der Hebel einfach unheimlich lang (Interviewerin: Mhm (bejahend)). Weil man möchte ja auch wenn man auf das Optische eingeht und keine extreme Längendifferenz haben, warum man ja die Prothese dann nicht viel viel (Interviewerin: Ja.) kürzer macht, als (Interviewerin: Ja.) die erhaltene Seite, oder ich weiß immer nie wie ich das ausdrücken darf, auf jeden Fall als die andere Körperseite (Interviewerin: Mhm (bejahend)) und natürlich kann man bestimmt irgendwie die Muskulatur etwas stärken. Durch ’s Tragen sich an das Gewicht gewöhnen, aber letztendlich bleibt ja die physikalische Komponente (Interviewerin: Mhm (bejahend)) mit dieser extremen Hebelwirkung mit der schweren Hand am Ende (Interviewerin: Ja.). Es ist unheimlich schwierig für die Leute weit weg vom Körper zu greifen (Interviewerin: Mhm (bejahend)). Das ist einfach zu schwer. Also ich denke von beiden Seiten kann man leichte Kompromisse machen, (Interviewerin: Mhm (bejahend)) also was heißt Kompromisse. Von der Technikerseite aus, dass man vielleicht kürzer wird, die Akkus, alles nach oben verlagert, um das Gewicht möglichst nah am Stumpf zu halten (Interviewerin: Mhm (bejahend)) und von der Therapeutenseite eben durch Muskelkräftigung versuchen das Gewicht besser tragen zu können aber, perfekt machen kann (Interviewerin: Ja, mhm (bejahend).) man das keinem. #00:38:56-6#

Interviewerin: Ähm und dann haben wir ganz am Anfang auch mal erwähnt, dass&dass eben oft die Leute kommen und die Prothese in der Tasche liegt und dass man&dass die eben gar nicht getragen wird. Was sind denn so die Hauptgründe dafür, dass die dann&dass die nicht akzeptiert wird, dass die nicht benutzt wird? Also ‘n paar haben wir jetzt bestimmt schon gesammelt, es ist auch nicht schlimm, wenn sich’s doppelt, wenn wir da einfach nochmal gemeinsam überlegen, was so die&die wichtigsten Gründe dafür sind, dass jemand sagt: „Die passt halt nicht, die trag ich nicht.“. #00:39:23-2#

Teilnehmerin 38: Das ist das eigentlich, wenn man merkt, dass es ohne Prothese

schneller geht. (Interviewerin: Mhm (bejahend)) Dann wird die Prothese weggelegt. Das ist oftmals bei langen Unterarmstümpfen. Weil einfach die Längendifferenz nicht so extrem ist, da ist es ganz schwierig ‘n Bewusstsein zu schaffen, warum die Prothese besser wäre, oder was damit schneller gehen kann, wenn man übt, weil da wird dieses Training meistens einfach nicht mehr durchgezogen. Weil die merken: „Nee, geht doch schneller ohne.“ (Interviewerin: Mhm (bejahend)). Und ja, wenn die Passform nicht richtig ist oder wenn Ausschläge entstehen oder sowas (Interviewerin: Mhm (bejahend)), dann ist natürlich auch klar, dass die Prothese in der Tasche landet. Aber das ist ja eher so von der Technikerseite aus (Interviewerin: Ja.). Ja. Ich glaube, wenn die Funktionen gut anzusteuern sind und der Anwender sich darüber bewusst ist, welchen Vorteil er hat, dann wird die Prothese auch wirklich viel getragen, also (Interviewerin: Mhm (bejahend)), also so sind meine Erfahrungen. Also die meisten bei uns tragen sie wirklich viel (Interviewerin: Mhm (bejahend)). #00:40:19-8#

Teilnehmer 39: Man muss das-&man muss einfach sehen, warum, oder das sind ja meist mehrere Sachen, äh am Ende ist es nachher so, dass er ‘se nicht rich-&nicht richtig ansteuern kann sie f-&und dann kommt vom Patienten natürlich nur: „Sie funktioniert nicht.“ (Interviewerin: Mhm (bejahend)), die Frage ist natürlich: Warum funktioniert sie nicht? Passform, ähm ist die Abnahme vielleicht falsch, was auch immer. Können X Gründe sein. Oder hat er ‘se einfach lange nicht mehr getragen (Interviewerin: Mhm (bejahend)) ähm da hat sich an&an&an seinem Stumpf schon was geändert (Interviewerin: Mhm (bejahend)) und ja. Das sind eigentlich so die&die Gründe. Also wir machen’s grundsätzlich so, eigentlich bei allen Prothesentrainern, die haben halt zusätzlich ‘n Trainingsprogramm ohne Prothese, das heißt wir lassen uns von&von der Technik ähm (Interviewerin: Mhm (bejahend)) Schäfte anfertigen, an diesen Schäften sind an diversen Stellen, je nachdem was er trainieren soll ähm Ösen angefertigt. Das heißt die machen Übungen bei uns am Seilzug, ähm also Kräftigungsübungen (Teilnehmerin 36: Mhm (bejahend)), ganz gezielt auf die Sachen. Und ohne das, kommen die bei uns auch nicht raus (Interviewerin: Mhm (bejahend)) und das hat einfach zur Folge, dass die ähm ja, deutlich besser auch ansteuern können in den Bereichen dann und ähm natürlich dann auch die Prothese besser funktioniert. Und ich hab‘ ähm nebenbei dann ähm auch noch den Effekt, dass ich dann nicht unter Umständen den Fall hab, dass der Stumpf (Interviewerin: Mhm (bejahend)) ähm kleiner wird, sich groß verändert, sondern-. Es sei denn der würde da jetzt-. Es ist kein Bodybuildingtraining was die da machen (lacht) (Interviewerin: Mhm (bejahend)). Ähm also das haben wir noch nicht gehabt, dass da jemand dann dementsprechend auf einmal Muskelmasse (...?) (Interviewerin: Mhm (bejahend)). Aber das sind halt so Möglichkeiten, wo man das vielleicht ‘n bisschen beeinflussen kann dann. #00:41:47-8#

Interviewerin: Ja, mhm (bejahend). #00:41:51-0#

Teilnehmerin 36: Ähm. Ich finde es gibt noch die Aspekte beim im Hochsommer und im kalten Winter (Teilnehmerin 38 und Teilnehmer 39: Ja.), diese Temperaturgeschichten. (Teilnehmer 39: Das stimmt.) Also es ist alles wunderbar, ja (Interviewerin: Mhm (bejahend)), von der Technikerseite und der Patient ist motiviert, aber im Sommer, wenn die stark schwitzen (Interviewerin: Mhm (bejahend)), dann ähm ist das unangenehm und es besteht auch die Gefahr, dass die Prothese rutscht (Interviewerin: Mhm (bejahend)). Und im Winter haben die oft richtig ‘n kalten Stumpf in der Prothese. (Interviewerin: Mhm (bejahend)) Also die ähm&h- Gründe gibt’s

auch. #00:42:20-5#

Interviewerin: Ja. #00:42:18-7#

Teilnehmerin 38: Da kann man ja Schaftheizungen einbauen. (lacht) #00:42:22-2#

Teilnehmer 39: (lacht) #00:42:23-5#

Teilnehmerin 36: Ehrlich? Oder ist das jetzt Spaß? #00:42:22-2#

Teilnehmerin 38: Ja, nein, klar, natürlich. #00:42:25-5#

Teilnehmerin 36: Ich muss jetzt mal wieder aufschreiben, dass ich ‘n paar Sachen hab (alle lachen). #00:42:31-9#

Interviewerin: Und gibt’s aber auch irgendwas, wo jemand ‘ne Prothese zu ‘nem ganz bestimmten Zweck halt haben wollte, um weiß nicht&um weiß nicht, zurück in Beruf zu gehen oder um einfach irgendwas besonderes wieder zu schaffen und wo da die Prothese das dann einfach vielleicht nicht erfüllt, oder halt nicht&nicht richtig funktioniert und dann gesagt wird: „Naja, dann&dann bringt die nichts.“? Gab’s da irgendwelche Beispiele oder Fälle von denen Sie berichten können? #00:42:56-7#

Teilnehmerin 38: Also es ist erstmal alles irgendwie möglich, also wir haben schon Schäfte gebaut wo ‘ne Axt nachher draufgeschraubt wurde oder sowas (alle lachen), (um?) da auch noch Holz hacken zu können und so, aber es gibt auch den Fall, also das hatten wir bei einem, der hatte einen Finger nur verloren und wollte gerne noch den Abzug seines Gewehrs ziehen können (Interviewerin: Mhm (bejahend)) und da war’s uns nicht möglich irgendwas zu machen, dass das wirklich noch funktioniert. (Interviewerin: Mhm (bejahend)) Weil der Hebel, das passte irgendwie alles nicht und so. Und dann (hat er jetzt?) gesagt letztendlich: „Nee, das macht keinen Sinn da irgendwo mit ‘ner Prothese was zu machen.“ (Interviewerin: Mhm (bejahend)). Ich glaube das ist oft im Teilhandbereich, dass man da schauen muss, ob ‘ne Prothese Sinn macht oder nicht (Interviewerin: Mhm (bejahend)). Weil je nachdem wie viel noch erhalten ist und wie auch die Möglichkeit ist die Prothese anzusetzen, also die Finger die einzelnen, kann man schauen ob das Sinn macht oder nicht. (Interviewerin: Mhm (bejahend)) #00:43:45-6#

Teilnehmerin 36: Kennst, ich sag jetzt einfach mal du, kennst du diese äh Websites (wendet sich an Teilnehmerin 38) ähm aus USA, die im Hinblick auf verschiedene, ganz individuelle Anwendungen ähm (Teilnehmerin 38: Nee, ich glaub nicht.) Vorschläge haben? Dann schick ich dir das mal. #00:43:58-2#

Teilnehmerin 38: Ja. (lacht) #00:43:56-3#

Teilnehmerin 36: Dann tauschen wir nachher noch Mails-ähm-adressen aus. #00:44:00-2#

Protokollant: Also ganz individuelle, nicht ganz, (Teilnehmerin 36: Ganz individu-) im Sinne von? #00:44:04-7#

Teilnehmerin 36: Also ähm eben so Richtung Gewehr, (Protokollant: Achse.) äh

angeln, Golf spielen (Teilnehmerin 38: Ach so, ja doch&doch&doch, den Link hatt‘ ich schon.) ähm wir haben da jetzt was bestellt für ein Kind was schwimmen möchte (Interviewerin: Mhm (bejahend)) und einfach äh die ist sehr engagiert beim Schwimmen und die möchte einfach ‘n bisschen mehr Hebel ähm, also bisschen mehr Wasser wegdrücken können. Also da sind wir noch dabei (Teilnehmerin 38: Ja.), deshalb kann ich noch gar nicht sagen, ob das jetzt funktioniert. Aber da braucht man ja manchmal Ideen und sei es, dass man nur draufguckt, was die jetzt machen. Ja. #00:44:30-5#

Teilnehmerin 38: Das ist ‘ne gute Idee! #00:44:34-8#

Interviewerin: Ok, dann wüsst‘ ich gerne als nächstes, also es wird sich, wahrscheinlich wieder werden sich jetzt einige Punkte doppeln, was aber nicht schlimm ist, ähm was muss die Prothese denn aus Ihrer Sicht an so Eigenschaften und Funktionen erfüllen, damit man damit möglichst gut am Alltag wieder teilnehmen kann und möglichst gut wieder sich integrieren kann und einfach, weiß nicht, um eben möglichst kein Handicap zu haben, sondern einfach so teilnehmen zu können, fast wie davor? Was sind so- #00:45:01-8#

Teilnehmer 39: Das Wichtigste auch kam ja eben schon mehrfach auf (Interviewerin: Ja.), ist die Greiffunktion erstmal für die meisten (lacht). #00:45:06-3#

Teilnehmerin 38: Also ich find es ist nicht wichtig, dass eine Hand super viele Griffe hat, sondern (Interviewerin: Mhm (bejahend)) sie muss stabil sein, meiner Meinung nach, weil es nützt dem Anwender nichts, wenn er alle zwei Wochen wieder zu uns rennen muss, die Hand muss zum Service geschickt werden oder zur Reparatur, weil schon wieder irgendein Finger abgebrochen ist oder so. #00:45:25-6#

Interviewerin: Ok, stabil das heißt, dass sie einfach viel Gewicht aushalten muss, oder- #00:45:32-1#

Teilnehmerin 38: Mhm (bejahend), viel&ja viel Gewicht, Druck, alles. (lacht) #00:45:33-3#

Interviewerin: Ok. (lachend) #00:45:34-0#

Teilnehmerin 38: Also, dass das Material (Teilnehmer 39: (...??)) nicht zu nachgiebig sein darf. (Interviewerin: Ja, mhm (bejahend).) Und ähm, also es ist schon gut, wenn verschiedene Griffe vorhanden sind, aber meiner Meinung nach m-&muss eine Hand nicht bis zu neun Griffen haben, weil meistens werden eher so vier verschiedene Griffe benutzt. Also (Interviewerin: Mhm (bejahend)) mal ‘n bisschen was zu präziserem Greifen, bisschen was, wo ich mehr Kraft aufbringen kann, dann ‘ne offene Hand um was zu tragen (Interviewerin: Mhm (bejahend)) ja und dabei bleibt’s eigentlich schon. Also-. Das ist, also robust muss sie sein und ein bisschen verschiedene Greifmöglichkeiten. Weil man kann dem Anwender ‘ne Prothese nicht als Ersatz der Hand verkaufen (Interviewerin: Mhm (bejahend)), sondern nur als Hilfsmittel. #00:46:18-2#

Teilnehmer 39: Das stimmt. #00:46:18-2#

Interviewerin: Ja, mhm (bejahend). #00:46:19-6#

Teilnehmerin 36: ‘Nen Knackpunkt sind die Handschuhe. #00:46:22-2#

Teilnehmerin 38: Ja. #00:46:20-4#

Teilnehmerin 36: Denn ähm die s-&müssen auf der einen Seite äh müssen die irgendwie robust sein (Interviewerin: Mhm (bejahend)), äh dann müssen sie wasserdicht sein, das heißt die dürfen nicht an irgend ‘ner Stelle kaputt sein (Interviewerin: Mhm (bejahend)) und, also soweit ich weiß, sind die Patienten, zumindest unsere Patenten, wechseln die Handschuhe selber nicht (Teilnehmerin 38: Mhm (verneinend)). Das heißt ähm (Teilnehmer 39: Richtig.) und&und grad bei Kindern, ja? Dann wollen die eigentlich matschen oder irgendwie malen, dann ist der wieder schmutzig (Interviewerin: Mhm (bejahend)), dann ähm also dieser Handschuh als&als Oberfläche, ähm aber auch vom Material (Interviewerin: Mhm (bejahend)) ähm das ist ‘n ganz schwieriges Thema (Interviewerin: Mhm (bejahend), sind zum Teil wahnsinnig empfindlich, dann aber auch weich und lassen die Griffe zu (Interviewerin: Mhm (bejahend)) und f-&ich finde das ist irgendwie ganz äh problematisch, (Interviewerin: Mhm (bejahend)) dass der Handschuh selber nicht gewechselt werden kann. #00:47:11-2#

Interviewerin: Ok, das heißt es müsste eigentlich so sein, dass man eigentlich selber austauschen kann. (Teilnehmerin 36: Ja.) Je nachdem was man grade macht? #00:47:15-7#

Teilnehmerin 36: Genau. #00:47:18-6#

Interviewerin: Ja. #00:47:18-6#

Teilnehmerin 36: Ja. #00:47:19-7#

Teilnehmerin 38: Ja, das wär‘ echt. ‘N robuster Handschuh, der sich nicht von innen, also wenn&wenn man mehrere Gelenke hat an der Hand (Interviewerin: Mhm (bejahend)), drückt sich ja irgendwann an der Stelle, wo das Gelenk häufig bewegt wird, (Interviewerin: Ja.) der Handschuh auch durch (Interviewerin: Mhm (bejahend)). Dann ist es wieder nicht mehr wasserabweisend, weil Feuchtigkeit (Teilnehmerin 36: Ja.) an die Hand drankommen kann, oder auch Sand, das ist noch schlimmer und das Material soll aber trotzdem, so ’n PVC-Handschuh der’s sehr schmutzempfindlich. (Teilnehmerin 36: Ja.) Wenn ich da einmal mit ’m Kuli draufgemalt habe, das kriegt man nie wieder ab. (Interviewerin: Mhm (bejahend)) Aber die Silikonhandschuhe sind zu weich, als dass sie zu schnell kaputtgehen, also (Teilnehmerin 36: Mhm (bejahend)) das ist richtig schwierig. #00:47:48-0#

Teilnehmer 39: Ja, ist auch so, ja. #00:47:47-9#

Interviewerin: Mhm (bejahend), das heißt dafür wär die Lösung einfach, dass man auch so wie wir vorher gesagt haben eigentlich für jede Situation halt einfach einen hat, der entweder eben dann leicht zu reinigen ist, oder der halt einfach widerstandsfähig ist? (Teilnehmerin 38: Mhm (bejahend)) Ok. Mhm (bejahend) #00:48:03-9#

Teilnehmerin 36: Und, dass der Patient das selber ähm an- und ausziehen kann,

‘ne? #00:48:06-0#

Interviewerin: Mhm (bejahend), ja. (Teilnehmerin 38: Ja das wär‘ (...???).) #00:48:06-5#

Teilnehmer 39: Weil wenn man dann den Techniker fragt, wie problematisch es ist allein den Handschuh (Teilnehmerin 36: Ja.) darüber zuziehen, wenn das jetzt ein Patient mit einer Hand, wohlgemerkt, (Teilnehmerin 38: Da gibt’s auch welche, die das schaffen (..??)) selber machen soll ähm sind wir wahrscheinlich eher wieder dabei: Was abnehmen, was neues draufschrauben ähm, (Interviewerin: Mhm (bejahend)) (Teilnehmerin 36: Mhm (bejahend)) ich denke, gut, wäre&wäre schön, (Teilnehmerin 36: Wir wünschen uns jetzt erstmal nur, es ist kurz vor Weihnachten.) das&das&das ’n Wunsch, da sind wir bei realistisch und was&derjenige (Teilnehmerin 36: Ja.) muss es ja selber umsetzen können und wie gesagt (Teilnehmerin 38: Ja.), sie kommt aus der Technik (zeigt auf Teilnehmerin 38) dann weiß sie, wie aufwendig das ist, einen Handschuh darüber zu kriegen (Interviewerin: Mhm (bejahend)) oder den Handschuh (wenn?) man ihn tauscht. #00:48:41-6#

Teilnehmerin 38: Das wär‘ ja alles nicht so schlimm, wenn das jetzt einmal in ‘nem halben Jahr wäre zum Tauschen (Teilnehmer 39: Ja.), wenn das so lange halten würde. Aber das tut’s ja leider nicht immer. (Interviewerin: Mhm (bejahend)) #00:48:48-1#

Teilnehmer 39: Ich denke die Schwierigkeit dann tatsächlich, kam ja eben schon, diese unterschiedlichen Materialen, ich weiß nicht die werden wahrscheinlich bekannt sein. Auch die Firma (Name eines Prothesenherstellers aus Datenschutzgründen ausgelassen) hat ja ein ähm die können ja inzwischen auch den Arm so nachbilden ähm wie der andere und ich kann mit ’m Kugelschreiben raufschreiben und einmal wischen und das Zeug ist weg. Solche Materialen haben sie, die Schwierigkeit dabei ist aber eben, wo sie ja auch offensichtlich noch forschen, wie krieg ich das mit der Bewegung hin? Wie krieg ich das mit ’m (...?) hin, weil das Material es offensichtlich ja auch noch nicht so hergibt (Teilnehmerin 36: Ja.) (Interviewerin: Mhm (bejahend)), wie sie’s gerne wollten. Aber da ist ja wahrscheinlich auch noch nicht aller Ende der Forschung (lacht) sag ich mal. #00:49:26-3#

Interviewerin: Ähm ok, das heißt wir haben jetzt den Handschuh, so dass was der abhaben äh abkönnen muss. Ähm dann, dass die Hand vor allem robust sein sollte und eigentlich gar nicht so super viel verschiedene Greifmuster haben muss. Hauptsache sie ist halt robust und&und es funktioniert. Gibt’s noch irgendwas, was die erfüllen muss, was die haben müsste, damit man möglichst gut im Alltag damit klarkommt? #00:49:49-2#

Teilnehmerin 38: Also schön ist natürlich, wenn sie optisch gesehen sich nicht so sehr zu der ähm anderen Hand unterscheidet. (Interviewerin: Mhm (bejahend)) Also es gibt ja Hände, die sind sehr sehr kantig, sehr breit. So zum Beispiel wie die Hand von Touchbionics, die I-limb, die find ich sehr kastig, wobei die äh Michelangelo-Hand, die sieht optisch sehr gut aus (Interviewerin: Mhm (bejahend)). Also die hat auch diesen natürlichen Handmodus drin (Interviewerin: Mhm (bejahend)), das heißt man läuft nicht immer mit so ’ner offenen Hand oder mit ‘ner Faust durch die Gegend. Also ich glaube, dass das für viele auch nochmal ‘n

zusätzlicher Punkt ist, was eben die Optik angeht, warum&ob ‘ne Prothese getragen wird oder nicht. #00:50:23-3#

Interviewerin: Und das heißt quasi, wenn ich mit der Prothese nichts mach, dass die dann halt einfach ‘ne&‘ne Form oder einfach ‘ne Haltung annimmt, die halt normal aussieht? #00:50:31-6#

Teilnehmerin 38: Ja, genau. So ’ne (Interviewerin: Physiologisch?) natürliche, entspannte Hand (Interviewerin: Mhm (bejahend)). Ja. #00:50:35-7#

Interviewerin: Ähm dann würde mich noch eine Sache interessieren, das haben wir ganz am Anfang äh ganz kurz einmal erwähnt, dass für viele so das Ding ist eben die Prothese ist kalt, ist schwer, man fühlt damit nichts. Ähm mich würd‘ interessieren, weil wir da ganz verschiedene Meinungen bisher gehört haben was sowas angeht wie dass&dass man in die Prothese ‘ne Art Tastsinn einbaut. Also dass man irgendein Feedback darüber bekommen kann, einerseits vielleicht wirklich was&was man fühlen kann, ‘ne Oberflächenbeschaffenheit oder ‘ne Temperatur oder sowas und andererseits eben auch ‘ne Rückmeldung zu bekommen darüber, in welcher Position sich die Prothese gerade befindet, oder weiß nicht, mit&mit was für ‘ner Kraft ich irgendwas äh berühre oder eben wie viel&wie viel Kraft bei ‘nem Griff mit drin ist. Ist sowas wichtig? Wär‘ sowas was, was es den&den Leuten erleichtern würde, aus Ihrer Sicht? #00:51:20-7#

Teilnehmer 39: Das kommt drauf an, wie die Rückmeldung erfolgt. (Interviewerin: Mhm (bejahend)) Ich äh, wenn ich das jetzt so höre, erinnert mich das eher an äh, nennen wir’s mal Assistenzsysteme in einem Auto. Klar kann ich da tausend reinmachen (Interviewerin: Mhm (bejahend)), aber die Frage ist, kann ich mich dann noch auf das Eigentliche konzentrieren (Interviewerin: Mhm (bejahend)) und kann derjenige dann wirklich noch mit seiner Hand greifen, oder guckt der wie auch immer das dann gemacht wird, ähm kriegt er dann Leuchten oder was, wo er sich noch drauf konzentrieren muss, wie stark er dann greift. Weil schlussendlich, die die’s gut ähm handeln können (Interviewerin: Mhm (bejahend)) und mit gut handeln mein ich, wir haben hier jetzt Gläser stehen, nehmen wir mal an, wir hätten jetzt diese Plastikbecher, die’s weiß ich, auf ’m Weihnachtsmarkt vielleicht auch gibt. Ich hab‘ da jetzt mein Wasser drin und die Kunst ist es dann ja für den Patienten dieses Glas zu nehmen ohne, dass er’s zerquetscht. (Interviewerin: Ja.) Und das bekommen die, die sie gut ansteuern können (Interviewerin: Mhm (bejahend)) auch so hin. Und dann stellt sich mir die Frage, wenn ich jetzt noch ‘ne Rückmeldung kriege, wie stark ich drücke, mh ob das dann noch ‘n Sinn für denjenigen macht (Interviewerin: Mhm (bejahend)). Also ‘n Gefühl ähm wie kriegt er das Gefühl vermittelt? Er hat ja keinen Tastsinn (Interviewerin: Mhm (bejahend), ja.). Wie soll er das vermittelt kriegen? #00:52:29-9#

Teilnehmerin 38: Gab’s da nicht mal von (Name eines Prothesenherstellers aus Datenschutzgründen ausgelassen) auch diese Hand, diese Sensor-Hand oder so, die haben sie schon aus dem Sortiment wieder rausgenommen, mein ich. Das war (Teilnehmer 39: (..??)) eine, die nachgegriffen hat (Teilnehmer 39: Ja, und zwar-) und das war das Problem, auch wenn die Gegenstände rausgerutscht sind, sollte sie eigentlich nachgreifen, aber grade beim Hände geben oder so dann hat sie nur noch zugedrückt (Interviewerin: Mhm (bejahend)) wenn einer rausgezogen hat also ich glaube das ist technisch (Teilnehmer 39: Nee, die&die greift nachher so, die

Schulung hab ich auch selber gem-&das war glaub ich sogar die, wo wir da oben im (..?) (lachend) genau.) auch sehr schwierig umzusetzen. #00:52:53-4#

Teilnehmerin 36: (Diese?) SensorSpeed? #00:52:53-6#

Teilnehmerin 38: Ja, ich (…?)- #00:53:00-6#

Teilnehmer 39: Genau. Und das Problem dabei ist tatsächlich, wenn ich jetzt demjenigen, ich hab‘ auch mit solchen Patienten gearbeitet, das war tatsächlich mit der Abschlusstest, sag ich mal, bevor ich die da bei uns komplett entlassen habe, denke hab ich die Hand gegeben. Und der ähm, es ist natürlich so, wenn die, das geht&ging um ‘ne PT-Flasche. Wenn man die öffnet, das war die Idee die dahintersteckte, dass die Hand nachgreift (Teilnehmerin 36: Mhm (bejahend)), dass&weil die Luft ähm rausgeht, dass da&dass die&dass sich da durch die Veränderung, dass die das trotzdem noch halten kann. Nachteil ist tatsächlich, wenn derjenige zugreift (Interviewerin: Mhm (bejahend)) und dosiert es nicht richtig und die greift nach und ich fang dann an zu ziehen (Teilnehmerin 36: Mhm (bejahend)), dann fängt die Hand an zu greifen, das gibt auch ‘ne Quetschung. #00:53:34-6#

Teilnehmerin 38: Also die Grundidee find ich nicht schlecht, aber ich (Teilnehmer 39: Wie gesagt-) in welcher Form kann man (Teilnehmer 39: Also er hat aber ja trotzdem keinen&keinen sensorischen Inpu-&Rückpu-&ähm Input gekriegt, wieder selber der Patient. Das war ja das, was Sie grad sagten, so hab‘ ich Sie zumindest verstanden, (Interviewerin: Ja, mhm (bejahend)) dass derjenige ‘ne Rückmeldung kriegt. Das ist ja noch was anderes, da macht die Hand das ja automatisch, er hat aber immer ja noch kein-&kein&ja, keine Rückmeldung.) das umsetzen, dass ist glaub ich (hier die Frage?). Ach so ja. (Interviewerin: Ok, aber-) Aber das haben manche Hände doch schon (Interviewerin: Mhm (bejahend)), wenn man zugreift und die volle Endkraft sozusagen aufgebaut ist, dann ist da so ’n Vibrator, Vibrator (lachend), (alle lachen) Vibrationssignal drin, (Teilnehmer 39: Das Handy vibriert (...?)) und ähm dann ähm brummt es zum Beispiel zwei Mal (Interviewerin: Mhm (bejahend)), wenn man jetzt nicht weiter zugreifen kann (Interviewerin: Ja.). Also, für v-&viele ist (Teilnehmer 39: Ist das-) das gut, um zu wissen, dass man da eben jetzt wirklich nicht weiter greifen kann und dass&dass der Gegenstand in der Hand jetzt sozusagen gut gesperrt ist (Interviewerin: Mhm (bejahend)). #00:54:26-6#

Interviewerin: Wenn wir das aber ganz kurz einmal sortieren. Das heißt, Sie haben gesagt, vom Ding her, wenn&wenn ich richtig trainiert bin, wenn ich&wenn ich quasi mit meiner Prothese gut umgeh, die gut ansteuern kann, dann funktioniert das eigentlich auch so, dann ist das eigentlich nicht nötig. Oder dann ist es halt viel mehr nochmal so ’n, so ’ne, weiß nicht, ‘ne Störung, dass&dass wenn hier noch was vibriert oder drückt oder wie auch immer man es dann löst, weil ich dann halt nochmal irgendwas hab, was mich halt irgendwie ablenkt von dem, was ich eigentlich tun soll? #00:54:50-9#

Teilnehmer 39: Ich bin grade ähm so auf der (Interviewerin: Mhm (bejahend)) Ebene, wenn wir über fühlen reden (Interviewerin: Ja.), dann stellt sich der Patient vor (Interviewerin: Ja.), dass er wieder fühlen kann (Interviewerin: Mhm (bejahend)). Er wird aber nicht wieder fühlen&fühlen können mit der ähm Hand, es sei denn Sie können ihm irgendein Gefühl im Gehirn vermitteln, was die Hand fühlt (Interviewerin: Mhm (bejahend)). Dann mag das vielleicht auch möglich sein. Aber das ist ja das,

was ich, wenn ich zu dem Patienten sag, er kann wieder fühlen, dann erwartet er von mir, dass er kann Taubheitsgefühl (Interviewerin: Mhm (bejahend)) oder kein Dings, sondern er&er fühlt die Oberfläche. (Interviewerin: Ja.) Und das bringt&er fühlt die Oberfläche ja nun nicht, wenn ich ihm wie auch immer durch einen Ton (Interviewerin: Mhm (bejahend)), durch ein Bild oder was auch immer sage, das ist jetzt weich, das ist jetzt hart. Das kann er dann vielleicht sehen (Interviewerin: Ja.), aber damit hat er immer noch kein Gefühl und das ist für den Patienten oder aus meiner Sicht ist das&sind das zwei verschiedene Sachen. (Interviewerin: Mhm (bejahend)) Was&was erwart ich ihn (Interviewerin: Mhm (bejahend)), da sind wir ganz stark in diesem psychologischen Bereich drin. Wenn ich dem Patienten sage, ich kann wieder ähm einer Prothese die Möglichkeit geben, dass du fühlst (Interviewerin: Mhm (bejahend)), dann heißt das für den, er will fühlen können. Der will nicht sehen, ob’s da summt oder sonst was (Interviewerin: Mhm (bejahend)) und dann stell ich mir die Frage, was für ’n Mehrgewinn hat er dadurch. (Interviewerin: Mhm (bejahend)) Es sei denn irgendjemand in der Runde sagt, er hat den und den Mehrgewinn oder den und den Nutzen, ich seh‘ ihn nur momentan nicht? #00:56:10-7#

Interviewerin: Ja, sagt jemand, dass es ‘n Nutzen gibt, dass es irgendeinen Mehrgewinn dadurch gibt? #00:56:16-7#

Teilnehmerin 36: Also ich denk manchmal beim Kochen (dann?), ‘ne? Also das ist ja sowieso so schwierig, (Interviewerin: Mhm (bejahend) so meinetwegen die Überlegung ich hab jetzt irgendwie ‘n Topf mit Nudelwasser, (Interviewerin: Mhm (bejahend)) will das abgießen, äh da sagt mir jetzt aber Kollegen wenn’s ‘n Silikonhandschuh ist, ja, dann ist das kein Problem, wenn der äh, wenn die Prothesenhand da irgendwie an den Topf rankommt (Teilnehmerin 38: Mhm (bejahend)). Jetzt weiß ich nicht wie das mit&mit andern Handschuhen ist? #00:56:41-1#

Teilnehmerin 38: Die PVC-Handschuhe die schmelzen. #00:56:39-0#

Teilnehmerin 36: Die&die schmelzen. Also da an so ‘ner Stelle wär’s eben so im&im&im Küchen, im Kochbereich, könnte das hilfreich sein, ja (Interviewerin: Mhm (bejahend)). Wenn das zu heiß wird und dann im Grunde genommen gibt’s so ’n&so ’n, wie so ’n Warnpiepen (Interviewerin: Mhm (bejahend)) (Teilnehmerin 38: Mhm (bejahend)). Ähm- #00:56:56-8#

Teilnehmer 39: Dann wär’s aber schon zu spät oder? (Teilnehmer 37: (...??) Dann ist er äh schon geschmolzen (lacht). #00:57:01-5#

Teilnehmerin 36: Ja. #00:57:04-1#

Teilnehmerin 38: Obwohl das ist vielleicht, (glaube ja?) schwierig da wieder umzusetzen. (Teilnehmer 39: Ja.) Das wäre dann ja, dass man wieder dem Anwender das Bewusstsein schaffen muss, wo sind die Grenzen denn. (Teilnehmerin 36: Mhm (bejahend)) (Teilnehmer 39: Ja.) (Interviewerin: Mhm (bejahend)) Wäre manchmal praktisch, ja (lachend). #00:57:17-8#

Teilnehmerin 36: Letztendlich ist auch nicht dramatisch, ja. Es ist das Hilfsmittel (Interviewerin: Mhm (bejahend)), ist ja nicht wie bei einem (Interviewerin: Ja.)

Querschnittgelähmten, der dann ‘ne Wunde hat (Interviewerin: Ja.), weil er das nicht spürt, dass es heiß ist und er kommt (Interviewerin: Mhm (bejahend)) an die Heizung oder so. (Interviewerin: Mhm (bejahend)) Ähm, ja. #00:57:31-1#

Teilnehmer 39: Bei der Frage ist ja wahrscheinlich eher ‘n Grundgedanke? (Teilnehmerin 36: (lacht)) Ja, wieso, wir können ja mal die Frage in die andere Richtung stellen (lacht). Ähm, was denken Sie denn, dass es ähm, dass es demjenigen bringt, oder was ist&was ist der Grundgedanke dahinter? #00:57:43-4#

Interviewerin: Ähm so ’n bisschen ist na-&also wird natürlich von vielen Leuten die&die Prothesen tragen schon rückgemeldet, dass&dass das was am meisten fehlt daran, dass es den Arm nicht mehr gibt, oder was viel fehlt, dass es halt irgendein Feedback&also dass man halt, weiß nicht, dass wenn ich irgendwas berühr, dass ich halt weiß, was das ist. Dass ich nicht immer hingucken muss. Und natürlich denkt man darüber nach, wie sowas technisch möglich wäre (Teilnehmer 39: Mhm (bejahend)), wie man sowas lösen könnte. Ähm aber da gehen eben die Meinungen sehr auseinander, also deshalb so die Frage, um mal so ’n bisschen auszutesten, wie vielleicht auch eben wie ’n Techniker darauf guckt, wie ’n Therapeut darauf guckt, verglichen damit, wie die&die Menschen, die die Prothesen dann tragen, wie das für die ist. Weil&weil da die Meinungen ganz weit auseinandergehen. Eben von: „Das ist irgendwas, was noch störend und ablenkend ist, wenn ich da noch irgendein Ding hab, was&was irgendwie ‘ne Rückmeldung gibt.“, bis zu: „Naja, aber ich&ich bekomm halt ‘n Feedback ich muss irgendwo nicht hingucken oder ich hab‘ halt irgendwie nochmal mehr Sicherheit bei dem, wie ich die benutz.“. (Teilnehmer 39: Mhm (bejahend)) Deshalb halt einfach so ’n bisschen die Frage wie Sie da drauf gucken, weil wenn&wenn wir mal von der anderen Seite gucken, Sie haben vorher gesagt naja, wenn mich da noch irgendwas vibriert oder drückt oder sonst was, bin ich eben abgelenkt davon. Wenn&das heißt, die Prothese müsste das vielleicht von alleine können, dass sie&dass sie eben irgend ‘ne Intelligenz hat und versteht wie fest drück ich zu, wann mach ich irgendwas kaputt. Wär‘ das dann irgendwas, was Sinn machen würde? #00:59:03-4#

Teilnehmer 39: Es wäre (höchstens?) so, wenn die äh Prothese erkennen würde (Interviewerin: Mhm (bejahend)), wobei, wie gesagt, den Patienten hab ich bei uns noch nicht gesehen (Interviewerin: Mhm (bejahend)), bin ich wieder bei diesem Werbevideo von (Name eines Prothesenherstellers aus Datenschutzgründen ausgelassen) ähm, klar, wäre es&der der da sein Portemonnaie rauszieht, wenn die Prothese dann erkennt, dass es das Portemonnaie in der Hand hat und selber zugreifen kann (Interviewerin: Mhm (bejahend)), dann wäre ’s sicherlich ‘ne Erleichterung (Interviewerin: Mhm (bejahend)). Nur ich stell mir grade die Frage wie viele unserer Patienten (Interviewerin: Mhm (bejahend)), weiß nicht ob ihr so jemanden habt, der sie so nutzt oder so voll nutzen kann. Ähm die nutzen sie schon alle sehr bewusst (Interviewerin: Mhm (bejahend)) und es wird keiner mit ‘ner Prothesenhand, weiß ich, in&in ‘ne Box fassen, wo man was fühlen muss. Dass sie mir jetzt sagt: „Ok, da ist was Weiches drin, oder was Hartes.“. Das wird nicht passieren. Das wäre nur wirklich ‘n-&‘ne Erleichterung, vielleicht, dass&dass würd‘ ich gar nicht auf ’s Fühlen schieben, da müsste man dann vielleicht schauen, welche Sachen macht der (Interviewerin: Mhm (bejahend)) im Alltag, im Beruf, im&in der Freizeit, wie auch immer. Und wo macht es vielleicht Sinn, eine Technik in die Prothese einzubringen, bei bestimmten Bewegungen, Handlungen, wo dann ‘ne Unterstützung (Interviewerin: Mhm (bejahend)), so würd ich’s eher sehen, ähm ja,

(kurze Pause) möglich ist. Aber wie gesagt, ich hab‘ jetzt keine Beispiele. Da müsste man dann tatsächlich wahrscheinlich mal forschen, wo macht es Sinn, oder macht es keinen Sinn. (Interviewerin: Mhm (bejahend)) #01:00:20-1#

Teilnehmerin 36: Ähm ich hab‘ noch ’n konkretes Bespiel im Kinderbereich (Interviewerin: Mhm (bejahend)). Ähm&äh:: so ’ne Situation, da gibt’s vielleicht Nachwuchs in der Familie (Interviewerin: Mhm (bejahend)) und dann ähm besteht die Frage und auch irgendwie die Sorge, dass (Interviewerin: Mhm (bejahend)) jetzt das ältere Geschwisterkind mit der Prothese das die Dosierung nicht hinkriegt (Interviewerin: Mhm (bejahend)) und das Kind mit diesem Greifen, dieses Baby mit dem Greifen irgendwie verletzt. (Interviewerin: Ja.) Also wenn man da bei der Kinderhand so ’ne, wie so ’ne Sensibilität irgendwie hätte, nach dem Motto, dass da spürt die Prothese, das ist ein (Interviewerin: Mhm (bejahend))&ein&ein anderer Körperkontakt und wird dann blockiert (Interviewerin: Ja.). Oder auch im Spiel untereinander zwischen den Kindern. (Interviewerin: Mhm (bejahend)) Also die Frage steht immer wieder im Raum und ‘n bisschen Unsicherheit find ich ist da&hängt da auch dran an dem Thema. #01:01:08-9#

Interviewerin: Ok, das heißt also einerseits eben da im Umgang mit anderen, was wir auch vorher hatten mit ich geb‘ die Hand und das drückt zu (Teilnehmerin 36: Mhm (bejahend)), dann vielleicht beim Kochen, damit die Prothese nicht wegschmilzt ähm ja, ok. Äh hatt‘ ich noch irgendwas? (kurze Pause) Nee. Ähm. (kurze Pause) Nee, ok. Dann würd‘ ich das erstmal so stehen lassen. Dann sind wir schon bei der letzten Frage angekommen. Ähm und zwar würd‘ ich gerne, also ich weiß, das haben Sie vorher auch nochmal gesagt, es doppeln sich einige Antworten aber das ist danach bei der Auswertung eigentlich ganz gut, wenn man&wenn man halt manchmal Sachen hat, die eben halt nochmal klar formuliert und klar definiert sind und man die da einfach rausziehen kann. Und zwar würd‘ ich ähm, dass wir gerne jetzt einmal zusammen-äh-tragen was man an den Prothesen, die jetzt gerade auf ’m Markt sind verbessern müsste, damit&oder halt wo Punkte sind, wo Sie sagen, wenn Sie da irgendwie mitmischen dürften oder mitbestimmen dürften, was Sie verbessern würden, was man verändern würde, damit die eben noch besser funktioniert oder ähm damit Erwartungen von Patienten einfach noch besser erfüllt werden könne? #01:02:17-1#

Teilnehmerin 38: Also wir waren letztens auf diesem Expertentreff von (Name eines Prothesenherstellers aus Datenschutzgründen ausgelassen) (Interviewerin: Mhm (bejahend)) und die haben ja jetzt die mh Bebionic-Hand aufgekauft (Interviewerin: Mhm (bejahend)) und da war jetzt die Frage auch irgendwie was könnte man eigentlich machen, was wäre das beste Versorgungsbeispiel für einen. Und im Grunde genommen sind wir da auf den Kompromiss gekommen, dass es nicht möglich ist irgendwo ‘ne Hand zu bauen, die sowohl dieses extrem Robuste hat, (Interviewerin: Mhm (bejahend)) also auch diese viele feine Funktionen. Dass man im Grunde genommen darauf hinauslaufen muss, immer diese Wechselversorgung zu haben. Dass es einmal zum Beispiel den Greifer oder die äh, die normale Myo(Name eines Prothesenherstellers aus Datenschutzgründen ausgelassen)-Hand gibt und einmal die Bebionic-Hand, um eben auch grade im Arbeits- und im Essensbereich miteinander wechseln zu können (Interviewerin: Mhm (bejahend)). Weil auch aus hygienischen Gründen, also&und das ist aber nur im jetzigen Zeitpunkt (Interviewerin: Mhm (bejahend)) sehr schwer den Krankenkassen s-&zu rechtfertigen (Interviewerin: Mhm (bejahend)), also die wollen nicht zwei Prothesen

bezahlen (Interviewerin: Ja.) Wobei man dann ja wieder den Service und Leihhand (Interviewerin: Mhm (bejahend)) ‘ne&‘ne, geht alles so weiter diese ganze Geschichte (Interviewerin: Mhm (bejahend)), aber im Grunde genommen diese Kombination aus robust und Funktionsfähigkeit, das ist sehr sehr schwer (Interviewerin: Mhm (bejahend)). Also das müsste ‘ne perfekte Hand haben. (Interviewerin: Mhm (bejahend), ja.) #01:03:27-6#

Interviewerin: Das heißt, was wir vorher hatten, dass es gar nicht so viele Funktionen haben muss, sondern vor allem eben robust sein muss und die wenigen dann kann. Wenn sie aber robust wäre und die ganzen Funktionen könnte, würde man die Funktionen dann aber trotzdem gerne annehmen? #01:03:40-8#

Teilnehmerin 38: Ja. Also was heißt Sie brauchen nicht viele Funktionen haben. Also wie gesagt, es ist gut, wenn Sie verschiedenen Griffe (Interviewerin: Ja.) hat (Interviewerin: Mhm (bejahend)), einfach weil ähm man ja irgendwie mit den Gegenständen umgehen muss. Also man kann nicht alles mit diesem Dreipunktgriff zum Beispiel machen (Interviewerin: Ja.). Weil sonst könnte man ja jedem einfach die Myo(Name eines Prothesenherstellers aus Datenschutzgründen ausgelassen)-Hand geben und sagen die ist robust, jetzt werd‘ glücklich damit (Interviewerin: Mhm (bejahend)), sondern es ist ja schon, die andre Hand bietet ja auch ihre Vorteile (Interviewerin: Ja.) einfach, weil man eben Gegenstände ganz anders greifen und halten kann. (Interviewerin: Mhm (bejahend)) Und ja. Aber ich bin der Meinung, dass eine Hand nicht unbedingt diesen Mausklick braucht oder so (Interviewerin: Mhm (bejahend)). Weil jetzt jemand der doppelseitig amputiert ist, ja für den ist das vielleicht ganz praktisch und toll (Interviewerin: Mhm (bejahend)), aber was brauch ich für andere diesen Griff? Die werden auch sagen, warum soll ich mir das mit der Prothese viel umständlicher machen, ich nehm‘ die Maus einfach in die andere Hand. (Interviewerin: Ja, ja. Mhm (bejahend)) Also deswegen. Das mein ich mit diesem: „Sie brauchen nicht übermäßig viel Funktion.“. #01:04:34-3#

Interviewerin: Mhm (bejahend) #01:04:35-5#

Teilnehmerin 36: Also idealerweise, ja jetzt bin ich nicht Technikerin, es&aber wenn ich jetzt mir was wünschen darf (Interviewerin: Mhm (bejahend)), ja dann hätte ich den App, wo ich sagen würde ok, jetzt geh ich in den, weiß ich nicht, ähm Büro-äh-bereich rein (Interviewerin: Ja.), bin irgendwie feinmotorisch, wenig Kraft, oder jetzt bin ich im Küchenapp (Interviewerin: Mhm (bejahend)), in der&in der Küchenapp, äh in ‘nem Küchenmodus, so rum, oder in dem Kraft/Handwerk/Gartenmodus (Interviewerin: Ja.), also so dieses, wenn man dazwischen wechseln könnte (Interviewerin: Mhm (bejahend)). Ähm und vielleicht auch sogar, dass daran verbunden sind eben die verschiedenen äh Griffoptionen. Man kann ja jetzt schon verschiedene Level ansteuern (Interviewerin: Mhm (bejahend)). Aber man braucht ja je nachdem in welchem Kontext man arbeitet, eben eigentlich relativ wenig. Die sind aber unterschiedlich zum Teil (Interviewerin: Mhm (bejahend)). #01:05:33-2#

Teilnehmerin 38: Als quasi so wie Touchbionics das mit den grip chips da gemacht hat, (Teilnehmerin 36: Ja:.) dass man verschiedene Griffe raufládt (Interviewerin: Mhm (bejahend)) und dann in jedem Bereich dann einfach die Hand einmal drüber hält und die Gr-&die Griffe reingeladen werden wieder. #01:05:43-3#

Teilnehmerin 36: Äh genau, dass is‘ so ’n erster Ausblick (Teilnehmerin 38: Mhm (bejahend)) dahin, ja. #01:05:49-0#

Teilnehmer 39: Der Vorteil ist ja für ’n Therapeuten, dass was du grade gesagt hast, eigentlich se-&suchen wir grade die Eierlegende Wollmilchsau. Ähm die Hand die robust ist, (Interviewerin: Mhm (bejahend)) die alles kann und die ich dann ähm, App find ich gar nicht so schlecht, ähm eben in einer Form ähm anpassen kann, auf die Bedürfnisse jeweiligen Patienten. (Interviewerin: Mhm (bejahend)) Die sind ja mannigfaltig wie wir schon gesehen haben, aber wenn’s ‘ne Möglichkeit gibt ein Produkt zu haben, was zwar alle Funktionen kann (Interviewerin: Mhm (bejahend)), ich aber die Möglichkeit habe, Funktionen über einfache Mittel oder eben auch der Patient selber, rauszuschalten, dann hab ich zum einen die Möglichkeit des Trainingseffektes vom Kleinen zum Großen (Interviewerin: Mhm (bejahend)) und ich hab die Möglichkeit verschiedenste Patientengruppen unter Umständen damit zu versorgen und dann im Idealfall mich auf verschiedene Tages-ähm und auch zu verschiedenen Tageszeiten, verschiedenen Tätigkeiten (Interviewerin: Mhm (bejahend)) das ganz anzupassen, aber- #01:06:37-8#

Teilnehmerin 38: Das find ich auch wichtig von der Programmierung her (Teilnehmer 39: Genau.). Dass man irgendwie so ’n Grundmuster hat und wenn man möchte, bestimmte Griffe rausnehmen und nicht immer dieses feste Grundmuster nur haben (Interviewerin: Mhm (bejahend)), also das ist zum Beispiel bei der Vincent-Hand so, da kann man manche Dinge nicht rausnehmen, das ist aber am Anfang einfach zu viel für den Patienten, (Interviewerin: Mhm (bejahend)) also und was wichtig ist, dass die, wenn es verschiedene Griffe sind, die schnell anzusteuern sind, dass man nicht immer über einen ähm einen Griff in die anderen schalten muss (Interviewerin: Mhm (bejahend)). Also ich weiß nicht, ob das Vincent Programmierungssystem bekannt ist, auf jeden Fall da muss man immer über ’n langes Öffnen-Signal in die offene Hand gehen (Interviewerin: Mhm (bejahend)) und von da aus muss man zum Beispiel ein langes Schließen-Signal geben, um dann wieder in den andern Griff zu kommen. (Interviewerin: Mhm (bejahend)) Das dauert viel zu lange. (Interviewerin: Mhm (bejahend)) Das ist&technisch find ich das&haben sie sich das toll überlegt (lacht), ja, aber äh praktisch ist das, wenn da jemand dann erstmal so, ich sag jetzt mal 30 Sekunden braucht, bis er in dem richtigen Griff ist um das Glas zu greifen, das&das dauert zu lange. #01:07:30-7#

Interviewerin: Ja. Ok also das, dass&dass das einfach die Hand schnell reagieren kann, dass ich schnell (Teilnehmerin 38: Mhm (bejahend)) das machen kann, was ich möchte, dass sie robust ist, dass sie sich aber auch von den Funktionen her einfach daran anpasst, was ich von ihr möchte. Eben ob ich jetzt im Küchenmodus bin oder halt im Büromodus. Gibt’s noch irgendwas, was wichtig wäre? #01:07:48-1#

Teilnehmer 39: Wir reden eigentlich über ’n intuitives Greifen dann (Interviewerin: Ja.). Dass der Patient halt ähm nicht tausend Schritte sich im Kopf überlegen muss. Was muss ich jetzt wie ansteuern, damit die Hand das und das macht (Interviewerin: Mhm (bejahend)), sondern, das wäre eigentlich das&das äh Nonplusultra, dass der intuitiv, ich will das Glas greifen (Interviewerin: Mhm (bejahend)) und dann dementsprechend einfach das Ganze ansteuern kann. (Interviewerin: Mhm (bejahend)) Und dann natürlich, und sind wir bei dem Punkt, den Sie eben schon angesprochen haben, dass dann unsere lieben Krankenkassen auch noch sagen: „Jo, das ist das Produkt was ich auch gerne einmal bezahle, aber dafür hab ich das

All-in-Produkt.“. (Interviewerin: Mhm (bejahend)) Da ist eben die Frage, wie nah kommt man an sowas ran? #01:08:26-7#

Interviewerin: Ja. Ähm mir ist jetzt wieder eingefallen, was ich vorhin noch zu dieser äh Rück-&Rückmeldungs- und Haptiksache sagen wollte und zwar, Sie haben gesagt naja, wer&wer braucht denn das, dass er mit der Prothese da nach hinten in die Hosentasche greift. Wenn wir jetzt mal überlegen, dass jemand eben beide äh Arme weghat, wenn wir dann nochmal darüber nachdenken wie sinnvoll sowas ist mit so ’ner Rückmeldung? Ähm was fällt Ihnen dazu noch ein, also wenn wir- #01:08:53-6#

Teilnehmer 39: Dazu fällt mir spontan ‘n Patient ein, da weiß ich wie lange es gedauert hat, (Interviewerin: Mhm (bejahend)) um überhaupt erstmal ihn ähm, da ging’s erstmal darum, dass er erstmal eine Seite ansteuern konnte (Interviewerin: Mhm (bejahend)), bevor wir über die zweite geredet haben (Interviewerin: Mhm (bejahend)) und damit hat er erstmal die ersten Sachen ähm gemacht und war dann tatsächlich froh, dass er nachher irgendwann ‘n&‘n&‘n Wasserglas erstmal greifen konnte. (Interviewerin: Mhm (bejahend)) Und ähm ja, klar wär’s für denjenigen ‘ne Erleichterung, aber da ja. Ähm das sind&das ist, (Leute?) das sind ja Projekte die sind&die dauern ewig lange (Interviewerin: Ja.) ehe man da überhaupt-. Wir haben halt sehr viele äh Patienten, hier über einen Arzt (...?) hier auch etliche gelandet, aus Kriegsgebieten gehabt und da ging’s halt einfach nur, da war zwar viel Geld vorhanden, aber ähm da hat man dann einen Zeitraum, wie’s bei (Name eines Prothesenherstellers aus Datenschutzgründen ausgelassen) auch häufig ist, bekommen, die die in Deutschland waren und in der Zeit sollte dann möglichst das All-in-Paket geschnürt werden. (Interviewerin: Mhm (bejahend)) Das heißt er sollte seine Prothese kriegen, der sollte möglichst viel können derjenige und am Ende wird er mit der Prothese mit ‘nem relativ guten Ergebnis in dem Fall sogar in Flieger gesetzt und keiner hat sich Gedanken darüber gemacht in, ich sag jetzt nicht wo er hingeflogen ist, ich sag jetzt mal in Timbuktu, wie denn da diese Prothese überhaupt und von wem, weitergepflegt wird und ähm ob überhaupt mal jemand nachschaut und ähm im Idealfall dürfen die dann nach ‘nem Jahr oder wann mal wieder nach Deutschland kommen und dann sieht man: Hat er sie überhaupt getragen, funktioniert der Akku überhaupt noch und ähm&ähm da reden wir um ganz andere Sachen dann auch noch (Interviewerin: Ja.). Also ja, das wäre auch zum Beispiel was, ähm wie wird das Ding gewartet? Toll wenn das an Technik alles möglich ist, aber ähm wenn er dann Zuhause ist, wie oft muss der zum Techniker, wie ist das weltweit, wir sind in ‘ner globalen Welt, zu sehen ähm der ist halt eben nicht unbedingt jetzt ähm hier gerade in der Nähe von Hannover (Interviewerin: Mhm (bejahend)), der sitzt vielleicht wo ganz anders, das sind auch so Sachen die&die denk ich mit berücksichtigt werden müssen. Ähm ja, weil sonst sagt der Patient, wenn das ewig wartungsintensiv ist und er alle paar Wochen wieder beim Techniker sitzen muss, da hab ich auch kein Bock mehr drauf. (Interviewerin: Mhm (bejahend)) Der hat nämlich vielleicht auch mal ‘n Leben drumherum. (Interviewerin: Mhm (bejahend)) #01:11:07-7#

Teilnehmerin 36: Also ein Aspekt der meiner Meinung nach noch nicht angesprochen ist (Interviewerin: Mhm (bejahend)) und der auch deutlich wird bei der beid-äh-seitig betroffenen (Interviewerin: Mhm (bejahend)) Patientensituation, dass ist das ähm Anziehen der Prothese (Interviewerin: Mhm (bejahend)). Ähm das ist f-&für Kinder ‘n großes Problem (Interviewerin: Mhm (bejahend)), je nach

Sch-&Schaftlänge, je nachdem äh wie so die Technik des Anziehens ist, aber das betrifft jetzt nicht direkt die&die Technik (Interviewerin: Mhm (bejahend)), das Können des&der&der Prothese aber ähm genau, im Grunde genommen diesen Part dazwischen, ja? Wie bekomm ich denn jetzt gut und sicher äh die Prothese überhaupt an den Arm (Interviewerin: Mhm (bejahend)) und das ist ‘ne riesen Baustelle bei&bei Beidhändigen sowieso, bei Kindern aber auch (Interviewerin: Mhm (bejahend)) ähm passt hier nicht ganz rein, aber ist auf jeden Fall (Interviewerin: Mhm (bejahend)) ähm ‘n großes Thema. #01:12:02-5#

Protokollant: Passt schon rein. #01:12:03-0#

Interviewerin: Ja, ja, passt rein! Also das ich einfach, dass sie einfach anzuziehen ist, aber eben dann trotzdem ja sicher halten muss, ‘ne? #01:12:11-5#

Teilnehmerin 36: Genau, genau. #01:12:14-7#

Interviewerin: Mhm (bejahend), ja. #01:12:13-1#

Teilnehmerin 36: Und das ist eben im Gegensatz zur Prothetik untere Extremität, da stehen die ja mit ihrem Gewicht drauf, auch da hab ich (Interviewerin: Mhm (bejahend)), zur Not kann ich dann in der Schwungphase mal die Prothese verlieren (Interviewerin: Mhm (bejahend)) ja, was irgendwie ‘n Alptraum ist (Interviewerin: Mhm (bejahend)), aber der Arm der hängt immer auch nach unten, ja und (Interviewerin: Ja, klar.) also wenn ich irgendwie laufe, also ähm, ja. #01:12:33-1#

Teilnehmerin 38: Geringes Gewicht wär‘ auch toll, wenn wir noch über die perfekte Prothese sprechen (Interviewerin: Mhm (bejahend)). Möglichst wenig wiegen, ganz kleine Akkus und ja. #01:12:43-0#

Teilnehmer 39: (...???) (lachend) #01:12:51-4#

Teilnehmerin 38: Ganz kleine Elektroden, alles mini (lachend). Mini leicht, aber trotzdem super robust. #01:12:58-5#

Interviewerin: Gibt’s noch irgendwas, was wir da jetzt noch nicht gesammelt haben was da noch wichtig wäre? #01:13:04-9#

Teilnehmerin 36: Also im Hinblick auf das Anziehen und den Schaft ähm in der Erfahrungen die ich ähm ‘n bisschen mühsam gemacht hab (Interviewerin: Mhm (bejahend)), dass ähm ist das Thema, dass man eben nicht ‘ne Prothese verordnet, die angepasst wird, damit geübt wird und dann ist der Patient fertig, sondern dass es wichtig ist, dass der Patient weiß ich bin&dass viele Kontakte mit der (Interviewerin: Mhm (bejahend)) ähm mit der Technik, vielleicht ‘n bisschen weniger dann mit der&mit der Therapie (Interviewerin: Mhm (bejahend)), aber das bleibt auch so, ja? Und bei jeder Gewichtsschwankung, ja, (Interviewerin: Mhm (bejahend)) müssen die wieder antanzen und da kann ich nicht wie bei der unteren Extremität ‘nen Socken zusätzlich anziehen oder weglassen, sondern dann haben die keinen Kontakt mehr zu den Elektroden (Interviewerin: Mhm (bejahend)), dann funktioniert das nicht (Interviewerin: Mhm (bejahend), ja.). Also das, einfach dieses Bewusstsein, ohne dass der Patient jetzt schuld hat oder ‘n Fehler gemacht hat (Interviewerin: Mhm (bejahend)), man ist ‘n kleines bisschen verheiratet und muss sich immer wieder

sehen, immer wieder anpassen, immer wieder neue Handschuhe, mal ‘ne Wartung, das ist ‘n ganz langer Prozess und das&so ’n bisschen wie mit den Zahnspangenkindern (Interviewerin: Mhm (bejahend)), ja? Die müssen auch regelmäßig hin ähm das gehört dann einfach zum Leben dazu. (Interviewerin: Mhm (bejahend), ja.) #01:14:21-5#

Interviewerin: Und trotzdem wie wir dann vorher gesagt haben, wär’s dann eben ‘ne Erleichterung, wenn sowas wie Handschuh anziehen oder Robustheit halt gegeben wäre (Teilnehmerin 36: Genau.) und man eben nicht so oft antanzen muss, ‘ne? (Teilnehmerin 36: Genau.) Ja, mhm (bejahend). Ok ähm dann würd‘ ich jetzt gerne zum Abschluss einfach nochmal ganz kurz sammeln. Also wir haben Vor- und Nachteile so ’n bisschen die Probleme, wenn man jetzt jemanden rehabilitiert was da auftritt, dann Feedback von den Patienten. Äh gibt’s noch irgendwas, was vielleicht nicht gefragt wurde, was aber ‘n wichtiger Punkt ist, der noch angesprochen werden sollte oder ähm gibt’s irgendwas, was, von den Dingen die wir jetzt besprochen haben, was besonders wichtig ist, was man nochmal oder was Sie gerne nochmal unterstreichen wollen, irgendwie mit auf ’n Weg geben wollen, äh was so im Kopf bleiben sollte? #01:15:05-8#

Teilnehmerin 38: Vielleicht einfach, dass es unheimlich schwierig ist eine Hand oder eine Prothese oder ein Prothesensystem (Interviewerin: Mhm (bejahend)) für alle Patienten zu machen, weil es dann doch sehr individuell ist (Interviewerin: Ja.) und jeder unterschiedliche Ansprüche hat. Also da hab ich nämlich grade nochmal an das Thema Akkuleistung auch gedacht (Interviewerin: Mhm (bejahend)), weil das ist ja, manche benutzen ihre Prothese vielleicht zwar haben sie acht Stunden am Tag an (Interviewerin: Mhm (bejahend)) aber haben sie vielleicht nur drei Stunden an und brauchen sie dann (Interviewerin: Mhm (bejahend)) und andere haben sie wirklich den ganzen Tag an und den ganzen Tag auch zum Greifen wird sie aktiv benutzt, wo man mit ‘ner normalen Akkuleistung von, ich glaub die (Name eines Prothesenherstellers aus Datenschutzgründen ausgelassen)-Akkus haben 1800 Milliampere oder so, also überhaupt nicht ausreichend (Interviewerin: Mhm (bejahend)). Und das ist halt, da wird’s ja schon wieder schwierig was Allgemeines zu entwickeln (Interviewerin: Ja, mhm (bejahend).). Sondern dass es doch immer sehr individuell ist, also müsste man im Grunde genommen ein System haben, was man immer auf die verschiedenen Bedürfnisse anpassen kann, wo man immer aufstocken kann (Interviewerin: Mhm (bejahend)) oder was wegnehmen kann. Also wie bei den Griffen zum Beispiel, dass man da ‘n Grundsystem hat, aber auch was dazusetzen kann. Hm, schwierig. #01:16:07-2#

Interviewerin: Also so ’n bisschen einfach ‘ne gewisse Individualität, die halt an die Person und an das wofür die Prothese dann gedacht ist und wofür die benutzt wird halt angepasst ist? #01:16:14-4#

Teilnehmerin 38: Ja. #01:16:15-2#

Interviewerin: Mhm (bejahend) #01:16:15-2#

Teilnehmer 39: Ich denke schon, dass das&ich find das gar nicht so abwegig was die (Name von Teilnehmerin 36 aus Datenschutzgründen ausgelassen) gesagt hat. Ähm die Technik geht weiter (Interviewerin: Mhm (bejahend)). Ähm wir leben halt in ‘nem Zeitalter der Apps und sonstigen Geschichten und ich denk das ist auch bei

den Patienten nicht weit weg. Das es in diese Richtung geht und da sind wir auch bei diesen&bei den Akkugeschichten immer im selben Bereich. Wenn&wenn's 'n Büroarbeitsplatz ist, heutzutage (Interviewerin: Mhm (bejahend)) packen Sie Ihr Handy auf, weiß ich, auf 'ne magnetische Ladeschale oder sonstige Geschichten (Interviewerin: Mhm (bejahend)) wenn 'se an der Arbeit sind ähm das man da auch die&in die Richtung halt einfach mit weiterdenkt (Interviewerin: Mhm (bejahend)). Neben der Funktionalität. Aber ich find's halt ähm sehr wichtig halt eben auch, dass man eben ähm mehrere Möglichkeiten da vielleicht vereinen kann und eben auch einfach für den Patienten das Ganze zu (...?) (Interviewerin: Mhm (bejahend), ja.) #01:17:01-7#

Teilnehmer 37: Ja an der unteren Extremität gibt es das ja schon, 'ne? Beim Genium (Interviewerin: Mhm (bejahend)) (...??) da kann der Patient seine Elektroden selber verstellen, die Modi wechseln (Interviewerin: Mhm (bejahend)) und da gibt's ja auch äh individuelle Moduseinstellungen (Interviewerin: Mhm (bejahend)) und (...??) (Interviewerin: Ja.) über 's Handy über 'ne App drauf zugegriffen werden kann. #01:17:23-9#

Interviewerin: Mhm (bejahend), also wirklich diese individuelle Anpassung an das, was derjenige braucht? #01:17:28-1#

Teilnehmer 37: Genau. #01:17:27-2#

Interviewerin: Mhm (bejahend) #01:17:29-1#

Teilnehmer 37: (Man hat aber?) vielleicht wenn, ähm 'n Prothesensystem, obere Extremität auch ähm mit der zunehmenden Aktivität des Patienten, mithalten kann. (Interviewerin: Ja.) Wo die, ich sag mal jetzt, äh Kenevo-Kniegelenk, untere Extremität, die haben ja auch drei verschiedene (Interviewerin: Mhm (bejahend)) Aktivitätsmodi, wo man dann von A anfängt und dann irgendwann bei B Plus und C landet (Interviewerin: Mhm (bejahend), ja.) in 'ner freien Schwungphase. (Interviewerin: Mhm (bejahend)) Das man das auf 'ne Hand vielleicht irgendwie ummünzen kann. #01:18:05-6#

Teilnehmer 39: Ist grad aber&grad im Kinderbereich selten. Da hat man's ja häufig, dass dann wirklich ähm, oder haben wir jetzt 'n paar Mal gehabt, war allerdings auch bei Beinprothesen, wo dann einfach die Versorgung schlichtweg (Interviewerin: Mhm (bejahend)) nicht mehr in Ordnung war und nachher der eigentliche Hausarzt sagte: „Komisch, Oberkörper ist schon so lang, Beine sind noch so lang. Irgendwo hat da wohl mal jemand geschlafen, hat nicht ge-&geschaut.“, das heißt der ist, ist halt sowohl physiotherapeutisch als auch orthopädi-&äh von der Orthopädietechnik (Interviewerin: Mhm (bejahend)), ähm weiß nicht wer da geguckt hat, aber als ich den das erste Mal gesehen hab, denjenigen, hab ich gedacht: „Das gibt's doch nicht, sowas muss man doch sehen.“ (Interviewerin: Mhm (bejahend)), dass man da dann eben alle auch ähm ja mit im Boot hat oder eventuell sogar Möglichkeiten hat (und?) sagt: „Hier, da und da muss regelmäßig nachgeguckt werden.“ (Interviewerin: Mhm (bejahend)) oder der Patient dann einfach auch mal 'ne&'ne Rückmeldung (...?) hier: Da und da muss nochmal nachgehakt werden (Interviewerin: Mhm (bejahend)). Das 's bei den Krankenkassen üb-&im Übrigen häufig auch so. Grade im Kinderbereich hab ich da schon Sachen erlebt, wo dann: „Ja, er ist ja jetzt versorgt und dann sind wir ja damit durch.“ und wenn man dann sagt: „Kleinen Moment mal, der Junge ist

grad 25 ähm Sie glauben doch nicht, dass er eine Prothese jetzt bis&ähm bis er 80 ist trägt?“, da sind häufig halt auch einfach ähm ja (Interviewerin: Mhm (bejahend)) Sacharb-&ja Sacharbeiter die vielleicht vorher noch nie damit zu tun haben, die überhaupt nicht nachdenken und denen gar nicht bewusst ist, dass es nicht um eine Prothese geht, sondern dass das Ding eben, ja ein langer Prozess ist und dass da tatsächlich vielleicht gewisse ähm Kontrollen auch vorgegeben werden, die ein-&einzuhalten (Interviewerin: Mhm (bejahend)) sind. Um da einfach den Prozess auch weiterzuführen. #01:19:35-3#

Interviewerin: Mhm (bejahend), wo man einfach mal drüber guckt sitzt die noch richtig, passt das richtig? #01:19:36-1#

Teilnehmer 39: Genau. #01:19:39-1#

Interviewerin: Ja, mhm (bejahend). #01:19:39-9#

Teilnehmer 39: ‘N Auto muss ich ja auch zur Wartung bringen. #01:19:40-6#

Interviewerin: Mhm (bejahend) #01:19:42-4#

Teilnehmerin 36: In dem Rahmen ähm was du sagtest jetzt auch dieses persönlich&dieses persönliche Profil des Patienten eigentlich abholen. Aus meiner Sicht kommt da wirklich zu kurz äh die Anamnese, ja. Also das heißt (Interviewerin: Mhm (bejahend)) im Vorfeld das Patientengespräch ähm aus meiner Sicht müssten die, das gibt es ja auch bei bestimmten (Interviewerin: Mhm (bejahend)) ähm ärztlichen Untersuchungen im Vorfeld wirklich sich die Zeit nehmen, die Patienten ähm und ein&einen Fragebogen ausfüllen. (Interviewerin: Mhm (bejahend)) Also Richtung genau, wie Leben sie, was haben sie für Erwartungen, wie ist das Umfeld, wie ist die Geschichte (Interviewerin: Mhm (bejahend)). Ähm und&und ganz ehrlich, ich mein, wir arbeiten ja relativ interdisziplinär hier (Interviewerin: Mhm (bejahend)) ja, es sind Therapeuten, Techniker in einem Haus (Interviewerin: Mhm (bejahend)), es kommt <u>immer</u> zu kurz, ja? (Interviewerin: Mhm (bejahend)) Es kommt immer zu kurz das reine Gespräch, weil am reinen Gespräch passiert weder technisch noch therapeutisch was, das heißt es fällt&fällt durch. (Interviewerin: Ja.) Ähm und ich glaube da könnte man, unabhängig von all den technischen Möglichkeiten, ja, den Registern die man dann ziehen kann. Aber dieser Baustein der ist irgendwie&wird unterbewertet (Interviewerin: Mhm (bejahend)), also ja. #01:20:55-5#

Interviewerin: Um halt einfach ohne diese ganzen technisch-therapeutischen Aspekte mal mit dem Patienten zu erarbeiten was braucht der eigentlich, wofür wird das eigentlich gedacht sein (Teilnehmerin 36: Genau.) und wie muss das eigentlich an ihn angepasst sein? #01:21:05-5#

Teilnehmerin 36: Genau, damit wir den abholen (Interviewerin: Mhm (bejahend)) und nicht sagen (Interviewerin: Ja.): „Mensch, das geht jetzt alles.“. Äh ich fand’s interessant, dass- #01:21:00-5#

Teilnehmerin 38: Mhm (bejahend), nee das ist&dieser Punkt auch mit dem Feedback, was die Probleme geben kann zum Beispiel (Interviewerin: Mhm (bejahend)), jetzt eben mit ob das jetzt ‘n weicher oder ‘n harter (Interviewerin: Ja.) Gegenstand ist, ich glaub das können wir gar nicht so genau sagen, ob die das

brauchen oder nicht. Das wäre jetzt was, was man wirklich eher die Anwender (Interviewerin: Mhm (bejahend)) fragen kann.) #01:21:21-5#

Teilnehmer 39: (Überlegen?) was will der, (Teilnehmerin 38: Ja.) was will der Patient. #01:21:27-7#

Teilnehmerin 36: Und genauso müsste es, parallel zu dieser (...?)prothese untere Extremität, da bekommen (Interviewerin: Mhm (bejahend)) ja die Patienten ‘n halbes Jahr ‘ne Prothese, bevor die endgültige ähm Versorgung genehmigt wird (Interviewerin: Ja.). Das gibt’s nicht bei der&äh bei den Armprothesen. (Interviewerin: Mhm (bejahend)) Das müsste es genauso geben, ja? Und nicht nur für zwei Wochen ‘ne Leihhand und dann diskutiert man nochmal mit den Herstellern: „Oh, kannst du mir noch ‘ne Woche draufgeben?“. So, dass müsste genauso&vielleicht nicht ‘n halbes Jahr (Interviewerin: Ja.), aber warum gibt’s das nicht? Und die Prothesen sind zum Teil teurer, als die von der unteren Extremität (Interviewerin: Mhm (bejahend)), (Teilnehmer 39: Also-) die Systeme. #01:22:01-2#

Teilnehmer 39: Wir haben Leihprothesen gehabt, wo die Krankenkasse gesagt hat: „Wir machen es nur, wenn eine ähm Leihversorgung stattfindet.“ und die Krankenkasse hat danach ein Video (Teilnehmerin 36: Wie la-&wie lange Leihversorgung?)-. Das&äh das waren&waren zwei, drei Monate jetzt glaub ich. (Teilnehmerin 36: (...?)) Ja, ja jetzt kommt’s ähm und ähm sie wollten dann ein Video haben, dass derjenige das Ganze dann auch ansteuern kann. War in dem Fall auch ein junger Mensch, um die 16 war der glaub ich und das funktionierte auch und dann ging’s halt um die Genehmigung, dass er&dass er die dann komplett genehmigt kriegt. Dann wurde sie ihm aber komplett erst weggenommen, weil Leihgabe zu Ende, dann ging der Prozess bei der Krankenkasse los (Teilnehmerin 36: Mhm (bejahend)), das heißt er hat dann nochmal fast ‘n halbes Jahr gewartet, bis wir zur eigentlichen Versorgung (gingen?). Und das sind so Sachen, das geht überhaupt nicht. Und da ist es mir völlig egal, ob der von Geburt an ähm ‘n Arm weniger hat oder durch ’n traumatisches Ereignis. In dem Fall war’s sogar noch ’n traumatisches Ereignis. Ähm wenn Sie jemandem ‘ne Leihversorgung geben, bin ich voll dabei, dass das&dass das ok ist, zum Ausprobieren, beziehungsweise dass der auch einfach merkt das und das ähm in (...??) geht’s. Aber dann, muss auch ‘ne Versorgung gewährleistet sein, dass der dann ähm ‘n Arm schnellstmöglich bekommt (Interviewerin: Mhm (bejahend)) und nicht, dass dann die Bürokratie in Deutschland losgeht oder bei den Krankenkassen. Wenn ich so jemanden ‘n halbes Jahr, ich hab den erlebt, der war psychisch total am Ende, da brauchten wir gar nicht mit ’m Arm anfangen, da mussten wir erstmal wieder: „Jaja jetzt kriegst du ihn ja auch und alles gut.“, aber das ist&das ist für denjenigen ‘n Martyrium, das geht gar nicht. #01:23:35-8#

Teilnehmer 37: Ja gut, jetzt hat sich aber so ’n bisschen (Interviewerin: (hustet)) (..?) der Rückenwind durch das (..?) #01:23:42-8#

Teilnehmer 39: Klar, inzwischen hat sich da einiges geändert, weil solche Sachen halt- #01:23:43-0#

Teilnehmerin 38: So ist es bei uns auch. (Teilnehmer 39: Ja.) Wir haben vier Wochen Testversorgung und dann kann auch schon mal sein, dass ‘n halbes Jahr nichts mehr danach ist. #01:23:47-8#

Teilnehmer 39: Aber das ist für denjenigen, (Teilnehmerin 38: Schlimm.) geht gar nicht. #01:23:52-8#

Teilnehmer 37: Ja das ist, ‘ne? Aber da muss man sich auch (hinterklemmen?), müssen wir auch ‘n Patienten mit ins Boot nehmen, dass der mal seiner Krankenkasse und Kostenträgern Druck macht. (...?) innerhalb von drei Wochen ‘ne Entscheidung fällen und wenn ‘se ’s nicht getan haben, gilt ’s eigentlich als genehmigt. #01:24:09-1#

Teilnehmerin 38: Wobei die halt grade so im jugendlichen Bereich sich se:hr lange Zeit lassen auch dann mit so, nach dem Motto: „In dem halben Jahr wo wir jetzt quasi schon diskutieren, ob genehmigt oder nicht, sparen wir uns ja schon eine Schafterneuerung, weil es hätte ja sein können, dass derjenige in der Zeit wächst.“, ‘ne? Also das ist ja schon nicht unüberlegt- #01:24:20-6#

Teilnehmer 39: Bei uns ist es auch so, dass Sanitätshaus ist da inzwischen so, die machen das für den, die haben eigene Anwälte und das wird auch so schnell durchgeboxt ähm da fackeln die auch nicht lange ähm aber anders kriegst du’s inzwischen trotzdem nicht hin, also- (Interviewerin: Mhm (bejahend)) #01:24:32-3#

Teilnehmer 37: Eigene Anwälte? Das ist ja interessant. #01:24:40-1#

Teilnehmer 39: Also d-&habt ihr das nicht? #01:24:37-7#

Teilnehmerin 36: Haben wir auch im Hintergrund. #01:24:42-2#

Teilnehmer 39: Ja, also wenn sie’s (...?) sie hauen nicht jedes Mal den Anwalt los. Aber wenn ähm die scheuen sich da auch nicht da dann zu (plagen?), ‘ne? #01:24:49-1#

Interviewerin: Mhm (bejahend) #01:24:50-8#

Teilnehmer 39: Aber es ist traurig, #01:24:54-8#

Interviewerin: Nee, aber trotzdem. Das einfach als Punkt, dass halt sowas wie so ’ne&so ’ne Probeversorgung da sein muss, wo man einfach mal testen kann und dann trotzdem danach halt weiß, dass die Prothese halt kommt, ‘ne? #01:25:01-7#

Teilnehmerin 38: Sowas muss möglich sein, wenn man eine Hand entwickelt, dass man schon beachtet, man muss ‘n Service Center haben, man muss Testhände haben (Interviewerin: Mhm (bejahend)) und nicht wie jetzt ‘ne Firma (Name eines Prothesenherstellers aus Datenschutzgründen ausgelassen): „Ja wir kaufen Bebionic, aber Testhände und Servicehände, ja da müssen ‘se drei Monate drauf warten.“. Ja, das ist doch ‘n Scherz? (Interviewerin: Mhm (bejahend)) Also man muss quasi erstmal die Bandbreite in diese Richtung entwickelt (Interviewerin: Ja.) werden und dann kann man eigentlich erst den Verkauf starten, so. Also- #01:25:22-0#

Teilnehmerin 36: Stimmt, das ’s gut. #01:25:22-5#

Teilnehmer 39: Ich meine, wir müssen ja nichts vormachen und es gibt ja nur noch im Endeffekt, sag ich mal, zwei große Anbieter (Teilnehmerin 38: Ja.), die den Markt aufgekauft haben und ja ich will nicht sagen sie können mehr oder weniger machen was sie wollen (Interviewerin: Mhm (bejahend)), aber es ist einfach so, sie geben einfach die Regeln vor. Das ist das Problem. #01:25:42-2#

Interviewerin: Gibt's noch irgend 'nen anderen Punkt hinzuzufügen oder abschließend zu sagen? (kurze Pause) Ok, dann würd ich sagen, dass wir mit dem Gespräch erstmal durch sind, wir machen die Mikrofone wieder aus.
