# Supplementary material for: What do users and their aiding professionals want from future devices in upper limb prosthetics? A focus group study
Source: PLoS One. 2023 Dec 29;18(12):e0295516. doi: 10.1371/journal.pone.0295516 (PMC10756510; doi:10.1371/journal.pone.0295516)
Supplement: S1 Appendix — (ZIP) [file pone.0295516.s001.zip › FocusGroup_Transcripts/FGP1.pdf]

Interviewerin: Ich würd’ Sie bitten wenn ich gleich die erste Frage stell’, dass da einfach erstmal der Reihe nach geantwortet wird, damit äh sich jeder auch nochmal vorstellen kann, mit Vor - (lachend) und Nachnamen, damit wir einmal die Stimmen mit den Namen äh auf’m:::: Gerät haben ähm::::: (kurze Pause) genau. Und zwar würd’ ich Sie bitten, wenn Sie sich vorstellen, dass Sie als Person die eine Prothese trägt, eine andere Person mit ’ner Armprothese treffen, dass Sie einmal ähm versuchen sich das vorzustellen und erzählen, was Ihnen da als erstes auffällt, auf was Sie da als erstes achten. #00:00:34-7#

(kurze Pause)
#00:00:35-1#

Teilnehmerin 23: Oh Gott. #00:00:36-6#

Interviewerin: (lacht) #00:00:37-8#

Teilnehmerin 23: (unverständlich) (lachend) (…?) Anfang. #00:00:37-6#

Interviewerin: Ja, oder Herr (Name von Teilnehmer 1, aus Datenschutzgründen ausgelassen) (Teilnehmerin 23: Ja.) möchten Sie anfangen? #00:00:40-6#

Teilnehmer 1: Ähm ähm ja eh ich meinetwegen, kann ich machen. #00:00:44-3#

Interviewerin: Mhm (bejahend) #00:00:43-5#

Teilnehmer 1: Mein Name ist (Name aus Datenschutzgründen ausgelassen), ich bin 65 Jahre und ähm komme aus Berlin (Interviewerin: mhm (bejahend)). Ich habe ähm meine ersten Prothesen, myoelektrische Prothesen, im Alter von 16 Jahren hier in Hannover bekommen (Interviewerin: Mhm (bejahend)) in (Name eines Sanitätshauses, aus Datenschutzgründen ausgelassen). Glaube das waren die ersten, wenn überhaupt, und habe seitdem nur diese myoelektrischen Prothesen getragen. (Interviewerin: Mhm (bejahend)) Ähm wenn ich jemand anders sehe, dann äh ist das für mich interessant, wie weit er mit dieser Prothese tatsächlich was machen-. Ich sehe-, ich hab‘ viele Videos gesehen wo Prothesenträger vorgestellt werden und ihr Können sozusagen zeigen, aber da ist es immer so, dass sozusagen bei Einhändern, bei einseitig versorgten Personen, dass die eben halt sehr viel mit der gesunden Hand machen und die rechte Hand irgendwie (Interviewerin: Mhm (bejahend)) nur halt Assistenzfunktion hat. Bei mir isses so, ich bin ja beidseitig versorgt (Interviewerin: Ja.) und ich bin (unverständlich) auf die Handfunktionalität angewiesen. (Interviewerin: Mhm (bejahend)) Und das ist für mich eben halt interessant, wie jemand ähm (…??). #00:01:43-7#

Interviewerin: Ja (kurze Pause), dass Sie einfach vergleichen was die andere Person kann und, und was sie, welche Funktion die damit ausüben kann? #00:01:49-2#

Teilnehmer 1: Genau. #00:01:48-5#

Interviewerin: Mhm. #00:01:51-1#

Teilnehmer 1: Bei mir kommt jetzt noch dazu, dass ich Parkinson erkrankt bin (Interviewerin: Ja.) und dass diese Krankheit eben halt Muskelströme auch verändert (Interviewerin: Mhm (bejahend)) und dadurch eben es zu äh einseitig Beeinträchtigungen kommt die mich noch weiter behindern (…??). #00:02:02-2#

Interviewerin: Ja, ok vielen Dank. Herr (Name des Teilnehmers 8, aus Datenschutzgründen ausgelassen)? #00:02:10-1#

Teilnehmer 8: Mh tatsächlich also mein Name ist (Name aus Datenschutzgründen

ausgelassen), vom Alter her bin ich wohl achtzig, bin neun, also 36 geboren, 1936 geboren, 1936, und (kurze Pause) hab diese:::. Beschädigung durch Kriegseinwirkung, noch während der Nazizeit, also während der, während des, Zweiten Weltkriegs erlebt, als Kind (kurze Pause). Ja, am Anfang hatte ich natürlich diese:: einfache Holzhand… #00:02:33-8#

Interviewerin: Mhm (bejahend), ganz kurz, dazu wie die Versorgung ist, dazu kommen wir gleich noch (Teilnehmer 8: Kommen wir gleich noch, gut.). Genau. Erstmal einfach: Wenn, wenn Sie ’ne andre Person sehen, was fällt da auf, auf was achten Sie? #00:02:41-7#

Teilnehmer 8: Was fällt mir auf? Eigentlich achte ich nicht drauf, mir ist es (Interviewerin: Mhm (bejahend)) nur heute aufgefallen weil wir halt speziell in der Runde sind (kurze Pause) und weil seine Prothese (zeigt auf Teilnehmer 1) so elegant und gut aussah im Vergleich zu meiner. Und äh deswegen ist es mir aufgefallen (Interviewerin: Mhm (bejahend)) ansonsten achte ich auf sowas nicht. #00:03:07-0#

Interviewerin: Ok, ja. Und Sie, Frau (Name der Teilnehmerin 23, aus Datenschutzgründen ausgelassen) (lachend)? #00:03:11-1#

Teilnehmerin 23: Ja, also::: ich heiße (Name aus Datenschutzgründen ausgelassen) ich bin 38 Jahre alt, ich komme aus (Name des Wohnorts aus Datenschutzgründen ausgelassen) u:::nd äh seit Geburt bin ich behindert. (kurze Pause) Joa::h (seufzt) und ja (kurze Pause). Ähm (kurze Pause) ich muss jetzt lernen mit diese, mit der Armprothese zu klarkommen. (Interviewerin: Ja..) Weil seit ähm, seit öh, seit ein Jahr hab‘ ich die Armprothese bekommen und bin ich die ganze Zeit am (seufzt) Üben. (Interviewerin + Teilnehmer 8: Mhm (bejahend)). #00:03:51-4#

Interviewerin: Und wenn Sie jetzt, wie hier draußen in so ’ne Runde kommen und sehen, dass eben äh die anderen Personen auch ’ne Prothese tragen, gibt’s irgendwas auf das Sie achten? #00:03:58-8#

Teilnehmerin 23: Nein, (Interviewerin: Ok) überhaupt (Interviewerin: Ja, mhm (bejahend), ok.) nicht. #00:04:02-6#

Interviewerin: Gut, wir hatten jetzt den Blick auf die anderen Personen gerichtet, ich würd‘ aber eben gerne zurück zu Ihnen kommen, weil Sie ja heute die Hauptpersonen sind und möchte gerne wissen, warum Sie sich gerade für Ihre Prothese entschieden haben. Es gibt ja verschiedene Prothesenarten, Sie haben jetzt gerade Ihre, wie kam’s zu der Entscheidung? Was hat es, was war da ausschlaggebend? #00:04:16-2#

Teilnehmer 8: Also für mich war die Myoelektrik ein enormer Fortschritt. (Interviewerin: Mhm (bejahend)) Weil ich eben greifen und etwas halten konnte. Zum Beispiel ein rohes Ei anfassen konnte (Interviewerin: Mhm (bejahend)) mit dieser Prothese und ich, und aber auch ’n Koffer mit 10, 15 Kilo hochheben kann. (Interviewerin: Mhm (bejahend)). (kurze Pause) Ja, was hab‘ ich im Beruf gemacht? Alles Mögliche. Traktor gefahren, äh&ähm Büroarbeiten, ähm große Empfänge mitgemacht, wo also so ’ne Hand in der man ein Messer festhalten (Interviewerin: Mhm (bejahend)) konnte, was für mich unheimlich wichtig war, dass ich beim, bei der, beim Essen hier tatsächlich mein Schnitzel schneiden konnte und das Messer festhalten konnte (kurze Pause) und deswegen war ich für die Myo sehr begeistert, unwahrscheinlich begeistert, hab auch schon gleich glaub die dritte, vierte, fünfte davon über (Name eines Prothesenherstellers aus Datenschutzgründen ausgelassen) erhalten und bei (Name eines Sanitätshauses, aus Datenschutzgründen ausgelassen) anfertigen lassen. Dann habe ich diese Michelangelo-Hand (Interviewerin: Mhm (bejahend)) gesehen und war von der ganzen Aufmachung her sehr begeistert aber im Endeffekt von der Handhabung mit keinem entscheidenden Unterschied gegenüber der Myoelektrik erlebt, im Gegenteil: ich hatte sogar einen Nachteil weil ich das Messer da nicht so konkret führen konnte und halten konnte wie ich das mit der Myo, mit dem (Interviewerin: Mhm (bejahend))

Myo-Arm erledigt habe. Aber sie war eben groß angekündigt, sie sollte sich weiter öffnen lassen, ich hab‘ auch alle Tests bestanden, übe sie auch noch, trag sie auch als Zweitprothese, wenn die mal in der Werkstatt ist (zeigt auf seine Prothese) oder irgendetwas geändert werden muss und dann nehm‘ ich die Myo, dann nehm‘ ich die Michelangelo-Hand. Also so dass ich eigentlich beide in Betrieb habe, aber vorzugsweise die Myoelektrik. #00:06:34-1#

Interviewerin: Mhm (bejahend), weil des, weil die besser funktioniert als die Michelangelo für Sie? #00:06:36-4#

Teilnehmer 8: Ja, weil ich mit der besser klarkomme und ich sogar ein bisschen den Eindruck hab, sie sieht ein bisschen netter aus. #00:06:43-2#

Interviewerin: Ok. Und meinen Sie, dass das, dass Sie das Gefühl haben Sie kommen damit besser klar, liegt das an der Gewöhnung, weil man die einfach lange hat, oder liegt das daran, dass bei der andren was schlechter funktioniert als bei der die sie jetzt tragen, einfach besser gelöst ist? #00:06:54-5#

Teilnehmer 8: Es geht bei mir, dass ich die Myoelektrik trage, weil sie etwas besser funktioniert, zum Beispiel mit dem Messergriff und mit dem (Interviewerin: Mhm (bejahend)) Tragegriff festzuhalten. Und weil sie etwas besser aussieht. (Interviewerin: Mhm (bejahend)). #00:07:08-0#

Interviewerin: Und wie ist es bei den anderen? Wie kam da die Entscheidung zu der Prothesenart? #00:07:12-9#

(kurze Pause)
#00:07:12-9#

Teilnehmerin 23: Also ich hab mich auch entschieden zu diese Armprothese, weil ich brauche tägliche Unterstützung Zuhause (Interviewerin: Mhm (bejahend)) u:::nd ja die hab ich erstmal seit ein Jahr (Interviewerin: Mhm (bejahend)), ja seit ein Jahr soweit, sonst war ich 37 Jahre ohne Armprothese und (kurze Pause) die ist schon gut, (Interviewerin: Mhm (bejahend)) krieg ich ganz, die hilft mir ganz viel, ich kann auch Fahrrad fahren (lachend). Abe::::r Übung muss man machen. #00:07:36-1#

Interviewerin: Ja, das heißt Sie hatten die ganze Zeit davor gar keine Versorgung und dann war das die erste? #00:07:42-1#

Teilnehmerin 23: Genau, (Interviewerin: Ok.) das ist die erste Armprothese. #00:07:45-3#

Interviewerin: Ok, ja. Und wie kam’s dann zu der Entscheidung, weil Sie hätten ja auch ’ne andere nehmen können? #00:07:52-1#

Teilnehmerin 23: Ä::hm ja:::, das äh, das hat mir so ähm von Arzt in (Name des Wohnorts aus Datenschutzgründen ausgelassen) eine Firma gesagt ich soll (Interviewerin: Mhm (bejahend)) sich äh:: entscheiden für diese Prothese, weil die sind besser. #00:08:08-8#

Interviewerin: Ja, ok. Und was stand zur Auswahl, wenn Sie sagen, die ist besser? Besser als welche? #00:08:13-6#

Teilnehmerin 23: Oh ich, ich weiß es nicht wie heißt die andere. #00:08:21-4#

Interviewerin: Ok, nich schlimm! #00:08:21-4#

Teilnehmerin 23: Ok (lachend) (Interviewerin: Ja..) #00:08:22-0#

Interviewerin: Und bei Ihnen Herr (Name von Teilnehmer 1, aus Datenschutzgründen ausgelassen)? #00:08:23-5#

Teilnehmer 1: Ähm bei mir war's so, dass ich am Anfang gar keine andre, keine Wahl hatte. (Interviewerin: Mhm (bejahend)) Also, ich war 16 Jahre alt und wollte eine möglichst natürliche Hand haben und (Interviewerin: Mhm (bejahend)) da waren die natürlich das einzig in Frage kommenden, (Interviewerin: Mhm (bejahend)) es gab keine Alternativen dazu. Ähm das äh muss ja nicht bedeuten, dass ich nicht im Laufe der Zeit jetzt hätte wechseln können, aber da kommt bei mir dazu, dass ich vom Schaft her äh spezielle Anforderungen habe, (Interviewerin: Mhm (bejahend)) die aus der Historie kommen. Also (Name eines Sanitätshauses, aus Datenschutzgründen ausgelassen) die haben früher spezielle Schäfte gem-, gefertigt (Interviewerin: Mhm (bejahend)) mit porösem Schaftmaterial und mit Drehgelenk, das 'ne gewissen Supination und äh Pron-, Pronation (Interviewerin: Mhm (bejahend)) erlaubt und daran bin ich irgendwie ziemlich gewöhnt und finde das funktionell hilfreich (Interviewerin: Ja.), so dass nicht unbedingt 'ne Kombination 'ner neuen Hand an dieser Prothese, a-, an den eineinhalb Jahr alten Schäften (Interviewerin: Mhm (bejahend)) die ich jetzt schon ewig trage möglich war, also deshalb hatte ich jetzt keine äh richtige Alternative (..??) dann der Neuentwicklung von Prothesen. (Interviewerin: Mhm (bejahend)) Dazu kommt, dass ich irgendwie von der Funktionalität die diese neuen Prothesen bieten skeptisch bin, dass die jetzt wesentlich was jetzt, (Interviewerin: Mhm (bejahend)) mhh wesentliche Neuerungen bringen. Also das Schnelllaufende, das ist natürlich ein wichtiger Punkt (Interviewerin: Mhm (bejahend), ja.) bei der Myo-, bei der Myo-Hand die es gibt und ä::h gewissen Steuerung und die, die Geschwindigkeitsveränderung (Interviewerin: Mhm (bejahend)) sowas ist natürlich ganz wichtig, 'n fahrbares System (wie bei?) Myoelektrik von (Name eines Prothesenherstellers aus Datenschutzgründen ausgelassen) und (Interviewerin: Mhm (bejahend)) äh ja::, das, das ist eigentlich im Prinzip das, was ich dazu sagen (möchte?). #00:09:59-7#

Interviewerin: Das heißt, dann ist einfach 'ne Skepsis da, was die Neuen betrifft und dann bleibt man, bleiben Sie lieber bei dem was, wo Sie einfach schon (Teilnehmer 1: Ja.) dran gewöhnt sind (Teilnehmer 1: Ja, ja genau)? Mhm (bejahend). #00:10:10-7#

Teilnehmer 1: (Im Prinzip, das ist im Wesentlichen ..??) Ich hab nur meine Zweifel, wie diese Prothesen (Interviewerin: Ja) die ich doppelt jetzt benutze, die haben sehr hohen Reparaturbedarf also (Interviewerin: Ja) das ist praktisch, sind immer Prothesen in der Reparatur und ich (Interviewerin: Mhm (bejahend)) habe meine Zweifel, dass die neuen Prothesen noch nicht so ausgereift sind, dass da das (Interviewerin: Ok) noch häufiger vorkommt (Interviewerin: Ja). #00:10:33-8#

Interviewerin: Dass einfach die Angst groß ist, dass man dann ganz ohne da ist, dann lässt man's lieber so wie's ist (Teilnehmer 1: (Genau?))? Mhm, ja. #00:10:41-7#

Teilnehmer 1: Ich wäre natürlich interessiert den Funktionsspielraum zu erweitern und (Interviewerin: Ja) das ist natürlich 'n ganz fundamentales Interesse aber, (Interviewerin: Ja) ähm (kurze Pause) ich bin bisher nicht dazu, hatte mich nicht entscheiden können (Interviewerin: Mhm (bejahend), ja). #00:11:03-3#

Interviewerin: Und bei Ihnen Herr (Name von Teilnehmer 8, aus Datenschutzgründen ausgelassen), wenn Sie dann eben sagen, Sie haben sich ja informiert, Sie haben gesehen da gibt's was neues, aber dann hat doch irgendwie die Gewöhnung und das, dass es eben bei der anderen eingespielt ist, dass die Funktion, weiß nicht, also ich weiß nicht, liegt das, dass Sie eben sagen bei dem anderen mit dem Messergriff, meinen Sie dass es auch daran liegen kann, dass man einfach an die Prothese gewöhnt ist und einfach äh::m in den, in die Griffe leichter reinkommt, oder ist es wirklich das, dass die andere, die neuere obwohl sie vielleicht andere Sachen verspricht, halt da 'n Nachteil hat, der dann eben so wichtig ist,

dass man sie halt weglässt? #00:11:35-7#

Teilnehmer 8: Also ich bin zu der Michelangelo-Hand (Interviewerin: Mhm (bejahend)) gekommen (Interviewerin: Ja), weil ich mir sehr viel versprochen habe unter anderem, dass ich dieses Drehgelenk verwenden kann (Interviewerin: Mhm (bejahend)), hab aber dann festgestellt, dass das nur mechanisch zu, zu erledigen ist (Interviewerin: Mhm (bejahend)) und das ist natürlich nicht sehr schön, dass man erst mit der linken Hand die rechte einschalten muss, um sie drehen zu können, oder bewegen zu können. Das war für mich dann nicht angenehm und der eindeutige Nachteil ist, dass ich dieses Messer nicht in der Qualität und in der vernünftigen Weise (Interviewerin: Mhm (bejahend)) handhaben kann, wie ich das mit der Myoelektrik-Hand ha-, machen kann. (Interviewerin: Ja) Deswegen bevorzuge ich jetzt die Myoelektrik (Interviewerin: Mhm (bejahend), ja, ok Danke schön.) #00:12:16-9#

(kurze Pause)
#00:12:16-9#

Interviewerin: Ok, wir haben jetzt e-, erstmal besprochen warum die Prothese, warum Sie sich dafür entschieden haben. Sie haben auch schon gesagt, was daran äh, was an der anderen nicht so gut läuft, mich würd' interessieren, was fallen Ihnen für Dinge ein, die positiv sind, wo Sie ganz klar sagen würden, dass das ich dann die Prothese hatte, Sie haben ja den Vergleich zu 'ner anderen davor (zeigt auf Teilnehmer 1 und Teilnehmer 8), Sie haben den Vergleich zu davor gar keine Versorgung (zeigt auf Teilnehmerin 23) - was bringt das für Vorteile mit? Also welche positiven Eigenschaften die&die, die sie der Prothese jetzt ganz klar der Prothese zuschreiben würden? #00:12:47-2#

(kurze Pause)
#00:12:49-3#

Interviewerin: (lacht) #00:12:51-2#

Teilnehmer 8: Also ganz viele, wirklich sehr viele Vorteile. Also ich kann 'n Glas halten wie ich's eben gemacht hab, ich kann äh 'ne Flasche eingießen, ich bin beweglicher, ich war zum Beispiel ständig im Stadtrat und da ist es gewöhnlich, dass man die Hand gibt. Jetzt hatte ich immer die Hand umdrehen müssen, die linke, um&um&um guten Tag (Interviewerin: Mhm (bejahend)) zu sagen, das war immer sehr umständlich, jetzt mache ich das mit der Prothese, das ging natürlich auch mit der Michelangelo sehr (Interviewerin: Mhm (bejahend)) gut, die ist noch etwas weicher, (Interviewerin: Ja) da hat se' sogar einen Vorteil, aber das hebt nicht den Vorteil gegenüber des Messers auf. Ansonsten also man kann etwas halten, man kann sich also fast wie mit beiden Händen bewegen (Interviewerin: Mhm (bejahend)) man kann also wi-, und es ist auch von der Haltung her angenehmer als wenn man nur eben die als Stütze oder nur als irgendwie, dass man etwas dran hat.. (Interviewerin: Können Sie da noch, 'tschuldigung nochmal, ganz kurz, weil ich hab Sie vorher unterbrochen was für 'ne Versorgung Sie davor hatten, also in&in was für 'nem Vergleich das steht?) Ja ich hatte vorher nur diese, diese Prothese, eine Plastikhand (Interviewerin: Mhm (bejahend)) mit einem schwarzen Handschuh drüber, da konnte man mechanisch diese, diesen Daumen auf klemmen (Interviewerin: Mhm (bejahend)) also nur eine Klemmvorrichtung, mehr war nicht zu machen (Protokollant: Haben se' das mit der linken Hand gemacht?). Mit&mit der linken Hand musste ich das auf-, a:::nziehen, (Interviewerin: Mhm (bejahend)) dann klappte das auf. Gut. Ich kann natürlich dazu sagen, dass ich zwischenzeitlich sogar Paddelboot gefahren bin, (…?) äh und da mir eben 'ne feste Verbindung (Interviewerin: Ja) der Prothese gemacht hab, was nur unangenehm war, wenn ich umgekippt bin (Interviewerin: Mhm (bejahend)) und ich dann unter Wasser mit&mit (..?) in Schwierigkeiten war. Aber ich hab's überlebt. Nein, also die alte Prothese, das war eigentlich für, ich hat se' ja nun in der ganzen Jugend, in der ganzen Kindheit erlebt, gut. Man konnte damit auch ganz gut sich verteidigen, (Interviewerin: Mhm (bejahend),

(lachend)), verwenden, aber es für einen jungen Menschen schon eine erhebliche Problematik so einen schwarzen Handschuh (Interviewerin: Ja) und so ‘ne konstante Hand nebendran zu haben mit der man nicht viel anfangen konnte. Ich konnte zwar Fahrrad fahren, ich hab Sport gemacht (Interviewerin: Mhm (bejahend)) und Fußball und alles mögliche, aber Fußball ohne Prothese. Mit dem einen Arm, mit dem Stumpf nebendran (Interviewerin: Mhm (bejahend)). Also äh das war deswegen war ich sehr begeistert, es gab ja dann die&den Handschuh, den&den hellen, die ich leider sehr viel beschmutzt habe, wo es immer zu Reinigungs- und Kostenproblemen kam, die nicht einfach zu bewältigen sind, auch wenn ich als Kriegsbeschädigter bevorzugt bin und nicht immer die Krankenkasse erst beantragen muss, aber äh es ist immer eine schwierige Angelegenheit sowas wieder ersetzt zu bekommen. (Interviewerin: Mhm (bejahend)) #00:16:26-2#

Interviewerin: Das heißt, dass aber auch bei der neuen Prothese eben im Vergleich zu dieser andren wo der schwarze Handschuh drüber war, dass auch das Optische und dieses Kosmetische wie das Erscheinungsbild, dass das auch einfach ‘ne große Rolle spielt, dass das als Vorteil von Ihnen wahrgenommen wird? #00:16:36-0#

Teilnehmer 8: Ja, absolut. #00:16:37-8#

Interviewerin: Mhm (bejahend) #00:16:38-6#

Teilnehmer 8: Beim Tanzen war es schon angenehm, dass (Teilnehmer und Moderatorin lachen) ich eine Hand hatte, die aussah wie eine Hand und nicht mehr wie eine schwarze Prothese. #00:16:44-8#

Interviewerin: Ja. Das heißt, dass es schon auch einfach viel den Effekt hat nicht nur ‘nen Hilfsgerät was mich unterstützt (Teilnehmer 8: Ja), sondern auch einfach das so ein, weiß nicht, vervollständigen oder (Teilnehmer 8: Absolut.)-. Mhm (bejahend), ja. #00:16:54-4#

Teilnehmer 8: Absolut, absolut. Das ist auch ästhetischer und man fühlt sich wohler und man fühlt sich kompletter. #00:16:56-5#

Interviewerin: Und noch von Funktionen die Sie als positiv beschreib-, weil jetzt haben Sie gesagt eben sie fühlt sich gut an, sie sieht gut aus, aber noch von Funktionen die Sie als positiv beschreiben? #00:17:08-2#

Teilnehmer 8: Also ich hab‘ gesagt ich kann ein rohes Ei (Interviewerin: Ja) festhalten, (Interviewerin: Mhm (bejahend)), so empfindsam kann ich damit umgehen (Interviewerin: Mhm (bejahend)), ganz vorsichtig, ich kann aber auch mal ‘ne Tasche oder sonst etwas an-, ich kann den Türgriff aufmachen, wenn ich die linke Hand (Interviewerin: Mhm (bejahend)) irgendwas in der Hand halte. Meine größten Schwierigkeiten waren, dass ich (kurze Pause) wenn‘s am Buffet etwas zum Verteilen gibt, (tut, als würde er in der linken Hand einen Teller halten) hier links den Teller halten musste und dann nichts mehr drauf tun konnte (Interviewerin: Mhm (bejahend)). Das hat mal die Michelangelo versprochen, dass sie‘s kann, aber ich hab‘s zwei Mal probiert äh der Buffet&das Buffet war nicht begeistert (alle lachen). (kurze Pause) #00:17:49-4#

Interviewerin: Wie ist das bei den anderen? Was für Vorteile sehen Sie an der Prothese? Oder was für positive Dinge die Ihnen einfallen? #00:17:57-6#

Teilnehmerin 23: Also bei mir positive Dinge das ist das mhh sie unt-, unterstützt mich schon am Haushalt. Also ich versuche noch mit&auch mit Gabel und Messer essen, aber das ist noch nicht so ganz einfach (Interviewerin: Mhm (bejahend)) und joah::. Fahrrad fahren kann ich auch schon (Interviewerin: Mhm (bejahend)) also… #00:18:28-9#

Interviewerin: Und wenn Sie sagen im Haushalt, was fällt Ihnen da ein, was für Tätigkeiten

sind es wo Sie sie dann benutzten? #00:18:34-5#

Teilnehmerin 23: Wäsche aufhängen kann ich, zusammen mach ich das mit den Wäscheklammer und in Küche schneid‘ Gemüse und dann krieg ich schon ein bisschen Unterstützung (lachend). #00:18:45-5#

Interviewerin: Ja, aber benutzen Sie sie vor allem Zuhause, Sie sagen zum Fahrrad fahren und zu Hause oder gibt‘s noch andere Situationen in denen Sie sie tragen, in denen Sie was bringt? #00:18:53-1#

Teilnehmerin 23: Äh:::m. Ich äh, ja ich äh, am meisten trage ich sie erstmal Zuha::use (Interviewerin: Mhm (bejahend)). Also ich zwing mich schon rauszugehen (Interviewerin: Mhm (bejahend)) Aber ist nicht so ganz einfach (Interviewerin: Ja). #00:19:06-5#

Interviewerin: Und bei Ihnen, Herr (Name von Teilnehmer 1, aus Datenschutzgründen ausgelassen)? #00:19:06-9#

Teilnehmer 1: Ähm bei aller Kritik die ich an den Prothesen habe, die nicht (Interviewerin: Mhm (bejahend)) gering ist (Interviewerin: Ja), ermöglichen die myoelektrischen Prothesen mir ein selbstbestimmtes Leben. Ich lebe alleine (Interviewerin: Mhm (bejahend)) und ich mache alles äh allein (Interviewerin: Mhm (bejahend)). Ich fahre Auto und ähm ja. Was daran eben das Wichtigste ist, ist die Greiffunktion. Das ist natürlich das eigentliche und äh::m das sich das eben an der Reparaturbedarf doch ‘n bisschen im Vergleich verringert hat. Dann die ästhetische Komponente oder die ästhetische Seite ist natürlich wichtig. War sehr wichtig irgendwie in meiner Jugend natürlich (Interviewerin: Mhm (bejahend)) und (kurze Pause) mittlerweile sollte man sich natürlich davon nicht so sehr beeinflussen lassen, aber es ist immer irgendwie noch (Interviewerin: Ja) natürlich befremdlich, wenn jemand der, wenn man jemanden die Hand gibt, was ja hier in Deutschland sehr verbreitet ist (Interviewerin: Mhm (bejahend)) und er dann erst merkt, dass man ‘ne künstliche Hand hat und entsprechend reagiert. Ja, also und es is‘ schon ohne diese Prothesen könnte ich eben nicht so gut leben also (Interviewerin: Mhm (bejahend)) nicht jedenfalls so, dann wäre ich in einem Heim oder sonst wo (Interviewerin: Mhm (bejahend), ja). Also insofern ist das schon sehr gravierend und bin ich natürlich froh, dass ich diese Prothesen haben kann. #00:20:16-4#

Teilnehmer 8: Ich hätte direkt eine Frage (Interviewerin: Ja, bitte!) wenn das passt. Ich hab‘ Sie ja bewundert, gleich als ich Sie gesehen habe (wendet sich an Teilnehmer 1). (Feine?) Hand und sieht schöner aus als meine (alle lachen), obwohl ich sehr eingebildet auf meine Myoelektrik-Hand bin, sie schmutzt natürlich, aber ich mach natürlich auch viel. Aber Sie haben sogar vorhin die Blätter umgedreht, wie konnten Sie das, das schaff ich nicht? #00:20:39-8#

Teilnehmer 1: (…???) (macht vor, wie er die Blätter umdreht) #00:20:45-4#

Teilnehmer 8: Ja. Sie sind sehr geschickt. Da könnte ich noch ‘ne Menge dazu lernen. #00:20:49-4#

Teilnehmer 1: Naja, weiß nicht ob das jetzt Geschicktheit ist. Ich meinte, das ist einfach ‘ne Technik, die man sich irgendwann angewöhnt. #00:20:56-1#

Teilnehmer 8: Angewöhnt, ja. Wenn ich Sie (Teilnehmer 1: Hängt davon ab, wie die Hand, wie die Oberflächenstruktur ist, ob das einfach geht oder nicht. Bei manchen ä::h Speisekarten ist es sehr schwierig, wenn die sehr stark zusammenkleben, (Interviewerin: Mhm (bejahend)) dann kann man nicht schieben. Schieben ist natürlich auch wichtig bei der Oberfläche der&des Handschuhs, dass man dann sozusagen manipulieren kann. Wenn das jetzt ganz glatt ist, kann man das eben halt durch schieben in die Hand nehmen). Also ich

bin beeindruckt (Interviewerinin lacht). #00:21:32-0#

Interviewerin: Ok, wir haben über die positiven Sachen gesprochen, Sie haben mir auch schon gesagt, Sie haben äh auch nicht wenig Kritik für die Prothesen über, was sind denn die negativen Dinge, was sind denn Dinge, die stören an der Prothese, die besser sein müssten? #00:21:48-0#

Teilnehmer 1: Ich hab‘ hier mal was aufgeschrieben (holt Blatt aus einem Ordner) wo ich das im Laufe meines Lebens gesammelt habe. Da stehen viele Punkte drauf die (…??). Also da hab‘ ich jetzt hier drei Punkte hervorgehoben. Einmal was mich sehr stört ist, dass ‘ne unzureichende Signaltrennung von Muskeln vorhanden ist (Interviewerin: Mhm (bejahend)), also dass es bei Bewegung irgendwie zu Fehlsteuerung kommt (Interviewerin: Ja) einfach. Das ist also sehr schlecht und finde ich. Das es ja auch keine digitale Signalverarbeitung stattfindet, das finde ich auch eben schlecht und irgendwie nicht zeitgemäß, (Interviewerin: Mhm (bejahend)) das müsste eigentlich schon seit Jahren viel besser sein (Interviewerin: Mhm (bejahend)) und, dass&der andere Punkt ist ä::hm, die sich, das war früher noch schlimmer aber es ist jetzt das es auch äh::m elektromagnetische Störungen gibt wo man in der (Interviewerin: Mhm (bejahend)) Nähe von irgendwelchen äh:: Halogenlampen oder Leuchstofflampen&Röhren war, dann ging das immer total schief. (Interviewerin: Mhm (bejahend)). Und die Akkukapazität die finde ich auch nicht ausreichend, die könnte durchaus besser sein finde ich, nach dem heutigen Stand der Akkutechnologie. Und was äh ganz äh gravierend ist, dass überhaupt keine Möglichkeit ist Touchpads zu be- (Interviewerin: Mhm (bejahend)) da hab ich schon mal vor Jahren Kontakt mit (Name eines Prothesenherstellers aus Datenschutzgründen ausgelassen) gehabt und die haben mir äh ganz lapidar geantwortet, dass da&dass da&dass die da gucken, aber da ist nix passiert, dass is‘ einfach (Interviewerin: Mhm (bejahend)), null. Und immer mehr (kurze Pause) Interface ist basierend auf Touchpad und (Interviewerin: Ja) (kurze Pause) ja, das. (Wobei ich?) noch früher andere Sachen die ich, die ich hier aufgeschrieben habe. #00:23:28-6#

Interviewerin: Wollen äh, wollen Sie, also wir haben Zeit, Sie dürfen auch gerne noch mehr nennen, (Teilnehmer 1: Ja) wenn noch mehr da sind. #00:23:33-5#

Teilnehmer 1: Ja, ich fang mal an, wenn Sie nichts dagegen haben (Interviewerin: Ja.) (Teilnehmer 8: Bitte!) Also ich hätte hier ‘n Punkt von was die Hand angeht, äh::m Schutz vor elektromagnetischen Störungen, diese Signaltrennungen, die Verbesserung des Elektrodenkontakts, also da war‘s zum Beispiel so, dass die äh Abhängigkeit von Feuchtigkeit und Temperatur ganz gravierend ist (Interviewerin: Mhm (bejahend)). Also im Winter wenn&wenn‘s kalt und die Haut (Interviewerin: Ja) trocken ist, dann funktioniert das plötzlich nicht mehr (Interviewerin: Mhm (bejahend)) und wenn ich in&in was weiß ich, in der Karibik bin (Interviewerin: Mhm (bejahend)) und jetzt tropfnass bin, dann ist natürlich auch Fehlsteuerung (Interviewerin: Ja.). Äh::m deshalb fande ich das auch immer gut eben weil diese spezielle poröse Struktur dieses Schaftes, die äh erlaubte eben halt, dass die Feuchtigkeit nach außen drang und irgendwie weitestgehend das äh konstant gehalten wurde. (Interviewerin: Mhm (bejahend)). Und dann die Elektrodenaufhängung, also bei mir ist es so, dass ich jetzt durch das Alter oder auch durch Parkinson, sind die Ärme&Arme dünner geworden und jetzt ist der Kontakt nicht mehr so äh eng, (Interviewerin: Mhm (bejahend)) das hat natürlich auch gleich ‘n Einfluss, also insofern wäre dann so ‘ne adaptive Aufhängung ganz gut oder eben, dass es ‘n Kontaktmittel gibt, die auch&das man auftragen könnte, dass einen gleichen, oder annähernd gleichen Kontakt garantiert. (kurze Pause) Und ja, der Reparaturbedarf, das ist ‘ne andere Geschichte. Was den Handschuh angeht, diese Farbgestaltung der Fingernägel mit dem weißen Rand und der Nagelhaut, dass ist nach paar Tagen weg (Teilnehmer 8 nickt) (Interviewerin: Mhm (bejahend)) irgendwie, das bringt nichts (Interviewerin: Mhm (bejahend)) und da müsste man sich irgendwie bisschen intensiver mit beschäftigen. Und dann eben halt diese Verschmutzungsempfindlichkeit, also zum Beispiel Lebensmittelfarben wie Tomaten,

Möhren, Hokkaidokürbisse oder Kugelschreibertinte und andere Tinten und zum Beispiel auch eben die Zeitung. Man kann nicht die Zeitung frisch lesen (Teilnehmer 8: Mhm (bejahend)) ohne total schwarze Hände zu bekommen (Interviewerin: Mhm (bejahend)). Was den Schaft angeht, dass ist dieses poröse und nicht Wärme isolierende finde ich gut, dann gibt‘s eben halt das speziell, ich weiß nicht ob Sie das kennen, das Drehgelenk im Sch-, das sozusagen auf der Hälfte&oder äh im unteren Teil des Arms ist hier ein Drehgelenk (zeigt auf seine Prothese). Das ermöglicht das sozusagen den unteren Teil zu drehen (Interviewerin: Ja), das kann man mit der&mit dem&mit der Supination des Unterarms machen, wenn‘s auch&es sieht nur sehr gering aus, (Interviewerin: Mhm (bejahend)) aber wenn ich zum Beispiel Suppe mit einem Löffel esse, dann erfordert schon ‘ne kleine&also bei ‘ner Veränderung kann man das anpassen, das ist sehr hilfreich, finde ich. (Interviewerin: Ja.) Ja und dann vielleicht innovative Materialien, hab‘ ich jetzt nicht gesehen, dass das irgendwie beim Schaft jetzt in der Diskussion war. Ein bisschen Probleme hatte ich mit allergischen Reaktionen. Also Teile werden ja&bei der Elektrode wird ja ‘n Teil also ‘ne Fassung in den Schaft integriert und da wurde offenbar irgendwie ’n Klebe&‘n Klebstoff verwendet, der allergische Reaktionen bei mir ausgelöst hat. #00:26:46-1#

Interviewerin: Von der Elektrode die dran war? #00:26:49-6#

Teilnehmer 1: Bitte? #00:26:49-6#

Interviewerin: Von der Elektrode, nicht vom Schaft (Teilnehmer 1: Nein, nicht von der Elektrode, sondern von diesem&da ist so ‘ne gewisse Einbettung, die die Elektrode aufnimmt und diese Einbettung wird in den Schaft integriert)? Und das ist die Einbettung die die Reaktion-. (Teilnehmer 1: Genau, die Einbettung, da (…???) Mhm. #00:27:11-6#

Teilnehmer 1: Ja die Geräuschwahrnehmung das war am Anfang ‘n großes Problem, hat mich gestört, aber mittlerweile höre ich nicht mehr so gut, insofern (lachend) (Interviewerin: Mhm (bejahend)) ist das nicht mehr so gravierend (alle lachen). (Interviewerin: Ok.) Und dann was natürlich noch wünschenswert wäre, der Griffkontakt, diese punktuelle Greifmöglichkeit, das ist natürlich insbesondere, wenn die&wenn der Handschuh langsam aushärtet, am Anfang ist der sehr weich (Interviewerin: Mhm (bejahend)), man kann ähm punktuell was relativ festhalten. Aber so ‘n punktueller Festgriff das ist natürlich (Interviewerin: Mhm (bejahend)) ve-. Also ich hab immer Probleme mit dem Messer wenn ich das angreife und was sch-, schneide, ‘n Steak oder sowas, das ist irgendwie immer schwierig also (Interviewerin: Ja), man kann nicht fest genug greifen, deshalb wäre es wünschenswert, wenn das ‘n bisschen ähm adaptieren würde, also wenn das ‘n bisschen weicher wäre und wenn man sozusagen aus diesem Punktgriff ‘n Flächen-&‘n bisschen flächig das festhalten (Interviewerin: Mhm (bejahend)), mit der Folge, dass es vielleicht einfach ‘n stabileren Sitz garantiert also das, (Interviewerin: Ja).. Und die&was die Schaftechnologie angeht, da könnte man sicher auch noch was machen, ich mein heutzutage ist ja äh:::&bei mir ist es jetzt mittlerweile so, dass&dass ich einfach eben mit dem Arm in&in den Schaft kann und manchmal mit ‘ner bestimmten Folie, die ich doppelt nehme (Interviewerin: Mhm (bejahend)) kann man das erleichtern, wenn der Arm sich ver-&verdickt hat oder sowas. Aber das ist ja nicht mehr die State of the Art jetzt. Heutzutage wird das ja mit anderen&mit ir-, ich weiß nicht wie der Begriff da ist? #00:28:38-4#

Interviewerin: Diese Anz-&Einz- oder Anzugshilfen&Anziehhilfen (Teilnehmer 8: Die hab‘ ich dabei.). Ja. #00:28:58-0#

Teilnehmer 1: Also das f-&finde ich ist nicht unbedingt notwendig und aufwendig (Teilnehmer 8 holt seine Anziehhilfe aus der Tasche und zeigt sie.). Ahja. (Teilnehmer 8: Das ist dieses Teil das ich drüberzieh‘.) Sie ziehen das vorne raus, ja? (Teilnehmer 8: Ja, ich kann‘s ja dann mal vorführen. Wenn ihr euch nicht erschreckt.) Aber Sie, Sie haben

innen k-&keinen Überzug über den Stumpf? (Teilnehmer 8: Nein.) (Interviewerin: Ich würd‘ vorschlagen, dass wir das später machen.) (Teilnehmer 8: Nachher machen.) (Interviewerin: Genau, dass wir erstmal jetzt hier beim Thema.) (Teilnehmer 8: Weil ich da auch noch was dazu sagen möchte.) (Interviewerin: Ja, genau.) Und dann anderer Punkt die Handpositionierung finde ich auch noch ein wichtiger Punkt. ‘Ne aktive oder ‘ne passive, also ich fänd‘s gut, wenn man den&die Hand, auch wenn‘s nur sozusagen mit der anderen Hand ist, wenn man die ‘n bisschen in der Orientierung verstellen könnte (Interviewerin: Mhm (bejahend)). Also manchmal&also dass man den abwinkeln könnte vom Arm, die Hand und die&dass die dann den Moment irgendwie fest einrastet, dass man ‘ne spezielle Handposition beim Essen hat. Und dass die nicht irgendwie immer so grade nach vorne zeigt, dass ist irgendwie, ja, dass müsste also ich würde v-&vorziehen, wenn man das sozusagen aktiv mit der anderen Hand machen könnte und weniger also man kann sich ja vorstellen, dass das auch elektrisch oder so halt, aber ich meine die Anzahl der Signale die zur Verfügung st-&stehen zur Steuerung der Prothese ist ja nun begrenzt und ob das dann ‘ne richtige Entwicklungsrichtung ist, weiß ich nicht. Interviewerin: Mhm (bejahend)). Ja, das war eigentlich im Prinzip das, was ich eigentlich jetzt-. #00:30:35-0#

Interviewerin: Können Sie das nochmal kurz-. Weil vorher haben Sie gesagt, dass da das Drehgelenk (zeigt auf den Arm), das können Sie drehen. Was ist genau die Bewegung, wo Sie sagen, dass es fehlt, das hab‘ ich jetzt noch nicht ganz verstanden, wo ähm Sie jetzt gesagt haben beim Essen das Feststellen (Teilnehmer 1: Ja.), welche Bewegung genau ist das, die da fehlt, die Sie da vermissen, wenn Sie das nochmal-. #00:30:54-2#

Protokollant: Sie meinen diese Bewegung hier? (zeigt Flexion/Extension im Handgelenk) #00:30:51-9#

Interviewerin: Die da? #00:30:53-5#

Teilnehmer 1: Ja, ich (kurze Pause). (Teilnehmer 8: Ich glaub ich weiß welche er meint.) Ich meine diese&diese Drehbewegung is‘ und die Orient-&also wenn ich jetzt zum Beispiel ‘n Löffel habe, der wenn ich das jetzt so fasse (tut, als hätte er einen Löffel in der Hand, den er zum Mund führt) dann ist der&ist die Ebene auf&des Löffels sozusagen so schräg. Ich brauch aber ‘ne Ebene die verhin- also die gewährleistet, dass die Suppe nicht vom Löffel läuft. Deshalb muss das horizontal sein, das ist jetzt hier nicht gegeben. (Interviewerin: Mhm (bejahend)). In der Regel mach ich das natürlich <u>so </u>(führt es vor), dass man das horizontal hat (Interviewerin: Mhm (bejahend)) und dann ähm mit den&mit der Schulter und so weiter das irgendwie (Interviewerin: Ok ja, sie kompensieren das über die-. Ja.)-. Und wenn man das jetzt äh bisschen&bisschen adaptieren könnte am Anfang schon, dass man sich nicht immer verrenken muss (Interviewerin: Mhm (bejahend)) oder, dass man das eben auch durch diese Drehbewegung ausgleichen kann, (Interviewerin: Ja) das wäre gut. Also&also da weiß ich jetzt auch nicht&kann ich nicht so genau sagen was ich&also ich könnte mir vorstellen, wenn ich jetzt die auch ‘n bisschen nach innen drehen könnte oder nach oben klap-&klappen könnte sozusagen, dass es ‘n Vorteil gäbe. (Interviewerin: Ja) Aber jetzt ganz genau kann ich das jetzt n-&nicht sagen. #00:31:53-9#

Interviewerin: Gab‘s noch ‘n Punkt den Sie haben, weil sonst würde ich&Sie haben gesagt, dass Sie (wendet sich an Teilnehmer 8) da noch ähm was gerne hinzufügen würden? #00:31:58-5#

Teilnehmer 8: Eigentlich kann ich nur ergänzen. Einmal diese Verschmutzung, (Interviewerin: Mhm (bejahend)) die also sehr unangenehm ist, dass der Handschuh verschmutzt, man dann das ganze Teil austauschen muss, was sehr teuer ist (Interviewerin: Mhm (bejahend)) und ähm das gleiche gilt für die Hygiene. (Interviewerin: Mhm (bejahend)) Es wäre ganz angenehm denn hier zwischen (Gelenken?), zwischen den Fingern können sich Schmutzteile (Interviewerin: Mhm (bejahend)) oder Essensreste aufhalten und die müsste man ja irgendwie entfernen können (Interviewerin: Ja). Ich krieg diese Finger aber

nicht auseinander (zeigt auf die Finger II-IV) und kann sie nur unter ganz schwierigen Umständen reinigen. Das Geräusch ist meiner Ansicht nach besser geworden, also leiser geworden, man hört‘s jetzt nicht (bewegt die Prothese auf und zu). Bei der Michelangelo ist aber dazu immer noch ein Piep-Ton nötig (Interviewerin: Mhm (bejahend)). Der meine Frau immer sehr nervös macht, (alle lachen) wenn ich jedes Mal piepse, bloß weil ich irgendetwas angreife. (Interviewerin: Mhm (bejahend)) Und äh ja, die Elektroden die sitzen ja da oben (zeigt den Sitz der Elektroden) und müssen sehr exakt sitzen (Teilnehmer 1: Mhm (bejahend)), das muss also wirklich exakt sein. Jetzt hab‘ ich tatsächlich ‘n bisschen abgenommen, schon verändert sich der Arm und an manchen Stellen fängt der dann an stehen zu bleiben. Ich muss also nachjustieren und (Name eines Sanitätshauses, aus Datenschutzgründen ausgelassen) beziehungsweise (Name eines Orthopädietechnikers aus Datenschutzgründen ausgelassen) aufsuchen, dass man da irgendwie was macht. Er sagt aber: „Sehr viel ändern kann man eigentlich nicht.“, äh es müssten dann ein neuer Innenschaft äh&äh hergestellt werden. Der neue Innenschaft ist bei mir weicher geworden, als früher (Teilnehmer 1: Mhm (bejahend)) und das ist ganz angenehm, aber eins ist natürlich auch klar: Wenn ich richtig schwer arbeite und dabei ins Schwitzen gerade (Interviewerin: Mhm (bejahend)), dann fangen die Elektroden an feucht zu werden und ich bekomm‘ also unregelmäßige Aktionen oder ich muss sie ganz abstellen (Teilnehmer 1: Mhm (bejahend)). Ich kann natürlich die Hand so abstellen (demonstriert, wie er die Hand abstellen kann). Jetzt bewegt sich gar nichts mehr. (Interviewerin: Mhm (bejahend)). Dann kann ich hier wieder einschalten, dann kann ich auf- und zumachen, in allen Richtungen. (Öffnet und schließt die Prothese, während er den Arm auf verschiedenen Höhen hält) Ob ich sie oberhalb, unterhalb halte, das funktioniert bei mir sehr gut (Interviewerin: Mhm (bejahend)). Aber wie gesagt, ich bin auch nur Unterarmamputiert und habe auf diese Weise nicht viele Möglichkeiten mit dem Stumpf zu arbeiten. #00:34:35-4#

Interviewerin: Aber das heißt so das Problem mit den Elektroden, dass die nicht immer greifen, haben Sie auch, wenn Sie sagen Sie arbeiten schwer, sie schwitzen und dann- #00:34:43-1#

Teilnehmer 8: Sobald ich äh, sobald sie feucht werden oder ich schwitze oder körperliche Veränderungen, dass sich da der Arm etwas verändert oder die Muskelstruktur, dann hab‘ ich Schwierigkeiten die Bewegungen auszuführen. (Öffnet und schließt seine Prothese, die Prothese stockt) Ah, jetzt da, das ist jetzt schon wieder eine&ein Ausfall, ‘ne? Ja, so. Jetzt wenn ich da was reintun will, (Interviewerin: Mhm (bejahend)) steh ich natürlich verzweifelt da (Interviewerin: Mhm (bejahend)), hab hier irgendwas in der Hand, muss das erst abstellen und versuchen die Hand wieder richtig in die Stellung zu bringen. Das sind Nachteile. Also die Passform ist eine äußerst wichtige Sache (Interviewerin: Mhm (bejahend)) und die muss wirklich bestens geregelt werden, dass diese Elektroden auch an der richtigen Stelle, wo mein Muskel sich bewegt innen drin, ähm dass der auch anschlägt, nicht? Und das ist der Akku, den ich da noch hab‘, ‘ne? (Interviewerin: Mhm (bejahend)) (holt den Akku raus und zeigt ihn). Jetzt bin ich natürlich leblos. #00:35:52-5#

Interviewerin: Mhm (bejahend), ja. Und noch zur Laufzeit, weil Sie Herr (Name von Teilnehmer 1, aus Datenschutzgründen ausgelassen) haben gesagt, dass die Akkulaufzeit auch ‘n Problem ist. Ist das&empfinden Sie das genauso, ist das bei Ihnen auch ‘n Problem? #00:36:04-5#

Teilnehmer 8: Jetzt kommen wir wieder-. Ja, also ich hab‘ vorhin vergessen den Michelangelo drauf hinzuweisen, die kann ich nur aufladen, wenn ich die ganze Hand hinlege und das Aufladegerät dran stücke (zeigt auf die Michelangelo-Prothese). Hier kann ich natürlich einen Akku hier reinstecken (deutet auf die myoelektrische Prothese, die er trägt) und einen anderen Akku nehmen und damit weiterarbeiten. Für mich also kein Problem zwei Akkus am Tag zu verbrauchen und äh dann bin ich aber gut versorgt (Interviewerin: Mhm (bejahend)). #00:36:34-1#

Teilnehmer 1: Ja bei doppelseitig Amputierten ist es natürlich ein höherer Bedarf (Teilnehmer 8: Höherer Bedarf.) weil mehr Aktivität stattfindet. (Interviewerin: Ja, klar.) Mhm (bejahend). #00:36:40-7#

Interviewerin: Aber haben Sie dann auch sowas wie diesen&diesen Tauschakku? #00:36:44-7#

Teilnehmer 1: Ja, klar. #00:36:44-7#

Interviewerin: Ok, aber trotzdem ist es halt einfach, der andre ist einfach schneller aufgebraucht und das- #00:36:48-0#

Teilnehmer 1: Ja, ich mein man ist unterwegs, (gerade?) während der Berufstätigkeit und also immer den Ersatzakku mitzuschleppen (Interviewerin: Mhm (bejahend)) (Teilnehmer 8: Mhm (bejahend)), das ist natürlich irgendwie lästig. #00:36:52-4#

Interviewerin: Ja, klar. #00:36:52-4#

Teilnehmer 1: Und ich f-&glaube nicht, dass es sein muss. Weil die Akkukapazität finde ich nicht so überragend groß (Interviewerin: Ja, mhm (bejahend)). #00:36:59-3#

Teilnehmer 8: Haben Sie die gleichen Akkus drin? #00:37:02-0#

Teilnehmer 1: Nein, ich habe jetzt auch schon mal aufgrund meiner besonderen Voraussetzungen hab ich noch immer die alten Akkus, diese Nickel-Cadmium-Akkus. (Teilnehmer 8: Mhm (bejahend)) Diese etwas länglichen, quadratischen, kleinen Blöcke. (Teilnehmer 8: Mhm, kenne ich nicht.) #00:37:13-0#

Interviewerin: Frau (Name der Teilnehmerin 23, aus Datenschutzgründen ausgelassen) ich würd‘ Sie gerne nochmal- (Teilnehmer 8: Ja. (lachend)) #00:37:15-6#

Teilnehmerin 23: Ja, ich kann auch was zu sagen zu meiner Armprothese. (Interviewerin: Ja.) Also was ist mein Problem bei der Armprothese, dass sie so&so schwer ist (Interviewerin: Ok). We::il, ich hab hier keine Schulter und was stört mich noch immer, diese Schulter hier. Weil bisschen mehr ist hier mit der Armprothese (Interviewerin: Mhm (bejahend)) (Teilnehmerin 23 zeigt auf die Stelle, an der ihre Schulter fehlt) und das ist mein größtes Problem. Och ja, (kurze Pause) und sonst ja, (kurze Pause) sonst ist alles gut (lacht). #00:37:44-2#

Interviewerin: Aber wenn Sie jetzt ein bisschen Bezug auf das nehmen, was die anderen gesagt haben, wenn Sie sagen, Sie üben einfach gerade noch zu Hause, Sie nutzen das (Teilnehmerin 23: Ja). Sowas wie zum Beispiel mit den Elektroden (Teilnehmerin 23: Ja.), oder gibt‘s einfach Sachen, wo Sie sagen, bei den Übungen die ich Zuhause mach‘, wenn ich die benutz‘, merk ich, dass das auch so ist, dass ich auch solche (…??)? #00:38:09-1#

Teilnehmerin 23: Also ne, da wenn ich übe mit ihr dann hab‘ ich keine Probleme (Interviewerin: Mhm (bejahend), ok.), also das funktioniert alles richtig gut, da kann ich mich gar nicht beschweren, also ist alles gut. #00:38:21-1#

Interviewerin: Mhm (bejahend), ok. Und was mich jetzt noch interessieren würde ist: Sie haben viele technische Aspekte genannt die einfach negativ sind. Gibt‘s Situationen, in denen man das besonders merkt? So wie Sie zum Beispiel gesagt haben (wendet sich an Teilnehmer 8) nach der Arbeit, dann greifen die Elektroden nicht oder (wendet sich an Teilnehmer 1) vorher haben Sie gesagt mit dem Essen, fällt Ihnen da irgendwas ein, einfach ‘ne Situation, wo Sie sich besonders oft ärgern, weil eben irgendwas von den Dingen nicht funktioniert? #00:38:46-5#

(kurze Pause)
#00:38:47-7#

Teilnehmer 1: Also es gibt Situationen die kritisch sind, wenn ich irgendwie unterwegs bin, öffne die Tür und plötzlich stelle ich fest, da ist ‘nen elektromagnetisches Feld das verhindert, dass die Prothese wieder sich öffnet (Interviewerin: Mhm (bejahend)). Oder, dass sie sich schließt, wenn ich mit meiner Tochter unterwegs war, als sie klein war, und sie die Hand angefasst hat und es kommt zum unwillkürlichen Schließen (Interviewerin: Mhm (bejahend)). Da muss man höllisch aufpassen, dass&das man&das ist natürlich äh echt ‘n Nachteil, aber- #00:39:17-5#

Teilnehmer 8: Gut, da kann ich noch ‘n Beispiel sagen: Ich hab mit der Prothese den Kühlschrank aufgemacht (Interviewerin: Mhm (bejahend)) und da ist scheinbar durch‘s Aufmachen dann Fehlstrom entstanden und ich hing fest (alle lachen). Nicht? Ich konnte nicht mehr öffnen (Teilnehmer 1: Ja.) und äh musste dann also mit Gewalt mit der Hand die Prothese aufmachen, um wieder vom Kühlschrank loszukommen. (Teilnehmer 1: Ja, wenn das geht. Also ich hab‘ die bisher nicht aufgekriegt sozusagen, aktiv mit der Hand, gegen den Widerstand. Das finde ich extrem schwierig. Und auch dieses Ausschalten, man hat ja ‘n Schalter in der Hand, der erlaubt es elektrisch auszuschalten. Da ist es irgendwie immer problematisch, dass es irgendwie, weiß ich nicht, ‘n Herstellungs-äh-mangel, der geht so schwer teilweise, dass man den nicht mehr betätigen kann.) Das kann ich bestätigen, ich musste richtig Gewalt anwenden, um den Daumen aufzukriegen und hatte schon hohe Befürchtungen, dass ich (Name eines Sanitätshauses, aus Datenschutzgründen ausgelassen) zur neuen Reparatur anhalten muss. Man macht ja nicht gern was kaputt an den Prothesen, weil es ist ja für einen immer große-&großer Aufwand. Man muss es hinbringen, danach muss es wieder angepasst werden (Interviewerin: Ja.) und es muss dann wieder so funktionieren, dass man also wirklich damit umgehen kann. Es ist schlimm genug, wenn man ‘ne neue Prothese kriegt, dass die dann auch wieder in der ganzen Qualität funktioniert die man braucht. Denn sonst macht‘s keinen Spaß, wenn man nur die Hälfte mit machen kann, macht‘s kein Spaß, ‘ne? (Interviewerin: Ja.) #00:41:01-2#

Interviewerin: Und, was mich dann jetzt noch interessieren würde ist, ob es Situationen gibt, in denen Sie die Prothese gar nicht nutzen. Also vielleicht jetzt nicht mal auf diese Nachteile bezogen, die wir gerade die ganze Zeit besprochen haben, sondern auch einfach, ob‘s Situationen gibt, wo Sie sie einfach weglassen und wieso das so ist? #00:41:11-7#

Teilnehmer 8: Also, wenn ich ins Schwimmbad gehe, habe ich keine Elektrische an (lachend) (alle lachen) (Interviewerin: Mhm (bejahend)). A::ber vor einigen Jahren hab ich eine Schwimmprothese bekommen. Ich tausche die dann aus (Interviewerin: Mhm (bejahend)) und äh mit der kann ich dann schwimmen. Ich meine ohne Arm ko-&mit einem Arm konnte ich auch schwimmen, mit einem Arm und Stumpf, aber jetzt hab‘ ich die Schwimmprothese an und es sieht so aus als wenn ich mit beiden Händen, das ist sehr angenehm, man läuft nicht mehr mit dem Stumpf herum (Teilnehmer 1: Mhm (bejahend)). Das ist mit dieser Schwimmprothese, die eine ganz normale und unbewegliche ist, da merk ich auch was für ein Unterschied gegenüber es der (Interviewerin: Mhm (bejahend)) Myoelektrik ist. (Interviewerin: Ja.) Wenn ich die anhab‘ kann ich nichts festhalten, sondern muss es immer gegen den Körper drücken, um was festzuhalten, wenn ich die Hand, äh die andere frei habe. Da merk ich eben erst wie viel Möglichkeiten die Myoelektrik bietet (Interviewerin: Mhm (bejahend)). Oder ähm hier auch die Michelangelo bietet.
#00:41:55-8#

Interviewerin: Und außerhalb des Schwimmbads, des Schwimmens? Gibt‘s noch irgendwas was&wo es Ihnen einfällt? Vielleicht eben doch auch, dass sie halt irgendwo gar nicht funktioniert und Sie sagen, da lass ich sie weg, da nutz ich sie nicht? #00:41:59-1#

(kurze Pause)
#00:42:01-3#

Interviewerin: Oder fällt jemandem- #00:42:04-2#

Teilnehmer 8: Also ich geb‘ Ihnen manchmal recht, bin ich in der Früh zu faul die Prothese anzuziehen (lachend). Zum Beispiel äh zum Rasieren, oder mich zu waschen (Interviewerin: Mhm (bejahend)) und mich zu waschen oder so, das mach ich ohne Prothese. Also weil erstens Mal wird se‘ feucht und zweitens Mal brauch ich sie dazu nicht (Interviewerin: Mhm (bejahend), ja). #00:42:14-2#

Interviewerin: Und bei den anderen? #00:42:20-2#

Teilnehmer 1: Also bei mir ist es so, dass ich äh natürlich ähm schlafen geh ohne die Prothese. Ähm wenn ich irgendwo bin am Strand bin ich ohne Prothese (Interviewerin: Mhm (bejahend)) und morgens wenn ich aufstehe und Zeit habe und das Frühstück mache und so weiter, das mach ich meistens auch ohne. Also sofern es möglich ist irgendwie und das ist irgendwie möglich in gewisser Weise. Dann genieße ich das ohne, weil die direkte Aktion mit dem Feedback was man über Kontakt über den&die Haut äh über die Sensorik&äh also Haptik, weil das irgendwie doch immer angenehmer ist (Interviewerin: Mhm (bejahend)). Ja, das. Aber sonst brauch ich die unbedingt, also ich hab‘ jetzt durch Historie nicht nur ‘n zweites Paar, sondern auch noch ‘n altes drittes Paar (Interviewerin: Mhm (bejahend)) und das ist schon ganz wichtig. Sonst wenn da&wenn da alle ausfallen würden, dann könnte ich sofort irgendwie, müsste ich Notsignal geben (Interviewerin: Mhm (bejahend)). Weil ich dann nicht (Teilnehmer 8: Mhm (bejahend)) rausgehen könnte. #00:43:36-9#

Interviewerin: Ja. #00:43:38-7#

Teilnehmerin 23: Ja also ich ähm. Schlafen geh ich auch ohne Armprothese, weil das geht nicht mit Armprothese zu schlafen (lachend) (Interviewerin: Mhm (bejahend)), das wär zu schwierig. Sonst ja, manchmal denk ich nicht an die Armprothese, dass ich eine Armprothese Zuhause habe, das ist bei mir Problem (lachend). Ich äh stehe morgens auf und geh ich zur Arbeit: „Oh, meine Prothese.“ Das vergess‘ ich immer (Teilnehmer 8: Mhm (bejahend)). Das ist schwierig. #00:43:57-0#

Interviewerin: Und Sie haben vorher gesagt Sie müssen sich manchmal zwingen die zu benutzen. #00:43:58-9#

Teilnehmerin 23: Genau, ja. #00:43:58-9#
-
Interviewerin: Ist das, weil einfach durch-&äh ja oder woran liegt das? #00:44:03-2#

Teilnehmerin 23: Bei den&die Armprothese liegt daran, dass äh ja die ist ziemlich schwer (Interviewerin: Mhm (bejahend)) und dieses ganze, wie sagt man das hier (zeigt auf ihre Schulter)? Wie das aussieht alles hier, das gefällt mir nicht (Interviewerin: Ja.), weil sie so&so (groß?). (Interviewerin: Ok.) Und das ist mein Problem an der Armprothese. #00:44:17-1#

Interviewerin: Also hauptsächlich auch dieses, was Sie gesagt haben, das Gewicht (Teilnehmerin 23: Genau.) Aber auch einfach dieses Optische? #00:44:21-3#

Teilnehmerin 23: Genau, genau. Der Optische und ich hab‘ auch immer so ein Gürtel hier (Interviewerin: Mhm (bejahend)) (zeigt den Verlauf des Gurtes, der die Prothese am Oberkörper befestigt). Und das ist auch nicht so schön (Teilnehmer 8: Mhm (bejahend)). Wenn ich schöne Top anziehe oder sowas dann sieht man den Gürtel immer hier. (Teilnehmer 8: Mhm (bejahend)) (Interviewerin: Ja.) #00:44:32-2#

Interviewerin: Aber von der Funktion her, weil wenn Sie sagen eben, haben Sie vorher auch schon gesagt, dass dieses Optische, das&das Wichtigste ist, (Teilnehmerin 23: Ja.) aber von der Funktion her, ist es dann doch was wo es sich lohnt sich zu zwingen? #00:44:40-0#

Teilnehmerin 23: Genau, das lohnt sich zu zwingen, ja (lachend). #00:44:43-8#

Interviewerin: Ok. #00:44:46-5#

Teilnehmerin 23: Ich hab‘ auch Therapie gemacht, die haben mich auch ganz geholfen und die haben gesagt zu mir mein Kopf ist das Problem (lachend). (Interviewerin: Ok, ja.) Ja. (Teilnehmer 8: Mhm (bejahend)) Aber ich hab auch nicht so v-&ich hab sie erstmal seit einem Jahr und (kurze Pause) ich übe (lachend). #00:45:04-6#

Interviewerin: Ja klar und wenn Sie gesagt haben Sie haben davor so lange ohne, (Teilnehmerin 23: Genau, ja.) dann ist man wahrscheinlich da auch ganz gut drin. (Teilnehmerin 23: Ja.) #00:45:08-6#

Teilnehmerin 23: Ja und ich hab‘ auch Zuhause ein Tochter und hab ich alles mit einem Arm gemacht bei ihr und ja, so ein jetzt Arm dazu zu haben ist ganz anderes Gefühl im Körper (lachend). (Interviewerin: Ja, klar.) #00:45:24-5#

Interviewerin: Und, noch eine Frage: (wendet sich an Teilnehmer 8) Sie haben gesagt im Schwimmbad nutzen Sie sie nicht. Wie ist das denn im Bad, wenn man zum Beispiel duscht, da darf die ja eigentlich auch nicht mit dabei sein, also nicht nass werden? (Teilnehmer 8: Nee, nee nee). Also (…??). #00:45:39-9#

Teilnehmer 8: Also ich habe gesagt zum Morgen, zur Toilette (Interviewerin: Mhm (bejahend)) dann zieh ich die noch nicht an (Interviewerin: Ja.). Zum Frühstück zieh ich sie meisten an, weil&um zum Beispiel ein Ei zu halten (alle lachen) und das zu essen ist gar nicht so einfach, ‘ne? (Interviewerin: Mhm (bejahend)) Äh da gibt es schon&also da wär ich schon dankbar wenn noch ‘n paar Hilfsmittel dazu kämen und sich Leute oder man sich gemeinsam Gedanken macht wie man das verbessern kann. Wie man da (alte?) Funktionen machen kann. Es ist auch nicht leicht Marmelade aufzustreichen auf Brot, oder Butter, das sind alles sehr komplizierte Sachen und man merkt hier an solchen Stellen, dass man doch tatsächlich behindert ist (Interviewerin: Mhm (bejahend)), nicht? Obwohl ich im Leben alles gemacht habe äh vom Auto fahren, vom (kurze Pause) ach Stapelfahrer fahren, Maschinen verladen auf den LKW (Interviewerin: Mhm (bejahend)) und alle Dinge gemacht, körperlich schwere genauso wie eben da im Büro dann Maschinen zu verkaufen oder die Kostenrechnung zu erstellen oder dergleichen. Das einzige was halt war, dass meine Überzieher, so ‘nen ich meine Handschuhe: Die Überzieher, äh dabei häufig verschmutzten (Interviewerin: Mhm (bejahend)) und ich dann vom Versorgungsamt heftige Vorwürfe bekam, weshalb ich so einen heftigen Verschleiß hier sich bei mir feststellt (Interviewerin: Mhm (bejahend)). Hier ist es ja auch wieder (zeigt auf seine Prothese) ich hab‘ ‘ne schmutzige Hand und krieg die nicht mehr sauber (Interviewerin: Mhm (bejahend)). Da innen überhaupt nicht, das ist ‘nen ganz großes Problem, sieht nicht appetitlich aus. #00:47:21-8#

Interviewerin: Und Sie haben gerade gesagt, wenn es da&also wenn man da noch Hilfsmittel hinzufügen könnte. Was wären das denn für welche? #00:47:26-6#

Teilnehmer 8: Ich hab‘ mir selber zum Beispiel einmal ein Messer gebaut (Interviewerin: Mhm (bejahend)). Mit Hilfe von verschiedenen-. Indem ich ein Messer das ich hier genau reinstecken konnte (zeigt auf den Raum zwischen dem II. und III. Finger an seiner Prothese) mit einer kleinen Schablone oben, dann hielt es fest und ich machte so zu (schließt seine Prothese vollständig). Nein, so drei Viertel zu (schließt die Prothese zu drei Vierteln)

(Interviewerin: Mhm (bejahend)), soweit zu und hatte das Messer da drin und damit war es fest. Dann schaltete ich aus und das war fest (Interviewerin: Mhm (bejahend)) und ich konnte damit jedes Kotelett, jede-, Kotelett ist schon schwieriger als Schnitzel (Interviewerin: Mhm (bejahend)). Ja auf sowas muss ein Einarmiger achten (Interviewerin: Ja.), 'ne? Oder Sie ja auch (wendet sich an Teilnehmer 1), nicht? (Teilnehmer 1: Ja, nein ist klar.) Also manchmal bin ich dann beim Bestellen am überlegen: „Soll ich das nehmen?" (Teilnehmer 1: So geht es mir auch.) Nicht? Weil (kurze Pause) Eisbein essen ist eine Kunst, 'ne? #00:48:37-7#

Teilnehmer 1: Aber solche speziellen Messer zu konstruieren bringt finde ich nicht so viel, weil man das nicht bei sich führt und man geht in Restaurant und (Interviewerin: Mhm (bejahend)) mit unterschiedlichen Bestecken und man verreist und so weiter (Teilnehmer 8: Ja.) also das ist&hindert einen schon ganz wesentlich. (Teilnehmer 8: Das man di-) Ist besser man schafft die Voraussetzungen jedes Messer irgendwie gut anzufassen. #00:48:56-9#

Interviewerin: Ja. Das heißt es muss eigentlich schon was sein was die Prothese in sich hat, was (Teilnehmer 1: Ganz genau.) (Teilnehmer 8: Ja.) die von alleine kann? #00:49:01-6#

Teilnehmer 8: Also ich hatte zu dem Messer ein kleines Etui, (Interviewerin: Ja.) wo ich das reinstecke, zuschloss und dann gelegentlich mitführte (Interviewerin: Mhm (bejahend)). Wenn ich's vergessen hatte, dann war aus mit dem Essen (Interviewerin: Ja.). #00:49:12-9#

Interviewerin: Wir sind tatsächlich schon bei der Hälfte der Zeit angekommen. Ich weiß nicht, es hat jetzt niemand was gesagt, aber ich würde anbieten, dass wir jetzt einfach 'ne 15 minütige Pause machen, dass Sie genau&hinten steht, ich weiß nicht ob so spät jemand Kaffee möchte, aber Kaffee und nochmal Wasser und noch was Kleines zur Stärkung. Dann würden wir einfach kurz 'nen Moment Pause einlegen. #00:49:30-0#

(kurze Pause)
#00:49:30-0#

Interviewerin: Vor der Pause haben Sie erzählt äh:::m in welcher Situation Sie die Prothese freiwillig nicht nutzen. Ich wüsste jetzt gerne, Sie haben es vorher auch schon angesprochen, wenn die Prothese kaputt geht, was fehlt dann am meisten? Oder wie kompensieren Sie das? Sie haben schon gesagt, Sie haben Ihre Prothesen Zuhause (wendet sich an Teilnehmer 1) aber da wüsste ich gerne was&wie Sie damit umgehen, wenn die Prothese mal gar nicht das ist, wenn die kaputt geht. #01:14:31-1#

Teilnehmer 1: Also bei mir ist es so, eigentlich gibt es gar keine Ersatzversorgung von Seiten der Krankenkasse, das ist 'n Problem (Interviewerin: Mhm (bejahend)). Irgendwann hat man das erreicht, hab' ich erreicht, dass trotzdem in der Situation als doppelt Amputierter, (Interviewerin: Mhm (bejahend)) dass die das dann doch gemacht haben, aber jetzt irgendwie, wenn es darum geht Ersatzprothesen zu reparieren kommt immer Widerstand, man muss immer 'ne Einzelfallentscheidung machen (Interviewerin: Mhm (bejahend)). Wenn die Prothesen ausfallen, oder mal doppelt ausfallen, das kommt ja relativ selten vor (räuspert sich), dann bleib ich Zuhause und kann ohne zusätzliche Hilfe Zuhause mein Leben für ein paar Tage organisieren (Interviewerin: Mhm (bejahend)). Also das ist schon möglich. #01:15:06-3#

Interviewerin: Und Sie haben jetzt erreicht, dass Sie jetzt eben auch 'ne Ers-&Ersatzprothese haben. Wie war das davor? (Teilnehmer 1: Davor das-) Da sind Sie einfach Zuhause geblieben? #01:15:12-6#

Teilnehmer 1: Davor, das&als ich äh (kurze Pause) da sind wir&haben wir sozusagen lebte ich bei meinen Eltern. Als 16-jähriger bin ich zu Schule gegangen (Interviewerin: Mhm

(bejahend)) und hab dann noch ‘ne zusätzlich Aus-, also das ist passiert nachdem ich (mir?) die Volksschule beendet hatte (Interviewerin: Mhm (bejahend)), ‘n Unfall, ‘n Explosionsunfall und äh dann wurde&haben wir uns entschieden, dass ich noch weiter äh als-&einen Quereinstieg auf die Realschule mache (Interviewerin: Mhm (bejahend)) und hab dann sozusagen Schulunterricht weiterhin gehabt. Und äh dann ist es so gewesen, wenn die Prothese ausgefallen ist, was sehr häufig der Fall war, dann hat mein Vater eben äh mich in den Wagen gesetzt und wir sind direkt zu (Name eines Prothesenherstellers aus Datenschutzgründen ausgelassen) gefahren und die haben das v-&vor Ort dann direkt ge-&am gleichen Tag reparieren können. So, dass kein Ausfall von Schulbesuch stattfinden konnte (Interviewerin: Mhm (bejahend)). (Teilnehmer 8: Mhm (bejahend)) Oder, also damals war dann (Name eines Sanitätshauses, aus Datenschutzgründen ausgelassen) e-&existierte ja schon und (räuspert sich) aber die konnten das natürlich nicht reparieren, sondern man&man ist immer direkt, die haben sozusagen nur vermittelt, dass wir eben direkt von äh (Name eines Prothesenherstellers aus Datenschutzgründen ausgelassen) reparieren lassen konnten (Interviewerin: Mhm (bejahend)). #01:16:19-2#

Teilnehmer 8: Naja, ich hab‘ eine My-&einen Myo-Arm (Interviewerin: Ja.) (kurze Pause) den ich also ständig und unbedingt brauche, bis auf das, dass ich im Schwimmen nicht mit der Elektrik reingehe, weil sie sonst kaputt wär (Interviewerin: Mhm (bejahend)), ähm ich weiß natürlich, dass solche Arme auch schon 20 000-21 000 Euro kosten und ähm irgendwann hab ich dann diese Michelangelo-Hand bei (Name eines Prothesenherstellers aus Datenschutzgründen ausgelassen) in der ja Vorbereitung gesehen und wollte sie unbedingt haben und das ist jetzt meine Ersatzhand (Interviewerin: Mhm (bejahend)). Also im Moment trage ich wechselseitig Michelangelo und die Myoelektrik. Michelangelo hab‘ ich ja schon gesagt ein, zwei Nachteile (Interviewerin: Mhm (bejahend)) sind zum Beispiel, dass ich eben das Messer nicht so halten kann in dem Griff mhm (bejahend)) wie ich es mit der Myo-Hand kann und das andere: Sie ist wesentlich schwerer und wenn man den Akku aufladen muss, muss man immer die <u>ganze</u> Prothese hinstellen (Interviewerin: Mhm (bejahend)) und&und aufladen, während ich beim Myo-Arm nur den Akku in die Ladestation stecke. (Interviewerin: Und-) Ja. #01:17:43-2#

Interviewerin: Bevor Sie die Michelangelo-Hand hatten, da hatten Sie ja dann nur die (zeigt auf die Myoelektrik-Prothese, die der Teilnehmer trägt). Was war dann, wenn die- #01:17:41-8#

Teilnehmer 8: Ja, ich muss ganz ehrlich sagen als Kriegsbeschädigter bin ich <u>etwas</u> bevorzugt die ha-&ich hatte zwei Myo-Arme, (Interviewerin: Ok, ja.) die sich aber dann immer dann erübrigten, wenn ich unterschiedliche Gewichte hatte. (Interviewerin: Mhm (bejahend)). Wenn ich einen starken Arm hatte, dann war die Prothese nicht mehr so brauchbar (Interviewerin: Mhm (bejahend)). Ich konnte sie kaum mehr anziehen, weil die Elektroden nicht mehr stimmten und auch der Innenschaft zu eng wurde, ‘ne? Dann musste man einen neuen Innenschaft machen (Interviewerin: Mhm (bejahend)) und meistens wurde dann eine neue Prothese (ge-..?) und die Hand konnte man ja umstecken, also die Hand war von der einen Prothese zur andern verwendbar. (Interviewerin: Mhm (bejahend)) Das ist jetzt nicht mehr der Fall. Die Hand von der Myo passt nicht auf die Hand von der Michelangelo. #01:18:34-3#

Interviewerin: Ok, das heißt man muss einfach, wenn was ausfällt das Ganze- #01:18:38-6#

Teilnehmer 8: Da muss ich, ja, ja. (Interviewerin: Mhm (bejahend)) (Teilnehmer 1 räuspert sich) Und die Hand Michelangelo wird auch jährlich zur Überholung&Garantieüberholung zu (Name eines Prothesenherstellers aus Datenschutzgründen ausgelassen) geschickt. (Interviewerin: mhm (bejahend)) Über (Name eines Sanitätshauses, aus Datenschutzgründen ausgelassen). #01:18:58-9#

Interviewerin: Und bei Ihnen Frau (Name der Teilnehmerin 23 aus Datenschutzgründen

ausgelassen))? Sie haben ja gesagt, eben Sie gewöhnen sich erst dran, Sie nutzen Sie noch nicht so lange. Gibt’s denn irgendwelche Hilfsmittel die Sie Zuhause haben, wenn Sie eben keine Prothese tragen, die dann eine Unterstützung sind? Oder brauchen Sie sowas gar nicht (lachend)? #01:19:10-2#

Teilnehmerin 23: Ehrlich zu sein, brauche ich gar nicht (lachend) (Interviewerin: Ja.). Aber die ist relati-&relativ neu und wenn was bei der Armprothese kaputt geht, dann kommt die Firma (Name eines Prothesenherstellers aus Datenschutzgründen ausgelassen) nach (Name des Wohnorts aus Datenschutzgründen ausgelasse). #01:19:33-5#

Interviewerin: Ah, echt? #01:19:38-3#

Teilnehmerin 23: Ja, genau (lachend)! (Teilnehmer 8: Wow!) #01:19:37-6#

Interviewerin: Das ist Luxus! (lachend) #01:19:40-5#

Teilnehmerin 23: Letztes Mal war glaub ich, war so&war richtig ni-&hat sie nicht richtig funktioniert, oder da war was (Interviewerin: Mhm (bejahend)) aber er ist nach (Name des Wohnortsaus Datenschutzgründen ausgelassen) gekommen, hat sich das dann alles angeschaut und ja, war nichts kaputt (Interviewerin: Ja.). #01:19:51-4#

Interviewerin: Und bei den anderen, ähm (wendet sich an Teilnehmer 8) wenn Sie zum Beispiel sagen die Michelangelo funktioniert da nicht so gut, aber Sie müssen sie ja nutzen, gibt’s da irgendein Hilfsmittel, wie Sie vorhin gesagt haben dieses Messer. Haben Sie da noch mehr wo Sie sagen da mogeln Sie sich so ein bisschen durch und halt das- #01:20:03-8#

Teilnehmer 8: Dann muss ich mich, dann arbeite ich lieber ohne Prothese halt mit dem Stumpf irgendetwas feste und schneide das mit dem Messer (Interviewerin: Ja.). Dann äh wird’s für mich schwierig, dann in den Beruf noch zu gehen, was ich immer noch mache oder Sitzungen besuchen, tue ich einarmig mit dem Stumpf nicht mehr, das mach ich nicht (Interviewerin: Mhm (bejahend)). Ich wollt auch nur noch sagen äh bei (Name eines Prothesenherstellers aus Datenschutzgründen ausgelassen) also ich hätte mir natürlich ein bisschen mehr, wie soll ich sagen, Hilfen und Eingewöhnungsmöglichkeiten mit der Michelangelo versprochen. Dass ich da mehr erreiche, was man tatsächlich mit der Hand durchführen kann. Ich wurde ja dann doch ins kalte Wasser gestoßen, mit der Hand eben zurecht zu kommen, nachdem ich die Tests gemacht hatte, dann wurde die fertiggestellt, sie passt auch genau und gut (Interviewerin: Mhm (bejahend)) äh ich bringe auch alle Funktionen zum Tragen, aber wie gesagt, das musste ich mir alles sehr in Eigenarbeit an-&zulegen. Und wie gesagt mit dem Messer bin ich heute (Interviewerin: Mhm (bejahend)) nach wie vor im Unreinen weil ich einfach keine Möglichkeit seh‘ das wirklich festzuhalten. #01:21:08-5#

Interviewerin: Und Hilfen in welcher Form? Was hätten Sie sich da vorgestellt, was wäre da nötig gewesen? #01:21:15-3#

Teilnehmer 8: Also es gab mal so ein Brettchen mit dem man das Brot festklammern konnte und dann streichen konnte. Das Schwierigste ist zum Beispiel also das linke Hemdknöpfchen zuzumachen ist ja mit der Prothese unmöglich (Interviewerin: Mhm (bejahend)). Das muss ich dann über (drüberstüpfen?). Und Fingernägel zu feilen, da wären ein bisschen bessere, es gibt dazu Knipser, die man mit d-&mit dem Stumpf oder mit der Prothese anknipsen kann. Aber sie sind nicht sehr präzise und nicht sehr, sie könnten verbessert sein. (Interviewerin: Mhm (bejahend)) #01:22:08-1#

Teilnehmer 1: Sie haben keinen Knopflochverschließer? #01:22:07-7#

Teilnehmer 8: Nein? #01:22:09-1#

Teilnehmer 1: Ja, das ist ein fundamentales Instrument (alle lachen) was ich benutze (lachend). Das ist ein kleiner Stab der eine Drahtschlaufe hat, so klein und spitz zulaufend und eine kleine Öffnung haben (Interviewerin: Mhm (bejahend)) so also kann man sozusagen durchstecken durch das Knopfloch und dann den Knopf einhängen und wieder zurückziehen. Also das wird hier gefertigt, hier, bei Bedarf. #01:22:27-1#

Interviewerin: Gibt's da noch mehr von solchen Sachen, die man dann so in der Trickkiste hat? #01:22:27-6#

Teilnehmer 1: Mh ja, es gab f-&dann spezielle Löffel und so weiter, aber das hab ich dann alles nicht benu-. Das ist un-&unpraktisch für den täglichen Einsatz. #01:22:33-1#

Teilnehmer 8: Es gab mal eine Gabel, die an der Seite geschärft war (Teilnehmer 1 räuspert sich), so dass man sie als Messer verwenden konnte (Interviewerin: Mhm (bejahend)) aber nur begrenzt. Nicht? Also ein Schnitzel kann man damit nicht essen (alle lachen). #01:22:42-2#

Interviewerin: Und die Löffel? Was war an denen besonders, was hatten die- #01:22:46-6#

Teilnehmer 1: Die waren&der Griff war sozusagen erweitert, so dass der Griff hier in diese Öffnung passte (Zeigt auf die Öffnung zwischen Daumen und Zeigefinger der Prothese). (Interviewerin: Ja, mhm (bejahend)), sodass man&oder auch das übrige Besteck (Interviewerin: Mhm (bejahend)), so dass man eben besseren Griff hatte (Interviewerin: Ja) und das anpassen konnte an die Geometrie sozusagen. Aber (kurze Pause)- #01:23:03-4#

Teilnehmer 8: (öffnet und schließt seine Prothese) Das Geräusch ist schon auch schön, 'ne? Ihres ist leiser! #01:23:02-9#

Teilnehmer 1: Ja, ich hab' jetzt unterschiedliche. Also das ist jetzt 'n altes Modell was keine variable Geschwindigkeit hat, also nicht diese DMC Dynamic Mode Control. Die hab' ich auch, aber das ist im Moment nicht in Betrieb, sozusagen, weil ich irgendwie natürlich- #01:23:15-4#

Teilnehmer 8: Sie überfordern mich schon wieder. Ich kann nur langsam zu-, oh siehst du? Weil das nicht- #01:23:20-6#

Teilnehmer 1: Ham' se' nicht diese DMC Hand? #01:23:18-8#

Teilnehmer 8: Nee. #01:23:23-4#

(kurze Pause)
#01:23:24-7#

Teilnehmer 8: Nee, ich kann nur ganz auf. Mit Muskelbewegung das so steuern, dass es langsam geht. #01:23:36-7#

Teilnehmer 1: Ja, das ist natürlich das klassische Modell. #01:23:34-9#

Teilnehmer 8: Ja, DMC hab' ich wieder nicht erfahren. (Teilnehmer 1: Sollten Sie mal fragen!) Bin ich wieder völlig rückständig. #01:23:46-7#

Teilnehmer 1: Also die läuft schneller einfach. Das ist 'n natürlicheres Empfinden, wenn die (Interviewerin: Ja.) Hand schnell aufgeht und zugeht und wenn mal (..?). Aber die Steuerung funktioniert nur nicht so optimal wie ich mir das vorstellen könnte, aber im Prinzip ist das ein

guter Ansatz. #01:24:00-2#

Interviewerin: Ja. Und, wenn ich da jetzt einfach so rein höre ist es ja so ein bisschen: Scheinbar gibt's ja viele Sachen- #01:24:13-8#

Teilnehmer 8: Man weiß sie nicht. #01:24:13-8#

Interviewerin: Ok. #01:24:16-3#

Teilnehmer 8: Man erfährt sie nicht. #01:24:14-5#

Interviewerin: Ja. #01:24:17-4#

Teilnehmer 8: Wie gesagt, ich musste alles mit eigener Initiative zu Beispiel an der etwas schwierigeren Michelangelo-Hand mir aneignen, damit ich damit überhaupt da&die grun-&die grundsätzlichen Dinge erledige mit dieser Hand, nicht? Sie sollte ja piepsen und dann größer werden und äh so verschiedene Sachen. Das musste man alles über den Muskel&über den Armmuskel trainieren (Interviewerin: Mhm (bejahend)). Aber DMC hab' ich nicht. Hm. Hat mir keiner gesagt. #01:24:49-9#

Teilnehmer 1: Ja das is&die ist ers-&kann man diese Hand ersetzen einfach durch ein DMC-Modell. Das ist&erfordert keine weiteren Anpassungen. #01:24:55-4#

Teilnehmer 1: Ja. Nur die Hand kostet auch schon wieder. Aber ich bin ja fein raus, 'ne? Noch einmal, ich sag das noch einmal: Als Kriegsbeschädigter hab' ich die Möglichkeit das vom Versorgungsamt kostenlos gestellt zu kriegen, was für mich enorm wichtig ist. Und, wie soll ich sagen, die waren bisher auch sehr tolerant (Interviewerin: Mhm (bejahend)) und großzügig wenn ich das notwendig hatte, konnte ich es auch bekommen. (Interviewerin: Mhm (bejahend)). Wie gesagt, ich hatte oft Schriftwechsel, mit dem Hinweis, dass ich nicht unter der Lebensqualität leiden möchte und deswegen eine Michelangelo haben möchte und mit vielen Begründungen haben sie dann genehmigt. Ich weiß aber auch, dass die 51 000 Euro kostet. (Interviewerin: Mhm (bejahend)) #01:25:42-4#

Interviewerin: Mh, wenn wir da jetzt weitermachen, was wir eben gesagt haben. Wir haben so viel über die Nachteile gesprochen, über die Vorteile, auch darüber warum Sie sich dafür entschieden haben. Würd' ich Sie bitten, dass Sie sich einmal vorstellen, dass Sie an der Stelle der Entwickler sind, also dass Sie quasi die Möglichkeit hätten die Prothese die Sie haben, so zu machen, dass sie für Sie ideal ist. Und sicherlich doppeln sich jetzt ein paar Sachen, weil wir jetzt einfach die Themen durchgesprochen haben, aber das fände ich gar nicht schlecht, dass wir einfach nochmal da unterstreichen: Was sin-&was würden Sie zum Beispiel an den Funktionen verändern, was müsste sich da tun, damit Sie sagen könnten, das ist die ideale Prothese für mich? Die, die keinen Nachteil hat, mit der ich zufrieden bin? #01:26:27-7#

(kurze Pause) #01:26:27-7#

Interviewerin: Frau (Name der Teilnehmerin 23 aus Datenschutzgründen ausgelassen)? Weil Sie gesagt haben, dass Sie sich da so ein bisschen, dass Sie sich zwingen müssen das zu benutzen, dass es eben mit dieser Schulter, das haben Sie gesagt, aber: Was müsste die noch haben, damit Sie sie komplett überzeugt, damit sie sagen können: „Ja!“. #01:26:48-6#

Teilnehmerin 23: Also bei mir ist nur Problem dieses Schulter. Diese Schulter, wenn das ver-&wenn sich das verbessert schon, dass das so schön aussieht wie normale Schulter, (Interviewerin: Mhm (bejahend)) dann wäre das schon schön (lachend). Aber, also ja bei mir ist nur das Problem diese Schulter. #01:27:08-0#

Interviewerin: Ja. #01:27:10-1#

Teilnehmerin 23: Also mehr kann ich nicht meckern, an dieser Armprothese. #01:27:12-7#

Interviewerin: Und wenn Sie sich aber ganz frei raus wünschen dürften was die noch für ‘ne Funktion hat, was die&dass die noch irgendwas was Sie sich- #01:27:18-4#

Teilnehmerin 23: Dass die Finger bewegen sich auch alle. #01:27:21-9#

Interviewerin: Ok, dass es nicht nur dieses ist, (zeigt den Dreipunktgriff) (Teilnehmer 8: Mhm (bejahend)) sondern&sondern auch di- #01:27:23-6#

Teilnehmerin 23: Genau die a:::lle Finger bewegen sich. #01:27:26-1#

Interviewerin: Ja. Und ähm gibt’s bestimmte Bewegungen die Ihnen dann wichtig wären? Nur&also ist es nur das, dass sich die Finger bewegen, oder in einer bestimmten Situation, oder eine bestimmte- #01:27:34-8#

Teilnehmerin 23: Nee, also bei mir ist nur, dass die Finger sich bewegen und, dass das kann man in den Restaurant so richtig mit Messer und Gabel essen. #01:27:41-1#

Interviewerin: Ja. #01:27:42-6#

Teilnehmerin 23: Das ist das. #01:27:44-3#

Interviewerin: Und, aber es gibt ja jetzt einfach auch noch andere Situationen in denen man dieses Feinfühlige braucht. Wie Sie sagen im Restaurant mit Messer und Gabel. Wenn Sie sagen, Sie benutzen die im Haushalt, gibt’s da irgendwas wo Sie zum Beispiel sagen in der Küche, ode::r weiß nicht, bei irgendwelchen anderen Sachen wo sowas eben von Vorteil wäre, dass&dass die auch mit den Fingern so Bewegungen machen kann? Irgendeine bestimmte Situation? #01:27:58-9#

Teilnehmerin 23: Mhh. (kurze Pause) Mh, eigentlich nicht. #01:28:07-2#

Interviewerin: Ok, ja. Und bei den anderen? Herr (Name des Teilnehmer 1 aus Datenschutzgründen ausgelassen)? #01:28:08-6#

Teilnehmer 1: Also (räuspert sich) ich würde mir am meisten wünschen ähm, diese Steuerung der Prothese. Das Signal von den Muskeln, dass das besser ähm ge-&also besser funktionieren würde (Interviewerin: Mhm (bejahend)). So, dass ich unter allen Umständen ‘ne eindeutige Signalübertragung habe und ‘ne Trennung was&was sie damit bewirken, öffnen und schließen und möglicherweise auch eben halt bei der DMC ist es ja so, dass das die Geschwindigkeit abhängig ist von dem&von der Signalstärke also was von der&vom Muskel an Signal übertragen wird. Dass das eben problemlos funktioniert (Interviewerin: Mhm (bejahend)). Wenn ich jetzt mich bewege oder irgendwie unterschiedliche, wie gesagt ähm Temperaturen, Feuchtigkeit, das funktioniert nicht so, wie das eigentlich sein müsste. Das wäre der eigentlich mein Hauptwünsch an die Entwickler. (Interviewerin: Mhm (bejahend)) Dass das eindeutig funktioniert. #01:29:14-1#

Interviewerin: Das heißt könnte man das benennen, als einfach, dass die eben zuverlässig so die, die Sachen die sie eben machen kann, (Teilnehmer 1: Genau, genau.) dass das einfach zuverlässig passiert? #01:29:22-8#

Teilnehmer 1: Das ist absolut vorrangig. #01:29:28-2#

Interviewerin: Und aber, also das hat Vorrang, das verstehe ich. Aber wenn wir trotzdem nochmal weitergucken: wenn es noch eine Wunschfunktion gäbe, wo man&wo man sagt Sie dürfen sich da eine aussuchen, sie dürfen da eine dazu bauen, gäbe es da eine? So wie Sie vorher zum Beispiel gesagt haben mit dem Löffel, dass die sich- #01:29:42-3#

Teilnehmer 1: Ja, das wäre dann eben, die äh Positionierung der Hand, oder die-. Dass es vielleicht zwei, drei also wenige Grund-äh-einstellungen gibt, die sich unterscheiden. Also zum Beispiel, dass man eben halt, das ist ja bei der Michelangelo-Hand glaub ich der Fall, dass man eben halt diese Zeigefingerfunktion, dieses Zeigen mit dem Zeigefinger, dass man das hat. Oder dass man die äh dass man irgendwas ähm umfassen kann, da wo sich die Finger dann um den runden äh um das runde Element was man greift schließen sozusagen (Interviewerin: Mhm (bejahend)). #01:30:14-0#

Interviewerin: Und bei Ihnen (wendet sich an Teilnehmer 8)? #01:30:16-4#

Teilnehmer 8: Also- #01:30:20-1#

Teilnehmer 1: Also ich halte nich‘ so viel davon jetzt da irgendwelche Sensoren einzubauen, die einem viel Feedback geben, also ich glaube nicht, dass das so viel bringt aber (kurze Pause) ich hätte gerne diese Grundfunktionalitäten, dass die eben perfekt funktionieren (Interviewerin: Ja.) und nicht äh jetzt schon irgendwelche Zukunfts-äh-visionen realisieren, die dann unzureichend und auf der Basis der uns äh nich-&also das wobei die Grundfunktionen nicht hinreichend funktionieren (Interviewerin: Mhm (bejahend)). #01:30:45-5#

Interviewerin: Ja, sondern dass erstmal das, dieses Grundding- #01:30:47-0#

Teilnehmer 1: Ja, ich hab bei (Name eines Prothesenherstellers aus Datenschutzgründen ausgelassen) den voll&wenn ich in das Science Center gehe, da werden totale Visionen vorgestellt (Interviewerin: Mhm (bejahend)) und natürlich kann man damit viel Geld kriegen (Interviewerin: Mhm (bejahend)) wenn man verspricht das Blaue vom Himmel, was man eigentlich ein-äh-&realisieren möchte (Interviewerin: Mhm (bejahend)), aber f-&für den Benutzer am unteren Ende der möchte erstmal ‘ne vernünftige Grundfunktionalität haben die dann auch natürlich verlässlich funktioniert. #01:31:17-5#

Interviewerin: Ja. #01:31:17-5# #01:31:19-9#

Teilnehmer 8: Ja das kann ich bestätigen. Die Grundfunktionen müssen, ja, ständig und stets und wirklich funktionieren, die müssen vorhanden sein. Äh zusätzlich hätt‘ ich natürlich auch wunderbar, wenn sich die Finger bewegen lassen und man mit den Fingern einiges mehr tasten (Interviewerin: Mhm (bejahend)) und&und&und anfangen kann. (kurze Pause) Ähm das Drehgelenk wär ‘ne ganz wichtige Angelegenheit, wenn das wenigstens in geringem Ausmaß noch vorhanden wär, denn auch ich hab beim Essen natürlich Schwierigkeiten. Wenn ich das Messer hier hab, dann steht das in einer bestimmten Richtung, ich kann nicht verändern und nichts drehen. Das geht natürlich mit andern Geräten genauso. Im Haushalt gäbe es viele Funktionen, die man zusätzlich gebrauchen könnte. Denn- #01:32:17-9#

Interviewerin: Welche denn zum Beispiel? #01:32:17-9#

Teilnehmer 8: Ja ich hab‘ schon mal gesagt, man muss Gläser spülen. (Interviewerin: Mhm (bejahend)) Da muss man ja nun was tun. Ich hab‘ hier das Glas in der Hand und äh muss es irgendwie sauber kriegen. Man kann hier irgendetwas reinstecken, aber das ist alles sehr unbefriedigend (Interviewerin: Mhm (bejahend)) und nicht sehr gut gelöst. Das wär‘ zum Beispiel eine Sache beim Gläsern. In der Früh beim&bei ja Rasieren und so, das macht man mit einer Hand wahrscheinlich genauso, ich weiß nicht wie‘s Ihnen geht (wendet sich an

Teilnehmer 1)? #01:32:47-0#

Teilnehmer 1: Na ich nutze ‘n elektrischen Rasierapparat. Ich kann das mit&mit der Hand (Teilnehmer 8: Mit der Hand machen, ja.) als auch mit den beiden Armstümpfen sozusagen, das ist kein Problem. #01:32:55-7#

Teilnehmer 8: Hm. Ja gut, ich komme da mit einer Hand zurecht äh ähm (Interviewerin: Mhm (bejahend)) am Morgen. Wobei man gelegentlich auch am Rücken sich schrubben muss, was natürlich mit einer Hand nicht ganz so einfach ist, wenn man auf die linke Seite muss. Wenn man Linkshänder ist, hat man Schwierigkeiten (Teilnehmer 1: Ja, das-), ‘ne? Sich am Rücken zu äh&äh ja. Da mal&also jucken darf’s einen da nicht (alle lachen). Das ist dann schon ‘ne Schwierigkeit. Sonst sitzt man wieder auf ‘nem Baum und reibt sich den Rücken (Interviewerin: Mhm (bejahend)), um sich irgendwelche Hilfsmittel zu machen. Ja natürlich (kurze Pause). (Teilnehmer 1: Ja.) Das&das, wie gesagt, also dieses Drehgelenk, wenn man das noch entwickeln könnte (Interviewerin: Mhm (bejahend)), dass das über die Myoelektrik-Funktion, durch den Armstumpf bewegt werden kann, nicht mechanisch, indem ich da rübergreife. (Interviewerin: Mhm (bejahend)). Das ist mir einfach zu unmöglich und (kurze Pause) die Möglichkeit, dass man einen Teller hält und gleichzeitig sich noch was vom Tablett äh auflädt, das wär‘ eine wichtige Funktion weil ich muss immer wenn ich im Buffet bin irgendeinen erwischen, der mir den Teller hält (Teilnehmer 1: Genau.). Wenn da nichts zum Abstellen ist, nicht? Also dann muss&das ist eine unheimlich unangenehme Sache, vor allem wenn’s dann immer der Oberbürgermeister ist, der neben einem steht (alle lachen). #01:34:38-8#

Interviewerin: Aber kann ich da, dann noch kurz einhaken? #01:34:40-0#

Teilnehmer 8: Ja. #01:34:40-0#

Interviewerin: Wenn&wenn Sie sagen beim Buffet den einen Teller halten, mit der Hand trotzdem auftun. Wenn wir das technisch betrachten, was müsste die dann können, damit sowas funktionieren würde? #01:34:46-8#

Teilnehmer 8: Also wir haben das Drehgelenk (Interviewerin: Mhm (bejahend)), dann müsste sich d- (versucht es mit seiner Prothese zu zeigen) oh soweit geht’s schon gar nicht, weil der Handschuh da ist (Interviewerin: Mhm (bejahend)). #01:34:53-8#

Teilnehmer 1: Aber das sollte doch die Michelangelo-Hand k-&können? #01:34:57-1#

Teilnehmer 8: Die sollte es können, ja. (kurze Pause) Und dann eben so aufmachen (Interviewerin: Mhm (bejahend)), dass der Teller da drin liegt, aber auch bei der Michelangelo liegt er se:::hr wackelig und&und&und (Teilnehmer 1: Mhm (bejahend), ach so.) es ist also (Interviewerin: Mhm (bejahend)), es darf einen wirklich dann keiner anstoßen (Teilnehmer 1: Mhm (bejahend), ok.), dann ist der Teller weg (Interviewerin: Mhm (bejahend)). Ja? Und wie gesagt, hier geht’s jetzt gar nicht (zeigt auf seine Prothese), weil das würde ja nicht&nicht ausreichen, ja? (Interviewerin: Ja klar, ok.) #01:35:18-7#

Teilnehmer 1: Also ich- #01:35:20-5#

Teilnehmer 8: Ist übrigens eine Verbesserung bei der Michelan-&bei der Myoelektrik, dass man jetzt wirklich drehen kann (Interviewerin: Mhm (bejahend)). Früher in der war das Gelenk so, dass ich dann die Hand heraushatte (Interviewerin: Mhm (bejahend)), wenn ich zu viel gedreht habe. Das war natürlich unangenehm, wenn ich mit der Hand herumlief (alle lachen) und (Interviewerin: Ja, das kann ich mir vorstellen.) erst wieder mühsam aufstecken musste, wenn ich-, ‘ne? Da bin ich auch sehr zufrieden. Die Elektroden (kurze Pause) wenn die etwas verbessert werden können, dass die&die Auflagfläche nicht so ganz knapp gehalten ist, sonst funktioniert’s nicht. Es muss also sehr exakt aufliegen und jede kleine

Veränderung am Arm äh verschlechtert dann (Interviewerin: Mhm (bejahend)) die (kurze Pause) die&die&diese Elektrode, die Wirkung der El-&El&Elektrode. Ja. #01:36:21-8#

Teilnehmer 1: Ich hab‘ noch zwei Sachen. #01:36:24-6#

Interviewerin: Genau, ja Sie- #01:36:25-0#

Teilnehmer 1: Und zwar, einmal fände ich's gut, wenn die total wasserresistent wären (Interviewerin: Mhm (bejahend)). Also, dass man unter die Dusche gehen könnte. (Interviewerin: Mhm (bejahend)) Also ohne Prothesen kann ich zum Beispiel meinen Kopf ähm am Hinterkopf meine Haare nicht waschen (Interviewerin: Ja.). (Teilnehmer 8: Oh.) Weil ich mit den äh Stümpfen nicht dahinreiche und mit den Händen kann ich nicht unter die Dusche gehen, das ist-. #01:36:46-6#

Interviewerin: Ja. #01:36:46-6#

Teilnehmer 1: Dann noch ‘n anderer Punkt, der vielleicht doch wichtig ist, äh das Anziehen von einem Mantel. Das ist auch schwierig, weil man mit der Prothese nach hinten sozusagen dann den&das Ärmelloch finden muss (Interviewerin: Mhm (bejahend)) (Teilnehmer 8: Mhm (bejahend)) und wenn da natürlich ‘ne Sensorik möglich wäre, dass man spürt was&wo man grade ist, das wär natürlich ‘n Vorteil dann (Interviewerin: Ja.). Also so, da&das ist für mich echt ‘n Problem, ‘n Mantel anzuziehen. Und ‘ne andere Frage war noch, bei dieser äh, bei dieser Art von Prothese, ich hatte auch mal eine, die mit nicht porös war, da hatte ich das Problem, dass natürlich der Arm sich in der Prothese&im äh Sch-&Schaft bewegt und da Hohlräume entstehen, wo irgendwie dann auch immer äh irgendwelche Geräusche durch Luft-äh::-verdrängung (Teilnehmer 8: Ja!) entstehen. (Interviewerin: Mhm (bejahend)) Also das war sehr unangenehm, (Interviewerin: Mhm (bejahend)) also sowas, deshalb fand ich eben halt diese porö-&poröse Eigenschaft sehr gut. #01:37:38-7#

Teilnehmer 8: Ja. Das kann ich ergänzen, das ist besonders bei der Michelangelo-Hand so, dass man also wirklich Geräusche hat die klingen, als wenn man grad äh gepupst hätte (alle lachen) (Teilnehmer 1: Genau.) oder so. Äh das ist sehr unangenehm. Bei der Michelangelo gibt's einen Stöpsel, den man vorne draufmachen kann, aber er hat bei mir auch nicht allzu viel verbessert. Man steckt da ‘n Stöpsel drauf, damit die Luft nicht rausgeht, aber sie drückt sich an der Seite raus (Interviewerin: Mhm (bejahend)) und äh&äh macht dann diese Geräusche. #01:38:03-3#

Teilnehmer 1: Ja, das ist völlig unakzeptabel, finde ich. #01:38:04-9#

Teilnehmer 8: Ja, ja. #01:38:04-9#

Interviewerin: Mhm (bejahend). Und jetzt Herr (Name von Teilnehmer 8, aus Datenschutzgründen ausgelassen) hatten Sie vorher noch gesagt eben, dass es&dass Ihnen im Alltag ganz viel einfällt was da noch an Hilfsmittel nötig wär‘. Wir sind aber bei den Gläsern stehen geblieben. #01:38:14-8#

Teilnehmer 8: Stehen geblieben. Gläser, betrifft natürlich genauso Tassen, das ist natürlich sowieso klar. Ich muss mit der Prothese den Teller halten und kann ihn dann vielleicht abspülen (Interviewerin: Ja.), wenn ich nicht die Geschirrspülmaschine benütze. Die aber für ‘nen Ein-Personen-Haushalt nicht sehr rentabel ist. Ähm da wären natürlich Verbesserungen da, weil ich muss ja dann den Teller drehen und die Rückseite soll ja vom Teller auch gesäubert werden. #01:38:41-0#

Interviewerin: Ja. #01:38:41-0#

Teilnehmer 8: Das sind die einen Sachen. Äh (kurze Pause) noch schwieriger ist es, wenn man Pfannen oder schwerere Gelegen-&Ge-&Gegenstände äh säubern muss, das ist dann noch viel schwieriger. Was natürlich (kurze Pause) naja, wo ruf ich denn immer meine Frau? Wenn sie sagt ich soll mal die Betten frisch beziehen. Das schaffe ich nicht, auch mit der Prothese nicht, weil man kann die Seiten festhalten und rumdrehen, aber es ist sehr schwierig. Man muss da vielleicht doch, also wie gesagt, da ist wieder das Gelenk eigentlich gefordert, das ich ja nicht habe (Interviewerin: Mhm (bejahend)). Und äh da gibt es viele Beispiele. Also das mit dem Ding ist genauso äh von der Gartenarbeit will ich nicht sprechen, weil dann sind die Prothesen kaputt. (Interviewerin: Mhm (bejahend)). Sie müssen haltbar sein, stabil sein, so dass ich auch mal im Garten arbeiten kann zum Beispiel mit d-&dem Rasenmäher umgehe, der nicht elektrisch ist, sondern mit diesem normalen Rasenmäher, die ich bevorzuge, damit's nicht dauernd so 'n äh Lärm oder so 'n Kabelgewirr gibt. #01:39:52-8#

Teilnehmer 1: Sehr lobenswert. #01:39:54-8#

Interviewerin: Aber wenn wir da auch nochmal drauf gucken, eben was fehlt der Prothese, damit sie sowas könnte? Was wär' technisch ähm, was müsste man technisch verändern? #01:40:06-5#

Teilnehmer 8: Ja also da war die Dreh-&die Drehbewegung auf alle Fälle (Interviewerin: Genau, ja. Die viel löst.) wichtig. Die Fingerbewegungen sind auch sehr wichtig. Und (kurze Pause) wie ich jetzt festgestellt hab, dass ich einfacher die F-&das Gefühl zum&zum Schließen und Öffnen habe. #01:40:30-0#

Interviewerin: Ja. #01:40:31-0#

Teilnehmer 8: Dieses DMDC oder was noch diese- #01:40:34-4#

Teilnehmer 1: DMC #01:40:34-4#

Teilnehmer 8: DMC, 'ne? #01:40:35-8#

Interviewerin: Mhm (bejahend). #01:40:37-3#

Teilnehmer 8: Was er schon (gesa-?) (wendet sich an Teilnehmer 1), was es angeblich schon gibt, aber ich nicht habe. Und, um Gottes Willen diese Reinigung (Interviewerin: Mhm (bejahend)) und diese Verschmutzung ist unheimlich wichtig. Dass man nicht mit solchen schmutzigen (kurze Pause) und manchmal auch unhygienischen Dingen rumläuft. (kurze Pause) Weil ich weiß zum Beispiel auch, dass so ein Überzieher nicht unter 1000 Euro kostet (Interviewerin: Mhm (bejahend)). (kurze Pause) Nicht? #01:41:05-8#

Interviewerin: Dann würd' ich da gern nochmal 'n bisschen weitergehen. Und zwar haben Sie (wendet sich an Teilnehmer 1) das grade schon mal gesagt mit dem Mantel Anziehen und eben der Bewegung&äh mit der&dem Fühlen (Teilnehmer 1: Mhm (bejahend)), dass es halt irgendne' Art von Sensorik weiter(gibt?). Das ist ja 'ne Eigenschaft, die eben technisch Ihre Prothesen noch nicht können, dass man halt da (Teilnehmer 1: Genau.) irgendso'n Feedback bekommt. Da würde mich interessieren in was für Situationen ist das noch wichtig, dass&wo Ihnen das eben einfällt, ähm wo&wo's gut wäre sowas zu haben und w-&was für'ne Art von, eben ist das eher „Was macht meine Hand grade?“, dass ich da nicht hingucken muss, oder ist es das, dass man eben was wahrnimmt? #01:41:33-8#

Teilnehmer 1: Ja. (Teilnehmer 8: Genau.) Genau, alles wird&äh ich mein das ist natürlich kennzeichnend dafür, dass man alle Aktionen mit der Hand kontrolliert. (Interviewerin: Ja.) Das ist ja untypisch eigentlich. Weil viel, wenn ich auf der Straße gehe und irgendwie 'n Portemonnaie haben will, (Interviewerin: Mhm (bejahend)) dann muss ich gu-&hingucken

vor hinfassen (Interviewerin: Mhm (bejahend)) und so weiter. Das ma-&braucht man ja normalerweise nicht, man fühlt das. Also das ist schon sehr (kurze Pause) äh:: m großer Unterschied zum normalen Menschen. Aber (kurze Pause) dieses ja, das (kurze Pause) ich denke mal, dass s-&also die absolute Gleichwertigkeit nicht herstellbar ist. Aber vielleicht ersatzweise irgendne‘ rudimentäre Sensibilität in der Hand hat (Teilnehmer 8: Hm.). (kurze Pause) ich, äh ich hab‘ da keine konkrete Vorstellung. Also wenn ich jetzt mir&mich vor-&mir vorstelle mit dem Anziehen eines Mantels (Interviewerin: Mhm (bejahend)), (räuspert sich) (kurze Pause). Weiß nicht, wie das realisiert werden sollte. (Teilnehmer 8: Mhm (bejahend)). Aber (Interviewerin: Mhm (bejahend)) man müsste einfach ‘n gewisses Druckempfinden vielleicht realisieren. #01:42:48-3#

Interviewerin: Ja. #01:42:52-4#

Protokollant: Wo müsste das sein, das Druckempfinden? #01:42:57-3#

Teilnehmer 1: Es müsste irgendwie an der Oberfläche sein, finde ich. Also jetzt, wenn ich-. Damit könnte man auch wahrscheinlich (kurze Pause) äh ja-. Das w-&wäre vielleicht universell nutzbar in anderen Zusammenhängen, also einmal, wenn ich jetzt Widerstand spüre, dann müsste ich ‘n bisschen unterscheiden können: Wie ist dieser Widerstand auch. #01:43:24-6#

Teilnehmer 8: Mhm (bejahend) #01:43:24-6#

Teilnehmer 1: (Räuspert sich) Aber hab‘ ich mir jetzt nicht soweit Gedanken gemacht. (Teilnehmer 8: Aber, aber-) #01:43:28-1#

Teilnehmer 8: Aber du fühlst auch, wenn du hier irgendwas anfässt über den Armstumpf, ob du fester oder&oder äh- #01:43:35-1#

Teilnehmer 1: Nein, das mach ich nur über die Optik, also. #01:43:37-8#

Teilnehmer 8: Nur über die Optik? #01:43:35-9#

Teilnehmer 1: Also ich weiß natürlich, die Routine erlaubt es natürlich zu wissen, dass wenn ich hier was äh anfange ich über die mh, ich weiß wie lange ich sozusagen greifen muss, bis&bis die gewisse Differenz zu ist. Das ist ja- #01:43:51-9#

Teilnehmer 8: Aber du machst eleganter als ich. #01:43:54-4#

Interviewerin: Aber Herr (Name von Teilnehmer 8, aus Datenschutzgründen ausgelassen) heißt das, dass Sie eben- #01:43:55-8#

Teilnehmer 8: Ich muss es alles aus der Schulter machen, nicht? Wir sprechen wieder vom Drehgelenk. Ich kann eigentlich das Ding nur fassen und dann steht’s schon schräg (Interviewerin: Mhm (bejahend)), wenn ich’s also hinstellen will, muss ich’s also über die Schulter regulieren. Das geht nur in begrenztem Maße. #01:44:09-2#

Teilnehmer 1: Ja. #01:44:09-2#

Interviewerin: Aber wenn wir das jetzt nochmal zurück zu dem Gefühl gehen, das heißt Sie (wendet sich an Teilnehmer 8) nehmen quasi über den Armstumpf war, wie fest oder-, (Teilnehmer 8: Ja.) können Sie das nochmal genauer erklären? #01:44:14-5#

Teilnehmer 8: Also, wie soll ich ‘n das sagen? (kurze Pause) Ich fühl natürlich auch noch die Nerven innerhalb des Armstumpfs. Die brauch ich ja auch, um die Elektrode auszulösen und ich kann also das fühlen, dass das jetzt leichter (Interviewerin: Mhm (bejahend))

und&und&und äh nicht so fest zugeschlossen werden muss, wie zum Beispiel wenn ich so ‘ne Flasche nehme. #01:44:42-8#

Interviewerin: Ja. #01:44:44-0#

Teilnehmer 8: Nicht? Und zwar spür ich das, wie fest ich zudrücken kann. #01:44:50-6#

Teilnehmer 1: Mh, das ist interessant. Das ist bei mir nicht der Fall. #01:44:57-8#

Teilnehmer 8: Das heißt ich kann ‘n Tennisball nehmen, Tischtennisball, und kann ihn nicht zerdrücken. #01:45:06-4#

Interviewerin: Mhm (bejahend) #01:45:06-4#

Teilnehmer 8: Weil ich merke, weil ich spür, dass er&dass dann&also dass da nicht mehr Gewalt nötig ist (Interviewerin: Ja.), nicht mehr Kraft nötig ist. #01:45:17-2#

Interviewerin: Aber empfinden Sie das dann trotzdem auch so, dass es eben so wie Herr (Name von Teilnehmer 1, aus Datenschutzgründen ausgelassen) sagt, dass es in manchen Situationen einfach trotzdem-. Also Sie haben vorher gesagt, Sie können das Ei anfassen und machen’s nicht kaputt aber fallen Ihnen trotzdem Situationen ein, in denen es eben&in denen Sie das vermissen, dass es da irgendein Feedback gäbe. Dass man nicht hingucken oder- #01:45:38-9#

Teilnehmer 8: Naja, es&ich hab‘ ja gesagt (Interviewerin: Mhm (bejahend)) diese, wenn das natürlich zu schnell fließt oder irgendwie zu schnell die Kraft überträgt (Interviewerin: Ja.), dann hab ich die Möglichkeit nicht mehr das zu stoppen, dann zerdrückt es di-&da-&das Teil (Interviewerin: Mhm (bejahend)). Das war ja deswegen so interessant mit seinem, dem DMC, ‘ne? #01:45:54-8#

Teilnehmer 1: Genau. #01:45:54-8# #01:45:55-6#

Teilnehmer 8: Äh::m ja? #01:45:58-1#

Teilnehmer 1: Naja DMC ist eigentlich nur, sozusagen äh. Es gibt ‘ne Entwicklung die auch sozusagen es ermöglicht, hab ich gesehen, ähm sensitiv zu greifen. Also bei diesen Plastikbechern (Interviewerin: Ja.) (Teilnehmer 8: Ja.), das Beispiel, wenn man hält ‘n Plastikbecher im Flugzeug, man g-&gießt die Flüssigkeit rein, man muss sozusagen je mehr Flüssigkeit im V-&im Plastikbecher ist, muss man immer ‘n bisschen weiter nachjustieren. #01:46:15-8#

Interviewerin: Mhm (bejahend) #01:46:15-8#

Teilnehmer 1: Also das ist&das ist nicht DMC. DMC meint einfach nur, wenn ich ‘n s-&starkes Signal gebe mit meinem Muskel, dann läuft die Prothese ganz schnell. #01:46:23-9#

Teilnehmer 8: Mhm (bejahend) #01:46:23-9#

Teilnehmer 1: Und wenn ich ‘n (..?) mache, dann läuft sie langsam (Teilnehmer 8: Mhm (bejahend)). #01:46:28-0#

Teilnehmer 8: Also das muss feinfühliger werden. Das muss so sein, also dass man gleichmäßig spürt wie viel Druck man ausüben kann oder s-&soll, um das zu erreichen, was man haben will. #01:46:40-1#

Interviewerin: Also das man quasi zurückgemeldet bekommt, wie viel Kraft da grade (Teilnehmer 8: Ja, ja.) drin ist? #01:46:43-5#

Teilnehmer 8: Ja, wie viel Kraft da notwendig ist, um das in der richtigen Weise zu (Teilnehmer 1: Ja.) greifen. #01:46:48-8#

Teilnehmer 1: Ja, das wäre vielleicht ganz sinnvoll. Wenn man an einer Stelle so in diesem Punkt wo man den Griffkontakt hat, dass man da feststellt, wie stark man zugreift (Teilnehmer 8: Ja.), dass, dass man&das würde vielleicht doch irgendwie helfen. #01:47:03-5#

Interviewerin: Mhm (bejahend) #01:47:06-8#

Teilnehmer 8: Gegen Gewichtserleichterung hab‘ ich natürlich auch nichts einzuwenden. (kurze Pause) Wenn (die weniger Gewicht hätt‘?). (Interviewerin: Mhm (bejahend)). #01:47:17-8#

Interviewerin: Frau (Name der Teilnehmerin 23, aus Datenschutzgründen ausgelassen) fällt Ihnen zu dem Thema, dass man&dass man eben, dass man Rückmeldung von Hand bekommt, irgendne‘ Art Gefühl da drin hat, was ein wo sowas wichtig wär? Oder nicht so richtig? #01:47:34-1#

Teilnehmerin 23: Nee, nicht so wirklich, nee. #01:47:35-7#

Interviewerin: Ok, ja. #01:47:36-2#

Teilnehmer 8: Ja, (wendet sich an Teilnehmerin 23) das werden Sie noch erleben, wenn Sie’s öf-&öf-&öfters benutzen (Teilnehmerin 23: Genau, ja.) und dann, wie gesagt, jetzt wo Sie fragen (Interviewerin: Mhm (bejahend)), sind einem die Dinge nicht da, aber im Allgemeinen, im&i- (kurze Pause) wenn ich an meinen Beruf und meinen Job denke, natürlich (kurze Pause). Wie oft, ich musste Reden halten in der Stadt, dann hab‘ ich das Blatt hier rein getan, (demonstriert, wie er Blätter mit der Prothese hält) (Interviewerin: Mhm (bejahend)) vielleicht zehn Blätter, die ich da, damit ich mir da, da musst ich die Hand drehen, damit ich’s auch lesen konnte (Interviewerin: Ja.) und jetzt wenn ich aber das Blatt loslassen wollte, musste ich aufmachen. War ich nicht geschickt genug, waren&waren die Blätter am Boden und ich konnte meine Programme unten am Boden zusammen(suchen?). Also ich konnte die nicht so steuern, dass ich also hier nur ein Blatt aufmache. Ich kann’s natürlich jetzt so (Interviewerin: Mhm (bejahend)), aber das war ja schon sehr umständlich (Interviewerin: Mhm (bejahend)), das geht meistens schief. Und vor allen Dingen, ich hatte ja den Eindruck, dass ich frei reden möchte und nicht immer nur vom Blatt ablesen. #01:48:54-1#

Interviewerin: Aber nochmal kurz auf dieses Feedback, diese Rückmeldung, zurückzukommen, Sie haben jetzt gesagt, eben dass man vielleicht so ‘ne Art Druck wahrnimmt, oder den Widerstand gegen den man angeht. Gibt’s auch sowas, das Sie sagen, dass ähm dass manchmal fehlt, dass man ‘ne Temperatur wahrnimmt, oder, weiß nicht (Teilnehmer 1: Eigentlich nicht, eigentlich find ich wichtig-) ob einfach- #01:49:06-8#

Teilnehmer 1: Hab‘ ich jetzt äh also aus meiner Lebenserfahrung hab ich jetzt keine Situation erlebt, wo ich das jetzt für&also klar wenn man jetzt irgendwie ‘n Wasserhahn aufdreht und man weiß nicht, ist das Wasser zu heiß (Interviewerin: Mhm (bejahend)) (Teilnehmer 8: Mhm (bejahend)), oder kann man nur mit seiner Nase vielleicht drangehen (alle lachen). Aber, oder irgendwie durch andere Mechan-&Hilfsmittel. Aber jetzt, dass (räuspert sich), dass&d-&dass man großen Aufwand vielleicht Unter-&Untersuchungsaufwand oder Realisierungsaufwand äh erzeugt, um das zu realisieren (Interviewerin: Mhm (bejahend)), halte ich für nicht sinnvoll, oder- #01:49:51-9#

Interviewerin: Ja. (Teilnehmer 8: Mhm (bejahend)) #01:49:55-4#

Teilnehmer 8: Naja, beim Einhänder, ich probier's natürlich mit meiner normalen Hand und Sie (wendet sich an Teilnehmerin 23) wahrscheinlich auch (Teilnehmerin 23: Genau, bei mir ist auch so.) so. #01:50:05-0#

Interviewerin: Mhm (bejahend). #01:50:09-2#

Teilnehmer 8: Deswegen sag ich ja (wendet sich an Teilnehmer 1). Ich bewunder‘ Sie unwahrscheinlich, wie Sie da-&das klarmachen. (kurze Pause) #01:50:19-1#

Teilnehmer 1: Das ist nur reiner Selbsterhaltungstrieb. #01:50:22-8#

Teilnehmer 8: Ja, aber- #01:50:27-2#

Interviewerin: Ähm ja, wir sind tatsächlich, neigen wir uns langsam schon dem Ende zu (Teilnehmer 8: Mhm (bejahend)). Ähm ich würd‘ einmal nochmal kurz zusammenfassen: Wir haben über Vor- und Nachteile gesprochen, darüber warum Sie sich für die Prothese entschieden haben, was passiert wenn die ausfällt, wenn die kaputt geht, wann Sie sie nicht benutzen. Gibt's irgendwas, was Ihnen jetzt noch einfällt, wo Sie vielleicht vorher, weiß nicht, den Gedanken nicht hatten und Ihnen noch irgendwas einfällt zu den vorherigen Fragen, was Sie jetzt gerne noch ergänzen würden? #01:50:57-3#

(kurze Pause)
#01:50:59-0#

Teilnehmer 8: Also es ist klar, meine (kurze Pause) Ersatzhand muss genauso gut funktionieren, wie meine Originalhand. (Interviewerin: Ja.) Und äh, da man natürlich die Ersatzhand nicht so oft trägt, vielleicht 'n halbes Jahr gar nicht, oder 'n Jahr nicht (Interviewerin: Mhm (bejahend)), äh kann es einem passieren, dass man die Ersatzhand braucht (lachend) und benötigt (Interviewerin: Mhm (bejahend)) und dann ist nicht die Möglichkeit, damit umzugehen. Das ist mir schon passiert und wie man das abhilft weiß ich nicht. Es müsste halt öfters mal überprüft werden (kurze Pause) und es wäre ganz nett, wenn es sowas wie Sport- oder Bewegungstherapie geben würde. Weil wie gesagt, dass ich, ich der eigentlich viel Sport mache und alle möglichen Dinge tu, trotz allem Haltungsschäden aufweise (Interviewerin: Mhm (bejahend)), ‘ne? Durch die Prothese. Es ist eindeutig durch die Prothese verursacht. Anders ist das eigentlich nicht zu erklären (Interviewerin: Mhm (bejahend)), dass ma- #01:51:55-8#

Teilnehmer 1: Ich finde, es müsste ‘ne stärkere Bindung, oder 'n Zusammenhang zwischen den Hers-&Herstellern von Prothesen und den Nutzern äh existieren. Also ich hab' während meiner gesamten Zeit keinen Kontakt entsprechend zu (Name eines Prothesenherstellers aus Datenschutzgründen ausgelassen) gehabt. Oder kein Interesse auch von denen jetzt. #01:52:17-3#

Interviewerin: Mhm (bejahend). Also, dass es einfach irgendeinen Verbindungsweg gibt, wo man eben Rückmeldung geben kann (Teilnehmer 1: Genau.), oder- #01:52:26-0#

Teilnehmer 1: Genau, Austausch von Erfahrungen und&und Anforderungen und so weiter. #01:52:30-5#

Interviewerin: Mhm (bejahend) #01:52:30-5#

Teilnehmer 8: Ich schließ‘ mich voll und ganz an. #01:52:32-9#

Interviewerin: Mhm (bejahend) #01:52:34-0#

Teilnehmer 8: Allerdings nehmen Sie mich nicht zum Beispiel (…???). (Teilnehmer 1: Und eben als besonderer Punkt natürlich möchte ich immer wieder noch auf diese Touchpad-Geschichte hinweisen. Dass man das also wirklich als Punkt auch aufnimmt.) #01:52:47-2#

Interviewerin: Mhm (bejahend) #01:52:47-2#

Teilnehmer 1: Dass&ich weiß nicht, ob das sozusagen elektrotechnisch realisierbar ist, aber das wäre vielleicht (kurze Pause) ‘ne&‘ne Studie wert, oder für ‘ne Diplomarbeit oder was weiß ich, ‘ne B-&Bachelor. #01:53:05-6#

Interviewerin: Also das man einfach irgendeinen Touchscreen damit bedienen kann (…??) (Teilnehmer 1: Nein also man kennt ja diese, es gibt ja Stifte da sch-). Ja. (Teilnehmer 1: Aber diese Stifte funktionieren eben nur so, dass sie denn&denn die elektrische Leitfähigkeit der Haut eines normalen Menschen übertragen. Wenn ich so ’n Stift nehme zur&bringt das überhaupt nichts. Ich kann das nicht bed-, nee also. Es werden ja elektrostatische Eigenschaften benutzt. Es gibt zwar auch Techniken, wo druckempfindlich äh wird, aber das tritt immer weiter in&in den H-&Hintergrund, weil (kurze Pause) diese elektrostatische Eigenschaft ‘ne größere Anwendbarkeit bie-, wie man kann mit zwei Fingern auf der Oberfläche operieren zum groß machen und so weiter. Das ist bei den Grö-&bei den Druckempfindlichen nicht der Fall. Insofern.) #01:53:53-1#

Teilnehmer 1: Diese Hände, diese Handschuhe müssten eben halt im Prinzip ‘ne elektrische Leitfähigkeit vom Arm zu&zu äh haben. (Interviewerin: Mhm (bejahend)) So, dass sie eben halt das Potential weiterleiten. (kurze Pause) Mh ja. Ich hab‘ da schon viel gesurft im Internet und so weiter, Google hat mal geantwortet und hat ‘ne Empfehlung gegeben: Man kann das realisieren indem man ‘n elektrisch leitfähigen Stift hat, also irgendwas metallisches, und dann ‘ne große Antenne dran hat. Also ein Meter Draht oder sowas (Interviewerin: Mhm (bejahend)). Das würde&realisiert dann auch in gewisser Weise die&die&das Potential was man braucht, aber, das wäre natürlich schön, wenn man sowas in einen kleinen handlichen Stift packen könnte. #01:54:31-6#

Interviewerin: Ja. Frau (Name der Teilnehmerin 23 aus Datenschutzgründen ausgelassen), gibt’s bei Ihnen noch irgendwas, was Sie gerne hinzufügen würden? #01:54:38-1#

Teilnehmerin 23: Mh, nee. #01:54:36-1#

Interviewerin: Ok (lachend). #01:54:41-5#

Teilnehmer 1: Aber ich würde gerne hinzufügen: Wir haben vorhin von den Fingerbewegungen gesagt. Ich weiß zum Beispiel, dass es für mich unheimlich wichtig wär’, wenn ich jetzt den einen Finger nach oben stellen könnte. #01:54:48-3#

Interviewerin: Mhm (bejahend) #01:54:49-2#

Teilnehmer 1: Und damit beim Autofahren, weiß nicht genau, zum Beispiel eine Taste drücken könnte, zum Beispiel diese Schnellblinker da, ‘ne? D-&diese äh &äh Blinkanlage bedienen kann. Jedes Mal muss ich erst die Akrobatik machen, aber verraten Sie’s jetzt nicht, weil sonst ver-&verlangen die von mir, die&den Umbau. ‘Ne? Aber diese Finger, das gilt nicht nur für diesen einen Knopf, das gilt für viele andere Knöpfe die auf der rechten Seite sind, die eben nur dann zu bewegen sind, wenn ich einen Finger rausstrecken kann und nich‘ die ganzen vier, fünf Finger da habe, dann kann ich keinen Knopf einzeln an-&an-&angreifen. (Teilnehmer 1: Ja, das finde ich auch. Das kann ich unterstützen.). Nicht? #01:55:23-8#

Interviewerin: Also einfach nur nochmal, dass die Finger sich eben einzeln bewegen lassen? #01:55:27-2#

Teilnehmer 8: Einzeln bewegen ja, und ein Finger richtig zum Fingerzeig gehoben werden kann. #01:55:32-4#

Teilnehmer 1: Das ist ja auch wichtig, um ‘ne Tastatur zu bedienen. (Interviewerin: Ja.) Im Prinzip mit zwei Händen. #01:55:35-9#

Teilnehmer 8: Mhm (bejahend). Das wär’ schon wichtig. #01:55:41-1#

Interviewerin: Gibt’s sonst noch irgendwas, fällt Ihnen noch was ein (lachend)? #01:55:44-0#

Teilnehmer 8: Beim nächsten Mal fällt mir noch etliches ein. Ich hätt’&ich wollt mir’s auch notieren, weil ich schon geahnt hab, dass ihr mir solche Fragen stellt. #01:55:58-3#

Interviewerin: Ok, aber wenn’s jetzt grade erstmal keine mehr gibt, dann würd‘ ich äh das Gespräch erstmal jetzt hier für beendet erklären ähm und mich ganz herzlich bedanken. Wie:: ich vorher gesagt hab’, gibt’s den Fragebogen noch. Den würde ich Ihnen jetzt einmal nochmal austeilen, dass Sie den nochmal ausfüllen.
