# Supplementary material for: What do users and their aiding professionals want from future devices in upper limb prosthetics? A focus group study
Source: PLoS One. 2023 Dec 29;18(12):e0295516. doi: 10.1371/journal.pone.0295516 (PMC10756510; doi:10.1371/journal.pone.0295516)
Supplement: S1 Appendix — (ZIP) [file pone.0295516.s001.zip › FocusGroup_Transcripts/FGP2.pdf]

Interviewer: Ok. Und dann legen wir los mit der ersten Frage. Wenn ich die stell, würd ich sie bitten, dass wir einmal hier bei Ihnen starten. Und ähm einfach einmal der Reihe der nach durchgehen. Damit ich einmal zu jedem Namen ähm einfach was Gesagtes hab, die Stimme danach sp-, leichter identifizieren kann. Und würd Sie bitten, dass Sie sich zu Beginn einmal alle vorstellen. Und dann danach bei den weiteren Fragen dürfen Sie ganz frei antworten, so wie es Ihnen gefällt. Wer möchte darf sprechen. Ähm ich würd Sie äh zu Beginn bitten, dass Sie sich vorstellen, dass Sie als jemand der ‚ne Prothese nutzt ´n anderen Menschen treffen der eine trägt. Und dann würde ich äh gerne von Ihnen wissen, was Ihnen da als erstes auffällt. Was, auf was Sie als erstes achten, wenn Sie einen anderen Menschen sehen, der auch `ne Prothese trägt und nutzt. #00:02:16-4#

Teilnehmerin 1: Wenn ich jemand treffe, (..?) auf ‚ne Prothese trägt (Interviewer: mhm (bejahend), ja.). Gute Frage (lachend). Da hab ich gar keine Antwort drauf. Ich achte eigentlich gar nicht auf ne Prothese (Interviewer: mhm (bejahend)). Weil am besten ist die Prothese, die nicht auffällt. #00:02:25-2#

Interviewer: Ja. #00:02:28-7#

Teilnehmerin 1: Das ist halt einfach so. #00:02:29-7#

Interviewer: Und können Sie sagen welche Prothese auffallen würde. Und welche nicht auffällt. Also wann's, wann die Prothese- #00:02:35-7#

Teilnehmerin 1: Meistens (Interviewer: mhm (bejahend)) fällt der Träger auf. Das heißt mir fällt das also nur auf, dass viele diese typische Prothesenhaltung haben. Schiefe Schulter. (Interviewer: mhm (bejahend)) Verdickte Körperhaltung. Äh die Prothese an sich ist gar nicht das Entscheidende (Interviewer: mhm (bejahend)). Sondern de Körperhaltung eigentlich. (Interviewer: mhm (bejahend)) #00:02:50-9#

Interviewer: Und was ist ‚ne verdickte Körperhaltung? #00:02:52-8#

Teilnehmerin 1: Also viele Leute stehen so diese typische (Interviewer: mhm (bejahend)) Amputiertenhaltung. Eine Schulter hochgezogen extrem (Interviewer: Ok.). (Interviewer: mhm (bejahend)) Ähm stehen an sich krumm. Und das fällt mir also deutlich mehr auf als ob der ‚ne Prothese trägt oder nicht. #00:03:04-2#

Interviewer: Ja, ok. Danke schön. #00:03:05-6#

Teilnehmerin 1: Das sind also so die wichtigsten (Interviewer: mhm (bejahend)) Geschichten. Weil die Prothese an sich ist immer die gleiche. #00:03:11-8#

Interviewer: Ja, klar. Und Sie? Ach so, können Sie nachträglich noch Ihren Namen einmal sagen, das w- #00:03:15-7#

Teilnehmerin 1: (Name aus Datenschutzgründen ausgelassen). #00:03:17-7#

Interviewer: Danke. #00:03:18-6#

Teilnehmerin 1: Bitte (lachend). Jetzt du (an Teilnehmer 2 gerichtet) #00:03:19-1#

Teilnehmer 2: mhm (bejahend). Also ich bin (Name aus Datenschutzgründen ausgelassen). (Interviewer: mhm (bejahend)) Ähm ja, ich sag dann, ich achte da auch nicht so sehr drauf. Also meist, also kommt auch nicht so oft vor, dass man jemand trifft. (Interviewer: mhm (bejahend)) Also so jetzt in dieser Runde vielleicht hier mal. Oder wenn man hier ähm zum Service ist oder wie auch immer. (Interviewer: Ja.) Trifft man hier vielleicht mal jemanden (Interviewer: mhm (bejahend)). Aber sonst, achte ich da ehrlich gesagt auch nicht so drauf. Wie mein Vorredner schon gesagt hat, also. (..??) Körperhaltung vielleicht, also. (Interviewer: mhm (bejahend)) Manche Leute verstecken's auch einfach. Ich gehe da relativ frei mit um. (Interviewer: mhm (bejahend)) Und zeig die auch. #00:03:55-7#

Interviewer: Ja. Und gibt's aber irgendwas, dass man irgendwas vergleicht? Wenn man zum Beispiel hierherkommt. Und hier jemanden trifft, den man davor noch nicht getroffen hat? #00:04:03-4#

Teilnehmer 2: Ja klar, man hat eben-. Vorne hab ich das selber gemerkt. Wie gesagt, so oft trifft man keinen. (Interviewer: mhm (bejahend)) Und dann hat man sich mal eben schon mal ausgetauscht. Hier, was hast du für ‚ne Hand? Ähm was ist das für'n Modell? Oder (Interviewer: Ja) wie auch immer. #00:04:16-1#

Interviewer: Und mit welchem Zweck macht man das? #00:04:18-4#

Teilnehmer 2: Ja um zu gucken was, was hat man selber. Was hat der andere. (Interviewer: Ja.) Gibt's vielelicht Verbesserungsmöglichkeiten, was anderes. (Interviewer: mhm (bejahend)) Wie kommt derjenige damit klar. (Interviewer: Ja.) #00:04:30-1#

Teilnehmerin 3: (Name aus Datenschutzgründen ausgelassen) ist mein Name. Also wie schon gesagt jetzt hier, wie der (Name von Teilnehmerin 1) schon gesagt hat, was (..??) auffallen würd's (Interviewer: mhm (bejahend)) mir jetzt auch nicht so. Außer wegen der Körperhaltung (Interviewer: mhm (bejahend)). Ansonsten so wie jetzt hier wenn (..?) jetzt oder äh Reparatur bin und so. Dann achte ich immer auf'n Schaft irgendwie. Was hat er für'n Motiv. So oh, was hat der (Interviewer: Ja.). Wie der Kollege schon (neben Ihnen?) (Interviewer: Ja.) gesagt hat. Aber ansonsten gibt's da jetzt im Moment nicht so. #00:04:54-0#

Interviewer: mhm (bejahend). Also es ist auch nicht schlimm, wenn sich die Antworten doppeln. Einfach, mich interessiert's, was jeder Einzelne von Ihnen denkt. Und es ist auch interessant wenn jeder das gleiche denkt. Ähm weil es natürlich dann das Bild verstärkt. Also gerne, es ist nicht schlimm, wenn sich Antworten wiederholen. Ja. #00:05:10-1#

Teilnehmer 4: Ja, (Name aus Datenschutzgründen ausgelassen) ist mein Name. (Interviewer: mhm (bejahend)) Was mir so auffällt, die Farbe sofort. Wenn ich eine Person treffe (Interviewer: Ja.) die eine Prothese trägt, (..?) Farbe fällt sofort auf. (Interviewer: Ok.) Und habe ich sofort Gedanken, warum kann man das einfach nicht kopieren. (Interviewer: mhm (bejahend)) (..??) (Interviewer: mhm (bejahend)) Farbe Kosmetikhandschuh (Interviewer: mhm (bejahend)) (..??). Ja, Körperhaltung natürlich auch, fällt sofort auf. (Interviewer: Ja.) Nicht bei jedem. (..??). #00:05:35-6#

Interviewer: Ja. Aber das heißt Ihnen ist nicht nur die Form, oder die Größe, sondern vor allem die Farbe ist das was wichtig ist? #00:05:40-4#

Teilnehmer 4: Ja. Vor allem die Farbe (..??). (Interviewer: mhm (bejahend)) Ich habe auch so ein Beruf, dass ich lieber nicht (..??). (Interviewer: mhm (bejahend)) Deswegen (..??) Aber die Farbe verrät das. #00:05:54-6#

Interviewer: Ja, ok. mhm (bejahend) #00:05:58-8#

Teilnehmer 4: Ansonsten (..?) wenn man sich mit Kollegen trifft, dann austauschen (.??) (Interviewer: mhm (bejahend)) (..???) (Interviewer: Ja.) Was mir persönlich fehlt, dass man gleichzeitig nicht, ähm naja zwei (Arbeitsschritte?) machen kann. #00:06:16-9#

Interviewer: mhm (bejahend) #00:06:17-6#

Teilnehmer 4: Zum Beispiel aufmachen und drehen gleichzeitig (Interviewer: mhm (bejahend), ja.). Man muss immer schalten und das hinkt und die Geschwindigkeit (…??) #00:06:23-0#

Interviewer: Da würde ich Sie bitten, dass Sie das sich merken. Weil da kom-, dazu kommen wir nachher nochmal. Das Sie das nochmal genauer erklären. (Teilnehmer 4: Ja.) Was da genau fehlt. mhm (bejahend), Danke schön. #00:06:30-2#

Teilnehmer 4: (..?) #00:06:32-3#

Teilnehmer 5: Ja, (Name aus Datenschutzgründen ausgelassen) ist mein Name. Ähm ich schlage auch in die gleiche Kerbe ein, wie hier viele der Vorredner (Interviewer: mhm (bejahend)). Das vor allen Dingen Ästhetik ähm besonders wichtig ist. Mh auch (..?) ich hab noch nie davor (..?) die eine Unterarmprothese tragen. (Interviewer: mhm (bejahend)) Von daher hab ich erstmal äh so wie ich’s äh eigentlich unangenehm finde, wenn’s bei mri gemacht wird (Interviewer: (lacht)), dass man die ganze Zeit auf meinen Unterarm starrt. Hab ich das heute bei allen anderen gemacht. (Interviewer: Ja.) Das heißt ihre Unterarme ganz genau angeguckt. Und ähm die Optik mir angeguckt. Und ähm angeschaut wie ist der Handschuh angepasst. (Interviewer: mhm (bejahend)). Äh ist der lang, ist der kurz. Wie ist die Farbe? Geht’s über’n Ellbogen hinaus oder nicht (Interviewer: Ja.). Und das war einfach spannend das zu sehen. Wird ‚ne Uhr getragen oder nicht? Und äh, äh das sind einfach, das sind einfach Möglichkeiten (alle lachen), die ähm, ja die nochmal zeigen was, was für Möglichkeiten ich auch habe. (Interviewer: Ja.) Und was ich vielleicht noch nicht genutzt habe. Und was wir mal ausprobierne könnten. (Interviewer: Ja.) Ähm also bis vor äh einem Jahr habe ich zum Beispiel keine ähm kurzen T-shirts getragen. (Interviewer: mhm (bejahend)) Weil ich auch wollte, dass das verdeckt wird. Ja? Und ähm von daher äh achte ich darauf bei anderen (Interviewer: mhm (bejahend)) wie das getragen wird. Jetzt sehe ich, dass andere das auch so tragen. Und äh das (..?) einfach, ja, bestärkt (..?) Entscheidung zu sagen (Interviewer: mhm (bejahend)) ich trag’s jetzt so. Und äh fühl mich da wohl mit. #00:07:36-1#

Interviewer: Ja. Und da, wäre noch eine Frage, ist eben das worauf man bei der Ästhetik achtet eher so wie machen das die anderen und kann ich das auch so

machen? Dass man da bestärkt wird. Oder ist es schon sowas, dass man halt sagt was ist noch an der, was bei mir vielleicht ausbaufähig wäre, wo ich dann zum Techniker gehen kann und sagen kann hier das möchte ich eigentlich auch so, das habe ich besser gesehen? #00:07:54-7#

Teilnehmer 5: Mh das ist, also das is zweierlei. (Interviewer: mhm (bejahend)) Ich glaub einmal ist es tatsächlich die, die (Interviewer: mhm (bejahend)) Ästhetik. Wie, wie machen andere das? Was könnte ich kopieren? (Interviewer: Ja.) Was möchte ich überhaupt nicht machen. Und die zweite Seite ist glaube ich, welche Funktionen hat auch die Prothese. (Interviewer: mhm (bejahend)) Wie bewegt die sich irgendwie. Wie wird das Ganze ins Körperbild eingebaut, ne? (Interviewer: Ja, mhm (bejahend)) Das ähm ist auch interessant. Hat das dann mit der Prothese wiederum zu tun. Und mit den Funktionen, die die Prothese hat. (Interviewer: mhm (bejahend)) Es gibt ja ganz viele unterschiedliche Prothesen. Die uns hier auch schon angeboten wurden. Und ähm gab's dann natürlich auch die Frage, habe ich da die Richtige? Oder die für mich am besten (Interviewer: Ja.) passende (Interviewer: mhm (bejahend)) Prothese ausgewählt schon. Oder gibt's noch eine, die Funktionen hat, die:: äh ich noch besser in meinem beruflichen oder privanten Alltag dann integriegen kann. #00:08:30-0#

Interviewer: mhm (bejahend), ja. Okay. Danke schön. #00:08:32-0#

Teilnehmer 6: Ja, mein Name ist (Name aus Datenschutzgründen ausgelassen). Ähm ich erkenne meistens die Prothesenträger direkt an ihrer Hal-, äh Körperhaltung (Kind von Teilnehmer Nr. 5: Guck mal (Name aus Datenschutzgründen ausgelassen), ein Auto!) (..??). Zu sprechen, weil ich nicht weiß wie der gegenüber reagiert. Ähm (..?) zufällig noch ein paar (..??) mit ,nem Kollegen zusammen (Interviewer: mhm (bejahend)). Ins Gespräch gekommen. (Interviewer: Ja.) Seitdem (..?) hin und wieder mal Kontakt. Und äh ja. Also (..?) das ich dann so viele (Interviewer: mhm (bejahend)) Prothesenträger immer treffe, das (..?). Weil was ganz toll ist (Interviewer: Ja.) ich führe ganz, ganz (..??) macht. Weil (..?) schön austasuchen. #00:09:12-7#

Interviewer: Ja. Aber das heißt wo, also was Ihnen dann als erstes aufgefallen ist, ist die Körperhaltung? Oder das wobei man, (..?) (Teilnehmer 6: „Dadurch seh ich's am Ende einfach am schnellsten.) Ja, okay. #00:09:21-6#

Teilnehmer 6: Weil ich bin zum Beispiel Schulter Ex und (..??) (Interviewer: mhm (bejahend)) und ja. T-shirts trag ich seit, seit zwei Jahren. (Interviewer: mhm (bejahend)) Früher hab ich nur langärmlige Hemden getragen, hochgekrempelt. #00:09:36-1#

Interviewer: Das heißt das ist schon aber auch was, dass, dass es n Prozess ist eben sagen zu können man zeigt es. Und das man erstmal wollte, dass es unauffällig ist? #00:09:43-0#

Teilnehmer 6: Ja. #00:09:43-3#

Interviewer: Okay, ja. #00:09:44-5#

Teilnehmer 6: Weil man, (..??) wie siehen (..?) gegenüber mich? #00:09:48-1#

Interviewer: mhm (bejahend) #00:09:48-7#

Teilnehmer 6: (Kind spricht im Hintergrund) Wenn ich eben unterwegs bin. (Interviewer: mhm (bejahend)). Die halten das (..??) #00:09:56-4#

Interviewer: Okay, Danke schön. (Durcheinander sprechen im Hintergrund) Okay. Dann würde ich gerne ähm als nächstes wissen, Sie haben's gerade schon gesagt Herr (Name von Teilnehmer 5 aus Datenschutzgründen ausgelassen), dass verschiedene Prothesentypen angeboten wurden. Warum Sie sich denn grade für Ihre entschieden haben? Also was war ausschlaggebend daran, warum nicht für eine andere? War es überhaupt Ihre Entscheidung? Können Sie darauf eine Antwort geben? Also wer möchte, diesmal nicht mehr in der Reihenfolge. Sondern wer möchte darf erzählen. #00:10:25-1#

Teilnehmer 6 (?): Also ich habe meine jetzige (.?) (Interviewer: mhm (bejahend)) also jetzigen Aufbau bei (Name eines Prothesenherstellers aus Datenschutzgründen ausgelassen) herstellen lassen. (Interviewer: mhm (bejahend)) So wie's jetzt ist. Ähm und Anforderungen waren an (Name eines Prothesenherstellers aus Datenschutzgründen ausgelassen) gestellt worden äh ich bin Maschinenbauzeichner. Ich muss ,ne, ,ne Zeichenmaschine bewältigen (Interviewer: mhm (bejahend)). Und und mit arbeiten. Und muss mich auch zwischendurch festhalten. Und dadurch bin ich auf die vielen manuellen (..?) gekommen. Weil ich, man arretieren kann. (Interviewer: mhm (bejahend)) Die Hand drehen kann, öffnen kann, greifen kann (Interviewer: mhm (bejahend), ja.) und was machen kann. (Interviewer: Ja.) So. Und das war für mich, viel entscheidender, als diese ganzen elektrischen äh Schn-, äh Schnickschnack. (Interviewer: mhm (bejahend)) Weil die mir da viel zu feinfühlig ist und viel zu viel kaputtgeht. (Interviewer: mhm (bejahend)) #00:11:17-7#

Interviewer: Ok, ja. Das heißt das war quasi angepasst an der Beruf und was sie dann zu können hat? #00:11:21-0#

Teilnehmer 6: Ja, genau. #00:11:23-2#

Interviewer: Wie war das bei den anderen? (Lärm im Hingergrund) #00:11:26-1#

Teilnehmerin 3: Also bei mir war das jetzt so, ähm ich bin jetzt in ner Firma. Bin Staplerfahrer. (Interviewer: mhm (bejahend)) Dann hab ich auch die Speedhand von (Name eines Prothesenherstellers aus Datenschutzgründen ausgelassen) genommen. Und die ist äh schnell. (Interviewer: Ja.) Und robust. (Interviewer: Ja.) Und wenn man in der Firma ist halt, äh muss man halt, ich muss jetzt auch-. Ich fahr ja Stapler, hab jetzt nur den Joystick hier. Fahr die Gabel hoch und runter (Interviewer: mhm (bejahend)) und muss dran denken, (Lärm im Hintergrund) (..??) Ich brauch keine, keine Hand die halt mehr Funktionen hat. #00:11:50-9#

Interviewer: mhm (bejahend). Aber das heißt auch, die Entscheidung war quasi basierend darauf: „Was brauch ich für den Job?"? #00:11:54-4#

Teilnehmerin 3: Job. Genau. #00:11:55-2#

Interviewer: Okay. #00:11:57-4#

Teilnehmerin 3: Erstmal muss ich den Job, erstmal wieder arbeiten. Und der Rest kommt von alleine. (Zustimmendes Raunen) #00:11:59-9#

Interviewer: Ja. #00:12:00-3# #00:12:01-0#

Teilnehmerin 3: Also ich hab ne al-, ne Bebionic Hand zuhause (Interviewer: mhm (bejahend)) und die nehme ich dann halt äh für's zuhause. Also für privat. #00:12:07-5#

Interviewer: Okay. #00:12:07-5#

Teilnehmerin 3: Also das ist ‚ne Arbeitshand. Und ich hab eine für zuhause noch. Also (Krankenkasse?) (Interviewer: mhm (bejahend)) #00:12:14-3#

Interviewer: Und warum dann aber für zuhause ne, die andere? Also- #00:12:15-8#

Teilnehmerin 3: Ja, die ist filigraner. Man kann (Interviewer: mhm (bejahend)) halt n vernünftigen Stift nehmen. Mann kann auch vernünftig damit essen. Ne Gabel nehmen. (Interviewer: Okay.) Also man kann dann halt mehr Funktionen hat man dann (Interviewer: Ja.). Man kann auch nem Hobby nachgehen. #00:12:24-7#

Interviewer: Ja, aber das heißt, dass eben die andere Hand, das ist auch wieder nach den Funktionen die sie kann und nicht zum Beispiel das Aussehen? Das in erster Linie ist das was sie kann? #00:12:32-1#

Teilnehmerin 3: Erst ist die Funktion, ja. #00:12:34-7#

Interviewer: mhm (bejahend), okay. Und bei den anderen? Gibt's da noch irgendwas hinzuzufügen? #00:12:37-9#

Teilnehmerin 1: Also grundsätzlich ist es glaub ich äh in erster Linie muss man die Funktion seiner Hand steuern. Weil es gibt halt noch nichts, es gibt mein System am Markt wo man sagen kann äh was mir persönlich n deutlichen Vorteil bringt. Es gibt einfach nichts. Weil eine Standardhand, auf, zu äh ist ausreichend. Sagen wir's mal so. (Interviewer: mhm (bejahend)) Äh das, das Problem ist ja glaube ich die Steuerung. Nach wie vor. (Interviewer: mhm (bejahend)) Äh man würde natürlich gerne, ich hab das ja grade auch schon gehört bei dem Kollegen, mehrere Funktionen gleichzeitig machen. Ich hab also auch schon, ich weiß nicht was alles, probiert. (Name vom Orthopädietechniker) wird das wissen. (Interviewer: mhm (bejahend)) Äh aber ich hab noch kein, noch keine Lösung gefunden, die für mich persönlich die Beste ist. (Interviewer: Ja.) So, das heißt für mich ist also entscheidend: Funktionalität und Holt-, Haltbarkeit. (Interviewer: mhm (bejahend)) Weil ich auch keine Lust mehr habe, äh ich bin jetzt 17 Jahre Prothesenträger, einmal die Woche zu meinem Orthopädietechniker zu fahren. (Interviewer: Ja.) Und irgendwas reparieren zu lassen. Deswegen die möglichst einfach Lösung. #00:13:37-7#

Interviewer: Okay. Das heißt das, an der ersten Stelle stand, dass sie halt hält. #00:13:39-8#

Teilnehmerin 1: Ja. #00:13:37-8#

Interviewer: Dass das einfach funktioniert? #00:13:41-2#

Teilnehmerin 1: Funktionieren muss sie und halten muss sie. (Interviewer: mhm (bejahend)) Weil die Problematik ist, dass der ganze Krempel-. Je filigraner der wird, je feinfühliger der wird, je mehr Funktionen ich habe, desto mehr geht kaputt (Interviewer: mhm (bejahend). okay.) Und deswegen hab ich mich also immer rückgerüstet. Ich hab also schon deutlich besser aufgerüstete Prothesen gehabt (Interviewer: mhm (bejahend)), mit ganz vielen Stellmotoren. Mit Handdrehung. Mit allem möglichen Käse. Und hab also alles wieder, back to basic (lacht). #00:14:06-5#

Interviewer: Okay. Das heißt Sie sagen auch, dass die Haltbarkeit wichtiger ist als der Funktionsumfang? Dass Sie lieber weniger Funktionen hat aber eben hält? #00:14:13-0#

Teilnehmerin 1: Ich hab noch keine wir-, ich hab noch keine Möglichkeit gefunden, dass ich die vielen Funktionen die verschiedene Hände hat, einfach wie (..??) für mich nutzen kann. (Interviewer: mhm (bejahend)) Weil (Interviewer: Ja.) ich hatte immer das Problem, dass ich nur eine Auf- und eine Zubewegung machen kann. Mehr geht halt einfach nicht. (Interviewer: mhm (bejahend)) Und wenn ich jetzt irgendwelche weiteren äh Geschichten nutze, muss ich halt immer umschalten. Und diese ganze Umschaltung dauert viel, dauert lange. Dauert viel äh (Interviewer: mhm (bejahend)) und lenkt ab. Das ist das große Problem. (Interviewer: Ja.) #00:14:37-2#

Interviewer: Ja. #00:14:39-2#

Teilnehmer 6?: Also unserer Zeit habe ich das mit meiner norma-, mit meiner gesunden Hand längst erledigt. (Interviewer: mhm (bejahend)) (Teilnehmer Nr. 1: Ja.) Wenn ich aber, ich sag mal so, an der Kasse stehe und ich zieh mein Portmonnaie raus. Und (..?) hingestellt, (Interviewer: mhm (bejahend)) dann kann ich schon schnell bezahlen. (Interviewer: Ja.) Dann kann man die Karte rausholen (Lärm im Hintergrund) (..???). Braucht man (..?) auf und zu. (Interviewer: Ja.) Meine Prothese wird rein, also privat genutzt, als Gegenhalter (Interviewer: Ja.), als Festhalter (Interviewer: Ja, mhm (bejahend)). Er hält, sie hält was und dann ist gut. (Interviewer: mhm (bejahend)) Das andere macht die andere Hand (Interviewer: Ja.). (Sie?) aber, dann nüchtern schon (Teilnehmer 1: Ja was andere ist es letztendlich nicht.) sehr, sehr entlastet (..?) #00:15:15-8#

Interviewer: mhm (bejahend), ja. #00:15:16-1#

Teilnehmer 5: Ja bei mir war der ausschlaggebende Punkt, wenn ich mich richtig erinnere, ähm dass ich ähm, da haben wir gerade schon drüber gesprochen, drauf achten wollte dass ich meine Körperhaltung äh eingermaßen (Interviewer: mhm (bejahend)) verbessere. Und mit der Schmuckprothese (Interviewer: mhm (bejahend)) war es so, dass ich-. Oder ohne Prothese, mit Schmuckprothese, (Interviewer: mhm (bejahend)) war es oft so, dass ich beim Flaschen öffnen den Körper ein bisschen verdreht habe. (Interviewer: Ja.) Und dann auch meine Schulter einge-, ähm ja eingeschraubt habe. Und ähm da haben wir im Gespräch

versucht herauszufinden welche Funktionen ich brauche. (Interviewer: mhm (bejahend)) In welchem Maße. (Interviewer: mhm (bejahend)) Vor allen Dingen aber auch beruflichen Situationen. (Interviewer: Ja.) Ich ähm, es mir helfen würde einfach ne, ne Greiffunktion zu haben. Die sozusagen auf der selben Höhe ist wie bei der gesunden Hand. (Interviewer: mhm (bejahend)) Und äh dann wurde die, diese Prothese, diese Prothesenform vorgeschlagen. (Interviewer: Ja.) Genau. Und das ist ähm, sozusagen mein erster Versuch. Den ich seit einem (Interviewer: OK.) Jahr jetzt gerade teste. #00:16:01-7#

Interviewer: Ja. Und, aber würden Sie sagen, jetzt rückblickend, wenn Sie sagen davor war es ne Schmuckprothese, war's ne gute Entscheidung? #00:16:10-8#

Teilnehmer 5: Ähm, ja. Also ich fühl mich, (Interviewer: mhm (bejahend)) mit der Prothese wohl. Aber vor allen Dingen (Interviewer: mhm (bejahend)) im beruflichen Kontext fühle ich mich sehr wohl damit (Interviewer: mhm (bejahend)). (Interviewer: Ja.) Ähm ich bin Lehrer und hab dann Situation oft, wo ich äh Gegenstände halten kann. Und einfach ne freie Hand habe. (Interviewer: mhm (bejahend)) Und das hatte ich vorher nicht. (Interviewer: mhm (bejahend)) Auch ne natürliche Bewegung habe. Ne natürliche (Interviewer: mhm (bejahend)) Körperhaltung dabei. Und nicht n Zettel irgendwie an den Körper drücken muss (Interviewer: Ja.), weil ich gleichzeitig nem Kind ne Unterschrift geben muss. Oder n Buch festhalten muss. (Interviewer: Ja.) Ähm das war jetzt nur, nur ein Beispiel. Das gibt's ganz viele (Interviewer: mhm (bejahend)). Türe öffnen etc. Ähm da ist schon ne deutliche Erleichterung (Interviewer: Ok.), ja. Wobei ich im privaten Kontext sagen muss, wenn ich m-, mit meinen Kindern zu tun habe, dass ich da auch gerne ohne Prothese (Interviewer: mhm (bejahend)) weiterhin ähm agiere, nicht? Weil ich einfach n ganz anderen Zugang habe dann auch nochmal. Ja. #00:16:51-7#

Interviewer: Warum genau? Was macht den anderen Zugang aus? #00:16:57-3#

Teilnehmer 5: Naja, es ist schon n Zwischenstück was da ist, nicht? (Interviewer: mhm (bejahend)) Zwischen meinem Körper und dem meiner Kinder. Und (Interviewer: Ja.) ich würd jetzt meinen Sohn so nie hochheben. Und in die Luft schmeißen. (Interviewer: Ok.) Das mach ich aber ohne Prothese ohne Probleme. (Interviewer: Ja, ok. mhm (bejahend)) Ja? Und schmeiß ihn hoch, fang ihn wieder auf. Und das kann ich aber (..?) mit Prothese nicht. Weil ich mir das nicht zutraue. (Interviewer: Ja.) Und das ist einfach auch irgendwie n, dann doch noch n Hindernis, ja? #00:17:12-5#

Interviewer: Ja, ok. #00:17:14-3#

Teilnehmer 6?: Da würde man das Kind auch wahrscheinlich mit verletzen. #00:17:20-3#

Interviewer: Warum? #00:17:20-3#

Teilnehmer 6: Weil die Dinger doch sehr hart sind. (Interviewer: Ok, ja.) Und (..??). #00:17:24-5#

Teilnehmer 5?: (Lärm im Hintergrund) (..??) Mit mehr Gefühl in der, äh (Interviewer: mhm (bejahend)) im, im Unterarm (Interviewer: Ja, klar.). Als jetzt mit der Prothese.

(Interviewer: mhm (bejahend)) Ja. Ich mein, ich glaub, da spreche ich auch für alle anderen. Ich, ich spür sehr viel, nicht? (Interviewer: mhm (bejahend)) Auch mit dem Arm. Und wenn, wenn mir jemand die Prothese berührt, dann merk ich das sofort. (Interviewer: mhm (bejahend)) (Zustimmendes Raunen) Ich mach dann so'n Spielchen, dass ich meine Augen schließe und dann dürfen die Kinder das anfassen (Interviewer: (lacht)). Und ich merk das eigentlich immer. (Interviewer: Ja.) Und äh, aber das ist trotzdem noch was anderes, ob ich jetzt ‚ne Prothese trage oder nicht. (Interviewer: mhm (bejahend)) Ja? Von daher, das, das Natürlichste ist natürlich keine Prothese zu tragen (Interviewer: Ja.), ja? Aber äh es ist doch schön einfach ähm ja, auch im, im Umgang mit Menschen im Beruflichen. Aber auch in privaten Situationen wo's jetzt nicht so ähm körperbetont ist (Interviewer: mhm (bejahend)) wie zum Beispiel beim Spielen. Ähm ist es doch schön auch weitere Funktionen zu haben. Die diese (Interviewer: Ja.) Prothese bietet. #00:18:10-5#

Interviewer: Ja, klar. Gibt's da noch irgendwas hinzuzufügen? Möchte da noch jemand was dazufügen? #00:18:17-8#

(kurze Pause) #00:18:18-7#

Interviewer: Okay. Ähm dann würde ich gerne als nächstes wissen ähm was Sie sagen würden, was so die drei positivsten Sachen sind, die Sie an der Prothese finden. Also viel haben wir jetzt wahrscheinlich schon gesagt. Eben was die Erleichterung ist, dass die Prothese da ist. Aber einfach was Sie an der Prothese so am meisten schätzen. Wenn Sie so dei drei wichtigsten Punkte aussuchen müssten. #00:18:38-2#

Teilnehmer 2?: Kosmetik. #00:18:40-4#

Teilnehmerin 1: Watt? #00:18:41-0#

Teilnehmerin 3?: Die Selbstständigkeit. Also (Interviewer: mhm (bejahend))-. #00:18:44-5#

Teilnehmer 4: Und dann noch die (..??) (Lärm im Hintergrund). Für die Wirbelsäule ist das auch besser. (..??). (Interviewer: mhm (bejahend)) #00:18:54-0#

Teilnehmerin 1: Also grundsätzlich was ich daran schätze. Ich hab mir also angewöhnt von Anfang an eine Prothese überhaupt zu tragen. Das ist ja schon mal n Kampf. (Interviewer: Ja. Ja.) Äh es gibt zwei Möglichkeiten. Entweder man akzeptiert das und trägt sie. (Interviewer: mhm (bejahend)) Oder man akzeptiert es und trägt sie nicht. Äh die Problematik wie ich mich verbiegen muss, wenn ich keine Prothese trage. Das geht also beim Anziehen los. (Interviewer: mhm (bejahend)) Äh Hose schließen. Gürtel. Äh ich bin also ständig als Einhänder äh ohne Prothese eigentlich aufgeschmissen. (Interviewer: mhm (bejahend)) Weil ich kann nicht zwei Sachen gleichzeitig. (Interviewer: Ja.) Das heißt dieses (Kinderstimme im Hintergrund) äh Nutzinstrument. So nenn ich das mal ganz einfach. Muss man also letztendlich als Körperersatzteil genau so sehen wie's ist. (Interviewer: mhm (bejahend)) Es ist'n n Ersatzteil, was eigentlich die gesunde Hand entastet (Interviewer: mhm (bejahend)). Mehr ist es nicht. Und äh das ist auch der einzige Vorteil. Äh und, dass man letztendlich schneller vorankommt. Wie ohne Prothese.

(Interviewer: mhm (bejahend)) Das ist der Grund, warum ich so’n Ding überhaupt trage. Und man muss sich halt angewöhnen, das Ding immer ’n ganzen Tag von Anfang an zu tragen. Sonst wird das eh nichts. (Interviewer: Ja.) (Anderer Teilnehmer: Genau.) Also ich hab ganz viele Leute kennengelernt im Laufe meiner Prothesenzeit, die mit Prothese tragen, äh die dann in ‚ner Platiktüte haben. Und tragen sie ja auch. (Interviewer: mhm (bejahend)) (alle lachen) Bringt aber nicht so ganz viel. (Interviewer: mhm (bejahend)) Äh die akzeptieren die entweder gar nicht. Äh oder nur als schmückendes Beiwerk. Ich weiß es nicht. Also wie gesagt, mir wird nichts bringen, äh zusätzlich bin ich natürlich auch Oberarmpatient. (Interviewer: mhm (bejahend)) Das heißt also mir fehlt der Ellenbogen. Äh von daher hab ich gar keine Möglichkeiten (Interviewer: mhm (bejahend)) mit dem Stumpf irgendwas zu machen. (Interviewer: Ja.) (Fast?) nichts. Also Prothese. Ende. #00:20:24-7#

Interviewer: OK. #00:20:24-7#

Teilnehmer 5: Das ist bei mir noch’n bisschen anders. Da ich von, von Geburt an äh fehlt mir die rechte Hand. Das heißt ich hab so das, das rechte Handgelenk. (Interviewer: mhm (bejahend)) Noch, wenn man das so formulieren kann überhaupt. Ähm und das heißt ich kann auch ohne Prothese ne Hose anziehen und (..?) zumachen. (Interviewer: mhm (bejahend)) Aber ich merk halt dabei, dass ich dann immer ne Körperhaltung annehme, die, die nicht (Interviewer: Ja.) gesund ist. Ja? #00:20:42-6#

Teilnehmerin 1: Können kann ich’s auch. Aber wie gesagt, ob man das möchte. Das ist halt so immer das Problem. (Teilnehmer 5: Genau.) Weil- #00:20:47-5#

Teilnehmer 5: Das ist, ich merke dass es einfach auch angenehm ist, das mit ‚ner Prothese zu machen. (Interviewer: mhm (bejahend)) Genau diese Bewegung. (Interviewer: Ja.) Als ne andere, langfristig ne andere Körperhaltung (Interviewer: mhm (bejahend)) (..?), ja. (Interviewer: mhm (bejahend)) #00:20:55-5#

Interviewer: Okay, das heißt wir haben die Körperhaltung. Sie (wendet sich an Teilnehmer 4) haben gesagt Entlastung der anderen Seite. Was würden Sie sagen, Herr (Name von Teilnehmer 2 aus Datenschutzgründen ausgelassen), was Sie an Ihrer Prothese am meisten schätzen? #00:21:04-4#

Teilnehmer 2: Mh, ja auch die Optik sag ich mal. (Interviewer: mhm (bejahend)) Die Kosmetik. Aber auch die Funktion einfach. Also ich trag die Michelangelo Hand. (Interviewer: mhm (bejahend)) Und ähm das hat mich jetzt, ja, insgesamt auch weitergebracht. Dass man noch mehr, feiner greifen kann. (Interviewer: mhm (bejahend)) Und solche Geschichten. Bei mir ist es äh wie bei Herrn (Name von Teilnehmer 5 aus Datenschutzgründen ausgelassen) auch so. Ich bin auch ohne Hand geboren. (Interviewer: mhm (bejahend)) Kenne also sozusagen mein Leben lang Prothese. (Interviewer: Ja.) Ich hab also als fast Säugling schon ne Prothese getragen. Und bin mit Eigenkraftprothesen aufgewachsen. (Interviewer: mhm (bejahend)) Äh bis ich dann irgendwann im 18. Lebensjahr die erste Myoelektrische gehabt habe. (Interviewer: mhm (bejahend)) So war das für mich nie n Thema. Äh die Prothese anzuziehen oder nicht. Also ich habe das immer, die immer getragen. Das war für mich morgens wie ne Brille aufziehen. Hätte ich bald gesagt. (Interviewer: Ja.) Die Prothese anzuziehen. Und äh klar, auch zuhause, sag ich mal. Ziehe ich die Prothese auch schon mal aus. Ähm um einige Sachen so wie

Kinder oder sowas (Interviewer: mhm (bejahend)), ich hab auch ne Tochter, äh wenn man damit spielt. Vielleicht mehr das Gefühl dafür zu haben. Aber genauso trage ich die Prothese auch äh für alle möglichen Sachen. (Interviewer: Ja.) Also eigentlich morgens anziehen, abends wieder ausziehen. (Interviewer: mhm (bejahend)) Und äh für mich war's auch diese, diese mich für diese Prothese zu entscheiden, weil äh auch um diese Entlastung vom, vom Körper (..??). Ich sitze im Büro am Computer den ganzen Tag. Da ist es schon angenehmer, wenn man auch das Handgelenk (Interviewer: mhm (bejahend)) (..?) diese Handgelenkgeschichte, dass man das so'n bisschen entlastet. (Interviewer: Ja.) Dass man diese natürlichere Haltung hat. (Interviewer: Ja.) Auch Fahrrad fahren. Und äh diese Geschichten. (Interviewer: mhm (bejahend)) Das war für mich eigentlich äh wichtig, sag ich mal. #00:22:34-7#

Interviewer: Und bei der Optik? Was würden Sie sagen, was ist das Wichtigste was sie können oder haben muss? #00:22:39-6#

Teilnehmer 2: Also bei dieser ist es einfach so, wenn die äh wenn man unterwegs ist, fällt diese Hand relativ wenig auf. (Interviewer: mhm (bejahend)) Weil die halt diese natürlichere Form hat. (Interviewer: mhm (bejahend)) Also zum Beispiel, ich hab auch noch die SpeedHand. (Interviewer: Ja.) Ähm die natürlich immer diesen Griff hat. Das vom, von der (Interviewer: mhm (bejahend)), vom Weiten her, sag ich mal, schon eher auffällt. (Interviewer: Ja.) Obwohl grundsätzlich für mich das nie n Problem war. Ähm ich hab mich nie versteckt oder sowas. (Interviewer: mhm (bejahend)) Ähm und wenn mich Leute ansprechen oder mich angucken, dann frag ich sie entweder, ob ich ihnen das erklären kann. (Interviewer: mhm (bejahend)) Ähm am besten sind immer Kinder die fragen. Und die Eltern ihnen immer sagen: (lacht) „Äh (komm?), frag nicht!". (Interviewer: (lacht)) Äh dann zieh ich die auch schon mal auf. Und geb denen die Hand: „Hier, kannst ausprobieren." (lacht). (Interviewer: mhm (bejahend)) Ähm das finde ich eigentlich, ja, ganz wichtig so. (Interviewer: mhm (bejahend), ja.) #00:23:25-4#

Interviewer: Und trotzdem, aber das heißt Sie sagen, dass es eher die Handform ist? Weil Herr (Name von Teilnehmer 4 aus Datenschutzgründen ausgelassen) hat ja gesagt, vor allem die Farbe ist ihm wichtig. #00:23:30-5#

Teilnehmer 4: Naja die Form (..??). #00:23:31-6#

Interviewer: Ja. #00:23:32-9#

Teilnehmer 2: Ja die Farbe- #00:23:33-4#

Teilnehmerin 1: Aber Farben gibt's ja reichlich eigentlich. #00:23:36-4#

Teilnehmer 2: Wie gesagt, die ist jetzt, sieht jetzt schön aus. Meistens ist (Interviewer: mhm (bejahend)) sie ziemlich dreckig. (Interviewer: (lacht)) Das interessiert mich auch nicht. (Gemurmel) Ähm fällt vielleicht mehr auf. Da mach ich mir eher weniger n Kopf. (Interviewer: mhm (bejahend)) (..??) #00:23:49-2#

Interviewer: Und Sie haben noch gesagt, was ich wichtig sind ähm sind die Funktionen? Was würden Sie denn sagen, was ist die wichtigste Funktion? Oder was sind die wichtigsten Funktionen die die Hand kann? #00:23:59-4#

Teilnehmer 2: Einfach die Greiffunktion. (Interviewer: mhm (bejahend)) Dass man was festhalten kann. Also n Glas tragen kann oder sowas. #00:24:08-5#

Teilnehmerin 1: Also n Glas tragen, n Glas halten kann ich auch mit ner SpeedHand. Da brauch (Teilnehmer 2: Ja.) ich jetzt keine Michelangelo Hand. (lacht) #00:24:12-3#

Teilnehmer 2: Eben, das stimmt. (alle lachen) #00:24:15-6#

Teilnehmer?: Ja das ist ja dann wieder so die Optik und äh- #00:24:17-5#

Teilnehmerin 1: Aber mit der kann ich besser (lacht). #00:24:19-7#

Teilnehmer 2?: Mit dieser kann ich auch. Weil die Finger einzeln sind zum Beispiel (..??). Handschuh drüber ziehen, um dann auch andere Tätigkeiten zu machen. (Interviewer: Ja.) Was mit der anderen Hand eben nicht geht. Also ich bin auch in der freiwilligen Feuerwehr. Und äh da wird's auch noch n bisschen dreckig. Und da bin ich schon froh, wenn ich da was drüber ziehen kann. (Interviewer: Ja.) Und dann nicht die SpeedHand nehme. #00:24:38-3#

Interviewer: Ja, ok. Und Herr (Name von Teilnehmer 3 aus Datenschutzgründen ausgelassen), bei Ihnen? Sie haben gesagt, dass äh die Selbstständigkeit n großer Punkt ist. Was macht die Prothese denn aus, dass Sie selbstständiger sind? #00:24:47-3#

Teilnehmerin 3: Also bei das jetzt, denn ich hatte plötzlich n Unfall. (Interviewer: mhm (bejahend)) Feuerwerkskörper. Und äh das Essen allein schon. Ich bin Italiener und (..?) Fischesser auch. Und äh, ne. Das ging nicht ohne Hand (..?) (Interviewer: mhm (bejahend)). Da, da hab ich gehungert. Ehrlich. (alle lachen) (Wollte?) n Schnitzel essen oder n schönen (..?) Kotlett. (alle lachen) (Teilnehmer ?: Aber nur wenn man schneiden kann.) Ja, ja. #00:25:07-3#

Interviewer: mhm (bejahend) #00:25:08-3#

Teilnehmerin 3: Also das auch. #00:25:08-8#

Interviewer: Ja. Gibt's da- #00:25:10-3#

Teilnehmerin 3: Auto fahren. (Interviewer: mhm (bejahend)) Fahrrad fahren. #00:25:12-9#

Interviewer: Ja, also einfach dass man sich selbstständig bewegen kann? #00:25:15-2#

Teilnehmerin 3: Selbstständig bewegen kann. (Interviewer: mhm (bejahend)) Und einfach, einfach mal loslegt einfach. (..?), ja. #00:25:19-4#

Interviewer: Ja. Und Herr (Name von Teilnehmer 4 aus Datenschutzgründen ausgelassen) bei Ihnen? Fällt Ihnen noch irgendwas ein, was an der Prothese gut ist? #00:25:24-8#

Teilnehmer 4: Ja, also die Greiffunktion ist am besten (Interviewer: mhm (bejahend)). Ja, was noch gut ist, äh diese Silikonhandschuhe, die sind kosmetisch sehr schön. (Interviewer: mhm (bejahend)) Die werden nicht dreckig. (Interviewer: mhm (bejahend)) Aber die sind sehr (glitischig?). Wenn man was Kleines greift (Interviewer: mhm (bejahend)), dann rutscht das raus. Also bei (..?). (Interviewer: Ja.) PVC ist viel besser zum Greifen. (Interviewer: mhm (bejahend)) Ist aber kosmetisch (Interviewer: OK, ja.) (..??). Aber sonst ja. (..??) Bisschen schwieriger ist bei (..????) Ich hab ja auch (..??) Zum Drehen auch. #00:26:07-7#

Teilnehmer 6?: N Akkuschrauber immer dabei? #00:26:10-1#

Teilnehmerin 1: Ne Prothese tragen ist kein Ponyhof halt, nicht? #00:26:14-0#

Teilnehmer 6: Ja, aber man muss sie ja auch akzeptieren. Solange man die Prothese nicht akzeptiert hat (Interviewer: Ja.), (..??) nicht, nicht mit seinem Handicap auseinandergesetzt hat (Interviewer: Ja.), geht das schwer irgend-, irgendje-, irgendein System äh anzunehmen. Und zu bedienen. (Interviewer: Ja.) Da fehlt einfach (was?). (Interviewer: Ja.) (…??) So und wenn du dich mit deiner Prothese (..?) iden-, identifiezierst, (Interviewer: mhm (bejahend)) ja dann, dann kannst du da ne ganze Menge mehr noch machen. (Interviewer: mhm (bejahend) Als, als wie man sich vorstellen kann. (Teilnehmer 4 raunt zustimmend) #00:26:44-3#

Interviewer: Das heißt schon, um die Vorteile zu nutzen ist es erstmal ne Kopfsache? #00:26:48-3#

Teilnehmer 6?: Ja. (Interviewer: Ok.) Das ist wie beim Rauchen. Wenn ich rauche, ja? (Interviewer: mhm (bejahend)) Bin ich abhängig von meiner Zigarette. Wenn ich se loswerden will, muss ich se wegschmeißen. #00:26:56-0#

Interviewer: Ja. #00:26:57-2#

Teilnehmer 6: Das ist aber ne Kopfsache die wegzuschmeißen. (Interviewer: Ja, ja.) #00:26:58-3#

Teilnehmerin 1: Ich will die Prothese ja auch wegschmeißen. Hätte ich kein Problem mit. (alle lachen) #00:27:00-8#

Teilnehmer 6: Äh ne (lachend). #00:27:02-5#

Teilnehmer 2: Aber es sind halt Leute- #00:27:05-5#

Teilnehmer 6: Nochmal dazu. Meine Prothese ist mein einzigstes Hilfsmittel. (Interviewer: mhm (bejahend)) Was ich in meinem ganzen, ganzen Tagesablauf benommen, benutze. (Interviewer: mhm (bejahend)) Was heißt benutze? #00:27:13-1#

Teilnehmerin 1: Schon klar. Nur gäbe’s da ne andere Möglichkeite glaube ich, wäre hier kein Einziger-. Wenn es eine Reimplantationstechnik gäbe, die also dafür sorgen würde, dass man-. Ich will jetzt keine 100%. Aber ich sag mal, wenn ich ne

Chance hätte 80% wieder zu erlangen, würde ich's morgen machen. #00:27:26-9#

Teilnehmer 6: Natürlich. (Interviewer: mhm (bejahend)) #00:27:28-5#

Teilnehmerin 1: So. Problematik ist, dass es nicht geht. Ich hab also in, in Österreich jemanden getroffen damals. Der hat sich beide Hände reimplantieren lassen. Einer von den wenigen Patienten, die das überhaupt über zig Jahre durchgehalten haben. Äh und er ist dann jetzt letztendlich verstorben an den Medikamenten, die er (Teilnehmer?: Schlucken muss.) (Interviewer: mhm (bejahend)) über viele Jahre schlucken musste. Das ist dann der Haken an der, und ist also auch teuer gekauft. (Interviewer: mhm (bejahend)) #00:27:48-0#

Teilnehmer 5: Es passt zwar nicht mehr zu der Frage, die Sie gerade gestellt haben (Interviewer: mhm (bejahend)), aber ich hab mir diese Frage auch oft gestellt. Welche Möglichkeiten ich hätte. Nämlich aus eigenen Zellen mir ne Hand äh entwickeln lassen könnte. Und die anpflanzen lassen würde oder nicht. Also ich kann nicht so klar antworten. (Interviewer: mhm (bejahend)) #00:27:58-3#

Teilnehmerin 1: Also ich mach das morgen auf den Tag, wenn ich ne Chance hätte definitiv. Ja. #00:28:03-9#

Interviewer: mhm (bejahend). Herr (Name von Teilnehmer 2 aus Datenschutzgründen ausgelassen) Sie wollten gerade noch was sagen? #00:28:05-5#

Teilnehmer 2: Ähm ja, sie fragen immer nach den Funktionen und sowas. (Interviewer: Ja.) Also das fällt mir immer bisschen schwer das zu beantworten, weil viele Sachen, zumindest geht mir das so, macht man einfach automatisch. #00:28:16-0#

Interviewer: Ja, klar. #00:28:17-8#

Teilnehmer 2: Denkt man eigentlich gar nicht mehr drüber nach, wozu brauch ich die eigentlich jetzt? (Interviewer: mhm (bejahend)) Oder äh welchen Griff nehme ich jetzt, um jetzt irgendwas zu machen. (Interviewer: mhm (bejahend)) Das macht man einfach. Vom Kopf her. #00:28:26-4#

Teilnehmerin 1: Ja. #00:28:25-0#

Teilnehmer 2: Das ist einfach so. (Interviewer: Ja.) Deswegen ist immer bisschen schwierig das zu beantworten (lachend). Wofür brauche ich jetzt genau das. #00:28:31-2#

Teilnehmer 6: Deswegen sagte ich ja eben, man muss sich mit seiner äh Prothese identifizieren. (Teilnehmer 2: Ja, genau.) Das geht dann alles auf automatisch. (Interviewer: mhm (bejahend)) Wenn man das da oben begriffen hat (zeigt auf seinen Kopf), dann läuft das da auch in der Hand. (Teilnehmer 2: Ja, klar.) #00:28:39-3#

Interviewer: Wobei das ja wahrscheinlich auch ein Vorteil ist, dass man eben Sachen so automatisch machen kann. Oder? Dass man gar nicht mehr darüber nachdenken

muss? #00:28:47-8#

Teilnehmer 2: Ja, auf jeden Fall! Klar. Wenn ich jedes denken müsste ähm wie muss ich die jetzt ansteuern (Interviewer: Ja.) um den und den Griff zu machen, dann ist was falsch. Also- #00:28:55-7#

Interviewer: Ja, klar. #00:28:56-6#

Teilnehmer 2: Das, das muss schon automatisch gehen. (Interviewer: mhm (bejahend)) Ja, klar. #00:28:56-2#

Interviewer: Und wenn wir jetzt mal in die andere Richtung gucken. Und mal sammeln was so die drei negativsten Dinge sind. Die am wenigsten funktionieren. Oder die Ihnen am wenigsten gefallen daran? #00:29:07-4#

(irgendjemand antwortet im Hintergrund) (alle lachen) #00:29:10-4#

Teilnehmerin 1: Der Orthopädietechniker. (alle lachen) #00:29:11-7#

Teilnehmer 6: Der, der Herr (Name von Orthopädietechniker aus Datenschutzgründen ausgelassen) (alle lachen). Der braucht Zeit um aufzuschreiben und dann macht er nichts. #00:29:21-6#

Teilnehmerin 1: Also das Negativste letztendlich ist die Haltbarkeit. Also tatsächlich bei mir zumindestens. (Interviewer: mhm (bejahend)) Weil ich gehöre ja zu den äh wenigen Prothesenträgern in äh der Welt glaub ich, die ständig ihre Prothese kaputt haben. Ich weiß nicht woran’s liegt. An mir, hör ich immer wieder. (Interviewer: (lacht)) Äh ausschließlich an mir. Weil alle anderen haben dieses Problem nicht. (Interviewer: mhm (bejahend)) Ich bin der einzige, der sie ständig kaputt hat. Immer. #00:29:38-6#

Teilnehmer 6: Also hier in der Runde nicht. (alle lachen) (…??) #00:29:42-4#

Interviewer: Was geht denn kaputt? Wo sind denn dann die Schwachstellen? #00:29:44-9#

Teilnehmerin 1: Äh die Hand ist also, ich würd mal sagen so im, im vier bis fünf, sechs Wochenrhythmus spätestens kaputt. (Interviewer: mhm (bejahend)) Äh zwischenzeitlich weiß ich, dass bei Herrn (Name von Teilnehmer 3 das ähnlich ist. (Interviewer: mhm (bejahend)) Äh ist also irgendwo wahrscheinlich konstruktiv bedingt. Dass die Dinger nicht dazu geeignet sind, damit tatsächlich was zu machen. Sondern eher zum Angucken. Hab ich so das Gefühl. (Interviewer: mhm (bejahend)) #00:30:07-3#

(Durcheinandersprechen, unverständlich) #00:30:10-4#

Teilnehmerin 1: Ellenbogen ist das gleiche Problem. Einmal feste draufgelegt. Ich hab auch n elektrischen Ellenbogen. Das Problem ist, auch der ist also eigentlich mehr in Reparatur (Interviewer: mhm (bejahend)), wie ich ihn tatsächlich nutzen könnte. Deswegen nutze ich ihn also höchst selten. (Interviewer: mhm (bejahend)) Äh damit ich also möglichst lange was davon habe. Ist glaub ich die falsche

Entscheidung. Normalerweise müsste man die Zeit haben, täglich zum Orthopädietechniker zu fahren. (Interviewer: mhm (bejahend)) Und den (Krimm?) zur Reparatur zu geben. (Interviewer: mhm (bejahend)) #00:30:33-4#

Interviewer: Und aber wissen Sie da selber Bescheid, was da genau kaputtgeht? #00:30:34-3#

Teilnehmer 1: Also viele Sachen erfährt man ja als Träger nicht, was denn daran kaputtgeht. (Interviewer: mhm (bejahend)) Ich hab mich nur gewundert. Also ich hab äh DynamicArm heißt das Ding, richtig? Äh der also für mich offensichtlich äh heile in eine Reparatur geht. Dann äh (Teilnehmer 6: Kaputt wieder zurückkommt.) mit zahlreichen Defekten belegt ist. Wo ich mich dann immer wunder, wie die da drangekommen sind. (Interviewer: mhm (bejahend)) Äh und immer ist es der Patienten Schuld. (Interviewer: mhm (bejahend)) Äh (..?) schon erschreckend. Ist einfach so. (Interviewer: mhm (bejahend)) #00:31:03-6#

Teilnehmer 2: Äh ich hatte zuletzt mit dieser Prothese technische Aussetzer. (Interviewer: mhm (bejahend)) Äh das heißt äh dass ich beim, dass es sich automatisch ausgeschaltet hat. Obwohl ich die Funktion nicht bedient habe. (Interviewer: Ja.) Das passierte beim Fahrrad fahren, was noch äh ungefährlich ist. Aber auch äh einige Mal beim Autofahren. (Interviewer: mhm (bejahend)) Das heißt ich hab dann ähm das Lenkrad festgehalten. Und äh plötzlich hat die Prothese sich ausgeschaltet. Und ich konnte sie nicht vom Lenkrad entfernen. (Interviewer: mhm (bejahend)) Was grundsätzlich auch kein Problem ist. Äh aber einmal war ich tatsächlich im Kreisel. Und wenn die Prothese genau in diesem ausschaltet (Interviewer: mhm (bejahend)) (alle lachen) (durcheinander sprechen) und ich aus dem Kreisel raus muss. Und schnell irgendwie meinen Körper drehen muss. (Interviewer: Ja.) Äh dann ist das schon gefährlich, ja? (Interviewer: Ja, klar.) Und die ist jetzt auch eingeschickt worden. Und, und ähm ja, soll, es soll herausgefunden werden woran das liegt. (Interviewer: mhm (bejahend)) Das Zweite ist eben, was grade schon gesagt wurde, (Interviewer: mhm (bejahend)) die Haltbarkeit von den (Interviewer: mhm (bejahend)) (..?) oder die Haltbarkeit von Kosmetikhandschuhen. Ja ich greife auch viel. Ich halt dann auch fest. (Interviewer: Ja.) Und halt mich auch an Dingen fest. Äh und da ist der Kosmetikhandschuh gerade an dieser Stelle, wo der Druck sehr hoch ist (Interviewer: mhm (bejahend)) eben stark belastet. (Interviewer: mhm (bejahend)) Und ne dritte Sache, die ähm ja die mit der Prothese auch zu tun hat äh ist, dass ich allergisch reagiert habe auf die Kontaktstellen. (Interviewer: mhm (bejahend)) Das heißt dass die Haut an der Stelle, wo die beiden Kontakte ähm zu finden sind hie innen drin ähm dann allergisch reagiert hat. (Interviewer: mhm (bejahend)) Und dann tragen auch für ne kurze Zeit nicht mehr möglich war. (Interviewer: Ja.) Das sind so die drei Schwachstellen, die jetzt an dieser Prothese erstmal ausmachen würden. #00:32:17-5#

Interviewer: Ja. Wie haben Sie dem Abhilfe v-, ähm verschaffen mit dieser allergischen Reaktion? #00:32:20-7#

Teilnehmer 2: Ähm mit ner äh Behandlung. (Interviewer: mhm (bejahend)) Durch, durch Cremes. (Interviewer: Ja.) Äh und einfach n ähm kompletten Verzicht auf die myoelektrische Prothese für ungefährt drei Monate. #00:32:30-9#

Interviewer: Okay, ja. #00:32:32-0#

Teilnehmer 2: Ja. (Teilnehmer ?: Boah, das könnt ich nicht.) Anders äh (Interviewer: mhm (bejahend)) ging da gar nicht. Nicht? (Interviewer: Aber jetzt-) Also sobald ich die Prothese wieder angezogen habe, fing das sofort wieder an. (Interviewer: Ja.) Also flammte das wieder auf. (Interviewer: mhm (bejahend)) Nee jetzt ist's äh weg. Als wenn's nie dagewesen wäre. (Interviewer: Okay.) Weiß nicht, ob sie nicht noch verstärkt wurde durch, durch die Jahreszeit. Oder durch ähm, durch, durch andere Faktoren. (Interviewer: mhm (bejahend)) Das kann ich nicht beurteilen. Aber jetzt ist es Gott sei Dank wieder weg. (Interviewer: Ja, okay. mhm (bejahend)) #00:32:54-5#

Interviewer: Und bei den anderen? Gibt's noch irgendwelche negativen Punkte, die wichtig wären zu erwähnen? #00:32:59-7#

Teilnehmer 6: (..??) Also ich bin jetzt Schulter Ex. (Interviewer: mhm (bejahend)) Und hier, ich hab mein (..?) einmal für'n Zug und einmal für's oben Anbinden. (Interviewer: mhm (bejahend)) Und da muss man regelmäßig drauf achten, dass man äh nicht wund wird. Weil äh durch die ganzen Bewegungen im Körper (Interviewer: mhm (bejahend)) äh reibt das hier oben. Äh man schwitzt auch viel. (Interviewer: Ja.) Äh ich bin einer, der sehr viel dabei schwitzt. Äh morgens beim Duschen geht man einfach hin und muss richtig da Stellen mit Wasser (Interviewer: mhm (bejahend)) äh nachlaufen lassen. (Interviewer: Ja.) Und das find ich nicht in Ordnung dann, ne? #00:33:29-4#

Interviewer: Ja. #00:33:28-9#

Teilnehmer 6: Ich trage meine Prothese ja auch wirklich 14, 15 und 16 Stunden am Tag. (Interviewer: mhm (bejahend)) (..?) (Interviewer: Ja.) #00:33:40-4#

Interviewer: Gibt's noch irgendwas hinzuzufügen? #00:33:39-9#

Teilnehmer 2: Also bei mir ist es auch so mit der Elektronik, dass die zwischendurch mal spinnt. Beziehungsweise auch äh die Hand als solches sehr empfindlich ist. Also grade so der Daumenbereich. Also einmal im Jahr der Daumen ist äh meistens kaputt. (Interviewer: mhm (bejahend)) (lacht). Immer dann, wenn er zum Service muss, ist der Daumen komischerweise auch kaputt. #00:33:58-4#

Interviewer: Okay. Und was heißt Aussetzer? #00:34:01-3#

Teilnehmer 2: Ja, dass man ähm, dass die dann äh auf und zu geht. Oder ähm zudrückt äh wenn man's gar nicht will. (Interviewer: mhm (bejahend)) Wo man nichts dran machen kann. Dass man entweder einmal komplett wieder ausmachen muss (Teilnehmer 1?: mhm (bejahend)). Äh und hofft, dass sie dann wieder richtig funktioniert. Aber manchmal, wie gesagt, beim letzten Mal hatte ich's jetzt auch grade, ähm dann hat die immer zugedrückt von alleine. Und äh (Interviewer: mhm (bejahend)) ich konnt se nicht mehr richig steuern. (Interviewer: Ja.) Hatte letztendlich wahrscheinlich was mit diesem Daumen zu tun, der dann defekt war. (Interviewer: mhm (bejahend)) #00:34:29-9#

Teilnehmerin 1: Quatsch. (Name eines Prothesenhersteller aus Datenschutzgründen ausgelassen) Fernbedienung (..?). #00:34:28-6#

Teilnehmer 2: (alle lachen) Ja, oder so. Das Jahr war wieder um, ne? #00:34:33-8#

Teilnehmer ?: Die haben nichts zu tun. (alle lachen) Mach Service. #00:34:36-3#

Teilnehmer 4: (..??) Elektrosmog und dann geht die Hand auf. (Interviewer: mhm (bejahend)) Oder ich arbeite bei der Bahn. Oberleitung. 15 000 Volt und dann die ist auch schon immer ganz schlecht (..?) (Interviewer: mhm (bejahend)) Dann ist eine Störung oder so. (Teilnehmer 6: Ja.) #00:34:53-5#

Teilnehmer 6: Grade, grade wenn man, wenn man n Handy, wenn man dann n Handy dann (..?) und die Antennen dann alle da s-, da schön in die Hand reinstrahlen. (Teilnehmer 4: Ja.) Dann den Stecker reinmachen will. Und hat in dem Moment, wo man den Stecker einführen möchte (alle lachen), (..?). #00:35:09-1#

Teilnehmer 4: Ja (lacht). #00:35:11-0#

Interviewer: Das heißt das ist einfach Wechselwirkungen mit anderen elektronischen Geräten? #00:35:13-5#

Teilnehmer 6: Ja. #00:35:14-6#

Teilnehmer 4: Ja und (..??) #00:35:14-4#

Interviewer: mhm (bejahend). Ja. #00:35:23-5#

Teilnehmer 4: (…???) Wen man bisschen schließt, (Interviewer: mhm (bejahend)) dann ist auch Störung. (Interviewer: mhm (bejahend)) Ich hab auch manchmal Probleme, na ja, dann muss man sich gewöhnen (Interviewer: Ja.) (..??). Mehrmals was ausprobieren, was passt. Wir benutzen Seuerung (..???). #00:35:43-8#

Interviewer: Und jetzt haben Sie gesagt, dass eben wenn sich die Haut verändert, man schwitzt, dass das nicht so n gutes Signal gibt. #00:35:50-3#

Teilnehmer 4: Nee (…?) #00:35:48-9#

Teilnehmer ?: (..?) #00:35:51-1#

Interviewer: Kennen das andere auch? #00:35:52-3#

Teilnehmerin 2/1: Ja. #00:35:53-0#

Teilnehmer 6: Gibt’s, da gibt’s n Übersignal, einfach ne Störung von, von dem einen Kontakt (Interviewer: mhm (bejahend)) zu dem anderen Kontakt über diese, über’s Schwitzwasser. (Interviewer: Ja.) Dass man so- #00:36:01-8#

Teilnehmerin 1: Also Feuchtigkeit generell ist natürlich der Tod einer jeden Elektrode. Das heißt also ob das jetzt durch Schwitzen, die Luftfeuchtigkeit oder sonst irgendwas, (Teilnehmer 4: mhm (bejahend)) äh-. (Interviewer: mhm (bejahend)) Sobald das, die Kontaktstellen feucht werden, am besten noch Armumgreifend, sorgt das dafür, dass man zahlreiche Fehlbedienungen hat.

(Interviewer: mhm (bejahend)) Das ist halt definitv (..?). #00:36:18-4#

Teilnehmer 6: (..??) (Lärm im Hintergrund) Akklimatisieren, eigentlich wenn's dann wieder n bisschen abgetrocknet ist, geht's wieder. #00:36:27-4#

Interviewer: Okay, ja. Gibt's da noch andere Zusammenhänge zwischen eben der Haut, wie die gerade ist von der Temperatur oder weiß nicht- #00:36:36-1#

Teilnehmer 1: Es gibt Zusammenhänge mit'm Stumpf. (Interviewer: mhm (bejahend)) Also wie gesagt, ich äh gehöre zu dieser Problemgruppe. Glaube ich. Das heißt, es gibt Tage, wo ich also nicht in die Prothese reinkomme generell. (Interviewer: mhm (bejahend)) Ich hab also schon zwei unterschiedliche Schäfte. Äh musst ich lange für kämpfen, um die zu bekommen. Es gibt da so Tage, da ist der Stumpf anders als an anderen Tagen. (Interviewer: mhm (bejahend)) Was es ist weiß ich nicht. Auf jeden Fall ist er dicker. Äh wo's herkommt ist unbegreiflich. Wir haben versucht das über wochenlanges Messen (Interviewer: mhm (bejahend)) äh zu ermitteln. Das ist mir nicht gelungen. Es kann keiner sagen warum das so ist. Ich weiß aber, äh es gibt Tage, da komme ich defintiv nicht in meinen normalen Schaft rein. (Interviewer: mhm (bejahend)) Da muss ich einen etwas weiteren nehmen. (Interviewer: Ja.) Weiter bedeutet also da n Millimeter. (Interviewer: mhm (bejahend)) Äh ist ausreichend um da reinzukommen oder nicht reinzukommen. #00:37:20-9#

Interviewer: Ja. Und beeinflusst das dann auch die Funktion? #00:37:24-4#

Teilnehmerin 1: Äh ja. Wenn ich nicht reinkomme ist klar, hab ich gar keine Funktion. #00:37:26-5#

Interviewer: Klar, ja. Ja, das ist nachvollziehbar. (lacht) #00:37:25-9#

Teilnehmerin 1: Äh ansonsten ist es für mich da n Unterschied. Der etwas weitere Schaft passt an verschiedenen Tagen. Zumindestens mal dann für zwei, drei Stunden. Danach kann ich wieder wechseln. (Interviewer: mhm (bejahend)) Dann komm ich auch wieder in meinen normalen Schaft rein. Als wenn Wassereinlagerungen da wären oder sonst irgendwas. (Kinderstimme im Hintergrund) Aber messbar ist das Ganze nicht. Das (..?) daran. Äh es weiß keiner wo's herkommt. Ich hab das über Jahre hin versucht zu ergründen. Mir ist es nicht gelungen. (Interviewer: mhm (bejahend)) Das gleiche Phänomen wie Phantomschmerz. (Interviewer: Ja.) Er ist mal da, mal ist er weg. (Interviewer: Ja. Und man kann's halt nicht richtig messen.) Äh und man kann's nicht steuern. Man weiß auch nie wann er kommt oder wann er geht. Meistens wenn ich keine Prothese anhab. #00:38:02-0#

Teilnehmerin 3: (..??) #00:38:07-0#

Teilnehmerin 1: Ja. #00:38:08-2#

Interviewer: mhm (bejahend). Das heißt aber, dass die Prothese gegen Phantomschmerzen? #00:38:13-8#

Teilnehmerin 1: Also ich glaube es liegt am Kopf. Das heißt solange man tagsüber

mit der Prothese beschäftigt ist. Und zumindest das Gefühl erzeugt für den Kopf, man könnte damit äh normale Funktionen ausführen, hat man keinen Phantomschmerz. Äh in dem Moment, wo man zu Ruhephasen neigt, vielleicht mal n Abend vor'm Fernseher das Ding auszieht. (Interviewer: mhm (bejahend)) Weil's irgendwie ja doch nicht so angenehm ist, dann hat man, wenn man Pech hat, äh beginnen irgendwelche Phantomschmerzen. (Interviewer: mhm (bejahend)) Weil der Kopf nichts zu tun hat. (Interviewer: mhm (bejahend)) Das ist meine persönliche äh Feststellung. Die ich gemacht habe. Ziehe ich danach die Prothese wieder an, ist weg. (Interviewer: Okay, ja.) #00:38:48-3#

Teilnehmerin 3: (…??) #00:38:53-2# #00:38:53-1#

Teilnehmerin 1: Reset? (lacht) #00:38:55-8#

Teilnehmerin 3: Ja ich, also ich hab nicht viel zu tun mit den Phantomschmerzen. (Generell nicht?) (Kinderstimme im Hintergrund) (..???) (Interviewer: mhm (bejahend)) #00:39:04-0#

Teilnehmer 4?: Bei mir sind die immer da. Deswegen weglegen kann ich nicht. Aber ich hab's akzeptiert. Das tut bisschen weh. Und (..?) (Interviewer: mhm (bejahend)) (…??) #00:39:13-6#

Interviewer: Ja. Und jetzt aber noch zu diesen negativen Eigenschaften. Gibt's noch irgendwas, was jemand gerne noch hinzufügen würde? Was noch wichtig wäre? (Kinderstimme im Hintergrund) #00:39:21-8#

Teilnehmerin 3: Ähm ganz am Anfang hatte ich äh ne diese (..?). (Interviewer: mhm (bejahend)) Mit der Akkulaufleistung. (Interviewer: Ja.) (..??) Ich hatte äh den Akkus hatte ich, in fünf Stunden hatte ich die leergefahren. (Interviewer: Ok.) Also durch die Auf- und Zubewegung auch im Stapler (Interviewer: Ja.). (..?) Kisten und fahren, man kann sich das nicht vorstellen, dass sie n (..?) Akkusystem 2000 Volt Miliamper, (Interviewer: mhm (bejahend)) dass du die in fünf Stunden platt fährst. Aber das war so. (Interviewer: mhm (bejahend)) Das haben wir auch dann messen lasen und alles. (Interviewer: Ja.) Und äh ich brauchte n neues leistungstärkere A-, Akkus. (Interviewer: Ja.) Aber mittlerweile ist der Markt jetzt schon so, dass das alles- #00:39:58-4#

Interviewer: Dass es das- #00:39:59-0#

Teilnehmerin 1: Na, Rucksack. (alle lachen) (durcheinander sprechen) #00:40:00-3#

Interviewer: Das heißt jetzt haben Sie- #00:40:02-9#

Teilnehmerin 3: (..?) umgerüstet, dann passt das jetzt. #00:40:05-6#

Interviewer: Ja. Also einfach mit nem anderen Akkus der halt länger hält? #00:40:08-1#

Teilnehmerin 3: Genau. #00:40:07-0#

Interviewer: Wie ist das bei den anderen mit der Akkulaufzeit? #00:40:11-3#

Teilnehmer 4: (..??) #00:40:21-4#

Interviewer: Das heißt, Sie achten schon darauf, dass Sie den sparen, damit der nicht alle geht? #00:40:28-4#

Teilnehmer 4: Ja, zum (Beispiel im Garten?) da muss ich auch aufpassen (Interviewer: mhm (bejahend)). Dass die nicht dreckig wird. Nicht zu doll drücken. Das kann kaputtgehen. (Interviewer: mhm (bejahend)) Ein bisschen schonen (vielleicht?) (..?) Aber schonen muss man (..??) (Interviewer: Ja.)- #00:40:41-0#

Teilnehmerin 1: Also ne Vitrinenprothese. #00:40:43-8#

Teilnehmer 4: (Ja?) (lacht) #00:40:42-8#

Teilnehmerin 1: Ja, wenn du se in ne Vitrine legst, geht se nicht kaputt. So eine hab ich auch. #00:40:47-5#

Teilnehmerin 6: Also Akkuleistung, ich habe Wechselakkus. (Interviewer: mhm (bejahend))Ich kann (..??) Ich wechsel jeden morgen den Akku. (Interviewer: mhm (bejahend)) Und abends äh ist (immer noch voll?) (..??). (Interviewer: Ja.) Äh die Prothese ist äh angeschaltet äh den ganzen Tag. (Interviewer: mhm (bejahend)) (Kinderstimme im Hintergrund) Betriebsbereit. (Interviewer: mhm (bejahend)) Nutzbereit. Ich hab da kein Problem. #00:41:14-9#

Interviewer: Und das heißt aber, der hält auch von morgens bis abends? #00:41:15-8#

Teilnehmer 6: (nickt) #00:41:17-1# #00:41:17-2#

Interviewer: Ja, okay.

Teilnehmerin 1: Die Problematik ist natürlich auch letztendlich was, was man mit seiner Prothese macht. Ich gehöre jetzt zu den Patienten, das nervt ja immer den Orthopädietechniker, dass ich ständig überprüfe, ob das Ding auch in Bereitschaft ist. (Interviewer: mhm (bejahend)) Weil ich äh zu viele Reparaturen hatte. Das ist halt einfach so. (Interviewer: Ja.) Weil immer dann wenn du das Ding brauchst, kaputt. (Interviewer: mhm (bejahend)) So also pobiere ich ständig, den ganzen Tag, ob die Hand grade läuft. Ob der Ellenbogen schaltet. (Interviewer: mhm (bejahend)) Äh das frisst natürlich Akku. (Interviewer: Ja, klar.) Das ist vollkommen klar. Also wie gesagt auch da, aber das kostet. (Teilnehmer? : (..?) Und so’n 800 mA Akku äh da käme ich glaube ich n halben Tag mit dem. Noch nicht mal. (Interviewer: mhm (bejahend)) (Lärm im Hintergrund) #00:41:56-8#

Teilnehmer ?: (..??) #00:41:57-2#

Teilnehmerin 1: Aber ist auch nur ne Speed oder nicht? (Interviewer: Ja.) #00:41:56-3#

Teilnehmer 2?: Ja, das ist ne SpeedHand. #00:41:58-8#

Teilnehmerin 1: Deswegen also wie gesagt- #00:42:00-1#

Teilnehmer 6?: (..??) #00:42:03-6#

Teilnehmerin 1: Ja, aber ist ähnlich. (Teilnehmer?: Ja.) Also ich sag mal vom Verbrauch. Vom Stromverbrauch her glaube ich, sind die ähnlich. (??) Ja hallo. #00:42:12-0#

Teilnehmerin 6?: (..?) (alle lachen) #00:42:09-2#

Teilnehmerin 1: (lacht) Hatt ich auch schon. Deswegen weiß ich's. #00:42:15-2#

Teilnehmerin 6?: Ja, für mich reicht (..?). Soll, ich wo-, ich wollte eigentlich dieses (..?) nicht haben. #00:42:17-7#

Teilnehmerin 1: Ja, aber wie gesagt, ich hab jetzt hier drin ich glaube 2,2 auch. Sowas um den Dreh. Und äh damit komm ich über'n Tag. (Teilnehmer? : (..?) #00:42:27-8#

Interviewer: Das heißt aber, dass ist eben auch ne sehr individuelle Sache wie der- #00:42:27-6#

Teilnehmerin 1: Ja. #00:42:30-1#

Interviewer: Ja. #00:42:30-1#

Teilnehmerin 1: Also je mehr man daran rumbastelt. Je mehr man damit macht. Tatsächlich je mehr Bewegungen man macht umso mehr Probleme habe ich auch. (Interviewer: Ja.) Das ist normal. (Interviewer: Ja.) (Teilnehmer? : Ja.) #00:42:35-8#

Interviewer: Dann würde ich gerne zur nächsten Frage übergehen, wenn's da erstmal nichts mehr gibt. Und zwar würde ich gern von Ihnen wissen, ob's Situationen gibt, in denen die Sie, in denen Sie die Prothese gar nicht nutzen. Sie haben schon gesagt (an Teilnehmer 5 gewandt) eben wenn Sie mit den Kindern spielen, dass sie dann wegkommt. Wie ist das denn bei den anderen? Oder gibt's noch irgendwelche Momente, wo man sagt da ähm kommt die ab. Da ist eben nicht dabei? #00:42:55-3#

Teilnehmerin 1: Im Wasser. #00:42:55-9#

Interviewer: mhm (bejahend) #00:42:55-1#

Teilnehmer ?: Ja. #00:42:58-6#

Teilnehmerin 3?: Genau, schlafen. #00:42:59-8#

Teilnehmerin 1: Also alles was äh mit Wasser zu tun hat äh lässt man die Prothese besser ganz weit von weg. (Interviewer: mhm (bejahend)) (Teilnehmer ?: Schwimmen passt nicht.) Äh nee, schwimmen einmal. Nicht weit, aber tief. (alle lachen) Äh das bedaure ich eigentlich relativ äh sehr, weil wie gesagt ich war äh

aktiv Surfer (Interviewer: mhm (bejahend)) Äh alles was mit Wasser zu tun hat oder sonst irgendwas, kann man heute natürlich knicken. Da geht gar nichts mehr. (Interviewer: mhm (bejahend)) #00:43:23-3#

Interviewer: Das heißt es gibt auch kein anderes Hilfsmittel, auf das Sie zurückgreifen können? #00:43:25-7#

Teilnehmerin 1: Gibt nichts gescheites. Ich hab also so viel probiert. Gemacht und getan. Es funktioniert alles nicht, weil die Bewegungsmöglichkeiten fehlen. (Interviewer: Ja.) Und äh selbst mit ‚nem Greifer oder sonst irgendwas, der sorgt dann dafür, dass man nicht mehr gescheit schwimmen kann. (Interviewer: Ja.) Das ist das nächste Problem. Und man fällt ja auch schon mal rein. (Interviewer: mhm (bejahend)) Das ist als nicht machbar. (Interviewer: mhm (bejahend)) Und elektrische Bauteile, geht sowieso mal gar nichts. (Interviewer: Ja.) Also- #00:43:47-6#

Interviewer: Okay. Wie ist das bei den anderen? #00:43:50-7#

Teilnehmer 5: Ich arbeite als Lehrer auch viel am Schreibtisch (Interviewer: mhm (bejahend), ja.) Und äh tippe auch viel, wenn ich Arbeitsblätter erstelle oder Texte schreibe. (Interviewer: mhm (bejahend)) Und da hilft mir die Prothese ähm gar nicht. (Interviewer: mhm (bejahend)) Also da, ich hab n System entwickelt, wie ich mit äh fünf Fingern und eben dem Stumpft tippen kann. (Interviewer: mhm (bejahend), ja.) Und auch recht schnell mittlerweile. Ja was heißt mittlerweile. Mh schon (..?) lange. (Interviewer: Ja, ja.) Aber- #00:44:10-1#

Teilnehmerin 1: So gelernt. Letztendlich, weil (Teilnehmer 5: Ja, ja.) der Unterschied ist ja, dass m-, muss man wirklich so sehen, also jemand der mit äh seiner entsprechenden Behinderung aufwächst (Teilnehmer 5: mhm (bejahend)) hat mehr Möglichkeiten, wie jemand der, ich sag mal 30 Jahre oder 40 Jahre n Arm gehabt hat (Interviewer: mhm (bejahend)). Und kennt die Nutzung. Und dann ist der am nächsten Tag weg. (Mehrere Teilnehmer: Ja) Das ist immer das große Problem. Das äh sieht (..?) wieder, weil (Kinderstimme im Hintergrund) äh es ist dann selbstverständlicher (Interviewer: mhm (bejahend)) damit aufzuwachsen. Sag ich mal. Als wenn's von jetzt auf gleich weg ist das Ding. #00:44:35-5#

Teilnehmer 5: Richtig. Aber das stört mich (Interviewer: Ja.), äh das stört mich. Äh ich hab zwar n, versuch dann zwar grade zu sitzen, aber ich merke sobald ich eine halbe Stunde (Interviewer: mhm (bejahend)) oder Stunde am Schreibtisch gesessen habe, (Interviewer: mhm (bejahend)) dass ich dann ähm immer noch Rückenschmerzen bekomme. Weil ich natürlich ähm krumm sitze, ne? (Interviewer: Ja.) Aber ich hab keine Alternative dazu. Ich muss die Texte schreiben. Ich will sie auch schreiben. (Interviewer: mhm (bejahend)) Ähm will auch am Computer weiterhin arbeiten (Interviewer: mhm (bejahend))- #00:44:55-0#

Teilnehmerin 1: Sprachsteuerung. #00:44:56-5#

Teilnehmer 5: Ja, ich hab schon einige Systeme ausprobiert (Interviewer: Ja.), aber äh bin noch nicht zufrieden damit. Und einige kosten auch extrem viel. Und äh das Geld dafür will ich auch nicht ausgeben. (Interviewer: mhm (bejahend)) (Teilnehmerin 1: mhm (bejahend)) #00:45:03-9#

Interviewer: Aber um mal von der anderen Seite zu gucken, was müsste sie denn können die Prothese, damit sie dabei ne Hilfe ist? Gibt's, also, gibt's da ne Vorstellung die Sie haben, wie- #00:45:13-2#

Teilnehmer 5: Überhaupt nicht. #00:45:13-1#

Interviewer: Nee? Okay. #00:45:15-0#

Teilnehmer 5: Dafür ist das System jetzt so eingefahren. Ähm (Interviewer: mhm (bejahend)) ich mach das ähm also zu Beginn des Studiums hab ich damit angefangen. (Interviewer: mhm (bejahend)) Hab aber kurz vorher schon, schon am Schreibtisch getippt. (Interviewer: Ja.) Äh bin so schnell geworden, dass ich während des Studiums bei, bei Referaten gefragt wurde, ob ich nicht tippen kann. Weil ich's schneller kann. (Interviewer: Okay, ja.) Ähm von daher, ähm (Interviewer: mhm (bejahend)). Also es waren jetzt keine Schnelltipper, ja. Das, das muss man dazu sagen (alle lachen) die in der Gruppe waren. Ähm von daher glaub ich nicht, dass ne Prothese das (irgendwie?) ersetzen kann. (Interviewer: mhm (bejahend), okay) Es wäre schön, (..??) (Interviewer: Ja.) auch ne gesunde Körperhaltung einnehmen könnte. (Interviewer: Ja.) Aber ich glaube nicht, dass das an Effizienz das überbieten könnte. (Interviewer: mhm (bejahend), okay) #00:45:46-6#

Interviewer: Einfach weil sie nicht schnell genug wäre? Weil sie das halt nicht aufgreifen (Teilnehmer 5: Ja, genau.) könnte? Okay. mhm (bejahend) #00:45:51-4#

Teilnehmer 6: Also ich habe auch'n Computerarbeitsplatz. (Interviewer: Ja.) Ja? Aber meine Linke tippt immer mit. (Interviewer: mhm (bejahend)) (..?) (Du hast das schon gesehen?), ne? (Interviewer: Ja.) (Das funktioniert unglaublich?). #00:46:07-6#

Teilnehmerin 1: Ja, also ich glaube da gewöhnt sich auch wirklich jeder so seine eigenen Besonderheiten an (Mehrere Teilnehmer: Ja, mhm (bejahend)) letztendlich (Interviewer: mhm (bejahend)). Weil man macht das ja so, wie es für mich persönlich funktioniert. #00:46:18-8#

Interviewer: Ja, klar. #00:46:18-8#

Teilnehmerin 1: So. Und das heißt, man muss also ja letztendlich äh improvisieren. Und man muss Lösungen finden. Die für einen individuell passen. (Interviewer: mhm (bejahend)) Ob dann irgendjemand anders mit ner ähnlichen Lösung zurechtkommt (Interviewer: mhm (bejahend)), das muss man immer sehen. Also ich halte das für sehr unwahrscheinlich. (Interviewer: Ja.) Äh weil man da wirklich n Weg für sich suchen muss. (Teilnehmer ?: Ja.) Äh und nicht- Gibt ja so tolle Beschreibungen, was man alles toll machen kann als Amputierter. Finde ich klasse, funktioniert nichts von. Äh ist so. Und äh von daher muss man also wiriklich sich damit selbst beschäftigen. (Interviewer: mhm (bejahend)) Und äh einen Weg suchen, wie's denn nicht funktioniet. (Interviewer: Ja.) So. Dieser Weg muss für keinen anderen funktionieren. Deswegen brauch ich kein Buch schreiben „Ich und die Prothese". (Interviewer: Ja, klar.) Weil: Klappt halt nicht. (Teilnehmer 6: Richtig.) #00:46:58-2#

Interviewer: mhm (bejahend) und aber Herr (Name von Teilnehmer 3 aus Datenschutzgründen ausgelassen), Sie haben vorher noch erzählt, dass Sie eben eine für die Freizeit haben und eine für die Arbeit. Gibt's auch Momente wo Sie gar keine tragen? Oder- #00:47:07-0#

Teilnehmerin 3: Ja auch. Also ich hab auch zwei Kinder. Wenn wir draußen im Garten sind (Interviewer: mhm (bejahend)) spielen Fußball, in Pool springen also zieh ich (natürlich?) aus. (Interviewer: mhm (bejahend)) Zum Schlafen natürlich. (Interviewer: mhm (bejahend)) Also ich bin auch mal froh, wenn ich sie nach acht oder zehn Stunden am Tag auch mal ausziehen kann. Das ist ja- #00:47:23-5#

Interviewer: Und warum? #00:47:24-2#

Teilnehmerin 3: Das ist ja auch, ich glaub so ne Prothese wiegt 800 Gramm? Ungefähr? Das ist schon- #00:47:29-7#

Teilnehmerin 1: Also das gef-, gefühlte Gewicht ist enorm. #00:47:34-8#

Teilnehmerin 3: Ja. #00:47:33-3#

Teilnehmerin 1: Muss man wirklich so sagen. #00:47:35-1#

Interviewer: Okay. Das heißt ein Punkt ist das Gewicht. Gibt's noch n andren Punkt, warum sie froh sind wenn die dann mal weg ist? #00:47:38-2#

Teilnehmerin 3: Und im Sommer, dann schwitzt man halt n bisschen viel. (Interviewer: mhm (bejahend)) Und man kriegt auch schon mal so rote Stellen halt. (Interviewer: Ja.) Das ist, das ist halt so. Und das- #00:47:49-4#

Interviewer: mhm (bejahend). Und mit den- #00:47:51-2#

Teilnehmer 2: Stich- #00:47:51-3#

Interviewer: Ja? #00:47:51-3#

Teilnehmer 5: Stichwort Sport ist eigentlich (Interviewer: mhm (bejahend)) wo man (..??) #00:47:51-5#

Interviewer: Ja, ich würd gerne einmal nochmal- #00:47:54-9#

Teilnehmer 5: Ja, ja. #00:47:55-9#

Interviewer: (An Teilnehmer 3 gewandt) Sie haben gesagt mit den Kindern. Aber ist das nur mit den Kindern im Pool. Oder allgemein wenn sie mit ihren Kindern draußen spielen? #00:48:01-3#

Teilnehmerin 3: Allgemein. Also mit, also wie schon gesagt, also (Interviewer: mhm (bejahend)) mit Kindern sollte schon. Also mit Kindern bin ich auch sehr vorsichtig. (Interviewer: mhm (bejahend)) Also zieh ich die Prothese auch aus. #00:48:08-9#

Interviewer: Und-. Okay. Aber ist das die Angst, dass die Prothese irgendwie

verletzen kann? Oder woran genau liegt das? #00:48:13-9#

Teilnehmerin 3: Ja, es ist schon passiert. Deswegen. Also ich hab den schon mal (Interviewer: mhm (bejahend)) am Arm dann gepackt. Und wollte gar nicht so fest fassen. (Interviewer: mhm (bejahend)) Und man probiert ja auch das Beste (aus?) der Prothese. (Interviewer: Ja.) Aber man soll es (..??) wenn se noch Kleinkind sind, dann (Interviewer: mhm (bejahend)) besser ohne Prothese. #00:48:26-7#

Interviewer: Okay. Das heißt- #00:48:28-2#

Teilnehmerin 3: (..?) Angst. Es ist die Angst. (Interviewer: Ja. Ok.) Richtig. #00:48:28-8#

Interviewer: Aber das heißt, da war nicht mehr das, dass die eben hart ist. Und irgendwo dagegen gestoßen ist. Sondern dass die einfach zuge-, stärker zugegriffen hat als sie sollte? #00:48:34-2#

Teilnehmerin 3: Ja. Je nachdem was man macht. Genau. #00:48:36-7#

Teilnehmerin 1: Also es kann schon kneifen. Und gerade Kleinkinder sind (Interviewer: mhm (bejahend)) da glaube ich nicht so empfänglich für. Also das tut schon weh. #00:48:42-7#

Teilnehmer 6: Kann auch nem Erwachsenen schon mal wehtun. #00:48:43-7#

Teilnehmerin 1: Ja. Ich hab (Name vom Orthopädietechniker) also auch mal so in Oberschenkel versehentlich gekniffen. (Teilnehmer ?: mhm (bejahend)) Und das tut dann schon gut. #00:48:49-5#

Interviewer: Okay. #00:48:50-3#

Teilnehmer 6: Man lebt, man merkt dass man noch lebt (lacht). #00:48:53-0#

Interviewer: Jetzt wollten Sie noch (an Teilnehmer 5 gewandt) glaube ich mit dem Sport? #00:48:56-2#

Teilnehmer 5: Ja, ich (steh voll?) auf Fußball. Ja ich spiel auch Fußball. Und hab äh (Interviewer: mhm (bejahend)) jahrelang mit Prothese gespielt. Und äh irgendwann hat äh mein Trainer auf ner Party dann zu mir gesagt: „Zieh das Scheißteil aus! Weil du spielst viel besser wenn du keine hast.“. (Interviewer: mhm (bejahend)) Und ähm ich spiel seit, aufgrund dieser (lacht) Aussprache ähm ich spiel jetzt seit ungefährt drei Jahren ohne. (Interviewer: mhm (bejahend)) Ähm und merke einfach, dass es vom Kopf her ne andere Sache ist. (Interviewer: mhm (bejahend)) Allein wenn ich in Zweikampf reingehe. Ich mein ich spiele nicht professionell Fußball. (Interviewer: mhm (bejahend)) Ähm hab nie hoch gespielt. Aber jetzt auch nur noch hobbymäßig. Aber ich merk einfach (Interviewer: Ja.), das ist was anderes, wenn ich in Zweikampf reingeh und keine Prothese trage. Als wenn ich mit reingehe. Dann versuche ich wirklich nochmal, hab (Interviewer: mhm (bejahend)) schon mal versucht die so’n bisschen zur Seite zu ziehen. (..?) Wollte niemand verletzen. Das war immer so mein Hintergedanke. „Oh jetzt bloß niemandem wehtun dabei!“ (Interviewer: mhm (bejahend)) Und jetzt ist es egal. Weil ich jetzt weiß ich

hab nichts ähm mehr was, was der andere auch irgendwie (Interviewer: mhm (bejahend))-. Ich hab kein, kein Vor- oder Nachteil. (Interviewer: Ja.) Es ist jetzt wirklich nur n Zweikampf (Interviewer: Ja.) auf Augenhöhe. Wenn man das überhaupt so (..?) kann. #00:49:42-3#

Interviewer: Ja. Aber das heißt der Punkt war, die Angst jemanden zu verletzen? #00:49:47-9#

Teilnehmer 5: Genau, ja. #00:49:47-4#

Interviewer: Und war das aber ne begründete Angst? Also ist das schon mal passiert? Oder ist das einfach so ne Kopfsache, dass es halt einfach- #00:49:52-6#

Teilnehmer 5: Ich glaube das war eher ne Kopfsache. Aber ich hab ähm wirklich nie jemanden verletzt. Aber ich bin ja auch nie so in Zweikämpfe (Interviewer: Ja.) reingegangen wie ich's sonst normalerweise tun würde. (Interviewer: Ja, ok. mhm (bejahend)) Mit zwei Händen. #00:50:00-5#

Teilnehmerin 1: Aber das größte Problem, glaube ich, ist- #00:50:01-8#

Teilnehmer 5: Und ich, ich hab tatsächlich auch mal ne Rückmeldung bekommen. Von, von nem Gegenspieler. (Interviewer: mhm (bejahend)) Ne? Der gesagt hat äh „Bist du bescheuert? Du spielst hier mit Prothese und willst-. Was, was passiert wenn wir äh ähm wenn, wenn ich mich jetzt verletze deswegen?“, ne? (Interviewer: mhm (bejahend), ja.) Da war ich auch ganz irritiert. Weil ich mich gefragt habe: Oh äh, hab ich gar nicht drüber nachgedacht, dass (Interviewer: mhm (bejahend)) andere da irgendwie ne Gefahr sehen. #00:50:16-7#

Interviewer: Ja, ok. mhm (bejahend) #00:50:19-1#

Teilnehmerin 1: Aber die größte Gefahr ist, dass ich selber auf das Ding drauffalle. Weil dann ist's nämlich hin. (Teilnehmer 2: Ja, ja. Genau.) (Teilnehmer 4: mhm (bejahend)) Deswegen wird die wahrscheinlich eher geschont. Sag ich mal. Weil Mitspieler kann man mal eben schnell- #00:50:28-3#

Teilnehmerin 3: Klatschen. (alle lachen) #00:50:30-4#

Teilnehmer 6: (..??) #00:50:31-5#

Teilnehmerin 1: Der holt auf jeden Fall erstmal Luft dann. #00:50:31-4#

Interviewer: Gibt's noch irgendwo ne Situation wo die weggelassen wird? Wo die nie geb-, genutzt wird? Wo die auch einfach vielleicht unnütz ist? #00:50:41-2#

Teilnehmer 4: (..??) (durcheinander sprechen) #00:50:39-6#

Teilnehmer 2?: Also ich hab se auch beim Sport. (durcheinander sprechen) Tschuldigung. Beim Sport weggelassen. (Interviewer: mhm (bejahend)) Ich hab früher äh Badmington gespielt. Auch vereinsmäßig. (Interviewer: mhm (bejahend)) Ähm und da hab ich sie auch immer weggelassen. Weil mir das äh, ich mein ich würd ich da keine anderen von mit verletzen. Aber einfach vom, vom Gefühl her.

Weil man da ja auch einfach viel routiert. Sag ich mal. Und wenn man dann schwitzt äh dann hätt's auch sein können, dass die mal durch die Gegend fliegt. Und äh das wollte ich natürlich vermeiden. Obwohl das natürlich mit dem-. Äh einerseits wär's für'n Aufschlag, sag ich mal, wenn man ja so'n Aufschlag macht, so (Führt Bewegung vor). (Interviewer: mhm (bejahend)) Normalerweise äh hilfreicher gewesen. Aber ich hab das immer mit einer Hand gemacht. (Interviewer: Ja.) Und das hat auch gut funktioniert. Auch Tennis hab ich gespielt. Also das äh ging auch immer (Interviewer: mhm (bejahend)) ohne Prothese. Und war mir auch, auch allein aus ähm ja, wegen schwitzen udn sowas, wesentlich angenehmer. (Interviewer: mhm (bejahend)) Und heute beim Fahrrad fahren und sowas. Fahr ich natürlich nur mit Prothese. (Interviewer: Ja.) Weil man einfach auch diese Haltung natürlich dann (Interviewer: Ja.) entsprechend äh besser hat. Und den Griff hat. #00:51:35-4#

Interviewer: Aber das heißt, einerseits das Schwitzen. Und andererseits haben Sie gesagt, die Angst dass es einfach dann nicht mehr richtig hält? Dass sie halt auch einfach runterfällt? #00:51:42-5#

Teilnehmer 2: Ja. Also wenn, wenn man, also ich hab's auch wenn ich stark schwitze, sag ich mal, dass sich das äh Schwitzwasser hier in der Prothese sammelt (Interviewer: mhm (bejahend)). Und äh, ich sag mal, wenn man dann was anheben würde zum Beispiel, was Schweres, dann würd'se wegziehen. (Interviewer: Ja.) Äh und äh ich sag mal, beim Sport würd's vielleicht auch passieren. Ich hab's nicht ausprobiert aber (Interviewer: mhm (bejahend)) äh vielleicht dass sie da abrutscht oder was- #00:52:02-5#

Interviewer: mhm (bejahend), ja. #00:52:04-7#

Teilnehmer 6: Hängt auf einmal so ne Hand am Koffer. Und- (Mehrere TA: Ja, mhm (bejahend)) #00:52:06-4#

Teilnehmer 2: Ja, zum Beispiel (lacht). #00:52:06-1#

Teilnehmerin 1: Also die Problematik ist beim starken Schwitzen, egal was. Oder bei, bei verschiedenen sportlichen Aktivitäten verliert man das Ding definitiv. (Interviewer: mhm (bejahend)) Hält <u>nicht</u>. Weil die Befestigung halt einfach nicht so ist wie eine gewachsene Hand. Das ist halt das Problem. Es hängt alles, (Mehrere Teilnehmer: mhm (bejahend), ja genau, ja.) es hängt ja alles (Interviewer: Ja.) letztendlich an den Weichteilen. (Interviewer: Ja, klar.) Äh und die kann man dann anschnüren wie man möchte. Das ist völlig Latte. Äh (Interviewer: mhm (bejahend)) das einzige was man dann schafft, ist dass man n Blutsturz kriegt. Aber ansonsten (Interviewer: mhm (bejahend)) halten tut trotzdem nicht. Sobald man star anfängt zu schwitzen, rutscht das Ding. (Interviewer: Ja, ok.) (durcheinander sprechen) #00:52:34-9#

Teilnehmer 2?: Ich hab extra einen Carbon(..?) vorne drin, damit ich ähm (Durcheinander sprechen)- #00:52:39-4#

Interviewer: Ganz kurz einmal, (an die murmelnden Teilnehmer gewandt) wenn wir kurz nicht nebenher-. Weil das nachher wirklich schwierig-. Danke. #00:52:42-2#

Teilnehmer 2?: Ich hab auch noch ne Carbonspange hier vorne drin. Damit ich äh auch, auch schwere Sachen halten kann. (Interviewer: Ja.) Wie zum Beispiel

schwere Taschen. Dass das möglich ist. Äh aber auch da. Wenn ich stark schwitze hilft das auch nicht mehr. (Interviewer: mhm (bejahend)) (..?) wenn ich ne Wasserkiste trage damit (Interviewer: mhm (bejahend)) und ich stark schwitze, dann äh muss ich auch irgendwann aufpassen. Und äh das Gewicht wirklich auf die linke Seite verlagern (Interviewer: Ja.), damit ähm, damit die Prothese nicht abrutscht. #00:53:00-4#

Interviewer: Ja, ok. #00:53:03-3#

Teilnehmer 6: Da hab ich kein Problem mit. Weil ich bin Schulter Ex. Ich (..?) (Interviewer: mhm (bejahend)) #00:53:06-7#

Interviewer: Herr (Name von Teilnehmer 4 aus Datenschutzgründen ausgelassen) Sie wollten vorhin noch glaube ich hinzufügen? #00:53:09-5#

Teilnehmer 4: Naja, bei mir ist ja auch so. Grobe, schwere Arbeit (Interviewer: Ja.) und danach geht das auch nicht. (..?) zum Beispiel Holz hacken, sowas. Geht kaputt. (Interviewer: Ja.) Da muss ich dann irgendwie-. Ich hab noch eine Schmuckprothese (Interviewer: mhm (bejahend)) die bisschen Greiffunktion hat. (Interviewer: mhm (bejahend)) Wenn die kaputt geht, ist nicht so schlimm. (Interviewer: Ja.) Ja und dann (schicke?) ich ein. (Interviewer: mhm (bejahend)) Befestigung ist ja auch (Interviewer: mhm (bejahend))- Das sind Weichteile. Gurt, schwitzen (Interviewer: mhm (bejahend)) also. Aber sonst. Naja und im Wasser natürlich, kann man nichts machen. (Interviewer: mhm (bejahend)) Und man hat auch Angst, dass man jemanden verletzt. (Interviewer: Ja.) Ich hab schon einen verletzt. Aus versehen, auf der Arbeit. (Interviewer: mhm (bejahend)) (..??) auch, dass (..?) der eine wollte mich angreifen. Hab ich mich nur mit der Schulter bewegt. Ja. Hab ich (sie?) die Finger gebrochen. (Interviewer: Oh Gott.) Angreifer. #00:53:58-9#

Interviewer: Ja. Aber das heißt bei groben Arbeiten (Teilnehmer 4: (..??)) benutzen Sie nicht keine Prothese, sondern Sie benutzen dann einfach ne andere? #00:54:04-4#

Teilnehmer 4: Ja. Andere. Oder paar Hilfsgeräte, wenn das richtig schwere (..?) Arbeit (Interviewer: mhm (bejahend)). Schraub(zwingen?) oder noch was. (Interviewer: mhm (bejahend), ja.) Maschinen, oder zum Machinen bedienen ist auch hier zu viel (Interviewer: Ja.) Vibration (Interviewer: Ok.). Deswegen (..??) muss man (..?). #00:54:18-4#

Interviewer: mhm (bejahend) und was für Hilfsmittel gibt's, die Sie dann benutzen? #00:54:23-9#

Teilnehmer 4: Da mach ich meistens (..?). Schraubenzieher. Schraubstock. (Interviewer: mhm (bejahend)) Weiß (nicht?) (..??). (Interviewer: Ja.) man kann das (..?). Naja, so Bohrmaschinen und sowas. (Interviewer: Ja.) Ja. Die Geräte die man, ne? Muss man schon selber drauf kommen. (Interviewer: Ja, ok.) Was man braucht. (Interviewer: mhm (bejahend)) Zum Beispiel ein Nagel in die Wand. Da mach ich erstmal kleines Loch vorn. Nagel reinstecken. (Interviewer: mhm (bejahend)) In die Wand schlagen. (Interviewer: Ja.) So Kleinigkeiten ne? #00:54:49-6#

Interviewer: Das heißt aber das ist nichts, was sie dann eben kaufen oder bekommen. Sondern Sie überlegen selber wie man das überbrücken kann? #00:54:56-8#

Teilnehmer 4: Ja. (Interviewer: Ok.) Oder mit (Name von Orthopädietechniker aus Datenschutzgründen ausgelassen) zusammen nachdenken. Was kann man machen? Dann gibt's (..?) nachdenk. (Interviewer: Ja.) (Man?) findet schon ne Lösung. (Interviewer: Ja, ok. mhm (bejahend)) #00:55:06-9#

Teilnehmerin 1: Statt Hammer, Nagelautomaten. #00:55:07-0#

Teilnehmer 4: Ja, hab ich auch (schon?) (alle lachen) #00:55:12-8#

(Durcheinander sprechen, Gelächter) #00:55:15-6#

Teilnehmer 4: Ja, geht auch. #00:55:15-7#

Teilnehmer 6: (..???) #00:55:17-8#

Interviewer: Okay dann ich glaub jetzt hat jeder dazu was gesagt. Wenn's nichts mehr hinzuzufügen gibt ähm dann sind wir jetzt tatsächlich auch schon weit fortgeschritten in der Zeit. Dann würde ich sagen wir machen jetzt die kurze Pause. Einfach (..?) und ähm unterbrechen. Und dann würd ich sagen, weiß nicht, 15 Minuten? Oder ist das zu lang? Wie viel- #00:55:36-9#

Mehrere Teilnehmer: (zustimmendes Raunen) #00:55:39-2#

Pause #00:55:39-2#

Interviewer: Okay, dann äh (Teilnehmer ?: (..?) würde ich gerne jetzt als nächstes, Sie haben, vorher haben wir drüber geredet, was ist wenn, also wann die Prothese nicht genutzt wird. Jetzt wüsst ich gerne was ist, wenn die kaputtgeht? Was macht man dann? Gibt's irgendwelche Hilfsmittel? Wie geht man damit um? Wenn Sie dazu irgendwas erzählen könnten. #01:14:07-3#

Teilnehmerin 1: Dann nimmt man die andere. #01:14:12-1#

Mehrere Teilnehmer durcheinander: Ja #01:14:12-7#

(lachen) #01:14:15-3#

Teilnehmerin 1: Also ich kann ohne Prothese defiinitiv gar nichts machen, weil ich mir einfach angewöhnt hab, (eine?) Prothese täglich zu nutzen. Das ist mein Problem. (Interviewer: mhm (bejahend), ja.) Das heißt äh ich quäl mich dann schon wirklich ab. Äh ne Hose anzuziehen oder sonst irgendwas. Weil ich's mir so dermaßen angewöhnt habe, dass ich ohne nicht zurechtkomme. (Interviewer: mhm (bejahend)) Muss aber dazu sagen, dass ich in der glücklichen Lage bin, dass ich mehrere zu besitzen als eine. (Interviewer: mhm (bejahend)) #01:14:34-1#

Interviewer: Das heißt Sie können einfach auf ne andere zurückgreifen und dann-

#01:14:37-1#

Teilnehmerin 1: Ich muss. (Interviewer: mhm (bejahend)) Weil sonst ist der Tag für mich gelaufen. (Interviewer: Ja.) Ich hab äh ne Eintragung im Führerschein, wo drin steht ich muss ne Prothese tragen. Und das heißt nicht im Kofferraum, sondern am Körper. Und die muss auch wenigstens funktionieren. Ansonsten kann ich kein Auto fahren. (Interviewer: mhm (bejahend)) Kann ich kein Auto fahren, kann ich nichts machen. Das ist mein Problem. #01:14:52-3#

Interviewer: Und wenn Sie die anderen benutzen, ist trotzdem der Alltag irgendwie eingeschränkt? Oder ist dann alles ganz normal möglich? #01:14:58-5#

Teilnehmerin 1: Ich hab da baugleiche Prothesen. (Interviewer: mhm (bejahend)) So dass ich also im Prinzip dann letztendlich die andere nutze. Also eine ist eigentlich ständig in Reparatur. (Interviewer: mhm (bejahend)) (Oder?) (Name von Orthopädietechnikr aus Datenschutzgründen ausgelassen)?   #01:15:09-6#

Orthopädietechniker: (..??) #01:15:08-2#

Teilnehmerin 1: Ach so, das war's.   #01:15:11-4#

(Durcheinander sprechen) #01:15:10-9#

Interviewer: Wie's das bei den anderen? Was ist wenn die Prothese kaputt geht? #01:15:14-5#

Teilnehmer 6: Also ich kann mich dem (Name von Teilnehmer 1) nur, nur anschließen. (Interviewer: mhm (bejahend)) Wenn meine Prothese kaputtgeht, dann bin ich erstmal aufgeschmissen. (Interviewer: mhm (bejahend)) Dann bin ich im Arsch. So. Ich bin aber auch in dem selben Umfang glücklich (Interviewer: mhm (bejahend)), ich kann nämlich zuhause (..??) und dann dem Herrn (Name von Orthopädietechniker aus Datenschutzgründen ausgelassen) (dann um die Ecke bringen?). #01:15:34-6#

Interviewer: Und auch die ist von der Bauweise her so, dass es irgendwie- #01:15:35-8#

Teilnehmer 6: Ich hab drei (..?) bewusst gleiche (gesetzt?). (Interviewer: mhm (bejahend)) Nur n bisschen was verändert. Und ne Kleinigkeit verändert. (Interviewer: mhm (bejahend)) (..?) gleichen Prothesen, ja.   #01:15:43-3#

Interviewer: Okay, ja. Und bei den anderen? #01:15:47-2#

Teilnehmer 2: Also ich hab im Moment, sag ich mal, auch ne Ersatzhand. Oder n Ersatzarm. Ähm hab ich jahrelang nie gehabt. Also es ist, für mich ist das schon Luxus. (Interviewer: mhm (bejahend)) (lacht). Ähm aber ich könnte jetzt, ohne Prothese wäre ich jetzt auch nicht so sehr glücklich. (Kinderstimme im Hintergrund) Weil ich kann mir schon helfen. (Interviewer: mhm (bejahend)) Also das es ist kein Problem. Aber ähm so im Alltag wär's schon blöde. (Interviewer: mhm (bejahend)) Sag ich mal. Sonst früher war das so, wenn die kaputt war bin ich halt hierher gefahren. Hab die reparieren lassen. (Interviewer: mhm (bejahend)) Wenn's,

meistens ging's ja auch sofort früher. Aber heute bei der Elektronik und bei den Händen (Interviewer: mhm (bejahend)) ist das leider ja nicht mehr machbar. Dann müsste man länger drauf verzichten. Und das wäre nicht mehr so gut. #01:16:22-7#

Interviewer: Ja. Ja. Und bevor Sie die Ersatzhand hatten, gab's dann irgendwelche Hilfsmittel oder so, auf die Sie zurückgegriffen haben? #01:16:28-4#

Teilnehmer 2: Nö. #01:16:26-6#

Interviewer: Okay, ja. #01:16:27-8#

Teilnehmer 2:Gab's nicht. #01:16:30-3#

Interviewer: mhm (bejahend) Und bei den anderen? #01:16:34-0#

Teilnehmer 4: Ich hab eine Schmuckprothese. Zu Ersatz, wenn die kaputt geht. (Interviewer: mhm (bejahend)) Meistens hab ich nur die. Schmuck hängt zuhause, wenn die kaputt geht. (Interviewer: mhm (bejahend)) Kann ich aber nicht mehr viel machen. (Interviewer: Ja.) Naja. Bin aber auch gewohnt, auch ohne Prothese und na ich hab so ein Küchenhelfer. Wo man Brot festmachen kann. (Interviewer: mhm (bejahend)) Und (..?). (Interviewer: mhm (bejahend)) Aber sonst, Schmuckprothese nur für die Schönheit. (..??) #01:17:02-0#

Interviewer: Ja ok. Aber die Funktionen bleiben eingeschränkt? #01:17:03-0#

Teilnehmer 4: Ja. #01:17:01-5#

Teilnehmerin 1: Ja Schmuckprothese ist Schmuck. (Interviewer: mhm (bejahend)) (Teilnehmer 4: Ja.) Die nützt ja nichts. (Interviewer: Ja.) #01:17:06-8#

Interviewer: Und wie ist es aber, können Sie differenzieren oder sagen welche Situationen dann besonders schwerfallen. Wenn die Prothese wegfällt. Wann die besonder fehlt? #01:17:16-1#

Teilnehmer 4: Naja bei viele Kleinigkeiten. Nicht? (Rechte?) Hand zum Wasser Aufmachen. (Interviewer: mhm (bejahend)) Brot schmieren. Hose anziehen. (Interviewer: Ja.) Ja. (..??) #01:17:24-1#

Teilnehmerin 3?: Alltägliches Leben oder? #01:17:27-9#

Teilnehmer 4: Ja (auf Dinge?) was man gar nicht so achtet. (Interviewer: mhm (bejahend)) Und ja. Aber sonst naja, kann man schon ohne. Stirbt man nicht. (Interviewer: mhm (bejahend)) Aber (mir fällt?) alles langsamer. (Interviewer: Ja, klar.) Deswegen. Alles was man macht ist noch langsamer. (Interviewer: mhm (bejahend)) (..??) #01:17:42-6#

Interviewer: Aber Sie kommen trotzdem alleine zurecht? Langsamer aber Sie schaffen's? #01:17:46-2#

Teilnehmer 4: Ja, ja. #01:17:46-2#

Interviewer: Ja, ok. #01:17:47-3#

Teilnehmer 5: Ich hab auch wie Herr (Name von Teilnehmer 4) noch eine Schmuckprohese zuhause (Interviewer: mhm (bejahend)). Die ich dann äh anziehen kann. Jetzt ähm beruflich würde ich das tun, ja. (Interviewer: mhm (bejahend)) Um das einfach optisch auszugleichen. (Interviewer: mhm (bejahend)) Ähm aber zuhause wär's jetzt für mich kein Problem. Weil ich die anderen Sachen auch so machen (Interviewer: Ja.) könnte. (Interviewer: Ja.) Also hab da auch keine weiteren Hilfsmittel die ich irgendwie benutze. (Interviewer: Ja.) #01:18:04-5#

Interviewer: Das heißt das ist eben nur dieses, das Auftreten und das Optische? #01:18:08-2#

Teilnehmer 5: Genau, ja. Also das würde ich mich-. Vielleicht ist das auch nur ne Frage des Kopfes. Ich mein ich könnte ja auch letztendlich ohne Prothese in die Schule gehen. (Interviewer: Ja.) Das äh würde auch nur n paar Wochen oder Monate dauernd, bis sich die Schüler dann und die (Interviewer: ja.) Kollegen dran gewöhnt hätten. Ähm aber es, ich hab's halt noch nie gemacht. Von daher ist das ne große Hürde das mal zu tun. (Interviewer: Ja, klar.) Von daher brauche ich diese Schmuckprothese, um einfach (Interviewer: Ja.) komplett in die Schule zu gehen. (Interviewer: Ja.) Also für mich ist das irgendwie so'n, so'n Körpergefühl auch. Dass ich weiß jetzt bin ich komplett. So kann ich ähm mich präsentieren. (Interviewer: mhm (bejahend)) So kann ich (Teilnehmer 4: (??)) irgendwie vor andere Leute treten. Äh ohne da wär's irgendwie, ich würde mich n bisschen nackt fühlen. Im ersten Moment. #01:18:39-7#

Interviewer: Ja. #01:18:40-6#

Teilnehmer 5: Auch wenn ich gefragt werde ähm: „Können Sie mal ausziehen?“. Dann sag ich immer: „Wir kennen uns noch nicht so gut.“ (lacht). Ich würd dich jetzt auch nicht fragen irgendwie n Teil von dir auszuziehen. #01:18:48-3#

Teilnehmerin 1: Ich schon. #01:18:50-4#

Teilnehmer 5: Äh ich brau-, ich brauch zwei Hände (lachen). Das muss erst ne gewisse vertraute Atmosphäre dann sein. #01:18:53-8#

Interviewer: mhm (bejahend), okay das heißt es wirklich was, was halt einfach das Körperbild vollständig macht und dazugehört? #01:18:57-3#

Teilnehmer 5: Ja. Ja. #01:19:00-9#

Interviewer: mhm (bejahend), okay. #01:19:00-0#

Teilnehmer 5: Weil's einfach auch seit meinem dritten Lebensjahr dazugehört (Lachen anderer TA). #01:19:05-0#

Teilnehmer 4: Das wird aber keiner merken, wenn du die nicht anziehst. (Teilnehmer lachen) (..??) #01:19:10-1#

Teilnehmer 6: Also, also ganz ehrlich ähm früher haben meine Hände auf der Theke gelegen. (Teilnehmer 4 (lacht)) Und die danach gefragt haben, sind dann (unten?) gelegen. (lacht) #01:19:20-5#

Interviewer: mhm (bejahend) #01:19:21-7#

Teilnehmerin 1: Kannst du ne (Handlage?)? Ja sicher. (lacht) #01:19:26-2#

Teilnehmer 6: (..??) #01:19:30-2#

Interviewer: Ähm (Teilnehmer 6 (..??) Ja? #01:19:31-1#

Teilnehmer 6: Um mit der Prothese irgendwie (..?), können, muss man dagegen nen sehr schwarzen Humor bekommen. (Teilnehmer 4: Ja.) #01:19:36-9#

Interviewer: Ist das nötig? #01:19:37-4#

Teilnehmer 6: Ja. #01:19:39-3#

Interviewer: Okay. (lacht) Ähm und bei Ihnen Herr (Name von Teilnehmer 3 aus Datenschutzgründen ausgelassen)? Wenn die (Teilnehmer 3: „Also ich, ich habe-) Prothese wegfällt? #01:19:44-0#

Teilnehmerin 3: Auch Erzsatz äh Prothesen zuhause. Also ich hab's auch geschafft, die Ersatzprothese auch in vier Wochen kaputt zu kriegen. (Interviewer: Ok.) Und dann hab ich von (Name von Orthopädietechniker aus Datenschutzgründen ausgelassen) halt ne Leihhand gekriegt. (Interviewer: mhm (bejahend)) Also- #01:19:55-1#

Interviewer: Das heißt- #01:19:55-1#

Teilnehmerin 3: Ich bin auch gut versorgt. #01:19:56-6#

Interviewer: Und die ist dann aber auch von der Art und Weise gleich? Also es ist nicht nur ne Schmuckprothese oder so, sondern die kann das was Ihre jetzt kann? #01:20:02-4#

Teilnehmerin 3: Genau. Alles gleich. (Interviewer: Okay, mhm (bejahend)) (Kinderstimme im Hintergrund) #01:20:03-3#

Interviewer: Und wenn Sie das nicht hätten? Würde's trotzdem funktionieren? #01:20:08-7#

Teilnehmerin 3: Pf. #01:20:08-3#

Teilnehmerin 1: Nichts mit Arbeit oder? #01:20:09-4#

Teilnehmerin 3: (..??) nicht mit Arbeit. (Interviewer: mhm (bejahend)) Mit Arbeit schon mal gar nicht. (Interviewer: Ja.) Und äh, und gar nichts (..?). (Interviewer: Ja.) Kein Auto fahren. Kein Fahrrad fahren. (..?) Draußen mit den Kindern spielen.

(Interviewer: Ja, ok.) Das geht. #01:20:25-5#

Interviewer: Okay. Dann würde ich gerne jetzt als nächstes wissen, wenn Sie sich vorstellen würden, nach dem Ganzen was wir jetzt gesammelt haben an Pro und Contra und so weiter, ähm dass Sie die Möglichkeit hätten mal auf die Entwicklerseite zu gehen. Und die Prothese so verändern könnten, wie Sie das wollen würden. Auch ohne jetzt technische Grenzen. Sondern wir stellen uns vor Sie können zaubern und machen sich die perfekte Prothese. Wie würde die sein? Wie müsste die aussehen? #01:20:49-3#

Teilnehmer 1: Zack, fertig, so (zeigt auf seine normale Hand). #01:20:51-0# #01:20:51-7#

Durcheinander sprechen

Interviewer: Was heißt das denn „genau so"? #01:20:52-2#

Teilnehmer 6?: (..?) Hand. #01:20:54-6#

Interviewer: mhm (bejahend) Und was macht aber, also- Okay wie ne Hand. Und wenn wir uns das dann doch technisch vorstellen. Was ist das, also was sind die entscheidenden Punkte, die so ne Prothese ner Hand ähnlich machen? Ist das die Optik? Ist das die Funktion? Was bringt die da an- #01:21:08-2#

Teilnehmer 6: Sie (sehen?) eine gesunde, eine gesunde Hand. Und ne Prothese im Gegensatz (Interviewer: Ja.)- #01:21:15-6#

Interviewer: Ja. Und wenn wir aber möglichst nah rangehen wollen? Was sind dann, was ist da wichtiger? Ist das die Funktion? Ist das die Optik? (Teilnehmer 2?: Das schaffen se, das schaffen se technisch nicht.) (Teilnehmer 1: Geht nicht) Okay, aber wir gucken mal nicht darauf was wir nicht schaffen. Sondern wir überlegen mal was sie haben müsste, ähm damit sie möglichst perfekt wäre. #01:21:28-3#

Teilnehmer 5: Also der Handschuh müsste für mich robust sein (reden im Hintergrund). Ja? Er müsste (Interviewer: Ja.) auch wiederstandfähig sein. Er müsste- #01:21:34-8#

Interviewer: Einen Moment. (Reden im Hintergrund) Herr (Name von Teilnehmer 4 aus Datenschutzgründen ausgelassen), ganz kurz, dass wir hier einmal die Aufnahme machen. #01:21:37-3#

Teilnehmer 5: Der äh Handschuh müsste leicht zu reinigen rein. (Interviewer: mhm (bejahend)) Das ist für mich immer ne, ich mein bei dieser Hand ist es jetzt besser als noch bei der Schmuckprothese. (Interviewer: mhm (bejahend)) Da (..?) war's wirklich so, dass die nach vier Wochen ähm so'n, so'n dunkelgründen Farbton angenommen hat. (Interviewer: mhm (bejahend)) Hier ist es leichter geworden. Aber auch hier ist jetzt n Kugelschreibabdruck drauf und den wieder abzukriegen-. (Interviewer: Ja.) Ich mein, es, es wird irgendwie gehen. Aber es verfärbt sich viel. (Interviewer: mhm (bejahend)) Und dann sieht das nach ner gewissen Zeit unsauber aus. (Interviewer: Ja.) Und das ist irgendwie äh also es halt, es wäre halt schön wenn's-. Das wurd grad schon gesagt, es soll so aussehen wie ne natürliche

Hand (Interviewer: mhm (bejahend)). Ähm wenn’s so nah wie möglich an diesen Farbton der, der natürlichen Hand herankommt wär’s schon gut. (Interviewer: mhm (bejahend)) Und wenn’s dann auch so bleibt. Und, und von der Reinigung her leicht (Interviewer: Ja.) äh, ja, so, so instand zu halten wäre. Ne? (Interviewer: Ja.) Wäre das, wäre das gut. Das würde ich mir wünschen. (Interviewer: Ja.) #01:22:19-3#

Interviewer: Und bei den anderen? #01:22:20-0#

Teilnehmerin 1: Ja zumindestens die Funktion annähernd einer normalen, menschlichen Hand. (Interviewer: Ok.) Da sind wir ganz weit von weg. Also selbst-. Ah ich kenne alle Prothesensysteme am Markt. Ich hab glaub ich alle getestet die’s gibt. Und ich habe noch nichts gefunden, was mich grundsätzlich dahingehend zufrieden stellen würde. Das ich sagen könnte das (Interviewer: mhm (bejahend)) wäre zumindest mal ne sinnvolle Alternative. Weil wie gesagt, die Problematik ist ganz einfach: Das ist’n Greifwerkzeug und sonst nichts. (Interviewer: mhm (bejahend)) Äh und das geht letztendlich glaub ich nicht mal-. Ich kenne Roboterhände. Äh die eine Vielzahl von Funktionen machen. (Interviewer: mhm (bejahend)) Äh aber das Problem ist, dass, dass man sie als Patienten nicht bedienen können. Und wenn ich dann n ganzen Computer hinter mir hertragen muss. Der dann letztendlich entscheidet was ich jetzt grade wie mache. Äh dann ist das für mich kein Prothese tragen. Sondern es ist n fremdgesteuert. Ist genau das gleiche (Interviewer: mhm (bejahend)) als wenn man Auto alleine fährt. Hätt ich auch nicht so gerne. Ich möchte schon auch vielleicht n bisschen selber bestimmen können was das Ding macht. (Interviewer: Ja.) Und äh die Problematik, dass die System die am Markt sind, es gibt wie gesagt Hightechsysteme. Äh für Roboter. Die ganz viele Sachen können. Es gibt auch tolle Filme im Internet. Kann man also sich äh reingoogeln. (Interviewer: mhm (bejahend)) Äh was für tolle Funktionen es gibt. Grade in A-, in Amerika. Was man nicht sieht ist, dass dahinter n ganzes Team sitzt von Leuten, die diese Prothese steuern. Weil der Träger, der da drin steht, das ist nur n, ne Schaufensterpuppe. #01:23:44-7#

Interviewer: Ja, okay. Aber um das mal als Punkte festzuhalten. Das heißt Sie haben gesagt es muss was sein, was ich selber bedienen kann. #01:23:49-0#

Teilnehmerin 1: Ja. #01:23:48-1#

Interviewer: Es soll keinen riesigen Computer hinsich-, hinter sich herschleppen. (Teilnehmer 1: Richtig.) Das heißt es muss eben klein sein. Und was muss es für Funktionen haben? Was sind die Funktionen, die sie zum Beispiel an so ner Roboterhand sehen, wo sie sagen: Ja sowas. Wenn der Computer und das nicht selber bedienen können nicht dabei wäre. Das wär das, was ich haben will. #01:24:03-4#

Teilnehmerin 1: Also ne zeitnahe Bewegung, dass ich irgendwo druntergreifen könnte mit den Fingern. (Interviewer: mhm (bejahend)) Das geht also momentan mit keinem einzigen System. #01:24:09-4#

Interviewer: Also druntergreifen, das heißt eben irgendwas aufheben? #01:24:13-0#

Teilnehmerin 1: Die Funktion die ich hier auch hätte (Interviewer: Ja.) äh also

irgendwo mal tatsächlich auch ne Funktion geht mit ner Prothese überhaupt gar nicht. (Interviewer: Ja.) So. Ich muss also überlegen, wie ich was greife. (Interviewer: mhm (bejahend)) Und ich kann eigentlich nur grobe Gegenstände halten. Ich kann se tragen. Äh ich kann sie irgendwohin buxieren. (Interviewer: Ja.) Äh es gibt aber keine Möglichkeit, mit ner einfachen Hand muss ich jetzt schon jonglieren. Um das Ding überhaupt zu kriegen. Weil, kann ich schon nicht greifen. (Interviewer: Ja.) Da geht's schon los. #01:24:36-1#

Interviewer: Okay. Also das Greifen. Dieses Aufheben. Gib- #01:24:39-3#

Teilnehmerin 1: Wenn ich so mache, funktioniert's natürlich. Ganz klar. (Interviewer: Ja.) Sieht auch immer toll aus. Ist aber keine Funktion für mich. (Interviewer: Ja.) #01:24:46-7#

Interviewer: Und was gibt's noch für Funktionen die wichtig wären? #01:24:47-2#

Teilnehmerin 1: Äh ne simultane Steuerung, die ich also selber bestimme. Dass ich also diese, dieses zeitversetzte (Interviewer: mhm (bejahend)), die zeitversetzte Arbeitsweise der Prothese nicht mehr habe. Das heißt also es sind (Interviewer: mhm (bejahend)) Mikrosekunden die das dauert. Bis das Ding überhaupt (Interviewer: Ja.) auf meine Muskelsignale reagiert. Äh und ich kann halt immer nur eins. (Interviewer: mhm (bejahend)) Entweder öffnen oder schließen. Oder umschalten. Links rum drehen. Oder rechts rum drehen. (Interviewer: mhm (bejahend)) Das ist zwar alles toll, dauert aber viel zu lange. (Interviewer: Ja.) Das heißt wir sind noch lange nicht in einem, in einem Zeitablauf wo man sagen könnte das funktioniert auch so wie ich das möchte. (Interviewer: mhm (bejahend)) #01:25:19-0#

Interviewer: Okay. Also es soll schneller sein. Dass die Übertragung eben sofort quasi passiert? #01:25:24-3#

Teilnehmerin 1: Ja. #01:25:24-1#

Interviewer: mhm (bejahend) #01:25:25-5#

Teilnehmerin 1: Es muss stabil sein. So dass ich also auch tatsächlich nicht an der ersten Wasserkiste die ich ins Auto stellen will scheiter. (Interviewer: mhm (bejahend)) Und irgendwas abgebrochen habe. Das ist momentan sicherlich eins der größten Probleme. (Interviewer: Ja.) Äh einen Daumen der von oben greift und tatsächlich auch Kraft ausüben könnte. (Interviewer: mhm (bejahend)) Wäre auch Klasse. Äh mehr Funktionen muss die Hand schon gar nicht haben. (Interviewer: Ja.) Aber von oben greifen, dass man also so (macht es vor) wäre toll. (Interviewer: Ok.) #01:25:46-9#

Interviewer: Also das sie quasi (Teilnehmer 1: Funktioniert aber auch noch nicht.) n Gegenstand zwischen die Finger und den Daumen einklemmen können? (Teilnehmer 1: Ja.) Wenn wir das als- #01:25:50-2#

Teilnehmerin 1: Also die Greiffunktion (Interviewer: Ja.) die jede normale Hand also auch kann. (Interviewer: mhm (bejahend)) Äh wäre toll. #01:25:58-2#

Interviewer: Okay. Jetzt Herr (Name von Teilnehmer 4 aus Datenschutzgründen ausgelassen), haben sie ganz am Anfang ähm in der ersten Fragerunde erwähnt, dass irgendne gleichzeitige Bewegung- #01:26:03-9#

Teilnehmer 4: Ja, zum Beispiel kann (..?), drehen und aufmachen. (macht es mit seiner Hand vor) (Interviewer: mhm (bejahend)) Das kann keine Prothese. (Interviewer: mhm (bejahend)) Gleichzeitig zwei Funktionen. Zum Beispiel drehen und aufmachen. #01:26:15-5#

Teilnehmerin 1: Würd auch nichts bringen im Alltag. Das ist das Problem. #01:26:17-8#

Teilnehmer 4: Ja. #01:26:19-2#

Interviewer: Wo, wo fehlt Ihnen sowas? Gibt's ne bestimmte Situation wo Sie sagen da wäre das super, wenn sie das könnte? #01:26:23-2#

Teilnehmer 4: Ja, dann könnte ich (Wasser trinken?) vielleicht. (Interviewer: Ok ja.) (lachen) #01:26:28-2#

Interviewer: Das man quasi festhalten kann und das- #01:26:31-5#

Teilnehmer 4: Oder auch Kugelschreiber halten, (..??) (Interviewer: Ja.) Muss die ganz nach hier paar Minuten alles (Interviewer: mhm (bejahend)) einstellen. Und dann ist das noch Silikon. Wohl sauber, aber sehr glatt. (Interviewer: mhm (bejahend)) (..??) Dass es dann besser greifen (..??) (Interviewer: mhm (bejahend)) Andere Material. Also PVC wird schnell dreckig. Kann man besser greifen. (Interviewer: mhm (bejahend)) Und das ist kosmetisch wunderbar. Kann man nicht (..?) (Interviewer: Ja.), funktioniert das nicht so. Aber sehr glatt. (Interviewer: mhm (bejahend)) #01:26:59-8#

Interviewer: Das heißt einmal, dass es rutschfest ist. Dass man was gut greifen kann. Und dann, dass einfach mehrere Griffe gleichzeitig möglich sind? #01:27:03-8#

Teilnehmer 4: Diese (Finger?) fehlt natürlich. (Interviewer: mhm (bejahend)) Und, dass ist ja (..?) kosmetisch (Interviewer: mhm (bejahend)) (..?) Greifzange ist das noch. (Interviewer: Ja.) Aber nicht einfache Werkzeug. (Interviewer: Ja.) Ja und das so'n bisschen schneller (..??) (Interviewer: mhm (bejahend)) (..??) schneller und dann bringt das (..??) #01:27:24-8#

Interviewer: Okay. Also auch die Geschwindigkeit. #01:27:24-4#

Teilnehmer 4: Man muss sich doch selber verlangsamen, dass die Prothese funktioniert. (Interviewer: mhm (bejahend)) Auch (..?) nicht so schnell. (Interviewer: Ja.) #01:27:32-5#

Teilnehmerin 1: Aber in der Theorie gibt's ja diese Möglichkeit, sag ich mal, durch eine klitzekleine OP, mit die Muskeln splitten zu lassen. Dann kannst du ja letztendlich drei Sachen gleichzeitg machen. #01:27:41-2#

Teilnehmer 4: Ja, aber- #01:27:44-4#

Teilnehmerin 1: Ich kenn jetzt keinen, der groß geklappt hat so. Äh das es dann tatsächlich funktioniert aber- #01:27:48-1#

Teilnehmer 4: (..??) (Interviewer: mhm (bejahend)) (..?) (Interviewer: mhm (bejahend)) Meine wiegt 4,5 Kilo. (Interviewer: mhm (bejahend)) Über vier Kilo ist schon (..??) Gewicht. (Interviewer: Ja.) Aber (..??) (Interviewer: Ja, klar. Natürlich.) (Kinderstimme im Hintergrund) #01:28:08-6#

Interviewer: Und wenn Sie jetzt aber gucken, von den ganzen Sachen die Sie genannt haben, was wär das Wichtigste für Sie? Von, an Veränderungen. Gibt's einen Punkt wo Sie sagen eine von den Sachen ist erstmal die Wichtigste? Das es die gibt- #01:28:17-5#

Teilnehmer 4: Ja die Greiffunktion. #01:28:18-8#

Interviewer: Das wär das Wichtigste? #01:28:19-9#

Teilnehmer 4: (..??) natürlich. #01:28:21-0#

Interviewer: Ja. #01:28:22-9#

Teilnehmer 6?: Die Geschwindigkeit. #01:28:24-6#

Teilnehmer 4: Und Geschwindigkeit. #01:28:23-8#

Interviewer: Okay ja. mhm (bejahend) #01:28:29-0#

Teilnehmer 4: Dass es so ähnlich wie die andere (sind?). (lacht) #01:28:29-9#

Interviewer: mhm (bejahend), ja. #01:28:32-2#

Teilnehmer 2: Zwei Sachen sind mir grad noch eingefallen, (Interviewer: mhm (bejahend)) äh einmal das, das Aufladen wär einfacher. Wenn man nicht jedes Mal den Handschuh runterziehen müsste. (Interviewer: mhm (bejahend)) Sondern wenn es kontaktlos gehen würden. Wie beim Handy auch. (Interviewer: Ja.) Ja, das ist so ne Kleinigkeit. Ähm und äh die Kontakte die innen drin sind. Dass man die irgendwie verändert. (Interviewer: mhm (bejahend)) Ähm das die keinen direkten Hautkontakt haben. (Interviewer: mhm (bejahend)) Und äh ich hab das ja vorhin erwähnt, (Interviewer: mhm (bejahend)) dass ich da ne allergische Reaktion hatte. (Interviewer: Ja.) Dass ähm, ja, ich glaub da gibt's ja auch schon Überlegungen. Oder auch schon äh- #01:28:55-7#

Teilnehmerin 1: Es gibt die schon. Es gibt in USA implantierte Elektroden letztendlich. Die aber auf'm deutschen Markt in etwa 10, 15 Jahren kommen werden. #01:29:02-5#

Teilnehmer 2: Ja. #01:29:01-2#

Interviewer: mhm (bejahend)   #01:29:02-1#

Teilnehmerin 1: Da geh ich mal von aus. Weil solange brauchenwir in Deutschland, bis wir in den Körper eingreifen dürfen.   #01:29:08-6#

Teilnehmer 2: Also ich würd gar nicht sagen, dass ich, dass in den Körper eingegriffen wird. Sondern dass, dass die, dass die Muskelsignale (Interviewer: mhm (bejahend)) die ja letztendlich ausschlaggebend sind. Dafür dass die Hand geoff-, geöffnet und geschlossen werden kann. (Interviewer: Ja.) Dass die aufgenommen werden können. Äh durch das Material hindurch. Und eben nicht mh (..??). #01:29:22-0#

Teilnehmerin 1: Gibt’s auch schon. Aber funktioniert ja noch nicht hun, 100 prozentig zuverlässig.   #01:29:25-9#

Teilnehmer 2: Aber das wünsch ich mir. Das war ja die Frage. #01:29:25-4#

Interviewer: Ja, genau. #01:29:27-9#

(lachen)   #01:29:29-2#

Teilnehmer 1: Hat aber nichts mit der Hand zu tun. Sondern mit den Elektroden. #01:29:31-7#

Teilnehmer 2: Ja. #01:29:30-6#

Interviewer: Aber es geht um alles. Das- (lacht) #01:29:33-6#

Teilnehmerin 1: Achso::: #01:29:35-1#

Interviewer: Aber, das heißt die Ursache für so’n Wunsch ist diese allergische Reaktion? Oder auch einfach die Funktion von den Elektroden, die Übertragung? #01:29:39-3#

Teilnehmer 2: Mit Sicherheit kann man dann auch ähm mithilfe einer anderen Übertragung ähm mehrere (Interviewer: mhm (bejahend)) Funktionen gleichzeitig übertragen. (Interviewer: mhm (bejahend))   #01:29:48-7#

Interviewer: Nee, aber ihre Idee kommt daher, dass eben diese allergische Reaktion- #01:29:52-0#

Teilnehmer 2: Ja! Ja klar, das war der Ausschlag.   #01:29:50-5#

Teilnehmerin 1: Okay, ja, mhm (bejahend). Ja, ok. #01:29:52-3#

Teilnehmer 2: Ausschlag. (lacht)   #01:29:56-3#

Teilnehmer ?: (..??) #01:29:57-0#

Teilnehmerin 1: Aber das hab ich jetzt also wie gesagt, selten gehört, dass äh jemand allergisch auf die Standardelektroden reagiert. Also- #01:30:04-1#

Teilnehmer 2: mhm (bejahend) #01:30:06-4#

Interviewer: Wie ist- #01:30:04-7#

Teilnehmerin 1: Also man reagiert auf den Hautkontakt an sich. Der Liner zum Beispiel. Das haben glaub ich alle. Dass man Rötungen kriegt von der Haut. Das sind aber Reibungen glaube ich, die durch das Tragen der Prothese kommen tatsächlich zum Tragen kommen #01:30:14-3#

Interviewer: Ja, ja das. Also das ist glaube ich einfach individuell abhängig. (Teilnehmer 1: Ja.) Manche Leute sind halt gegen die Stoffe allergisch. Genau. #01:30:20-6#

Teilnehmer 6?: (..??) ich glaube (..??) ähm (..??) #01:30:30-4#

Interviewer: Ja. #01:30:31-9#

Teilnehmer 6: (..??) #01:30:35-7#

Interviewer: Aber das heißt, das ist, haben Sie vorher auch schon gesagt, dass die einfach so doll reiben dass das einfach- #01:30:37-4#

Teilnehmer 6: (..?) die Körperbewegung (..??) #01:30:38-7#

Interviewer: Ja, klar. mhm (bejahend) #01:30:43-3#

Teilnehmer 5: Mir fällt noch was ein, (Interviewer: Ja.) Aber das gibt's glaub ich auch schon. (Oder?) zumindest ansatzweise, dass man selber die Hand individualiseren kann für bestimmte Momente. (Interviewer: mhm (bejahend)) Dass ich also (..?) für den Beruf brauch ich die und die Funktion. Also kann ich das einstellen. (Interviewer: mhm (bejahend)) Mit meinem Handy zum Beispiel. Sage (Interviewer: Ja.) Beruf. Da hab ich jetzt die Einstellung gespeichert. (Interviewer: mhm (bejahend)) Und möchte das dann abrufen. Beim Rechner zuhause (bin?) und was andres machen möchte. Kochen, essen (Interviewer: Ja.). Besteck. Dass ich dann einfach nur über die App sozusagen das einstellen (könnte?). (Interviewer: mhm (bejahend)) Und zack weiß die Hand sofort (..??)- #01:31:06-1#

Teilnehmerin 1: Aber eigentlich sollte man doch eine Hand entwickeln können, die also mit diesen beiden lächerlichen Muskeln die ma zur Verfügung hat, genau die Bewegung machen kann, die ich so auch konnte. Dann brauch ich nicht irgendwas für die Arbeit. Und irgendwas für, für mich privat. Sondern ich hätte einfach ne Hand mit der ich alles kann. Ohne irgendwo daran rumzufummeln. Sondern das Ding muss einfach so funkionieren, dass ich sagen kann okay, wenn ich jetzt koche, wenn ich schneide, wenn ich backe. Wenn ich egal was mache, dann muss das ein Prozess sein, dass es funktioniert. Sagen wir's mal so. #01:31:33-3#

Teilnehmer 5: Aber das ist glaub ich ne Wunschvorstellung. Dass- #01:31:34-6#

Teilnehmerin 1: Das geht nicht (…?). Deswegen sag ich ja, das wäre dann die Reimplantation. #01:31:38-8#

Interviewer: Okay. Aber wenn's- #01:31:41-9#

Teilnehmer ?: (…?) #01:31:42-4#

Interviewer: Also wenn's die Möglichkeit gäbe, dass Sie alles auf einmal haben können, hätten Sie lieber alles auf einmal oder hätten Sie lieber das, dass die sich eben je nach Umgebung das anpassen kann? #01:31:52-3#

Teilnehmer 5: Können Sie das nochmal präzisieren? Weil ich das nicht genau- #01:31:52-3#

Interviewer: Ja. Also zum Beispiel wenn man Ihnen jetzt sagen würde Ihre Hand kann die Griffe die sie haben wollen im Job. Und die kann aber auch gleichzeitig ohne irgendwas umzustellen die Griffe die man zum Beispiel beim Kochen, Besteck benutzen und so weiter braucht. Hätten Sie das lieber alles auf einmal wenn es möglich wäre? Oder wäre das schon gewünscht, dass man sagen kann (Teilnehmer 5: Dass ich differenzieren kann?) ich stell mir das ein. Genau. mhm (bejahend), ja. Also ich hab nicht immer alle auf einmal, sondern ich stell mir ein, was ich grade haben möchte. Und es sind nicht hunderte (..?) #01:32:17-9#

Teilnehmer 5: Ich glaub die Frage beantwortet sich irgendwie von alleine, oder? Ob ich dann sozusagen das (beste?) Paket automatisch habe. (Interviewer: mhm (bejahend)) Oder wechseln kann zwischen (..??)- #01:32:25-2#

Interviewer: Ja es gibt tatsächlich Leute, die sagen Sie haben lieber wenige. Weil das dann übersichtlicher ist. Deshalb frag ich. Genau. #01:32:29-0#

Teilnehmer 5: Das, das ist glaub ich ganz abhängig von anderen Faktoren noch, (Interviewer: mhm (bejahend), ja.) die, die bei dieser Frage mit berücksichtigt werden müssen. (Interviewer: mhm (bejahend)) Die jetzt gar nicht- #01:32:34-5#

Teilnehmerin 1: Ich glaube es sind, (..?) eigentlich die gleichen Griffe die man braucht. Mein ich. #01:32:40-6#

Teilnehmer 5: Es ist halt die Frage der Bedienbarkeit, ne? (Interviewer: mhm (bejahend), ja klar.) Wie einfach ist-. Das, kann, kann ich so nicht beantworten. #01:32:46-0#

Interviewer: Ja, ja. #01:32:45-2#

Teilnehmer 6: Aber oft ist auch weniger wert. #01:32:47-7#

Teilnehmer 5: Aktuell ja. Bei der Prothese (..??) #01:32:53-3#

Interviewer: mhm (bejahend). Gibt's noch andere Situationen die Ihnen einfallen, wo eben irgendne bestimmte Funktion fehlt? Wo Sie den Gedanken haben, das wär super, wenn die das könnte? #01:33:02-9#

Teilnehmer 2?: (Speicherfunktion?) (lacht) #01:33:08-0#

Interviewer: Inwiefern Speicherfunktion? #01:33:09-8#

Teilnehmerin 1: Implantiertes Handy. (lacht) #01:33:12-7#

Teilnehmer 2: (..??). Also jetzt ohne Witz. Wenn ich äh alle Medien (..?) speichern könnten. (Interviewer: mhm (bejahend)) (..?) (Interviewer: (lacht) die, das, bescheuert, weil ich noch nie darüber nachgedacht habe. Und ich könnte (Interviewer: mhm (bejahend)) darüber Sachen über'n Beamer präsentieren. Das hätte schon was. #01:33:25-5#

(lachen) #01:33:27-6#

Teilnehmerin 1: Aufgabe an dich! (An Orthopädietechniker gewandt) #01:33:27-7#

Teilnehmer 2: Wenn ich mir sowieso schon so'n Technikteil was (1000?) Euro kostet an meine Hand reinbauenl lasse, (..??). (Interviewer: Ja.) Das ist, das ist jetzt wirklich Spielerei. Ja?(Interviewer: Ja.) #01:33:37-9#

Teilnehmerin 1: Also ich seh das größte Problem tatäschlich auch wirklich in der Funktionalität der Steuerung. Also ich hab so viele programmierbare Geschichten probiert. Das ist alles toll. Äh aber es lenkt mich letztendlich von dem, von dem Wesentlichen wieder ab. #01:33:55-8#

Interviewer: mhm (bejahend) #01:33:56-5#

Teilnehmerin 1: Weil ich muss letztendlich irgendwo das Ding über mein Handy dann umschalten. Andere App. Äh um den nächsten Griff machen zu können. Das alles schon (Teilnehmer ?: Viel zu umständlich.) eigentlich viel zu umständlich. (Interviewer: mhm (bejahend)) Das ist das Problem. Also lieber, ich sag mal vier, fünf Griffe die vernünftig funktionieren. Auch wiederholbar. Ohne große Umschaltzeiten. Ohne irgendwelchen großen Firlefanz. (Interviewer: mhm (bejahend)) Käme ich besser mit zurück. Wie ne Hand die 3000 Griffe kann. Und ich find se grade nicht. #01:34:24-0#

Interviewer: Okay. Das heißt da sind wir bei dem Punkt, dass weniger mehr ist. (Teilnehmer Nr. 1: Ja.) Dass lieber wenig, was schnell und einfach funktioniert? #01:34:30-2#

Teilnehmerin 1: Robust, schnell, zuverlässig, wiederholbar. #01:34:32-9#

Interviewer: mhm (bejahend) #01:34:34-0#

Teilnehmerin 1: Also ich hab ne Hand getestet, ich möchte den Namen nicht sagen. Ich hab die Griffe die ich brauchte nie gefunden. Das war immer das größte Problem. (Interviewer: mhm (bejahend)) #01:34:40-8#

Interviewer: Ok. Das heißt Sie hatten die, aber eigentlich waren sie nicht verfügbar, weil's eben (..??) #01:34:41-7#

Teilnehmerin 1: Ich hab se getestet. Ich hätte se auch länger testen können. (Interviewer: mhm (bejahend)) Ich hab se nach n paar Tagen wieder abgegeben,

hab gesagt: „Hier. Bitte schön. Kein Interesse. Brauch ich nicht.“ (Interviewer: mhm (bejahend), ok.) #01:34:51-2#

Interviewer: Und bei Ihnen Herr (Name von Teilnehmer 2 aus Datenschutzgründen ausgelassen)? Haben Sie noch irgendwas wo Sie sagen, irgendein Verbesserungsvorschlag oder irgendwas, was Sie verändern würden? #01:35:00-1#

Teilnehmer 2: Spontan jetzt nicht, nee. Also Robustheit (Interviewer: mhm (bejahend)) das wär schon mal- #01:35:04-1#

Interviewer: mhm (bejahend), ja. Und Robustheit inwiefern? Von der Technik her? Von, vom Äußeren her? #01:35:10-5#

Teilnehmer 2: Mh vom Äußeren her ist, dass die nicht so halt schnell, nicht kaputtgeht. Wenn man was handwerkliches macht. (Interviewer: mhm (bejahend)) Oder so. Oder Kraft ausübt oder sowas (Interviewer: Ja.). Sind die da schon sehr empfindlich. Ich mein ich hab jetzt, arbeite nicht im Handwerk oder sowas. (..??) (Interviewer: mhm (bejahend)) Oder irgendwie sowas. Aber ähm da muss man schon manchmal n bisschen aufpassen. Dass man (Interviewer: Ja.) das Teil nicht unbedingt äh kaputt macht. (Interviewer: mhm (bejahend)) #01:35:29-4#

Teilnehmerin 1: Und ich glaub wir müssen n bisschen differenzieren auch (Interviewer: mhm (bejahend)) weil wir haben ja hier so ne gemischte Gruppe. Sag ich mal. Drei Leute haben ja also, letztendlich, nur die Hand nötig. (Interviewer: mhm (bejahend)) Und drei Leute n bisschen mehr. Wobei einer wie gesagt Schulter Ex ist. Zwei Oberarm. (Interviewer: mhm (bejahend)) Äh da fehlt, also ich würd mir wünschen zum Beispiel, mal n Ellenbogen. Da wär ich schon wieder ganz weit vorne. (lacht) Äh wenn ich seh er kann mit der Hand über Kopf arbeiten. Das ist für mich unmöglich. (Interviewer: mhm (bejahend)) Äh und für’n Schulter Exer ist es noch schlechter natürlich. (Interviewer: mhm (bejahend)) Gar keine Frage. #01:35:56-7#

Teilnehmer 6: Ähm ich hab (..??) #01:35:58-6#

Teilnehmerin 1: Äh von daher sinken letztendlich dann auch die Ansprüche. Weil man muss so’n bissche zurückschrauben. (Interviewer: mhm (bejahend)) Ist halt einfach so. Weil ich muss ja auch mit den gleichen Muskeln den Ellenbogen auch noch bedienen. (Interviewer: Ja.) Das heißt einfach, dass nicht so viele Ansprüche da sind, weil man- #01:36:13-3#

Teilnehmerin 1: Weil’s nicht funktioniert. (Interviewer: Weiß, dass es-. Ok. Ja.) Weil alles andere behindert einen mehr, als, als es tatsächlich nützt. (Interviewer: mhm (bejahend)) Denn, wie gesagt, elektrischer Ellenbogen ist toll. Äh ist mir zu anfällig. Ich nutz ihn kaum noch. (Interviewer: Ja.) All-, alle elektrische, auch der ist laufend kaputt. Aber er ist n bisschen robuster als der andere. (Interviewer: Ja.) #01:36:32-6#

Interviewer: Und Herr (Name von Teilnehmer 3 aus Datenschutzgründen ausgelassen) gibt’s bei Ihnen noch irgendwas, was Sie beisteuern wollen? #01:36:36-7#

Teilnehmerin 3: (..??) (alle lachen) #01:36:41-8#

Teilnehmerin 1: Nicht sowas sagen. Sowas gibt's. #01:36:43-4#

Teilnehmerin 3: Hä? Gibt's ne? #01:36:43-4#

Teilnehmerin 1: mhm (bejahend). Muss man glaub ich auch nicht haben. #01:36:46-6#

Teilnehmerin 3: Okay. #01:36:46-7#

Interviewer: Und warum? Gibt's ne Ursache dafür? #01:36:51-3#

Teilnehmerin 3: Ja. Es ist schon, die riecht voll. Es ist schon etwas (..?). #01:36:53-9#

Teilnehmerin 1: Die Verbindung ist das Problem, glaub ich. (Teilnehmer 3 (..??)) #01:36:54-9#

Interviewer: Ich würd gern einmal bei Herrn (Name von Teilnehmer 3 aus Datenschutzgründen ausgelassen)) bleiben. Dass einfach, dass das Gewicht das dranhängt? Oder ist das auch einfach das Gefühl im- #01:37:04-0#

Teilnehmerin 3: Das Gefühl auch. Es ist ja (..?). Ja. (Interviewer: Ja.) #01:37:06-5#

Interviewer: Das heißt am liebsten das die, dass der Schaft kürzer ist. Und man einfach nur das vorne was hat? #01:37:09-9#

Teilnehmerin 3: Leichter, kürzer. (Interviewer: Ja.) Ja. (Interviewer: Ok.) #01:37:09-8#

Interviewer: Gibt's noch was, was jemand hinzufügen möchte? Zu den Veränderungen, zu den Verbesserungsvorschlägen? #01:37:18-7#

(kurze Pause) #01:37:18-1#

Interviewer: Ok. #01:37:22-0#

Teilnehmerin 1: Ne andere Verbindung vielleicht noch. (Teilnehmer Nr. ?: Ja, ja) Der Befestigung. #01:37:24-6#

Interviewer: Von wo nach wo? #01:37:26-6#

Teilnehmerin 1: Von den, ne generell (Interviewer: mhm (bejahend)) verbesserte Verbindung der Befestigung am jeweiligen Stumpf. (Interviewer: mhm (bejahend)) Weil das ist nach wie vor das größte, die größte Schwachstelle finde ich. Äh weil man immer das Gefühl hat, man verliert grade, in den Sommermonaten, in der nächsten Sekunde velrier ich die Prothese. (Interviewer: mhm (bejahend)) Bei der nächsten äh Schaffensphase, ich trage irgendwas (Interviewer: mhm (bejahend)), verlier ich die Prothese. Äh und das Gewicht hängt halt komplett an den Weichteilen. (Interviewer: mhm (bejahend)) Ständig, den ganzen Tag. Das ist schon Belastung.

Auch für den, für den Rest. (Interviewer: Ja.) Und auch ne Kopfsache. (Interviewer: Ja.) Weil er das einmal gehabt hat, dass man in der Fußgängerzone die Prothese verliert, äh achtet's nächste Mal drauf. (Interviewer: mhm (bejahend)) (lachen) #01:38:11-4#

Interviewer: Okay, das heißt es geht einfach darum, dass die stabil stitzt. Und dass die nicht so sehr die Weichteile belastet? #01:38:14-5#

Teilnehmerin 1: Es gibt (Sprechen im Hintergrund) ja eigentlich nur ein, oder es gibt zwei Möglichkeiten. Entweder n, n Silikonliner. (Interviewer: mhm (bejahend)) Den ich auf den Stumpf ziehe und dann nicht mit diesem in die Prothese ziehe. (Interviewer: Ja.) Oder ich hab n Hartschalenschaft oder n Weichschalenschaft, wo ich mich so reinziehe. (Interviewer: mhm (bejahend)) Letztendlich ist es aber immer n Kontakt auf der Haut. Äh oder ne OP. Dass n, n Dübel da reingesetzt wird. (Interviewer: mhm (bejahend)) Das gibt es natürlich auch zwischenzeitlich. (Interviewer: Ja.) Äh dann hab ich sicherlich ne sichere Verbindung. (Interviewer: mhm (bejahend)) Kann das nicht verlieren. Hab aber dann das Infektionsrisiko. (Interviewer: Ja, klar.) weil ich ständig offene Stellen habe. (Interviewer: mhm (bejahend)) Äh da gibt's also nocht nichts. Und ich wüsste auch nicht, wie man's lösen könnte. (Interviewer: Ja.) Leider. (Interviewer: Ja.) #01:38:51-0#

Interviewer: Ich glaub Sie wollten gerade noch irgendwas einwerfen (an Teilnehmer 6 gewandt)? #01:38:53-9#

Teilnehmer 6: Ja. Das Problem ist ja auch die ganzen einzelnen (Verbindungen?). (Interviewer: mhm (bejahend)) Die man hat. Ich hab jetzt zum Beispiel ne Hand. Ich habe n Koffer gezogen. (Interviewer: mhm (bejahend)) Und dann (war auf einmal?) der Koffer stehengeblieben (lacht), die Hand war immer noch dran. (Interviewer: Ok. Ja.) Wie komm-, wie komm ich hier an, an die Hand wieder ran. Krieg ich jetzt so nicht wieder rangeschraubt, wenn die, wenn die am Koffer ist. #01:39:12-4#

Interviewer: Also einfach dass die komplett, (Teilnehmer 6: Die kann ich ja dann nicht mehr öffnen. Die ist ja geschlossen) ja. #01:39:16-2#

Teilnehmer 6: (..?). Ich habe keine Möglichkeit die zu öffnen. (Interviewer: mhm (bejahend)) Die muss ich (..?) Koffer auseinanderbauen. (Interviewer: mhm (bejahend)) Ich hab aber keine Hilfsmittel dabei. (Interviewer: mhm (bejahend)) Wie geht's dann weiter? #01:39:22-9#

Interviewer: Ja. Also dass die Komponenten innerhalb des Arms einfach gut zusammenhalten? #01:39:30-7#

Teilnehmer 6: Ja. #01:39:28-7#

Interviewer: Ja, ok. Ähm dann hätt ich als nächste Frage ähm und tatsächlich auch schon letzte Frage eine, die ein bisschen abstrakter ist. Und zwar haben wir jetzt vor allem über, einfach über Greiffunktionen, über Funktionen vom Halten und so weiter gesprochen. Was die Prothesen ja nicht können, ist irgendne Art von Gefühl. Oder von ner Rückmeldung weiterzugeben. Und ich würd gerne von Ihnen wissen, ob das was ist, was Sie sich vorstellen könnten, was was nützliches wäre. Dass man irgendne Art von Rückmeldung, Feedback bekommt. Über Sachen die man anfasst.

Zum Beispiel, weiß nicht, heiß, kalt, oder mit wie viel Kraft ich irgendwas-. Oder weiß nicht. Ob's da irgendn Punkt gibt wo Sie sagen, wenn das so'n Arm könnte, wenn ich sowas, wenn ich irgendne Art von Rückmeldung ähm über das was der Arm macht bekommen könnte, wäre das was hilfreiches? Und wenn ja, wo müsste das sein? #01:40:14-9#

Teilnehmer 6?: Also außer heiß, kalt bekomm ich immer ne Rückmeldung irgendwo. (Interviewer: mhm (bejahend)) Weil ich höre meinen Motor. Und ich greife. (Interviewer: Ja.) Und wie, hör ich auch wie fest ich greife. (Interviewer: mhm (bejahend)) Das hört man irgendwann. Das hat man im Ohr. An-, andere Seiten wo man da an, an der Prothese (hoch und runter?) geht. Mit, mit irgendwelchen (Interviewer: mhm (bejahend)) Fingern oder so. (Interviewer: mhm (bejahend)) Kribbelt's natürlich auf'm Stumpf. (Interviewer: mhm (bejahend)) Ist einfach so. (Interviewer: Ja.) Und das einzigste was fehlt ist heiß und kalt. #01:40:42-6#

Interviewer: Und ist das was, was fehlt? Was nötig wäre? #01:40:46-7#

Teilnehmer 6: Weiß ich nicht. Ich hab mich da dran gewöhnt, dass es nicht ist. (Interviewer: Ok, ja.) #01:40:54-7#

Interviewer: Aber das heißt eben, dass n Gefühl über die Kraft, wie also durch dieses Hören. Dass Sie darüber ne Information haben wie viel Kraft da drin ist. Und dass Sie auch (Teilnehmer 6: Ja mit wie viel Kraft ich zufasse.) Ja, mhm (bejahend). Genau. Ja. #01:41:03-5#

Teilnehmer 6: Weil ich, kann ja, kann ja zufassen. Und richtig zufassen. (Interviewer: mhm (bejahend)) Und hier, wenn der Motor schwergängiger wird, hör ich das. (Interviewer: Ok, ja.) Ich höre die, die, die Veränderung im Motor. (Interviewer: mhm (bejahend), ja.) Ja und dann weiß ich reicht's oder reicht es nicht. (Interviewer: mhm (bejahend)) #01:41:18-7#

Interviewer: Und was meinen die anderen dazu? #01:41:22-1#

Teilnehmer 2: Also ich bräuchte die Funktion heiß, kalt oder sowas nicht. Weil ich's einfach nicht kenne. (Interviewer: mhm (bejahend)) Weil ich äh ohne Hand geboren bin. Also ich vermiss (Interviewer: Ja.) da jetzt nichts und könnte mir jetzt auch nicht (.?) unbedingt Mehrwert durch vorstellen. (Interviewer: mhm (bejahend)) #01:41:35-9#

Teilnehmerin 1: Sie, für mich persönlich muss sie diese Funktion zum Beispiel nicht haben. (Interviewer: mhm (bejahend)) Ne Rückmeldung darüber. Weil es gibt da ja zahlreiche Studien und zahlreiche Versuche wo man (gewählt?) hat äh mehr oder minder gewöhnt man sich letztendlich als Prothesenträger irgendwelche Berührgen an. (Interviewer: mhm (bejahend)) Das heißt wenn ich hier drauf eine Berührung habe, hab ich ne Rückmeldung die Haut letztendlich. (Interviewer: Ja.) Äh und irgendwann, wenn man's lange genug macht, bildet man sich zumindestens ein, das Ding hat Gefühl. (lachen) #01:42:03-4#

Teilnehmer 4?: Ja. (lachen) #01:42:04-9#

Interviewer: Und wie ist es bei Ihnen mit der, eben mit der Kraft mit der Sie

zugreifen? #01:42:07-9#

Teilnehmerin 1: Auch die ist steuerbar. (Interviewer: mhm (bejahend)) Weil ich weiß als äh, oder merke an, ja. Eigentlich ist der, der Akkustikapparat, das heißt also der, der Motor, daran (Interviewer: mhm (bejahend)) hört man, wie feste man greift. Oder wie langsam oder wie schnell. (Interviewer: Ja.) #01:42:22-7#

Interviewer: Das heißt also das ist- #01:42:23-7#

Teilnehmerin 1: Letztendlich ist das also auch n Gewöhnungprozess. #01:42:24-4#

Interviewer: mhm (bejahend), ja. #01:42:24-4#

Teilnehmerin 1: Und äh da fehlt mir als nichts wie gesagt. Was sicherlich eher fehlt ist flüssige Bewegungen. Oder sonst irgendwas. (Interviewer: mhm (bejahend)) Weil es ist halt alles noch nicht so (Interviewer: mhm (bejahend)) auf dem Stand, wie man's gerne hätte. #01:42:35-5#

Interviewer: Ja. Und die anderen? #01:42:39-8#

Teilnehmer 5: Kann mich da nur anschließen. Wirklich. Was (er gesagt hat?). (Interviewer: mhm (bejahend)) Heiß und kalt ist jetzt nichts, was, was ich irgendwie in der Arbeit bräuchte. Und alles andere, darüber bekomm ich bereits unmittelbar über den Anschluss den unmittelbaren Hautkontakt (Interviewer: Ja.) gibt. Und die Hautberührung gibt ne (Interviewer: Ja.) Rückmeldung. (Interviewer: mhm (bejahend)) #01:42:54-2#

Interviewer: Ja. Wie ist das mit Oberflächenbeschaffenheiten? Wenn man irgendwas eben, irgendne Oberfläche berührt? #01:43:00-0#

Teilnehmerin 3?: Ja gut man sieht's. (..?) (lachen) #01:43:04-1#

Teilnehmer 5: Es ist, es gibt jetzt keine-. Also ich überleg grade (Interviewer: mhm (bejahend)) noch, (durcheinander sprechen) (..?). Wenn ich Türen öffne oder schließe (Interviewer: Ja.). Oder, oder n Gegenstand berühre wie ne Tasse. Oder äh irgendwas festhalte (Interviewer: mhm (bejahend)), was (..??) ich da habe. (Interviewer: Ja) #01:43:18-8#

Interviewer: Und Sie sagen, Herr (Name von Teilnehmer 3 aus Datenschutzgründen ausgelassen), Sie sehen das. Das heißt das reicht auch aus, dass man's sieht? #01:43:22-5#

Teilnehmerin 3: Ja. (..?) darauf kann ich mich jetzt schon darauf einstellen. (Interviewer: mhm (bejahend)) #01:43:26-2#

Teilnehmer 2?: (Ja und wenn man?) die Hautberührung hatte. Und man spürt (..?) mit dem gesunden Arm. (Interviewer: mhm (bejahend)) #01:43:33-0#

Teilnehmer 6: Oberflächen (..??) A (nehm ich's mir?) (..?) und B (Interviewer: Ja.) ich habe ja beide Hände am (..?) (Interviewer: Ja.) #01:43:38-6#

Teilnehmer ?: (..??) #01:43:41-2#

Interviewer: mhm (bejahend) #01:43:44-4#

Teilnehmer 5: Weiß nicht wie das, (Interviewer: Ja.)-. Nein, seh ich jetzt (..) (lacht) #01:43:46-6#

Interviewer: OK. Also nichts, was jetzt irgendwie in Ihren Augen nötig wäre? #01:43:54-6#

Teilnehmerin 1: Also da fehlt glaub ich, das fehlt mir am wenigsten (Interviewer: mhm (bejahend)). Ist meine persönliche Meinung. Ich glaube das teilt sich. Äh eiskalt ist uninteressant. (Interviewer: mhm (bejahend)) Weil tut ja auch nicht weh. (Interviewer: Ja.) (lachen) Kostet maximal n Handschuh. (lachen) #01:44:11-3#

Teilnehmer 6: Außerdem, außerdem (sind wir ja?), ne? Die besten Maßträger, ne? Wir können (gewinkelt?) am längste am längsten Maße halten. #01:44:19-4#

Teilnehmerin 1: Natürlich. (lachen) #01:44:19-3#

Teilnehmer 6: Und wir können da auch nochmal weggehen. Das kann keiner. (lachen) #01:44:25-7#

Interviewer: Ok. Ähm aber wenn Sie sagen, äh Herr (Name von Teilnehmer 1 aus Datenschutzgründen ausgelassen), dass, dass das eben nicht das Wichtigste ist. Wenn wir jetzt nochmal abschließend sagen, was ist denn das Wichtigste? Also wenn wir die Runde langsam beenden. Und sagen, dass sowas wie dieses Gefühl, dass das nciht das Wichtigste ist, was man jetzt erstmal verändern müsste. Was ist denn das Wichtigste? #01:44:42-9#

Teilnehmer 1: Weil wie gesagt, das Gefühl, Gefühl an sich würd mir, würd mir keinen Vorteil bringen letztendlich. (Interviewer: Ja.) Weil ich kann mir nicht vorstellen, (Interviewer: mhm (bejahend)) dass es einen Sensor gäbe, der tatsächlich das reale Gefühl einer gesunden Hand wiedespiegeln würde. Äh weil ich nicht wüsste, wie man’s übertragen möchte. (Interviewer: mhm (bejahend)) Weil die maximale Geschichte kann ich ja nun wieder nur kriegen über die Haut. (Interviewer: mhm (bejahend)) Äh das Wichtigste ist ne zuverlässige, robuste Prothese. Die zumindestens annährend so schnell arbeitet, wie eine normale Hand. Die sicher greift. (Interviewer: Ja.) Die nicht beim kleinsten Überlasten defekt ist. Äh und dann ewig lange in Reparatur ist, weil die Ersatzteile grad nciht lieferbar sind. Das ist glaube ich (Interviewer: Ja.), für mich persönlich wäre es das Wichtigste. (Interviewer: mhm (bejahend)) #01:45:23-3#

Teilnehmer 6: Und die Serivicezeiten sollten schon deutlich unter sechs Wochen liegen. #01:45:28-9#

Teilnehmerin 1: (Name von Jemandem aus Datenschutzgründen ausgelassen)?(lacht) #01:45:30-8#

Teilnehmer 6: Ich würde mal tippen so auf sechs Stunden. Dann würd das passen. #01:45:32-1#

Interviewer: Ok. Und was gibt’s sonst noch an so, wenn wir das einfach abschließen, an so der wichtigste Punkt, denn wir hier aus dem Gespräch mitnehmen sollen? Was einfach das Wichtigste wäre, das anders funktioniert. Oder was einfach das wichtigste an der Prothese is? #01:45:49-1#

Teilnehmer 4: Funktion und Befestigung. (Interviewer: mhm (bejahend)) Das ist das Wichtigste. (Interviewer: Ja.) Das die Funktion (..??) (Interviewer: mhm (bejahend)) und nicht, dass man Gefühl hat (..??). (Interviewer: Ja, ja, ok.) (..??) Nicht wie die eigene Hand. Das (..??) (Interviewer: Ja.) (..??) #01:46:14-2#

Teilnehmer 5?: Ja, ne absolute Verlässlichkeit. Und auch äh (Teilnehmer 4: Ja.) die Funktion. Ne? (Interviewer: mhm (bejahend)) Dass ich mich drauf einstellen kann. Und weiß ähm in gewissen Situationen, wo ich einfach auch nicht mehr drüber nachdenke, (Interviewer: mhm (bejahend), sonderns wo’s automatisch passiert, dass es dann (Interviewer: Ja.) wirklich funktioniert. Ich hab vorhin von, von der Situation mit’m Auto fahren gesprochen. Und das war einfach schon grenztwertig. (Interviewer: mhm (bejahend)) Also wenn ich da überlegt hätte, dass ich deshalb n Unfall gebaut hätte. Ähm das, ähm das (Interviewer: Klar)-. Deswegen würde ich sagen, dass das für mich das Wichtigste ist. (Interviewer: mhm (bejahend)) Dass ich mich zu 100 % drauf verlassen (Interviewer: mhm (bejahend)) kann, dass die Prothese auch wirklich die Signale die ich sende, (Interviewer: mhm (bejahend)) ob jetzt bewusst oder unbewusst, (Interviewer: Ja.) dass die auch umgesetzt werden. (Interviewer: Ja.) Eins zu eins. Ganz egal ob ich schwitze. Ob ich gerade aufgeregt bin oder nicht. Äh dass das wirklich funktioniert. (Interviewer: Ja.) Ja. Und wenn’s drei Funktionen sind die ich habe. Dann ist es halt so. Aber die müssen sitzen, ja? (Interviewer: mhm (bejahend)) Und wenn’s 20 Funktionen sind. Gut. Dann ist’s halt besser. Aber es muss funktionieren. (Interviewer: Ja.) #01:47:03-3#

Interviewer: Und Sie Herr (Name von Teilnehmer 2 aus Datenschutzgründen ausgelassen), haben Sie noch irgendwas? So als wichtigsten Punkt? #01:47:07-5#

Teilnehmer 2: Ich glaub wurde alles gesagt. (Interviewer: Ok.) Also, Schnelligkeit, Zuverlässigkeit, (Interviewer: mhm (bejahend)) und natürlich auch Optik. #01:47:13-9#

Interviewer: Ja. Herr (Name von Teilnehmer 3 aus Datenschutzgründen ausgelassen)? #01:47:14-6#

Teilnehmerin 3: Kann (mich?) auch nur anschließen. #01:47:16-7#

Interviewer: Okay. (lacht) Ok, dann ähm würd ich sagen dass wir soweit durch sind. Genau, wenn niemand mehr irgendwas noch zu ergänzen hat. Oder zu revidieren? Irgendwas? Ähm dann würde ich einfach sagen, dass wir hier ähm das Gespräch beenden. Die Mikrofone wieder ausmachen.
