# Supplementary material for: What do users and their aiding professionals want from future devices in upper limb prosthetics? A focus group study
Source: PLoS One. 2023 Dec 29;18(12):e0295516. doi: 10.1371/journal.pone.0295516 (PMC10756510; doi:10.1371/journal.pone.0295516)
Supplement: S1 Appendix — (ZIP) [file pone.0295516.s001.zip › FocusGroup_Transcripts/FGP3.pdf]

Interviewerin: Ähm und dann würd‘ ich Sie bitten, wenn ich gleich die erste Frage stell‘, dass wir die einmal der Reihe nach beantworten, also dass da erstmal jeder zu Wort kommt und, dass Sie sich ganz kurz mit Vor- und Nachnamen einmal vorstellen, damit wir die Stimme mit dem Namen später verknüpfen können. Genau, danach darf gerne in den weiteren Fragen frei geantwortet werden, aber dass wir da einmal der Reihe nach gehen. (kurze Pause) Ja ich würd‘ Sie bitten ähm zum Einstieg sich vorzustellen, Sie als Prothesenträger, wenn Sie auf einen anderen Menschen mit einer Armprothese treffen, auf was Sie da zuerst achten? #00:00:57-2#

Teilnehmer 9: Also mein Name ist (Name aus Datenschutzgründen ausgelassen), bin 50 Jahre und Prothesenträger. (Interviewerin: Ja.) Mh wo ich drauf achte sind die Hände. Komisch, aber wahr. #00:01:14-5#

Interviewerin: Mhm (bejahend). Und was an den Händen? Einfach das Aussehen, oder- #00:01:19-2#

Teilnehmer 9: Nein, ob die Person auch ‘ne Prothese trägt oder nicht. (Interviewerin: Ok.) We::il ich&mein Vater, der hat zwei B-&zwei Beine verloren und hat dann Beinprothesen und das fällt sofort äh ins Gewicht, also fällt sofort auf. (Interviewerin: Mhm (bejahend)) Und dann sieht man an den Leuten, ob Sie auch Prothesenträger sind oder nicht. #00:01:32-6#

Interviewerin: Ja. Aber wenn Sie feststellen, dass es ein Prothesenträger ist, gibt’s was ähm worauf Sie an der Prothese achten? #00:01:40-2#

Teilnehmer 9: Nein. #00:01:40-2#

Interviewerin: Ok, ja. Gut. #00:01:44-5#

Teilnehmer 5: Äh mein Name ist (Name aus Datenschutzgründen ausgelassen), ich bin 49 Jahre alt, (kurze Pause) seit ‘93 ich habe eine Prothese (Interviewerin: Mhm (bejahend)). (kurze Pause) #00:01:58-3#

Interviewerin: Und, wenn Sie einen anderen Menschen treffen, der auch eine Prothese hat (Teilnehmer 5: Prothese hat.), ist das irgendwas wichtig, wenn Sie das sehen? Gucken Sie auf irgendwas Besonderes? #00:02:05-4#

Teilnehmer 5: Nein, ich gucke nur diese Prothese. (Interviewerin: Mhm (bejahend)) Wenn ich möglich habe z-&zu mit ihm reden, dann ich ge-&ich ka-&ich rede sofort und dann ich frage, wenn ich darf und dann-. Weil ich will, welche Prothese trägt und dann wie funktioniert. Ä::h (kurze Pause) was kann mehr als meine Prothese machen (Interviewerin: Ja.), was kann ich noch erfahren, solche Sachen. #00:02:31-5#

Interviewerin: Ja, Danke schön. Herr (Name von Teilnehmer 3)? #00:02:31-1#

Teilnehmer 3: Also mein Name ist (Name aus Datenschutzgründen ausgelassen), ich bin 63 Jahre alt und hab durch einen Autounfall meinen&meine linke Hand verloren. Das heißt, eigentlich war&hatte ich erst nur ‘n Plexusabriss und hab die Hand dann freiwillig abschneiden lassen, weil sie (Interviewerin: Mhm (bejahend))

mich sehr gestört hat und trage seit 2008 äh eine Armprothese und wenn ich jemanden treffe, der auch so ‘n Ding trägt gucke ich mir glaub ich trotzdem zuerst dessen Gesicht an (Interviewerin: Mhm (bejahend)), weil das&und schau mir an ob das ‘n netter Mensch ist oder nicht und wenn’s was&wenn er, sagen wir’s mal so: ‘N interessantes Modell trägt, (Interviewerin: Ja.) dann frag ich ihn was hast du da und was macht das anders als das was ich hab (Interviewerin: Mhm (bejahend)) und fang an&versuch rauszubringen, ob da was zu lernen ist oder nicht. (Interviewerin: Ja.) Aber ich glaube letztlich ist mir das ziemlich egal ob der ‘ne Handprothese trägt, oder nicht. #00:03:12-5#

Interviewerin: Ja, das ist klar. Aber eben geht’s dann so ‘n bisschen darum, dass man einfach darauf achtet: Kann die Prothese mehr als meine kann, gibt’s da noch irgendwas was ich vielleicht nicht erf- #00:03:19-1#

Teilnehmer 3: Nee, ich glaube sogar ich find wichtiger sogar: Passt das zu dem? #00:03:22-7#

Interviewerin: Ja, ok. Mhm (bejahend) #00:03:23-2#

Teilnehmer 3: ‘Ne? Also es gibt ja Menschen, mit ganz unterschiedlichen äh die ganz unterschiedlich leben, ganz unterschiedliche Vorhaben in ihrem Leben auch haben. Der eine möchte schön aussehen, der andre möchte Holz hacken und das sind zwei Dinge, für die man völlig unterschiedliche Arme braucht und ich glaub, für mich w-&ist dann eher wichtig: Ist das stimmig bei diesem Menschen, passt das zu dem? (Interviewerin: Mhm (bejahend)) #00:03:39-6#

Interviewerin: Ja, ok. Danke schön. Frau (Name von Teilnehmerin 4 aus Datenschutzgründen ausgelassen)? #00:03:41-7#

Teilnehmerin 4: Ich heiße (Name aus Datenschutzgründen ausgelassen), bin 57 Jahre, hatte seit dem dritten Lebensjahr ‘ne Armprothese. Ist ein Contergan-Fall gewesen, dit wurde nicht anerkannt. Hab eigentlich die ganzen Sachen schon von vorne bis hinten durch (Interviewerin: Mhm (bejahend)). Was es von früher bis jetzt gibt an Technik. U::nd ja, wenn ich andere sehe, finde ich das normal (Interviewerin: Mhm (bejahend)). Weil i-&ich bin so aufgewachsen und ich bin auch auf ‘ne normale Schule gegangen. Ich kenn das nicht anders. #00:04:14-4#

Interviewerin: Ja. Und gibt’s trotzdem so ‘n bisschen sowas, dass man guckt eben, gibt’s noch irgendwas, was da für mich neu ist, was es vielleicht an neuer Technik gibt, die irgendwer hat, die ich nicht hab? #00:04:21-5#

Teilnehmerin 4: Mh:: das war jetzt vor kurzem. Ich hab seit einem Jahr die Prothese (Interviewerin: Mhm (bejahend)) wo ich immer wieder gefragt habe: „Ich möchte mal eine mit fünf Fingern haben, die sich bewegt.“ (Interviewerin: Mhm (bejahend), ja.) Ich hab‘ das gesehen im Fernseh (Teilnehmer 3: Mhm (bejahend)), das möcht ich auch gerne mal haben oder ausprobieren und seit einem Jahr hab ich die (Interviewerin: Mhm (bejahend)) und ja (kurze Pause) Vor- und Nachteile hat die Prothese. #00:04:43-2#

Interviewerin: Ja, ok. Darauf komm ich- #00:04:46-5#

Teilnehmer 3: Es gibt nichts Schlimmeres als einen erfüllten Wunsch (lachend). (Alle lachen.) #00:04:52-1#

Teilnehmerin 4: Ja, weil die erfüllt nicht alle meine Wünsche. (lachend) Hab‘ ich auch schon bemängelt (lachend). #00:04:56-5#

Interviewerin: Ok, dazu kommen wir gleich. Wir haben jetzt einmal auf die Prothesen von anderen Leuten geguckt. Darauf geguckt was ihnen da auffällt. Ähm ich würd‘ jetzt gerne zurück zu Ihnen kommen und einmal von Ihnen wissen, warum Sie sich ausgerechnet für Ihre Prothese entschieden haben. Also wie Sie gesagt haben, (wendet sich an Teilnehmerin 4) Sie hatten davor was anderes, wie kam’s dazu, dass&dass es jetzt gerade die Prothese geworden ist? #00:05:36-5#

Teilnehmerin 4: Ja::, ich bin damit aufgewachsen, ich habe also angefangen mit&mit ‘nem Greifer (Interviewerin: Mhm (bejahend)) mit zwei Haken (kurze Pause) nur. Dann hab‘ ich also, früher gab’s diese Holzhände, die man nicht bewegen konnte (Interviewerin: Mhm (bejahend)), dann konnte man die schon ‘n bisschen bewegen, dann kam so ‘n Lederhandschuh drüber. Also, ich hab die ganzen Stufen mitgemacht u::nd so wie Herr (Name von Teilnehmer 5 aus Datenschutzgründen ausgelassen), hab ich auch so eine gehabt (Interviewerin: Mhm (bejahend)) bis v-&die hab ich auch noch Zuhause. (Interviewerin: Mhm (bejahend)) Die wechsle ich ab und zu auch mal, weil mit der Prothese kann ich das nich‘ alles machen. #00:06:36-6#

Interviewerin: Ok, ja. #00:06:38-0#

Teilnehmerin 4: Also:: schwere Sachen heben, machen, auch Gartenarbeiten: Geht nicht. #00:06:42-1#

Interviewerin: Mhm (bejahend). Aber Sie haben gerade gesagt, dass Sie über’s Fernsehen erfahren haben, dass es sowas gibt (Teilnehmerin 4: Mhm (bejahend)) und was war dann da quasi was Sie bewegt hat, dass Sie gesagt haben: „Dann will ich sowas jetzt auch haben.“? #00:06:48-3#

Teilnehmerin 4: Ja ich möchte das haben, dass alle fünf Finger sich bewegen. #00:06:53-9#

Interviewerin: Ja, ok. #00:06:52-2#

Teilnehmerin 4: Ich möchte (kurze Pause) irgendwo wie ‘n normaler Mensch leben. (Interviewerin: Mhm (bejahend)) Dass die Finger sich bewegen, vielleicht auch drehen, aber das haben wir hier nicht hingekriegt, gibt’s jetzt auch, (kurze Pause) a::ber, dass es am wenigsten auffällt. (Interviewerin: Mhm (bejahend)) Ich bin immer so durch’s Leben gekommen, (kurze Pause) also find das gar nicht so schlecht, dass man eine Prothese trägt und viele sagen auch: „Oh!“. Die haben mich schon zig Mal gesehen und sagen: „Oh, ist mir noch nie aufgefallen!“. (Interviewerin: Mhm (bejahend)) #00:07:22-6#

Interviewerin: Ok, wie ist das bei den anderen? Wie kam’s da zu der Entscheidung, dass es <u>die</u> Prothese ist? #00:07:26-7#

Teilnehmer 9: Mein&äh meine Entscheidung lag darin, dass ich in 2010 einen Ab- und Ausriss hatte von der Hand, sie wurde in der Medizinischen Hochschule wieder replantiert (Interviewerin: Mhm (bejahend)) und es war dann, war das ‘ne Hand ohne Funktion. In 2015 hab‘ ich dann gesagt: „Ich kann nicht mehr. Ich möchte auch nicht mehr.“. Um äh ‘n bisschen Lebensqualität wieder zu erhalten brauchte ich dann natürlich was anderes als meine Hand, die nicht funktioniert. (Interviewerin: Mhm (bejahend)) Durch ganz viele Schmerzen, weil ich Linkshänder bin von (..??) und äh es ist dann so weit gekommen, dass ich in 2015 also gesagt habe: „Weg damit!“. Dann haben ‘se, meine Berufsgenossenschaft hat sich dann lange Zeit gelassen, dass ich erst im Anfang 2017 diese bekommen habe. (Interviewerin: Mhm (bejahend)) U::nd jetzt geht ich da&hab ich fürchterliche Schmerzen von der äh von den Nerven her (Interviewerin: Mhm (bejahend)). Mein Arm muss sich erstmal dran gewöhnen, hat man mir gesagt, aber jetzt trag ich die schon fast ‘n Jahr und es wurde nicht besser. Also so nach ca. ‘ner halben Stunde bis ‘ner Stunde muss dann die wieder ablegen. (Interviewerin: Mhm (bejahend)) U:nd das war&zu der Zeit war das erst vor der Quantum war ja ‘ne andere von der I-limb und hier, bei (Name eines Sanitätshauses aus Datenschutzgründen ausgelassen) hab ich äh die Information bekommen, dass es ‘ne Quantum gibt. (Interviewerin: Mhm (bejahend) Das war eben die Neueste, die auf dem Markt ist und dann hab‘ ich ‘se bekommen. (Interviewerin: Mhm (bejahend)). #00:08:45-7#

Interviewerin: Und haben Sie sich im Vorfeld selber informiert, was es gibt? #00:08:50-2#

Teilnehmer 9: Ich hab mich im Vorfeld informiert auf M-&auf Messen (Interviewerin: Mhm (bejahend)) in Düsseldorf und in Leipzig glaub ich war das, (Interviewerin: Mhm (bejahend)) u::nd äh meine Berufsgenossenschaft hat sich da schon quergestellt. „Warum informierst du dich? Ich g-&ich gebe dir das was ich möchte!“ (Interviewerin: Mhm (bejahend) u::nd dann hab ich gesagt: „Naja, dann gehen wir, mit dem Rechtsanwalt gehen wir leider vor.“, weil es gibt immer die Neueste vom technischen Zustand und das alle fünf Jahre, dass man da ‘ne Neue bekommen könnte. #00:09:23-7#

Interviewerin: Ja. Und war dann das Ausschlaggebende einfach, das war das neueste Modell, (Teilnehmer 9: Genau.) dann haben Sie sich am davon versprochen, oder gab’s auch was&was Sie wussten an Funktionen, an Aussehen, wo Sie gesagt haben, dass- #00:09:37-4#

Teilnehmer 9: Von Funktion her äh, ‘tschuldigung dass ich unterbrochen habe. #00:09:39-3#

Interviewerin: Alles gut. #00:09:38-8#

Teilnehmer 9: Von Funktion her wusste ich, dass die bis dato am meisten konnte. #00:09:45-5#

Interviewerin: Ok. #00:09:48-7#

Teilnehmer 9: Mit Gestiksteuerung, was viele andere eben noch nicht können, glaub ich (Teilnehmer 9: (..?)). Können ‘se? #00:09:56-0#

Teilnehmerin 4: Ja. #00:09:56-0#

Teilnehmer 9: Ach, ich weiß es nicht. Ich hab‘ keine Erfahrung damit, ich bin noch ‘n Neula-&Neuling (Teilnehmerin 4: Mhm (bejahend)) eigentlich auf diesem Gebiet. (Interviewerin: Mhm (bejahend)) #00:10:04-3#

Interviewerin: Ok. #00:10:04-3#

Teilnehmerin 4: Aber es funktioniert auch nicht immer. (lachend) #00:10:06-1#

Teilnehmer 9: Nee. Manchmal rutscht die mir so runter, dass die in ‘ne Ecke fliegt. (Interviewerin: Mhm (bejahend)) #00:10:15-0#

Teilnehmerin 4: Ja, darum geht’s nicht, von der Gestiksteuerung. Auch wenn man jetzt das macht (Teilnehmer 9: Mhm (bejahend)), macht ‘se ja nicht das, was ‘se soll (lachend). #00:10:22-5#

Teilnehmer 9: Das kenn ich auch. (Interviewerin: Mhm (bejahend)) #00:10:27-7#

Teilnehmerin 4: Verflucht man ‘se. (lachend) #00:10:28-6#

Interviewerin: Herr (Name von Teilnehmer 3 aus Datenschutzgründen ausgelassen), bei Ihnen? #00:10:27-6#

Teilnehmer 3: (kurze Pause) Wie war die Frage? #00:10:34-0#

Interviewerin: Ähm wieso Sie sich- #00:10:37-4#

Teilnehmer 3: Warum ich diese Hand hab? #00:10:37-4#

Interviewerin: Ja, genau. #00:10:37-4#

Teilnehmer 3: Ach so, ok. Also mh, ich hab‘ das ja vorhin schon gesagt, also mich interessiert eigentlich am meisten: Passt das zu dem, macht der&nützt es dem was? (Interviewerin: Ja.) Und ich hab‘ entsprechend ähm, ich hab ‘n ganzen Prothesenbaukasten, also ich hab Zuhause ‘ne I-limb-Hand in der Schublade liegen (Interviewerin: Mhm (bejahend)), die ist aussortiert worden, weil sie ähm der mechanischen Belastung meines Lebens einfach nicht standgehalten hat. Die hatten das Problem, dass&d-&die Motoren sind ja bei dieser Hand gleichzeitig die Fingerglieder (Interviewerin: Mhm (bejahend)) und dieser Motor dreht sich mit ‘ner relativ hohen Drehzahl und wenn der Motor sich verwindet, dann kriegt der, d-&dann wird die Lagerreibung in dem Motor höher, dann wird die Wicklung im Anker warm (Interviewerin: Mhm (bejahend)) und dann hat ‘se nach kurze Zeit ‘n Wicklungsschluss und mir sind bei dieser Hand in der Zeit wo ich sie hatte, das waren etwa fünf Jahre oder so, ich würde sagen sieben oder acht Mal die Finger stehengeblieben. (Interviewerin: Mhm (bejahend)) Also immer einzelne Finger, die dann ausgetauscht werden mussten und&und deshalb war das eigentlich-. Ich hatte mir davon versprochen, dass man&dass man damit also wirklich ‘ne gewisse Geschicklichkeit erreicht. Ich mach&meine Hobbies sind äh Segelfliegen, Segeln, ich mach Modellbau und solche Dinge und ich hatte gehofft, dass ich damit sozusagen mechanische Vorteile, hand-&hagesch-&handwerklich geschickliche Vorteile

(Interviewerin: Ja.) erreichen könnte. Das hat&hat sich aber n::ur zum Teil bewahrheitet, eben wegen der mechanischen äh Insuffizienz dieser Handprothese. Ich hab‘ dann als nächstes ‘ne, so ‘ne Bebionic bekommen und benutze für gröbere Arbeiten oder auch für Dinge wo es wirklich auf Geschicklichkeit und Kraft ankommt, also beim Basteln und so, noch ‘n (Name eines Prothesenherstellers aus Datenschutzgründen ausgelassen) Systemgreifer. Und das ist (kurze Pause) eigentlich, wenn man ganz ehrlich ist, die praktischste Prothese. (Interviewerin: Mhm (bejahend)). Weil die ist total simpel, sie hat mörderisch viel Kraft, sie hat ‘ne ganz hohe Geschicklichkeit in diesen Fingerspitzen, aber sie sieht so gnadenlos scheiße aus. (alle lachen) (Teilnehmerin 4: Stimmt.) Und (lachend), und deshalb&deshalb äh denk ich, also meiner Erfahrung nach ist es so, dass nicht eine Prothese (Interviewerin: Ja.) die Bedürfnisse eines Menschen befriedigen kann (Teilnehmerin 4: Mhm (bejahend)). Diese hier ist Klasse, weil sie aussieht wie ‘ne Hand (Interviewerin: Mhm (bejahend)), man kann damit (kurze Pause) Auto fahren, Fahrrad fahren, den Lenker festhalten, äh und sie ist mechanisch stabil genug, um dem normalen Alltag-. Und ich finde sie sieht auch cool aus, also ich denke eher, ich fühl mich nich‘ behindert. Also ich tu ‘ne Menge Dinge, die auch Leute die auch zwei Hände haben nicht tun können (Interviewerin: Mhm (bejahend)) und&und äh, deshalb&und ich denk auch irgendwie so&bei so ‘ner Hand: „If you can’t hide it, show it.“ Also wenn man das nicht verbergen kann, dann zeig’s der Welt (Interviewerin: Ja.). Und ich mach eigentlich auch die Erfahrung, dass äh die Umwelt darauf richtig toll reagiert. Also ich bin mal durch die Stadt gegangen mit ‘ner Einkaufstüte durch die Hand, da zupft so ‘n kleiner Junge an meiner Hand und sagt: „Woah, du hast ‘ne Hand wie Luke Skywalker! Darf ich das mal angucken?“ (alle lachen). Und&und äh ich glaube, dass man den Leuten durchaus zutrauen kann, mit sowas auch umzugehen (Interviewerin: Mhm (bejahend)). Und ich hab‘ damit eigentlich nur gute Erfahrungen gemacht (Interviewerin: Mhm (bejahend)). Also es gibt nicht (kurze Pause), ich kann die Frage: „Warum hast du jetzt genau diese?“, nicht beantworten, sondern&sondern ich glaube es ist so ein Mischmasch aus allem. Ich hab‘ auch ‘ne ganze Menge selbst gebasteltes Zeug. Ich hab‘ noch ‘n Arm, der hat überhaupt keine elek-&myoelektrische Funktionalität und da bastle ich allerlei Adapter dran für, keine Ahnung, wenn ich beim Segelflugzeug den&den&den Landeklappengriff betätigen will. Das geht&ist ‘ne rein mechanische Tat. Und Sie wollen nicht, dass da irgendwas Elektrisches ausfällt, also ist es besser das mechanisch zu lösen. (Interviewerin: Mhm (bejahend)). Oder ich hab‘ ‘n kleinen Doppeldecker, da betätige ich den Gasgriff mit ‘nem Adapter der auf den Gashebel geschoben wird und&und äh insofern, dass es ‘ne Prothese ist auf der einen Seite. Diese hier bringt mir psychosoziale Vorteile, weil die Leute sagen: „Ok, der sieht ja so halbwegs normal aus und irgendwie hat er was Witziges.“. (Interviewerin: Mhm (bejahend)). Und andere Dinge sind einfach schiere Mechanik. Und dann, ich benutz immer das, was der Situation am meisten angemessen ist. Ich hätt‘ natürlich was das irgendwie&am liebsten was, das irgendwie alles kann, dann muss ich nicht immer wechseln. #00:14:20-7#

Interviewerin: Ja. Aber das heißt, es geht eben hauptsächlich einfach darum, dass sie zu Ihnen passt, zu Ihrem Leben passt, die einfach den Aufgaben dann gerecht wird, die Sie&die Sie erfüllen, soll, als dass das Sie sagen, es soll in erster Linie so aussehen, dass das eben niemandem auffällt, dass ich dazugehör‘? #00:14:34-2#

Teilnehmer 3: Ich kann ‘n Beispiel erzählen. (Interviewerin: Mhm (bejahend)) Als ich den&als ich den ersten Arm bekommen hab, hab ich mein Segelflugzeug angehängt

(Interviewerin: Mhm (bejahend)) und bin damit hier, bei (Name eines Sanitätshauses aus Datenschutzgründen ausgelassen) auf den Hof gefahren und dann kam (Name einer Ärztin aus Datenschutzgründen ausgelassen) und (Name eines Orthopädietechnikers aus Datenschutzgründen ausgelassen) von&von (Name eines Sanitätshauses aus Datenschutzgründen ausgelassen)und die haben dann zusammen äh überlegt wie viel Arm müssen wir dranlassen, damit die Krafteinleitung in den Landeklappenhebel des Flugzeugs groß genug ist, wie viel Arm muss abgeschnitten werden, damit&damit wir ins Gesunde kommen und ‘ne ordentliche Heilung haben (Interviewerin: Ja.) und da ist dann ‘n Strich gemacht worden und am nächsten Tag, ich hab’s Flugzeug nach Hause gebracht, ‘ne, am nächsten Tag ist der Arm amputiert worden und na-&drei Monate später bin ich wieder geflogen. (Interviewerin: Mhm (bejahend)) Also es ist&ich&ich hab&verfolge da eher ‘n sehr praktischen Ansatz. #00:15:15-8#

Interviewerin: Ja, ok. Danke schön. #00:15:18-9#

Teilnehmer 3: Bitte. #00:15:18-9#

Interviewerin: Herr (Name von Teilnehmer 5 aus Datenschutzgründen ausgelassen), bei Ihnen, warum haben Sie sich für die Prothese entschieden, die Sie tragen? #00:15:25-6#

Teilnehmer 5: Diese Prothese? #00:15:26-9#

Interviewerin: Mhm (bejahend) #00:15:26-9#

Teilnehmer 5: Versicherung hat nur diese Prothese bezahlt, deswegen (lachend). (alle lachen) #00:15:35-4#

Interviewerin: Ok, das heißt, das heißt das war nicht Ihre- #00:15:37-8#

Teilnehmer 3: Ist ‘n guter Grund. #00:15:38-2#

Teilnehmer 9: Ist das so? #00:15:41-1#

Teilnehmer 5: Weil ich habe äh jetzt letzte Zeit (Interviewerin: Mhm (bejahend)) alle Neuigkeit-Prothese hab ich probiert, zwei Wochen. #00:15:47-8#

Interviewerin: Ja, ja. #00:15:47-8#

Teilnehmer 5: (Name eines Orthopädietechnikers aus Datenschutzgründen ausgelassen) hat das mir angebaut hier (Interviewerin: Mhm (bejahend)) und dann ich habe das alles äh probiert, diese letzte zwei (Interviewerin: Getestet?), getestet (Interviewerin: Ja.). Michelangelo und gibt’s noch eine neue Firma. #00:16:06-0#

Teilnehmerin 4: Ja, diese. (Zeigt auf ihre Prothese) #00:16:07-6#

Teilnehmer 5: Das, ‘ne? #00:16:11-7#

Teilnehmerin 4: Ja. #00:16:10-0#

Teilnehmer 5: Das I- #00:16:13-5#

Teilnehmer 3: Was ist das drunter? (Zeigt auf Prothese von Teilnehmerin 4) I-limb? Bebionic? #00:16:18-9#

Teilnehmer 5: Beispiel äh: Das hab‘ ich probiert, das wir haben nur fünf Funktion eingestellt. #00:16:25-6#

Teilnehmer 9: A:::h! #00:16:25-6#

Teilnehmerin 4: Ja, ja. #00:16:27-0#

Teilnehmer 5: Äh hier am Anfang, aber dann (kurze Pause) ich&ich benutze Prothese wie meine echte Hand. Sag ich das (Teilnehmerin 4: Ja.) (Interviewerin: Mhm (bejahend)). Wie meine echte Hand. Mit das, (kurze Pause) mach ich alles (zeigt auf seine Prothese): Ich koche, ich mache Tep-&äh Tapeten, ich mache Fließen, alles! (Interviewerin: Mhm (bejahend)) Ich mach alles! Du weißt das (richtet sich an seine Begleiterin, seine Ehefrau.). Aber fast, wie gesagt, manchmal die vergessen meine Bekannte oder meine Freunde, die sagen: „Ja, kannst du diese Tüte, kannst du das auch, kannst du das (alles?)-“. Ich sage: „Einen Moment! Ich habe eine Hand!“. Dann die sagen: „Entschuldigung.“. Mit Prothese: Fast alles mach‘ ich. (Interviewerin: Mhm (bejahend)) Fast alles. Ich brauche keine Hilfe, gar nichts. Egal, einkaufen, wirklich alles! Äh Laminat mach ich selber. Letztes Mal hab‘ ich mein Zuhause komplett auch gemacht. (Interviewerin: Mhm (bejahend)). Aber dann hab‘ ich die andere Prothesen probiert (kurze Pause) äh das hat bisschen anders Vorteil aber die&diese neue Prothese hat&die haben fünf Bewegung (Interviewerin: Ja.) eingestellt, aber dann äh hab ich immer das gefühlt. Hab‘ ich hier auch. Und dann, was sollte nicht machen, diese Hand, hat diese komische Sachen gemacht! (alle lachen laut) Und dann war ich hier: „Nein das ist unglaublich, das geht nicht“, hab‘ ich gesagt: „Bitte schön, hab ich alles gefilmt!“. (Interviewerin: Mhm (bejahend)). Die haben geguckt und dann war die Hersteller, von Hersteller dieser Mann auch hier, er hat gesagt: „Das ist unglaublich!“. Weil die Techniker hat sowas nicht eingestellt in diese Hand (alle lachen). „Wie können Sie diese Sachen machen?“, hab‘ ich gesagt: „Ich weiß das auch nicht.“. Extra das hab‘ ich alles gefilmt. (Teilnehmerin 4: Hm.) Und dann hab‘ ich Michelangelo probiert (Interviewerin: Ja.). Äh, das hat auch manche Vorteile, kann man bisschen mehr als diese Prothese machen. Weil das hat das und dann das dreht sich nur (Interviewerin: Mhm (bejahend)). Aber mit Michelangelo kann man so anfassen, kann man so anfassen (Interviewerin: Mhm (bejahend), ja.) (Teilnehmerin 4: Genau.), äh hat bisschen mehr Vorteile. (Interviewerin: Ja.) Und dann äh man muss die rechte Arm nicht so viel belästen (Interviewerin: Belasten?). Weil ich habe hier Probleme jetzt meine Schulter auch. (Interviewerin: Mhm (bejahend)) Aber das hat kein, Krankenkasse zahlt das nicht. Wir kämpfen seit wie viel Monate? (wendet sich an seine Ehefrau) Äh die wollen das nicht zahlen. (Teilnehmerin 4: Mhm (bejahend)) Jetzt ich habe rechte Arm auch Prothese, äh Probleme. #00:19:00-2#

Interviewerin: Das heißt Sie hätten gerne ‘ne andere, aber die Versicherung-. #00:19:04-7#

Teilnehmer 5: Ja, ich hätte gerne andere, weil mit die andere, ich kann noch mehr Sachen machen. (Interviewerin: Mhm (bejahend)) Manche Sachen ich kann so-,

weiß ich nicht. Mit das, wenn halte das so fest und dann ich muss immer ausschalten, sonst das geht allein auf. (Interviewerin: Ja.) Aber mit Michelangelo (kurze Pause) kann man mehr Sachen machen. Äh aber jetzt ich muss das r-. #00:19:25-2#

Teilnehmer 3: Darf ich Sie was fragen? #00:19:25-2#

Teilnehmer 5: Bitte. #00:19:27-7#

Teilnehmer 3: Wo ist die Amputation? #00:19:27-2#

Teilnehmer 5: Amputat-. #00:19:27-7#

Teilnehmer 3: Sie haben ‘n Ellbogen auch, ‘ne? #00:19:28-5#

Teilnehmer 5: Hier, bis hier. (Zeigt die Stelle an seinem Arm.) #00:19:28-0#

Teilnehmer 3: Ja, ja. Ok. #00:19:31-1#

Teilnehmer 5: Ich habe auch Autounfall gehabt damals und dann (Teilnehmer 3: Ja.) die haben falsch operiert, wurde falsch operiert. (Teilnehmer 3: Ja.) Dann (..?) reingekommen, die wollten von hier Amputation machen, aber dann die Ärztin hat gesagt, wenn die amputieren von hier, dann ich kann nicht ein richtige Prothese tragen, deswegen die haben noch bisschen höher (Interviewerin: Mhm (bejahend)) amputiert. #00:19:47-9#

Interviewerin: Sie haben jetzt erzählt was die Prothese gut <u>kann</u>. Ich würde dabei gerne einmal bleiben. Können wir mal sammeln, was für positive Dinge, also ich weiß, es gibt viele negative Dinge an den Prothesen, aber was sind die Positiven? Was sehen Sie als Vorteil von&von Ihrer Prothese, die Sie tragen? #00:20:04-6#

Teilnehmer 9: Mehr Lebensqualität zu bekommen. #00:20:08-7#

Interviewerin: Wodurch? #00:20:08-7#

Teilnehmer 9: Durch die Prothese, dass man da auch äh mit der Hand wieder was machen kann. #00:20:12-0#

Interviewerin: Mhm (bejahend) #00:20:13-5#

Teilnehmer 9: Äh, ich hab‘ schon versucht zu schreiben, aber das funktioniert noch nicht, weil das geht ja über den Arm. (kurze Pause) U::nd dann krieg ich schon&hab ich schon Probleme mit&mit beiden Armen jetzt. (Interviewerin: Mhm (bejahend)) (Teilnehmerin 4: Mhm (bejahend)). Dass ich da leider aussetzen muss, bei sowas #00:20:23-5#

Interviewerin: Und bei den anderen? Was für Vorteile? #00:20:27-5#

Teilnehmer 3: Die Vorteile sind, also egal was. Wenn Sie zum Beispiel zwei Drähte zusammenlöten wollen. (Interviewerin: Ja.) Das ist eigentlich augenfällig, ob Sie eine Hand oder zwei Hände haben, macht ‘n himmelweiten Unterschied (demonstriert,

was die Hände beim Löten tun). Ich hab‘, bevor ich ‘ne Hand hatte, um den zweiten Draht festzuhalten, hatte ich so kleine Stative mit ‘ner Krokodilklemme drin, hab dann die Dinger genau ausgerichtet, Sie kommen mit dem Lötkolben dran: Oh! Es äh hat sich bewegt und die beiden Drähte berühren sich nicht mehr. Sie können nicht löten, Sie fangen wieder an das auszurichten. (Interviewerin: Mhm (bejahend)) Dauert ewig. Da sind Sie, obwohl so ‘ne Hand nicht im Ansatz das kann, was ‘ne natürliche Hand zustande bringt, trotzdem weit vorne. #00:21:01-1#

Interviewerin: Ja. #00:21:01-6#

Teilnehmer 3: Und (kurze Pause) ich glaube es sind vor allem die praktischen Dinge. Und manchmal, wenn Sie ‘n Anzug anziehen, sieht’s einfach besser aus, wenn was aus’m Ärmel schaut. (Teilnehmerin 4: (lacht)) Also das spielt auch ‘ne Rolle, so dieser&der&der soziale Aspekt. Leute können besser damit umgehen. W-&wenn Sie mit einem leeren Ärmel, also ich würd‘ nicht gern mit ‘nem leeren Ärmel rumlaufen, weil (kurze Pause) da hab ich so Bilder wie Wolfgang Borcherts „Draußen vor der Tür“ oder sowas im Kopf, ‘ne? Das ist so dieser&dieser Kriegsversehrte und es gab ja Zeiten wo&wo behindert sein auch gleichzeitig irgendwie, ja wie soll ich das sagen, so&mit so ‘nem sozialen Stigma verbunden ist, ‘ne? (Interviewerin: Mhm (bejahend)) Also jemand der behindert ist äh, so will ich nicht auftreten. Also, insofern finde ich es schon wichtig, dass auch was auf‘m&aus‘m Ärmel schaut aber&aber ich glaube der wesentlichere Grund ist, es&es tut was für mich. (Interviewerin: Mhm (bejahend)). Tja. Egal bei was eigentlich, also wenn Sie ‘nen Laib Brot schneiden wollen oder so und können den nicht festhalten, dann äh, Sie können natürlich zum Bäcker gehen und sagen: „Schneid mir das mal!“. Sie können bei fast allem irgendjemanden fragen: „Mach mir das mal!“ und&aber es ist auch schick, wenn man‘s selber, weil manchmal ist niemand da, ‘ne? #00:22:47-2#

Interviewerin: Aber ist es dann besonders wichtig, diese Funktionalität bei&in der Freizeit, im&im Sozialen, in der Arbeit, wo&wo macht sich das besonders bemerkbar, dass es eben gut ist, niemanden fragen zu müssen, sondern da diese Selbstständigkeit oder diesen&diesen sozialen Effekt den Sie sagen, wo hat&also können Sie das unterteilen, dass es irgendwo ‘ne besondere Relevanz hat? #00:23:04-3#

Teilnehmer 3: Naja, in allen Lebensbereichen. #00:23:08-0#

Interviewerin: Ja. Mhm (bejahend) #00:23:08-0#

Teilnehmer 3: Wie woll-&wie soll man das trennen? Also das ist- #00:23:09-9#

Interviewerin: Weiß nicht, manche sagen ja, dass&dass zum Beispiel das Wichtigste war, dass man wieder zur Arbeit gehen kann, dass das den höchsten Stellenwert hatte. Deshalb frag ich, ob‘s verschiedene Stellenw- #00:23:16-0#

Teilnehmer 3: Na, bei mir war das&bei mir war das ja so, ich hab den Arm ja nicht wirklich verloren, ich hab&das ist auch in ‘nem ziemlich jungen Alter passiert, ich war 17 und da hat jemand in ‘nem Au-&mich in ‘nem Auto gegen Baum gefahren, also ich hab’s nicht selbst gemacht und seitdem (kurze Pause) das war erstmal ‘ne&war erstmal äh auch so ‘ne Lebenskatastrophe. Ich wollte Pilot werden bei der Lufthansa und war irgendwie zwei Jahre vor dem Abitur (Interviewerin: Mhm (bejahend)) und

dann waren diese Pläne auf einmal hinüber. Und ich hab‘ dann die 15 darauffolgenden Jahre äh im Wesentlichen damit verbracht ‘nen Beruf zu kriegen mit dem man Geld verdient und B, ‘ne Pilotenlizenz zu kriegen, damit ich fliegen konnte. (Interviewerin: Mhm (bejahend)) Und äh so im&im äh als Nebeneffekt ist dann rausgekommen, dass ich so mein Leben auch ganz erfolgreich hinkriegt hab (Interviewerin: Mhm (bejahend)) und&und das mich, dass von&heute seh‘ ich die Schwierigkeiten die ich da hatte eigentlich eher als Gewinn. Weil Sie lernen daraus (Interviewerin: Mhm (bejahend)) damit auch irgendwie mit Problemen umzugehen und&und daraus zieht man auch ‘ne Menge Selbstbewusstsein letztlich. #00:24:09-2#

Interviewerin: Ja, ja. Und bei Ihnen Frau (Name der Teilnehmerin 4 aus Datenschutzgründen ausgelassen), wenn wir auf die positiven Dinge gucken? #00:24:11-1#

Teilnehmerin 4: Ich kann gar nicht ohne. #00:24:14-7#

Interviewerin: Ja. #00:24:15-0#

Teilnehmerin 4: We::il ich kenn das von klein auf mit ‘ner Prothese. Es war immer unterschiedlich gewesen, die Macharten, aber (kurze Pause) wenn ich die nicht habe, wenn sie mal kaputt ist, bin ich aufgeschmissen. (Interviewerin: Mhm (bejahend)) Weil das ist für mich ‘ne normale Hand. #00:24:30-3#

Interviewerin: Ja. #00:24:30-7#

Teilnehmerin 4: Und so leb ich. Ich hab‘ äh gelernt damit, bin technische Zeichnerin im Maschinenbau und da hab ich genau gearbeitet wie alle anderen auch. Also für mich ist mein Leben normal gelaufen wie alle anderen auch (Interviewerin: Mhm (bejahend)), als ob ich ‘n normaler Mensch gewesen bin. Hab noch Geschwister und (kurze Pause) ich kenn das nicht anders. #00:24:53-8#

Interviewerin: Und würden Sie aber das sagen, also dass das, dass Leben normal gelaufen ist, dass Sie das der Prothese verdanken, dass das Leben- #00:24:58-2#

Teilnehmerin 4: Ja. #00:24:58-2#

Interviewerin: Ja, ok. #00:24:59-5#

Teilnehmerin 4: Das würd‘ ich schon sagen. Weil ohne Prothese bin ich aufgeschmissen. (Interviewerin: Ja.) Dann könnte ich nicht sehr viel machen. Also wenn die mal wirklich kaputt ist, dann wird’s schon eng. (Interviewerin: Ja.) #00:25:11-1#

Interviewerin: Und bei Ihnen, Herr (Name von Teilnehmer 5 aus Datenschutzgründen ausgelassen)? Sie haben vorher schon gesagt, dass Sie dank der Prothese <u>alles</u> machen können? #00:25:15-2#

Teilnehmer 5: Ja, das ist mein Lebenqualität. #00:25:17-1#

Interviewerin: Ja. #00:25:17-1#

Teilnehmer 5: Beispiel: Mein Tochter. Sie ist jetzt 13. (Interviewerin: Mhm (bejahend)) Äh, sie war zwei Jahre alt, meine Frau hat angefangen zum Arbeit wieder. Und dann Beispiel, wenn sie duscht (Interviewerin: Mhm (bejahend)), dann ich kann mit das Beispiel Föhn behalten und dann Haare. Das kleine Beispiel. (Interviewerin: Mhm (bejahend)) Aber <u>fast</u> seit ich Prothese habe, äh ok ich war Gewichtheber damals, (Interviewerin: Mhm (bejahend)) in der Türkei, ich war ein Sportler und dann mit Lizenz so. Und ich habe auch viele Medaillen damals. Jetzt solche Sachen nicht. Aber ich kann in meinem Leben, ohne Hilfe brauchen (Interviewerin: Ja.), alles machen. <u>Alles machen</u>. O::hne Hilfe brauchen. Ich kann alles machen. (Interviewerin: Mhm (bejahend)). Ich kann Fahrrad reparieren, ich kann alles machen. Aber ohne Prothese, natürlich kann ich das nicht. #00:26:05-8#

Interviewerin: Ja. Ok. Ähm ja, ich weiß, dass es außer den Vorteilen auch Nachteile gibt, die Sie ja g-&vorher schon erwähnt haben. Was sind denn so die gravierendsten Nachteile, wo Sie sagen, das ist schlecht an der Prothese, das funktioniert nicht so, wie ich das&wie ich das gerne hätte? #00:26:29-2#

Teilnehmer 9: Ich versuche mal anzufangen (Interviewerin: Mhm (bejahend)) und zwar, ist es das Gewicht, was mich sehr stört. (Interviewerin: Mhm (bejahend)) Wo ich auch äh Probleme mit den Armen und der Muskulatur habe. U::nd, was die Herrschaften hier schon gesagt haben, dass die Prothese das macht, was ‘se nicht soll. (Interviewerin: Mhm (bejahend)) (alle lachen) Das ist, manchmal geht ‘n Finger hoch und runter, obwohl er wirklich gar nichts dazu kann. Hört sich komisch an, aber trotzdem, ja, Die haben ihr Eigenleben, glaub ich. (Interviewerin: Mhm (bejahend)) Ja, das ist das, was ich dazu zu sagen habe. #00:27:21-6#

Interviewerin: Das heißt, das Gewicht einmal, einfach vom Gefühl her. Aber dann auch einfach die Funktion? #00:27:26-5#

Teilnehmer 9: Ja. #00:27:26-5#

Interviewerin: Mhm (bejahend). Und gibt’s Situationen, in denen das besonders eben nicht funktioniert, oder tut was&was es nicht soll? Gibt’s da b-&besondere Situationen, wo Ihnen das besonders auffällt, wo das vielleicht besonders stört? #00:27:43-4#

Teilnehmer 9: Es stört mich dauerhaft, muss ich sagen. #00:27:48-1#

Interviewerin: Mhm (bejahend), ja. #00:27:48-1#

Teilnehmer 9: U::nd, äh bestimmte Situationen kann ich nicht benennen im Moment. #00:27:53-7#

Interviewerin: Ok, ja. Bei den anderen? Was fällt den anderen dazu ein? #00:28:00-2#

Teilnehmer 5: Beispiel, das myoelektrische Prothese (Interviewerin: Mhm (bejahend)) am Anfang vor viele Jahre, ich habe die Erste gehabt. Weil am Anfang war die andre Prothese, dann (Name eines Orthopädietechnikers aus Datenschutzgründen ausgelassen) hat gesagt, gibt’s ein myoelektrische Prothese

und dann&das hätte hier ein Sensor (Interviewerin: Mhm (bejahend)). Dann hab‘ ich die erste Prot-&das gekriegt, erste Mal. Und dann Beispiel ich fahre mit Straßenbahn, was macht man? Ich habe eine Tüte und dann man festhaltet (Interviewerin: Mhm (bejahend)). Das geht nicht wieder auf (zeigt auf seine Prothese). Weil diese Stromstörung hat das kaputt gemacht, oder das war fest. Und dann bis zum Endstation fahr ich (Teilnehmer 3 (lacht laut)). Wirklich! Ist das so! Dann kommt der Fahrer: „Ja, hier ist nicht ein Hotel!“, sagt er zu mir. Ich sage: „Ja, ich weiß das. Aber ich kann nicht aussteigen, weil ich habe ein Prothese und das ist hängengeblieben. Was soll ich jetzt machen?“. „Ja, scheiße, was sollen wir jetzt machen?“, sagt er. Und dann hab‘ ich gesagt: „Ja, weiß nicht. Müssen wir Feuerwehr anrufen, das und dies.“ #00:29:10-0#

Teilnehmer 3: Das Rohr durchsägen. #00:29:11-1#

Teilnehmer 5: Wirklich, das war Katastrophe! (Interviewerin: Ja.) Und dann hab‘ ich gesagt: „Ok, jetzt nur ich will Sie als Zeuge.“, hab ich gesagt. Äh dann jetzt mach ich kaputt, (Interviewerin: Mhm (bejahend)) die hier gibt’s die Sachen. Hab‘ ich das so gemacht (demonstriert, wie er seine Prothese manuell geöffnet hat) und dann ist alle kaputt gegangen. Dann bin&damals war diese Handies nicht, dann könnte man Foto machen, oder das&etwas machen. Dann bin ich hierhergekommen, dann sag ich: „Ja so und so hat passiert und dann ich habe so gemacht.“. (Name eines Orthopädietechnikers aus Datenschutzgründen ausgelassen) sagt: „Mh hätte man das..“, das und dies, ich sage: „Ja, (Name eines Orthopädietechnikers aus Datenschutzgründen ausgelassen), ist das so!“ und dann er schreibt dahin. Was sagt (Name eines Prothesenherstellers aus Datenschutzgründen ausgelassen)? „Nein.“, äh weil ich gesagt habe, ich habe das so gemacht. Sonst, das hat Garantie sowieso. Ich müsste paar tausend Euro Geld zahlen. Hab‘ ich gesagt: „Nein, ich zahle das nicht.“. Dann ich fahre da hin, sollen die gleiche Hand geben da, jemand soll mit mir (Interviewerin: Mhm (bejahend)) mit Z-&mit Bus oder mit äh Straßenbahn fahren (Interviewerin: Ja.). (Name von Prothesenhersteller aus Datenschutzgründen ausgelassen) sagt: „Nein, das ist unmöglich.“. Dann hab‘ ich extra von hier, nach (Name des Orts aus Datenschutzgründen ausgelassen) gefahren (Interviewerin: Mhm (bejahend)) und dann jemand ist mit mir gekommen, dann wir haben das festgestellt, das hat weiß nicht was. Dann die haben paar Mal immer das was geändert, dann jetzt ist ok. Jetzt stirbt das nicht mehr. #00:30:19-5#

Interviewerin: Und jetzt können Sie auch in der Bahn- #00:30:22-1#

Teilnehmer 5: In der Bahn kann ich das alles machen, nur äh wenn äh Fernseher oder Computer wenn steht da, (Interviewerin: Ja.) wenn ich so, ich gehe durch (Begleitung Teilnehmer 5: Vorbei.), vorbei, das macht auf, (Interviewerin: Ok.) selber. Und dann, wenn ich im Hand was habe, dann fällt das runter. (Interviewerin: Mhm (bejahend)) Deswegen jetzt, wenn ich nehme ein Beispiel Wasserglas, (Interviewerin: Mhm (bejahend)) dann schalt ich das aus (Interviewerin: Ja.), dann egal wohin ich laufe, das passiert nichts. Aber immer noch das ist nicht im Griff. #00:30:48-2#

Interviewerin: Ok, ja. Das heißt es gibt irgendwelche, weiß nicht, Wechselströme- #00:30:51-2#

Teilnehmer 5: Das fehlt noch was. Das fehlt noch was. Weil wie ich gesagt habt, die

andre Hand, ich habe solche Sache gemacht, die haben schockiert hier. „Wie können Sie das machen?“ (Interviewerin: Mhm (bejahend)). Jetzt Beispiel Michelangelo, die haben ganz neue Hand gemacht, das kostet jetzt ein Prothese komplett 69 000 Euro. (Interviewerin: Mhm (bejahend)) (Teilnehmerin 4: Mhm (bejahend)) Und wirklich, hier in Deutschland, das ist 69 000 Euro. Die schicken diese Teile nach Türkei (kurze Pause), in der Türkei ist das 30 000 Euro. (Interviewerin: Mhm (bejahend)) Kleine Beispiel. Das ist hier, alles Katastrophe. Die gleiche Hand (Interviewerin: Ja.), hier 69 000 Euro, aber in der Türkei die gleiche Hand von (Name eines Prothesenherstellers aus Datenschutzgründen ausgelassen) die Teile 30 000 Euro. #00:31:30-1#

Teilnehmer 3: Ja, (Name eines Prothesenherstellers aus Datenschutzgründen ausgelassen) schöpft halt ab, was der Markt hergibt. #00:31:31-4#

Teilnehmer 5: Ja. Ja, wirklich. #00:31:33-7#

Teilnehmer 3: Ist so. #00:31:33-7#

Teilnehmer 5: Ja und zweite Sache: Die haben jetzt Michelangelo gemacht, hab ich mit (Name eines Orthopädietechnikers aus Datenschutzgründen ausgelassen) das auch gesprochen (Interviewerin: Mhm (bejahend)). Das hat ein (kurze Pause) äh Minus: Was ist das? Man kann viele Sachen mehr als das machen, (Interviewerin: Mhm (bejahend)) aber: Die haben kein Schalter. (…???) Michelangelo, das äh funktioniert immer. Man kann nicht ausschalten (Interviewerin: Mhm (bejahend)). Das hab‘ ich auch gesagt (Interviewerin: Ja.), hab ich gesagt. So teure Hä-&Hand, Beispiel ich will was richtig festhalten und dann ausschalten (Interviewerin: Ja.). Geht das nicht! Hab‘ ich das 15 Tage probiert. (Teilnehmerin 4: Der Akku dann auch schnell leer.) Ja, richtig. Hab‘ ich 15 Tage das auch probiert, zwei Wochen. Hat das- #00:32:14-4#

Interviewerin: Und jetzt wenn&kann ich in die Runde fragen mit diesen, weiß nicht was das ist, Wechselstörungen, dass man eben irgendwo rankommt und das dann aufhört zu funktionieren, kennen das andere auch? Haben Sie sowas auch? #00:32:25-8#

Teilnehmer 3: Hab‘ ich so nicht beobachtet. Wobei, was der Herr da sagt, die Hand macht Dinge, die man nicht erwartet (Interviewerin: Mhm (bejahend)) oder so, das Problem hat glaub ich jeder. Ich hab’s für mich dadurch gelöst, ich hab‘ dem&hab äh (Name eines Prothesenherstellers aus Datenschutzgründen ausgelassen) war ja damals noch ‘ne selbständige Firma, jetzt gehört sie ja (Name eines Prothesenherstellers aus Datenschutzgründen ausgelassen), ich hab denen einfach die Software dazu abgeschwatzt und Sie können ja die Schwellenwerte, w-&wo&wo das Ding auslöst können Sie ja selber feststellen. (Interviewerin: Mhm (bejahend)) Und ich hab‘ das Ding auf ’n Laptop installiert und so lange gefummelt, bis es für mich gepasst hat (Interviewerin: Mhm (bejahend)). Und jetzt geht sie sehr schwer auf und leicht zu. Was dazu&weil ich immer das Problem hatte, ich hab‘ Sachen hingeschmissen. Sie tragen ‘ne Tasche oder so und gucken nicht mehr hin, machen irgend ‘ne Beweg-&Bewegung, ich hab, bei mir ist das ja durch ‘n Plexusabriss gekommen. (Interviewerin: Mhm (bejahend)) Und ich betreibe die Hand eigentlich mit der Oberarmmuskulatur und nicht unten. (Interviewerin: Ja.) Und hab kein aktiv bewegliches Ellenbogengelenk, das ist wegen der Fliegerei so geblieben.

(Interviewerin: Mhm (bejahend)) Das ist mal so zertifiziert worden und ich wollt das nicht abschneiden lassen, weil dann hätt‘ ich wieder ganz von vorne mit den Behörden anfangen müssen. (Interviewerin: Mhm (bejahend)) Und äh was ich aber merke ist äh, dass die-&die Stromaufnahme dieser Hände, die ist einfach viel zu groß um durch ’n ganzen Tag wirklich zu kommen. Also normalerweise, wenn ich damit den ganzen Tag rumlaufe äh, muss ich etwas nach ‘nem halben Tag, braucht’s ‘n neuen Akku. (Interviewerin: Mhm (bejahend)) Und äh, ich bin mal von Holland mit ’m Auto hergefahren und hab schön auch mit links gelenkt und irgendwann, als ich dann abends aussteigen wollte, (Interviewerin: Mhm (bejahend)) ist mir dasselbe passiert, wie Herrn (Name von Teilnehmer 5 aus Datenschutzgründen ausgelassen) (Teilnehmerin 4 lacht.), nur hing meine Hand am Lenkrad fest (lachend). Die hing fest und ging nicht mehr auf. Ich hab‘ das Problem dann so gelöst: Ich hab den Arm einfach abgemacht, hab den Arm einfach im Gelenk gedreht, dann geht die ja los (Interviewerin: Mhm (bejahend)). Dann stand das Auto nachts da und am Lenkrad hing ‘ne linke Hand. Am nächsten Morgen klingeln die Nachbarn: „Sie haben Ihre Hand im Auto vergessen!“. Nein, ich konnt‘ sie nicht aufmachen (lachend). (Teilnehmerin 4 lacht) #00:34:21-7#

Interviewerin: Das heißt die Akkulaufzeit ist- #00:34:25-4#

Teilnehmer 3: Die Akkulaufzeit, also bei dieser Hand zumindest äh, ich glaube sie hat ‘ne deutlich höhere Stromaufnahme, als ‘ne I-limb-Hand und (Interviewerin: Mhm (bejahend)) dafür ist sie stärker, ist ja ‘n Vorteil, aber es ist gleichzeitig auch ein Nachteil, weil die Stromaufnahme deutlich höher ist und nach ‘nem guten halben Tag ist der Akku platt (Interviewerin: Mhm (bejahend)). #00:34:36-9#

Interviewerin: Wie behilft man sich dann? Gibt’s Extraakkus? #00:34:42-4#

Teilnehmerin 4: Nee. #00:34:41-3#

Teilnehmer 3: Hier nicht. Ich hab‘ mit (Name eines Orthopädietechnikers aus Datenschutzgründen ausgelassen) gesprochen und hab gesagt: „Den nächsten Arm machst du bitte mit ‘ner Klappe!“, dass ich den Arm, also d-&das&haben wir eigentlich auf dem Plan, dass wir&dass wir den Arm aufsägen und ‘ne Klappe reinmachen, so dass man den Akku-. Das ist ja ‘n ganz normaler LiPo, wie’s ihn auch im Modellbau gibt. Dass man den einfach wechseln kann. (Interviewerin: Ja.) #00:35:03-4#

Interviewerin: Sie haben jetzt gerade genickt, ähm Frau (Name der Teilnehmerin 4 aus Datenschutzgründen ausgelassen) als es um die Akkulaufzeit und um dieses, dass es irgendwelche Bewegungen macht, die man nicht haben will. Das heißt, Sie kennen das auch? #00:35:14-0#

Teilnehmerin 4: Die sind ganz krass, teilweise. Mh, mein Sohn ist Informatiker (Interviewerin: Mhm (bejahend)), der kennt sich ja ‘n bisschen damit und ich hab ja ‘n iPad, wo ich auch meine Hände mit steuern kann (Interviewerin: Ja.). U:nd wenn er mich ärgern will, dann setzt er sich hin, in die Ecke irgendwo, wenn ich das nicht merke und der steuert meine Hand (alle lachen). Also der kann das Ruckzuck machen, ‘ne? (Interviewerin: Mhm (bejahend)) Der macht mir dann Griffe rein, so Pommesgabel und sowas. Ja und amüsiert sich dann, ‘ne? Ähm das finde ich dann nicht so lustig und was er sagt: Das kann also jeder von außen steuern

(Interviewerin: Mhm (bejahend)). Das find ich also auch nicht so lustig. Da haben wir auch schon mal (Name eines Orthopädietechnikers aus Datenschutzgründen ausgelassen) drauf angesprochen, keine Rückmeldung bekommen. (Interviewerin: Mhm (bejahend)) #00:36:12-1#

Interviewerin: Also einfach das Gefühl, dass auch jemand anders sich da einhacken könnte? #00:36:17-1#

Teilnehmerin 4: Es könnte jetzt, wenn jetzt jemand mich hier steuern würde, (Interviewerin: Mhm (bejahend)) mit 'm iPod, könnte ich sonst was machen (Interviewerin: Mhm (bejahend)), ohne dass ich irgendwas machen kann. (Interviewerin: Mhm (bejahend)) Und das ist nicht ok. (Interviewerin: Ja.) Und eben Akku. Der ist also ziemlich mau. (Interviewerin: Mhm (bejahend)) Also wenn ich den ganzen Tag was mache und so, jeden Tag muss ich ranhängen, sonst-. Ich hab's mal vergessen, nächsten Morgen saß ich natürlich da und hab nichts gehabt, 'ne? (Interviewerin: Mhm (bejahend)) #00:36:42-1#

Interviewerin: Wie ist das bei Ihnen, Herr (Name von Teilnehmer 5 aus Datenschutzgründen ausgelassen), Herr (Name von Teilnehmer 9 aus Datenschutzgründen ausgelassen) mit der Akkulaufzeit oder mit solchen-. Sie haben ja schon gesagt, dass sie oft was macht, was Sie nicht machen soll, aber mit der Akkulaufzeit? #00:36:50-2#

Teilnehmer 9: Da kann ich le-&im Moment noch nicht zu sagen, weil ich die alle halbe Stunde, alle Stunde dann wieder anschließe (Interviewerin: Ja, mhm (bejahend)). Ich brauch circa zwei bis drei Stunden, bis sich das alles regeneriert im Arm (Interviewerin: Mhm (bejahend)), dann sch-&bau ich sie dann wieder für 'ne halbe Stunde an (Interviewerin: Mhm (bejahend)). Das ärgert mich fürchterlich natürlich, (Teilnehmerin 4: Mhm (bejahend)) dass ich nicht länger kann (Interviewerin: Mhm (bejahend)), aber- #00:37:05-8#

Interviewerin: Ok, das heißt aber einfach, die ist halt noch nicht den ganzen Tag so im Gebrauch, dass man da eine Auskunft geben könnte? (Teilnehmer 9: Nein.) Mhm (bejahend). Und bei Ihnen, Herr (Name von Teilnehmer 5 aus Datenschutzgründen ausgelassen)? #00:37:10-8#

Teilnehmer 5: Jetzt mit diese Prothese? #00:37:13-7#

Interviewerin: Genau. Mit der Prothese- #00:37:17-6#

Teilnehmer 5: Äh ich trage ganze Tag (Interviewerin: Ja.), Beispiel ich kann drei Tage ohne äh laden (Interviewerin: Ah, mhm (bejahend)), geht das. Weil (hustet) mit der ander Prothese was ganz anders, ich habe immer, ich musste ein Ersatz-äh-akku bei mir haben- #00:37:38-8#

Interviewerin: Darf ich ganz kurz unterbrechen? Die andere Prothese, wa-&können Sie kurz nochmal sagen was für eine das war? #00:37:44-3#

Teilnehmer 5: Das war die gleiche Prothese, aber die Akku war ganz anders. Das war hier draußen. #00:37:49-4#

Interviewerin: Konnte man austauschen? #00:37:50-6#

Teilnehmer 5: Austauschen. Könnte man das austauschen. Äh ich arbeite als Reinigungskraft, Beispiel. (Interviewerin: Mhm (bejahend)) Dann ein Tag ich wollte die Kanne sauber machen, in mein Arbeit hier in Zoo, da (Interviewerin: Mhm (bejahend)) und dann Kanne ist in meine Hand geblieben, weil Akku war leer und dann die Ersatzakku war in mein Jacke, Zuhause. (Interviewerin: Mhm (bejahend)) Dann mit Straßenbahn bin ich nach Hause gefahren, dann hab ich das gewechselt. Aber seit ich diese Akku habe, (..???) nach Hause mit Straßenbahn, die alle gucken mich so an. Aber ja, aber das, ich weiß nicht, ich möchte das früh, ich geh zum Arbeit um fünf. Halb vier, halb fünf ich trage diese Prothese (Interviewerin: Mhm (bejahend)) und das bis zum Abend zehn Uhr, elf Uhr. Hab‘ ich das in mein Hand und wie ich gesagt habe: Ich koche Zuhause jeden Tag, weil die essen nicht von draußen. Meine Frau und Tochter sie geht nicht zum irgendwohin, ich muss jeden Tag kochen und jeden Tag was sie wünscht, muss ich das kochen. Eine esst das, die andere esst anders und ich&ich mach auch natürlich, die&weiß nicht, ich sauge, alles, ich mach alles. Haushalt. #00:39:06-7#

Interviewerin: Und das heißt die Prothese hält alles durch? #00:39:09-7#

Teilnehmer 5: Hält das alles durch und dann Abend, wenn ich will das laden, dann zwei äh Punkte ist leer, das bedeutet ich habe noch die Hälfte. #00:39:21-0#

Interviewerin: Ja. #00:39:22-9#

Teilnehmerin 4: Mh. #00:39:22-9#

Interviewerin: Ok, das heißt bei Ihnen hält das lange. #00:39:23-0#

Teilnehmer 3: Nur ein Motor. #00:39:25-0#

Interviewerin: Ja. #00:39:25-5#

Teilnehmer 3: Gegen fünf Motoren. #00:39:27-3#

Interviewerin: Ja, klar. Ähm gibt’s aber sonst noch Nachteile? #00:39:30-2#

Teilnehmer 5: Aber dieser Motor ist nicht stark wie die andern. (Interviewerin: Mhm (bejahend), ja.) Weil die andre können noch mehr Möglichkeit (Interviewerin: Ja.), aber dann das äh schluckt die Akku. Weil ich habe das zwei Woche, wie ich gesagt habe. Schade gibt’s kein andre Möglichkeit außer (Name eines Prothesenherstellers aus Datenschutzgründen ausgelassen) und die andre. (Interviewerin: Mhm (bejahend), ja.) Das ist Problem. #00:39:45-2#

Interviewerin: Mhm (bejahend), ja. Aber gibt’s noch andere Nachteile die jetzt noch jemandem einfallen, die nicht genannt wurden? Irgendwas, wo Sie sagen das ist auch noch was, was stört, ‘ne Funktion vielleicht, oder-? #00:39:52-7#

Teilnehmerin 4: Das hat ja so eine- #00:39:53-9#

Teilnehmer 3: Natürlich. Entschuldigung. #00:39:55-9#

Teilnehmerin 4: Ich hatte auch so eine (Interviewerin: Mhm (bejahend)) und hatte die Alarmanlagen im Kaufhaus ausgelöst. #00:40:01-0#

Teilnehmer 5: Ja, ich die auch. Richtig! #00:40:01-8#

Teilnehmerin 4: Also- #00:40:02-6#

Interviewerin: Woran liegt das? #00:40:10-6#

Teilnehmerin 4: Gleiche Frequenz. #00:40:09-0#

Interviewerin: Ok, ja. #00:40:05-8#

Teilnehmer 5: Überall: Kaufland, Kaufhof, Realkauf und dann, das löst immer die Alarmanlagen. #00:40:11-4#

Teilnehmer 3: Aber was sendet denn da in der Hand? #00:40:13-3#

Teilnehmerin 4: Es sind ja auf der gleichen Frequenz. #00:40:14-5#

Teilnehmer 3: Aber was denn? Also das ist so ‘ne SensorHand Speed, ‘ne? (wendet sich an Teilnehmer 5 und deutet auf seine Prothese) #00:40:18-3#

Teilnehmer 5: Ja. #00:40:20-2#

Teilnehmer 3: Da ist doch nichts drin, das sendet. Da ist ‘n Sensor drin der merkt, ob sich was bewegt und&und der&die Stromübertragung geht über Kabel. Also ich wüsste nicht was da senden sollte. #00:40:28-8#

Teilnehmer 5: Ich weiß es auch nicht. #00:40:27-6#

Teilnehmerin 4: Irgendwas, was da- #00:40:29-8#

Teilnehmer 3: Also ich könnte es verstehen so, diese Hand (zeigt auf seine Prothese) (Teilnehmerin 4: Die sowieso.) kann sich über Bluetooth mit nem&mit nem Rechner verbinden, aber da braucht erstmal der Rechner, da müsste so ‘n (Dongel?) reinstecken, wo ‘n Bluetoothsender- und Empfänger drinsteckt und nur dann. Also die Konfiguration können ‘se darüber möglicherweise feststellen. #00:40:42-3#

Teilnehmerin 4: Mit der Hand hatte ich das auch noch nicht, aber mit der Hand hab‘ ich das auch. #00:40:41-8#

Teilnehmer 5: Aber jetzt paar Jahre, jetzt seit paar Jahre, hat das auch geändert jetzt (Teilnehmerin 4: Mhm (bejahend), genau.), weil äh damals bei C&A hat das die Alarm gelöst und dann kommt sofort die Sicherheit. (Teilnehmerin 4: Jaja, genau.) Und dann die sind auch nicht nett. (Teilnehmerin 4: Nee nee.) Die: „Whuaaaaa!“ #00:40:58-3#

Teilnehmerin 4: Jaja. #00:41:01-7#

Teilnehmer 5: Dann hab‘ ich gesagt: „Ein Moment! Dann ich&rufen Sie erstmal Polizei.“. (Teilnehmerin 4: Mhm (bejahend)) Die Polizei soll hierherkommen. Weil ich habe erzählt, ich habe eine Prothese. Nein, ich muss mit ihm zusammen zum ein Zimmer, die sollen mich untersuchen (Teilnehmerin 4: Nee!). Dann hab‘ ich gesagt: „Nein! Rufen Sie Polizei!“. „Nein.“, dann ich habe angerufen. #00:41:19-6#

Teilnehmerin 4: Mhm (bejahend) #00:41:19-6#

Teilnehmer 5: Dann ich habe gesagt: „So.“. „Und jetzt“, hab‘ ich gesagt, „die Polizei die sollen das schriftlich zu mir geben, weil ich mache (Teilnehmerin 4: Mhm (bejahend)) Anzeige“. (Teilnehmerin 4: Mhm (bejahend)). Dann die sollen eigene Sachen, diese Alarmsachen (Teilnehmerin 4: Mhm (bejahend)) noch bisschen ändern oder verbessern. Die sollen das machen, nicht ich. #00:41:39-5#

Teilnehmerin 4: Nee, nee. #00:41:41-3#

Teilnehmer 5: Weil haben Sie recht. Bei Realkauf (Interviewerin: Mhm (bejahend)) damals, hat immer passiert. C&A- #00:41:51-4#

Teilnehmerin 4: (..??) #00:41:50-1#

Interviewerin: Das heißt einfach, die Alarmanlage (geht an?) #00:41:52-1#

Teilnehmer 5: Ja, sofort. #00:41:52-1#

Teilnehmerin 4: Sofort gehen die an. #00:41:52-1#

Interviewerin: Ja, ja. Gibt’s da jetzt grade noch zu den Nachteilen äh irgendwas, was jemand ergänzen möchte? #00:42:01-2#

Teilnehmer 3: Also ich hab‘ ja Erfahrung mit ‘ner Vielzahl von Prothesen gemacht. (Interviewerin: Ja.) Äh ich glaub ich hab‘ einmal so alle ausprobiert, was (Interviewerin: Mhm (bejahend)) wir hier am Tisch sitzen haben und ich glaube, so ‘ne eindimensionale Prothese, die sich so wie die von Herrn (Name von Teilnehmer 5 aus Datenschutzgründen ausgelassen) schnell bewegt und äh (Interviewerin: Mhm (bejahend)) ist glaub ich in den meisten Fällen nützlicher (Interviewerin: Mhm (bejahend). Also ich hab‘ mal irgendwo ‘nen&und während auf der einen Seite, auf der andern Seite ist natürlich ‘ne Prothese mit fünf Fingern so vom sozialpsychologischen äh Standpunkt her (Interviewerin: Mhm (bejahend)) ist sie einfach hübscher. (Interviewerin: Mhm (bejahend)) Und ich&ich find’s fair, wenn man beide hat. Und- #00:42:51-4#

Interviewerin: Ja, aber, da&da unterbrech‘ ich ganz kurz, aber das heißt, dass manchmal sogar das, dass man lieber auf Funktionen verzichtet und sagt dafür sind die Funktionen die da sind funktionieren lange, eben vom Akku her und funktionieren, als das man sagt ich hab ganz viele, sondern-. Kann man das so sagen? #00:43:13-4#

Teilnehmer 3: Genau das ist der Punkt. #00:43:14-9#

Teilnehmerin 4: Genau. #00:43:16-3#

Interviewerin: Mhm (bejahend), ok. #00:43:17-6#

Teilnehmer 3: Ich hab‘ mal irgendwo ‘n Film gesehen. Ich glaub es war auf ‘ner Messe in Leipzig, so ‘ne Rehamesse in Leipzig. Da ging’s um irgendwie ‘ne myoelektrische Prothese für die dritte Welt. Die ähnelte so ’n bisschen diesem (..?)-Haken (Interviewerin: Mhm (bejahend)) aber war grau, aus Plastik, relativ preiswert herzustellen und tat einfach, man hatte ‘n Haken, um (Interviewerin: Mhm (bejahend)) was schweres zu tragen und das Ding klappte sich auf uns zu, damit man was greifen konnte. Und ich fand, das war gar nicht so ’ne schlechte Idee. (Interviewerin: Mhm (bejahend), ja.) Und was ich auch finde ist, wenn man’s dann schon (kurze Pause) wie soll ich das sagen? Was ich wichtig finde: Es darf ruhig technisch aussehen, aber es sollte ein einigermaßen schickes Design haben (Interviewerin: Mhm (bejahend)). Also diese, wie soll ich das sagen? Ohne jetzt Ihnen zu nahe treten zu wollen (wendet sich an Teilnehmer 5). #00:43:54-5#

Teilnehmer 5: Nein, nein. #00:43:54-5#

Teilnehmer 3: Äh ich finde, Sie machen das echt ganz Klasse. Ich glaube, Sie sind fast der Geschickteste von uns allen hier. Aber&aber ich finde dieses fleischfarbene Gummidesign so richtig kacke (lachend). Weil wenn Sie sowas tragen, sehen Sie, auch wenn Sie es gar nicht sind, behindert aus (Interviewerin: Mhm (bejahend)) (Teilnehmerin 4: Mhm (bejahend)). Und ich glaube, es ist Unsinn zu versuchen das zu verstecken was man hat (Interviewerin: Ja.). Dass man sozusagen anders ist. Sie verstecken ja auch nicht, dass Sie blaue Augen haben oder äh rote Haare oder sonst irgendwas (Interviewerin: Mhm (bejahend)). Oder wenn Sie die Sportlerin da angucken (zeigt auf ein Bild das im Raum hängt) mit ihrem Kohlefaserfuß äh der ist einfach der Funktion wegen so, das muss so sein. Und äh, ich würde mir was wünschen, das richtig cool aussieht, richtig toll funktioniert und eigentlich vergleichsweise simpel ist. (Interviewerin: Mhm (bejahend), ja.) So nach den Erfahrungen, die ich so gemacht habe. #00:44:36-4#

Interviewerin: Mhm (bejahend). Um jetzt noch, weil wir sind schon fast einfach bei der Hälfte der Zeit und jetzt hat sich niemand gemeldet, ich würde jetzt gleich nach der nächsten Frage sagen, dass wir einfach die Pause machen. Ähm, aber vor der Pause würde ich gerne noch einmal fragen: Gibt’s Situationen in denen Sie die Prothese nicht nutzen, in denen Sie freiwillig sagen: „Ich lass die weg.“? Sie haben ja schon gesagt, Herr (Name von Teilnehmer 9 aus Datenschutzgründen ausgelassen), dass einfach durch die Schmerzen, dass es oft nicht möglich ist, dass es immer nur ähm in Abschnitten geht. Aber gibt’s bei den anderen ‘ne Situation in der Sie sagen, da benutz ich die Prothese nicht? #00:45:05-2#

Teilnehmer 3: Ja. #00:45:05-2#

Interviewerin: Welche? #00:45:03-5#

Teilnehmer 3: Ich segle. Also ich&ich, es gibt halt ‘ne Segelyacht und ich bin mal ausger-&ich hatte diese Prothese an und bin vom Steg über die Heckklappe auf das Boot gestiegen und die Heckklappe war nass, ich bin ausgerutscht (Interviewerin: Mhm (bejahend)) und dann hing ich am (Heckkorb?) und hatte die größte Mühe dafür zu sorgen, dass die Hand nicht ins Wasser kommt. Dann ist nämlich das gut

60 000 Euro Stück einmal in Salzwasser getunkt, hinüber. (Interviewerin: Mhm (bejahend)). Und seitdem hab‘ ich gesagt: „Nur noch mechanisch!“. (Interviewerin: Ja.) und jetzt hab‘ ich so ’n Käpt’n- Hook-Haken. (Teilnehmerin 4 lacht) Für diesen Zweck. #00:45:28-4#

Interviewerin: Das heißt aber es ist- #00:45:33-8#

Teilnehmer 3: Es gibt einfach Umgebungen&Umgebungen in denen so ‘ne Prothese viel zu empfindlich ist. #00:45:37-7#

Interviewerin: Ja. Aber das heißt, wenn die Prothese wasserdicht wäre, wenn das nichts ausmachen würde, würden Sie die auch da benutzen? #00:45:42-8#

Teilnehmer 3: Natürlich! #00:45:42-8#

Interviewerin: Ja. #00:45:44-7#

Teilnehmer 3: Klar! #00:45:44-7#

Interviewerin: Mhm (bejahend). Und bei Ihnen Frau (Name von Teilnehmerin 4 aus Datenschutzgründen ausgelassen)? #00:45:45-7#

Teilnehmerin 4: Ich leg ‘se eigentlich nur zum Schlafen und zum Duschen ab (lachend). Sonst- #00:45:51-6#

Interviewerin: Also auch der Aspekt Wasser und halt einfach nachts? #00:45:54-2#

Teilnehmerin 4: Mhm (bejahend), ja. #00:45:52-9#

Interviewerin: Ja, ok. Aber sonst gibt’s keine Situation? #00:45:57-5#

Teilnehmerin 4: Nachts lad ich die, aber sonst hab ich ‘se immer an. #00:46:01-2#

Interviewerin: Ja. #00:46:04-9#

Teilnehmer 3: Also ich benutz dann ja nicht keine Prothese (Interviewerin: Genau, Sie-), nur eine andere, die halt diesen Umweltbedingungen einfach gewachsen ist. (Interviewerin: Ja.) Das läuft dann im Wesentlichen auf schlichte Mechanik raus. Beim Fliegen ist es genau dasselbe. Da haben Sie das Problem je höher Sie kommen, desto kälter wird es. Je kälter es wird, desto geringer ist die Akkukapazität und je geringer die Akkukapazität, desto größer die Gefahr, dass das Ding dann, wenn’s funktionieren soll, nicht tut. (Interviewerin: Mhm (bejahend)) #00:46:33-7#

Interviewerin: Ja, aber das heißt auch dann nicht ohne Prothese, sondern eben einfach nur mit einer, die dem halt gewachsen ist? #00:46:40-0#

Teilnehmer 3: Ganz genau, ja. #00:46:38-5#

Interviewerin: Ja. Also- #00:46:41-9#

Teilnehmer 3: Also wenn&wenn man was bauen könnte, dass sozusagen so ‘ne

geringe Stromaufnahme und so ‘ne hohe mechanische Stabilität hat, dass man&dass es sozusagen unter allen Bedingungen funktionieren würde (Interviewerin: Mhm (bejahend)), dann würd‘ ich, wenn das Design noch einigermaßen hübsch ist, d-&sozusagen mit einem auskommen und das immer benutzen. (Interviewerin: Mhm (bejahend)) Das wär‘ mir eigentlich am liebsten. (Interviewerin: Mhm (bejahend)) #00:47:06-6#

Interviewerin: Ja. #00:47:06-6#

Teilnehmer 3: Aber so ist es halt noch nicht. #00:47:07-2#

Interviewerin: Ja. Und Herr (Name von Teilnehmer 5 aus Datenschutzgründen ausgelassen), bei Ihnen? #00:47:09-9#

(kurze Pause) #00:47:12-8#

Teilnehmer 5: Auch nur wenn ich zum Schlafen geh oder zum Schwimmen (Interviewerin: Mhm (bejahend)), sonst ich trage immer. #00:47:16-4#

Interviewerin: Und wie ist es beim Schwimmen: Tragen Sie da gar nichts oder gibt’s dann was anderes, was Sie tragen? #00:47:26-4#

Teilnehmer 5: Nein, hab‘ ich gar nichts. Hab‘ ich nur das und dann wenn wirklich, wie er gesagt hat, wenn gibt’s andre Möglichkeit mit Prothese schwimmen kann (Interviewerin: Mhm (bejahend)), dann ich hätte gerne mit Prothese schwimmen. Warum nicht? (Interviewerin: Mhm (bejahend)) Aber-. #00:47:33-5#

Teilnehmerin 4: Gibt es nicht. #00:47:33-5#

Teilnehmer 5: Gibt es nicht. (lachend) #00:47:35-4#

Teilnehmer 9: Oh doch, gibt es. #00:47:36-9#

Teilnehmer 5: Oder, oder wir können selber etwas bauen. #00:47:38-2#

Teilnehmer 3: Nein, das ist nicht mehr so weit weg. Ich glaube es gibt inzwischen ‘nen Bein, mit dem man d-&das Genius Bein von (Name eines Prothesenherstellers aus Datenschutzgründen ausgelassen), damit kann man ins Wasser gehen. #00:47:41-4#

Teilnehmerin 4: Mhm (bejahend) #00:47:43-7#

Teilnehmer 9: Ist das so? Oder-. #00:47:46-0#

Teilnehmer 3: Es gibt&ich hab‘ irgendwo ‘n Film gesehen, da stapft einer mit ‘nem elektrischen Kniegelenk ins Wasser. Ja. #00:47:55-4#

Begleitung Teilnehmer 5: Darf ich vielleicht nur eins ergänzen? (Interviewerin: Mhm (bejahend)) Wir sind seit ‘92 zusammen und seit ‘96 verheiratet. Es gehört dazu, es gehört zu dem Menschen (Interviewerin: Mhm (bejahend)). Also ich glaube Menschen die ‘ne Amputation haben und ‘ne Prothese tragen, unterscheiden sich

von denen, die ‘ne Prothese meiden. Wenn jemand ‘ne Prothese trägt, dann trägt der Mensch die Prothese nicht für einen Anlass, oder für ‘ne gewisse Aufgabe (Interviewerin: Mhm (bejahend)), die gehört dazu, als wäre es ein Körperstück. Man kann glaube ich dann nicht mehr drauf verzichten (Interviewerin: Mhm (bejahend)), allein wenn wir auf die Körperhaltung achten, sehen wir drei mit zwei Händen, dass es ‘ne Stütze ist, er spielt ständig zum Beispiel, macht sie auf und zu (zeigt auf ihren Mann). Sie machen die gleiche Bewegung seh‘ ich gerade, wie bei meinem Mann (wendet sich an Teilnehmer 3), sie legen auch Ihre Hand drauf (wendet sich an Teilnehmerin 4). (Interviewerin: Mhm (bejahend)) Es gehört zu diesen Menschen. Ich glaube ein Verzicht auf eine Prothese, ich kann’s ja nur als Beobachtende&nah Beobachtende sagen, die äh die ist nicht gegeben, es ist nicht möglich auf ‘ne Prothese tagsüber zu verzichten und zu sagen die wird nur dann abgenommen, wenn sie saumäßig weh tut, wenn äh irgendwas mit der äh Innenschaft nicht stimmt, er hatte sehr lange Jahre Reaktionen&Hautreaktionen, dass das falsch gegossen war, dass äh dass das einfach nicht gut war für die Haut, er hat’s nur abgenommen, weil’s weh tat. So und äh auch da&auch wegen den Druckstellen (Teilnehmer 3: Ok.), dass es nicht mehr erträglich war. Und sonst äh die haben sie auch- #00:49:43-9#

Teilnehmer 5: (zeigt Druckstellen an seinem Stumpf) Aber Krankenkasse will das nicht zahlen, deswegen ich muss das tragen. Gucken Sie wie sieht das aus! #00:49:46-1#

Begleitung Teilnehmer 5: Ja. Und wir zum Beispiel sind grade in einem Widerspruchsverfahren mit der Krankenkasse, äh es wird ‘ne neue Prothese wird abgelehnt, weil man sagt, er hat eine myoelektronische Prothese und die würde ja ausreichen äh da ham‘ wir jetzt ‘n ärztliches Gutachten besorgt, ‘n ganz äh bitter bösen Widerspruch dazu geschrieben. Äh der Umgang mit Menschen, die ‘ne Amputation haben fehlt den Krankenkassen, auch den Berufsgenossenschaften, sie unterschätzen eigentlich die Wichtigkeit von einer Prothese (Interviewerin: Mhm (bejahend)) und beziehen sich auf irgendwas, was da ist und sieh zu, dass du klarkommst. Dass das ‘n Stück Lebensqualität und Selbstständigkeit und Sicherheit gibt, (Interviewerin: Mhm (bejahend)) unabhängig von der technischen Anwendung, von der mechanischen Anwendung, das vergisst man was das auch mit der Psyche macht. Das ist super wichtig, dass sie funktioniert, dass sie passt von der Optik, von der Farbe, von der Länge, von der Größe. Da fehlt dieses&dieses&dieses Schicke was Sie gesagt haben, dieses Feingefühl. Es gibt ganz viele Prothesen, die sehen einfach nur hässlich aus und passen nicht zu der Optik und man nimmt’s an, weil’s dann die Kasse halt zahlt (Interviewerin: Mhm (bejahend)). #00:50:50-7#

Teilnehmer 3: Muss man aber nicht. #00:50:50-7#

Begleitung Teilnehmer 5: Nein, will ich halt nur äh so sagen. Aber äh das ist schwierig. Halt der Kampf ist schwierig und mühselig und Sie müssen so viel Geduld investieren, dass äh sorgt dafür, dass einige auch äh an der seelischen Belastung halt drunter leiden (Interviewerin: Mhm (bejahend)), weil man abhängig von diesem Gerät ist. Und das ist echt kein Luxusstück, womit ich mal schicke ‘ne Gurke schneide, sondern es ist ‘ne Lebensqualität- #00:51:10-0#

Teilnehmer 3: Wieso? ‘Ne Gurke schneiden ist kein Luxus. #00:51:11-5#

Begleitung Teilnehmer 5: Nein, sag ich jetzt nur. #00:51:12-3#

Teilnehmer 3: Wenn man ‘ne Gurke essen möchte, muss man ‘ne Gurke schneiden. Was ist denn daran Luxus? #00:51:13-7#

Begleitung Teilnehmer 5: ‘Ne, so. Aber ‘ne? Wegen der Prothese, also damit dann schneiden. Also es ist, es ist, ein Stück weit äh gehört es zu einem und äh die muss funktionieren, auf die kann mein Mann hier überhaupt nicht verzichten. Und von der Optik her kann ich sagen, sehen Sie mal die Fingerlänge an und sehen Sie mal seine tatsächliche Hand an. Also auch das, da fehlt’s wirklich am Design, an der Grafik halt. (Interviewerin: Mhm (bejahend)) #00:51:35-2#

Teilnehmer 9: Jetzt zu der Michelangelo-Hand. (Name eines Prothesenherstellers aus Datenschutzgründen ausgelassen) hat nämlich nur eine Größe von der Hand und bei einigen da konnten wir zwischen (fünf?) unterscheiden, ‘ne? Also die Größe von der Hand, die hat man dann auch für links genommen, dass die Finger genauso lang sind wie in der rechten Seite, ‘ne? Und das, dass fand ich nämlich sehr, sehr gut, dass man da doch noch- #00:51:53-7#

Teilnehmerin 4: Die haben auch nur Herrenhände. Die Finger sind für Frauen nämlich zu lang. #00:51:56-9#

Teilnehmer 9: Ja? #00:51:56-9#

Teilnehmer 3: Die sind auch für ’n Kerl zu lang. #00:51:59-7#

Teilnehmerin 4: Also da, (kurze Pause) auch hier will ich mich- #00:52:00-2#

Teilnehmer 3: Aber gut mit den I-limb Dingern bin ich eigentlich durch. #00:52:04-0#

Teilnehmerin 4: Ja, hab‘ ich auch gesagt. Die sind zu lang. Und da hat man mir gesagt, das haben ‘se extra gemacht. Ich sag, warum das denn? Weil bei der andern waren ‘se kürzer und dann haben ‘se gesagt: „Nein, damit man ‘n besseren Griff hat.“ #00:52:17-9#

Teilnehmer 9: Das ändert ja nichts an der Situation. #00:52:19-7#

Teilnehmer 3: Naja, da ändert sich die Öffnungsweite, die Sie erreichen können. #00:52:26-7#

Teilnehmerin 4: Ja. #00:52:28-5#

Teilnehmer 3: Was Sie hier zwischen greifen können, das ist ja abhängig davon, wie lang die Finger letztlich gemacht werden. (Teilnehmerin 4: Genau.) Wenn Sie die Finger zu kurz machen, sind die Gegenstände die Sie anfassen können nur sehr begrenzt. #00:52:39-5#

Teilnehmerin 4: Ja, aber- #00:52:40-0#

Interviewerin: Ich würd‘ da ganz kurz einmal unterbrechen. Wir würden jetzt einmal die Pause machen und einfach sagen 15min, ich guck grad mal, wir haben ähm 20

vor sieben, dann sagen wir einfach bis fünf vor sieben machen wir ‘ne Pause, hier steht was zur Stärkung, Wasser, Kaffee, weiß nicht ob so spät noch jemand ‘n Kaffee möchte, aber er steht bereit. Wenn jemand zur Toilette muss äh, zeig ich gerne den Weg. Genau. Und dann sagen wir einfach 15 Minuten und danach geht’s weiter. #00:53:12-4#

Teilnehmerin 4: Ok. #00:53:12-4#

(kurze Pause) #00:53:12-4#

Interviewerin: Ja, wir haben vor der Pause darüber gesprochen in welchen Situationen Sie die Prothese weglassen, bewusst eben, wir haben gesagt mit dem Wasser und, dass es auch manchmal zu Schmerzen kommt. Ich wüsste gerne, was passiert, wenn die Prothese kaputt geht? Sie haben das ganz am Anfang gesagt, wenn die kaputt ist, dann bin ich erstmal aufgeschmissen. Ähm genau, was passiert dann, was fehlt dann, vielleicht auch was nutzen Sie stattdessen? Wenn Sie darauf einmal antworten würden. Weiß nicht wer (lachend)? #01:17:12-4#

Teilnehmer 5: Wenn Prothese kaputt ist, (Interviewerin: Ja.) dann unsere Leben ist kaputt (Interviewerin: Mhm (bejahend)). Und dann, wenn wir kommen hierher, Beispiel (Name eines Sanitätshauses aus Datenschutzgründen ausgelassen), dann das&wenn das dauert ein Monat, (Interviewerin: Mhm (bejahend)) ein Monat lang wir können nicht so viel machen und dann das dauert sowieso ein Monat oder zwei Monate. (Interviewerin: Mhm (bejahend)) Äh solche Sachen muss man auch lösen. Weil, ohne Prothese (kurze Pause) unsere Lebensqualität ist null. (Interviewerin: Mhm (bejahend)) #01:17:37-9#

Teilnehmerin 4: Mhm (bejahend) #01:17:38-4#

Teilnehmer 5: Also bei meine. Ich sage das so. Weil dann ich will meine Hose anziehen. Mit Prothese geht das besser. Ich will was kochen, mit Prothese geht das besser. Egal was ich mache, ich brauch die Prothese. #01:17:52-6#

Interviewerin: Mhm (bejahend). Und wenn Sie kaputt ist und in der Reparatur ist, haben Sie was anderes was Sie dann nutzen- #01:17:56-3#

Teilnehmer 5: Nein. Die geben keine Ersatzprothese. #01:17:58-1#

Teilnehmer 3: Echt nicht? #01:17:56-4#

Teilnehmer 5: Nein. #01:17:59-9#

Interviewerin: Und-. #01:18:00-5#

Teilnehmer 5: Er hat Beispiel kaputt, dann äh man kommt hier zu (Name eines Sanitätshauses aus Datenschutzgründen ausgelassen), (Name eines Orthopädietechnikers aus Datenschutzgründen ausgelassen) kann, dass&dass nicht ändern, äh dann&dann er schickt das zum Reparatur (Teilnehmer 3: Mhm (bejahend)), wenn jemand hat ein Ersatz Zuhause, dann kann man das tragen. (Interviewerin: Ja.) Aber sowieso wir haben kein Ersatzhand, weil (Interviewerin: Ja, aber-) bis zum Hand richtig kaputt geht, die zahlen das nicht. Und dann, ok, Schaft

haben wir und dann äh wir haben ein kaputte Hand, das bewegt sich nicht. Ok, dann kann man auch was machen. Was kann man machen? Wenn man will was schreiben, dann kann man so machen (Interviewerin: Festhalten.) aber Prothese funktioniert nicht. Wenn man will was tragen, dann kann man hier tragen, (deutet auf seinen Unterarm) nicht hier (deutet auf seine Prothesenhand) (Teilnehmerin 4: Mhm (bejahend)). Äh wenn man will etwas machen, dann kann man das ohne Bewegung, kann man so und dann Beispiel das und dies. (Interviewerin: Ja.) #01:18:46-5#

Interviewerin: Aber Sie haben vorher gesagt, Sie haben&Sie hatten davor ‘n älteres Modell. Haben Sie da zum Beispiel die Möglichkeit, dass wenn die Prothese kaputt ist, dass Sie das alte Modell benutzen? Oder- #01:18:57-3#

Teilnehmer 5: Das ist sowieso kaputt. #01:18:55-6#

Interviewerin: Ok, das ist kaputt und funktioniert gar nicht? #01:18:58-3#

Teilnehmer 5: Das ist auch funktioniert gar nichts. #01:18:59-9#

Interviewerin: Ja, ok. #01:18:59-9#

Teilnehmer 5: Hand funktioniert sowieso gar nichts Zuhause. #01:19:01-7#

Interviewerin: Mhm (bejahend) #01:19:02-9#

Teilnehmer 5: Das, wir haben auch kein Möglichkeit, das zum Rep-. Ich habe damals das überlegt. Hab‘ ich extra gefragt, hab ich gesagt: „(Name eines Orthopädietechnikers aus Datenschutzgründen ausgelassen), wenn ich das reparieren lasse, zahlt das Krankenkasse?“. Er hat gesagt: „Nein.“, ich muss selber das zahlen. Und dann hab‘ ich gesagt: „Haben Sie ein Möglichkeit, das zu gucken?“. Er ist sehr nette Mensch, er ist sehr der gute Mensch, aber er darf auch nicht was machen, vor die oben fragen. Und dann, wenn er fragt den: „Ja äh kann ich das nur schauen was kaputt ist, dann kann ich ein Kostenvoranschlag machen?“, er darf das auch nicht machen. (Interviewerin: Mhm (bejahend)) Weil er (..?) irgendwohin was zu sagen. #01:19:40-9#

Interviewerin: Das heißt wenn Ihre Prothese kaputt ist (Teilnehmer 5: Kaputt ist.), haben Sie nichts anderes? #01:19:44-4#

Teilnehmer 5: Nicht. Wa-&äh&&weil wir denken immer Hand, weil äh (Interviewerin: Ja.) Hand geht kaputt. (Interviewerin: Ja.) Diese Hand ist ganz so, das geht kaputt. (Zeigt auf die Hand seiner Prothese) (Interviewerin: Ja.) Das geht kaputt. Hier funktioniert alles. (Teilnehmerin 4: Mhm (bejahend)) Aber wenn das kaputt ist, dann wenn das wir hier abgeben (Teilnehmer 9: Genau wie bei mir.) dann wir müssen, oder äh (Teilnehmerin 4: Mhm (bejahend)) besser, wenn so ein Möglichkeit, egal alt, egal alte Technik (Interviewerin: Mhm (bejahend)), egal was. Hauptsache ein Ersatzhand (Interviewerin: Mhm (bejahend)) zu uns geben, bis unsere Hand zurückkommen. (Teilnehmerin 4: Ja.) Wir sollen das benutzen. #01:20:10-5#

Interviewerin: Ja. Und wenn Sie jetzt aber ganz ohne einen Ersatz sind, gibt’s irgendwas Zuhause, was Sie stattdessen benutzen? Irgendein Hilfsmittel? Irgendwas was Sie (kurze Pause), weiß nicht wie Herr (Name von Teilnehmer 3 aus

Datenschutzgründen ausgelassen) erzählt hat, er schraubt irgendwas dran, oder macht irgendwas? (Teilnehmer 5 schüttelt den Kopf) Haben Sie nichts, ok. #01:20:21-7#

Teilnehmer 5: Ich habe nichts. #01:20:22-5#

Interviewerin: Ok, mhm (bejahend), ja. Und wie ist das bei den anderen? Wenn die Prothese kaputt geht? #01:20:28-2#

Teilnehmerin 4: Muss ich herkommen und reparieren lassen. #01:20:29-2#

Interviewerin: Haben Sie irgendeinen Ersatz den Sie dann benutzen können? #01:20:35-3#

Teilnehmerin 4: Ich hab' noch die von (Name eines Prothesenherstellers aus Datenschutzgründen ausgelassen), die andere (Interviewerin: Mhm (bejahend)), die ich auch für Gartenarbeiten und so weiter nehme, weil mit der (zeigt auf die Prothese die sie trägt) kann ich das nicht machen (Interviewerin: Mhm (bejahend)). Ich hab' das jetzt ja vor 'nem Jahr bekommen (Interviewerin: Ja.) und mh- #01:20:49-6#

Teilnehmer 3: Wie viel Finger? (kurze Pause) Können Sie ruhig sagen. #01:20:53-2#

Teilnehmerin 4: Die hab' ich laufend, also jede Woche war ich fast hier und jede Finger wurden ausgetauscht schon. #01:20:59-8#

Interviewerin: Mhm (bejahend) #01:21:02-1#

Teilnehmerin 4: Schon alleine, wenn man in die Jacke reingeht knicken sie weg oder sonst wie was. #01:21:09-3#

Interviewerin: Das heißt die sind auch sehr anfällig? #01:21:07-5#

Teilnehmerin 4: Ja, <u>sehr</u> anfällig. Deshalb mh die haben ja immer gesagt, damit kann man 'ne Kiste Bier tragen, (Teilnehmer 3 lacht) äh hab ich noch nicht probiert (lachend), weil ich weiß was dann passiert. #01:21:22-9#

Teilnehmer 3: Geht. Ein Mal. #01:21:25-3#

Teilnehmer 9: Jo. #01:21:25-8#

Teilnehmerin 4: Ja, ein Mal. Dann nie wieder. #01:21:28-1#

Interviewerin: Und was fehlt dann am meisten? Also es ist klar, dass Sie die in allen Bereichen benutzen, aber gibt's irgendwas eben, wenn man den&den Ersatz hat, wenn man solange die Alte benutzt, gibt's da irgendwas, was besonders stört, was besonders fehlt in dem Moment? #01:21:47-5#

Teilnehmerin 4: Mh. Hier kann man feinere Sachen mit machen. (Zeigt auf die Prothese die sie trägt) #01:21:51-5#

Interviewerin: Mhm (bejahend) #01:21:52-9#

Teilnehmerin 4: Also, weil ich hier den Finger auch betätigen kann nur. #01:21:57-9#

Interviewerin: Mhm (bejahend) #01:21:59-8#

Teilnehmerin 4: Bei der anderen hab‘ ich immer nur diesen einfachen Griff. (Interviewerin: Mhm (bejahend), ja.) Und hier kann ich dann ‘n bisschen mehr machen. #01:22:07-5#

Interviewerin: Und nutzen Sie dann ähm außer dieser älteren auch noch irgendein anderes Hilfsmittel? #01:22:14-8#

Teilnehmerin 4: Nee. #01:22:14-8#

Interviewerin: Nee, ok. Ja. #01:22:16-8#

Teilnehmerin 4: Dann bin ich aufgeschmissen (Interviewerin: Mhm (bejahend)) #01:22:19-2#

Interviewerin: Und bei Ihnen, Herr (Name von Teilnehmer 3 aus Datenschutzgründen ausgelassen)? #01:22:19-7#

Teilnehmer 3: Ich hab‘ ‘n ganzen Eisenwarenladen Zuhause (Interviewerin: Mhm (bejahend)). #01:22:24-9#

Teilnehmer 9: Dann kommen wir doch zu Ihnen! #01:22:27-7#

Teilnehmer 3: Wie bitte? (Teilnehmerin 4 lacht) #01:22:29-6#

Teilnehmer 5: Ja, wenn kaputt ist- #01:22:32-0#

Teilnehmer 3: Nein äh, ja das hat sich so durch die, wie soll ich das sagen, das sich so ‘n bisschen durch die Geschichte ergeben (Interviewerin: Mhm (bejahend)). Also ich hatte am Anfang, weil ich sofort wieder fliegen wollte, hab‘ ich die erste Prothese selbst gekauft, die war rein mechanisch (Interviewerin: Mhm (bejahend)). Da war ‘ne (Name eines Prothesenherstellers aus Datenschutzgründen ausgelassen) Hand dran, aber ohne me-&ohne&die war nur fe-&da war nur ‘ne Feder drin, kein Motor und gar nichts. Die hab‘ ich auch immer noch. Und immer dann, wenn’s nicht so drauf ankommt (Interviewerin: Mhm (bejahend)), also (kurze Pause) dann benutz ich die auch hin und wieder mal. Also zum Beispiel beim Segeln oder so ist das hübsch, die macht kein&keine Kratzer ins Gelcoat, die kann ruhig mal nass werden, weil da ist nichts drin, das kaputt gehen kann und äh die hol ich halt raus wenn&wenn die elektrische Hand kaputt ist. Wobei es so ist, dass die I-limb-Hand war relativ häufig kaputt, was ich am Anfang schon geschildert hab (Interviewerin: Mhm (bejahend)). Die haben, also ich glaube die haben ein Problem mit der Konstruktion der Finger, da ist ein Konstruktionsfehler drin. (Interviewerin: Mhm (bejahend)) Und das hört man ja hier auch, alle andern berichten auch, dass sie schon stehen gebliebene Finger gehabt haben (Teilnehmerin 4: Mhm (bejahend)) und äh ich hab dann hat’s ein Treffen mit zwei Herren der Firm-&von (Name eines Prothesenherstellers aus Datenschutzgründen ausgelassen) gegeben und ich hab denen das geschildert und ziemlich drastisch auch geschildert und ihnen auch gesagt, was da&was ich glaube,

dass daran falsch ist (Interviewerin: Mhm (bejahend)) und (kurze Pause) die Hand war, da hatt' ich schon fünf oder sechs Finger durch (Interviewerin: Mhm (bejahend)) und dann haben die auf ihre Kosten diese Hand nochmal repariert und ich hab sie dann aber an die Seite gelegt und hab gesagt, die tut nichts und bin zur Krankenkasse gegangen und hab gesagt: „Ich möcht was stabileres haben.". Haben die auch bezahlt. (Interviewerin: Mhm (bejahend)) Wobei man dazu sagen muss, ich hab das auch wirklich bis zum Landessozialgericht durchgefochten. Also ich glaube die sehen mich irgendwie keine Ahnung von der Aktenlage her „mit dem ist nicht gut Kirschen essen" oder was (Interviewerin: Ja.). Das hat dann 'n bisschen geholfen andere Dinge durchzusetzen. (Interviewerin: Ja.) Und&und für's Grobe benutz ich halt 'n Systemgreifer. Also ich kann immer noch in die Schublade greifen, wenn was kaputt gegangen ist und das andere in der Zeit zur Reparatur bringen, insofern leide ich da nicht sehr. #01:24:21-7#

Interviewerin: Das heißt Sie haben jetzt nicht wie die anderen die Situation, dass Sie sagen dann steht erstmal so das Leben still, weil die Funktionen nicht mehr da sind, sondern Sie behelfen sich einfach mit den anderen- #01:24:30-4#

Teilnehmer 3: Das Leben steht nie still und man muss sich immer behelfen. #01:24:31-0#

Interviewerin: Mhm (bejahend). Aber würden Sie sagen, dass das die Einstellung ist mit der Sie da rangehen, oder ist es einfach das, dass Sie eben auch sagen, ich hab da halt 'ne Schublade und da sind ganz viele Sachen drin- #01:24:40-7#

Teilnehmer 3: Das eine ist ja das Resultat des anderen. #01:24:43-5#

Interviewerin: Ja, mhm (bejahend). #01:24:45-7#

Teilnehmer 3: Also, 'ne? Wenn Sie sich zufriedengeben und sagen: „Das ist halt so.", dann wird es&dann wird's auch so sein. (Interviewerin: Mhm (bejahend) Wenn Sie sich nicht zufriedengeben und sagen: „Da geht noch was." und das auch letztlich auch einigermaßen offensiv verfechten äh dann geht auch was. (Interviewerin: Mhm (bejahend)) Das&und da müssen Sie halt, ich hab' die Erfahrung gemacht, dass die Krankenkassen versuchen erstmal alles abzuwimmeln. Also das ist auch&das geht auch zum Teil wirklich weit unter die Gürtellinie. (Interviewerin: Mhm (bejahend)) Also, als ich gesagt hab, ich&ich fänd es 'ne gute Sache, wenn ich mit dem Fahrrad zur Arbeit fahren könnte, weil, also meine Krankenkasse wirbt in ihrer Kundenzeitschrift dafür, dass man sich bewegen soll. 3000 Schritte am Tag oder das Äquivalent mit dem Fahrrad fahren (Interviewerin: Mhm (bejahend)) oder sonst irgendwas, um Herz-Kreislauf-Erkrankungen vorzubeugen. Hab' ich gesagt, da mach ich gerne mit, ich würde gerne mit dem Fahrrad zur Arbeit fahren. Die Antwort der Krankenkassensachbearbeiterin, als ich eine Prothese haben wollte, die mich in die Lage versetzt den Fahrradlenker anzufassen und zu radeln, war: „Wir sind doch nicht dafür zuständig Ihre Sportgeräte zu bezahlen." (Interviewerin: Mhm (bejahend)). Da hab' ich gedacht, ist das jetzt Sport, wenn ich mit dem Fahrrad zur Arbeit fahren will und tun will, was die Krankenkasse mir eigentlich empfiehlt, nämlich mich zu bewegen? (Interviewerin: Mhm (bejahend)) Und äh ich hab' das nicht auf sich beruhen lassen. Sondern hab das dann wirklich durchgefochten und das Ganze hat dann mit 'nem Vergleich geendet, der darin bestand, dass ich aussuchen durfte was ich haben wollte und die Krankenkasse es bezahlt hat.

#01:26:10-8#

Interviewerin: Das heißt aber, das hör ich jetzt bei allen ein bisschen raus, dass eben wenn die kaputt ist, dass da auch die Kasse ‘n Großteil dazu beiträgt wie’s einem geht. Eben ob man ‘n Ersatz hat, ob man ‘ne andre Versorgung, oder-? Ist das so, da, also kann ich das- #01:26:29-8#

Teilnehmer 3: Natürlich. #01:26:30-9#

Interviewerin: Ja, ok. #01:26:29-1#

Teilnehmer 5: Und die Problem ist äh, wenn man nimmt ein Rezept von A-&von Arzt (Interviewerin: Mhm (bejahend)), erstmal man bringt diese Rezept hierher. Dann, die machen Kostenvoranschlag zum Krankenkasse, das dauert paar Wochen (Interviewerin: Mhm (bejahend)) und dann, wenn das genehmigt, dann die schicken Prothese zum Reparatur (Interviewerin: Mhm (bejahend)). Das dauert auch paar Woche (Teilnehmer 1: Ja.). #01:26:54-1#

Interviewerin: Also ist man einfach lange ohne und hat das Problem ganz lang? #01:26:56-0#

Teilnehmer 5: Ja. #01:26:57-7#

Interviewerin: Ja. Wie ist das bei Ihnen? (wendet sich an Teilnehmer 9) #01:26:57-4#

Teilnehmer 9: Bei mir ist es so, dass ich die::, wenn ich die Prothese nicht trage, ich habe dadurch, weil ich ja sie-&fast sieben Jahre ohne gelaufen bin, hab ich natürlich Hilfsmittel Zuhause. #01:27:07-1#

Interviewerin: Mhm (bejahend) #01:27:07-1#

Teilnehmer 9: Und- #01:27:09-4#

Interviewerin: Welche zum Beispiel, wenn ich da kurz- #01:27:10-0#

Teilnehmer 9: ‘N Einhänderbrettchen oder für die Küche dann, zum Schälen mit einer Hand und solche Sachen dann eben, die ich dann eben nutze dafür. (Interviewerin: Mhm (bejahend)). Wenn ich dann mit beiden Händen nicht kann. #01:27:18-9#

Interviewerin: Können Sie das nochmal ein bisschen ausführen, außer dem Brettchen, was es da noch für Hilfsmittel gibt? #01:27:22-8#

Teilnehmer 9: Also ‘n Einhänderbrett, das ist so ‘n Küchenhilfsmittel eigentlich, wo man Kartoffeln mit schält, ode::r, dann aber&dann in Tablettform und äh kann man irgendwas einspannen (Interviewerin: Mhm (bejahend)), ob das Brot ist morgens zum Schneiden oder Brötchen, das kann man alles einspannen, in diesem Küchenhilfsgerät und das nutz ich dann. #01:27:42-4#

Interviewerin: Und gibt’s noch andere Sachen? Sie haben gesagt, da&noch andere Dinge, die Sie da als Hilfe benutzten, was gibt’s da noch, was Sie dafür verwenden,

wenn die Prothese grade nicht da ist? #01:27:51-1#

Teilnehmer 9: Wenn die Prothese&leider hab‘ ich das noch nicht so oft gehabt. #01:27:53-9#

Interviewerin: Ok. #01:27:53-9#

Teilnehmer 9: Und wenn ich dann, ich komme eigentlich ohne Prothese besser klar als mit Prothese (Interviewerin: Mhm (bejahend)). Weil ich das ja fünf Jahre lang geübt habe (Interviewerin: Mhm (bejahend)), dass es auch ohne geht. #01:28:07-4#

Interviewerin: Ja, das heißt es ist gar nicht so ein großes Verlustding oder das Gefühl das Leben ist dann aufgeschmissen, sondern ähm es gibt Hilfsmittel und Sie kommen dann einfach- #01:28:19-8#

Teilnehmer 9: Ich komme damit im Moment noch besser zurecht. #01:28:21-4#

Interviewerin: Mhm (bejahend), ja, ok. Mh jetzt haben wir viel eben über gute Sachen gesprochen, über schlechte Sachen an der Prothese. Mich würde interessieren, wenn Sie sich jetzt einmal vorstellen, dass Sie in der Position sind zu entwickeln, was würden Sie verändern an jetzt speziell Ihrer Prothese die sie nutzen, dass sie für Sie ideal wird? Also das&ich weiß, dass sich da jetzt manche Aussagen doppeln werden, weil wir einfach ähm schon vorher ‘n bisschen darüber gesprochen haben, aber um das jetzt nochmal auf den Punkt zu bringen: Was müsste man konkret verändern, vor allem vielleicht an Funktionen, damit die ideal wird, damit die die perfekte Prothese ist? #01:29:12-0#

Teilnehmer 9: Erstmal muss man den Prototyp kennenlernen. #01:29:16-8#

Interviewerin: Nee, jetzt- #01:29:16-8#

Teilnehmer 3: Nee. Was man machen dürfte, Herr (Name von Teilnehmer 9 aus Datenschutzgründen ausgelassen) wenn man&wenn man selber entscheiden dürfte wie’s aussehen soll. #01:29:23-6#

Teilnehmer 9: Ok. #01:29:21-9#

Interviewerin: An Ihrer-. Also nicht jetzt an der die hier entwickelt wird, sondern- #01:29:24-1#

Teilnehmer 9: Nein&nein&nein&nein&nein. Das- #01:29:25-5#

Interviewerin: Genau, sondern die, die Sie benutzen. #01:29:27-5#

Teilnehmerin 4: Ich würd‘ so einen nehmen (zeigt auf die Prothese von Teilnehmer 2). (Interviewerin: Mhm (bejahend)) Von (Name eines Prothesenherstellers aus Datenschutzgründen ausgelassen), weil die stabile Finger hat (Interviewerin: Mhm (bejahend)), a::ber die Finger dürften sich ruhig bewegen, damit man auch mal feine und grobe Sachen machen könnte. #01:29:38-7#

Interviewerin: Das heißt Sie würden jetzt gar nicht die nehmen, die Sie eigentlich

tragen, sondern- #01:29:41-6#

Teilnehmerin 4: Nö::, die ist mir zu, zu lasch. #01:29:43-1#

Interviewerin: Mhm (bejahend) #01:29:44-1#

Teilnehmerin 4: Mit den kann ich kein Messer richtig halten, mit der andern konnt‘ ich das auch festklemmen, Messer, schneiden, alles. Das kann ich mit der hier nicht. Da knicken die Finger weg und alles. #01:29:55-1#

Interviewerin: Was heißt: „Und alles“? Also woran liegt das, die Finger knicken weg, was macht das noch aus? #01:30:00-4#

Teilnehmerin 4: (..??) #01:30:02-7#

Teilnehmer 3: Zu wenig Kraft, die’s einfach-. Also ‘ne Touch Bionic-Hand hat einfach nicht genügend Kraft (Interviewerin: Mhm (bejahend)), um etwas, egal was eigentlich, so festzuhalten, dass Sie äh&dass Sie es grade wenn es feine Griffe sind, die Kraft äh also Präzisionsgriff der Kraft erfordert, dass kann ‘ne Touch Bionics-Hand nicht, da dreht sich der Daumen zur Seite weg, also bei meiner war das so. Der Daumen dreht sich zur Seite weg, oder aber die Finger gehen unter der Krafteinwirkung kaputt also (Teilnehmerin 4: Schiebt sich hier zwischen.) als meine ganz neu war- (Teilnehmerin 4: Wenn Sie das Messer nehmen und schneiden wollen, schiebt sich das Messer hier zwischen, aber-) #01:30:37-3#

Interviewerin: Das heißt die Finger können sich zwar bewegen, aber bringen nicht viel, weil einfach die Kraft nicht da ist? #01:30:39-9#

Teilnehmerin 4: Genau. #01:30:43-0#

Teilnehmer 3: Genau, das trifft’s ziemlich gut, ja. #01:30:45-9#

Teilnehmerin 4: So in der Form seiner (..??). (Interviewerin: Mhm (bejahend)) Und was ich auch schon bei der hier bemängelt hatte damals, äh, dass die zu langsam ist. (Interviewerin: Mhm (bejahend)) Wenn man jetzt im Haushalt was macht, oder egal was, man muss immer auf die Gestentechnik abwarten. (Interviewerin: Mhm (bejahend)) Bis der Finger gezuckt hat (lachend) und das dauert zu lange. (Interviewerin: Mhm (bejahend)) Und das hatte ich dann bemängelt und äh ja dann kam ‘n neues Update auf mein (..?) und dann wurde das so ‘n bisschen schneller gemacht (Interviewerin: Mhm (bejahend)) und ich hab’s jetzt auf die schnellste Stufe, aber es ist mir noch zu langsam. #01:31:31-9#

Interviewerin: Das heißt einfach die quasi Reaktionsgeschwindigkeit? #01:31:33-7#

Teilnehmerin 4: Jo. #01:31:36-7#

Interviewerin: Mhm (bejahend) #01:31:36-7#

Teilnehmerin 4: Also das könnte ruhig schneller sein. Ich möchte nicht immer warten, bis der Finger gezuckt hat. Oder ich hab‘ nur diese vier Steuerungen (Interviewerin: Mhm (bejahend)). #01:31:49-0#

Interviewerin: Und bei den anderen? #01:31:49-7#

Teilnehmer 3: Es sollte mechanisch stabil sein. (Interviewerin: Mhm (bejahend)) Es darf ruhig technisch aussehen (Interviewerin: Mhm (bejahend)), es sollte ‘n adaptiven Griff können (nimmt ein Glas und zeigt, was er mit einem adaptiven Griff meint), es sollte ‘n feinen Griff können und mehr braucht’s eigentlich gar nicht. Ich finde nicht, dass die Vielfalt der Funktionen, ich muss nicht so machen können (zeigt einen Griff mit den Fingern). (Teilnehmerin 4: Mhm (bejahend)) Das ist zwar witzig, aber ist nicht wirklich nötig. Und äh, es darf ruhig technisch aussehen, es muss Umwelteinflüssen widerstehen können, also es muss nass werden dürfen (Interviewerin: Mhm (bejahend)), es muss ähm mechanische Kräfte aushalten können, ohne kaputt zu gehen, zum Beispiel radeln, Auto fahren, ‘ne Schubkarre schieben, ‘n Besen halten (Teilnehmerin 4: Schwere Tasche tragen.) ohne dass&ohne dass man Angst haben muss, also ohne, dass man Angst haben muss der Hand Schaden zuzufügen. (Interviewerin: Mhm (bejahend)). Und (kurze Pause) ich glaube, es muss sich, wenn es irgendwie geht, das haben sie hier schon relativ gut gemacht, die haben ja hier Federn eingebaut, dann sind, wenn man von außen dagegen kommt, dann ist es so, dass die Hand nachgibt. Aber wenn Sie äh&Sie äh die Finger sind trotzdem steinhart. Und ich könnte mir vorstellen, dass man irgendwie&dass der Griff weicher wird. Aus i-&also, dass man, dass&dass der Griff feinfühliger und weicher wird. (Interviewerin: Mhm (bejahend)) Wäre eine Möglichkeit, die andere Möglichkeit ist, und das find ich ‘n ziemlich guter zweiter, der macht was ganz Einfaches aber der macht das total zuverlässig und immer in gleicher Weise (Interviewerin: Mhm (bejahend)). Das ist z-&und ich glaube das ist ‘n riesen Vorteil, also. #01:33:07-1#

Interviewerin: Das heißt wir sind wieder einfach an dem Punkt, dass es viel wichtiger ist, dass es robust ist, dass es zuverlässig ist und dafür eben weniger Funktionen, aber die halt einfach so wie’s soll und in jeder Situation? #01:33:17-4#

Teilnehmer 3: Genau so ist es. (Interviewerin: Mhm (bejahend)) #01:33:19-1#

Interviewerin: Ok. #01:33:19-9#

Teilnehmerin 4: Die hat auch die Funktion, dass sie wegknickt. Wenn ich aus- und anschalte, ‘ne? Also jetzt ist ‘se fest, jetzt schalt ich ‘se mal aus- #01:33:26-5#

Teilnehmer 3: Also, wir wollen w-&ich glaube- #01:33:29-1#

Teilnehmerin 4: Ich kann&und das hat mich schon mal gestört. #01:33:30-9#

Teilnehmer 3: Ich glaube es ist wichtiger, dass das was da ist wirklich funktioniert (Interviewerin: Mhm (bejahend)), als das viele technische Gimmicks da sind. #01:33:34-7#

Teilnehmerin 4: Ja. #01:33:36-2#

Interviewerin: Mhm (bejahend). Und bei Ihnen, Herr (Name von Teilnehmer 5 aus Datenschutzgründen ausgelassen)? #01:33:38-8#

Teilnehmer 5: Soll eine Prothese sein wie die Roboter. Beispiel zwei Teile, hier ist ein Teile, soll man immer tragen. Dann soll man von hier abmachen, wenn man schlafen geht. (Teilnehmer 3: Mhm (bejahend)). Und dann (kurze Pause) wenn wir gucken die Filme, dann die haben so:: viele Möglichkeit. Die machen alles. Aber äh ok wir sind zufrieden, oder ich bin zufrieden. Aber Hauptsache (kurze Pause) äh, das ist noch ganz neue Technologie (zeigt auf die Prothesen der anderen), äh ich habe zwei Woche das probiert, (Interviewerin: Mhm (bejahend)) am Ende habe ich gesagt: „Nein, das nicht für mich.". Weil wirklich kann man nicht so viel machen, so teuer und kann man fast (Teilnehmerin 4: Mhm (bejahend)) weniger als das (zeigt auf seine Prothese) machen, 'ne? #01:34:31-1#

Interviewerin: Ganz kurz: Können Sie das nochmal genauer erklären? Was können Sie damit nicht machen? Wenn wir das einfach nochmal kurz sagen. Was kann Ihrer Meinung nach die Hand, die Sie ausprobiert haben, was konnte die nicht, was Ihre jetzt kann? #01:34:50-8#

Teilnehmer 5: Äh mit dieser Hand (Interviewerin: Ja.), ich habe immer mich so äh ohne Prothese gefühlt (Interviewerin: Mhm (bejahend)). Weil was ich nicht machen will, das macht selber, manche Sachen (Interviewerin: Mhm (bejahend)). Und wenn ich will was machen, dann muss ich immer äh mehr Belastung zu mein äh Neffen hier&oder Muskel immer und dann hab' ich immer Schmerzen (Interviewerin: Mhm (bejahend)) und ich musste das abmachen. Äh weniger Bewegung, aber Hauptsache, man soll sich sicher fühlen (Interviewerin: Mhm (bejahend)). Ich fühle mich mit dieser Prothese sicher (zeigt auf seine Prothese). Mit die andre Hand, hab ich auch (Name eines Orthopädietechnikers aus Datenschutzgründen ausgelassen) gesagt, weil die haben hier vor ich gefragt haben, die habe als Computer angemacht. Die haben gefragt: „Herr (Name von Teilnehmer 5 aus Datenschutzgründen ausgelassen) haben Sie jeden Tag diese Prothese genutzt?". Hab' ich gesagt: „Nein. Insgesamt in zwei Woche", habe ich gesagt, „zehn Stunde." Die haben gesagt: „Wie?". Hab' ich gesagt:"Ja, das hat das, das, das.", hab ich die Filme gezeigt. „Nein, das ist unglaublich!". Ist das so? Mit das kann man noch mehr machen, äh was bedeutet, wenn ich kann mit das mehr machen, dann ich fühle mich noch sicher. #01:36:07-7#

Interviewerin: Ja. Das heißt aber auch, dass so ein Punkt „die Sicherheit", wichtig ist. Haben Sie auch gesagt (wendet sich an Teilnehmerin 4) mit dem, jemand kann sich einhacken, kann mich steuern, dass das auch einfach ähm 'ne Rolle spielt, dass man sich- #01:36:18-6#

Teilnehmerin 4: Das ist schon ganz gro:ß. #01:36:16-8#

Teilnehmer 5: Und schnell geht kaputt. Das so:: schnell geht kaputt. (Interviewerin: Mhm (bejahend)) Weiß nicht. #01:36:24-1#

Teilnehmer 9: Also ich hab' zu wenig Erfahrungswerte damit, mit anderen Prothesen. (Interviewerin: Mhm (bejahend)) Hab überhaupt keine andere Möglichkeit gehabt als diese aus-&auszuprobieren (Interviewerin: Mhm (bejahend)), bis heute. Und deswegen kann ich darüber auch nichts sagen. #01:36:35-0#

Interviewerin: Ja. Aber so v-&von dem Gebrauch den Sie haben? Sie haben zum Beispiel diese Schmerzen geäußert, ist da irgendwas, wo Sie sagen würden, wenn

ich da was an der Prothese ändern könnte-? #01:36:53-4#

Teilnehmer 9: Ich kann’s Ihnen nicht sagen, die äh die Beuger- und Strecksehne, die sorgen ja dafür, dass ich die Bewegung mache (Interviewerin: Mhm (bejahend)) und die krampfen andauernd. Ich hab mit dem TENS Gerät gearbeitet, fünf Jahre lang, (Interviewerin: Mhm (bejahend)) um dieses noch aufrecht zu erhalten, weil sonst würd ich gar nichts mehr (..?), sonst wär ich gar nicht mehr in Frage gekommen für ‘ne Prothese. (Interviewerin: Mhm (bejahend)) U:nd ich kann leider nichts dazu sagen. #01:37:20-7#

Interviewerin: Aber zum Beispiel ganz zu Beginn haben Sie gesagt, dass für Sie ein großer Punkt auch das Gewicht ist. #01:37:28-7#

Teilnehmer 9: Ja. #01:37:28-7#

Interviewerin: Das heißt, wenn Sie da sowas verändern würden, wäre das was wo Sie sagen würden, das&das wär‘ für mich wichtig als Veränderung? #01:37:35-6#

Teilnehmer 9: Als erstes muss sie erstmal optimal sitzen (Interviewerin: Mhm (bejahend)). Das ist bei mir auch noch nicht so 100%ig, die wackelt und die rutscht mir runter andauernd u:nd das sind immer noch so Faktoren, die noch ausgebessert werden müssten bei mir. #01:37:55-0#

Interviewerin: Also die Faktoren, damit meinen Sie den Sitz? #01:37:58-0#

Teilnehmer 9: Ja. #01:37:57-2#

Interviewerin: Mhm (bejahend) #01:38:00-4#

Teilnehmer 9: Ja, auf jeden Fall und (kurze Pause) äh ich kann auch&ich kann überhaupt nichts dazu sagen, was andere Prothesen machen, oder ob ‘se besser sind, oder schlechter. #01:38:06-7#

Interviewerin: Mhm (bejahend), aber wir wollen jetzt gar nicht mal auf diese anderen Prothesen eingehen, sondern eben einfach auf Ihre. Das was Sie, weil Sie sagen ja trotzdem, auch wenn Sie immer wieder mal Pause machen müssen, nutzen Sie die. Das heißt, Sie haben mit Ihrer Prothese einen Erfahrungswert. Und was ist da einfach an Ihrer Prothese, dass Sie sagen, das was ich im Gebrauch mit meiner Prothese erfahren habe, da gibt’s Dinge, die ich gerne verändern würde. So wie Sie sagen der Sitz oder dieses Gewicht. Gibt’s da noch irgendwas, wo Sie sagen, das würde ich mir wünschen, wenn ich ‘n Wunsch frei hätte, den ich an der Prothese verändern würde, was&äh welcher wäre das? #01:38:32-5#

Teilnehmer 9: (seufzt) Da kann ich leider nichts zu sagen. #01:38:36-0#

Interviewerin: Ok, ja kein Problem, ja. #01:38:39-6#

Teilnehmer 5: Was ich bei Ihnen gesehen habe (wendet sich an Teilnehmer 9), die Schaft sitzt nicht richtig so. #01:38:43-4#

Teilnehmer 9: Mhm (bejahend) #01:38:43-4#

Teilnehmer 5: Äh das meine Meinung. Seit wie viel Jahre ich trage das immer und dann hab ich auch so viel äh wie ich gesagt habe, dann ich frage, warum ist das so und dann äh mit (..?) hab ich jemand kennengelernt er hat von über WhatsApp wir haben mit Kamera uns, er hat mir gezeigt das und das Bruder, so und so. Und dann ich habe gesagt: „Ja, aber guck mal, das gibt Möglichkeiten das und des.“ Wenn Prothese sitzt richtig (Teilnehmer 9: Mhm (bejahend)), dann das ist sofort 50% (Teilnehmer 9: Besser.) besser. Aber die Problem ist (Interviewerin: Mhm (bejahend)): (Name eines Prothesenherstellers aus Datenschutzgründen ausgelassen) (kurze Pause) damals war die ältere Leute da, ‘93, ‘94, bis zum 2000. Man fährt einmal hin, die machen ein äh Gipsabdruck, zweite Mal fährt man hin (Interviewerin: Mhm (bejahend)), die probieren das, dritte Mal, man kriegt die Prothese. Oder wenn man hat Möglichkeit da zu Hotel bleiben, in zwei Tage ein 100%ige Prothese (Interviewerin: Mhm (bejahend)). Jetzt die sind alle Jugendliche, man fährt ein Jahr lang dahin (Interviewerin: Mhm (bejahend)), wenn man Erfahrung hat von Prothese. Wenn jemand neu, die erste Prothese ist, man ist immer zufrieden. Gott sei Dank, ich habe eine Prothese. (Interviewerin: Mhm (bejahend)) Dann, die sind zufrieden. Äh aber ich, ich nicht. Weil ich weiß, wie soll das sitzen, (Interviewerin: Mhm (bejahend)) wie soll ich das bewegen, wie soll ich das benutzen. Vor vier Jahre, ich habe ein Jahr lang 30 oder 40 Mal nach (Name eines Prothesenherstellers aus Datenschutzgründen ausgelassen) gefahren (Interviewerin: Mhm (bejahend)) oder 50 Mal, ich weiß das nicht mehr jetzt. #01:40:21-7#

Begleitung Teilnehmer 5: Sie haben die Prothese falsch gegossen, das war das Problem. #01:40:23-8#

Teilnehmer 5: Und dann, äh- #01:40:27-4#

Teilnehmerin 4: Warum haben Sie denn das nicht hier gemacht? #01:40:30-2#

Teilnehmer 5: Bitte? #01:40:30-2#

Teilnehmerin 4: Warum lassen Sie das nicht hier machen? #01:40:33-0#

Teilnehmer 5: Äh (kurze Pause) bleibt das alles hier ‘ne? #01:40:37-2#

Interviewerin: Ja! Natürlich! #01:40:36-3#

Teilnehmer 5: Am Anfang, die&(Name eines Orthopädietechnikers aus Datenschutzgründen ausgelassen) oder weiß nicht. Er war die, er machte die Handprothese. #01:40:47-8#

Teilnehmerin 4: Mhm (bejahend) #01:40:47-8#

Teilnehmer 5: Und dann äh ich brauchte eine neue Schaft, das und dies. Dann ich komme hierher, er macht das, Probeschaft und dann ich sage: „Ja, hier drückt, hier das so.“. Er versucht bisschen mit den spielen, warm machen, das machen, des machen. Und dann am Ende er hatte keine Lust mehr. #01:41:20-1#

Teilnehmerin 4: Mh. #01:41:20-1#

Teilnehmer 5: Mit mir. (kurze Pause) #01:41:26-4#

Teilnehmerin 4: Ich hatte ihn auch anfangs. (lachend) #01:41:29-3#

Teilnehmer 5: Solche Leute äh passen normalerweise solche Arbeit nicht. #01:41:35-6#

Teilnehmerin 4: Richtig. #01:41:38-5#

Teilnehmer 5: Wenn man ein Arbeit macht, Beispiel ich bin ein Reinigungskraft, ich arbeite als Reinigungskraft, ich sage, wenn ich verkaufe Gold, dann ich muss mich gewöhnen zum diese Arbeit (Teilnehmerin 4: Mhm (bejahend)). Wenn ich muss putzen, dann ich muss das ganz herzlich machen. (Teilnehmerin 4: Mhm (bejahend)) Was ich nicht herzlich mach, das funktioniert nicht. (Teilnehmerin 4: Mhm (bejahend)) Solche Leute sollen Geduld haben zu uns und bisschen Respekt (Teilnehmerin 4: Mhm (bejahend)). Weil äh wir spielen nicht. Wenn drückt (Teilnehmerin 4: Ja, ich weiß.) nach einem Tag sieht man das und wenn man zeigt: „Ja guck mal, Herr Kollege, hier drückt das!". „Öh!". Oder wenn das funktioniert nicht gut, wenn das nicht dich&wo soll das, Beispiel: Zwei Zentimeter hoch, zwei Zentimeter unter, die sollen bisschen Geduld haben, ok? Die sollen nochmal messen, das und dies. (Teilnehmerin 4: Mhm (bejahend)) Ok das ist auch nicht, äh leichte Arbeit, ich weiß das, ich versteh das. Aber wenn, Beispiel, ich sage nur ein Beispiel: Vor drei Jahre (Name eines Prothesenherstellers aus Datenschutzgründen ausgelassen), die haben mir eine Prothese gemacht äh dann, seitdem, hab das weggeschmissen Zuhause, diese Schaft. Alles neu, alle Sachen neu, aber hab' ich nicht ein Tag getragen, weil äh, hat so viele Druckstellen und dann, die Elektrode ist nicht richtig wo soll sein (Teilnehmerin 4: Mhm (bejahend)). Beispiel: Ich will nichts machen, aber das geht alleine auf (Teilnehmerin 4: Mhm (bejahend)). Weil das&die haben das nicht gut gemacht. Seitdem dann war ich hier, (Name eines Orthopädietechnikers aus Datenschutzgründen ausgelassen) wollte was machen, ohne hinschicken. Er sagte: „Ich darf nicht, weil die haben das gemacht, ich muss hinschicken.". Dann ich habe nochmal hingefahren, am Ende hab gesagt, ich trage wieder die alte Prothese, das&das alte Schaft, Hand ist neu, ok? (Teilnehmerin 4: Mhm (bejahend)) Äh das seit vier Jahre, oder drei Jahre seitdem, aber die Schaft, das ganz nagelneu, aber ich&Beispiel ich kann das nicht tragen (Teilnehmerin 4: Mhm (bejahend)). #01:43:54-4#

Interviewerin: Das heißt 'n ganz wichtiger Punkt ist einfach auch die&das, dass es bequem ist, dass es richtig sitzt, aber (Teilnehmer 5: Dass die Fachmann soll sein.). Ok, aber auch die Kommunikation mit dem Techniker? #01:44:02-7#

Teilnehmer 5: Richtig, richtig. #01:44:04-2#

Teilnehmerin 4: Mhm (bejahend) #01:44:04-2#

Teilnehmer 5: Das- #01:44:06-3#

Interviewerin: Und das können die andern so unterschreiben? #01:44:07-1#

Teilnehmer 3: Ich hab' das anders gemacht. Ich hab' einfach&also ich hab das Ding einfach gnadenlos getragen, so lange bis wirklich Schaden da war (Interviewerin:

Mhm (bejahend)) und wenn Sie dann kommen und haben wirklich so ‘ne Blase auf dem&auf dem Stumpf, dann gibt’s keine Diskussion mehr darüber, das was geändert werden muss, ‘ne? Das müssen Sie einfach durchleiden (Interviewerin: Mhm (bejahend)). ‘Ne? Wenn Sie&wenn Sie ‘ne rote Druckstelle haben, dann sagt der: „Ach ich mach das mal ‘n bisschen weich.“ und „Das wird schon werden.“ und wenn Sie kommen und der Arm sieht wirklich schlimm aus und die&S-&ich hab Fotos gemacht davon und dann einfach diese&bin dann einfach mit diesem kaputten Stumpf da angekommen und hab gesagt: „So, das sind die Stellen wo’s drückt.“, dann wurde neu gebaut. (Interviewerin: Mhm (bejahend)) Weil das äh letztlich, Sie müssen ja auch ‘ne vernünftige Argumentationsbasis haben (Interviewerin: Mhm (bejahend)) und dazu gehört, man muss ja auch&sich auch klar machen, die Krankenkasse gibt sehr viel Geld aus und die Leute die’s bauen, geben sich ‘ne Menge Mühe damit. Und ich glaube man muss sich auch ‘ne&muss sich dann entsprechend viel Mühe geben das auch auszuprobieren und dann auch bereit sein, dafür ‘n bisschen Schmerz zu leiden (Interviewerin: Mhm (bejahend)) und das hab ich halt immer durchgezogen und eigentlich bin ich damit zu sehr zufriedenstellenden Ergebnissen gekommen. Also in meinem Fall, den Prothesenschaft hier hat (Name eines Sanitätshauses aus Datenschutzgründen ausgelassen) gebaut und das ist&das hat einfach gut geklappt. Aber eben&ich glaube es ist ‘ne sehr deutliche Kommunikation nötig, man muss einfach sagen: „Da tut’s weh!“, man muss es vorzeigen können und dann geht das auch. (Interviewerin: Mhm (bejahend)) #01:45:19-4#

Teilnehmer 5: (Name eines Orthopädietechnikers aus Datenschutzgründen ausgelassen) hat viel Geduld. #01:45:21-0#

Teilnehmerin 4: Ja. #01:45:21-0#

Teilnehmer 5: Er hat so:: viel Geduld, wirklich. Egal wenn man (Teilnehmerin 4: Also der macht’s gut.)- #01:45:23-7#

Teilnehmer 3: Wobei, meine hat (Name eines Orthopädietechnikers aus Datenschutzgründen ausgelassen) gebaut, das eben von Anfang an. #01:45:25-6#

Teilnehmerin 4: Meine auch. #01:45:25-6#

Interviewerin: Ich würd‘ jetzt gerne noch als letzte Frage einmal reinbringen und zwar haben wir jetzt über ganz viele verschiedene Fähigkeiten gesprochen, was ja technisch noch nicht möglich ist, bei Ihren Prothesen ist, das, dass man irgend ‘ne Art von Feedback bekommt, also was sensorisches. Und da würde ich gern einmal wissen, inwiefern das wichtig ist? Wir haben jetzt oft einfach gesagt, das eigentlich gar nicht mehr Funktionen dazukommen müssten, Hauptsache die anderen funktionieren gut, aber wir hatten zum Beispiel in der letzten Runde, weil ich glaub die Frage ist einfach nicht immer einfach zu äh verstehen, hatten wir das Beispiel, dass einer der Teilnehmer gesagt hat: „Wenn ich in den Mantel steigen will und ich guck nach hinten, ich seh‘ ja gar nicht, ich weiß ja gar nicht, ich fühl nicht, wo das Loch ist, wo mein Arm rein soll.“ und eben, dass er sich bei sowas wünschen würde, dass er da&dass die Prothese ihm irgendein Gefühl, ein Signal zurückgibt: „Da ist jetzt Widerstand, da ist jetzt was.“. Das mal als Beispiel worauf ich hinauswill. Wie stehen Sie dazu? Ist Ihnen sowas-. Ähm soll ich die Frage nochmal erklären? Sie gucken mich gerade ‘n bisschen- #01:46:19-4#

Begleitung Teilnehmer 5: Ich äh, ich muss es übersetzen. #01:46:21-0#

Interviewerin: Ja. #01:46:23-4#

(Begleitung von Teilnehmer 5 übersetzt ihm die Frage.) #01:46:35-2#

(Teilnehmer 5 schüttelt den Kopf) #01:46:35-2#

Begleitung Teilnehmer 5: Das war ‘n klares Zeichen. #01:46:33-9#

Teilnehmer 5: Das- #01:46:37-7#

Begleitung Teilnehmer 5: Braucht man nicht. #01:46:37-7#

Teilnehmer 5: Das braucht man nicht. Das ein komische (lachend)- #01:46:42-4#

Teilnehmer 9: Komische Frage war das. #01:46:42-4#

Teilnehmer 5: Wirklich für mich das ist das so. #01:46:44-3#

Begleitung Teilnehmer 5: Nein, nee&nee. Äh die Erwartungshaltung. #01:46:50-5#

Teilnehmer 5: Was die Dame sagt, das und dies. Das gibt’s noch wichtigere Sachen. #01:46:54-0#

Begleitung Teilnehmer 5: Wie ziehst du denn die Jacke an? Jetzt überlege ich auch wenn du ‘n Mantel anziehst. Er zieht’s ja von vorne an. #01:46:59-3#

Teilnehmer 5: Nein einfach. #01:47:01-9#

Teilnehmer 9: Erst den Arm rein. #01:47:00-6#

Begleitung Teilnehmer 5: Erst den Arm rein. Also- #01:47:05-6#

Interviewerin: Ok aber ich- #01:47:07-6#

Teilnehmer 5: Beispiel so. #01:47:08-4#

Interviewerin: Ja. Aber ich möchte jetzt gar nicht bei dem Jackenbeispiel bleiben. Sondern, da gehört ja noch mehr dazu. Zum Beispiel, mh wenn Sie irgendwas greifen wollen, müssen Sie hingucken, weil Sie nicht fühlen können, mit der anderen Hand können Sie fühlen. Ich hab’s angefasst, ich weiß ich hab’s in der Hand. #01:47:28-5#

Teilnehmerin 4: Mhm (bejahend) #01:47:30-5#

Teilnehmer 9: Ja, ja. #01:47:28-6#

Interviewerin: Es geht ja auch darum, dass Sie&dass zum Beispiel wenn die Prothese Ihnen ‘n Signal gibt: „Jetzt ist da der Gegenstand“, dass ich das fühlen

kann. Ist sowas wichtig? Oder wie müsste sowas aussehen, damit es hilfreich ist? #01:47:43-8#

Teilnehmer 3: Ich glaube es ist extrem wichtig, ich wag nur nicht zu hoffen, dass das realisierbar ist. #01:47:50-7#

Interviewerin: Mhm (bejahend). Und können Sie das, ok es ist wichtig- #01:47:56-1#

Teilnehmer 3: Also wenn Sie sich diese Hand angucken (Interviewerin: Ja.), die hat ja nur fünf Finger und damit Sie damit was sinnvolles anfangen können, müssen Sie tatsächlich schauen, was macht der einz-&wo ist jeder einzelne Finger gerade (Interviewerin: Mhm (bejahend)) und dann müssen Sie sich noch merken, bei dieser Hand ist es so, Sie schalten&schalten das Griffmuster um, indem Sie&indem Sie den in die Endstellung bringen und äh und dann äh einmal geöffnet halten und dann kommt ‘n anderer Griff. Dann müssen Sie sich die Frequenz dieser Muster merken, dann haben Sie hier noch ‘n Knopf (Interviewerin: Mhm (bejahend)), damit können Sie umstellen, Sie können den Daumen zur Seite klappen und dann passieren wieder andere Dinge. Sie müssen erstens sich merken: Was hab‘ ich zuletzt getan? (Interviewerin: Mhm (bejahend)) Und zu-&als zweites, Sie müssen&und da ist das Ding, das nur eine&das ‘ne eindimensionale Bewegung macht, ganz vorne. (Interviewerin: Ja.) Da wissen Sie immer was los ist. (Interviewerin: Mhm (bejahend)) Und insofern ist das eigentlich, glaube ich, praktischer. Wenn Sie&wenn Sie ‘ne mehrgliederige Hand benutzen wollen, müssen Sie wissen, was wo ist und da ist ‘n Feedback natürlich ‘ne extrem wichtige Sache. Seh‘ ich&ich wag nur nicht zu hoffen, wie das geht, weil ich mir nicht vorstellen kann, wie man das realisieren will (Interviewerin: Ok.). #01:49:11-0#

Interviewerin: Ok, dann sind wir jetzt gerade an dem Punkt, dass wir sagen, es wäre wichtig, dass es das gibt. Können Sie sich&also wir sind jetzt einfach mal dabei, dass wir nicht gucken, wie ist das technisch lösbar, aber wenn Sie sich vorstellen, wie müsste sowas aussehen, was müsste ich wahrnehmen. Ist das ‘n Widerstand, ‘n Druck? Wo müsst‘ ich den wahrnehmen? Wo müsste der sitzen? Also einfach, dass&dass wir nochmal ‘n bisschen genauer definieren, wie das sein müsste, damit man das wahrnimmt? #01:49:35-4#

(kurze Pause) #01:49:37-3#

Teilnehmer 3: Na, ich überlege gerade, was man&was&was ich wahrnehmen könnte. (Interviewerin: Mhm (bejahend)) Also irgend ‘ne Form von Signal. Es ist eigentlich ziemlich egal, was das ist, das könnte irgend ‘ne Vibration sein (Interviewerin: Mhm (bejahend)) oder so, solange man was, wenn man was berührt, fängt das Ding an sich zu bewegen. Das könnte theoretisch auch äh nen&nen&nen auk-&nen&nen akustisches Signal sein (Interviewerin: Mhm (bejahend)) oder so, ‘ne? #01:49:58-8#

Teilnehmerin 4: Ich glaub, akustisch stört nach ‘ner Zeit. Also mich würde das nerven, wenn ich jetzt (Teilnehmer 3: Man muss das ausschalten können.)-. Ja:: aber wenn ich was im Haushalt mache und das ist immer akustisch, das nervt nach ‘ner Zeit (Interviewerin: Mhm (bejahend)). Das war anfangs mit der Prothese hier auch, dass&dass ich mich an dieses Geräusch, an den Sensoren und alles, also Motoren, ‘ne? #01:50:19-6#

Interviewerin: Und was könnten Sie sich dann vorstellen, wie das ‘n Signal gibt. Was wäre was, was nicht stört? #01:50:23-6#

Teilnehmerin 4: Also ich würde eher sagen, weiß ich nicht, vielleicht eher Licht, oder irgendwas? #01:50:31-6#

Interviewerin: Mhm (bejahend) #01:50:31-6#

Teilnehmerin 4: Also vielleicht- #01:50:33-0#

Teilnehmer 3: Das ist ja auch komisch, wenn die Hand immer anfängt zu leuchten, außerdem muss man dann wieder hingucken, um das zu sehen (lachend). #01:50:38-9#

Teilnehmerin 4: Ja es muss nicht hier, äh- #01:50:38-4#

Teilnehmer 9: Eingebaute Taschenlampe. #01:50:39-7#

Teilnehmerin 4: Äh- #01:50:39-7#

Teilnehmer 3: Nee, ich glaube irgendwie so ‘ne Art Vibration oder so, würde glaub ich helfen (Teilnehmerin 4: Irgendsowas, ja.). #01:50:41-3#

Interviewerin: Mhm (bejahend). Und in welchen Situationen müsste das da sein? Also Sie haben gerade gesagt: „Ich fass was an und es gibt mir ‘n Signal zurück.“. In welcher Situation könnte das noch oder zum Beispiel wenn Sie sagen: „Ich muss mir immer merken wie&wie stehen die Finger, welcher ist wie.“. #01:50:51-6#

Teilnehmer 3: Genau. Also man könnte das ja, man könnte ja zum Beispiel ‘n kodiertes Signal zurückgeben, der sagt sozusagen: „Das ist mein Betriebszustand gerade.“ (Interviewerin: Mhm (bejahend) So dass&dass man ohne hinzugucken weiß, was passieren wird, ‘ne? (Interviewerin: Ja.) Also ich&da muss man&also ich könnt mi-&man kann ja irgendwie zum Beispiel verschieden frequente Signale irgendwie durch Vibration über den Prothesenschaft auf den Arm ausüben. Die spürt man ja. (Interviewerin: Mhm (bejahend), ja.) (Teilnehmerin 4: Mhm (bejahend)) Und äh über die Frequenz mit der man diese Vibration sendet, könnte man zum Beispiel erstens Mal ‘n Feedback über die Kraft probieren (Interviewerin: Mhm (bejahend)). Das kann man ja frequenzabhängig machen. Es wird&‘n (Variometer?) im Segelflugzeug funktioniert so, ‘ne? Wenn’s gut steigt, wird der T-&das macht ‘n Ton, ‘n Piepton, ‘ne? Wenn‘s runtergeht, mach ich „Uaaaah“ und wenn’s anfängt zu steigen dann halt „didd&didd&didd&didd&didd“ und der&die Frequenz des Piepens und der Ton wird immer höher, je schneller man steigt (Interviewerin: Mhm (bejahend)). Also&und das benutzt man, um nicht mehr auf’s Instrumentenbrett gucken zu müssen, sondern nach draußen, um zu gucken: Was geht um mich rum vor. Und man nimmt sozusagen das&diese&ne&ne Bewegung, für die man eigentlich kein Organ hat, nämlich steigen oder sinken, über diese Tonhöhe wahr. (Interviewerin: Ja.) Das könnte man durch ‘ne Vibration nachbilden zum Beispiel. #01:51:50-9#

Interviewerin: Ja, und aber jetzt haben wir zum Beispiel die Kraft mit der die greift,

die Stellung der Finger oder der Betriebszustand (Teilnehmer 3: Mhm (bejahend)) ähm oder auch: Ich greif was und da ist ’n Gegenstand. Was ist denn am wichtigsten? Wo ist es denn am wichtigsten, dass ich jetzt, dass ich da ‘n Signal zurückbekomm? Vielleicht können wir- #01:52:02-7#

Teilnehmer 3: Ob ich noch zugreife und ob der Griff sich löst, das will ich. Also was mir am häufigsten passiert ist, ich trag irgendwas (kurze Pause) was Herr (Nam von Teilnehmer 5 aus Datenschutzgründen ausgelassen) auch gesagt hat, ich trag irgendwas und wenn ich sicher sein will, dass ich es nicht verliere, dann schalt ich die Hand aus. Ich mach das beim Fliegen so. Also ich fass den Gashebel an (Interviewerin: Mhm (bejahend)) und äh schalte dann die Hand aus, weil ich dann weiß, die ist sicher in der geschlossenen Position und ich hab‘ diesen Hebel immer in der Hand, wenn ich&weil ich ihn die ganze Zeit brauche. (Interviewerin: Mhm (bejahend)). Und wenn ich da sozusagen ne&ne (kurze Pause) egal was eigentlich. Also ob das jetzt Tasche oder Hebel oder sonst irgendwas ist (Interviewerin: Mhm (bejahend)), sozusagen ‘ne Information kriege: „Jo, dein Griff ist noch fest und sicher.“ (Interviewerin: Mhm (bejahend)), das wär mir eigentlich am wichtigsten. Weil dieses unbeabsichtigte Loslassen, äh das ist eigentlich, was mich am meisten stört (Interviewerin: Mhm (bejahend)). #01:52:49-5#

Interviewerin: Und wie ist das bei den anderen? #01:52:52-5#

Teilnehmer 9: Ich spinne jetzt erstmal rum. #01:52:54-1#

Interviewerin: Ja, gerne (lachend). #01:52:56-8#

Teilnehmer 9: Und zwar (kurze Pause) ich hab‘ grade an Implan-&Implantation gedacht. Dass man Elektroden implantiert in den Beuger oder Strecker (Interviewerin: Mhm (bejahend)). Da merkt man sofort, ob da ‘n Widerstand ist, oder nicht. #01:53:11-5#

Teilnehmer 3: Warum? #01:53:16-3#

(kurze Pause) #01:53:16-3#

Teilnehmer 9: Das merkt man einfach. Das merkt man in der rechten Hand, genau wie in der Linken. (Interviewerin: Mhm (bejahend)) Aber dazu müssen die ja vorhanden noch sein, ‘ne? Natürlich #01:53:27-0#

Teilnehmer 3: Also Sie stellen sich so ‘n Interface vor sozusagen, zwischen der&zwischen dem Tastsensor und der dann direkt an den Nerv angepflanzt wird? #01:53:35-8#

Teilnehmer 9: Mhm (bejahend) #01:53:36-8#

Teilnehmer 3: Ich hätte ‘n bisschen Furcht, in meinen Körper ‘n Loch reinmachen zu lassen und äh dann sozusagen, also mir ist ganz wichtig, dass es sozusagen ‘ne Grenze zwischen innen und außen gibt. Das ist ja auch ‘ne Infektionsm-&‘n möglicher Infektionsherd, ‘ne? #01:53:53-8#

Teilnehmer 9: Ja, ja, das ist klar. #01:53:54-2#

Teilnehmer 3: Wenn Sie&wenn jetzt irgendwo an irgend ‘ner Stelle ‘n Draht aus Ihnen rauskommt, dann sind Sie sowas wie ’n Stomaträger oder so (Interviewerin: Mhm (bejahend)). Ich halt das für nicht ganz ungefährlich sowas zu machen. #01:54:04-9#

Teilnehmer 9: Ich habe drei Wochen das gehabt. Es war draußen aus ’m Körper. Weil ich hab ‘n Schrittmacher implantiert gekriegt, erstmal drei Wochen außerhalb und dann wurde es im-&implantiert, logischerweise und Infektion war da zum Glück gar nicht. #01:54:21-2#

Interviewerin: Aber Herr (Name von Teilnehmer 9 aus Datenschutzgründen ausgelassen), um nochmal kurz bei dem Gedanken zu bleiben, den Sie hatten, wann&also war’s der schon oder ähm kommt da noch was dazu? #01:54:32-4#

Teilnehmer 9: Nee, ich glaube das war’s dann schon. #01:54:32-5#

Interviewerin: Mhm (bejahend), ok. Ja. #01:54:37-6#

Teilnehmer 9: Also dann muss ich in medizinischen Bereich. Aber das kann ich leider nicht. #01:54:41-1#

Interviewerin: Und aber eben von dem was ich wahrnehm‘, was ist da für Sie am wichtigsten? Wo würden Sie sich das am meisten wünschen, dass&dass es irgendwie Rückmeldung von dieser Prothese gibt? #01:54:52-8#

Teilnehmer 9: Wenn ich’s entscheiden könnte wo an der Prothese, dann sind’s die Fingerkuppen, also die Fingerspitzen, dann aber von der Prothese (Interviewerin: Mhm (bejahend)). Damit ich das Empfinden dann wiederhabe. #01:55:07-7#

Interviewerin: Also, dass es eher darum geht einfach ‘n Gefühl zurückzubekommen? Die Umgebung wahrzunehmen (Teilnehmerin 4: Mhm (bejahend))? #01:55:09-5#

Teilnehmer 9: Ja, ja. #01:55:15-2#

Interviewerin: Das wäre das Wichtigste? #01:55:15-9#

Teilnehmer 9: Das wäre für mich das Wichtigste. #01:55:17-7#

Teilnehmerin 4: Würd‘ ich auch sagen. #01:55:19-2#

Interviewerin: Mhm (bejahend) #01:55:20-3#

Teilnehmerin 4: Den oberen Teil von den Fingern. #01:55:21-5#

Interviewerin: Mhm (bejahend) #01:55:22-4#

Teilnehmerin 4: Da hat man Gefühl, Kraft (Interviewerin: Mhm (bejahend)). #01:55:28-3#

Interviewerin: Und Herr (Name von Teilnehmer 5 aus Datenschutzgründen

ausgelassen)? Weil Sie haben jetzt zuerst so reagiert: „Nein, das braucht man nicht.“. Ähm, wenn Sie jetzt aber ähm noch gehört haben, was es noch dazu gibt, also&dass es nicht nur dieser Mantel ist, sondern eben auch Kraft spüren, oder spüren was macht die Prothese gerade. Ist es trotzdem nicht wichtig in Ihren Augen? #01:55:41-9#

(Teilnehmer 5 schüttelt den Kopf) #01:55:44-5#

Interviewerin: Nee, ist nicht wichtig? #01:55:46-0#

Teilnehmer 5: Nicht. Wie ich gesagt habe, wichtig ist für mich kräftig und dann äh, dass wenn zu ist, dann zu bleiben (Interviewerin: Ja.). Wenn auf ist, dann kann ein Signal das kann sein, ein Signal kommt „piep piep“, ich weiß dann, geht auf, aber Hauptsache ich halte das unter Kontrolle (Interviewerin: Mhm (bejahend)) immer. Äh und Gefühl, Gefühl stört uns noch mehr. Wenn hier, ‘tschuldigung (an die anderen Teilnehmer gerichtet), aber wenn wir haben jetzt hier Gefühl (Interviewerin: Mhm (bejahend)) und dann wir merken hier. (Teilnehmerin 4: Mhm (bejahend)) Weißt du, ich überlege jetzt. (Teilnehmerin 4: Mhm (bejahend)). #01:56:15-5#

Interviewerin: Nee und Sie dürfen auch ‘ne andere Meinung haben. Also Sie müssen das&jeder empfindet das anders. #01:56:19-3#

Teilnehmer 5: Mhh. Wenn ich fühle jetzt hier Gefühl (Interviewerin: Ja.) und dann das Signal geht zu meinem Kopf, Beispiel. Weil irgendwo soll ich das fühlen. (Interviewerin: Mhm (bejahend)) Äh, ich überlege dann, stört das mich? (Interviewerin: Würde Sie das stören?) Weiß nicht, ich überlege nur (Interviewerin: Mhm (bejahend)), jetzt weil solche Meinung hab ich bis jetzt nicht überlegt. Beispiel: Sowieso ich habe eine Prothese und dann äh ich muss immer denken jetzt ich muss aufmachen, wieder zu, auf, zu. Ich merke das sowieso hier (Interviewerin: Mhm (bejahend)). Wenn die andre Prothese, die sind <u>noch</u> schwerer. (Interviewerin: Mhm (bejahend)) Dann muss man denken: „Ja, ja, was sollt ich jetzt machen.“ So, so. #01:57:01-1#

Teilnehmerin 4: Mhm (bejahend), Gestentechnik. #01:56:58-6#

Teilnehmer 3: Wirklich das, ich habe zwei Wochen, dann hab‘ ich gesagt, ich hat&ich&ich war fertig mit dieser Hand (Teilnehmerin 4: Mhm (bejahend)). Dann weniger Belastung, sag ich. (Interviewerin: Mhm (bejahend)) Weiß nicht, weil Gefühl hab ich bis jetzt (Interviewerin: Mhm (bejahend))- #01:57:16-1#

Interviewerin: Ok. Dann sind&das war ja schon tatsächlich die letzte Frage. Ich würd‘ jetzt nochmal ganz kurz zusammenfassen. Wir haben über Nachteile und Vorteile gesprochen, über Situationen in denen Sie die Prothese weglassen, auch Situationen in denen es einfach stört, was passiert, wenn sie kaputt geht, was Sie verändern würden. Gibt’s noch was, was nicht gefragt wurde, was Ihnen aber am Herzen liegt? Oder gibt’s noch irgendwas, wo Sie jetzt sagen, nochmal drüber nachgedacht, hätt‘ ich das gerne noch ergänzt und hatte nicht die Gelegenheit das zu sagen? #01:57:39-6#

Teilnehmer 3: Also ich hab‘ soeben, als&als Herr (Name von Teilnehmer 5 aus Datenschutzgründen ausgelassen) gesagt hat: “Ich will gar nichts verändern“, hab

ich so überlegt: Eigentlich, wenn ich's mir frei aussuchen dürfte, (Interviewerin: Mhm (bejahend)) dann würd ich etwas haben wollen, das einer richtigen Hand so nah wie irgendwie möglich kommt (Interviewerin: Mhm (bejahend)). Man darf ja mal träumen und spinnen (Interviewerin: Klar.) Weil bisher&wir reden ja immer nur&wir haben ja viel geredet über das was ist und das was zurzeit möglich ist (Interviewerin: Mhm (bejahend)) und das was wir haben. Aber ich glaube, wenn ich's mir wirklich frei aussuchen dürfte, hätte ich wirklich etwas, das einer richtigen Hand so nah wie möglich kommt (Interviewerin: Mhm (bejahend)). Es darf ruhig technisch sein, aber&aber das wär' dann schon irgendwie 'nen Gefühl über die Lage der einzelnen Finger im Raum, wie viel Finger das sind, da kann man drüber diskutieren, es soll einigermaßen hübsch aussehen, es soll 'ne Rückmeldung geben, was es grade tut und ob's noch festhält und wie groß die Kraft ist, die ich ausübe (Interviewerin: Mhm (bejahend)). Und insofern, das gibt's noch nicht (Interviewerin: Mhm (bejahend)), also ich seh' das nirgendwo, bisher. Und aber ich fänd's schön, wenn's das gäbe. #01:59:10-2#

Interviewerin: Ja. Und wenn wir jetzt mal gucken, was von den Themen, die besprochen wurden, so das Wichtigste ist, was Sie einfach weitergeben möchten, fällt Ihnen dazu was ein? Wenn wir das einfach runterbrechen auf so das relevanteste, was wir heute Abend besprochen haben, das wichtigste Thema heute? #01:59:32-0#

Teilnehmer 3: Bezogen auf Hände, oder überhaupt? #01:59:37-6#

Interviewerin: Ganz überhaupt, einfach so&vielleicht das Resümee des heutigen Abends für Sie. Wenn Sie sagen, das liegt mir besonders am Herzen, dass sowas einfach bedacht wird oder, dass&dass&das war für mich das Wichtigste, was angesprochen wurde. #01:59:56-0#

Teilnehmer 9: Ich hab' noch 'ne Anmerkung. #01:59:58-4#

Interviewerin: Ja! #01:59:56-8#

Teilnehmer 9: Und zwar, es wäre doch schön, wenn die betroffenen Patienten wirklich Hände ausprobieren könnten (Interviewerin: Mhm (bejahend)). Weil ich konnte nämlich nur eine&eine ausprobieren und das was diese I-Limb Quantum und hätt ich die Möglichkeit gehabt (Name eines Prothesenherstellers aus Datenschutzgründen ausgelassen)-Hand auszuprobieren und so, dann hätt ich ja die Möglichkeit gehabt zu entscheiden. #02:00:16-3#

Interviewerin: Mhm (bejahend), ja. #02:00:16-8#

Teilnehmer 9: U::nd diese Möglichkeit wurde mir leider nicht geboten. Also das ist für mich&wäre für mich mit das Wichtigste. #02:00:23-2#

Interviewerin: Also, dass ich mich nicht gleich für 'ne Prothese entscheiden muss, sondern sagen kann, ich teste die und guck ob sie- #02:00:27-3#

Teilnehmer 9: Ja, welche denn am besten für mich ist. #02:00:29-9#

Interviewerin: Mhm (bejahend), ja. #02:00:30-5#

Teilnehmer 9: Das scheint mir sehr, sehr wichtig zu sein. Also für das was ich im Alltag auch mache. #02:00:32-9#

Teilnehmer 5: Richtig. #02:00:35-4#

Teilnehmer 3: Na, was ich noch ganz wichtig fände ist, äh meine Beobachtung ist so ‘n bisschen, äh das kam&das leuchtete hier vorhin auch mal kurz auf, ‘ne (Name eines Prothesenherstellers aus Datenschutzgründen ausgelassen) Prothese kostet in der Türkei etwa die Hälfte dessen, was man in Deutschland dafür bezahlt (Interviewerin: Mhm (bejahend)). Das hat ja irgendwie auch ‘n bisschen was mit dem politischen System zu tun und ich glaube, was ich ganz wichtig fände ist, dass äh die Verfügbarkeit, also einfach auch die Verfügbarkeit von Medizintechnik oder solchen Hilfsmitteln wie Händen. So ‘n bisschen aus diesem Bereich der&der staatlich regulierten äh äh des staatlich regulierten Gesundheitssystems, in dem sich die Anbieter ja, wie soll ich das sagen, durchaus ‘ne goldene Nase verdienen und&und äh manchmal auch auf Kosten der Verfügbarkeit für die Leute, die die Dinge, die Sie anbieten brauchen (Interviewerin: Mhm (bejahend)). Und, was ich mir wünschen würde ist, dass einfach die äh (kurze Pause) wie soll ich das sagen, dass die&dass man zu Lösungen kommt, die preiswerter sind, wo vielleicht der&wo vielleicht auch das zugelassene Hilfsmittel oder Arzneimittel nicht ganz so hoch gehängt wird, sondern wo einfach ‘ne Verfügbarkeit, ne&ne&vielleicht auch ‘ne Möglichkeit für die Leute die betroffen sind, der Veränderung. Also was Herr (Name von Teilnehmer 9 aus Datenschutzgründen ausgelassen) sagte: „Ich hab‘ nur eine Prothese ausprobiert und kenn gar nichts anderes.“, dass man einfach ‘ne? Das ist, wenn so ‘ne Pro-&wenn so ‘ne Hand 20 oder 25 000 Euro kostet (Interviewerin: Mhm (bejahend)), ist es natürlich schwer jemandem zu sagen, jetzt probierste erst die und dann die. (Interviewerin: Ja.) Wobei da hätt‘ ich auch gute Ideen zu, weil wenn Sie&wenn Sie ‘ne Hand ‘ne Weile gehabt haben, fünf Jahre oder sechs oder sieben Jahr, normalerweise zahlt die Krankenkasse dann ‘ne neue Hand (Interviewerin: Mhm (bejahend)). Die Hand gehört ja trotzdem, die Sie da zur Verfügung gestellt bekommen, die gehört ja gar nicht Ihnen, die gehört der Krankenkasse. Was ich nicht verstehe ist, warum die die Hände nicht wieder einsammeln und sagen: „Wir tun die jetzt alle in einen Pool und geben die den Leuten, die erstmal probieren wollen.“ (Interviewerin: Mhm (bejahend)) Denn da würde man ‘ne Menge Kosten mit sparen. #02:02:09-4#

Interviewerin: Ja, also auch einfach dieser Kostenfaktor und dieses, dass mir das Hilfsmittel dann auch wirklich zur Verfügung steht? #02:02:15-1#

Teilnehmer 3: Dass es zur Verfügung steht, also zum Beispiel, bei Vielen liegen zwei Hände in der Schublade (Interviewerin: Mhm (bejahend)). Und jemand anders hat nur eine und seine ist kaputt und er hat keine (Interviewerin: Ja.). Das ist doch nicht in Ordnung? #02:02:26-2#

Interviewerin: Ja. #02:02:24-4#

Teilnehmer 3: Aber wenn Sie sagen, ich würd‘ meine Hand jetzt gerne wieder abliefern, dann gibt’s keinen Ort, wo Sie das tun können. Also wenn&ich hab‘ den (Name eines Orthopädietechnikers aus Datenschutzgründen ausgelassen) gefragt, was mach ich jetzt mit der I-limb-Hand, die würd ich Ihnen jetzt gerne wiedergeben,

ich brauch Sie nicht. Da sagt der: „Ja, es gibt keinen Ort dafür, wo man die hintun kann.“ Und niemand würde was damit anfange, wenn ich sie (Name eines Sanitätshauses aus Datenschutzgründen ausgelassen) oder der Krankenkasse wiedergeben würde, was ich für ‘ne Verschwendung halte. (Interviewerin: Mhm (bejahend)) Weil die Leute, die einfach nur probieren wollen, können das doch auch mit ‘ner gebrauchten Hand tun. #02:02:54-4#

Teilnehmer 5: Richtig. #02:02:52-7#

Teilnehmerin 4: Bin auch der Meinung. Hab‘ ich auch schon mal angesprochen, aber- #02:02:56-7#

Teilnehmer 3: Nee, das interessiert keinen, weil es auch keinen&da ist kein Geld dran zu verdienen. Das macht die Sache nur billiger. #02:03:01-5#

Teilnehmerin 4: Nee, auch nicht in dritte Länder oder sowas. #02:03:04-0#

Teilnehmer 5: Wenn jemand stirbt, dann soll das zurückgeben. Wenn das und dies, dann (kurze Pause) jeder hat Möglichkeit, (Interviewerin: Mhm (bejahend)) äh Beispiel ok, wir tragen Prothese seit schon lange, aber Beispiel, wenn jemand, weil passiert das jeden Tag etwas Amputation (Interviewerin: Mhm (bejahend)) und dann, kann man des Schaft machen, dann kann man die Prothese prob-&Hand probieren (Teilnehmerin 4: Mhm (bejahend)). Äh diese Hand für mich ist gut, diese Hand nicht gut (Teilnehmer 3: Genau.). Oh, ok, ich kann das gut benutzen (Interviewerin: Ja.), dann jeder hat, was sie braucht (Teilnehmerin 4: Mhm (bejahend)). #02:03:35-4#

Interviewerin: Ok, gibt’s sonst irgendwas, was jemand ähm hinzufügen möchte? #02:03:40-4#

(kurze Pause) #02:03:41-7#

Interviewerin: Ok. Dann- #02:03:42-2#

Teilnehmer 3: Wann kriegen wir zu sehen was Sie entwickeln? #02:03:44-2#

Interviewerin: Ja, jetzt gleich. Dann würd‘ ich sagen, dass das Gespräch jetzt erstmal beendet ist, wir machen die Mikrofone aus.
