# Supplementary material for: What do users and their aiding professionals want from future devices in upper limb prosthetics? A focus group study
Source: PLoS One. 2023 Dec 29;18(12):e0295516. doi: 10.1371/journal.pone.0295516 (PMC10756510; doi:10.1371/journal.pone.0295516)
Supplement: S1 Appendix — (ZIP) [file pone.0295516.s001.zip › FocusGroup_Transcripts/FGP4.pdf]

Interviewerin: Ähm und wenn ich die erste Frage gleich einmal stell‘, würd ich bitten, dass die erste einfach der Reihe nach beantwortet wird und Sie einmal nochmal Name&Vorname, Nachname sagen, damit ich einfach die Stimme später leichter mit dem&zum Namen zuordnen kann. #00:00:13-1#

Alle Teilnehmer: Mhm (bejahend) #00:00:18-9#

Interviewerin: Genau, ich würd‘ gern zu Beginn von Ihnen wissen, wenn Sie als Fachmann eine Person treffen, die eine Armprothese trägt, was Ihnen da als erstes auffällt oder als was Sie da als erstes achten? #00:00:39-7#

Teilnehmer 27: (Name von Teilnehmer 27 aus Datenschutzgründen ausgelassen), ich achte als erstes darauf, auf die Gesamterscheinung des Patienten, den Habitus, wie er steht, wie er sich bewegt, wie er sich verhält und da seh‘ ich sehr schnell: Ist das Hilfsmittel ‘n Teil seines Körpers, oder steht das irgendwie neben ihm, sag ich mal, ja? (Interviewerin: Mhm (bejahend)) Das ist für mich die wichtigste erste Information. #00:01:01-6#

Interviewerin: Mhm (bejahend), das heißt das Ziel, wenn ich die Prothese anpass‘, wenn jemand ‘ne Prothese bekommen soll, ist, dass sich das einfach zu ‘nem Gesamtbild zusammenfügt? #00:01:10-7#

Teilnehmer 27: In sein Körperbild integriert. #00:01:12-1#

Interviewerin: Mhm (bejahend), ja. #00:01:13-2#

Teilnehmer 28: (Name von Teilnehmer 28 aus Datenschutzgründen ausgelassen), (den meisten?) Patienten schau ich automatisch erstmal welche Seite (Interviewerin: Mhm (bejahend)) und dann schau ich auch auf’s Gesicht, um zu schauen oder zu erahnen, was steckt hinter dem Menschen dahinter. (Interviewerin: Mhm (bejahend)) #00:01:25-3#

Teilnehmer 29: Mein Name ist (Name aus Datenschutzgründen ausgelassen), ähm also ich schau mir sehr häufig auch die Vorversorgung an, soweit sie existiert und möchte gerne wissen, wie er damit zurechtgekommen ist, was war gut daran, was war nicht gut daran. Das gibt uns immer wieder sehr viel Feedback darüber, äh was besser werden muss (Interviewerin: Mhm (bejahend)), wie die Akzeptanz des Hilfsmittels allgemein ist und äh was wir vielleicht verbessern können. #00:01:48-0#

Interviewerin: Und wie merkt man zum Beispiel wenn’s nicht akzeptiert ist oder wenn’s nicht passt? Fällt einem das auf, wenn man so ‘ne Person trifft, gleich von Anfang an? #00:01:59-0#

Teilnehmer 29: Ähm am ehesten sieht man’s daran, wie ähm, wie gut jemand diese Prothese handhaben kann (Interviewerin: Mhm (bejahend)), wie sehr er damit etwas demonstrieren kann, was er dann macht oder wie sehr er damit&äh wie sehr er beschreiben kann, was er für Tätigkeiten macht, welche Vorteile sie ihm bringt. (Interviewerin: Mhm (bejahend)) (kurze Pause) Ja. Das ist&genau&also ich würd‘ ihm also, würd‘ ihn fragen wo sind die Vorteile und würd‘ mir vielleicht durchaus was demonstrieren lassen von ihm, was er damit machen kann (Interviewerin: Ja.). Ja. #00:02:35-9#

Interviewerin: Und bei den anderen noch? Wenn&also eben, wenn ich so ‘ne Person treff‘, gibt’s irgendwas, wo mir gleich zu Beginn auffällt, dass da irgendwas nicht passt oder dass&dass die vielleicht nicht so funktioniert, wie sie funktionieren sollte, gleich auf den ersten Blick, was man dann im Hinterkopf hat, wenn man so jemanden versorgt? #00:02:48-0#

Teilnehmer 27: Für mich ist es das, wie er sich bewegt, wie er sich gestikuliert, ob er seine Prothese hinterm Rücken versteckt oder ob er mit der Hand vielleicht spricht (Interviewerin: Mhm (bejahend)) oder ja, sie einfach locker sich verhält mit dem Hilfsmittel oder ob er’s irgendwie versteckt oder verkrampft ist. (Interviewerin: Ja.) #00:03:06-5#

Interviewerin: Gibt’s noch was zuzufügen, oder- #00:03:06-3#

Teilnehmer 28: Ich denk ja, man&man schaut sich an ja in welchem Gemütszustand ist halt der Patient auch (Interviewerin: Mhm (bejahend)) und ist es (kurze Pause) ob man ‘n Lächeln ins Gesicht zaubern kann oder ob er (kurze Pause) irgendwelche Einschränkungen hat (Interviewerin: Mhm (bejahend)), dass man auch ‘n bisschen weiß vielleicht, wie man ihn angeht und wo man ansetzen muss. #00:03:26-0#

Interviewerin: Ja. Ok. Ähm dann würd‘ ich als nächstes bitten, dass Sie sich einmal die Situation vorstellen, dass jemand eben hierher kommt und ‘ne Versorgung möchte und zwar explizit ‘ne moderne Prothese. Was für Eigenschaften muss so jemand mitbringen, als Patient einfach damit so ‘ne Versorgung möglich ist, oder welche Voraussetzungen müssen einfach gegeben sein, damit ‘ne Versorgung möglich ist? #00:03:49-8# #00:03:46-8#

(kurze Pause) #00:03:53-2#

Teilnehmer 27: Fang ich an? #00:03:54-7#

Interviewerin: Mhm (bejahend), ja. #00:03:54-4#

Teilnehmer 27: Sie meinen mit einer bionischen Hand? Also multiartikulierenden Hand oder irgendeiner nicht Standard (Name eines Prothesenherstellers aus Datenschutzgründen ausgelassen) Hand, (Interviewerin: Mhm (bejahend), ja.) hab‘ ich richtig verstanden? #00:04:01-8#

Interviewerin: Zum Beispiel. Also einfach, dass wir, weiß nicht, es gibt ja die verschiedenen Arten, es gibt auch die ganz passiven, dass wir einfach möglichst auf die gucken, die so der neueste Stand der Dinge sind. #00:04:10-7#

Protokollant: State of the Art. #00:04:09-0#

Teilnehmer 27: Ok, ja. Ok. Wenn es jemand ist, der noch gar keine Prothese hat, dann hinterfragen wir erstmal sämtliche mh sozialen Hintergründe, Beruf, Hobby, Freizeit (Interviewerin: Mhm (bejahend)), was ganz klar meines Erachtens die Richtung angibt, welches Hilfsmittel auch für ihn geeignet ist. Das ist nicht Landwirt, der ‘ne Michelangelo-Hand oder ‘ne Bebionic-Hand haben will (Interviewerin: Mhm (bejahend)), äh allenfalls als Zusatz. Bei Kindern ist nochmal ganz anders, da sind

wir sehr eingeschränkt (Interviewerin: Mhm (bejahend)), da ist des Passteil&die Passteilmöglichkeit natürlich sehr gering, aber ich geh jetzt mal vom Erwachsenen aus, dann hinterfragen wir eben das, wo soll das Einsatzgebiet sein. (Interviewerin: Mhm (bejahend)) Ähm natürlich müssen wir auch klären, wir haben jetzt gerade ‘n Kind aus Russland, ist die Nachhaltigkeit auch gegeben, sowohl finanziell auch als ähm von der Struktur, von der Infrastruktur äh kann das Hilfsmittel gewartet werden, gepflegt werden etc.? (räuspert sich) Ähm dann ist die Vorversorgung wichtig. Kommt jemand aus ‘ner passiven Versorgung, hat noch nie ‘ne Funktionshand gehabt. Wir haben jetzt grade ‘n Beispiel: Eine Kundin hat mit 16 eine Myoprothese bekommen, sie ist jetzt äh Anfang 60 äh lange her, die Technik war lange nicht so, sie hat nur eine Erinnerung (Interviewerin: Mhm (bejahend)), dass sie von ihrem Fahrradlenker nicht mehr loskam mit der Hand, ja? Sie wollte&will jetzt eine funktionelle Hand, weil sie Überlasterscheinung in der haltenden Hand hat (Interviewerin: Mhm (bejahend)), was auch oft ‘n Grund ist von Kunden, äh die spät versorgt wurden oder lange Zeit gar nicht funktionell versorgt wurden. Hat aber ganz klar im Kopf, es darf auf keinen Fall eine (Name eines Prothesenherstellers aus Datenschutzgründen ausgelassen) Hand sein. Obwohl ich aus meiner Erfahrung sag, das was sie beschrieben hat, ist sie eigentlich die Anwenderin (Interviewerin: Mhm (bejahend)) für genau diese robuste Hand. Gartenarbeit und&und&und. Und dann wird’s schwierig, den Patienten, ich sag mal gefühlvoll dahin zu bringen, was eigentlich wirklich Sinn macht. Dann machen wir das gern über Testversorgung, wo wir sagen, wir beantragen eine Testversorgung, dann kann der Kunde einfach drei Hände verschiedenster Art einfach durchprobieren und sich dann wirklich ‘n realistisches, eigenes Bild machen. (Interviewerin: Mhm (bejahend)) Aber es ist meines Erachtens die Kunst in der Beratung zu sagen: Aus dem Wissen was wir haben und aus der Erfahrung heraus wissen wir, was für diesen Typen Menschen das Richtige ist, mit dem Fragezeichen vielleicht sind’s auch zwei Prothesen, was wir auch oft machen: Eine funktionelle Hand und für Sport und Freizeit auch eine so genannte Habitus-Hand. Was ich für ‘ne sehr gute Ergänzung sehe (Interviewerin: Mhm (bejahend)) oder eigentlich die beste Versorgung sehe, weil wir halt nie, genauso wie ‘n paar Schuhe, (Interviewerin: Mhm (bejahend)) nicht zum Sport unsre High Heels tragen und umgekehrt (Interviewerin: Mhm (bejahend), ja.) ähm und da geht meine Beratung dann hin. Aus meiner Erfahrung und aus dem, wie sich der Patient darstellt (Interviewerin: Mhm (bejahend)). Mit allem vielleicht an schlechten Erfahrungen, die er gesammelt hat oder auch an positiven oder was auch immer. #00:07:05-7#

Interviewerin: Und können Sie das noch einmal ausbauen, wenn Sie sagen eben, jetzt den&nicht für den Landwirt, sondern für irgendwen anders, dass Sie da einfach nochmal sagen, für wen sich so ‘ne Hand eignet, dass wir das noch ‘n bisschen genauer aufdröseln? #00:07:17-9#

Teilnehmer 27: Was ganz wichtig ist und das hatten wir gestern, äh wir hatten einen bilateral Amputierten, äh der ganz klar sagt: Zuverlässigkeit, Zuverlässigkeit, Zuverlässigkeit. (Interviewerin: Mhm (bejahend)) Klar, natürlich für jemanden der beide Hände verloren hat, ist es was anderes, als wenn jemand eine Hand verloren hat. Aber (kurze Pause) auch j-&wir haben Kunden, die berufstätig sind, die ‘ne Prothese 20h am Tag nutzen, die einfach sagen: „Ganz wichtig, dass ich mich darauf verlassen kann.“ (Interviewerin: Ja.) Und das ist bei vielen modernen Entwicklungen manchmal noch nicht so. Die werden schön dargestellt, aber wir wissen in der Praxis, dass die fragil und anfällig sind, zu anfällig für so ein Hilfsmittel

und das finde ich, ist ein ganz ganz wichtiger Punkt. (Interviewerin: Mhm (bejahend)) #00:07:59-1#

Teilnehmer 28: Ich denke auch, viele kommen heutzutage her mit gewissen Vorinformationen, die Sie sich über's Internet geholt haben und manche haben dann schon (kurze Pause) 'n Bild projiziert was: „Ah ich möchte unbedingt die Hand" (Interviewerin: Mhm (bejahend)) und sind aber auch schwierig abzuholen, zu sagen: „Jetzt lösen wir uns mal von dem Gedanken, weil für des wofür Sie die Prothese möchten, ist es vielleicht nicht ganz (kurze Pause) die erste Lösung." Aber, was ich heutzutage merke, es sind viele halt echt mit vielen Internetvorinformationen vorbelastet, auf 'ne Art und Weise und nicht mehr ganz frei ähm (kurze Pause) das macht's dann bisschen herausfordernder in der Versorgung, (Interviewerin: Mhm (bejahend)) die zufriedenzustellen. #00:08:41-1#

Interviewerin: Und wenn wir da nochmal einfach genauer drauf gucken eben, die&dass Sie sagen, die kommen mit dem fertigen Bild und die wollen irgendwas haben, wo Sie jetzt einfach als Fachmann sagen: „Das passt gar nicht zu dem, was Sie damit machen wollen." Welche Prothese passt denn zu was? Also wie kann ich das denn zu irgendwelchen Aufgaben, die die Prothese erfüllen soll zuordnen, welche Prothese zu was passt? #00:09:00-2#

Teilnehmer 28: Ich denk da muss man schon sagen, dass (Name von Teilnehmer 27 aus Datenschutzgründen ausgelassen) da die meiste (Interviewerin: Mhm (bejahend)) Erfahrung hat und ich glaub mit den Jahren kriegt man auch 'ne Art von Gespür, Bauchgefühl (Interviewerin: Mhm (bejahend), ja.) und (kurze Pause) kombiniert mit Anzahl der&der Reklamationen vielleicht, wo man dann auch sagt, also die Hand ist dafür nicht geeignet, (Interviewerin: Mhm (bejahend)), dafür, die Hand ist überhaupt nicht gut, ich glaub das kriegt man mit der Erfahrung, die&die Routine oder das Gespür dafür. #00:09:38-2#

Interviewerin: Ja. #00:09:40-8#

Teilnehmer 29: Also meine Erfahrungen mit äh mit diesen Patienten, die sich explizit eine sehr moderne Hand wünschen ist, dass die Erwartung viel zu hoch ist. Das hat ähm (Name von Teilnehmer 28 aus Datenschutzgründen ausgelassen) gerade eben schon gesagt (Interviewerin: Mhm (bejahend)), ähm und's gibt selbst Hersteller, die zugeben müssen, desto größer ist hinterher auch die Enttäuschung und desto schlechter ist hinterher die Akzeptanz des Hilfsmittels. Und grade durch's Internet ähm da kursieren so viele Entwicklungen, die getätigt werden, ähm so viele Hände, die niemals marktreif entwickelt werden, aber die unheimlich hohe Erwartungen ähm bei den Patienten wecken und äh das ist eigentlich ein sehr großes Problem. Die&es ist auch schwierig teilweise manche Kunden, die sehr technikaffin sind, ähm dann von&von ihren Erwartungen runterzubringen und vielleicht in 'ne andere Richtung zu lenken (Interviewerin: Mhm (bejahend)). Mein Eindruck, wenn's darum geht in welcher Situation die beste Hand ähm ist eigentlich so von systematischen Tests, die wir hier gemacht haben, zeigt sich eigentlich immer wieder, dass die H-&neuen Händen funktionell besser sind, die kommen eigentlich besser an (Interviewerin: Mhm (bejahend)). Es sei denn jemand ist seit vielen Jahren eingeschworen auf die alten, klassischen Hände und weiß einfach wie die laufen und äh kennt alle Erscheinungen wie zum Beispiel das Motorengeräusch (Interviewerin: Mhm (bejahend)) und kann damit die Griffkraft einschätzen, die diese Hände aufbauen.

Aber ansonsten sind die neuen Hände eigentlich ähm ähm funktionell überlegen. Aber der größte Nachteil ist eigentlich die Fragilität dieser Hände (Interviewerin: Mhm (bejahend)), also die mangelnde Zuverlässigkeit. Für Auslandspatienten wär' das eigentlich fast 'n Ausschlusskriterium (Interviewerin: Mhm (bejahend)), grade wenn die finanziellen Mittel nicht so da sind und für aktive ähm Anwender hier aus dem Inland, ist es auch sehr schwierig ähm, wenn die Belastungen hoch sind durch Sport oder durch Arbeitseinsatz, ähm kann man diese Hände auch nicht einsetzen, auch wenn sie vielleicht funktionell überlegen sein möchten (Interviewerin: Mhm (bejahend)), sein mögen. Ähm der Sprung was Funktionalität anbetrifft ist nicht immer so groß wie erwartet obendrein. Der ist zwar da, aber nicht so groß, dass man die Nachteile der mangelnden Zuverlässigkeit in Kauf nehmen sollte. (Interviewerin: Mhm (bejahend)) 'Ne? Es gibt also Dinge wie zum Beispiel Griffgeschwindigkeit, die einfach bei den alten Händen besser ist. Und das ist auch 'n Faktor den&den&der den meisten Patienten wichtig ist. Ähm (kurze Pause) - #00:12:18-1#

Teilnehmer 28: Auch mim Handschuh. Zum Beispiel im Moment is' es so, dass die drei führenden Händehersteller, auf allen Bildern wird die Hand promotet ohne Handschuh und dann kommt das böse Erwachen, wenn da 'n Handschuh draufkommt. #00:12:29-3#

Interviewerin: Mhm (bejahend), weil der nicht so ist, wie man ihn haben möchte? #00:12:32-7#

Teilnehmer 28: Optisch nicht. (Interviewerin: Mhm (bejahend)) (Also?) man hat Potential zum Verbessern, aber auch von der Funktion nehmen sie wahnsinnig viel weg. (Interviewerin: Mhm (bejahend)) Des is' auch auf 'ne Art 'ne Irreführung, grade was&was die Wirtschaft mit den Kunden macht 'n bisschen. (Interviewerin: Mhm (bejahend)) Weil diese Erwartungen mit denen sie kommen aus 'm Internet- #00:12:52-0#

Interviewerin: Halt einfach nicht zur Realität passen? #00:12:52-7#

Teilnehmer 28: (kurze Pause) Nicht, nicht ganz. #00:12:52-3#

Interviewerin: Mhm (bejahend). Ich würd' gern einmal nochmal einfach was die Voraussetzungen für die Versorgung angeht, im Hinblick einfach auf das Physiologische. Eben was muss der Patient auch einfach an körperlichen Gegebenheiten mitbringen, damit ich überhaupt so 'ne Prothese anpassen kann? Wenn wir darüber vielleicht nochmal&darauf dann eingehen könnten? #00:13:08-4#

Teilnehmer 29: Also ich f-&natürlich ähm erstmal die Co-Kontraktionsfähigkeit oder diese Triggersignale (Interviewerin: Mhm (bejahend)) für 'ne moderne Hand müssen gegeben werden können. (Interviewerin: Mhm (bejahend)) Das erfordert bei vielen Patienten die das nicht gewohnt sind solche Signale zu geben, erfordert das erstmal viel Übung oder einiges an Übung (Interviewerin: Mhm (bejahend)). Das ist aber ganz unterschiedlich, wie schnell die Patienten das verstehen (Interviewerin: Ja.). Co-Kontraktion kann manchmal 'n großes Problem sein bei manchen Patienten, bei anderen wiederum geht's sofort, oder ziemlich schnell (Interviewerin: Mhm (bejahend)). Ähm und das muss&müsste man halt vorher versuchen herauszufinden. Wenn die zum Beispiel mal 'n Drehansatz eingebaut hatten, der über Co-Kontraktion bedient wurde, dann weiß man ähm, dass es auch möglich sein

wird dieses Signal für ‘ne Hand zu nehmen (Interviewerin: Ja.), soweit es nicht schon ähm verwendet wird für den Dreheinsatz, der nach wie vor drinbleiben soll. Ähm ja ich w-&ich würde da als erstes ‘n Myotest machen oder halt beziehungsweise wenn man keinen Myotest macht, aber ‘ne Hand zur Verfügung hat (Interviewerin: Mhm (bejahend)), dann das konkret testen. (Interviewerin: Mhm (bejahend)) Und da ist es wahrscheinlich auch wichtig ähm, dass man ‘ne gewisse Übungsphase hat für den Patienten mit einer Testhand. Weil, der kann das nicht innerhalb von einer Stunde hier bei uns in der Werkstatt herausfinden (Interviewerin: Mhm (bejahend)), sondern der müsste die Gelegenheit haben das länger zu testen (Interviewerin: Ja.). Das ist eine der&der, sag ich mal, körperlichen Voraussetzungen, die ‘n Patient haben muss. ‘Ne gute und zuverlässig zu erbringende Myosignale auch ähm proportionale Signale, die man gut geben kann, aber auch vor allem diese Signale, diese Triggersignale, müssen gegeben werden können. (Interviewerin: Mhm (bejahend)). #00:14:42-7#

Interviewerin: Gibt’s da noch was hinzuzufügen? #00:14:44-0#

Teilnehmer 28: Ich denk die Anatomie ist halt wichtig, ob man sieht, der Stumpf da Vernarbungen sind, Verspannungen, Kontrakturen und das man Punkte findet, ob Neurome da sind, extreme Sensibilität im Stumpf (Interviewerin: Mhm (bejahend)) und ob man Punkte findet, wo man myosele-&elektrische Signale überhaupt finden kann (Interviewerin: Mhm (bejahend)), die der Patient umsetzt. Ich mein mit ‘ner Software kann man immer noch ja entscheiden eine Elektrode, zwei Elektroden, aber dass man dort Signale findet und schaut, wie ist die Haut&Hautoberfläche. (Interviewerin: Mhm (bejahend)) #00:15:18-6#

Teilnehmer 29: Ich könnte mir vorstellen, die Stumpflänge hat auch ‘ne gewisse Relevanz weil (kurze Pause) diese <u>neuen</u> Hände, (..?) oder wie immer man sie nennen möchte, sind auch immer ‘n Stückchen schwerer und letztendlich kommt es bei so ‘ner&grade bei kurzen Stümpfen auf jedes Gramm an und da macht sich jedes Gramm bemerkbar (Interviewerin: Mhm (bejahend)) oder es macht sich schon bemerkbar, ob Prothesenkomponenten dicht am Stumpf oder weit distal ähm am langen Hebel der Prothese angebracht werden und das muss man den Patienten zumindest klar machen ähm dass diese Hände zwar ‘n funktionellen Vorteil bringen können, aber das sie auch durchaus schwerer sind und das muss man&muss der halt auch für sich herausfinden (Interviewerin: Mhm (bejahend)) im Test. Wir können das selbst nicht ohne weiteres sagen, nur in dem wir uns den Stumpf anschauen. Das ist auch ‘ne Gefühlssache des Patienten, ob er mit diesem&diesem Mehr an Gewicht zurechtkommt. (Interviewerin: Ja.) #00:16:10-0#

Teilnehmer 27: Und auch ‘ne Frage des Schaftes. (Interviewerin: Mhm (bejahend)) Wenn der Prothesenschaft entsprechend gearbeitet ist, wird das Gewicht nicht so empfunden. Wir haben jetzt grade ein Mädchen da, die hat ‘n zweieinhalb Zentimeter Unterarm und hat ‘n Hebel von 40 Zentimeter da dran, will ‘ne Bebionic-Hand haben, die redet nicht ein Mal darüber, dass die zu schwer ist (Interviewerin: Mhm (bejahend)), weil die Funktionalität in ihrem Kopf überwiegt, kompensiert sie das (Interviewerin: Mhm (bejahend)). Sie hat nebenher noch eine Habitusprothese und hat da über Tag mal die Möglichkeit zu wechseln. Aber das sehen wir so grade die Jugendlichen, wenn die so eine Bebionic-Hand kriegen oder so ‘ne multiartikulierende Hand, dann haben die einen Motivationsschub (Interviewerin: Mhm (bejahend)), weil die Hand <u>cool</u> ist. Nichts anderes. Es geht um

cool sein, ja? W-&wie in unsrer Außenwelt auch. Und da kriegen die Motivationshebel auf einmal. Die (Name eines Prothesenherstellers aus Datenschutzgründen ausgelassen) Hand wurde kaum getragen aber die multiartikulierende Hand ist auf einmal das Tollste und die Eltern berichten mir: Früher Tragezeit vier Stunden, heute am liebsten noch durch die Nacht. (Interviewerin: Mhm (bejahend)) Ja? Und jetzt greifen Sie das mal, greifen Sie mal diese psychologische Charakteristik, die dem gehandicapten Menschen auf einmal durch den Kopf schießt, wenn er sagt: „Ich hab‘ zwei Hände, da bewegen sich zehn Finger.“ (Interviewerin: Ja.) Das hat viel mit Psychologie zu tun und nicht mit Quantifizierung von Hebellängen oder sonst irgendwas. Das ist ‘n riesen breites&breiter Fächer, der da von allen Seiten touchiert wird. #00:17:29-7#

Interviewerin: Mhm (bejahend). Das heißt aber eben, dass manche Nachteile, wo man eigentlich sagen könnte, da wird der Tragekomfort oder die Funktionalität negativ beeinflusst, dass die oft sogar aufzuheben sind, wenn man den Patienten eben an der richtigen Stelle abholt? #00:17:41-5#

Teilnehmer 27: Genau, genau. Das seh‘ ich immer bei den Kindern, wenn wir Kinder haben, 12 Monate, die sagen mir nicht: „(Name von Teilnehmer 27 aus Datenschutzgründen ausgelassen), ich möchte gerne ‘ne Myohand haben“. Da sag ich zu den Mitarbeitern, die müssen Elektroden am Arm haben und die müssen diese Hand bewegen und dann hast du drei Minuten Zeit denen kurz in die Augen zu gucken, ob da was flackert oder nicht. (Interviewerin: Mhm (bejahend)) Und wenn’s da flackert, tragen die die. (Interviewerin: Mhm (bejahend)) Ja? Und wenn die noch mit 12 Monaten sagen: „Oh, da ist noch ’n Vogel.“, oder sonst irgendwas abgelenkt sind, dann weiß ich, dass es zu früh ist. (Interviewerin: Ja.) Und mir sagen ganz oft Patienten, die zu uns kommen: „Ich hab‘ das Gefühl, dass ich hier befragt werde, dass ich hier verstanden werde, dass man hier Interesse hat.“ und auf einmal öffnen die sich und erzählen einem Dinge, die ihnen für ihre Prothese wichtig ist, die sie, obwohl sie vorher bei fünf andren Firmen waren, noch nie erzählt haben. Das sind einfach so die weichen Faktoren, sag ich mal, die, wie wenn wir zum Arzt gehen und sagen, wir haben ‘n Gefühl, dass wir uns dem anvertrauen, dann äh können wir darüber reden. Und dann kriegt man viele Informationen. (Interviewerin: Mhm (bejahend)) #00:18:50-5#

Interviewerin: Ähm gibt’s zu diesen Voraussetzungen noch irgendwas, was jemand hinzufügen möchte? Was ihm noch einfällt? (kurze Pause) Wo man auch nochmal drauf achtet, jetzt neben diesem&dem psychologischen Aspekt, neben diesem Aspekt mit den Elektroden, irgendwas was Ihnen einfällt, wo Sie noch drauf achten? #00:19:06-8#

Teilnehmer 27: Stabilität natürlich auch, also auch soziale Stabilität. (Interviewerin: Mhm (bejahend)) Wenn jemand, ich sag mal, drogenabhängig, Alkoholiker oder sonst irgendwas ist, ist der sicher nicht der richtige Kandidat für so ‘ne mulitartikulierende Hand, ganz klar. (Interviewerin: Ja.) Ähm ansonsten auch das was (Name von Teilnehmer 3 aus Datenschutzgründen ausgelassen) gesagt hat, mit Triggersignal oder Co-Kontraktionsfähigkeit, meines Erachtens alles erlernbar. (Interviewerin: Mhm (bejahend)) Es gibt keinen, der das nicht kann und deshalb nicht geeignet ist. Es gibt vielleicht einen der länger braucht, aber nicht einen der’s nicht kann und wenn die Leute von den Hilfsmitteln fasziniert sind, entwickeln die eine Energie, da sich auch reinzu-&das sehen wir immer an den Doppelamputierten, ja?

(Interviewerin: Mhm (bejahend)) Wenn einem beide Hände fehlen, dann sehen wir wirklich, was mit so 'ner Hand überhaupt m-&machbar ist. Das zeigt uns 'n einseitig Amputierter nie. (Interviewerin: Ja.) #00:19:55-7#

Interviewerin: Mhm (bejahend). Ok. #00:19:59-7#

Teilnehmer 28: Manchmal schaut man vielleicht doch auch, das hab' ich mal irgendwo gelernt und des find ich manchmal auch noch recht hilfreich, schaut ob (man?) in der Kurve, wo man den Patienten abholt. (Interviewerin: Mhm (bejahend)) Und&und manchmal ist es so bei Amputation, ist mein Eindruck, wenn man 'se so nach der Talsohle abholt, dann kommen auch wieder die Erfolge mit und dann ist es leichter sie zu guiden auf 'm Erf-&auf 'm Weg nach oben. Wenn noch jemand in einer Krise ist, dann kostet's sehr viel Aufwand (Interviewerin: Mhm (bejahend)) und man ist dann selber auch, also ich denk mir, bin auch 'n bisschen verunsichert, wie man jemand noch behandelt, der noch 'n bisschen auf 'm Weg nach unten ist (Interviewerin: Mhm (bejahend)) und ich denk mal (..?) so nach der Talsohle, geht's voran. (Interviewerin: Ja) #00:21:07-7#

Interviewerin: Ok. Ähm wenn wir uns jetzt vorstellen, dass der Patient der eben kam und jetzt gesagt hat: „Ich möchte gerne diese Versorgung haben.“, dass der die Prothese bekommen soll, dass Sie sagen, so wie der gerade ist, wie der hier steht, was der mitbringt, das passt, da kann man die Prothese anpassen und der den Schaft schon hat. Also der Schaft schon perfekt sitzt. Was gibt's dann noch äh für Probleme bei der Prothesenversorgung, die einfach häufig auftreten, bei der Anpassung, wo man sagt das ist was, was häufig einfach 'ne Schwierigkeit ist, die da im Weg steht, wo man dran arbeiten muss? #00:21:37-8#

Teilnehmer 27: Eigentlich keine. Könnt' ich jetzt nicht sagen. #00:21:42-8#

Teilnehmer 28: Kosmetisch- #00:21:43-6#

Teilnehmer 27: Also, wenn ich den Patienten gut informiert hab, den trainiert hab, mit dem Hilfsmittel (Interviewerin: Ja.), der den perfekten Schaft hat, wie Sie sagen, obwohl's den meines Erachtens nicht gibt, weil da sind wir noch lange nicht am Ende (Interviewerin: Ja, ok (lachend)) ähm dann mein ich, dass es da keine Schwierigkeiten gibt. (Interviewerin: Mhm (bejahend)) Dann wird der da reinwachsen, wir&oder ich sage, grade dieser Junge der jetzt grade aus Russland bei uns ist, beide Hände verloren hat, durch 'n Stromunfall, der hatte gestern das große Grinsen im Gesicht, als er seine Hand das erste Mal bewegt hat, wo ich ihm sage: „Du wirst nochmal weinen. Das wird nochmal richtig schlecht werden.“, das was (Name von Teilnehmer 2 aus Datenschutzgründen ausgelassen) gesagt hat, diese Talsohle, das find ich ganz wichtig, dass man das den Leuten erzählt. Wir kriegen oft aus Kliniken multitraumatisierte Patienten geschickt mit beiden Armen weg und beiden Beinen weg und dann geht irgendein Arzt her und sagt: „Nachher wird schon alles wieder gut sein.“ Ja, das sind falsche Infos. Also man muss den Leuten wirklich sagen äh: „Du hast 'n Handicap, freu dich wenn-„ und dann zeigen wir Filme von Leuten die versorgt sind und dann merken die Leute: „Ja, ich sehe das, ja.“. Man kann unheimlich viel über Film auch motivieren. Da seh' ich das jemand wirklich alles wieder, oder viele Dinge wieder, tut mit zwei Prothesenhänden und das finde ich wichtig. Aber ich denk, wenn die Vorbereitung stimmt, die Information stimmt, die Schulung stimmt, dann kenn ich keinen Patienten der mh da

ganz ausbricht, oder ich sag mal rückfällig wird, oder, wenn die Sozialfaktoren alle stimmen. #00:23:02-9#

Interviewerin: Ja. Und trotzdem haben wir jetzt davor gesagt, dass grade einfach, dass ‘n Signal zu bekommen, dass die Elektroden eben funktionieren und ‘n Signal empfangen, dass das ja einfach viel Übung äh braucht. Also, dass das- #00:23:21-1#

Teilnehmer 27: Braucht das viel Übung? (An Teilnehmer 3 gewandt.) #00:23:19-5#

Teilnehmer 29: Unterschiedlich. #00:23:20-9#

Teilnehmer 27: Das ist nur deine Interpretation, aber bei mir kommen die Kinder an, die haben noch nie ‘n Arm bewegt, ich weiß wo ich die Elektrode hinsetz‘. Ja, (Interviewerin: Mhm (bejahend)) wir sehen Schäfte, da ‘se-&werden Elektroden an Positionen gesetzt, wo man sagt, es ist nicht machbar. Es ist physiologisch und topographisch da wo die sitzt nicht na-&machbar in einem Elektrodenschaft da ‘n stabiles Signal zu kriegen. (Interviewerin: Mhm (bejahend)) Und dann kommen die Leute her und sagen: „Ja, die Hand, dann macht ‘se da automatisch auf und hier automatisch zu und das will ich alles gar nicht.“, das sind alles Schaftfehler, Prothesenschaftfehler (Interviewerin: Mhm (bejahend)) und die führen dann zu solchen Ergebnissen, dass auch irgendwelche Ärzte in Büchern schreiben: „Armprothesen werden nicht getragen.“, wie mein Alter Chef (Name eines Arztes aus Datenschutzgründen ausgelassen). (Interviewerin: Mhm (bejahend)) Ja? Und das&das sind die Fehler. Aber ich meine, wenn nicht jemand ‘n Plexusausriss hat, kann jeder, wir kriegen immer von Kostenträgern: „Das Kind soll einen Myotest machen“, mit Dysmelie. Ich hab‘ in 25 Jahren noch kein Kind gehabt, was kein Myosignal entwickelt, weil’s jetzt ‘ne Dysmelie hat. (Interviewerin: Mhm (bejahend)) Aber die Lehrmeinung sagt, wir müssen einen EMG Test machen. (Interviewerin: Mhm (bejahend)) Und wenn ich die Elektrode richtig positioniere, muss der auch nicht lernen. Das ist physiologisch, ich muss nur über die Physiologie wissen. (kurze Pause) #00:24:26-3#

Interviewerin: Und wenn aber jemand ‘n Plexusausriss hat? #00:24:29-1#

Teilnehmer 27: Dann ist die Frage, wenn er dann noch ‘n Plexusausriss hat und ‘ne hohe Amputation, eine Oberarmamputation. Dann ist man, aus meinem Wissen schon von vorne rein enorm eingegrenzt über die langfristige Versorgungsqualität eines solchen Patienten (Interviewerin: Mhm (bejahend)). Man sollte eher über irgend ‘ne TMR Operation nachgedacht werden, die Physiologie zu verbessern, als dem eine mulitartikulierende Hand oder das Zauberwerk der Nation vorzustellen. Der bringt dann einfach nicht das mit, was er eigentlich für dieses Hilfsmittel mitbringen muss. Und das machen wir oft, wir lassen uns Röntgenbilder geben, wir sind mit vielen Ärzten vernetzt, wir schicken den auch vor ‘ner Versorgung zur stumpfverbessernden Maßnahme irgendwohin (Interviewerin: Mhm (bejahend)) und das wird viel zu wenig gemacht, das seh‘ ich an Patienten, die zu uns kommen, wo einfach darüber überhaupt nicht nachgedacht wird. (Interviewerin: Mhm (bejahend)) #00:25:12-5#

Interviewerin: Das heißt eben wenn jemand einfach so diese Voraussetzung, dass es rein physiologisch machbar ist, die nicht mitbringt, dass man dann einfach oft

sagt, bevor man jetzt hier an der Prothese äh werkelt und das sonst wo anbringt, dann schickt man ihn lieber nochmal zum Arzt, damit da einfach nochmal- #00:25:28-1#

Teilnehmer 27: Genau. #00:25:28-1#

Interviewerin: Ok, mhm (bejahend). Und gibt's das dann auch, dass dann einfach nochmal 'ne Amputation durchgeführt wird, um die Stumpflänge zu verändern? #00:25:37-3#

Teilnehmer 27: Das gibt es, natürlich. (Interviewerin: Mhm (bejahend)) Wir haben jetzt 'ne neue Oberarmop-&oder jetzt, das war vor drei Jahren, 'ne Veränderung am Oberarmschaft, zur funktionellen Verbesserung am Schaft, gemacht, mit Ärzten. (Interviewerin: Mhm (bejahend)) Das wird jetzt zunehmend weiter in Deutschland gemacht, aber das ist-. Suchen Sie 'n Facharzt für Orthopädie, für technische Orthopädie. Den gibt es nicht mehr. Den gibt es nicht mehr. (Interviewerin: Mhm (bejahend)) Es gibt&da gibt's noch den (Name eines Arztes aus Datenschutzgründen ausgelassen) in (Name des Wohnortes aus Datenschutzgründen ausgelassen), ja? Dann gibt's noch 'n (Name eines Arztes aus Datenschutzgründen ausgelassen), dann gibt's noch (Name eines Krankenhauses aus Datenschutzgründen ausgelassen) ist&löst sich auch fast auf, also. Fachärzte da zu kriegen enorm schwer. (Interviewerin: Mhm (bejahend)) #00:26:19-4#

Interviewerin: Bei den anderen? Also vielleicht mh, vielleicht muss ich das umformulieren, vielleicht nicht mal jetzt Probleme, sondern einfach Dinge, wo man sagt, da muss ich besonders genau hingucken, da muss ich nochmal, also das kostet mich vielleicht mehr Zeit die Prothese anzupassen, einfach dass wir auf sowas gucken. Wo&wo, ja vielleicht nicht Schwierigkeiten, sondern was zeitaufwändiger ist, bei der Anpassung, wenn wir mal vom Schaf absehen. #00:26:39-4#

Teilnehmer 28: Ja bei manchen ist es halt Kosmetik. Ist halt manchmal nach dem guten Schaft, die zweite Herausforderung. (Interviewerin: Mhm (bejahend)) Also Komponenten unterzubringen und auch in 'ner Art, dass es halt schlank ist (Interviewerin: Mhm (bejahend)) und 'ne Ästhetik hat und nicht 'n Ofenrohr. (Interviewerin: Mhm (bejahend)) #00:27:01-5#

Interviewerin: Und was für 'n Spielraum gibt's da? #00:27:04-1#

(kurze Pause) #00:27:06-0#

Teilnehmer 28: Ja, das ist schon sehr anspruchsvoll (lachend). Ist schon- #00:27:10-0#

Teilnehmer 27: Normalerweise gibt's den Spielraum sehr gering (Interviewerin: Mhm (bejahend)), aber wir erweitern den Spielraum. Wir sägen auch mal 'ne (Name eines Prothesenherstellers aus Datenschutzgründen ausgelassen)-Hand ab, damit's eben ästhetisch wird und wir nehmen irgendwelche Industrieakkus und kaufen nicht bei (Name eines Prothesenherstellers aus Datenschutzgründen ausgelassen) ein, weil die dünner, flacher und leistungsfähiger sind. Ähm es ist in der Fantasie des Orthopädietechnikers, ja? (Interviewerin: Mhm (bejahend)) Die Industrie gibt uns 'n

kleinen, kleinen Teil vor. Wenn Sie im Kinderbereich gucken (räuspert sich), gibt's fast nichts. (Interviewerin: Ja.) Ja, also erschreckend. Ich sag immer den Entwicklungsleuten bei (Name eines Prothesenherstellers aus Datenschutzgründen ausgelassen): „Das ist euer Klientel der Zukunft und ihr macht da nichts.“, das gibt's doch nicht. (Interviewerin: Mhm (bejahend)) Ja, also das ist wirklich beschämend. Aber es liegt in der Fantasie des Orthopädietechnikers dann aus der Situation das so zu machen. Wenn wir sehen, was wir drüber nachdenken, wie über Passteilpositionierung und Aufbaulinien, dass es nachher alles ein Bild hat und eine Linie hat und auch akzeptiert wird. (Interviewerin: Mhm (bejahend)) Zu uns kommen so viele Kinder und äh auch Erwachsene, die furchtbar aussehende Prothesen haben. Hart, unförmig, unmenschlich, sag ich mal. Ähm, da verändern wir alles und sehen auf einmal, da ist ‘ne riesen Akzeptanz da, die vorher gar nicht da war. (Interviewerin: Mhm (bejahend)) #00:28:22-3#

Interviewerin: Und wenn Sie sagen eben, das haben Sie vorher schon kurz erwähnt (wendet sich an Teilnehmer 28), dass eben einfach Leute kommen und dann sieht man die Hand ohne den Handschuh und dann wird der drauf gemacht und das ist nicht das was man sich vorgestellt hat. Was haben denn die Menschen die kommen für Erwartungen? Wie&also&oder was stört am meisten? Ist es die Länge, die Form oder das Aussehen, was sind da so die Sachen, wo Sie sagen: „Das ist bei der Ästhetik am wichtigsten.“? #00:28:49-8#

Teilnehmer 28: (Es ist, nochmal?), es ist die Irreführung von der Industrie (Interviewerin: Mhm (bejahend)). Weil mh 90% der Hände werden gezeigt ohne Handschuh (Interviewerin: Mhm (bejahend)). U:nd natürlich im Video hört man nicht die Motorengeräusche (Interviewerin: Mhm (bejahend)). Das sind denk-&das sind also für mich zwei Punkte, wo (kurze Pause) ‘n großes Gap ist zwischen dem was suggeriert wird und was der Patient dann erhält. (Interviewerin: Ja.) #00:29:25-6#

Interviewerin: Und aber das, wo Sie sagen, dass Sie da Ihre Fantasie einsetzen und eben Sachen verändern, was sind die meisten Veränderungen, ist es grade die&die Länge, die Größe oder ist es auch einfach, weiß nicht, das Aussehen vom Handschuh? #00:29:35-3#

Teilnehmer 27: Alles. Alles zusammen. #00:29:37-3#

Interviewerin: Ok. #00:29:37-3#

Teilnehmer 27: Wir verkleiden Prothesen anders als andere, die dann auf einmal leiser werden durch diese Verkleidung (Interviewerin: Mhm (bejahend)). Die ein ästhetisches Bild haben und nicht hier ‘ne Schraube und hier ‘ne Kante und hier irgendwas, sondern die wirkliche eine möglichst formschöne Ästhetik einfach geben, ähm da kann man viel machen, aber es wird zu wenig gemacht. Es wird auch sowas von der Industrie gar nicht unterstützt (Interviewerin: Mhm (bejahend)), ja? Da heißt es, ‘n kleiner Bereich die Armprothetik in der Orthopädie, wer macht's, wer ist, wer tummelt sich da? (Name eines Prothesenherstellers aus Datenschutzgründen ausgelassen). Und ansonsten gibt's gar nicht mehr so viele, (Name eines Prothesenherstellers aus Datenschutzgründen ausgelassen) noch und dann (Name eines Prothesenherstellers aus Datenschutzgründen ausgelassen) (Interviewerin: Mhm (bejahend)) und&aber das sind auch keine Konkurrenzprodukte, oder (Name eines Prothesenherstellers aus Datenschutzgründen ausgelassen) kauft die Firmen

auf und geht dann doch wieder in diese Linie. Ähm, ja. (Interviewerin: Mhm (bejahend)) #00:30:22-8#

Teilnehmer 28: Oder die Gegenbewegung (grade so? (..?)) macht noch. #00:30:28-1#

Teilnehmer 29: Ja, ja. Ok. Ich wollt eigentlich noch was zur&zur&zur&zur Kosmetik sagen. (Interviewerin: Ja.) Also was wir, was wir sehr viel machen ist wirklich ähm äh viel tricksen mit der Anordnung der Bauteile, aber das haben meine Kollegen eigentlich auch schon so im Wesentlichen gesagt (Interviewerin: Mhm (bejahend)). Ähm da muss man wirklich sehr kreativ werden. Aber es geht dann manchmal nicht so sehr um die Kreativität, sondern es geht einfach darum äh etwas austüfteln zu können und ähm äh Erfahrung beim Aufbau zu haben, Augenmaß zu haben äh, beim Aufbau Hilfsmittel zu haben. Meistens nur einfache Hilfsmittel, ähm wir bringen unsere Bauteile halt in irgendwelchen Krümmungen von irgendwelchen ähm Stümpfen an, wenn solche Krümmungen vorhanden sind, wir ver-&wir testen unterschiedliche Anordnungen aus, in dem wir die Bauteile oder Dummys für die Bauteile mit&mit Gummiringen einfach befestigen (Interviewerin: Mhm (bejahend)) und uns verschiedene Positionen anschauen. Wir erzeugen sogar durch ‘ne veränderte Handstellung halt ähm Räume in denen wir die Bauteile reinsetzen können, also da werden wir sehr kreativ und haben auch sehr viel Erfahrung. Das ist auch etwas was gar nicht ohne weiteres zu handhaben ist, wenn man nicht ‘ne größere Menge an Prothesen baut. (Interviewerin: Mhm (bejahend)) Besonders schwierig sind da eigentlich die, jetzt im Unterarmbereich die Langstümpfe, und äh Stümpfe mit Fingerrudimenten, für meine Begriffe. Da wo man wirklich sehr viel hinschauen muss. Ähm kürz-&mittellange Stümpfe oder kürzere Stümpfe, da hat man dann Einbauplatz für Komponenten (Interviewerin: Mhm (bejahend)) aber in allen anderen Bereichen muss man bei Langstümpfen oder Fingerrudimenten, muss man da wirklich sehr kreativ werden und da kitzeln wir schon das Bestmögliche raus und das erfordert viel Erfahrung. Erfordert auch viel, dass man unter den Bauteilen die man zur Verfügung hat, die besten auswählt, die geeignetsten auswählt (Interviewerin: Mhm (bejahend)) ähm geht bis hin dazu, dass wir selbst unsre eigenen Akkusysteme für Kinder löten (Interviewerin: Mhm (bejahend)). Die wir halt besser einsetzen können und da wird der Aufwand manchmal schon ‘n bisschen größer, wenn’s wirklich darum geht genau das Beste was erstmal Größe der Prothese, Länge der Prothese, wenn’s darum geht das Beste rauszukitzeln, geht’s&dann wird’s auch wirklich auch aufwendiger (Interviewerin: Mhm (bejahend)) mit der Fertigung. #00:32:47-2#

Interviewerin: Und wenn Sie sagen eben mit den Fingerrudimenten oder den längeren Stümpfen, ist es da einfach der Platz, der fehlt, um Bauteile- #00:32:54-1#

Teilnehmer 29: Genau, es ist halt zwischen Prothesenhand und ähm Stumpfende kein Anbauplatz vorhanden, also müssen wir an die Seite dransetzen, die ganzen Bauteile und da muss man halt gucken, wo kriegt man das unter. Wir planen da sogar Muskelbäuche an die&die danach arm(oval?) bevorzugt Bauteile äh reinsetzen ähm also (Interviewerin: Mhm (bejahend)), ja wir berücksichtigen da viele Faktoren und wenn’s letztendlich nicht möglich ist ‘n schönen Arm zu bauen, dass es auch zumindest nur so wenig&so weit wie möglich reduzieren, in dem wir halt uns vorstellen, aus welchem Blickwinkel fallen denn irgendwelche Unebenheiten die wir da drin haben in der&in der Kosmetik, in der Silhouette der Prothese. Ähm wo&wo

packen wir diese Bauteile hin, damit das möglichst nicht ins Auge fällt (Interviewerin: Mhm (bejahend)). Also, manchmal&wir können da nicht immer Wunder leisten, aber wir leisten da ziemlich viel. (Interviewerin: Mhm (bejahend)) #00:33:39-9#

Interviewerin: Sie wollten grade auch noch was sagen, Herr (Name von Teilnehmer 2 aus Datenschutzgründen ausgelassen)? #00:33:41-3#

Teilnehmer 28: Das war nur allgemein, (wir haben jetzt?) innerhalb von der einen Richtung, Tendenz zur multiartikulierenden Hightechindustrie und die andere, natürlich auch durch den (..?) jetzt kommt grad die ganze 3D-Welle (Interviewerin: Mhm (bejahend)), ähm wo auch alles groß bejubelt wird und ja, man kann jetzt 'ne Hand da für 200 Dollar herstellen und in ('ner Dreiviertelstunde?) is' 'se fertig. Das ist auch jetzt 'n&so 'n Gegentrend, aber der ist auch 'n irritierendes Element gerade (Interviewerin: Mhm (bejahend)), find ich. (Interviewerin: Ja.) #00:34:12-8#

Interviewerin: Ok. Gibt's da zu dem Punkt noch irgendwas, was jemand hinzufügen möchte? #00:34:16-8#

(kurze Pause) #00:34:19-8#

Interviewerin: Gut. Dann, das haben Sie vorher ganz kurz angesprochen (wendet sich an Teilnehmer 27), das hab' ich nämlich auch einfach im Gespräch mit Prothesennutzern immer wieder gehört, mh dass man, dann die Prothese bekommt und eigentlich hat und am Ende doch einfach nicht trägt. Sie haben gesagt, dass Sie sowas gar nicht kennen, dass es sowas-. Aber können Sie sich vorstellen, eben woran es liegt, wenn das da- #00:34:47-6#

Teilnehmer 27: Schlechte Schäfte. #00:34:49-1#

Interviewerin: Mhm (bejahend) #00:34:50-5#

Teilnehmer 27: Keine Details beim Gipsabdruck. (Interviewerin: Mhm (bejahend)) Wir hören, wenn wir uns 'ne Stunde vor 'm Gipsabdruck mit den Patienten zusammensetzen, dann äh sagt der mir: „Das hat noch nie jemand gemacht. Ich bin da rein, dann hat d-&haben die 'ne Gipsbinde da rumgewickelt und dann war's das.", ja? Wir geben uns da einfach Mühe, 'n Röntgenbild anzugucken, zu palpieren, zu gucken, wie ist die skeletale Struktur. (Interviewerin: Mhm (bejahend)) Wir haben viele Dysmelien, da ist alles anders. Das ist kein Anatomiebuch, da muss man gucken oder man verschenkt unheimlich viel. Und wer die Sorgfalt da zu dem Zeitpunkt nicht aufwendet, der wird auch kein Ergebnis kriegen. (Interviewerin: Mhm (bejahend)) #00:35:24-7#

Teilnehmer 29: Der Schaft ist das Wichtigste, ja. Also es (spielt?) natürlich die Kosmetik für die Akzeptanz 'ne Rolle oder ob die Prothese weich ist oder hart ist (Interviewerin: Mhm (bejahend)), bei uns sind sie halt weich ähm, was die Akzeptanz auch erhöht (Interviewerin: Mhm (bejahend)). Aber Prothesenschäfte sind das Wichtigste und da müssen erstmal auch wichtig stabile Elektrodensignale gegeben werden können. Ich beobachte zum Beispiel immer wieder, dass Patienten, die&die ich dazu auffordere ähm die Prothese anzusteuern, dass sie dann plötzlich anfangen, die Prothese irgendwie zu halten und irgendwie Kräfte ausüben (Interviewerin: Mhm (bejahend)). Eigentlich nur, um den Elektrodenanpressdruck zu

erhöhen (Interviewerin: Mhm (bejahend)). Für meine Begriffe. Und das beobachte ich bei unseren Prothesen, die wir machen, das beobachten wir höchstens in der&in der Fertigungsphase, aber ich beobachte das bei ähm Schäften, die entweder nicht mehr passen oder die von Anfang an schlecht waren, (die sind dann meisten aus anderen Betrieben?) oder halt unsere Probeversorgung, die auch mal, im Ausnahmefall, natürlich erstmal, das ist ja Sinn und Zweck einer Probeversorgung, erstmal vielleicht nicht funktionieren, da müssen wir was ändern, an dem Elektrodenanpressdruck, wie weit ragen die Elektroden nach innen rein oder vielleicht muss was verengt werden. (Interviewerin: Ja.) Aber wenn das nicht stimmt äh, wenn die Elektrodensignale nicht, A nicht gut sind und B auch nicht stabil sind, unter Belastungssituationen nicht stabil sind, ähm dann ist das ‘n richtig großes Ärgernis. (Interviewerin: Mhm (bejahend)) Und was auch ‘n Ärgernis ist, ist wenn die Prothesenhaftung nicht stimmt, ähm das ist auch n Faktor, wenn die s-&äh runterrutscht. Äh das ist wirklich, das&das Schaftdesign, das ist das Wichtigste. (Interviewerin: Mhm (bejahend)) Ja, mit das Wichtigste. Kosmetik mag ‘ne Rolle spielen in der Akzeptanz, aber- #00:36:43-4#

Interviewerin: Ja. Aber Schaft und dadurch eben der Sitz der Elektroden- #00:36:47-7#

Teilnehmer 29: Der Sitz der Elektroden, genau, ja. (Interviewerin: Ja.) Und natürlich Druckstellen auch. Aber das ist ‘ne relative Selbstverständlichkeit, dass man das irgendwie versucht zu beheben. (Interviewerin: Mhm (bejahend)) Dazu muss man sich auch die knöchernen Strukturen anschauen, grade bei Dysmelien. Ä::hm auf meine Begriffe (gerade?) wirklich Elektroden, stabile Elektrodensignale und ähm die Prothesenhaftung (Interviewerin: Mhm (bejahend)) sind wichtige Faktoren. (Interviewerin: Ja.) #00:37:13-7#

Interviewerin: Fällt Ihnen noch was ein, Herr (Name von Teilnehmer 2 aus Datenschutzgründen ausgelassen) dazu? #00:37:16-8#

Teilnehmer 28: Wenn der Patient glaub ich (räuspert sich) glaub ich, nicht aus der Versorgung rausgeht und kein Erfolgserlebnis hat, was er im Kopf speichert, dann ist die Toleranz mit der Prothese (Interviewerin: Mhm (bejahend)) danach schwieriger. Und das kann dann dazu führen, dass sie irgendwann im Schrank liegt und er sie nicht benutzt. (Interviewerin: Mhm (bejahend)) Ich denk, wenn man gute Erfolgserlebnisse gibt und aufzeigt, was er damit machen kann, dann wird er sie auch nicht in den Schrank legen. #00:37:59-6#

Interviewerin: Das heißt dann sind wir einfach wieder bei diesem Psychologischen, dass man den halt an der richtigen Stelle (Teilnehmer 28: Auch, ja.) abholt und, ja, mhm (bejahend)) #00:38:06-2#

Teilnehmer 28: Ich denk, wissen Sie, er soll nicht des Gefühl kriegen, es ist ‘ne Belastung, sondern es ist ein positives Erlebnis, was ihn für ‘ne lange Zeit begleiten wird. (Interviewerin: Ja.) Und wenn der Weg&Pfeil nach oben zeigt, dann ist es glaub ich gewährleistet, dass er merkt, mit Prothese gehen einige Sachen besser, (Interviewerin: Mhm (bejahend)) es gibt auch Momente wo Patienten keine Lust habe ‘ne Prothese zu tr-&tragen, (Interviewerin: Mhm (bejahend)) aus meiner Sicht ist das auch tolerabel, nur er sollte sich dran erinnern, dass er noch eine hat. (Interviewerin: Ja, ok.) #00:38:45-3#

Teilnehmer 27: Pubertätszeit, zum Beispiel. Ja? Wenn wir die Kinder haben, wo wir sagen in der Pubertät da geht das so. Aber das ist normal, da geht auch andere Hormone mit denen durch (alle lachen). Das muss man wissen, den Eltern erklären, (Interviewerin: Mhm (bejahend)), vorausschauen und sagen: „Auch danach wird es wieder ‘ne andre Zeit geben.“, wir müssen gucken, dass wir in der Pubertät trotzdem das Kind an der Prothese gewöhnt lassen (Interviewerin: Mhm (bejahend)), dass nicht ‘n&‘n Abbruch dieser ganzen Entwicklungszeit kommt, sondern das man, mit welchen Mitteln auch immer, zu sagen man&das Kind präferiert dann&das Mädchen die Habitusprothese, wo’s die Fingernägel lackieren kann und eben unauffällig ist, ja? Das sind dann so kleine Brücken, die dann einfach bis, ich sag mal 18, 19, das Kind, oder den jungen Menschen, da dran halten die Prothese weiter zu tragen und dann trägt er sie auch wieder (Interviewerin: Ja.). #00:39:34-9#

Interviewerin: Ja, ok. Ähm und wenn wir jetzt einfach mal sagen jemand hat eben die Versorgung bekommen, hat die Prothese, nimmt die mit nach Hause und nutzt die auch wirklich viel. Was bekommt man denn danach ähm von Patienten, von Prothesenträgern zurückgemeldet an positiven, oder negativen Erfahrungen mit der Prothese, eben grade vielleicht wenn wir darauf gucken, dass viele mit ‘ner ganz ähm klaren Vorstellung rangehen, die vielleicht enttäuscht wird. Was sind da Erwartungen, die vielleicht nicht erfüllt werden, oder auch Erwartungen die übertroffen werden, wo gesagt wird: „Super, da funktioniert das jetzt viel besser, viel einfacher.“. Was können Sie da berichten, von&von Erfahrungen, die da rückgemeldet werden? #00:40:15-8#

Teilnehmer 27: Also wir haben&wir kriegen süß aus&aus Urlaubsaufenthalten dann Bilder geschickt, was die machen und was die an Sport damit machen und an Bewegung damit machen (Interviewerin: Mhm (bejahend)), oder Videos per E-Mail und ähm nettes, wirklich total schönes Feedback, WhatsApp, alles was da heute, Facetime, Bilder, und&und Videos und äh wo wir einfach sagen, es ist toll da hinzugucken und den Stolz zu sehen, wie die das&wie die sich gut fühlen und was die für Spaß da dran haben (Interviewerin: Ja.) und das&das ist ‘n tolles Gefühl. #00:40:42-0#

Interviewerin: Ja, das glaub ich. #00:40:44-8#

Teilnehmer 29: Wenn äh beidseitige Aktivitäten gelingen, denk ich mir, das ist äh ‘n großes Erfolgserlebnis. Ähm also beidseitig heißt, wo man ein Gegenstand hält mit der der Prothesenseite meistens und dann halt äh mit der gesunden Seite irgendwas damit machen kann. Ähm aber ‘n gutes Beispiel ist auch das Radfahren, also grade diese sportlichen Aktivitäten, grade für Kinder Dreirad fahren (Interviewerin: Mhm (bejahend)) ist so etwas wo der größte Aha-Effekt ist, (Interviewerin: Mhm (bejahend)) weil das können ‘se mit Pro-&ohne Prothese nicht. Ähm alle Aktivitäten die mit Stumpfaktivität nicht äh möglich sind, da kommt das größte Aha-Erlebnis, grade für&für Kinder (Interviewerin: Ja.). Mhm (bejahend). Ähm sportliche Aktivitäten können da auch immer wieder äh wichtig sein für Kinder, auch weil’s natürlich ‘n sozialen Aspekt gibt, auch für Erwachsene genauso. Weil man das halt in der Gemeinschaft irgendwie tut und (..??) zusammentut. #00:41:26-3#

Interviewerin: Ja. #00:41:26-3#

Teilnehmer 29: Ja. Ansonsten was beidseitige Tätigkeiten anbetrifft äh so die al-&die Aktivitäten des alltäglichen Lebens, also sich ankleiden, ähm manche Hausarbeiten, die damit besser gehen, ist denk ich mir ‘ne Erleichterung (Interviewerin: Mhm (bejahend)). Ähm Auto fahren vielleicht, könnte so ‘n Aha-Effekt sein. (Interviewerin: Ja.) #00:41:45-8#

Teilnehmer 28: Des Laufen auf der Straße, einfach. #00:41:51-3#

Teilnehmer 29: Das Unauffällige. #00:41:50-3#

Teilnehmer 28: Das Unauffällige. Du hast nicht mehr ‘n losen Ärmel, der da rumbaumelt. #00:42:09-5#

(Störende Geräusche im Hintergrund.) #00:41:59-2#

Teilnehmer 27: Stört das? #00:42:00-6#

Interviewerin: Ist das auf der Aufnahme? #00:42:00-2#

Protokollant: Kaum. Die Stimmen übertönen das. #00:42:09-8#

Teilnehmer 27: Ok. #00:42:10-2#

Teilnehmer 28: Ich denk einfach so das Laufen auf der Straße (Interviewerin: Mhm (bejahend)). Du wirst nicht mehr, (kurze Pause) das die Leute so gucken. Du bist mh vom Erscheinungsbild einfach integriert (Interviewerin: Mhm (bejahend)). Das ist&das ist, was ‘n großer Vorteil ist. (Interviewerin: Mhm (bejahend)) Die müssen sich nicht mehr ganz so (kurze Pause) verstecken und so weiter. (Interviewerin: Mhm (bejahend)) #00:42:35-3#

Teilnehmer 29: Das Unauffällige, das ist den meisten wichtig. Die meisten Leute wissen zwar, dass sie letztendlich ihre Behinderung nicht wirklich verbergen können (Interviewerin: Ja.), irgendwann merken die Leute natürlich, dass keine gesunde hat&Hand hat, sondern eine Prothese (Interviewerin: Mhm (bejahend)) aber, ähm man wird auch als&als Behinderter oder als Amputierter, immer wieder angeschaut von den Leuten, wenn man das ohne weiteres erkennen kann. Mit einer Armprothese erkennt man das nicht ohne weiteres, nicht auf ‘n flüchtigen Blick zumindest (Interviewerin: Ja.) ähm und da sind die Leute ganz froh drüber. Auch selbst wenn sie keine blöden Reaktionen von andern Menschen erfahren (Interviewerin: Mhm (bejahend)), ähm ist es ihnen doch lästig darauf vielleicht (täglich?) angesprochen zu werden, auch wenn man höflich angesprochen wird. Grade Kinder, hab’s&ich kenn die Schilderungen von&von Amputierten, die äh die Erfahrung haben mit Kindern die immer wieder da neugierig fragen: „Was ist da passiert?“ und „Wieso hast du keine Hand, wieso hast du keinen Arm?“. Ähm das können dann viele verstehen, das heißt die sagen erstmal Kinder müssen das fragen, Kinder wollen das wissen (Interviewerin: Mhm (bejahend)) aber irgendwann stört’s mal (Interviewerin: Ja.). Das ist mal wieder die&die Antwort, also die&die Äußerung, die man bekommt von Amputierten. Und es ist einfach gut für die auch einfach sich unauffällig im öffentlichen Raum bewegen zu können, ohne dass es jeder sofort sieht. Also auch wenn’s keine dummen Reaktionen gibt allein, dass Leute mal hinschauen, für ‘n paar Sekunden nur hinschauen (Interviewerin: Mhm

(bejahend)) und dann wegschauen, allein das ist halt auf die Dauer schon bisschen nervig. Selbst wenn’s keine blöden Blicke sind. (Interviewerin: Ja.) Dieses Unauffällige, das ist den Meisten auch schon natürlich ‘n Gewinn, (Interviewerin: Mhm (bejahend)) der drüber hinaus kommt. Über den funktionellen Gewinn hinaus, ist das halt ‘n Aspekt für viele auch (Interviewerin: Mhm (bejahend)). #00:43:54-0#

Interviewerin: Und an negativen Sachen? An Sachen, wo&wo vielleicht mehr erhofft war, als dann im Endeffekt geht? Gibt’s da irgendwas, was man häufig zurückbekommt? #00:44:05-0#

Teilnehmer 27: Schon. Wenn ich so seh‘, jemand, wir hatten jetzt ‘n Jungen aus Albanien da, Schulter exartikuliert, also gar kein Arm mehr auf der einen Seite und ein ganz kurzen Unterarm (Interviewerin: Mhm (bejahend)) mit dem hab ich am Tisch gesessen und gegessen und der hat sein Ei gegessen und sein Ei aufgeschlagen und’s war wirklich faszinierend zu sehen, mit welchem Gefühl er seine Hand bewegt (Interviewerin: Mhm (bejahend)), aber wo man ganz klar auch sieht, da ist jemand mit ‘ner so hohen Amputation, ist einfach limitiert, ja? Und das ist natürlich, wenn ich jetzt jemand seh‘, lange Unterarme beidseits und sehe beidseits Schulter Ex oder Schulter Ex und Oberarm, da sehen wir ganz klar, wo die Natur da Grenzen setzt. (Interviewerin: Mhm (bejahend)) Und da können wir den elektrischen Ellbogen und die elektrische Schulter und äh selbst mit TMR und ‘ner feineren Ansteuerung äh da ist schon schwierig, also da ist man einfach limitiert. Die Leute leben damit, wir haben viele solcher Patienten, die haben auch ‘n glückliches Auftreten, sind harmonisch in der Familie, Familie gegründet, Beruf, Sport, Freizeit, aber man sieht doch einfach was da einfach an Limitierung ist (Interviewerin: Mhm (bejahend)). #00:45:38-1#

Interviewerin: Ja. Fällt da den andren noch was ein? #00:45:41-1#

(kurze Pause) #00:45:42-6#

Teilnehmer 28: Im Bezug auf Armp-&ja, (kurze Pause) was&was er auch meinte, im Prinzip kann man bestätigen, je höher des Amputationsniveau ist (Interviewerin: Mhm (bejahend)), dann tauchen da einige Einschränkungen ein, wo man halt realisieren muss, man ist nicht ganz wieder so, wie man sein könnte (Interviewerin: Mhm (bejahend)) #00:46:00-8#

Interviewerin: Also einfach so ‘n bisschen das, dass halt doch die Prothese eben nicht, der Arm ist, der fehlt, sondern halt einfach ähm ‘n Hilfsgerät, das halt einfach nicht alles möglich ist? #00:46:05-9#

Teilnehmer 27: Genau. Die Natur ist das nicht so schnell zu toppen (Interviewerin: Mhm (bejahend)). Also wir haben wirklich Patienten wo wir sagen, ähm erstaunlich was die leisten, mit egal multiartikulierender Hand, oder auch (Name eines Prothesenherstellers aus Datenschutzgründen ausgelassen) Systemhand und auch sich selbst als nicht ein-&oder gehandicapt eigentlich einschätzen (Interviewerin: Mhm (bejahend)). Wo ich echt sag, Hut ab wie die sich auch selber in diese positive Entwicklung reintriggern und reinbringen (Interviewerin: Ja.). Aber es ist auch ‘ne&sehr ‘ne Frage natürlich der Persönlichkeit des einzelnen Patienten, des Amputierten, zu sagen: „Guck ich da nach vorn, oder guck ich da nach hinten?“. Und da&wenn man da gut begleitet und auch motiviert, das was wir immer tun mit

Vernetzung mit anderen Patienten ja, Patient kommt zu Patient und sieht nicht nur, (Name von Teilnehmer 2 aus Datenschutzgründen ausgelassen) hat so schön gesagt, ‘n übertriebenen Werbefilm auf YouTube, sondern‘s nimmt wirklich Realität wahr, dann ist das ‘ne tolle Motivation und auch ‘ne ehrliche Motivation und die hilft meines Erachtens wirklich weiter (Interviewerin: Mhm (bejahend)). Und ich seh’s immer so, dass ich sag, das ist wie in der Erziehung. Der erste Baustein, der muss gut sein für den Patienten, der durch ‘n Trauma zum Beispiel seine Hände verloren hat (Interviewerin: Mhm (bejahend)) und für den natürlich auf einmal das ganze Leben sich komplett gedreht hat (Interviewerin: Ja.). Wenn der zu Anfang ‘ne gute Vorbereitung, Schulung, Information, Vernetzung hat, dann kriegt der trotz Handicap glaub ich ‘ne Stabilität hin. Ich seh‘ immer wieder an den Patienten, wo’s von vornherein schief ging, ‘n schlechtes Management war, egal von Therapeuten, Ärzten, Technikern, äh dass dann nachher rauszukriegen, ist ganz schwer. Das ist wie ‘ne schlechte Erziehung, oder ‘ne schlechte Angewohnheit (Interviewerin: Ja.). Also da finde ich, muss viel mehr Richtung gewiesen werden und&und professioneller unser Beruf auch auftreten, von den Ärzteschaften angefangen über Information, das ist ganz, ganz wichtig. (Interviewerin: Mhm (bejahend)) #00:48:01-9#

Interviewerin: Und wenn wir jetzt aber nicht mal so dieses generelle Bild, einfach die generelle, vielleicht Einstellung zur Prothese, sondern vielleicht explizit auf irgendwelche Funktionen, wo man sagt, die funktioniert irgendwie nicht richtig, oder da, weiß nicht, wenn ich irgendwas in meinem Alltag oder in der Arbeit machen will, dann funktioniert das nicht so, wie ich mir das eigentlich vorgestellt habe, dass es mit der Prothese geht. Gibt’s das irgendwas, was Ihnen einfällt, was da rückgemeldet wird? #00:48:29-1#

Teilnehmer 27: Mir fällt Wasserfestigkeit als erstes ein. (Interviewerin: Mhm (bejahend)) Was ‘n, finde ich, ‘n unheimlich wichtiger Punkt ist, ja? (Interviewerin: Ja.) Die&wir haben oft technische Ausfälle aufgrund von Feuchtigkeit, (Name von Teilnehmer 2 aus Datenschutzgründen ausgelassen) hat’s angesprochen, die Hand, die multiartikulierenden Hände, die mit Handschuh angeboten werden, die sind von der Oberfläche so scharfkantig kostr-&konzipiert, dass der Handschuh schnellstmöglich kaputt ist und dann Wasser eindringt, dann Ausfälle kommen, auch schleichende Ausfälle kommen. Also Wasserfestigkeit, finde ich, wär‘ ‘n ganz ganz wichtiger Punkt. #00:49:04-0#

Interviewerin: Ja. #00:49:06-8#

Teilnehmer 28: Batteriedauer (Interviewerin: Mhm (bejahend)) (kurze Pause) auch noch. Weil ich mein, klar, mit fünf Fingern, die ziehen mehr Strom, (Interviewerin: Klar.) und dann hat man Patienten, die haben ‘ne Bebionic jetzt und dann ist nach vier, fünf Stunden die Batterie leer. (Interviewerin: Mhm (bejahend)) Und das sind jetzt keine Wechselakkus, die man dann rausnimmt (Interviewerin: Ja.), grade jetzt&des sind integrierte, also: Prothese ausziehen, ans Ladegerät hängen. (Interviewerin: Mhm (bejahend)) #00:49:29-3#

Teilnehmer 27: Gewicht. Was (Name von Teilnehmer 3 aus Datenschutzgründen ausgelassen) schon gesagt hat. Vor allen Dingen diese distalen Gewichtsanteile (Interviewerin: Ja.). Ja, ich frag mich immer, warum baut man nicht ‘ne Hand und setzt den Motor über ‘ne Welle näher an den Stumpf. (Interviewerin: Mhm

(bejahend)) Ja, wo man einfach sagt: Reduzierung des distalen Gewichts. Wird zu wenig drauf geguckt. (Interviewerin: Mhm (bejahend)) Robustheit wird immer noch zu wenig drauf geguckt. Hab‘ ich schon gesagt mit Wasserfestigkeit, oder? #00:49:45-7#

Interviewerin: Ja. #00:49:44-0#

Teilnehmer 28: Was noch? #00:49:49-5#

Interviewerin: Dann hatten wir vorher kurz dieses&die Zuverlässigkeit, was so von vielen einfach als wichtigster Punkt genannt wird. In was für Situationen versagt das denn? Wo gibt&kriegt man da ‘ne Rückmeldung wo die Zuverlässigkeit eben einfach nicht gegeben ist? #00:50:00-6#

Teilnehmer 29: Also grade dann, wenn Kräfte ähm auf diesen Prothesenhänden hebeln, (Interviewerin: Mhm (bejahend)) dann ist die Grenze bei multiartikulierenden Händen gerade&gerade ziemlich schnell erreicht (Interviewerin: Mhm (bejahend)). Ich denke da an Stielwerkzeuge die man führen muss. #00:50:12-7#

Kaffeemaschine verursacht laute Hintergrundgeräusche. #00:50:12-7#

Protokollant: Vielleicht warten wir kurz? (alle lachen) Ist die per Fernbedienung ausgelöst (lachend)? #00:50:18-0#

Teilnehmer 27: Ja, ja (lachend) #00:50:15-7#

Teilnehmer 28: Raucherpause. (alle lachen) #00:50:24-3#

Teilnehmer 27: Das war’s. #00:50:24-9#

Teilnehmer 29: Also wenn&wenn Stielwerkzeuge geführt werden äh:: beim, mir fällt spontan das ähm:::, das Schieben einer Schubkarre an&ein (Interviewerin: Mhm (bejahend)). Äh, äh oder äh, vielleicht ähm wenn man beim Fahrradlenker ist es wahrscheinlich nicht so sehr gegeben, dass sich dieses&dieser Hebel verkantet, aber wenn man damit mal stürzt (Interviewerin: Mhm (bejahend)), wäre das halt auch so ‘ne Einwirkung, wo halt die Fingerbügel verbiegen. Bei einer Myo-Bock-Hand, die ja einfach und robust aufgebaut ist (Interviewerin: Mhm (bejahend)), verbiegen die Fingerbügel, bei den neueren Händen ähm brechen die äh Kraftübertragungselemente oder die Finger selbst oder verbiegen sich und sind meistens nicht mehr ohne weiteres zu reparieren (Interviewerin: Mhm (bejahend)) diese Fingerb-&Fingerbügel die kann man auch ganz gut&können wir noch gut biegen, mit unseren Werkzeugen die in der Orthopädietechnik üblich sind, ähm aber alle anderen Reparaturen an multiartikulierenden Händen sind deutlich schwerer (Interviewerin: Mhm (bejahend)) und das ist so (kurze Pause) ja, so&so&so ‘ne Sache, wo halt die Hände häufig versagen. Wenn man drauf stürzt, könnte auch passieren, da sind die auch anfälliger. (kurze Pause) Sonstige Situationen fallen mir jetzt nicht ein (Interviewerin: Mhm (bejahend)), wo die Hände kaputt gehen. Bei Kindern wär’s vielleicht noch Spiel im Sandkasten (lachend). Also wenn man dann im Handschuh hinterher viel Sand drin findet, das ist natürlich auch Gift für diese Prothesenhände (Interviewerin: Ja, klar.). #00:51:51-1#

Interviewerin: Noch irgendein Problem oder so ‘ne&so ‘ne negative Eigenschaft, die Ihnen einfällt, die&die rückgemeldet wird? Außer jetzt, wir hatten die Wasserfestigkeit, die Robustheit- #00:52:00-2#

Teilnehmer 27: Preisgefüge. Es ist eigentlich alles zu teuer. (Interviewerin: Mhm (bejahend)) Wenn man guckt, so ‘ne (Name eines Prothesenherstellers aus Datenschutzgründen ausgelassen) Hand, die ist in den 60er Jahren entwickelt, da hat sich Elektronik geändert (Interviewerin: Mhm (bejahend)), kleine Komponenten geändert, aber die liegt heute bei 80 000 Euro ‘s Stück und das ist eigentlich (kurze Pause). Also mein Wunsch wäre eigentlich, dass ‘n Patient, wir haben einige BG Patienten, die haben mehrere Handkomponenten, (Interviewerin: Mhm (bejahend)) die sind natürlich ganz anders aufgestellt im Reparaturfall, ja? (Interviewerin: Ja.) Die machen&schrauben die eine Weg, machen die nächste drauf, schicken die eine ein, die sag-&haben natürlich ‘ne Stabilität in ihrer äh Rehabilitation (Interviewerin: Mhm (bejahend)). Aber jemand, der dann nur eine Hand hat, der wartet dann einfach viel zu lang auch auf Reparaturen oder die Kostenträger kommen und sagen multiartikulierende Hand, ganz toll, (Name von Teilnehmer 3 aus Datenschutzgründen ausgelassen) hat’s gesagt, hat sicher funktionell in vielen Bereichen einfach auch Vorteile (Interviewerin: Ja.), aber der Nachteil und da kommen wir wieder an den Punkt: Stabilität, Robustheit. Das muss einfach kontinuierlich für den Patienten da sein. Egal ob über mehr Robustheit oder über höhere Stückzahl. (Interviewerin: Mhm (bejahend)) Aber das ist, finde ich, ganz wichtig. (Interviewerin: Ja.) #00:53:12-0#

Interviewerin: Ok. Ähm und wenn wir jetzt eben vielleicht grade noch explizit darauf gucken: Wir haben darüber gesprochen ähm, was die negativen Aspekte sind, was müsste man denn noch verbessern, was ist denn, ok wir haben jetzt eben die Wasserfestigkeit, dieses robust sein, aber was gibt’s noch, vielleicht an Funktionen, die man hinzufügen müsste, damit so Erwartungen noch besser getroffen werden. Oder auch bei der Ästhetik: Was gibt’s für Sachen, wo Sie, wenn Sie sagen würden, Sie sitzen da, weiß nicht, ganz oben an der Entwicklerfront und dürften die einfach umbauen, so wie Sie glauben, dass es für die Patienten einfach am besten ankommt, um alle Erwartungen zu erfüllen, was müsste man da noch verändern? #00:53:45-2#

Teilnehmer 27: (Name eines Prothesenherstellers aus Datenschutzgründen ausgelassen) hat mal ein oder er hat einen Entwicklungsschritt gemacht, den fand ich sehr gut. Es gab früher verschiedene Hände, heute gibt es nur noch über ‘ne Programmierung der Elektronik eine verschiedene Spezifikation für diese Hand. Mh ich war oft in Wien, in der Entwicklungsabteilung bei (Name eines Prothesenherstellers aus Datenschutzgründen ausgelassen) und hab da einigen Ingenieuren gesagt, ich würde mir zum Beispiel wenn’s um die Hand geht (Interviewerin: Mhm (bejahend)), würd‘ ich mir eine Hand wünschen aus Titanflex-Fingern, die beweglich sind, die verbiegen, aber nicht brechen (Interviewerin: Mhm (bejahend)) (Interviewerin: Ja.) und die auch zu&aufgrund der Materialität nicht gleich so verbiegen, dass sie nicht mehr nutzbar sind, sondern, dass sie wieder verwendbar sind. Das ganze endoskeletal. Das heißt es gibt ‘n weichen Mantel drüber, der mit ‘ner Hülle mh verkleidet wird, die relativ schnell austauschbar ist. Selber vom Patienten austauschbar ist. (Interviewerin: Mhm (bejahend), ja.) Und diese Hand kann ich programmieren in entweder sechs, sieben oder acht verschiedene Grifffunktionen oder auch nur zwei. (Interviewerin: Mhm

(bejahend)) Ja? Weil alles was da am Markt angeboten wird, äh mit zwölf verschiedenen Griffen und äh der Gestiksteuerung und was nicht alles (Interviewerin: Ja.). Das ist auch meines Erachtens nicht wirklich real, sondern jemand der sagt: „Ich geh morgens zur Arbeit“, Beispiel ‘n Kunde von uns hat ‘n Hotel, äh der sagt: „Ich muss da funktionieren. Die Gäste kommen, ich muss die Rechnung schreiben, ich muss Frühstück machen, ich muss auf ’n Markt fahren, ich muss einkaufen, und&und&und.“. Der braucht eine schnelle Hand (Interviewerin: Mhm (bejahend)) mit vielleicht zwei Griffen, aber zuverlässig (Interviewerin: Ja.). Und wenn der zuhause ist und jetzt mit seinem, er hat grad&ist grad Papa geworden, und seinem Kind die Windeln anzieht (Interviewerin: Mhm (bejahend)), dann geht er vielleicht an seine Programmierstecker und sagt: „Ich mach aus der Hand jetzt noch weitere drei Griffe, weil ich Karten spielen will heute Abend mit meiner Frau oder&oder.“. Also so eine variable Hand (Interviewerin: Mhm (bejahend)), ja? Die ich mal downgraden kann, für simple, schnelle, zuverlässige Sachen oder die ich auch über ‘n Programmstecker, ‘ne Bluetoothschnittstelle äh verändern kann. (Interviewerin: Ja.) Das wär‘ so mein&meine Vision einer Hand, Ro-&also Robustheit ganz im Vordergrund, Anpassbarkeit an unsere verschiedenen Tätigkeiten die wir machen wollen, leicht möglichst (Interviewerin: Mhm (bejahend)), ja? Und zäh. Nicht hart, sondern zäh. #00:56:22-2#

Interviewerin: Ja. Und die anderen? #00:56:24-2#

Teilnehmer 28: Das ist ‘ne gute Idee, das mit zwei Typen. (Interviewerin: (lacht)) Find ich cool, ja. Also, dass‘ dir programmieren kannst, so jetzt Arbeitsmodus oder Daddymodus. Des isch ‘ne gute Idee. #00:56:34-5#

Teilnehmer 29: Also ich denk mal vom mechanischen Aspekt her, was die Hände aufweisen müssen (Interviewerin: Mhm (bejahend)), wird’s anscheinend schwierig sein, wirklich so viel an Funktionalität reinzubringen äh wie man sich das wünscht (Interviewerin: Mhm (bejahend)) ähm weil es scheitert wirklich an der Robustheit der Hände. Also man sieht das jetzt an der Firma (Name eines Prothesenherstellers aus Datenschutzgründen ausgelassen), der es zum Beispiel nicht, anscheinend offensichtlich, wie man hört, nicht gelingt, die Michelangelo-Hand zu miniaturisieren, obwohl sie das mal vorhatten. Ähm man sieht das an der Störanfälligkeit, ähm dieser multifunktionalen Hände und ich glaub, manchmal ist es besser, einfach nur einfache Veränderungen zu machen, die: mechanisch nicht aufwendig sind und sicher sind und nicht zu viel sich zu verkünsteln. (Interviewerin: Mhm (bejahend)) Also im Prototypenstatus&Stadium hatte die Michelangelo-Hand auch zum Beispiel die Möglichkeit die Finger zu abduzieren, so dass man die wie beim Beten ineinander verschränken konnte (Interviewerin: Ja.). Ähm das mussten sie (einfach?) mal rausnehmen, weil ähm die Finger ständig gebrochen sind (Interviewerin: Mhm (bejahend)), so wie man gehört hat. Man kann natürlich vieles machen, was sehr schön ist, also von&von der Haptik her, fühlt sich sowas natürlich ganz toll an, wenn man die Finger verschränken kann, aber es muss halt funktionieren und das ist die Funktional-&die&die Defektunanfälligkeit das Wichtigere. ‘N schönes Beispiel für ‘ne einfache Modifikation ähm an einer Hand, die äh ziemlich gut war, aber technisch nicht anspruchsvoll äh is‘ bei der Michelangelo-Hand dieses flexible Handgelenk, ähm das man (nicht stark?) dabei hilft Kräfte zu entkoppeln, zum Beispiel beim Fahrradfahren, zum Beispiel beim Schieben eines Einkaufswagens und in vielen anderen Situationen. Da hat man was gemacht, was ähm ohne irgendwelche Antriebe funktioniert und ohne irgendwelche elektronischen Sperren funktioniert,

ähm aber was trotzdem ähm ‘n funktionellen Gewinn bringt. (Interviewerin: Ja.) Das find ich ‘n gutes Beispiel also was, dass was gelungen ist, wo man mehr Funktionalität, aber sehr simpel und dementsprechend robust reingebracht hat. (Interviewerin: Mhm (bejahend)) Ähm einfache Sache, aber es führt zu großer Zufriedenheit. (Interviewerin: Ja.) Das ist immer wieder so ‘n&so ‘ne Sache, wenn man Tests durchführt mit der Michelangelo Hand, wo ‘n großes Feedback von den Patienten her kommt, dass (Interviewerin: Mhm (bejahend)) es also ‘ne gute&‘ne gute Funktion ist, ‘ne? (Interviewerin: Mhm (bejahend)) Und ich glaube, viel&viel mechanischen Aufwand kann man nicht betreiben, sonst wird das Ganze zu störanfällig. (Interviewerin: Ja.) #00:58:49-9#

Interviewerin: Auch das was Sie vorher gesagt haben, einfach dieses, dass die Bauteile oft so groß sind und sowas, dass man nicht so richtig weiß, wo man die verpacken soll, ist das auch was, wo man sagen würde, wenn’s irgend ‘ne Möglichkeit gibt oder wie Sie gesagt haben (wendet sich an Teilnehmer 27): „Warum baut man’s nicht einfach woanders hin, dass des da oben nicht so schwer ist?“, wenn man da nochmal konkret eingeht auf dieses Gewicht oder auf diese Größe, wär das eben was, dass man sagt, man verändert das insofern, dass es irgendwie, weiß nicht, kleiner ist, woanders sitzt? #00:59:14-1#

Teilnehmer 29: Ich glaub wir können uns da viel wünschen, dass es möglichst klein wird (Interviewerin: Ja.), aber es ist natürlich auch Grenzen und da, (..?) grad in der Akkutechnik (Interviewerin: Ja.), da gibt’s zwar neue Technologien- #00:59:20-1#

Teilnehmer 27: Auch über die Stückzahlen da. (Teilnehmer 29: Ja, die Stückzahlen.) Also die Industrie sagt uns ganz klar, wir reden hier über einen Entwicklungsaufwand über Stückzahlen, die sind einfach lächerlich (Interviewerin: Mhm (bejahend)). #00:59:28-8#

Teilnehmer 29: Ein Marketing, also Marketing von (Name eines Prothesenherstellers aus Datenschutzgründen ausgelassen) hat mal gesagt, wenn man bei einem Batteriehersteller anruft und möchte von dem äh Akkus entwickelt haben mit ‘ner Stückzahl von zwei, drei, viertausend Stück, da muss man aufpassen, dass er nicht auflegt, ‘ne? Also die (..?) ganz erheblich größere Stückzahlen (Interviewerin: Mhm (bejahend)) und das größte Problem ist eigentlich, man kann nur Zellen verwenden, die für einen anderen Zweck entwickelt sind, kann die adaptieren und wenn die plötzlich mal vom Markt verschwinden und nicht mehr hergestellt werden, hat man ‘n tierisches Problem als Hersteller. (Interviewerin: Mhm (bejahend), klar.) Und auch als Orthopädietechnikbetrieb, der plötzlich halt vielleicht Ersatz benötigt (Interviewerin: Mhm (bejahend)) und diesen Ersatz in der vorgesehenen Weise nicht mehr bekommt. (Interviewerin: Mhm (bejahend)) #01:00:00-2#

Teilnehmer 27: Und das ist zunehmend ‘n größeres Problem auch, weil sich da einfach die Industrie zentralisiert über Zusammenschlüsse, Stückzahlen und so weiter (Interviewerin: Ja.). Es immer schwieriger wird natürlich, so individual oder kleine Serien überhaupt zu kriegen. #01:00:12-5#

Teilnehmer 29: Also ich glaub manche Komponenten lassen sich ganz gut äh miniaturisieren, also das sieht man bei Mikrocontrollern, aber bei Akkus ist es halt so, da gibt’s zwar Entwicklungen, da gibt’s äh Lithium Polymer Akkus, ähm da hatten wir jetzt welche seit neuerer Zeit, die ähm erstaunlich klein sind, bei großer Kapazität

(Interviewerin: Mhm (bejahend)) aber da kann man halt nicht beliebig was miniaturisieren (Interviewerin: Ja, klar.). Bei mechanischen Komponenten, die Kräfte übertragen müssen genauso wenig, bei Motoren wahrscheinlich auch nicht ohne weiteres, ‘ne? (Interviewerin: Mhm (bejahend)) Natürlich wünscht man sich das immer, es gibt vielleicht manche Aspekte, die so ‘n bisschen übersehen werden. Wir haben zum Beispiel Erfolg mit altmodischen Nickel Metallhydrid Akkus, ganz normalen Zellen (Interviewerin: Mhm (bejahend)), ähm die die erstmal wenn man sie in die Hand nimmt gar nicht flacher erscheinen, gar nicht kleiner erscheinen als das, was die Industrie bietet, aber wir können die so beliebig positionieren auf unsern Prothesen, dass wir damit auf einmal äh die besten Ergebnisse ähm haben und das mit ‘ner veralteten Akkutechnologie. (Interviewerin: Mhm (bejahend)) Äh wo wir auf einmal feststellen, warum kommt die Industrie nicht auf die Idee sowas zu fertigen, manchmal ist äh nicht das der neuere Schrei immer das Beste, ‘ne? (Interviewerin: Mhm (bejahend)) Ja. #01:01:26-6#

Teilnehmer 27: Wie sind wir in der Zeit? #01:01:25-5#

Protokollant: Gut. #01:01:28-3#

Interviewerin: Ja. Wir sind jetzt bei einer Stunde, wir haben nicht mehr <u>sehr</u> viel vor uns (lachend). #01:01:30-1#

Teilnehmer 27: Ok. #01:01:33-0#

Teilnehmer 29: Ok. #01:01:33-0#

Interviewerin: (wendet sich an Teilnehmer 2) Fällt Ihnen noch was dazu ein, zu den Dingen, die vielleicht-. Ja. Die verbessert werden können. #01:01:38-7#

Teilnehmer 28: Ich denk dass des zum einen manchmal so ‘n bisschen was neues sein könnte, so ‘n, einfach, dass man auch jetzt ein, es ist ja schon dabei, bei Oberschenkeln, aber implantierte Elektroden? #01:01:49-9#

Interviewerin: Mhm (bejahend) #01:01:48-1#

Teilnehmer 28: Und dass der ganze wegfällt mit Kabel, Elektroden auflegen und so weiter. (Interviewerin: Mhm (bejahend)) Des wäre ‘ne smarte Sache, wären dann auch weniger Passteile, weniger Komponenten. #01:02:03-3#

Teilnehmer 27: Ich erinner‘ mich an den Vortrag von (Name eines Arztes aus Datenschutzgründen ausgelassen), ähm weiß jetzt nicht genau wo ich ‘n ein-, 15 Jahre her. #01:02:14-8#

Teilnehmer 28: Mhm (bejahend) #01:02:14-8#

Teilnehmer 27: Implantierte Elektroden und vor zwei Jahren hab‘ ich den Vortrag von ihm wieder gehört (Interviewerin: Mhm (bejahend)) und er ist kein Millimeter weiter. #01:02:24-6#

Teilnehmer 28: Ja, aber da gab’s jetzt beim letzten Mal ‘n Vortrag mal, in Leibniz. #01:02:33-5#

Teilnehmer 27: Da wird was gemacht. #01:02:33-8#

Teilnehmer 28: Dieser Zusammenschluss den’s da gibt von (Name einer Firma aus Datenschutzgründen ausgelassen) und&und (Name eines Prothesenherstellers aus Datenschutzgründen ausgelassen) (Teilnehmer 27: Ja.) so Research und zufällig an der (Name einer Universität aus Datenschutzgründen ausgelassen) Universität ähm über&über implantierte Elektroden für Oberschenkel. Des find ich ist schon, für’s Bein zu haben fänd‘ ich des fancy. Du hast hier vorne ‘n Sensor im Bizeps und im Quadrizeps und dann erkennt der Schwung- und Standphase und du brauchst nicht mehr irgendwie viel denken. Und bei Armen, der ist&der ist dagegen? #01:03:19-3#

Teilnehmer 27: Er kennt des Genium ja auch. Also du kannst des extern denk ich lösen oder du kannst des implantieren, aber die Implantationssache ist sehr&sehr schwierig, ist sehr komplex aufgrund der ganzen Gesetzgebung (Interviewerin: Mhm (bejahend)) und ich weiß nicht, ob sich das durchsetzen wird, aber es ist&ich&ich seh‘ nur an dem, ich weiß diesen Vortrag hab ich vor über zehn Jahren gehört (Teilnehmer 28: Mhm (bejahend)) und hab ihn vor kurzer Zeit, ich weiß nicht mehr, vielleicht anderthalb, zwei Jahren wieder gehört (Teilnehmer 28: Ja.) und ich hab mir gedacht, an den Vortrag konnt‘ ich mich genau erinnern und wir sind genau an dem Punkt und eigentlich nicht weiter (Interviewerin: Mhm (bejahend)). Und klar, kannst du bei (Name eines Prothesenherstellers aus Datenschutzgründen ausgelassen) in die Entwicklungsabteilung gehen, dann siehst du auch Mustererkennung und was nicht alles äh was auch Wege aufzeichnet, aber so der Durchbruch, der ist da noch nicht. #01:03:57-2#

Interviewerin: Ja. Und dann noch eine andere Sache und zwar ist man ja auch dran, sowas wie Haptik zu ermöglichen in den Händen. Was halten Sie denn davon? Weil ich weiß, manche ähm Prothesenträger haben gesagt, sie fin-&also sie finden das ist gar nicht mal sowas wichtiges, andere finden das für sich total wichtig, dass es so ‘ne Möglichkeit gibt, grade für ähm Ohnhänder, die einfach an beiden Seiten amputiert sind, was ist denn Ihre Meinung dazu? #01:04:17-9#

Teilnehmer 27: Sehr wichtig. ‘N sehr toller Vorteil, ja? Grade wenn wir die multiartikulierenden Hände sehen, die weniger Griffkraft haben, (Interviewerin: Mhm (bejahend)) da spielt die Haptik ‘ne Riesenrolle. Das sehen wir, wir machen so Fingerkuppen aus Silikon auf die Hände und normalerweise sind die Hände gummiert und der Unterschied ist gigantisch zwischen allein diesen zwei verschiedenen Materialien. (Interviewerin: Ja.) Aber Haptik ist auch ‘ne Frage der Außenhaut, ja? Wie kommt der Patient mit seiner Prothesenhand in die Jacke, in sein Seidenfutter (Interviewerin: Genau, ja.), ja? Also, es macht ‘n sehr, sehr großen, wichtigen Aspekt, ja? Handschuh sowieso. (Interviewerin: Mhm (bejahend)) Silikone, die nicht so mechanisch belastbar sind, PVC Materialien, die belastbarer sind, aber eben verschmutzen (wenn man dann ‘ne?) schmutzige Hand hat, was auch furchtbar ist, also da ist auch noch die Welt offen. #01:05:17-0#

Interviewerin: Ja. #01:05:19-5#

Teilnehmer 27: Das muss viel, viel mehr gemacht werden. #01:05:18-8#

Interviewerin: Und aber Haptik eben in welchem Sinne? Eher, dass&dass man

Gelenkstellung, über die Stellung der Hand, eher eben darüber, dass man eben in den Mantel kommt, weil man einfach hier irgendein Gefühl hat, oder dass man dieses&diese Oberflächen, dass man weiß: Wo hab ich die Hand eigentlich grade, was macht die eigentlich grade? Was wäre da besonders relevant, was müsste man da besonders beachten? #01:05:37-6#

Teilnehmer 27: Ich glaub Positionierung der Hand, das erfährt ‘n Amputierter wie ‘n Blinder. Der schärft seine Sinne, da brauch ich keine Sensorik (Interviewerin: Mhm (bejahend)), die ihm sagt wo seine Hand ist (Interviewerin: Mhm (bejahend)), das weiß er selbst. Also mir beschreiben Patienten, wenn ich ‘n guten Schaft hab, sitz ich im Dunkeln im Auto und ich weiß wo mein Schaltknauf ist, ja? Wie wir das auch wissen, wenn ich jetzt meine Becher greife und meine Hand zumach‘, weiß ich auch wo der ist (Interviewerin: Ja.), ja? Und ich glaub das ist nicht der Punkt, aber äh Friktionsverbesserung, Haftung oder auch nicht Haftung (Interviewerin: Mhm (bejahend)), also intelligente Oberflächen, die aus Grund der Erfahrung einfach dem Amputierten Erleichterung schaffen. (Interviewerin: Mhm (bejahend)) Das heißt sowohl im Griff, einfach ‘ne Verbesserung schaffen, also auch in der Außenhaut, Gleitfähigkeit um in die Jacke zu kommen, oder in der Stabilität, oder in der Schmutzresistenz, oder sonstigem. Das ist ‘n wichtiger Punkt. #01:06:21-6#

Teilnehmer 29: Also was diese Positionierung von Händen anbetrifft, ist es wirklich so, wenn äh ‘n Patient ‘ne neue Prothese bekommt und die ist anders aufgebaut, ‘n bisschen länger, ‘n bisschen kürzer, das merken die sofort. Die brauchen erstmal ‘ne Zeit, um sich dran zu gewöhnen (Interviewerin: Mhm (bejahend)). Und wenn jemand ‘ne Doppeltausstattung bekommt, sollte die&sollten die nach Möglichkeit auch möglichst identisch sein, diese Prothesen. Also die&es ist schon erstaunlich, dass die Prothesen so genau, so gut ins Körperabbild integriert werden können, beziehungsweise, dass die äh Propriozeption in den&der verbleibenden Gliedmaße halt noch so gut ist (Interviewerin: Mhm (bejahend)), dass man trotzdem noch weiß: Wo ist die Prothesenhand jetzt? (Interviewerin: Ja.) #01:06:54-0#

(kurze Pause) #01:06:57-8#

Teilnehmer 28: Ich bin (..?) ehrlich, wenn ich ganz ehrlich bin (lachend), ich bin immer am Überlegen so, wie ist dieses Wort „Haptik“ richtig definiert? Ich hab‘ das schon mehrmals gehört und ich bin da immer wieder am Nachdenken, weil ich’s nicht weiß. #01:07:11-1#

Interviewerin: Ja- #01:07:14-1#

Teilnehmer 28: Was, was genau die Definition von „Haptik“, Hosen runter, ich muss passen. #01:07:17-8#

Interviewerin: Alles gut, gar kein Problem. Nee, vielleicht, aber vielleicht können wir das auch gemeinsam kurz in&in dem Bezug definieren. Im Endeffekt geht’s darum irgend ‘ne Art von Sensorik, darum irgend ‘ne Art von Gefühl da rein zu kriegen. Dass man halt sagt, dass man eben, mh ja auch&auch irgend ‘ne Möglichkeit hat mit dieser Prothese mh zu fühlen. Nicht nur diese Funktionen zu haben, sondern auch irgend ‘ne Art von Gefühl zurückzubekommen. Das ist eben so ‘n bisschen die Frage, die sich jetzt stellt, in welcher Art und Weise ist sowas überhaupt relevant, eben, dass ich, dass ich, ja es haben einige Teilnehmer eben gesagt, sie fänden des

wichtig, diese Gelenkstellung, weil sie das eben nicht fühlen können, vielleicht ist es dann diese Schaftsache, ähm aber vielleicht auch eben sowas, dass man halt irgend ‘ne Art von Sensorik zurückbekommt, dass ich irgendwas anfass‘ und weiß:
Ist das heiß oder ist das kalt, oder&oder welche Stellung hat die Hand grade, ist die offen oder ist die zu. Ja, in so ‘ne Richtung. #01:08:10-6#

Teilnehmer 29: Das ist wahrscheinlich unheimlich wichtig, wenn man sowas hätte, aber es ist die Frage: Wie kann man’s äh bewerkstelligen? (Interviewerin: Mhm (bejahend)) Ich mein, man hat ja wenn’s um sensorisches Feedback gibt&geht schon experimentiert mit Vibrationssignalen. Das geht vielleicht noch ganz gut, weil sich’s relativ gut oder ziemlich gut miniaturisieren lässt. (Interviewerin: Ja.) Man hat die ja auch eingebaut in&in äh Handis und nehmen mit Sicherheit da auch keinen großen Platz weg (Interviewerin: Mhm (bejahend)) ähm aber ich hab‘ zum Beispiel mal gehört, dass diese Vibrats-&die Empfindlichkeit gegenüber Vibration stark abnimmt (Interviewerin: Mhm (bejahend)). Das heißt man hat davon anfänglich ‘n Gewinn und der reduziert sich im Laufe der Zeit. (Interviewerin: Mhm (bejahend)) Und ähm also ich hab da Resultate gehört, die waren auf die Dauer nicht so zufriedenstellend, wie man sich das erhofft. (Interviewerin: Ja.) Da ist man am Anfang zufrieden, man hat schon ‘n Gewinn, aber auf die Dauer irgendwie nicht (Interviewerin: Mhm (bejahend)), weil diese Empfindlich-&diese Empfindlichkeit gegenüber Vibration halt abnimmt (Interviewerin: Ja.). Und ich weiß nicht, was es anstelle dessen geben kann. Ich hab‘ mal gehört, dass irgendwelche Leute, oder irgendwelche Wissenschaftler experimentiert haben mit ähm mit der Möglichkeit Druckimpulse zu geben (Interviewerin: Mhm (bejahend)), also irgendwelche Komponenten eingebaut haben, die eben Druck vermitteln. Ich weiß nicht welche Ergebnisse es da gegeben hat (Interviewerin: Mhm (bejahend)), es ist wahrscheinlich schwierig, ähm sowas auf rein mechanische Art und Weise irgendwie zu bewerkstelligen. (Interviewerin: Mhm (bejahend)) Ähm man hat auch mal darüber nachgedacht, irgendwelche Mikrochips zu implantieren. Es ist wahrscheinlich alles sehr schwierig. (Interviewerin: Mhm (bejahend)) Weiß nicht, was jetzt da für Ideen entwickelt wurden. ‘N Gewinn wäre es mit Sicherheit, also wichtig ist an einer gesunden Hand, dass Zusammenspiel zwischen&zwischen der Handfunktion, mit allen ihren Freiheitsgraden und der Handgelenksfunktion, sondern auch der&der Tastsinn ist unheimlich wichtig. (Interviewerin: Ja.) Ähm ja, schön wenn man den imitieren könnte. Ähm wie man hört gibt’s auch da positiven&ein anderen positiven Aspekt, nämlich für, für Phantomschmerzen, da sind in dem Moment wo man halt ‘ne Rückmeldung von&von der Hand bekommt, auch die Phantomschmerzen abnehmen können (Interviewerin: Mhm (bejahend)). Ich weiß nicht wie weit das mittlerweile als wissenschaftlich erwiesen an-&an-&anzusehen ist, das hat man auch gehört. Ähm es gäbe wahrscheinlich viele positive Aspekte davon (Interviewerin: Ja.). Aber ist natürlich die Frage: Wie kann man das technisch lösen. #01:10:15-3#

Interviewerin: Ja. Und aber wenn Sie sagen, sie glauben, also&oder Sie wissen, dass wenn der Schaft gut sitzt, dass&dass man eigentlich einfach weiß, was die Hand grade macht, was die Prothese grade macht, ist es dann&ist dieses Sensorische, dass ich eben einfach was fühlen kann, ist das dann wichtiger? Wenn man da so rangeht? #01:10:39-6#

Teilnehmer 29: Die Stumpfhaut ist so ‘n bisschen der Ersatz für den Tastsinn. (Interviewerin: Mhm (bejahend)). Wenn- #01:10:42-6#

Teilnehmer 27: Fragen Sie zwei Patienten. Den frisch Amputierten, der sagt Ihnen: „Das brauch ich unbedingt.“ (Interviewerin: Ja.), ohne Erfahrung und fragen Sie den, unseren 10 Jahre Doppelamputierten, der sagt: „Das lös ich anders.“ (Interviewerin: Ja.) Und dann ist die Frage, ist es vom technischen Aufwand, von der Stabilität, von der Funktionalität wirklich ein wichtiger Punkt (Interviewerin: Mhm (bejahend)), oder müssen wir erst andere wichtige Punkte erfüllen (Interviewerin: Mhm (bejahend)) und da glaub ich, ist er nicht auf Platz eins. #01:11:05-9#

Teilnehmer 29: Das glaub ich auch, (Interviewerin: Ok.) dass es nicht auf Platz eins ist. Ich seh‘ da auch&kann mir keine richtige technische Lösung vorstellen, auch wenn’s von Gewinn wäre, theoretisch, es wird halt schwierig zu s-&sein das äh umzusetzen (Interviewerin: Mhm (bejahend)). #01:11:19-7#

Teilnehmer 28: Was würd’s ‘n bringen, frag ich jetzt mal, wenn der Patienten ‘n Glas greift mit der Prothese, (merkt?): Ok, die Cola ist kalt, oder der Kaffee ist heiß? #01:11:24-9#

Interviewerin: Mhm (bejahend) #01:11:27-1#

Teilnehmer 29: Das muss jemand- #01:11:28-7#

Teilnehmer 27: Für ’n Blinden, wär‘ das wichtig. #01:11:29-0#

Teilnehmer 28: Für ’n Blinden vielleicht, ja. Aber für ‘n Amputierten- #01:11:35-5#

Teilnehmer 27: Ja, aber für jemanden der mit Augenlicht erkennt, der Kaffee raucht, oder- #01:11:35-5#

Teilnehmer 28: Ja. #01:11:35-2#

Teilnehmer 27: Da schwimmt ‘n Stück Eiswürfel drin, der (Teilnehmer 28: Müsst’s hinkriegen.) m-&muss diese Information gerade nicht haben. (Teilnehmer 28: Mhm (bejahend)). (Interviewerin: Ja.) #01:11:40-4#

Teilnehmer 29: Wenn man ‘n gutes Prothesentraining gibt, dann weist man den Patienten eigentlich darauf hin, dass man nicht wie bei einer gesunden Hand äh merkt, die Wärmeabstrahlung von der Tasse (Interviewerin: Mhm (bejahend)) und das eine Prothesenhand halt nicht registriert und, dass man dementsprechend vorsichtig sein soll, wenn man das zum Mund führt und damit trinkt. (Interviewerin: Mhm (bejahend)) Wenn man überhaupt mit der Prothesenseite trinkt ähm. (kurze Pause) Also die Leute kompensieren das auf andre Art und Weise. Die wissen halt, äh Gegenstände sind heiß, Gegenstände sind kalt. Ich glaub das Wärmeempfinden ist nicht unbedingt das Wichtigste, das wäre eher das Empfinden einer Griffkraft, die man aufbaut bei hantieren mit empfindlichen Gegenständen (Interviewerin: Ja.). Ähm das wäre schön oder beziehungsweise noch wichtiger, wenn man merkt, dass ‘n Gegenstand anfängt zu rutschen. (Interviewerin: Mhm (bejahend)) Und das wirklich hinzukriegen mit Sensoren die zuverlässig funktionieren, wir hatten ja mal ‘ne SensorHand Speed, beziehungsweise die ist nach wie vor, soweit ich weiß, noch auf ’m Markt, wo ständig die Sensorik kaputt war. (Interviewerin: Mhm (bejahend)) Und äh die Hand auf einmal Automatismen, die Hand hat automatisch nachgegriffen, wenn ‘n Gegenstand gerutscht ist, die hat Automastismen entwickelt, die auch viele

Patienten gestört hat. (Interviewerin: Mhm (bejahend)) Also man ist da nicht wirklich weitergekommen (Interviewerin: Mhm (bejahend)) und keine Ahnung ob's da technische Lösungen geben kann, ich glaub's eher nicht (lachend). (Interviewerin: Mhm (bejahend)) #01:12:51-3#

Interviewerin: Aber das heißt eben, einerseits das, dass ich, wenn ich was greif, dass ich weiß, weiß nicht, wie weit oder wie fest muss ich zugreifen, ich stellt mir immer 'n rohes Ei vor (Teilnehmer 29: Mhm (bejahend)) einfach, dass man da so diese Feinheit hat und andererseits aber einfach, wenn ich, weiß nicht, 'n Teller oder so trag, dass ich halt merk: Sitzt der da noch gut? (..?) dass das schon was wär, was 'n&was 'n Vorteil wäre, was sinnvoll wäre? #01:13:08-7#

Teilnehmer 29: Theoretisch schon, ja. (Interviewerin: Mhm (bejahend)) #01:13:10-6#

Teilnehmer 27: Also so wie (Name von Teilnehmer 3 aus Datenschutzgründen ausgelassen) sagt, ja? (Interviewerin: Ja.) Wir haben, äh ich zeig Ihnen nachher noch 'n Film, wenn Sie möchten (Interviewerin: Mhm (bejahend), gerne (lachend)) von dem Doppelamputierten, da sieht man wie er 'n Ei greift und wie lässig er das macht (Interviewerin: Mhm (bejahend)). Ähm der beschreibt zum Beispiel diese Sensorhand, die hatte er, die also registriert (Interviewerin: Ja.), dass 'n Gegenstand in den Fingern rutscht, dann greift die Hand automatisch nach, ja? Das Beispiel was er mir erklärt hat war: Er steht auf einem Sektempfang, hat sein Sektglas da drin und das Sektglas pendelt 'n bisschen, wie so 'n&wie so 'n Kielschiff (Interviewerin: Mhm (bejahend)) und es reibt an diesem Sensor und die Hand macht zu und das Glas zerknallt (Interviewerin: Mhm (bejahend)). Oder er nimmt sein Enkel in die Hand, so auf den Arm, und der sitzt oben bei ihm in den Händen und der zappelt und die Hand drückt zu. (Interviewerin: Mhm (bejahend)) So. Wo unterscheide ich jetzt, wann ich diesen Sensor haben will und wann nicht? (Interviewerin: Mhm (bejahend)) Wenn wir <u>so</u> intelligente Prothesen bauen, dann können wir wieder darüber nachdenken, aber vorher ist es ein Nonsens (Interviewerin: Ja.) in meiner en-&in meinem Empfinden ein Sensor irgendwo zu positionieren (Interviewerin: Mhm (bejahend)), der anfällig ist, der das Gesamtbild der Prothese herunterfährt und der in einem kleinen Minifokus äh eine Sache erfüllen soll, wo ich einfach sag, aufgrund dieser Erklärung von dem Patienten sag ich mir (Interviewerin: Ja.), die Ingenieure haben ihre Hausaufgaben nicht gemacht, die haben kein Pflichtenheft gemacht (Interviewerin: Mhm (bejahend)), was nämlich auch passieren kann. Wenn das sauber gemacht ist, dann kann man darüber nachdenken. (Interviewerin: Ja.) Aber wir kommen <u>wieder</u> an den Punkt technisch zuverlässig, stabil <u>muss</u> gewährleistet sein. (Interviewerin: Ja.) #01:14:30-5#

Interviewerin: Das wär' nämlich meine nächste und eigentlich auch letzte Frage, dass&dass man, das heißt, dann kommt man eigentlich an den Punkt, dass man sagt, es ist eben wichtig, dass diese Grundvoraussetzungen, wo wir vorher dabei waren, dieses, dass es eben robust ist, dass die einfach zuverlässig ist, dass sie das macht was sie soll, dass das die erste Priorität ist und solche Sachen, grade weil sie einfach oft dann auch Nachteile mitbringen, gar nicht mal so wichtig sind. Gibt's da noch andere Sachen, wo Sie jetzt sagen würden ähm das muss gar nicht explizit verbessert werden, es soll genau so bleiben. Entweder weil's einfach schon super gut ist, oder des&des bringt einfach Nachteile mit sich, so wie wir jetzt diese Sensorik haben, dieses dass irgendwas verrutscht und es aber einfach auch zu oft nicht funktioniert, dass eben da der Vorteil nicht mehr groß genug ist, als dass man

diese Nachteile weglässt. Dass Sie einfach sagen, es gibt Sachen, wo man einfach sagen würde: Die lohnt sich’s nicht zu verbessern, weil die gut sind oder weil die nicht genug Vorteile bringen? #01:15:24-2#

Teilnehmer 27: Zu verbessern lohnt sich’s immer (Interviewerin: Mhm (bejahend)). Also alle Hände die wir da haben, sind verbesserungswürdig. (Interviewerin: Ja.) Da gibt’s nichts, was nicht verbesserungsfähig ist. Ähm, wie gesagt, nochmal: (Interviewerin: Mhm (bejahend)) Stabilität. Ich kenne Patienten, die von einer (Name eines Prothesenherstellers aus Datenschutzgründen ausgelassen) Sensor Hand, oder&oder VariPlus Hand, schnelle Griffgeschwindigkeit, was ‘n rie:sen Vorteil ist, was alle Multiartikulierenden nicht schaffen, äh umgestellt waren auf multiartikulierende Hände, technische Defekte hatten, die technischen Defekte zu häufigen Ausfällen ge-&und die Akzeptanz der Prothesennutzung runterging (Interviewerin: Mhm (bejahend)). Dann kann ich sagen, ich kann ‘n technisches, hoch entwickeltes Produkt machen, was dann Ausfallerscheinungen hat. Wenn mein Auto ständig ausfällt, werd ich das auch nicht mehr sehr gern fahren, weil ich sag: „Wenn ich jetzt fahr, dann ist es morgen wieder kaputt, oder in drei Wochen.“ Also nochmal, diese technische Stabilität die wird, finde ich, bei allen Entwicklungen einfach völlig unterschätzt. (Interviewerin: Mhm (bejahend)) #01:16:11-3#

Teilnehmer 29: Man muss ja bedenken, die Leute haben ja auch ihre Alltagssorgen, ihre Alltagsaufgaben zu bewältigen u:nd ähm teilweise sind sie darauf s-&hochgradig angewiesen, auf ihre Prothese, dann ist es sehr ärgerlich, wenn’s ausfällt und selbst wenn sie nicht so hochgradig drauf angewiesen sind, ist es lästig äh mit der Prothese, die nicht funktioniert zu uns vorbeizukommen oder die erstmal versandfertig zu machen, zu uns zu schicken und dann äh hat man Streit mit ’m Kostenträger vielleicht und ähm muss längere Zeit darauf verzichten. Man hat immer Arbeit damit (Interviewerin: Mhm (bejahend)) und diese Arbeit möchte man sich ersparen. Man möcht sich auch morgens ins Auto setzen und es muss funktionieren (Interviewerin: Ja.) Man ärgert sich darüber, würde sich schon darüber ärgern, wenn’s nur zwei oder drei Mal im Jahr kaputt ist und äh (Interviewerin: Mhm (bejahend)) diese multiartikulierenden Hände bei aktiven Prothesennutzern die sind durchaus zwei bis drei Mal im Jahr- #01:17:05-1#

Teilnehmer 27: Wenn’s gut geht. #01:17:05-6#

Teilnehmer 29: Jo. Kaputt. (Interviewerin: Mhm (bejahend)) #01:17:05-5#

Teilnehmer 27: Eher fünf bis acht. #01:17:09-1#

Teilnehmer 29: (Lachen). Ja. Und das ist halt kein Zustand für die. Dann verzichten die lieber auf ‘nen funktionellen Gewinn und sagen ich will das, was wirklich zuverlässig arbeitet und v-&in vielen anderen Aspekten sogar besser funktioniert. Griffgeschwindigkeit hört man immer wieder von (Interviewerin: Mhm (bejahend)) Prothesenanwendern. Griffkraft ist genau dasselbe ähm was mir im Verhältnis zur Michelangelo-Hand aufgefallen ist: Die Michelangelo-Hand ist von allen modernen Prothesenhänden die einzige, die ‘n Dreipunktgriff einigermaßen hinkriegt. (Interviewerin: Mhm (bejahend)) Weil die genügend Griffkraft hat und genügen starke Finger hat. Ähm. #01:17:40-8#

Protokollant: (zeigt den Dreipunktgriff mit seinen Fingern) Das? #01:17:40-1#

Teilnehmer 27: Mhm (bejahend) #01:17:43-0#

Teilnehmer 29: Ähm. Diesen Dreipunktgriff, genau. Das hat sich immer wieder gezeigt, wenn man Tests macht mit dieser Prothesenhand und die äh Anwender fragt, was die denn besser kann, dann kristallisiert sich sowas immer wieder raus und da denkt kein anderer Hersteller dran. (Interviewerin: Mhm (bejahend)) Ja. #01:17:55-0#

Interviewerin: Fällt Ihnen dazu noch was ein, Herr (Name von Teilnehmer 28 aus Datenschutzgründen ausgelassen)? #01:17:54-9#

Teilnehmer 28: (..???) wenn man, (..?) klar überall gibt’s das After-Sales-Service, wird natürlich alles gemessen, (..?) inzwischen auch was kommt zurück. Prozentzahl von Reklamationen. (Interviewerin: Mhm (bejahend)) Und die Myosachen die haben da schon ‘ne relativ hohe Ziffer (Interviewerin: Ja.). Also teilweise 15 Prozent für ‘n Produkt. (..?) im Autohaus, oder ‘ne Autofirma verkauft ‘n Auto und jedes zweite kommt zurück. (Interviewerin: Mhm (bejahend)) Hm. #01:18:22-3#

Interviewerin: Ja, ok. Dann sind wir tatsächlich ähm auch schon soweit durch. Ähm- #01:18:33-1#

Teilnehmer 27: Es wird jetzt anders. #01:18:34-1#

Teilnehmer 28: Wie? (alle lachen) #01:18:36-8#

Teilnehmer 27: Dank euch (lachend) wird das jetzt anders. Es wird die zu(verlässigere?) Hand konzipiert. #01:18:40-8#

Interviewerin: Einmal nochmal ganz kurz, zur Zusammenfassung: Wir haben über positive und negative Aspekte gesprochen, die Ihnen rückgemeldet werden von Patienten, auch darüber eben was bei der Anpassung wichtig ist, was da für Schwierigkeiten sind, was man vielleicht verbessern sollte, was man nicht verbessern sollte oder wo einfach, wo man auf diese Basispunkte, die einem wichtig sind zum-&äh zurückkommen sollte. Mh gibt’s noch irgendwas, was Sie ergänzen würden, was vielleicht auch vorher mal dran war, wo Sie jetzt nochmal sagen, da ist Ihnen noch irgendwas dazu eingefallen, das wär‘ noch wichtig? Oder gibt’s was, wo Sie sagen, das liegt Ihnen nochmal so am Herzen, dass man das vielleicht mitgibt als Feedback, was Sie nochmal unterstreichen wollen oder, ja. #01:19:24-8#

Teilnehmer 27: Also, wenn ich aus dem Gespräch meinen Wunschzettel (Interviewerin: Ja.) formulieren müsste, dann wär das eine, eine 3D gescannte Hand (Interviewerin: Mhm (bejahend)), eine&also eine Kopie der vorhandenen Hand, die auf ein modulares Skelett gesetzt wird. (Interviewerin: Mhm (bejahend)) So, das. Und dann eben wie gesagt, wie ich vorhin gesagt habe, mit verschiedenen äh programmierbaren, vom Patienten einfach programmierbaren, verschiedenen Funktionen. (Interviewerin: Ja.) Mit ‘nem Handgelenk, nicht zu schwer, aber die Robustheit im Vordergrund. (Interviewerin: Mhm (bejahend)) Und vielleicht auch noch dem Zusatz, dass ich sagen kann: Ich kann diese Hand mit ‘ner Kosmetik tragen oder ich kann diese Hand mit ‘ner futuristischen Außenhülle nutzen. (Interviewerin: Ja.) #01:20:10-1#

Teilnehmer 28: Setz mo‘ noch eins drauf, oder? Wasserdicht. (alle lachen) Und&und mit äh::: Solarzellen. #01:20:21-3#

Teilnehmer 27: Selbstregenerierbarer Oberfläche wie unsere natürliche Haut (Teilnehmer 28: Ja, genau.) (lachend). #01:20:25-1#

Teilnehmer 28: Wachs-&selbstwachsende Fingernägel. (alle lachen) Jo. (Teilnehmer 29: Ich glaub was wir nicht-) Ich hab‘ nichts mehr hinzuzufügen. #01:20:34-0#

Teilnehmer 29: Was wir nicht erwähnt haben, ist die Kompatibilität mit vorhandenen Systemen, da hatten wir auch schon das Problem mit der Michelangelo-Hand (Interviewerin: Mhm (bejahend)), dass es nicht kompatibel war mit den bisherigen Systemen, dass man wirklich- #01:20:44-6#

Teilnehmer 27: Ja, ist auch ‘n wichtiger Punkt, (Teilnehmer 29: Ja.) ganz wichtiger Punkt. Dass man, wenn jemand&wenn es verschiedene Handkomponenten gibt, dass man die auf einem Schaft&oder in einem System nutzen kann. (Interviewerin: Mhm (bejahend), ja.) #01:20:53-2#

Teilnehmer 29: Ich glaub selbst Kompatibilität mit ‘ner reinen Habitushand kann nichts schaden, wenn man das auch&wir&wir können das machen, aber da müssen wir bauen, äh eigenen Adapter bauen und so weiter. Also wenn man da irgendwie schnell ‘ne normale Habitushand draufmachen kann, kann manchmal ‘n Vorteil sein. Ist aber eher zweitrangig (Interviewerin: Mhm (bejahend)) also, wichtig ist die&zwischen den Handgelenksanschlüssen die Kompatibilität mit dem, was es sonst noch gibt, Greifer und elektrische Hooks. (Interviewerin: Ja.) #01:21:18-8#

Interviewerin: Gibt’s noch was, was jemand hinzufügen möchte (lachend)? #01:21:20-4#

(kurze Pause) #01:21:22-4#

Teilnehmer 28: Nö. #01:21:23-1#

Interviewerin: Ähm ja, dann sind wir durch, ich mach das Mikrofon aus.
