# Supplementary material for: What do users and their aiding professionals want from future devices in upper limb prosthetics? A focus group study
Source: PLoS One. 2023 Dec 29;18(12):e0295516. doi: 10.1371/journal.pone.0295516 (PMC10756510; doi:10.1371/journal.pone.0295516)
Supplement: S1 Appendix — (ZIP) [file pone.0295516.s001.zip › FocusGroup_Transcripts/FGP5.pdf]

Interviewerin: Die Mikrofone jetzt an. Ähm ich würd‘ äh bitte, wenn ich die erste Frage stell, dass wir die erstmal der Reihe nach beantworten und, dass Sie sich ganz kurz vorstellen mit Vor- und Nachnamen, damit wir einfach die Stimme so ’n bisschen dem Namen später zuordnen können. Genau. Ähm dann würd‘ ich Sie zum Einstieg bitten sich vorzustellen ähm wenn Sie ‘ne Person treffen, die eine Prothese trägt, was Ihnen da als erstes auffällt, auf was die als erste achten. #00:00:34-0#

Teilnehmer 15: Ja, mein Name ist (Name von Teilnehmer 15 aus Datenschutzgründen ausgelassen) (Interviewerin: Mhm (bejahend)) mh ich glaub es, am ehesten würden mir das Gesamterscheinungsbild ähm, da würd ich glaub ich kategorisieren, weniger auf Details. #00:00:48-5#

Interviewerin: Mhm (bejahend) und spielt für Sie aber die Prothese in dem Gesamterscheinungsbilde ‘ne Rolle, fällt die auf? #00:00:55-2#

Teilnehmer 15: Mir mehr als anderen, ja. #00:00:55-7#

Interviewerin: Mhm (bejahend) #00:00:56-5#

Teilnehmer 15: Mir würde es eher auffallen als äh dem Bekanntenkreis, ja. #00:00:59-4#

Interviewerin: Und können Sie da sagen was Ihnen da auffällt, auf was Sie da speziell achten? #00:01:02-2#

Teilnehmer 15: Proportionen. #00:01:03-3#

Interviewerin: Mhm (bejahend). #00:01:03-3#

Teilnehmer 15: Mir fallen meist äh unkorrekte Proportionen auf. (lacht) Warum auch immer. #00:01:07-6#

Interviewerin: Mhm (bejahend), ja, ok. Und gibt’s dann sowas, dass Sie irgendwie drauf achten was die kann, was Ihre vielleicht kann oder irgendwas was da- #00:01:13-5#

Teilnehmer 15: Nee. #00:01:15-4#

Interviewerin: Gar nicht? #00:01:13-5#

Interviewerin: Eigentlich überhaupt nicht. #00:01:18-2#

Interviewerin: Mhm (bejahend) #00:01:19-0#

Teilnehmer 15: Äh ich habe eigentlich die besten Erfahrungen auch im Beruf, ich bin Handwerker, (Interviewerin: Mhm (bejahend)) gemacht mit ähm rein Mechanischen (Interviewerin: Mhm (bejahend)). Ähm ich hatte eine Elektrische, also keine Myoelektrische, sondern Elektrische über Bowdenzug (Interviewerin: Mhm (bejahend)) und ähm weil ich mich wahrscheinlich sehr auf die Mechanische eingeschossen habe mh bin ich damit ganz gut klargekommen. #00:01:43-2#

Interviewerin: Ja. #00:01:44-4#

Teilnehmer 15: Mit der Elekt-&mit der Mechanischen. #00:01:46-4#

Interviewerin: Ok, das heißt es gibt gar nicht so irgendwie die Neugier, ob da irgendwer vielleicht was Neues- #00:01:50-8#

Teilnehmer 15: Doch, sehr, sehr! Aber ähm hab‘ ich kein Kontakt äh zu, bis jetzt. #00:01:53-3#

Interviewerin: Ja, ok. #00:01:55-7#

Teilnehmer 15: Also vor 34 Jahren mal, aber jetzt nicht mehr. #00:01:55-8#

Interviewerin: Mhm (bejahend). Aber, also wenn man dann so jemanden sieht, ist man da so ’n bisschen neugierig drauf ob der irgendwas hat, was vielleicht neuer ist, was nochmal anders funktioniert, oder ist das eben einfach nur: Wie passt das ins Körperbild, wie erscheint der? #00:02:09-1#

Teilnehmer 15: Nee, also das ist natürlich schon ‘ne Frage. Ich hab‘ mich glaub ich aber ähm in den letzten 20 Jahren nicht gefragt, ob <u>ich</u> dafür in Frage käme. Also ich hab‘ das nicht auf mich bezogen. #00:02:18-2#

Interviewerin: Mhm (bejahend), ok. #00:02:19-1#

Teilnehmer 15: Ähm ja, ich hab‘ mich jetzt 34 Jahre an diese Art der Prothese gewöhnt, ich hab, ich bin eigentlich ausschließlich mit Prothese von aufstehen bis ins Bett wieder und setze die auch ähm komplett ein am Tag. #00:02:35-9#

Interviewerin: Mhm (bejahend). Und wenn Sie sagen Sie haben das nicht auf sich bezogen, also nicht sich gefragt, ob das dann für Sie in Frage kommt, woran liegt das? Weil Sie einfach zufrieden sind wie’s ist? #00:02:44-7#

Teilnehmer 15: Na, vor 34 Jahren wurde, wurde mit, wurde myoelektrisch getestet. (Interviewerin: Ja.) Da wurde gesagt, dass bei mir die Muskelkraft nicht ausreicht. (Interviewerin: Mhm (bejahend)) Nun ist der Stand vor 34 Jahren möglicherweise ‘n ganz anderer als heute ähm deswegen hab ich nie darüber nachgedacht (Interviewerin: Mhm (bejahend)). #00:03:01-7#

Interviewerin: Ok, das heißt Sie haben’s für sich einfach ausgeschlossen, weil halt, weil man gesagt hat es passt nicht? #00:03:03-9#

Teilnehmer 15: Ja. #00:03:05-3#

Interviewerin: Mhm (bejahend), ok. Und bei Ihnen, Herr (Name von Teilnehmer 25 aus Datenschutzgründen ausgelassen)? #00:03:07-4#

Teilnehmer 25: Ja, also mein Name ist (Name von Teilnehmer 25 aus Datenschutzgründen ausgelassen) ähm und mir fällt jetzt direkt ähm auch bei Herrn (Name von Teilnehmer 15 aus Datenschutzgründen ausgelassen) dass ähm, ich hab hier eben auch schon die Hand gesehen, dass es ‘ne (Name eines

Prothesenherstellers aus Datenschutzgründen ausgelassen) ist, die einfach nur auf- und zugehen kann, also ich find nach meiner Meinung ist eigentlich ‘ne Hand, die schwerer ist als ‘ne Schmuckhand, die man eigentlich zu fas-&fast kaum Sachen verwenden kann, dann seh‘ ich auch, dass ähm Herrn (Name von Teilnehmer 15 aus Datenschutzgründen ausgelassen) den ähm, kein Ellenbogen hat und wahrscheinlich auch ‘n Unfall hatte? Ähm und ähm ich glaube, er benutzt die Prothese hauptsächlich auch dafür, dass ähm er eben halt das Ungleichgewicht ausbalancieren kann. #00:03:48-5#

Teilnehmer 15: Auch, auch. Ja. #00:03:51-2#

Teilnehmer 25: Und ich glaube, ähm, dass Sie viel mehr mit der rechten Hand machen ähm als mit der linken Hand erstmal, weil’s eben halt ja eingeschränkt ist, weil Sie ja kein Gelenk mehr haben. Ähm keinen Ellenbogen (Teilnehmer 15: Mhm (bejahend)) und auch ähm eben halt wahrscheinlich ähm nur auf- und zumachen können. Ja, bei der Prothese (Teilnehmer 15: Ja.) ähm Drehmotor hat sie glaub ich ja nicht mit drin? #00:04:11-8#

Teilnehmer 15: Nee, nee. Das ist ähm- #00:04:13-4#

Teilnehmer 25: Nee. Ja. Ganz genau, da müsste man sie ja auch dann- #00:04:17-5#

Teilnehmer 15: Das Einzige was ich habe, ist die Glocke hier. #00:04:17-3#

Teilnehmer 25: Dann immer dann eben halt eben ja auch noch per manuell drehen. #00:04:21-5#

Teilnehmer 15: Ja. #00:04:22-0#

Interviewerin: Ok, ich seh‘, Sie beobachten sehr genau, wenn Sie jemanden mit ‘ner Prothese sehen. Ähm und, Sie kriegen ja jetzt im neuen Jahr auch Prothesen (Teilnehmer 25: Ja.) an beiden Händen. Ist das dann auch was, wo man drauf achtet: Was hat da jemand für ‘ne Prothese, könnt ich sowas auch haben? Gibt’s- #00:04:38-0#

Teilnehmer 25: Ähm eigentlich nicht, weil (Interviewerin: Mhm (bejahend)) man muss auch so sagen, die Neueste ist ja jetzt dann auch ja grad, die ich ja dann kriege, die ähm Touch Bionics (Interviewerin: Mhm (bejahend)) ähm also eigentlich gibt’s ja bislang ja noch nichts Höheres. Ähm ‘ne (Name eines Prothesenherstellers aus Datenschutzgründen ausgelassen) hab‘ ich Zuhause rumfliegen (Interviewerin: Mhm (bejahend)), kann man von mir aus, können Sie als Briefbeschwerer benutzen wenn Sie wollen, das ist meine persönliche Meinung dazu (Interviewerin: Mhm (bejahend)). Wiegt gefühlt ‘n Kilo ungefähr (Interviewerin: Mhm (bejahend)) also wenn man die hat und ist eigentlich unnötig ähm und ähm ja. Ähm was man dann natürlich mal so schaut, ich bin auch Betreuer beim Behindertencamp, (Interviewerin: Mhm (bejahend)) deswegen kenn ich auch ganz viele andere Behinderte und weiß eben halt auch bisschen dann eben halt über auch paar Schicksale oder auch eben halt wie die mit den Prothesen zurechtkommen dann oder was die für Prothesen haben. Und da weiß ich auch zum Teil was jetzt ähm bei der Bio-&ähm bei der ja, ist ja die Bebionics ähm dieses ähm andere, dass da zum

Beispiel man da&da keinen beweglichen Daumen ja hat, keinen manue-&äh elektrischen und dass da auch viel mehr Sachen immer kaputt gehen als bei der Touch Bionics. (Interviewerin: Mhm (bejahend)) #00:05:47-7#

Interviewerin: Ok. Dann würd‘ ich als nächstes gerne wissen, warum Sie sich für die Art der Prothese entschieden haben, die Sie tragen. Sie haben ja vorher schon gesagt, Herr (Name von Teilnehmer 15 aus Datenschutzgründen ausgelassen), Sie hatten davor ‘ne andere, Sie haben jetzt mit der gute Erfahrungen gemacht. Wenn Sie einmal sagen könnten warum das so ist. Und Sie Herr (Name von Teilnehmer 25 aus Datenschutzgründen ausgelassen) warum, also wie die Entscheidung kam zu der die jetzt kommt und warum überhaupt die Entscheidung jetzt dann ‘ne Prothese zu haben, wenn Sie darauf einmal antworten könnten. #00:06:14-3#

Teilnehmer 15: Ähm. Ich hatt‘ ‘n Verkehrsunfall mit ’m Motorrad (Interviewerin: Mhm (bejahend)) und der Arzt der mich danach behandelt hat, den hatte ich gebeten sich mal zu erkundigen, was aus seiner Sicht die bestmögliche Adresse in Deutschland wäre, die über die Krankenkasse mit abgedeckt wird und das war damals, wie er sagte (Name eines Ortes aus Datenschutzgründen ausgelassen). (Interviewerin: Mhm (bejahend)) Und nach einem Jahr bin ich dann nach (Name eines Ortes aus Datenschutzgründen ausgelassen) auch äh gefahren und ähm da wurde mir ganz am Anfang sofort gesagt, dass die, dass zu dieser Zeit die mechanischen Prothesen äh falls man wirklich den kompletten Tagesablauf damit bestreitet (Interviewerin: Mhm (bejahend)) so robust sind, dass die am ehesten ähm problemlos mitmachen. Darauf hab‘ ich mich damals verlassen (Interviewerin: Mhm (bejahend)) und dann sind hier bei (Name eines Sanitätshauses aus Datenschutzgründen ausgelassen) mal die elektrischen ähm Prothese gewesen ähm mit der ich nicht so gut klargekommen bin. (Interviewerin: Mhm (bejahend)) Ähm (kurze Pause) ich hatte zwei mechanische und zwar einmal war’s auch möglich die&das Ellbogengelenk über ’n Bowdenzug zu heben (Interviewerin: Mhm (bejahend)) ähm und die macht jetzt wirklich nur auf und zu. (Interviewerin: Mhm (bejahend)) Sonst nichts. Ähm ja, ich hab‘ mich glaub ich drauf eingestellt. (Interviewerin: Mhm (bejahend)) #00:07:34-5#

Interviewerin: Und woran lag das davor, mit der davor, mit dieser Elektrischen? Dass das eben nicht so hingehauen hat? #00:07:40-7#

Teilnehmer 15: Weil der Aufwand im Grunde genommen der gl-&äh viel größer war und der, das Resultat das gleiche. (Interviewerin: Mhm (bejahend)) Ähm, ob ich jetzt mit Bowdenzug öffne und schließe oder äh elektrisch (kurze Pause) ja, ich glaube das ist der einzige Unterschied. (Interviewerin: Mhm (bejahend)) #00:07:58-1#

Interviewerin: Und gab’s dann sowas, dass Sie dann irgendwann mal gesagt haben, weiß nicht, sich mal informieren was es anderes gibt und überlegen ob da noch irgendwas in Frage kommt oder haben Sie ei-. #00:08:09-2#

Teilnehmer 15: (schüttelt den Kopf) #00:08:07-3#

Interviewerin: Nee. Ok. #00:08:06-2#

Teilnehmer 15: Nee. (lacht) Warum auch immer. Nee. #00:08:08-9#

Interviewerin: Mhm (bejahend) #00:08:11-2#

Teilnehmer 15: Also ich wollte ähm wieder Motorrad fahren und hab da mit meinem Arzt äh diskutiert, ob es möglich wäre eine Prothese, die, bei der ich die Auf-Bewegung abschalten kann (Interviewerin: Mhm (bejahend)). Weil ich hab's probiert, aber ich bin beim Beschleunigen jedes Mal mit der Prothese rausgerutscht und das war nicht ganz witzig. (Interviewerin: Mhm (bejahend)) Ähm deswegen hab ich das ganze Thema geknickt ähm aber das war der einzige Moment, wo ich über ‘ne andere Prothese nachgedacht hatte (Interviewerin: Mhm (bejahend)). Ähm steh dem aber nach wie vor völlig offen, natürlich. #00:08:44-8#

Interviewerin: Mhm (bejahend), ok. Und bei Ihnen, Herr (Name von Teilnehmer 25 aus Datenschutzgründen ausgelassen)? #00:08:46-3#

Teilnehmer 25: Ja, ich hatte ganz früher mal eine, ähm also ganz früher hatt‘ ich eben halt ähm, erstmal ganz von vorne, also mir wurde nie aufgezwungen sozusagen Prothesen zu tragen (Interviewerin: Mhm (bejahend)), wird auch immer nicht ähm dann hatt‘ ich eben halt die ähm bislang waren meine ganzen Prothesen immer nur bei&für die rechte Hand, ich hatt‘ auch keine für die linke Hand, dann erstmal einen Bowdenzug mit dieser Greifzange vorne dran, die man ja kennt. Ähm davon hatte ich mehrere, wo ich eben halt noch kleiner war. Ähm dann glaub ich mit acht oder so hatt‘ ich dann meine erste myoelektrische Prothese von (Name eines Prothesenherstellers aus Datenschutzgründen ausgelassen), ähm diese Standardhand die auf und zu (Interviewerin: Mhm (bejahend)), wo man eben halt das Gefühl hat, dass die ‘n halbes&‘n Kilo wiegt oder weiß ich, ähm hab ich damit paar Mal benutzt und dann auch mich entschieden ok, ich leg sie in die Ecke-. Ähm erstmal von dem Gewicht her, ähm weil's halt eben enorm sch-&schwer ist und wirklich ‘n Vorteil war's nicht für mich, immer. Und jetzt ähm, ähm damals wo ich dann beim ersten Mal beim Camp war, war&wurd ich dann eben halt ähm, ist man dann eben halt auf das Thema Prothesen ganz viel mehr wieder eingegangen (Interviewerin: Mhm (bejahend)), sag ich jetzt mal auch. Und dann wurd‘ ich äh mal überredet ähm mir eben halt die Touch Bionics dann eben halt mal anzuschauen (Interviewerin: Mhm (bejahend)) und die ähm ich dacht mir: „Ok, warum denn nicht?“. Schaden tut's denn nicht und warum ich jetzt auch „ja“ gesagt hab, dass ich welche will, weil ich wahrscheinlich auch die zum Auto fahren leider brauche. (Interviewerin: Mhm (bejahend)) Ähm deswegen hab‘ ich dann gesagt: „Ja, warum denn nicht?“. Wenn dann schon, dann die ähm Beste nehmen, die es jetzt auf 'm Markt gibt, denn man muss ja auch in Zukunft denken. Wie sieht's ähm in 30 Jahren aus? (Interviewerin: Mhm (bejahend)) Jetzt machen meine Gelenke noch alles mit, jetzt kann ich noch alles so, aber wie sieht's in 30 Jahren aus? Macht das mein Rücken dann noch mit, auch wenn ich eben halt fünf Zentimeter nur irgendwie ausgleichen muss ähm von der Armlänge (Interviewerin: Mhm (bejahend)), ähm weil ich hab eben halt das Glück, dass mir beide Hände fehlen ja. Ähm oder ähm wie meine Gelenke dann (spielen?), ob die versteifen, was weiß ich. (Interviewerin: Mhm (bejahend)) Und ähm jetzt könnte ja, ähm dann könnte ja die Kasse sagen: „Sie brauchen ja die 40 Jahre keine Prothese, warum jetzt?“. Und dann sitz ich da eben halt mit 120.000 Euro dann eben halt, die ich dann bezahlen müsste. (Interviewerin: Mhm (bejahend)) Und so, ist die ja verpflichtet mir (...?) zu geben, weil bis 18 ist ähm die Krankenkasse, jede, verpflichtet ähm pro äh das Beste ähm mögliche eben halt zu bezahlen. Das ist eben halt ‘n in dem Fall, also für mich zumindestens pro Hand 60.000 eben halt, ohne Schäfte (Interviewerin: Mhm (bejahend)). Die Hände kosten

ja 120 schon insgesamt und ähm dann hab‘ ich eben halt ‘n Folgeanspruch, auch wenn ich sie dann eben halt 10 Jahre nicht benutze und dann erst wieder ‘ne Neue. Die Krankenkasse ist dann verpflichtet, weil ich dann eben halt ‘n Anspruch hab, ähm dass ich die eben halt schon mal hatte, dass die mir die dann auch schon wieder dann geben. #00:11:34-9#

Interviewerin: Mhm (bejahend). Ok, das heißt die Entscheidung so ‘n bisschen einerseits daraus, dass Sie bei anderen gesehen haben, was sie eigentlich kann und- #00:11:42-6#

Teilnehmer 25: Nee, das noch nicht. #00:11:41-9#

Interviewerin: Nee? #00:11:41-9#

Teilnehmer 25: Also mir wurde die ja nun wirklich nur vorgestellt so dann, ähm extern dann- #00:11:44-4#

Interviewerin: Und von wem? #00:11:46-7#

Teilnehmer 25: Ähm von der Firma (Interviewerin: Ah, ok!) ähm Touch Bionics wirklich dann vorgestellt, ja dann. #00:11:52-8#

Interviewerin: Ok, ja. Ok und dann zum Autofahren und so ’n bisschen halt mit Blick einfach auf die Zukunft, um da vorzusagen? #00:11:58-5#

Teilnehmer 25: Ja. #00:11:57-7#

Interviewerin: Ok, mhm (bejahend). Ähm und wenn Sie beide mal, also Sie einerseits Herr (Name von Teilnehmer 15 aus Datenschutzgründen ausgelassen) mal sammeln was für positive Aspekte Sie an Ihrer Prothese sehen, wo Sie sagen das sind Dinge, die&die super funktionieren ähm und Sie Herr (Name von Teilnehmer 25 aus Datenschutzgründen ausgelassen) wenn Sie vielleicht eben sagen könnten das, was Sie da gesehen haben an der Touch Bionic, was da so die positiven Punkte sind, die wo Sie sagen da probieren Sie’s nochmal, obwohl Sie mit der Myoelektrischen sagen, dass da eben nicht so gute Erfahrungen damit waren, genau. #00:12:27-4#

(kurze Pause) #00:12:29-0#

Teilnehmer 15: Das was ich an der Prothese die ich jetzt trage mag, ist das sie sehr robust ist. (Interviewerin: Mhm (bejahend)) Ähm ich könnte meinen Job nicht machen, wenn ich keine Prothese hätte, mir wurde damals im&in der (..?)klinik gesagt, dass äh es wenig Sinn machen würde sich ‘ne Prothese zu besorgen, die würde sehr viel Geld kosten, steht dann ausschließlich in der Ecke. (Interviewerin: Mhm (bejahend)) Ähm das ist in meinem Fall genau das Gegenteil gewesen (Interviewerin: Mhm (bejahend)), muss ich schon sagen. Ähm und ich glaube, ähm ich für mich, ähm bin äh was so die Selbstbetrachtung angeht durch die Prothese sicherlich auch einfacher klargekommen als ohne. Mh (seufzt) ja es sind einfach die vielen kleinen Festhaltesachen, ähm gegenhalten und solche, diese ganzen Dinge die ich mit der Prothese mache (Interviewerin: Mhm (bejahend)), die ich äh, die einfach eigentlich alternativlos sind. Also ich arbeite in einer Werkstatt und die ist

natürlich auch für mich auch so ’n bisschen ausgerichtet, (Interviewerin: Mhm (bejahend)) aber ohne Prothese wär’s schlecht, ganz schlecht. (Interviewerin: Mhm (bejahend)) Und es ist auch komischerweise so, dass es viele Kunden gar nicht mitkriegen, wenn ich bei Kunden bin. (Interviewerin: Mhm (bejahend)) Was äh ich gar nicht nachvollziehen kann (lacht), aber ist- #00:13:47-6#

Interviewerin: Also das was Sie am Anfang gesagt haben, Ihnen fällt an ‘ner Person schnell auf, dass&dass da ‘ne Prothese getragen wird und den andren Leuten gar nicht. #00:13:53-1#

Teilnehmer 15: Ja. #00:13:51-5#

Interviewerin: Mhm (bejahend). Und wenn wir jetzt aber nochmal sammeln in der Werkstatt, was das genau äh für Funktionen, oder für Tätigkeiten mit der Prothese sind, wo sie einfach ‘ne Unterstützung ist, wo sie hilft? #00:14:04-2#

Teilnehmer 15: Ja bei allem was ich festhalten und gegenhalten kann. (Interviewerin: Mhm (bejahend)) Ähm ich bin Klavierbaumeister und ich muss also auch stimmen (Interviewerin: Mhm (bejahend)), das kann ich mit der Prothese dank der modernen Software kann ich das äh, ähm (komatisch?), brauch ich also keine Alterna-äh keine äh&ähm, egal. Ähm dann hab‘ ich natürlich Werkbänke (Interviewerin: Mhm (bejahend)) ähm&ähm aber wenn ich zum Beispiel Hammerköpfe anleime, alles das was ich leime, muss ich zum Beispiel mit einer Hand nehmen, mit der andern Hand äh leimen, Knochenleim oder wie auch immer (Interviewerin: Ja.) ähm vorbereiten. Und das sind also, das sind die typischen Einsatz-ähm-bereiche von der Prothese. #00:14:44-7#

Interviewerin: Mhm (bejahend). Und im Alltag? Außerhalb des Berufs? #00:14:46-9#

Teilnehmer 15: Kaum. #00:14:50-0#

Interviewerin: Mhm (bejahend) #00:14:51-5#

Teilnehmer 15: Äh::m was sicherlich auch damit zu tun hat, dass meine Frau das von Anfang an mitgekriegt hat und wir die Ehe dann im Grunde genommen ‘n bisschen so ausgerichtet haben, dass viele Dinge die ich so machen würde von meiner Frau übernommen wurden. #00:15:08-0#

Interviewerin: Mhm (bejahend), das heißt kaum heißt, dass eigentlich so die Hauptfunktionen und die Vorteile sich zeigen im Beruf aber drumrum gar nicht so sehr? #00:15:17-7#

Teilnehmer 15: Dann würd‘ ich improvisieren. #00:15:19-6#

Interviewerin: Mhm (bejahend) #00:15:20-8#

Teilnehmer 15: Aber es ist, also ich, im Moment fällt mir nichts ein. Wahrscheinlich wegen 34 Jahren Gewohnheit, äh dass ich andere Dinge machen würde als festhalten (Interviewerin: Mhm (bejahend)), gegenhalten, (Interviewerin: Mhm (bejahend), ja.) ähm unterlegen für das-. Äh also, wenn ich im Urlaub schon einen Tag auf ‘n-&auf ‘ne Prothese verzichten wollte das ist für mich kein&keine

Alternative, das äh (Interviewerin: Ja.) komm ich irgendwie nicht so mit klar. (Interviewerin: Mhm (bejahend)) Hat sicherlich auch mit dem mangelnden Gewicht zu tun. (Interviewerin: Mhm (bejahend), ja.) Also mh ich hab‘ im Moment bis jetzt noch keinerlei Probleme mit ’m Rücken und ich fand eigentlich das&die äh das Gewicht der elektrischen Prothese insgesamt fand ich das hilfreich, ich fand es nur nicht hilfreich, dass es die Hand war die so schwer war. (Interviewerin: Mhm (bejahend)) Wenn die Akkus hier oben gesessen hätten und hätten die Schulter so ’n bisschen runtergezogen, das, das hätt ich positiver gefunden. (Interviewerin: Mhm (bejahend), ja.) Aber die Hand war, die ist tatsächlich ‘n bisschen arg schwer, ja. (Interviewerin: Mhm (bejahend)) Durch die Motoren. #00:16:11-0#

Interviewerin: Genau und wenn wir dabei nochmal bleiben mit der Elektrischen ähm Sie haben vorher schon gesagt, einfach der Vorzug jetzt der gegenüber der anderen ist, dass&dass es einfach weniger aufwändig ist (Teilnehmer 15: Ja.), gibt’s noch irgendwas wo Sie sagen da hatte die andere schon auch Vorteile, aber da ist es einfach einfacher? #00:16:28-1#

Teilnehmer 15: Vielleicht hab‘ ich sie auch nicht lang genug genutzt, um das äh wirklich abschließend zu sagen. (Interviewerin: Mhm (bejahend)) (kurze Pause) Sie hatte einige Vorteile die aber gar nicht mit der Elektrik zu tun hatten. Sie hatte, also das hier ist jetzt so ’n durchgehender Weichkunststoff und die andere hatte so ’n&so ‘ne Art Netz (Interviewerin: Mhm (bejahend)) und das war atmungsaktiver. Das war zum Beispiel ‘n Vorteil, der aber nicht mit der Elektrik zu tun hatte. #00:16:51-7#

Interviewerin: Das macht gar nichts, also einfach-. Genau. #00:16:55-4#

Teilnehmer 15: Ach so, ja. Ähm mir wurde dann später gesagt, dass die Verarbeitung sowieso teils äh unfreundlich ist (Interviewerin: Mhm (bejahend)), dass man das nicht mehr macht, aber ‘ne atmungsaktive Schale (Interviewerin: Mhm (bejahend)) find ich schon sehr viel besser. (Interviewerin: Mhm (bejahend)) Mh der Handschuh selbst hat sich enorm verbessert im Laufe der Jahrzehnte ähm das war früher so, wenn ich ‘ne frische Jeans anhatte, ‘ne neue Jeans dann war der blau und wurd‘ auch nie wieder äh, das ist jetzt anders. Also da, die wird immer noch blau (lacht), aber ähm das dauert alles sehr viel, es ist alles sehr (im Hintergrund?). #00:17:24-6#

Interviewerin: Ok, ja. #00:17:24-6#

Teilnehmer 15: Ja. #00:17:25-8#

Interviewerin: Ok. Und bei Ihnen Herr (Name von Teilnehmer 25 aus Datenschutzgründen ausgelassen)? #00:17:30-0#

Teilnehmer 25: Ähm könnt ich nochmal kurz die Anfangfrage? #00:17:32-6#

Interviewerin: Ja, genau. Also was sind so die positiven Punkte, die Sie an der Touch Bionic gesehen haben, wo Sie gesagt haben: „Das, also das überzeugt mich dazu die auszuprobieren.“ (Teilnehmer 25: mhm (bejahend)) und vielleicht auch so ’n bisschen mit Blick darauf, weil Sie ja gesagt haben die Myoelektrische die Sie früher getragen haben, hat Sie nicht so überzeugt, die ist als Briefbeschwerer in der Ecke. #00:17:51-3#

Teilnehmer 25: Ähm, ja. Ähm vor allem deswegen, weil man die wirklich nur auf- und zumachen konnte (Interviewerin: Mhm (bejahend)) ähm was man ja natürlich mit der anderen ja auch machen kann, nur dass die ja nicht nur die Haupt-ähm-funktion, sondern zum Beispiel auch, dass man was ich damals ähm noch gut in Erinnerung hab, ich hab mal irgendwie jemandem ähm die Hand damit geschüttelt und dann aus Versehen zu fest zugedrückt oder so, so dass dann wirklich seine Hand zu sehr gequetscht worden ist und dann, bis man die dann die ja dann wieder aufkriegt, das dauert ja auch ‘n kleinen Moment (Interviewerin: Mhm (bejahend)). Und die zum Beispiel, hat ja dann diese Sensoren, dass ich ja dann zum Beispiel auch ‘n rohes Ei erkenne und dann wirklich nur so fest zudrücken, dass man das rohe Ei auch hochheben ja kann (Interviewerin: Mhm (bejahend)). Das find ich ist erstmal ein großes Fortschritt ähm und natürlich auch, dass man eben halt zum Beispiel mit der Appsteuerung dann auch verschiedene Sachen zum Beispiel zeigen kann (Interviewerin: Mhm (bejahend)). Sei es allein jetzt mal äh der Stinkefinger oder was weiß ich (Interviewerin: Mhm (bejahend)) jetzt mal, ähm gesagt, also das ist wirklich was, einen vor allem als Jugendlicher so oder grundsätzlich wirklich so, aber und zu, es fehlt so. (Interviewerin: Mhm (bejahend)) Also dann ähm und auch ähm, dass der Daumen sich drehen kann (Interviewerin: Mhm (bejahend)) und was weiß ich und der&das hat ja auch die verschiedenen Griffmustern ja. (Interviewerin: Mhm (bejahend)) Dann ja, ob zum Beispiel mit Computer schreiben oder dann das Handy eben halt bedienen und so weiter. #00:19:10-7#

Interviewerin: Also einfach das, dass es mehr Funktionen hat als die davor? #00:19:13-8#

Teilnehmer 25: Ja. #00:19:15-3#

Interviewerin: Ok. Und haben Sie so ‘ne Prothese dann auch schon mal ausprobiert? #00:19:18-1#

Teilnehmer 25: Ähm nee, die Testphase wird jetzt dann bald kommen. #00:19:21-7#

Interviewerin: Ok, ja. Und an der Myoelektrischen, die Sie früher getragen haben, gab’s da aber irgendwas wo Sie sagen, da gab’s doch irgendwas, was eben positiv war, was eben doch irgend ‘nen Sinn gemacht hat? #00:19:33-0#

Teilnehmer 25: Nein. #00:19:35-1#

Interviewerin: Gar nicht? #00:19:35-1#

Teilnehmer 25: Nein, gar nicht. #00:19:36-3#

Interviewerin: Ok. #00:19:38-2#

Teilnehmer 25: Ich glaub es liegt daran auch, dass ich ja eben halt mit meinem Handicap so geboren bin (Interviewerin: Mhm (bejahend)) und ähm mir alles eben halt ohne Prothesen äh beigebracht hab (Interviewerin: Mhm (bejahend)) ähm was ich, glaub ich, auch im Endeffekt besser ist, (Interviewerin: Mhm (bejahend)) wenn man wirklich komplett frei ist sozusagen von Prothesen. Prothesen können ja ausfallen, oder was weiß ich und dann steht man eben halt da und kann nichts mehr

machen und ähm damals war’s also wirklich das Gewicht erstmal, ich konnte sie damals höchstens zwei Stunden am Stück tragen (Interviewerin: Mhm (bejahend)), ähm und danach war Schluss. Ähm- #00:20:12-1#

Interviewerin: Und, ich unterbrech‘ hier noch ganz kurz, das heißt danach war Schluss. Woran lag das? Hat das dann wehgetan oder war das- #00:20:16-2#

Teilnehmer 25: Ähm von dem Gewicht einfach, also es wurde zu anstrengend. #00:20:20-5#

Interviewerin: Mhm (bejahend) #00:20:20-5#

Teilnehmer 25: Weil wenn man sozusagen, ähm der Stumpf ist ja relativ kurz, ähm und dann wirklich die ganze Zeit so ’n schwer-&äh Gewicht heben muss dann sozusagen die ganze Zeit (Interviewerin: Mhm (bejahend)), das kann man ja auch nicht den ganzen Tag machen. #00:20:32-6#

Interviewerin: Ok, das heißt es war einfach, weiß nicht, wie dann Muskelkater oder anstrengend? #00:20:35-7#

Teilnehmer 25: Ganz genau. #00:20:34-1#

Interviewerin: Mhm (bejahend), ja. #00:20:36-6#

Teilnehmer 25: Also es war einfach zu anstrengend dann irgendwann auch geworden. Ja und sonst irgendwie, also ich fand ‘s hat damals irgendwie nicht viel gebracht, vielleicht auch weiß ich da nur eben halt eine hatte und dann eben halt mehr eingeschränkt dann war (Interviewerin: Mhm (bejahend)) und ich glaube, es ist aber auch, wenn ich ähm zwei gehabt hätte, ähm ist es ist was ganz anderes, ne Kopfsache, dass man dann eben halt die eine Hand dann nur nimmt und dann was greift oder so, weil man ist es ja gewöhnt, dass man ja dann eben halt beide Hände, dann immer nimmt, das ist ‘ne ganz große Kopfsache ja auch. #00:21:10-0#

Interviewerin: Mhm (bejahend), ja. Und Sie haben jetzt grade schon gesagt eben was an der anderen doof war, wenn wir da mal noch weitermachen und mal die negativen Aspekte ähm sammeln, auch bei Ihnen Herr (Name von Teilnehmer 15 aus Datenschutzgründen ausgelassen), was nicht so gut läuft und was denn verbessert werden müsste. Also an der Myoelektrischen das Gewicht, dann haben Sie gesagt, dass, dass es eben nur die eine Funktion hatte, gibt’s noch irgendwas wo Sie gesagt haben da wo Sie’s ausprobiert haben, das war nicht so, wie’s hätte sein sollen? #00:21:39-4#

Teilnehmer 25: Definitiv das, dass die Prothese nicht erkennt ähm was für ‘n Gegenstand sie in der Hand hat. (Interviewerin: Mhm (bejahend)) Also das irgendwie ‘n Sensor da drin ist, dass wenn man eben halt mal ‘ne Hand schüttelt oder was weiß ich, die dann nicht eben halt die volle Kraft verwendet. Pf. Und sonst. Was glaub ich jetzt auch heutzutage ähm notwendig ist, dass man mit irgendeinem Finger oder so ‘n Handy bedienen könnte. (Interviewerin: Mhm (bejahend)) #00:22:11-1#

Interviewerin: Ja, dass das einfach was wäre, was eigentlich notwendig wäre als Funktion? Und wenn Sie nochmal überlegen eben warum Sie sich bis jetzt in der

ganzen Zeit nicht für ‘ne Prothese entschieden haben, was&was es da vielleicht noch an Punkten gab, wo Sie gesagt haben: „Nee das passt an den Prothesen einfach nicht, deshalb funktioniert das auch so.“? #00:22:28-2#

Teilnehmer 25: Ähm hauptsächlich, weil sich nicht viel getan hat. #00:22:33-2#

Interviewerin: Mhm (bejahend) #00:22:31-5#

Teilnehmer 25: Also bis jetzt in den vier, fünf Jahren so. Also es gab ja eigentlich immer noch die (Name eines Prothesenherstellers aus Datenschutzgründen ausgelassen) (Interviewerin: Mhm (bejahend)), oder die (Name eines Prothesenherstellers aus Datenschutzgründen ausgelassen) ist ja immer noch die ähm Standardhand, die Meistverkaufte jetzt, da kam irgendwann glaub ich ja Touch Bionics, Bebionics und die ähm, ähm Michalangelo-Hand ja von (Name eines Prothesenherstellers aus Datenschutzgründen ausgelassen) (Interviewerin: Ja.) ja eigentlich relativ gleichmäßig raus so, haben sich glaub ich jetzt auch eigentlich alle von gleichen abgeschaut sozusagen, würd ich jetzt mal ausgehen (Interviewerin: Mhm (bejahend)). Weil jetzt viel mehr Funktionen hat die eine oder die andere jetzt nicht großartig schon. Natürlich, das sind alles drei unterschiedliche Hände, wenn man sie jetzt so vergleicht, aber als großes Thema gesehen eigentlich außer jetzt die Bebionics, die mal bisschen abseits gesehen, fast dieselbe Hand. (Interviewerin: Mhm (bejahend)) Ähm das eine. Und sonst, ähm pf. Hab‘ ich gar nicht dran gedacht, so. Also das Einzige wo ich dann wirklich wieder leicht in Bezug gekommen bin ist durch ’s Camp und auch ähm vor allem wegen des Autofahren. (Interviewerin: Mhm (bejahend)) Weil mir eben halt gesagt worden ist, ich muss wahrscheinlich ‘ne Prothese beim Autofahren tragen. Also da sagt der Gesetzgeber ähm was eigentlich auch komplett dumm ist, sag ich jetzt mal, nicht dass es ge-&ich will nicht, dass es geändert wird, weil wie die Gesetzgrundlage ist, ist eigentlich grad gut sogar ähm für uns, ähm ich bin nur verpflichtet eine Prothese zu tragen (Interviewerin: Mhm (bejahend)), nur die ist nicht mehr aktiv ins Fahrgeschehen einzusetzen. Das heißt, ich könnte zum Beispiel die rechte Hand rein-&reinstecken und auf ’s Armaturenbrett legen und alles mit der linken Hand machen (Interviewerin: Mhm (bejahend)) das wäre dann wiederum erlaubt. #00:24:05-0#

Interviewerin: Mhm (bejahend). Ok, das heißt das als ein großer Grund, aber dann auch nicht mal so, dass Sie sagen, da&da find ich da brauch ich die für, sondern da muss ich sie halt haben und dann- #00:24:15-5#

Teilnehmer 25: Ja. #00:24:17-2#

Interviewerin: Ok. Mhm (bejahend). Ok, und bei Ihnen Herr (Name von Teilnehmer 15 aus Datenschutzgründen ausgelassen)? #00:24:18-2#

Teilnehmer 15: Ja, ich hätte gerne ‘ne zerlegbare Prothese ähm die pflegeleicht ist. (Interviewerin: Mhm (bejahend)) Und zwar dieser Gurt und die Polster, das ist alles so wie’s sein sollte (Interviewerin: Mhm (bejahend)) ähm aber dieser Hauptring mit den, mit den Gurten, die sind alle, die Sachen sind alles (vernietet?), beziehungsweise ähm über Schnalle und dann vernietet (Interviewerin: Mhm (bejahend)) und die Bowdenzüge nicht lösen und solche Sachen. Ähm mir reißt regelmäßig dieser Anschlag vom Ellebogen, mir bricht regelmäßig die, ähm ich glaub die sieht man nicht, nee die ist äh, doch hier. Mir bricht regelmäßig die

Rasterung von der, vom Ellebogen (Interviewerin: Mhm (bejahend)), was mit mechanischer Belastung zu tun hat. Also das ist schon klar, würd' ich 'se nicht nutzen, würde das nicht passieren (Interviewerin: Mhm (bejahend)). Ähm und im Laufe der Zeit wird der Bowdenzug sehr viel schwergängiger. Das wäre sicherlich ein Vorteil, wenn es elektrisch wäre (Interviewerin: Mhm (bejahend)), gar keine Frage. Aber ähm das, wenn er seinem Lebensende entgegengeht, dann ist es so, dass man schon vermutet, dass irgendwann jetzt irgendetwas reißen wird (Interviewerin: Mhm (bejahend)), weil es einfach so schwergängig wird. (Interviewerin: Mhm (bejahend)) Ohne, dass ich jetzt irgendwie fehlerhaft öle oder sowas, da&das&da mach ich gar nichts, aber es ist trotzdem so, dass es immer schwerer wird. #00:25:44-0#

Interviewerin: Ja. #00:25:46-8#

Teilnehmer 15: Ja, also eine Prothese die äh atmungsaktiv wäre, wo die Gurte alle durch Druckknöpfe beispielsweise (Interviewerin: Mhm (bejahend)), das können ja auch sehr flache Druckknöpfe, die (...?) eingesetzt sind, ähm solche Sachen. Das wäre für mich ähm im Alltag sehr sehr f-&sehr sehr hilfreich. #00:26:01-7#

Interviewerin: Mhm (bejahend) und wenn Sie nochmal sagen: Bei der die Sie davor hatten, bei der Elektrischen, haben Sie ja zum Beispiel schon mal gesagt mit dem Akku, dass eigentlich das Gewicht ganz gut war und das hätte an 'ner andren Stelle ähm also sein müssen. Gab's da noch irgendwas, wo Sie eben sagen, womit hatte zum Beispiel dieses Aufwendige, dass es Ihnen zu aufwendig war, womit hat das zum Beispiel zu tun? #00:26:23-4#

Teilnehmer 15: Ja, er sagt grade, dass die&dass er beim äh Hände schütteln es nicht kontrollieren kann. (Interviewerin: Mhm (bejahend)) Und das ist natürlich auch etwas, wenn ich's nur öffne mit der Feder, die Federbelastung, (Interviewerin: Mhm (bejahend)) ist im Grunde genommen der Punkt um den es da beim Festhalten oder wie auch immer geht, (Interviewerin: Mhm (bejahend)) ähm ist das für mich glaube ich, zumindest war es in der Zeit so, ähm für mich nachvollziehbar damit umzugehen, als wenn ich 'n&'n E-Motor bediene (Interviewerin: Mhm (bejahend)). Einfach nur auf und zu. (Interviewerin: Mhm (bejahend)) Vielleicht reicht meine Erfahrung auch einfach nicht aus (Interviewerin: Mhm (bejahend)). Möglich. #00:27:01-6#

Interviewerin: Ok, ja. Aber das ist was, was Sie einfach so wahrgenommen haben? #00:27:04-0#

Teilnehmer 15: Ja, ja. #00:27:03-9#

Interviewerin: Mhm (bejahend). Und Herr (Name von Teilnehmer 25 aus Datenschutzgründen ausgelassen), Sie haben grade noch gesagt, eben so im Camp kam dann nochmal so der Gedanke ok, dass man äh dass man doch nochmal 'ne Prothese nimmt? #00:27:15-2#

Teilnehmer 25: Ja, besser gesagt ich wurde von ähm jemandem ähm sozusagen über-&in Anführungszeichen überredet. Er hat mir eben halt ähm erzählt, was weiß ich, wie toll Prothesen sind und so weiter (Interviewerin: Mhm (bejahend)) blabla so und dann eben halt auch, eben halt die Touch Bionics und die hat sich jetzt dann

bisschen besser angehört als die andren (Interviewerin: Mhm (bejahend)), so dass die doch eben halt viele Funktionen hat die eben halt die anderen ja nicht haben (Interviewerin: Mhm (bejahend)), ähm sei es allein schon, dass der Daumen drehbar ist und dass man halt schon zum Teil einzelne Finger da bewegen kann (Interviewerin: Mhm (bejahend)). Und der hat mich dann sozusagen überredet und ich hab‘ dann eben halt „ja“ gesagt. Anschauen kann ich’s mir ja, nein sagen kann ich ja dann immer noch. (Interviewerin: Ja.) Und dann bei der Vorführung eben halt, hab‘ ich gesehen ok, die kann ja doch bisschen was. (Interviewerin: Mhm (bejahend)) Und wirklich auch in die Zukunft gedacht ist es glaub ich wirklich am sinnvollsten. #00:28:07-1#

Interviewerin: Mhm (bejahend) und ähm als Sie die gesehen haben, was war da so von den Funktionen das Wichtigste, wo Sie gesagt haben das ist was, was mich komplett überzeugt? Kann man das sagen? Gab’s irgend ‘nen Punkt der so der wichtigste war, der coolste vielleicht, was die dann kann? #00:28:19-9#

Teilnehmer 25: Weiß ich nicht, also was ich glaub ich ähm wenn ich jetzt mal komplett jetzt absehe von allen Funktionen oder was weiß ich, dass die eben halt bisschen auch Gestenbewegung kann, sag ich jetzt mal. (Interviewerin: Mhm (bejahend)) So dass eben halt zum Beispiel mit Hände schütteln oder so, dass die dann erkennt ich will jetzt die Hand schütteln und dann sozusagen in den Modus geht, dass eben sie eben halt die Hand so machen soll ohne dass man jetzt irgend ‘ne App öffnet und das halt einstellt (Interviewerin: Ja.). #00:28:53-4#

Interviewerin: Ist das das, was Sie auch gesagt haben mit dem Stinkefinger zeigen, ist das das was dann vielleicht einfach fehlt, wenn man, wo Sie sagen Sie kommen eigentlich ohne Prothese voll gut zurecht, gibt’s sowas, einfach sowas, dass man halt mit der Hand was zeigen kann? #00:29:07-8#

Teilnehmer 25: Nein, das jetzt nicht unbedingt. So das war (kurze Pause) wenn, eigentlich heißt es ja auch nur „Fick dich“ sozusagen und hab so ’n Selbsta-&Selbstbewusstsein, dass ähm wenn ich’s wirklich bräuchte sag ich’s der Person auch ins Gesicht. #00:29:23-7#

Interviewerin: Ok (lacht). Ok das heißt, es war einfach mehr so ’n Accessoire was sie halt hat, aber das ist nichts, (Teilnehmer 25: Ja.) wo Sie sagen: „Manchmal fehlt mir das, dass ich mit (Teilnehmer 25: Nein, nein.) meiner Hand einfach mal irgendwas zeigen kann? #00:29:33-6#

Teilnehmer 25: Nein. #00:29:35-2#

Interviewerin: Ok, dann würd‘ ich gerne wissen: Herr (Name von Teilnehmer 15 aus Datenschutzgründen ausgelassen), Sie haben vorher schon gesagt, dass Sie die eigentlich die ganze Zeit tragen und trotzdem haben Sie dann gesagt, dass Sie so außerhalb von Beruf, dass Ihnen gar nicht so groß einfällt, ähm wo die da besondere Funktionen erfüllt. Gibt’s Situationen, in denen Sie die weglassen, die Prothese nicht tragen? #00:29:51-3#

Teilnehmer 15: Nee. #00:29:52-3#

Interviewerin: Gar nicht? #00:29:53-7#

Teilnehmer 15: Außer beim Schlafen und unter der Dusche. #00:29:53-8#

Interviewerin: Mhm (bejahend). #00:29:57-9#

Teilnehmer 15: Wie gesagt, ich hab's im Urlaub mal probiert, aber das äh da bespra-&da entsprach mein Inneres nicht meinem Äußeren. #00:30:03-9#

Interviewerin: Mhm (bejahend) #00:30:06-3#

Teilnehmer 15: Ähm, ich weiß nicht wie ich's sagen soll. (kurze Pause) Ja, mit Prothese fühl ich mich kompletter, (Interviewerin: Mhm (bejahend)) drücken wir's einfach ganz simpel aus. #00:30:16-9#

Interviewerin: Ja. Und gibt's irgend 'ne Erklärung für das Gefühl? Ist es eben das Gewicht, das wie man sich im Spiegel sieht oder woran liegt das? #00:30:24-9#

Teilnehmer 15: Bestimmt. #00:30:24-2#

Interviewerin: Mhm (bejahend) #00:30:25-2#

(kurze Pause) #00:30:27-6#

Teilnehmer 15: Bestimmt. (kurze Pause) Also man muss dazu sagen, dass ich mh dass ich eigentlich immer arbeite, insofern (Interviewerin: Mhm (bejahend)) fehlt auch nicht viel-. Weil ich bin einfach langsam (Interviewerin: Mhm (bejahend)). Insofern fehlt äh Freizeit sowieso. Das ist&das sind mal Parties oder so. (Interviewerin: Mhm (bejahend)) Irgendwelche Aktionen, so urlaubsmäßig oder so, das (haben?) wir nicht. Autofahren brauch ich keine Prothese, Gott sei Dank. Genau, find ich schlimm genug, aber (Interviewerin: Mhm (bejahend)) will die Versicherung halt eben sehen. (Interviewerin: Mhm (bejahend)) Nee. Aber ich bin, wie gesagt, ich rede wie der Blinde von der Farbe. Ich, ich müsste einfach mal sehen welche Möglichkeiten es überhaupt gibt, damit hab' ich mich nie befasst. #00:31:19-4#

Interviewerin: Nee, aber das ist ja auch vollkommen ok. Es geht ja jetzt einfach um die Situation, wie's jetzt grade ist. #00:31:22-9#

Teilnehmer 15: Ok. #00:31:21-1#

Interviewerin: Genau, mhm (bejahend). Und inwieweit spielt das Optische, das Kosmetische von der Prothese 'ne Rolle? #00:31:31-0#

Teilnehmer 15: Zunehmend weniger, aber das hat 30 Jahre lang 'ne ganz große Rolle gespielt. #00:31:32-4#

Interviewerin: Mhm (bejahend) #00:31:34-3#

Teilnehmer 15: Ja. #00:31:36-3#

Interviewerin: Und was dabei? Das die-. Also was ist da so das Wichtigste, was man von der Prothese haben will, oder verlangt? #00:31:40-4#

Teilnehmer 15: Wie die anderen zu sein. #00:31:42-9#

Interviewerin: Mhm (bejahend) #00:31:41-7#

Teilnehmer 15: Das ähm ich hab‘ nie ähm nie eine Situation erlebt, die das äh (kurze Pause) auf die hinaus ich ähm das eigentlich so der G-&das ist ähm aber vom ersten Tag an so gewesen. #00:32:02-4#

Interviewerin: Mhm (bejahend) #00:32:03-2#

Teilnehmer 15: Muss ich schon sagen. (Interviewerin: Mhm (bejahend)) (kurze Pause) #00:32:11-4#

Interviewerin: Und würden S-&also hat da die Prothese ähm dem was Sie von ihr wollten genügt, also vom, was das Kosmetische angeht oder gibt’s da auch irgendwas- #00:32:19-2#

Teilnehmer 15: Diese Elektrische nicht. Die war von den Proportionen her, da war der Unterarm viel zu lang (Interviewerin: Ja, ok.). Das hat mich eigentlich auch immer gestört. Mh:: aber ansonsten ja. (Interviewerin: Mhm (bejahend)) Ansonsten, wie gesagt, es gibt (lacht) ich bin in England mehrmals gewesen bei einer Dame für mehrere Tage jeweils und äh am letzten Tag kam sie von den, äh grade von den Paralympics aus London zurück und da ist ihr das zum ersten Mal aufgefallen. #00:32:51-6#

Interviewerin: Mhm (bejahend) #00:32:51-6#

Teilnehmer 15: Das war ein großes Hallo (lacht), aber ähm viele Leute sehen ’s nicht. #00:32:58-4#

Interviewerin: Und aber würden Sie sagen oder kriegen Sie da ‘ne Rückmeldung woran das liegt, dass das nicht auffällt? Ist das, wie es in den Körper integriert ist, ist es ähm weil die so authentisch aussehen, wird da irgendwas rückgemeldet? #00:33:08-1#

Teilnehmer 15: Wahrscheinlich auch ‘n bisschen, dass ich damit allmählich relativ selbstverständlich umgehe. #00:33:14-5#

Interviewerin: Mhm (bejahend), ja. #00:33:17-6#

Teilnehmer 15: Wobei jeder der da so ’n kleines bisschen Plan hat, der wird mitkriegen, wenn ich den Ellebogen blockiere oder solche Geschichten natürlich (Interviewerin: Mhm (bejahend)) aber ähm (kurze Pause) ja ich mein, ich&ich sehe, dass unterschiedliche Leute eben sehr unterschiedlich damit umgehen. (Interviewerin: Ja.) Manche machen ‘n Zirkus, ähm das ist genau das was ich eben nicht möchte. Ich möchte einfach nur das machen, was jeder andere in der Situation eben auch so machen möchte. Ich möchte der sein, der ähm dadurch nicht rausfällt (Interviewerin: Mhm (bejahend), ja.). Warum auch immer. #00:33:49-5#

Interviewerin: Ok. Und bei Ihnen, Herr (Name von Teilnehmer 25 aus

Datenschutzgründen ausgelassen), gibt’s Situationen, in denen ‘ne Prothese fehlt, in denen irgendwas nicht möglich ist? #00:33:57-8#

Teilnehmer 25: Ähm, nein. Also ich glaub bei Herrn (Name von Teilnehmer 15 aus Datenschutzgründen ausgelassen) ist es vor allem, da Sie jetzt sozusagen ähm dass man das Gefühl hat, dass sozusagen oder sozusagen man denkt da ist wahrsch-&glaub ich noch ‘ne Hand, sag ich jetzt mal, vorhanden und vor allem auch für ’s Selbstbewusstsein, wenn man jetzt durch die Straße geht, oder so. Ähm ich hab‘ mich dran gewöhnt, wenn ich durch ‘ne Straße gehe oder was weiß ich, ähm sagen wir mal da sind zehn Leute, neun drehen sich um. (Interviewerin: Mhm (bejahend)) Hundertprozentig. Könnten wir in der Stadt ‘n Test machen. Ist wirklich so. (Interviewerin: Mhm (bejahend)) Es drehen sich ständig viele um, also, oder schauen dann mal. Ähm das ist einfach so, ich hab‘ mich dran gewöhnt. Also wenn ich mal mit Freunden unterwegs bin oder so, denen fällt ’s dann auch auf so und ähm die wundern sich dann auch so, aber ändern kann man’s nicht. Es ist ja auch, sag ich mal, neugierig. Wo ich Herrn (Name von Teilnehmer 15 aus Datenschutzgründen ausgelassen) hab‘ ich auch einmal kurz mir die Hand angeschaut, oder so, von Ihnen. Man, also ich finde man sieht’s, dass es auf jeden Fall ‘ne Fakehand ist. Allein wegen der Farbe und so weiter. (Interviewerin: Mhm (bejahend)) Und auch wie sie aussieht und von der Handhabung her auch schon. Ähm also meine, wenn ich dann meine hab, dann auch, die wird auch mit dem durchsichtigen Überzug dann auch sein (Interviewerin: Mhm (bejahend)), dass man dann auch direkt dann sieht. Ich finde ähm wenn, sollte man sich da auch nicht verstecken so. Abe das ist natürlich auch immer zu Person zu Person anders. #00:35:25-7#

Interviewerin: Klar. #00:35:27-5#

Teilnehmer 25: Also ähm ich betreue auch ähm nicht nur ähm das im Behindertencamp, sondern auch andere Sachen und so weiter, deswegen kenn ich auch ganz viele Charakter von kleinen Kindern, Kindern und was weiß ich so und ich glaube vielen hilft es auch wirklich, sich da sozusagen hinter zu verstecken. (Interviewerin: Mhm (bejahend)) Und ähm zu sagen: „Ich bin ja doch ganz normal.“ und ähm die eben halt nicht so ’n selbststarkes Bewusstsein haben wie ich und sagen: „Jo, ich hab‘ ‘ne Behinderung aber ich kann trotzdem alles, seht mich an!“ so nach dem Motto. Und dann sagen: „Ok, ich bin genauso wie du. (Interviewerin: Mhm (bejahend)) Ich hab zwar ‘ne Behinderung ähm aber man sieht’s mir nicht an:“ #00:36:06-9#

Interviewerin: Mhm (bejahend), ja. Ok, das heißt das Optische macht schon mal nichts aus und da wird auch die Prothese so sein, dass es auffällig ist und wenn Sie aber sonst überlegen, gibt’s irgendwas wo die Prothese vielleicht ‘ne Erleichterung ist, bis auf das Autofahren jetzt? #00:36:22-6#

Teilnehmer 25: Ähm ich könnte mir vorstellen beim Schwimmen, im Wasser. Ähm, ähm (...?). Aber sonst jetzt auch, sonst fällt mir jetzt auch grade nichts ein. (kurze Pause) Doch, ähm Gitarre spielen oder so, aber das kann ja auch nicht jeder. (Interviewerin: Mhm (bejahend)) Also ich find, ich mit meinem Marimbafon, Klavier, Schlagzeug und sonst eigentlich alle perkussiven) Instrumente, kann schon relativ viel spielen (Interviewerin: Mhm (bejahend)), das reicht dann eigentlich auch. #00:36:56-1#

Interviewerin: Ja und wenn Sie dann aber sagen Sie kriegen jetzt die neue Prothese und eigentlich gibt's außer dem Autofahren nicht so richtig was, wo die eigentlich fehlt, wird es schwer die dann zu benutzen? #00:37:08-8#

Teilnehmer 25: (seufzt) Ich glaube ja. Ich v-&würde, also man muss es ausprobieren natürlich, also ähm es ist definitiv vor allem, wie ich schon vorhin gesagt hab 'ne enorme Kopfsache (Interviewerin: Mhm (bejahend)), dass man halt eben jetzt ähm eben halt mit der rechten Hand zum Beispiel das Glas dann, also könnt ich zwar auch schon so ohne Prothese (Interviewerin: Ja.), ähm aber es ist glaub ich 'ne alleine eine große Kopfsache, weil ich's ja halt nie anders gewohnt bin (Interviewerin: Mhm (bejahend)), ich kenn 's ja gar nicht anders. Im Gegensatz zu Herrn (Name von Teilnehmer 15 aus Datenschutzgründen ausgelassen), wenn er jetzt zum Beispiel äh direkt dann ähm, da hab ich&wie heutzutage der technische Stand ist, könnte er wahrscheinlich im Jahr irgendwie genau, also ich würd jetzt nicht sagen gleich wie seine rechte Hand, aber zumindestens zu 60% die linke Hand benutzen wie seine rechte Hand (Interviewerin: Mhm (bejahend)) (Teilnehmer 15: Mhm (bejahend)). #00:37:59-9#

Interviewerin: Und aber Sie für sich selber würden sagen das- #00:38:06-2#

Teilnehmer 25: Ich würd 's wahrscheinlich ausprobieren und definitiv ähm damit arbeiten, (um wie?) sag ich jetzt mal, dass ich zumindest einmal weiß, wie man im besten Fall damit umgehen könnte (Interviewerin: Mhm (bejahend)) ähm und dann eben halt sehen, ob sie eben halt für mich sinnvoll ist, oder nicht. (Interviewerin: Mhm (bejahend)) #00:38:25-3#

Interviewerin: Ja, das heißt erstmal ausprobieren. Aber so richtig fehlen tut sie eigentlich nicht? #00:38:30-0#

Teilnehmer 25: Nein. #00:38:31-8#

Interviewerin: Mhm (bejahend) #00:38:32-3#

Teilnehmer 25: Sonst hätt' ich ja auch längst schon, sonst säß' ich jetzt hier mit zwei Prothesen (Interviewerin: Mhm (bejahend)). #00:38:36-9#

Interviewerin: Und, wie war das aber zum Beispiel ganz am Anfang? Sie haben gesagt ganz früher hatten Sie auch so eine mit so 'nem Haken, oder? #00:38:41-4#

Teilnehmer 25: Ja das ähm lag glaub ich eher ähm, ich muss dazu sagen meine Mutter ist Ärztin ähm kennt sich eben halt deswegen auch bisschen mit Mediz-&auch in diesem medizinischen Bereich (Interviewerin: Mhm (bejahend)) ähm aus ähm und sie hatte oder hat immer noch so die Meinung: Braucht man ja nicht unbedingt. Oder was weiß ich so, also hat mich sozusagen so aufwachsen lassen wie ich bin und auch jetzt nicht die ganze Zeit sozusagen bemuttert und mir das ähm gefüttert oder was weiß ich, sondern ich konnte mir alles selbst beibringen. Hab' ich auch gemacht. (Interviewerin: Ja.) Ähm und dann wird natürlich eben halt von anderen Leuten gesa-&ähm gesagt, weil die natürlich immer alles besser wissen. Zum Beispiel meinte meine Grundschullehrerin damals, sie wollte mir meine Jacken und Ärmel und so weiter umnähen, dass das eben halt nicht passiert. Warum? Kann den Kram

auch hochkrempeln (krempelt seine Ärmel hoch), dann geht’s ja auch. Ähm und dann glaub ich sozusagen, wurde, hat sie dann irgendwann sozusagen gleich eingeknickt und gesagt: „Warum denn nicht?“ so nach dem Motto „einmal ausprobiert“. (Interviewerin: Mhm (bejahend)) Auch. #00:39:46-8#

Interviewerin: Ok. Ähm wir sind jetzt schon ungefähr bei der Hälfte der Zeit angekommen. Ähm ja wir hätten auch noch so Zeit, dass wir einfach ‘ne&‘ne kurze Pause machen können, wenn Sie eine brauchen? #00:40:00-0#

Teilnehmer 15: Ich bräuchte keine. #00:40:01-4#

Teilnehmer 25: Ich auch nicht. #00:40:02-8#

Interviewerin: Ok, weil sonst können wir auch einfach weitermachen. #00:40:02-7#

Teilnehmer 15: Also von mir aus gern. #00:40:05-3#

Interviewerin: Dann ist es früher fertig, dann geht’s früher zurück zum Austauschschüler. Ok. Ähm dann wüsste ich gerne als nächstes von Ihnen, Herr (Name von Teilnehmer 15 aus Datenschutzgründen ausgelassen), Sie haben gesagt Sie benutzen die immer, was passiert denn wenn die kaputt ist, eben wenn die irgendwo-. Ja, wenn die irgendwo- #00:40:24-7#

Teilnehmer 15: Ganz schlecht. Ich hab‘ zwei, ähm die andere ist genauso mechanisch, aber dann hab ich mh ähm. Es ist schon passiert, da war die bei (Name eines Sanitätshauses aus Datenschutzgründen ausgelassen) und ähm die andere war kaputt (Interviewerin: Mhm (bejahend)), da ist das Ellebogengelenk gerissen, da muss ich den Tag anders strukturieren. Dann weiß ich, dass ich ganz bestimmte Sachen einfach nicht machen kann. Oder ich muss sie halt eben reparieren (Interviewerin: Mhm (bejahend)). Und ähm, also ohne Prothese ist bei mir ganz schlecht. Ganz. (Interviewerin: Mhm (bejahend)) Komm ich nicht mit klar. #00:40:53-2#

Interviewerin: Ja, aber das heißt es gibt ’n Ersatz und der wird dann auch benutzt. #00:40:56-6#

Teilnehmer 15: Ja. #00:40:59-8#

Interviewerin: Mhm (bejahend) #00:41:00-7#

Teilnehmer 15: Aber auch die ist nicht so mh von der Pflegefreundlichkeit her, wie ich mir das wünschen würde. (Interviewerin: Mhm (bejahend)) Also beispielsweise, wenn ich hier, das ist jetzt bei der so, nee bei der ist das ni-&die andere hat hier ‘ne Lederschnalle (Interviewerin: Ja.). Ich kann kein Kind auf ’n Arm nehmen, ohne dass es sich die Nase äh an d-&an dem Dorn (Interviewerin: Mhm (bejahend)) blutig macht. Es ist&das sind so Dinger, im 20. Jahrhundert geht das gar nicht. (Interviewerin: Mhm (bejahend)) Im 21. #00:41:24-8#

Interviewerin: Ja. Und ähm die Ersatzprothese ist das einfach die, die sie davor hatten? #00:41:28-8#

Teilnehmer 15: Genau. Das war die aus äh (Name eines Ortes aus Datenschutzgründen ausgelassen). #00:41:31-4#

Interviewerin: Ja, ok. Und gibt’s noch irgendwelche anderen Hilfsmittel die Sie benutzen, wo Sie einfach- #00:41:36-0#

Teilnehmer 15: Ja, ich hab‘ verschiedene Hooks gehabt. #00:41:38-6#

Interviewerin: Mhm (bejahend) #00:41:40-2#

Teilnehmer 15: Ähm den Runden, dann den mit dem (kurze Pause), hab ich am Anfang auch ziemlich viel benutzt (Interviewerin: Mhm (bejahend)), ähm würde ich wahrscheinlich auch wenn ich die andere Mechanische tragen würde benutzen können, (kurze Pause) aber besteht keine Notwendigkeit mehr, nee. #00:41:59-4#

Interviewerin: Mhm (bejahend), das heißt die benutzen Sie gar nicht? #00:42:03-6#

Teilnehmer 15: Eigentlich nicht, nee. #00:42:05-7#

Interviewerin: Ok, ja. #00:42:05-2#

Teilnehmer 15: Also ich werd‘ sie jetzt benutzen müssen, weil der Ring oben kaputt gegangen ist, (Interviewerin: Mhm (bejahend)) ähm ich kann die im Moment nicht einsetzen richtig, ähm da werd‘ ich dann die solange tragen, solange die dann hier zur Reparatur ist (Interviewerin: Mhm (bejahend)). #00:42:16-8#

Interviewerin: Und wenn die kaputtgeht, was ist dann so das, wo die am meisten fehlt? #00:42:22-3#

Teilnehmer 15: Das sind Arbeitsprozesse. (Interviewerin: Ja.) Handwerkliche Arbeitsprozesse. #00:42:28-4#

Interviewerin: Mhm (bejahend), die dann einfach nicht mehr möglich sind? #00:42:28-9#

Teilnehmer 15: Genau, ja. #00:42:30-5#

Interviewerin: Mhm (bejahend), ja. #00:42:32-5#

Teilnehmer 15: Und es ist halt eben auch blöd, wenn diese Sch-&ähm Schanierwand reißt, dass halt der Ellebogen zur andern Seite umklappt. (Interviewerin: Mhm (bejahend)) Das sorgt zwar für Erheiterung, aber- #00:42:41-9#

Interviewerin: Aber nicht für Sie. #00:42:41-5#

Teilnehmer 15: (lacht) Nee, nicht für mich. Zumal das auch dann begrenzt wird durch diesen Kunststoff und das bricht aus. (Interviewerin: Mhm (bejahend)) #00:42:51-1#

Interviewerin: Und wie ist es dann mit der Reparatur? Also wenn man die abgeben muss, ähm weil da könnt ich mir vorstellen, dass zum Beispiel bei den Elektrischen ist das ja oft einfach ‘n langer Prozess. Wie lange dauert das? Oder wie lang muss

man dann auskommen und sich behelfen ohne? #00:43:04-9#

Teilnehmer 15: Viel zu lange. #00:43:04-5#

Interviewerin: Mhm (bejahend) #00:43:05-9#

Teilnehmer 15: Für mich viel zu lange. Ich bin selbstständig, bei mir zählt die Stunde und wenn ich von, ich komme aus (Name der Stadt aus Datenschutzgründen ausgelassen), wenn ich dann hier bin, dann ist der Vormittag komplett gestrichen (Interviewerin: Mhm (bejahend)). Das muss ich dann-. Also sie darf eigentlich nicht kaputt gehen. (Interviewerin: Mhm (bejahend)) Und ich bin froh, dass sie so robust ist, wie sie ist. (Interviewerin: Ja.) 'N paar Schwachpunkte, aber grundsätzlich ist es ok. Nur, wenn es etwas gäbe was das Ganze vielfältiger gestalten würde elektrisch, natürlich. Würd' ich schon gerne probieren. #00:43:39-4#

Interviewerin: Aber das heißt, das ist dann auch für 'ne, zum Beispiel 'ne Elektrische, wär das 'n wichtiges Kriterium, dass die halt eben robust sein muss und funktionieren muss? #00:43:46-4#

Teilnehmer 15: Absolut, ja. Ja, sonst kann ich's nicht gebrauchen. Also Kosmetik brauch ich (…?) ähm robuste, keine elektrische. #00:43:56-4#

Interviewerin: Ist das der wichtigste Punkt, den 'ne Prothese erfüllen muss? #00:44:00-0#

Teilnehmer 15: (kurze Pause) Mh also nein, der Wichtigste nicht. Der Wichtigste ist, sie muss einsetzbar sein. Ähm Kosmetik ist sekundär, aber (Interviewerin: Mhm (bejahend)) also allmählich. Früher war das sicherlich äh anders (Interviewerin: Mhm (bejahend), aber das ist für mich heute nicht mehr so wichtig. #00:44:16-6#

Interviewerin: Ja und einsetzbar, was genau heißt das? #00:44:21-5#

Teilnehmer 15: Ja, ich muss festhalten können, ich muss gegenhalten können, ich muss ähm Sachen greifen können (Interviewerin: Mhm (bejahend)) ähm::: (kurze Pause). Ja, es wäre vielleicht wirklich sinnvoll, wenn äh wenn ich mehr Funktionen hätte. Ähm ich, normalerweise öffne ich mir mit dem Ellebogen die Türklinke, wenn ich das, weil ich's halt eben erstmal blockiere und dann - das kann ich mir sparen. (Interviewerin: Ja.) Ähm wenn es solche Funktionen hätte wäre das natürlich nicht schlecht. #00:44:55-7#

Interviewerin: Mhm (bejahend), mhm (bejahend) ok. Und bei Ihnen Herr (Name von Teilnehmer 25 aus Datenschutzgründen ausgelassen) gibt's irgendwas an Hilfsmitteln, die Sie benutzen? #00:45:01-5#

Teilnehmer 25: Ja, beim Schlagzeug spielen, Marimbafon spielen ähm da hab ich so Manschetten, ähm die auch von (Name eines Sanitätshauses aus Datenschutzgründen ausgelassen) hergestellt worden sind, die zieh ich mir einfach immer drüber und mach die dann mit Klettverschlüssen eben halt zu (Interviewerin: Mhm (bejahend)) ähm und da sind dann eben halt die entsprechenden (..?) dann drin und dann spiel ich dann eben halt so dann Schlagzeug oder Marimbafon und ähm sonst eigentlich nicht. #00:45:24-7#

Interviewerin: Und wie ist es zum Beispiel beim Schreiben? #00:45:27-0#

Teilnehmer 25: Ja da nimmt man meistens ‘n Blatt Papier und ‘n Stift. #00:45:30-9#

Interviewerin: Und das geht einfach so? #00:45:31-3#

Teilnehmer 25: Ja. #00:45:35-2#

Interviewerin: Krass. Und ähm was Sie vorher noch gesagt haben mit dem, dass es cool wär‘ wenn ‘ne Prothese so ’n Finger hätte, der halt ähm auf ’m Handy, auf ’m Touchding funktioniert, funktioniert das auch einfach so? #00:45:45-2#

Teilnehmer 25: Ja. #00:45:45-1#

Interviewerin: Ok. Das heißt Schlagzeug ist das Einzige wo irgendwas dazu genutzt wird? #00:45:50-0#

Teilnehmer 25: Ja. #00:45:52-0#

Interviewerin: Cool (lacht). Ähm dann würd‘ ich gerne als nächstes ‘ne bisschen abstraktere Frage stellen und zwar wenn Sie sich vorstellen, also jetzt mal ohne was die Technik machen kann, oder was Sie selber machen können, aber mal vorstellen wie die perfekte Prothese aussehen muss, was die können muss, was man an denen die es bis jetzt gibt verändern muss, wie wäre so eine? Also was könnte die, wenn man überhaupt nicht drauf achtet was gerade angeboten wird, was die können oder was eben nicht oder auf welche Art und Weise, was wäre die perfekte Prothese die man sich wünscht, die man auf jeden Fall haben wollen würde? #00:46:26-6#

Teilnehmer 25: Mhh. #00:46:33-3#

Interviewerin: (lacht) #00:46:33-3#

Teilnehmer 15: Also ähm ideal wäre, wenn sie über ’n Kopf gesteuert würde. (Interviewerin: Mhm (bejahend)) Natürlich. Vielleicht würden drei, vier, fünf Funktionen reichen, mir. Vielleicht auch, weil ich mir nicht mehr vorstellen kann, aber ähm etwas zu unternehmen, damit ich dann damit etwas machen kann (Interviewerin: Ja.) ist einfach ein Umweg. (Interviewerin: Ja.) Und wenn das so ideal wie bei einer, da denk ich ja nicht drüber nach, das mach ich dann (Interviewerin: Mhm (bejahend)) äh mit der rechten Seite, und wenn das mit der linken, das wär natürlich super, klar. (Interviewerin: Mhm (bejahend)) #00:47:10-0#

Interviewerin: Das heißt so ’ne Art von Intuition, dass es einfach sofort geht? #00:47:10-6#

Teilnehmer 15: Ja. #00:47:13-5#

Interviewerin: Und was wären die Funktionen, die sie können müsste? #00:47:17-9#

Teilnehmer 15: (seufzt) Äh:::m na von mir aus ähm einen beweglichen Ellenbogen (Interviewerin: Mhm (bejahend)), bewegliche Hand, (kurze Pause) drehbare Hand,

aber ich glaube bei mir wäre es nicht so wichtig, dass es einzelne Finger wären (Interviewerin: Ok, ja.), aber sie sollte schon fest zugreifen können. #00:47:47-1#

Interviewerin: Und die drehbare Hand, was fällt Ihnen da vor allem ein, in welchen Situationen sowas notwendig ist? #00:47:56-5#

Teilnehmer 15: Das ist alles Arbeit, alles harte Arbeit. Handwerkliche Tätigkeiten. #00:47:57-5#

Interviewerin: Mhm (bejahend) #00:47:59-9#

Teilnehmer 15: Wo ich etwas positionieren muss. (Interviewerin: Mhm (bejahend)) Ja, wenn ich etwas schraube, wenn ich irgendwas festhalten muss zum Bohren (Interviewerin: Mhm (bejahend)) solche Sachen. #00:48:10-5#

Interviewerin: Ok und was ähm zum Beispiel das Optische angeht? #00:48:16-1#

Teilnehmer 15: In dem Moment wäre es mir Wurst, weil ich dann doch wahrscheinlich ähm zur Feier-&zum Feierabend, zum Wochenende, zum Urlaub ‘ne andere Hand dranmachen würde. #00:48:28-3#

Interviewerin: Mhm (bejahend) #00:48:29-1#

Teilnehmer 15: Ähm, also, wenn ich daran denke, dann denke ich an eine Arbeitsprothese. #00:48:34-9#

Interviewerin: Mhm (bejahend), aber woran liegt das dann, dass Sie sagen würden, danach würden Sie ‘ne andere anziehen? #00:48:39-4#

Teilnehmer 15: Aus kosmetischen Gründen. Weil ich mir vorstelle, dass etwas was ähm, ja vielleicht mit dem Gedanken an meine alte Elektrische, (Interviewerin: Ja.) äh das war dieser&dieser Dreh, riesen (bollo?) da, ähm das ist ja nun, also das, da könnte ich in der Freizeit nichts mit anfangen. #00:48:58-2#

Interviewerin: Mhm (bejahend), ok aber wenn wir mal dabei bleiben, dass eben alles möglich wär, dann wenn die auch gut aussehen würde, würden Sie die auch immer tragen? #00:49:07-5#

Teilnehmer 15: Ja, natürlich. #00:49:09-9#

Interviewerin: Und wie müssen die dann aussehen? #00:49:10-5#

Teilnehmer 15: Schlicht. #00:49:10-8#

Interviewerin: Mhm (bejahend) #00:49:12-0#

Teilnehmer 15: Schlicht. Ähm wie gesagt, da sind mit ’m Gewicht hab ich nicht grundsätzlich das Problem ähm solang ’s richtig verteilt ist ähm na ich kann mir ja gar nicht vorstellen, wenn ich sage, dass ich die robust-, dass ich ‘ne robuste Prothese brauche, da weiß ich jetzt nicht, wie weit Elektronik robust ist (Interviewerin: Mhm (bejahend)), da kann ich gar nichts zu sagen. #00:49:34-7#

Interviewerin: Ok, aber robust-. Also was fällt Ihnen ein, wenn Sie sagen robust. In welcher Situation muss das robust sein und auf welche Art und Weise muss das robust sein? #00:49:46-3#

Teilnehmer 15: Also ähm wenn ich ‘n Instrument stimme (Interviewerin: Ja.), ähm dann habe ich das vor der Amputation mit der linken Hand, hab ich das mit der Handkante gemacht (haut mit der Handkante auf den Tisch). (Interviewerin: Ja.) Ähm wenn ‘ne Prothese das nicht mitmacht, kann ich damit nicht arbeiten. #00:50:03-3#

Interviewerin: Mhm (bejahend), ok, das h- #00:50:06-2#

Teilnehmer 15: Und das macht sie mit. #00:50:05-4#

Interviewerin: Das heißt sie muss halt einfach was abkönnen, dass sie nicht gleich zerfällt, wenn man sie irgendwo dagegendonnert, aber gibt’s noch irgendwas wo Sie sagen irgend ‘ne Situation, eben wo sie Robustheit zeigen muss, was sie abkönnen- #00:50:20-0#

Teilnehmer 15: Wenn ich Holz teile, oder&oder sowas festhalte, mit ’m Stecheisen zu arbeiten, wenn ich etwas säge (Interviewerin: Mhm (bejahend)), wenn ich ähm, ähm Dinge zuschneide (Interviewerin: Mhm (bejahend)) ähm&ähm mit ’m großen Lineal, dann muss ich richtig fest drücken, festhalten können (Interviewerin: Ja.). Und ähm das sind so die Dinge, wo das Ellenbogengelenk bricht, beispielsweise. #00:50:40-0#

Interviewerin: Ja. Und wenn Sie dann nochmal so ’n bisschen erzählen, Sie sagen grade bei der Arbeit, hat die halt so den höchsten Stellenwert. Ich kann mir nicht so genau vorstellen, ähm wie so ‘ne Arbeit dann aussieht, was sind das genau- #00:50:51-2#

Teilnehmer 15: Basteln. Das ist eigentlich nur basteln. #00:50:52-2#

Interviewerin: Mhm (bejahend), aber wenn Sie’s nochmal ‘n bisschen genauer einfach erzählen. Was sind das für Gegenstände die gegriffen werden müssen, welche, welche Funktionen (Teilnehmer 15: Mhm (bejahend)) eben da wo sie vielleicht auch sagen, das hab‘ ich früher so gemacht und jetzt behelfe ich mir halt mit der Prothese, das was die Prothese halt kann. Aber was müsste die, oder wo wär’s vielleicht ‘ne Erleichterung, dass die noch irgendwas abnehmen könnte, wenn Sie vielleicht so drüber nachdenken, wie’s früher mit der Linken war? #00:51:14-5#

Teilnehmer 15: Also, wenn das Ellebogengelenk beweglich wäre, ähm dann könnt ich sehr sehr viel mehr Teile tragen beispielsweise, die ich jetzt wirklich nur mit einer Hand tragen muss, obwohl sie sehr, sehr schwer sind, einfach um das Gleichgewicht zu halten. #00:51:25-4#

Interviewerin: Mhm (bejahend), was ist das zum Beispiel? #00:51:24-3#

Teilnehmer 15: Ja zum Beispiel (‘ne Mechanik?) vom Flügel oder vom Klavier. #00:51:27-4#

Interviewerin: Ja, mhm (bejahend) #00:51:29-3#

Teilnehmer 15: Ähm, das ist ‘ne Sache, die geht auch ziemlich auf ’n Rücken, das merk ich auch. #00:51:32-5#

Interviewerin: Ja. #00:51:30-7#

Teilnehmer 15: Und hätte ich jetzt einen Gelenk(…?) mit der linken Hand unterfassen könnte, (Interviewerin: Ja.) das wäre schon nicht schlecht. #00:51:41-2#

Interviewerin: Mhm (bejahend) #00:51:42-4#

Teilnehmer 15: Wir fahren ziemlich viel über ’s Jahr in Frankreich rum und kaufen da Flügel und Klaviere (Interviewerin: Mhm (bejahend)). Ähm dazu gehört ebenfalls auch der Transport. Ähm wir tragen mit ’m Gurt, aber das Gleichgewicht halt ich mit, natürlich auch mit ’n Händen und mit ’n Armen. Und auch da wäre äh die Prothese, die wirklich richtig gut hält (Interviewerin: Mhm (bejahend)), die ich so nicht einsetzen kann, weil ich sie nicht kontrollieren kann (Interviewerin: Ja.). Da muss ich (...?) arretieren. (Interviewerin: Ja.) Ähm das wäre schon nicht schlecht, ja, ganz klar. #00:52:13-6#

Interviewerin: Und von dem Handmodul, also nur von der ähm Handinnenfläche, was für Dinge- #00:52:16-3#

Teilnehmer 15: Festhalten, wirklich festhalten. #00:52:20-3#

Interviewerin: Ja und was sind das für Sachen? Sind das große Sachen, kleine Sachen, was muss die da vereinen? #00:52:23-5#

Teilnehmer 15: Ähm alles. Alles. Ähm jetzt muss ich eben alles über die so genannte Dritte, das ist bei mir die zweite Hand, einspannen. (Interviewerin: Mhm (bejahend)) Das sind dann sowas wie Maschinenschraubstöcke oder sowas. (Interviewerin: Mhm (bejahend)) Ähm ist nicht toll, weil das hinterlässt Spuren, früher hab‘ ich sowas fest-&Hammerköpfe, wenn ich den Hammerkopf zum Beispiel aufbohre und leime (Interviewerin: Mhm (bejahend)) ähm oder wenn ich äh Mechanikteile ausachsen muss. Die muss ich jetzt immer einspannen, die könnte ich dann festhalten, so wie ich sie früher festgehalten habe. (Interviewerin: Mhm (bejahend)) Die sind von dieser Größe, sind auch nicht sonderlich schwer, die Sachen sind aber, die ich da mache, sehr (deficile?) und deswegen kann ich’s mir nicht leisten äh plus minus ein Millimeter zu arbeiten. (Interviewerin: Ja, ja.) Also das sind Dinge die so im Bereich von 25 tausendstel stattfinden. Da muss ich schon sehr genau arbeiten, wenn ich da nicht richtig festhalten kann, dann ist schlecht. #00:53:13-3#

Interviewerin: Ok und das heißt, das ist dann einfach ‘n Griff mit der, mit der ganzen Hand. #00:53:19-2#

Teilnehmer 15: Ja. #00:53:20-4#

Interviewerin: Und gibt’s aber auch Sachen wo was feineres, mh weiß nicht, wo man noch irgendwas mit der, mit der Hand können muss oder irgendwas greifen muss an

Gegenständen, an Werkzeug? #00:53:29-9#

Teilnehmer 15: Ja, das würde sich da sicherlich ergeben. Aber ähm da fehlt mir jetzt im Moment die Phantasie, welche Möglichkeiten es gäbe, in welchen Situationen wie äh damit zu arbeiten wäre. (kurze Pause) #00:53:51-7#

Interviewerin: Oder gibt’s irgendwas was Ihnen zum Beispiel einfällt wo Sie einfach ähm sagen jetzt, weil die halt nur diese Greif- und vielleicht Gegenhaltefunktion hat, wo Sie eben noch&noch bei irgendwas sagen da wär’s eigentlich schön, wenn die Rechte mal entlastet wär, wenn ich parallel mit der Linken was machen könnte, was jetzt halt durch die Prothesenarten nicht geht? #00:54:14-7#

(kurze Pause) #00:54:19-0#

Interviewerin: Also muss auch nicht. #00:54:21-3#

Teilnehmer 15: Ich bin mir nicht sicher. #00:54:23-2#

Interviewerin: Mhm (bejahend) #00:54:23-2#

Teilnehmer 15: Ich bin nicht sicher, dass es so ‘ne Situation gäbe. Ähm (kurze Pause) bleiben wir dabei, dass es, dass wenn der Ellbogen funktioniert und mir beim Tragen, beim ausgewogenen Tragen helfen würde, das wäre ‘ne sehr große Erleichterung und ich eine Hand hätte, die ich äh im Idealfall gedanklich kontrollieren könnte, aber in der Lage wäre wirklich festzuhalten. #00:54:48-2#

Interviewerin: Mhm (bejahend), ok. Das heißt dieser ganz feste Griff eben bei irgendwas handwerklichen (Teilnehmer 15: Genau.) das ist schon was, was ‘n großer Punkt ist? #00:54:56-0#

Teilnehmer 15: Ja, also für mich wäre das vorteilhaft. #00:54:58-1#

Interviewerin: Mhm (bejahend) und wenn Sie noch an den Alltag außerhalb des Berufs denken, gibt’s noch irgendwas an Funktionen ähm die vielleicht so ‘ne Wunschprothese noch haben sollte? #00:55:08-2#

Teilnehmer 15: Eigentlich nicht. #00:55:14-5#

Interviewerin: Mhm (bejahend) #00:55:15-7#

Teilnehmer 15: Wenn ich im Beruf klarkäme und das die beruflichen Möglichkeiten verbessern oder leichter machen würde, das wäre schon, das wäre schon klasse. #00:55:23-4#

Interviewerin: Ja, ok. Und bei Ihnen, Herr (Name von Teilnehmer 25 aus Datenschutzgründen ausgelassen)? #00:55:26-9#

Teilnehmer 25: Ähm ja, also leicht oder zumindestens sehr gut austariert, weil da manchmal ja das Gewicht sehr viel vorne (Interviewerin: Mhm (bejahend)). Dann vor allem drehbar und am besten auch ‘ne Art Handgelenk, also dass die auch nach oben und nach unten geht. #00:55:44-4#

Interviewerin: Und drehbar? Wo s-&einfach das die Handflächen drehbar sind, das Handgelenk? #00:55:52-9#

Teilnehmer 25: Ja, also sozusagen, dass die von mir aus bei sowas kann die auch 360 Grad gehen, das ist ja dann eigentlich auch egal. #00:55:57-1#

Interviewerin: Und ganz kurz bei&bei wo oder bei welcher Funktion ist dieses Drehbare besonders wichtig? #00:56:02-6#

Teilnehmer 25: Ähm, dass man, sag ich jetzt mal irgendwie Sachen, also, ‘n Handgelenk würde man ja auch drehen sozusagen, wenn man jetzt irgendwas nimmt, so. (Interviewerin: Ja.) Das jetzt sozusagen. Weil wenn sie jetzt starr ist, muss man ja ‘n ganzen Arm aus- und verdrehen (Interviewerin: Mhm (bejahend)), ja. Dass es dann eben halt hingeht. Ähm dann eben halt, dass man irgendwie knicken kann oder so. Sei es man ähm arbeitet irgendwie an der Tastatur oder was weiß ich, ähm- #00:56:34-6#

Interviewerin: Und auch da: Knicken? Das&wo muss das hin? #00:56:35-2#

Teilnehmer 25: Ja, dass die eben halt so nach oben ist, weil sonst muss man ja sozusagen ja anders ja dann arbeiten ähm (Interviewerin: Ja.), dass ähm sie irgendwie einfach zu steuern ist ähm. Aber dafür direkt, ohne Operation oder irgendwas so (Interviewerin: Mhm (bejahend)) und eventuell auch mit ‘ner anderen Technik als jetzt, also mit der Muskelabnahme oder so, aber dann dass man irgendwie ohne großartig das üben, dass man die getrennt ansteuern muss, die Muskeln. Weil das ist ja für Leute, ähm die, also für mich oder für Leute die eben halt das seit Geburt an haben, ist es ja ziemlich mühselig, dass&die Muskeln erstmal aufzutrainieren, weil man die ja nicht benutzt vom Handgelenk und so weiter und dann die getrennt zu ähm, anzusteuern. Das ist immer enorm schwierig (Interviewerin: Mhm (bejahend)). Also wenn man das macht, dann, ähm also ist man auch schon fertig, weil es was komplett anderes ist. (Interviewerin: Mhm (bejahend)) #00:57:38-9#

Interviewerin: Und ist das fertig, weil das anstrengend ist oder fertig, weil es einfach schwierig ist sich vorzustellen was man da eigentlich machen soll? #00:57:45-7#

Teilnehmer 25: Ja, es ist irgendwie, also es ist jetzt nicht so, weil man irgendwie zu anstrengend ist, sondern es ist so schwierig zu sagen ok, der Muskelstrang ist das und der Muskelstrang ist das. (Interviewerin: Mhm (bejahend)) Sondern man weiß jetzt, also Sie wissen: Ok, der Muskelstrang sind was weiß ich, der Finger und das dann der Finger oder was weiß ich, so. Aber man kann es sich eben halt nicht richtig vorstellen, so richtig. #00:58:07-6#

Interviewerin: Ja, ok, mhm (bejahend). #00:58:12-1#

Teilnehmer 25: Ähm ja das, das ist und, dass die ich glaub auch so fünf oder so Griffmuster würden schon reichen (Interviewerin: Mhm (bejahend)). So, was weiß ich Handy halten, Messer halten, irgendwie was weiß ich, das würd‘ glaub ich schon reichen. #00:58:28-9#

Interviewerin: Mhm (bejahend), was wär‘ dann noch wichtig? Handy halten, Messer halten, was wär‘ noch irgendwas, wo so ’n Griff- #00:58:34-8#

Teilnehmer 25: ‘Ne Greiffunktion für ‘ne Wasserflasche oder was weiß ich (Interviewerin: Mhm (bejahend)) ähm (kurze Pause) vielleicht auch Funktionen, die man selbst einstellen kann? (Interviewerin: Mhm (bejahend)) (kurze Pause) #00:58:48-6#

Interviewerin: Das heißt, dass sich der Griff nochmal ändert bei bestimmten Situationen, dass man das selber noch dazu machen kann? #00:58:52-9#

Teilnehmer 25: Nee, dass man sozusagen selber einen sozusagen kreieren kann oder was weiß ich. Also vielleicht auch so ‘ne Art Pinzettengriff oder so wär auch sinnvoll glaub ich. #00:59:06-2#

Interviewerin: Pinzettengriff, was genau heißt das, wie müsste das? #00:59:12-6#

Teilnehmer 25: Ähm das heißt, meine ich, dass die Hand eben halt spitz nach vorne läuft oder was weiß ich. Das nennt man mein ich auch Pinzettengriff. #00:59:24-2#

Interviewerin: (Zeigt den Pinzettengriff mit den Fingern) Sowas? #00:59:21-3#

Teilnehmer 25: Ja, ganz genau. #00:59:23-5#

Interviewerin: Ja, mhm (bejahend) #00:59:25-2#

Teilnehmer 25: Ähm irgendwie kleine Sachen vom Boden aufzuheben oder was weiß ich, Kleingeld zum Beispiel oder so. #00:59:31-1#

Interviewerin: Mhm (bejahend) #00:59:32-0#

Teilnehmer 25: Ja das ist auch ‘n Thema, dass man auch mit der kleine Sachen aufheben kann eben halt auch, dass sie vor allem auch ähm so ’n Druckpunkt hat, dass eben halt die, sie weiß wie viel Kraft sie anwenden darf oder sollte (Interviewerin: Mhm (bejahend)), dass sie vor allem robust ist, dass man mit der auch arbeiten kann, Sachen heben kann. #00:59:54-4#

Interviewerin: Und da robust, was muss die zum Beispiel aushalten, was ist da besonders wichtig, wo die mitmachen muss? #00:59:57-8#

Teilnehmer 25: Also ich find die sollte auch wenn die mal runterfällt oder was weiß ich (Interviewerin: Ja.), das definitiv aushalten. Schwere Sachen irgendwie schleppen oder so (Interviewerin: Mhm (bejahend)), jetzt nicht nur 10 Kilo oder, also auch 50, 60 Kilo oder so (Interviewerin: Mhm (bejahend)), dass das möglich ist. #01:00:13-8#

Interviewerin: Und noch bei den Griffen, die man selber einstellen kann: Was würden Sie da dazupacken, was wär‘ da wichtig, dass man sich sowas selber einbauen oder einstellen kann? #01:00:21-0#

Teilnehmer 25: Weiß ich ehrlich gesagt nicht. #01:00:25-1#

Interviewerin: Ok, ja (lacht). #01:00:29-0#

Teilnehmer 25: Also ich glaub ich würde, wenn es wirklich ‘ne Prothese gäbe, die alles könnte, würd ich glaub ich mit der dann Gitarre spielen wollen (Interviewerin: Mhm (bejahend)). Dass man dann eben halt der Prothese sagt: „Ok, das ist ’n G-ähm-Akkord und das ist ’n D.“ oder was weiß ich (Interviewerin: Mhm (bejahend)) und dass man das halt relativ schnell wechseln kann und eben halt damit Gitarre spielen könnte (Interviewerin: Mhm (bejahend)). #01:00:50-4#

Interviewerin: Ok, gibt’s noch irgendwas, was ich&wichtig ist, irgend ‘ne Funktion oder-. Oder vielleicht auch was Optisches, was die haben sollte? #01:00:59-8#

Teilnehmer 25: Also mir ist das Optische eigentlich komplett egal. Ähm für Leute eben halt, die kein selbstbewusstes haben oder sich da eben halt hinter verstecken soll, wollen relativ ähm natürlich sag ich jetzt mal (Interviewerin: Mhm (bejahend)). Aber ich finde, es sollte jetzt auch nicht aussehen komplett wie die andere Hand gespiegelt so. (Interviewerin: Mhm (bejahend)) Weil es ist, ich find man muss immer noch erkennen können, dass es ‘ne Prothese ist (Interviewerin: Ja.) also, weil man, ich find man kann sich da nicht verstecken. Entweder man steht da komplett dazu oder gar nicht, aber da sozusagen in Selbstmitleid zu versinken, das ist find ich keine Option (Interviewerin: Mhm (bejahend)) so. Man muss dazu einfach stehen. #01:01:38-3#

Interviewerin: Ja. Ok. Ja? #01:01:43-8#

Teilnehmer 15: Mir ist noch eingefallen, dass der positive Unterschied für eine Elektrische darin besteht, dass es äh dass ich das über einen Impuls machen kann. (Interviewerin: Mhm (bejahend)) Während, wenn ich das über Rückenzug, oder Ellebogen mache, dann ist es ‘ne Kraftanstrengung, die mir für das Dosieren zum Beispiel von anderen Sachen (Interviewerin: Mhm (bejahend)) in dem Moment fehlt (Interviewerin: Ja.). Ähm also das, wäre für mich sicherlich ‘n großer Unterschied, wenn ich das ganze über Impuls, statt über Muskelkraft, weil die ja beibehalten wird, solange ich den, den&den&den, was weiß ich, äh die Hand aufhabe. (Interviewerin: Mhm (bejahend)) Ähm die dann fehlen würde. Also von daher, das wäre sicherlich ein großer Vorteil. #01:02:24-6#

Interviewerin: Also das man damit quasi so ’n bisschen variieren kann, wie viel Kraft ich- #01:02:31-1#

Teilnehmer 15: Ich konzentriere mich dann auf die Dinge die ich äh dann mache und nicht darauf den, die&die&die Hand offenzuhalten oder den Ellebogen hochzuheben. #01:02:36-6#

Interviewerin: Ok. Weil halt immer irgend ‘ne Bewegung notwendig ist, (Teilnehmer 15: Ja.) um das zu machen und dann der Kopf bei der Bewegung ist? #01:02:43-9#

Teilnehmer 15: Genau. #01:02:45-0#

Interviewerin: Mhm (bejahend). Und gibt’s so an dieser idealen Prothese jetzt noch irgendwas, was Ihnen beiden einfällt, was die- #01:02:51-8#

Teilnehmer 25: Ähm ja, dass ähm der Schaft sag ich jetzt mal, sehr flexibel ist auch, dass man da gut rein- und rauskommt, dass er im Sommer nicht zu warm ist und im Winter nicht zu kalt (Interviewerin: Mhm (bejahend)). Ähm das weiß ich auch von vielen äh so, dass ähm der wirklich dann total zu kalt wird oder so (Interviewerin: Mhm (bejahend)) und im Sommer ähm schwitzt man da halt so, ähm so enorm so, dass die auch hält, auch wenn's warm ist. (Interviewerin: Ja.) Ähm und dann nicht abflutscht. Und auch sag ich jetzt mal, dass die einfach anzulegen ist. Jetzt ohne was weiß ich, so 'ne Art Gleitgel oder was weiß ich was man da braucht, um da reinzuflutschen (Interviewerin: Mhm (bejahend)) und so weiter. Und, dass sie vielleicht auch jetzt nicht enorm so steif ist jetzt, wenn man da mit 'm Ellenbogen rein muss. #01:03:44-9#

Interviewerin: Mh, was heißt das? Das heißt, dass der Schaft nicht so steif ist? #01:03:46-0#

Teilnehmer 25: Ja. #01:03:47-4#

Interviewerin: Ok. Das heißt, man muss einfach reinkommen, fest drinsitzen und es muss eben bequem sein bei allen Temperaturen. #01:03:54-5#

Teilnehmer 25: Ja. #01:03:56-4#

Interviewerin: Ok. Und Sie haben grade so geguckt, als gäb 's da auch noch? Ist das Zustimmung oder noch 'n Punkt? #01:03:59-7#

Teilnehmer 15: Ja, ja. Also ähm ich, meine Prothese lagert immer im äh Schlafzimmer und bei den Temperaturen ist es morgens schon unangenehm kalt, ja. (Interviewerin: Mhm (bejahend)) Und das war bei diesem früheren Netz, wenn ich das richtig in Erinnerung habe nicht ganz so. (Das ist ja?) Struktur. #01:04:15-9#

Interviewerin: Das heißt eben auch, dass es halt warmgehalten wird, oder? #01:04:20-5#

Teilnehmer 15: Ja, dass der äh das führt zuerst dazu, dass der, die Prothese nicht warm und der Arm kalt wird. (Interviewerin: Ja.) Und das ist doof. #01:04:25-6#

Interviewerin: Ja. Und sonst? #01:04:31-8#

Teilnehmer 15: Das ist glaub ich auch nicht nötig. #01:04:26-7#

Interviewerin: Ja. Sollte es nicht sein (lacht). Und sonst aber vom Tragekomfort, sowas wie weiß nicht Druckstellen oder sowas, ist das 'n Punkt? #01:04:35-1#

Teilnehmer 15: Nein. #01:04:34-0#

Interviewerin: Gar nicht, ok. Ja. (Protokollant schiebt der Interviewerin einen Zettel zu, Interviewerin wendet sich an Protokollant) Ähm das musst du mir glaub ich nochmal erklären? #01:04:44-3#

Protokollant: Ach so, ähm (wendet sich an Teilnehmer 15) Sie haben vorhin davon

gesprochen, dass Sie ähm ja, dass wenn man v-&mit einem Impuls arbeiten könnte, dass das vielleicht etwas vereinfachen würde, wenn ich's richtig verstanden habe? #01:04:57-6#

Teilnehmer 15: Ja. #01:04:57-3#

Protokollant: Ähm was dann wahrscheinlich wegfallen würde, wäre dann die Rückmeldung des Bowdenzugs über die Griffkraft, wie ist das so? #01:05:07-3#

Teilnehmer 15: Ähm also wenn ich eine&etwas baue, mache, ‘ne handwerkliche Tätigkeit und ich sage einfach als Impuls, das muss passieren und das passiert dann ohne, dass ich mich darauf konzentriere, dass es passiert (Protokollant: Ok, mhm (bejahend)), ist es für mich einfacher, mich auf die Dinge zu konzentrieren, die ich dann damit mache, als wenn ich dann die ganze Zeit die Prothese in ‘ner bestimmten Position halten und unter Spannung halten muss. (Protokollant: Mhm (bejahend), verstehe.) Das ist blöd. #01:05:37-9#

Teilnehmer 25: Dazu auch, dass man, wenn man zum Beispiel grad irgendwie ‘n Impuls gegeben hat, also wenn man jetzt mal, wir zu den Tatsachen kommen, die heutzutage möglich wären, jetzt mal ähm, wenn man jetzt irgendwie ‘n Impuls grad gibt mit Muskelanspannung, dass man den nicht unbedingt halten muss, sondern dass die Prothese dann eben halt weiß, er soll's jetzt das eben halt mal machen, dann so belassen und dann ähm wenn man die dann ändern will, dann eben halt irgendwie ‘n anderen Impuls geben kann, (Teilnehmer 15: Genau.) also vor allem für mich ist es dann glaub ich auch schwierig, wenn ich dann beide dann gleichzeitig bedienen will, zum&um was dann zu machen, zum Beispiel wir nehmen jetzt mal ich will was schneiden und (..?) was festhalten, müsst ich ja mit der einen Hand das ähm den Befehl sozusagen geben, dass ähm er's zudrücken soll ähm und mit der anderen eben halt, dass er's Messer halten soll (Interviewerin: Mhm (bejahend)) und dann, dass dann noch dann die Arme sozusagen dann noch zu bewegen dann dabei, ist glaub ich ‘ne enorme Kopfsache, so dass man dann wirklich sagen kann: „Halt das fest, halt das fest und dann mach.“. #01:06:39-6#

Interviewerin: Also, dass die sich so ’n bisschen einfach merken kann (Teilnehmer 25: Ja.), was die grade tun soll und dann einfach erst wieder drauf reagiert (Teilnehmer 25: Ganz genau.), wenn sie was anderes machen soll? #01:06:47-7#

Teilnehmer 25: Ganz genau. #01:06:48-6#

Interviewerin: Ja, ok. Ähm und dann würd‘ ich gerne noch wissen, das haben Sie schon so ’n bisschen angesprochen, ähm damit dass man ‘ne Rückmeldung darüber bekommt, was man eigentlich, also was man da eigentlich grade macht, wie viel Kraft man grade aufwendet. Da würd‘ ich gern wissen, wie ist es denn mit sowas, dass die, dass man mit der Prothese auch was fühlen kann. Dass man halt irgend so ein Feedback bekommt, also einerseits eben, dass man, dass man fühlen kann ‘ne Beschaffenheit von irgendwas, ‘ne Struktur und andererseits halt eben so ‘ne Rückmeldung, das haben Sie schon gesagt Herr (Name von Teilnehmer 25 aus Datenschutzgründen ausgelassen), dass es ‘ne Rückmeldung darüber wie stark ich irgendwas festhalt- #01:07:19-8#

Teilnehmer 25: Sind wir jetzt in was möglich wäre oder was ähm vorstellbar ist?

#01:07:26-7#

Interviewerin: Was vorstellbar-. Also ob sowas wichtig ist, ob sowas ‘n Punkt ist, den’s gäben sollte und in welcher Form, was da besonders wichtig ist. Dass man irgendwas an Rückmeldung von der Prothese bekommt und welche das vor allem sein sollte. #01:07:38-5#

Teilnehmer 25: Ich glaube jetzt das kommt ja darauf an, auf was für einen Beruf. (...?) jetzt bei Herr (Name von Teilnehmer 15 aus Datenschutzgründen ausgelassen) ist es so, dass vielleicht auch ähm, dass es glatt s-, also ob die Oberfläche eben halt glatt oder porös ist, oder was weiß ich, was ich mir auch denke, was sinnvoll wäre zum Beispiel also kalt und warm zu f-&zu erfühlen, oder was weiß ich. Ähm ja und es könnte wahrscheinlich auch nicht schaden, wenn man eben halt ähm fühlen könnte ähm wie die ähm Oberflächeneigenschaft dann ist, ob eben rau, oder was weiß ich dann. #01:08:26-8#

Interviewerin: Mhm (bejahend). Und eben, und sowas, haben Sie vorher gesagt, ist auch wichtig, dass man halt fühlen kann oder irgend ‘ne Rückmeldung bekommt, wie fest ich zudrücke, wie viel Kraft da drin ist. #01:08:38-3#

Teilnehmer 25: Ich glaube das müsste man nicht selbst unbedingt wissen, sondern die Prothese sollte das (Interviewerin: Ok.) ähm wissen können. Also so dass man, ähm zumindest, dass die Prothese dann sozusagen merkt: „Ok, da ist was, jetzt nicht weiterdrücken.“, so. (Interviewerin: Ja.) Aber wenn man selber will, dass ähm weitergedrückt ist, dass man dann eben nochmal ‘n Impuls gibt oder so. #01:08:59-5#

Interviewerin: Mhm (bejahend), ok. Das heißt ich muss gar kein Feedback bekommen, sondern einfach die muss halt wissen und- #01:09:05-5#

Teilnehmer 25: Ja, also ich glaub (Interviewerin: Mhm (bejahend)) bei denen ähm, bei der Eigenschaft braucht man nicht unbedingt ‘n Feedback zu bekommen. #01:09:12-4#

Interviewerin: Mhm (bejahend) und andererseits würde das ja heißen, dass ich halt immer hingucken muss, wann hört die auf und halt hingucken muss, muss ich, also will ich nochmal mehr oder nicht, wenn ich ‘n-& also wäre das nicht was, was stört, wenn ich halt jedes Mal, weil ich muss ja dann jedes Mal selber drauf achten, ob ich jetzt mehr drück, oder nicht? Oder wär‘ sowas egal? #01:09:28-1#

Teilnehmer 25: Glaub ich eher nicht. #01:09:31-6#

Interviewerin: Mhm (bejahend) #01:09:31-6#

Teilnehmer 25: Weil Sie ja weiß dann sozusagen, dass ähm, sie merkt ja ob dann was ist und dann hä-&hätte sie’s ja auch in der Hand (Interviewerin: Ja.) also ähm ich glaube dieses ist dann zum Beispiel, wenn man was jetzt zerknüllen will oder was weiß ich (Interviewerin: Mhm (bejahend)), würde, oder zerdrücken. Dann wird meine Dose oder was weiß ich, die Hand will ja merken da ist ‘ne Dose also ähm ah ich will die dann eben halt zerdrücken (Interviewerin: Mhm (bejahend)) und dann geb‘ ich halt der Prothese dann nochmal den Impuls sozusagen, dass sie dann mehr

Kraft aufwenden soll. #01:09:57-5#

Interviewerin: Ok, ja. Das heißt das Wichtige für Sie wäre, dass man einfach irgendwas fühlen kann, dass man halt wirklich irgendwas eben die Temperatur, die Beschaffenheit, dass man so ‘ne Rückmeldung bekommt? #01:10:07-1#

Teilnehmer 25: Ja. #01:10:05-4#

Interviewerin: Mhm (bejahend) #01:10:09-5#

Teilnehmer 15: Ja, ich bin da wirklich überfordert (lacht). #01:10:09-2#

Interviewerin: Ja. #01:10:12-0#

Teilnehmer 15: Äh, kann ich mir gar nicht vorstellen. #01:10:13-8#

Interviewerin: Mhm (bejahend) #01:10:15-2#

Teilnehmer 15: Ähm schön wäre es natürlich, ähm aber ich glaube bei mir würde das nicht so ‘ne große Rolle spielen. #01:10:21-1#

Interviewerin: Mhm (bejahend), das heißt dass- #01:10:20-2#

Teilnehmer 15: Ich sehe was ich mache, vielleicht deswegen. #01:10:24-4#

Interviewerin: Ja. Und aber wär‘ das zum Beispiel in der Arbeit in gewisser Weise irgend ‘ne Entlastung, dass man halt nicht jedes Mal hingucken muss und zum Beispiel weiß: Hab ich das grade noch in der Hand, oder nicht? Sowas was Sie grade gesagt haben mit sowas je weniger ich mich halt konzentrieren muss auf das, was ich mach ähm und weiß, was für ’n Impuls ich geb‘, desto einfach ist es? #01:10:43-2#

Teilnehmer 15: Mhm (bejahend) #01:10:46-4#

(kurze Pause) #01:10:47-9#

Teilnehmer 15: Ah das sind schon die lustigsten Dinge passiert. Ähm an einem Tag ‘ne Badehose gesucht und die hatt‘ ich dann in der Hand, hab’s nicht mitgekriegt (Interviewerin: Mhm (bejahend)), solche Sachen. Ähm äh also mir kommt es glaub ich schlüssig vor (Interviewerin: Mhm (bejahend)), wenn er meint, dass die ähm Sache über Impulse (Interviewerin: Mhm (bejahend)), die Festigkeit über Impulse, äh dass man zwei Impulse einfach die Kraft verstärkt, solche Sachen. Ähm das kommt mir glaub ich, dass käme mir glaub ich entgegen. #01:11:17-5#

Interviewerin: Ok das heißt aber erst- #01:11:19-8#

Teilnehmer 15: Ohne zu fühlen, (Interviewerin: Mhm (bejahend)) zu sehen was passiert. #01:11:21-6#

Interviewerin: Ja. Ok. Das heißt eher so ‘ne Art Muster, dass ich halt weiß ich drück einmal (Teilnehmer 15: Ja.) das ist so, ich drück zwei Mal das ist so? #01:11:24-4#

Teilnehmer 15: Ja ja, genau. Mhm (bejahend) #01:11:24-9#

Interviewerin: Mhm (bejahend) #01:11:25-6#

Teilnehmer 15: Mhm (bejahend) #01:11:28-0#

Interviewerin: Ok und das an Rückmeldung, liegt, also würden Sie sagen das kann ich mir einfach nicht vorstellen wie sowas funktioniert, wie ich sowas zurückbekommen soll? #01:11:32-6#

Teilnehmer 15: Mhm (bejahend) #01:11:34-2#

Interviewerin: Ok. Das heißt es ist nicht mal das, dass es vielleicht nicht nötig ist, oder nicht wichtig, sondern einfach- #01:11:37-5#

Teilnehmer 15: Nee, nee. #01:11:40-3#

Interviewerin: Nee? Mhm (bejahend) #01:11:40-4#

Teilnehmer 15: Ähm das ist tatsächlich so, dass ich äh, dass man nach 35 Jahren einfach kaum noch Vorstellungen darüber hat, wie das sein könnte. #01:11:49-1#

Interviewerin: Mhm (bejahend) #01:11:48-5#

Teilnehmer 15: Dass es nicht so wie rechts ist, ist mir klar. #01:11:51-4#

Interviewerin: Ja. #01:11:52-7#

Teilnehmer 15: Ähm, hab‘ ich mich auch wirklich noch nie mit beschäftigt (Interviewerin: Mhm (bejahend)), also wäre interessant, klar. (Interviewerin: Mhm (bejahend)) #01:11:59-5#

Teilnehmer 25: Jetzt die Frage hab‘ ich mich eben auch grade ähm gestellt, wie das irgendwie rein technisch möglich ist so, aber ähm wir gehen jetzt ja in der Science-Fiction aus, dass alles hier möglich ist (Interviewerin: Mhm (bejahend)) ähm irgendwie aber wenn auch definitiv ohne OP oder was weiß ich so, (Interviewerin: Mhm (bejahend)) sondern so Schwups, ist es da. Aber das wird glaub ich nie möglich sein, weil also ich würde mich für ‘ne Prothese nie operieren lassen, oder was weiß ich. (Interviewerin: Mhm (bejahend)) Weil da sind ja schon so viele Sachen fehlgelaufen, Beispiel dass dann eben halt die Elektroden dann eben halt während der Autofahrt dann eben halt angefangen haben zu brennen (Interviewerin: Mhm (bejahend)) und der dann eben halt innerlich ver-&dann auch dann gebrannt hat, dann eben halt und dann eben halt ‘n Unfall gebaut hat, dann auch noch andere Menschen gefährdet dadurch. (Interviewerin: Mhm (bejahend)) Und eben halt da weil er auch umgekommen ist so, dass seh‘ ich unvorstellbar. (Interviewerin: Ja.) #01:12:50-3#

Interviewerin: Das heißt es, also egal was sie kann und was auf einen zukommt halt, es darf nicht in den Körper eingreifen? #01:12:55-6#

Teilnehmer 25: Nee, also das nicht. Also mit Sensoren irgendwie was weiß ich so dran, dass ist in Ordnung so, aber mehr nicht. #01:13:02-5#

Interviewerin: Ja, ok. Ähm ja, dann war's tatsächlich schon fast, wir sind ziemlich schnell durchgekommen. Ähm was ich gerne jetzt zum Abschluss noch wissen wollen würde, einfach wir haben jetzt ganz viel gesprochen über die Vorteile, über die Nachteile, darüber warum Sie sich für die Prothese entschieden haben, ähm was verbessert werden sollte. Gibt's noch irgendwas, was Ihnen einfällt, was noch wichtig ist, was wir vielleicht vorher wo dann, weiß ich nicht, wo wir weitergegangen sind und der Gedanken kam erst danach, irgendwas, was es noch zu ergänzen gibt zu dem, was wir vorher besprochen haben? #01:13:37-8#

(kurze Pause) #01:13:43-3#

Teilnehmer 15: Nee, von meiner Seite aus fällt mir grad nichts ein. (Interviewerin: Mhm (bejahend)) #01:13:51-5#

Teilnehmer 25: Nicht direkt jetzt. Sie haben ja vorhin gemeint so was ähm eben halt Vorteile für Herrn (Name von Teilnehmer 15 aus Datenschutzgründen ausgelassen) eben halt mit der Myoelektrischen geben könnte. Ich glaube da wär‘ für Sie vor allem der Vorteil, wenn Sie eben halt den Ellenbogen irgendwie benutzen könnten oder steuern könnten sozusagen. #01:14:11-6#

Teilnehmer 15: Das wäre ‘n Vorteil, ja. #01:14:10-3#

Teilnehmer 25: Dass man dann eben halt wirklich den hoch oder runter so machen kann dann (Teilnehmer 15: Ja.) aber damit, ich kann auch jetzt nicht sagen wie gut da mittlerweile die Technik oder so. (...?) ich eben halt eben noch nie beschäftigt hab (Teilnehmer 15: Mhm (bejahend)) weil ich ja eben halt noch beide hab eben halt (Interviewerin: Mhm (bejahend)) ähm deswegen interessiert's mich jetzt sozusagen nicht ganz (Teilnehmer 15: Ja, Ellebogen wär gut.) (…??). #01:14:33-1#

Interviewerin: Mhm (bejahend). Genau, wenn wir vielleicht nochmal so von der andren Seite draufgucken. Was ist denn besonders wichtig, eben was wir jetzt so besprochen haben? Vielleicht an ‘ner Veränderung die besonders, die Ihnen besonders am Herzen liegt, wo Sie nochmal so sagen würden, dass ist was, was ich heute nochmal unterstreichen möchte als so wichtigsten Punkt oder einfach ‘n sehr wichtigen Punkt? #01:14:55-5#

Teilnehmer 25: Dass die Gewichtsverteilung besser wird (Interviewerin: Mhm (bejahend)), irgendwie dass man vielleicht die Akkus irgendwie dünner machen kann und dann was weiß ich, mit in den Schaft irgendwie, der dann eben halt rumkommt (Interviewerin: Mhm (bejahend)) eben halt reinbaut, was weiß ich. Und dann, mir ist eben auch eingefallen, wenn ich die dann an der linken Hand hab, werd‘ ich ja wahrscheinlich auch dann nochmal irgendwie ‘n, ich würde mal schätzen zehn Zentimeter Minimum, (Interviewerin: Mhm (bejahend)) weil die Hand ist vielleicht 15 Zentimeter lang oder keine Ahnung, was 20 Zentimeter längeren Arm ja wahrscheinlich haben, jetzt mal grob gesagt. Wegen dem Akku dann noch, der wahrscheinlich dann hier sitzt und dann kommt wieder noch die Hand und das ist ja enorm viel mehr Platz, das wär‘ jetzt sozusagen fast bis zum Mikrofon jetzt dann (Interviewerin: Mhm (bejahend)). Dass man das dann eben wirklich jetzt fast auf null

bringt und dann (Interviewerin: Mhm (bejahend)) direkt hier jetzt bei, wo jetzt ja eigentlich die Handwurzelknochen wären, (Interviewerin: Mhm (bejahend)) (nur?) eben halt die Handwurzel fast beginnen würde schon (Interviewerin: Mhm (bejahend)). Weil sonst hat, ist man ja auch von den Proportionen komplett raus (Interviewerin: Ja.) eigentlich, finde ich. #01:15:51-0#

Interviewerin: Ok, das heißt, dass die Gewichtsverteilung so ist, dass es eben nicht vorne an der Prothesenhand ist, sondern möglichst ähm einfach am Körper? #01:15:56-6#

Teilnehmer 25: Ja, das auf jeden Fall (Interviewerin: Mhm (bejahend)) und auch, dass man eben halt wie jetzt in diesem Fall geschildert, grade eben halt auch ähm die Hand relativ dann, so viele zum Beispiel ich meine wenn irgendwie die Hand weggesprengt wird, oder was weiß ich (Interviewerin: Mhm (bejahend)), sich durch ’n Böller oder so, sieht meistens die Hand so aus wie meine Linke dann bei denen oder so (Interviewerin: Mhm (bejahend)). Dass dann die Hand wirklich fast dann ähm hier vorn dann gleich ist (Interviewerin: Mhm (bejahend)). So dass man eben halt nicht (Interviewerin: Mhm (bejahend)) dann noch die 30 Zentimeter länger dann hat, weil 15, oder keine Ahnung wie lang das ist (Interviewerin: Mhm (bejahend)) sondern relativ gleich hier dann die Hand hat. #01:16:34-4#

Interviewerin: Ok, das heißt würden Sie, also würden wir uns jetzt ‘ne Person vorstellen, die den Arm hatte, ‘n Unfall hat und der ist dann nicht mehr da. Das heißt, sagen wir es fehlt nicht nur die Hand bei der Person, sondern eben auch ‘n Stück vom Arm. Wär’s dann, würden Sie dann trotzdem die Hand so einbauen, dass die einfach direkt quasi am Körper aufhört, oder würden Sie das fehlende Stück auch noch durch die Prothese ersetzen? (Teilnehmer 25: Ähm das würd‘ ich ähm ersetzen, allein aus dem Grund, weil man sonst wieder Rückenprobleme kriegen könnte, weil man das dann automatisch ausgleicht, eben halt die Länge.) #01:17:03-1#

Interviewerin: Ok, das heißt schon so, wie’s normal ist, aber halt nicht übermäßig nach&lang, (Teilnehmer 25: Ja, ganz genau.) nicht länger als es normal ist? Mhm (bejahend), ok. Und bei Ihnen Herr (Name von Teilnehmer 15 aus Datenschutzgründen ausgelassen), gibt’s noch irgendwas, was besonders wichtig ist? Was Ihnen so ‘ne Sache ist, die besonders am Herzen liegt? #01:17:16-0#

Teilnehmer 15: Ich kann das nur nochmal zusammenfassen. #01:17:18-8#

Interviewerin: Mhm (bejahend) #01:17:18-8#

Teilnehmer 15: Also atmungsaktiv (Interviewerin: Ja.) finde ich klasse, ähm vernünftige Gewichtsverteilung seh‘ ich genauso, ähm (kurze Pause) und für mich wäre es wichtig, sie auseinander nehmen zu können (Interviewerin: Mhm (bejahend)). Ohne irgendwelche Spezialmittel oder Nieten aufzutrennen, oder solche, solche Geschichten. Ähm grade, wenn man Kundenkontakt hat ist es schwierig, wenn das nicht möglich ist. #01:17:44-3#

Interviewerin: Aus hygienischen, also- #01:17:45-8#

Teilnehmer 15: Ja, aus hygienischen Gründen. #01:17:46-6#

Interviewerin: Ja, ok. Mhm (bejahend) #01:17:48-9#

Teilnehmer 15: Und ähm dann sollte sie wie auch immer robust sein. #01:17:55-6#

Interviewerin: Ja. #01:17:53-7#

Teilnehmer 15: Ähm ja und das was ich damit mache, sollte kalkulierbar sein. #01:18:02-6#

Interviewerin: Mhm (bejahend) #01:18:03-4#

Teilnehmer 15: Deswegen war das mit diesen (...?) Impulsen (Interviewerin: Mhm (bejahend)), fand ich ‘ne ganz interessante Geschichte, ja. Ähm (kurze Pause) genau wenn sie darüber hinaus äußerlich noch einigermaßen korrekt daherkommt, hätt ich da nichts gegen (lacht). #01:18:19-2#

Interviewerin: Ja. Und nochmal kurz dieses kalkulierbar, das heißt es ist gar nicht mal so das Wichtige, dass&dass sie das quasi blind macht, (Teilnehmer 15: Nein.) sondern dass man halt einfach einschätzen kann was sie da macht? #01:18:28-6#

Teilnehmer 15: Ja. #01:18:30-0#

Interviewerin: Mhm (bejahend), ja, ok. Gut, ja wenn’s das war, dann ähm ist das Gespräch eigentlich erstmal vorbei.
